# Assessing the Sterile Insect Technique in Small, Non-Isolated Urban Areas: A Field Trial Against *Aedes albopictus* in Southern Switzerland

**DOI:** 10.3390/insects17080793

**Published:** 2026-07-31

**Authors:** Diego Parrondo Monton, Damiana Ravasi, Nikoleta Anicic, Mariantonietta Lettieri, Pietro Storelli, Arianna Puggioli, Nisia Trisconi, Matteo Tanadini, Eleonora Flacio

**Affiliations:** 1 Institute of Microbiology, Department for Environment Constructions and Design, University of Applied Sciences and Arts of Southern Switzerland (SUPSI), Via Flora Ruchat-Roncati 15, 6850 Mendrisio, Switzerland; diego.parrondo@supsi.ch (D.P.M.); nikoleta.anicic@supsi.ch (N.A.); mariantonietta.lettieri@supsi.ch (M.L.); pietro.storelli@supsi.ch (P.S.); eleonora.flacio@supsi.ch (E.F.); 2 Centro Agricoltura Ambiente “G. Nicoli”, Via Sant’Agata 835, 40014 Crevalcore, Italy; apuggioli@caa.it; 3 Zurich Data Scientists GmbH, Sihlquai 131, 8005 Zurich, Switzerland; nisia.trisconi@zurich-data-scientists.ch (N.T.); matteo.tanadini@zurich-data-scientists.ch (M.T.)

**Keywords:** *Aedes albopictus*, distance-based analysis, egg hatching rate, integrated vector management, mosquito surveillance, quasi-experimental design, sterile insect technique (SIT), urban ecology

## Abstract

The sterile insect technique (SIT) is considered a promising and environmentally sustainable tool for controlling *Aedes albopictus*. While current guidelines address its implementation in moderately large and partially isolated areas, evidence from small, fully urban and spatially connected environments remains limited. In 2025, we evaluated SIT in two municipalities in southern Switzerland, releasing sterile males in small (≈12 ha), non-isolated urban areas representing contrasting operational contexts within ongoing integrated vector management programs. Entomological responses were assessed using a spatial, distance-based framework integrating ovitrap-derived egg abundance and hatching rates from treated and surrounding zones, alongside adult female monitoring. Following releases, egg abundance declined within treated areas relative to surrounding zones, with model-based peak reductions of approximately 57% in Ascona and 67% in Losone. Egg hatching rates were also lower within treated areas, with estimated reductions of approximately 9% and 22%, respectively. For both indicators, spatial analyses revealed gradual distance-dependent patterns rather than abrupt boundary effects. No consistent pattern was detected for adult female abundance, likely reflecting limited sampling intensity. The spatial and temporal responses observed across the two study sites suggest that SIT may contribute to modifying local reproductive dynamics of *Ae. albopictus* even in small, non-isolated urban areas under operational conditions.

## Simple Summary

Urban mosquito control is challenging because cities are densely built and highly connected. The Asian tiger mosquito (*Aedes albopictus*) causes strong nuisance and can spread diseases such as dengue. An environmentally friendly approach, the sterile insect technique, works by releasing sterile male mosquitoes that mate with wild females, which lay eggs whose embryos fail to develop. We tested this method in two very small urban neighborhoods in southern Switzerland where routine mosquito control was already in place. Areas with sterile male releases produced fewer eggs and fewer viable eggs than nearby areas, and these effects gradually decreased with distance. No clear reduction in adult mosquitoes was detected, likely due to limited trapping. Overall, this study suggests that the sterile insect technique may provide added benefits to mosquito control programs even in small urban environments.

## 1. Introduction

Mosquito-borne arboviruses such as dengue, chikungunya, Zika, and yellow fever continue to expand globally, driven by the ecological adaptability and ongoing geographical spread of the highly competent vectors *Aedes* (*Stegomyia*) *aegypti* (Linnaeus) and *Aedes albopictus* (Skuse) [1,2,3]. The intensification of urbanization and climate change, together with the widespread use of chemical biocides in traditional control programs, has contributed to increasing outbreak frequency, highlighting the need for innovative and environmentally sustainable vector control strategies [4,5]. Among these, the sterile insect technique (SIT) has gained growing attention as a promising complementary approach capable of suppressing *Aedes* populations without relying on insecticides and with minimal ecological impact [6,7]. Originally developed for agricultural pests in the mid-20th century, SIT has more recently been incorporated into *Aedes* control programs in several countries, including China, Singapore, Brazil, and the USA [8,9,10], where it has demonstrated considerable potential when implemented as part of an integrated vector management (IVM) strategy.

The effectiveness of SIT is directly linked to the reproductive biology of *Aedes* mosquitoes. Released sterile males compete with wild males for mating opportunities, inseminating females without producing viable offspring. Females mated with sterile males continue to oviposit, but the eggs laid fail to hatch due to early embryonic mortality. By reducing the proportion of viable eggs, SIT can attenuate, or even reverse, the natural seasonal population growth of *Aedes* populations, ultimately leading to lower adult densities over time [11]. In addition, high densities of sterile males may increase mating pressure on females, potentially resulting in behavioral stress and reduced activity [12].

To foster effective and ethically responsible implementation, international organizations such as the World Health Organization (WHO), the Food and Agriculture Organization (FAO), and the International Atomic Energy Agency (IAEA) have developed detailed guidelines describing a stepwise, evidence-based process for the development and evaluation of SIT programs [13,14]. This framework, known as the Phased Conditional Approach (PCA), outlines specific requirements for the transition from laboratory validation (Phase I) to small-scale field trials (Phase II) and, ultimately, to operational deployment (Phase III) [7,15]. Classical SIT guidance emphasizes that Phase II trials should be conducted under carefully selected conditions, typically in moderately sized areas (≈20–30 hectares) that are ecologically stable and preferably partially isolated, to limit mosquito immigration and facilitate clear attribution of observed entomological effects to sterile-male releases. In addition, Phase II evaluations commonly rely on comparisons between treated areas and geographically separate, untreated control sites to distinguish SIT-induced effects from background population dynamics [7,14]. Standardized monitoring protocols using oviposition traps (ovitraps) and adult mosquito traps are further recommended to quantify changes in egg production, egg hatching rate, female abundance, and sterile-to-wild male ratios.

While this structured approach has proven effective in multiple settings [8,10], it also raises an operationally relevant question: how does SIT perform under conditions that fall outside the conservative assumptions of current implementation guidelines? In contemporary urban environments, suitable intervention areas are often small, densely populated, and fully connected to surrounding neighborhoods, making it difficult to identify sites that meet classical Phase II selection criteria. In particular, ecologically comparable and operationally independent control areas may be difficult to identify in highly connected urban systems, where environmental heterogeneity and mosquito movement across intervention boundaries are inherent features of the landscape. Understanding how SIT performs under these operational constraints is therefore important for informing public-health decision-making and for evaluating how existing implementation frameworks can be applied in realistic urban environments.

In 2023, we applied the PCA framework to design and implement a SIT pilot trial in the Canton of Ticino (Switzerland), following recommended site characteristics and area sizes [16]. That study demonstrated clear spatial and temporal patterns consistent with *Ae. albopictus* population suppression, including a 57% reduction in egg abundance and a 66% decrease in adult female abundance within the intervention area. Building on this experience, the present study was designed to evaluate SIT under conditions that more closely reflect the constraints commonly encountered in urban mosquito control programs. Specifically, sterile-male releases were implemented in very small (≈12 ha), non-isolated intervention areas embedded within a fully urban environment characterized by intense spatial connectivity.

Rather than relying on geographically separate control sites, we adopted a spatially explicit, distance-based analytical framework integrating pre-intervention baseline data with temporal and spatial gradients within and around the release areas. This approach was selected to account for the open and permeable nature of the study system and to explore whether patterns compatible with SIT activity could be detected under constrained operational conditions. More broadly, the study evaluates the usefulness of a distance-based framework for assessing SIT performance in urban environments where classical experimental designs may be difficult to implement.

## 2. Materials and Methods

### 2.1. Study Sites

**Figure 1:**
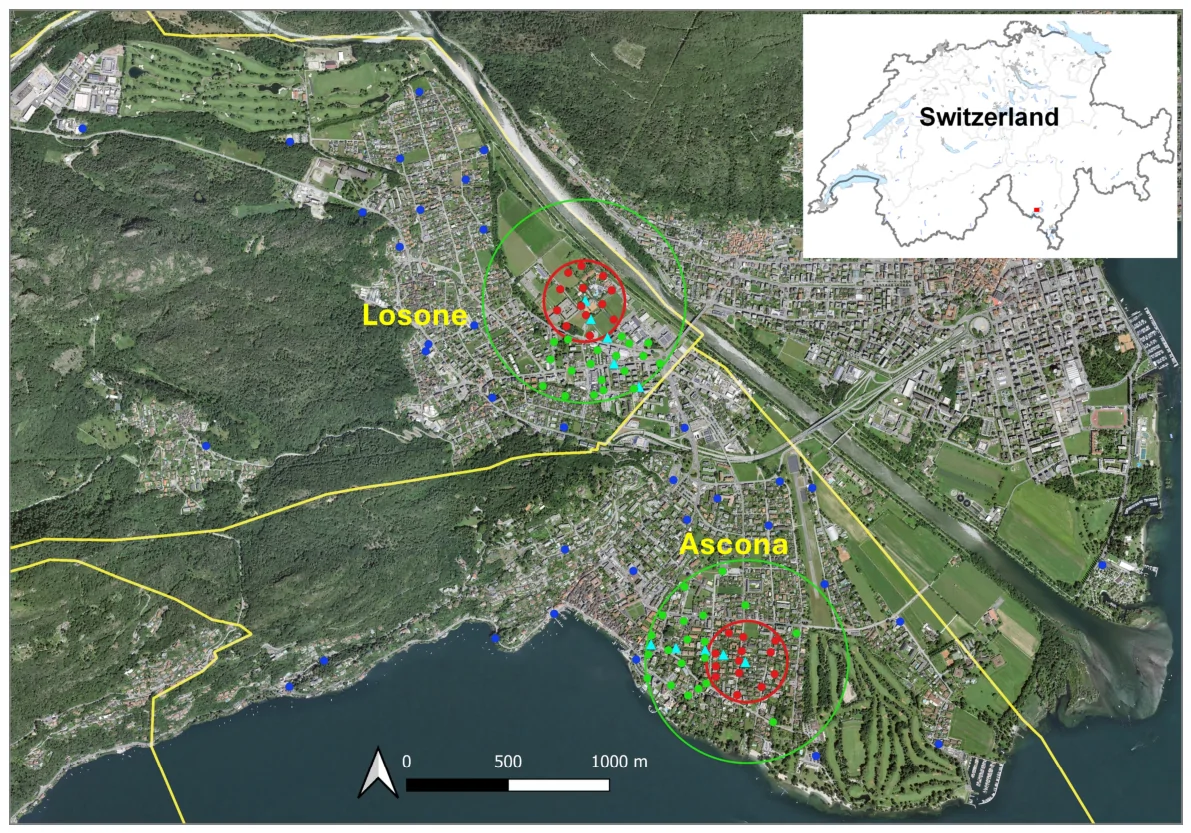
Study sites in Ascona and Losone. Red, green, and blue dots indicate ovitrap locations within the SIT-treated zone (red circle), the non-SIT buffer zone (green circle), and the non-SIT outside area, respectively. Adult mosquito traps are indicated by light blue triangles. The inset shows the location of the study sites in southern Switzerland. Base map imagery © Esri; map modified using QGIS v. 3.44.9.

The study took place in 2025. It was conducted in two municipalities of the Canton of Ticino in southern Switzerland, namely Ascona (46°09′20.52″ N, 8°46′07″ E, 196 m a.s.l., ≈1100 inhabitants/km^2^) and Losone (46°10′00″ N, 8°46′00″ E, 238 m a.s.l., ≈700 inhabitants/km^2^) (Figure 1). These neighboring municipalities form part of the Locarno urban agglomeration, a functionally interconnected urban area dominated by low-density settlements [17].

Both municipalities feature extensive residential areas with private gardens and interstitial green spaces, a settlement pattern typical of Canton Ticino and particularly favorable to the proliferation of *Ae. albopictus* [18,19]. The region experiences a mild Mediterranean-type climate, with warm summers, relatively mild winters, and among the highest annual sunshine levels in Switzerland, owing to its low elevation along Lake Maggiore [20,21].

*Aedes albopictus* became established in both municipalities in 2012, and by 2024 seasonal densities had reached relatively high levels, averaging approximately 100–200 eggs per ovitrap per sampling period, based on biweekly ovitrap collections from May to September. Both municipalities have been implementing an IVM program since 2010. This includes regular larviciding of public catch basins with VectoMax^®^ FG (Valent BioSciences, Libertyville, IL, USA) every eight weeks, combined with community-based source reduction [22,23,24]. Residents are encouraged to remove temporary water-holding containers and to treat permanent ones with the biological larvicide VectoBac^®^ G (Valent BioSciences).

Although both municipalities follow the same IVM framework, the two study sites were deliberately selected to represent contrasting operational scenarios in terms of urban structure and feasibility of preventive measures. The selected site in Ascona represents a challenging, worst-case scenario, being predominantly residential and characterized by numerous private gardens and a high proportion of secondary homes. The intermittent presence of property owners reduces familiarity with mosquito-prevention practices and results in inconsistent elimination of breeding sites. This setting is representative of densely built yet highly permeable urban areas where sustained *Ae. albopictus* control is particularly difficult to achieve.

In contrast, the selected site in Losone represents a more favorable, best-case operational scenario. The area is oriented toward tourism and public services and includes a hotel, sports facilities, and school buildings. In particular, the central hotel property systematically conducts breeding-site inspections and applies regular VectoBac^®^ G treatments, resulting in a context where recommended IVM measures can be implemented more consistently. This site is therefore representative of small private enterprises and sensitive service areas (e.g., schools and sports grounds) where the integration of SIT may be operationally relevant.

Both study sites were fully embedded within the surrounding urban matrix and were not geographically isolated. Routine municipal IVM activities continued unchanged throughout the study period, as the objective was to evaluate SIT performance across contrasting real-world operational contexts rather than under optimized control conditions.

### 2.2. Experimental Design

#### 2.2.1. Intervention Area

Sterile male *Ae. albopictus* were released within a 12-ha intervention area in each municipality. The SIT-treated zone was defined as a circular area with a radius of 200 m, selected to reflect the upper range of autonomous dispersal typically reported for *Ae. albopictus*. Dispersal distances of 100–200 m are commonly adopted as operational reference values by vector control programs in several European countries, including Italy, France, Spain, and Switzerland, particularly for delineating adulticiding perimeters during emergency responses to arbovirus introductions [25,26,27]. This spatial configuration was designed to maximize coverage of the intervention area by released sterile males while limiting, as far as possible under real urban conditions, the inward movement of wild mosquitoes from surrounding untreated areas. Although neither site was geographically isolated, the 200 m radius allowed the release core to be operationally defined and facilitated the analysis of spatial patterns in an open urban system.

#### 2.2.2. Release Period and Frequency

Based on available information on *Ae. albopictus* population densities in southern Switzerland and on estimates from comparable peri-urban settings, releases were planned at an intended rate of approximately 3000 viable sterile males per hectare per week. This release rate was considered consistent with mosquito abundance levels typically observed in residential areas of the Canton of Ticino and with operational overflooding ratios applied in previous SIT field trials against *Aedes* species [18,28,29,30]. This corresponded to a total of about 36,000 sterile males released per week for each 12-ha intervention area. The selected release rates were expected to maintain an effective sterile-to-wild male ratio in the range of approximately 5:1 to 10:1, which is generally considered necessary to induce substantial sterility in field populations. Because sterile-to-wild male ratios were not directly measured during the present study, this value should be interpreted as an operational expectation rather than a field-verified estimate. Sterile males were released across 18 evenly spaced release points to ensure a homogeneous spatial coverage of the entire site. A permit (BAFU-217.23-64640/2) for the release of sterile male *Ae. albopictus* in Ascona and Losone was granted by the Swiss Federal Office for the Environment, in accordance with the Ordinance on the Handling of Organisms in the Environment (No. 814.911). The releases were also authorized by the respective municipal authorities.

In order to maintain a continuous presence of sterile males throughout the peak activity period of *Ae. albopictus*, releases were conducted twice per week from mid-June (calendar week 25) to mid-September (calendar week 37) and once per week until the end of September (calendar week 40). Therefore, a total of 29 release events were carried out across 16 consecutive weeks. This release frequency was based on survival estimates derived from previous mark-release-recapture trials conducted in Morcote, Switzerland, which indicated a marked decline in sterile male longevity toward the end of the season [16].

Not all planned shipments resulted in successful releases owing to occasional delivery delays or failures (Table S1); in such cases, release numbers were increased to up to 4000 sterile males per hectare in the subsequent release event to partially compensate for missed releases. Also, releases had originally been planned to start in May but were delayed due to the timing of the release permit approval.

Throughout the study period, routine municipal IVM activities continued across the entire territory of both municipalities. These included regular larvicidal treatments of public catch basins with VectoMax^®^ FG, the provision of VectoBac^®^ G to residents for use on private properties, and ongoing public information campaigns on mosquito breeding sites and preventive measures. No additional or intensified control measures (e.g., door-to-door interventions or targeted inspections) were implemented specifically for this study, as the aim was to evaluate the SIT as an additional component integrated into the existing IVM framework. Although larvicidal products were made available to residents, no direct information was available on their actual use, application frequency, or level of awareness within the study areas, and household-level compliance was not verified.

#### 2.2.3. Entomological Monitoring

Entomological monitoring was conducted throughout the SIT intervention period at both study sites to quantify the effects of SIT on the *Ae. albopictus* population, focusing on three key parameters: egg abundance (standardized by ovitrap exposure time), egg hatch rate, and adult female abundance. Eggs and adult mosquitoes were collected using ovitraps and CO_2_-baited BG-Pro traps operated in sentinel mode, respectively. Egg hatch rate was calculated as the proportion of eggs that successfully hatched following a standardized laboratory hatching procedure based on FAO/IAEA protocols and applied as described in Parrondo Monton et al. [16]. The same procedures for embryonation, hatching, and assessment of unhatched eggs were used without modification. Detailed descriptions of trap design, laboratory procedures, species identification, and sex determination are provided in Parrondo Monton et al. [16].

Sampling was organized according to analytical zones defined by distance from the SIT release center, rather than by strictly isolated geographic units, in order to reflect the open and permeable nature of the urban landscape and the spatial structuring of *Ae. albopictus* populations. Because the study was conducted in an open urban system without geographically independent control areas, analyses were based on a quasi-experimental framework combining pre-intervention baseline data and spatial gradients across zones located at increasing distance from the release area. This approach was designed to evaluate spatial and temporal patterns potentially associated with sterile-male releases under operational conditions while accounting for the connectivity of the surrounding urban environment.

Because entomological monitoring was conducted through multiple sampling points distributed within and around the same intervention areas, individual ovitraps and adult traps represent sampling units within a spatially structured mosquito population rather than independent experimental replicates of the SIT intervention. Spatial and temporal dependence among observations was therefore accounted for during statistical analyses.

Three spatial zones were defined: the SIT-treated zone (within 200 m of the center, corresponding to the intervention area), a surrounding non-SIT buffer zone (200–500 m), and a non-SIT outside area beyond 500 m (Figure 1). This distance-based zoning is consistent with reported patterns of *Ae. albopictus* spatial activity in urban environments and allows assessment of spatial gradients in SIT effectiveness [18,31].

Oviposition data were obtained from two complementary sources: ovitraps deployed specifically for the SIT trial in 2025 (OTR-SIT) and ovitraps belonging to the routine municipal surveillance network (OTR-MON), which provided continuous data from both the pre-intervention year (2024) and the intervention year (2025). In the SIT-treated zone, 12 OTR-SIT were evenly distributed at an approximate density of one trap per hectare, complemented by one OTR-MON at each site (i.e., Ascona and Losone) to ensure continuity with the pre-intervention baseline. Detailed information on trap numbers and sample sizes by site, year, analytical zone, and trap network is provided in Table S2. In the non-SIT buffer zone, 15 OTR-SIT were installed at the Ascona site and 16 at the Losone site within a defined sector, reflecting operational constraints of the experimental design. Five OTR-MON in Ascona and three in Losone were also located within the buffer distance range. In Ascona, one OTR-MON was positioned within the same sector as the OTR-SIT, while the remaining traps were heterogeneously distributed across the surrounding urban area; in Losone, all three OTR-MON fell within the same sector as the OTR-SIT. No OTR-SIT were deployed in the non-SIT outside zone; however, OTR-MON located at distances greater than 500 m (Ascona: *n* = 19; Losone: *n* = 16) were used to characterize background temporal variability in oviposition activity, acknowledging their heterogeneous spatial distribution.

Adult female abundance was monitored using CO_2_-baited BG-Pro traps arranged along a linear transect extending from the SIT release center to approximately 500 m. At each study site, five traps were operated for 24 h per sampling event, with three traps located within the SIT-treated zone and two within the non-SIT buffer zone. This transect-based configuration was designed to explore potential spatial gradients in adult female abundance relative to the release core. Adult female data were analyzed primarily to explore potential trends, recognizing that the limited trap density could constrain inferential power at this spatial scale.

Egg abundance and egg hatch rate were assessed approximately every two weeks, in line with the routine municipal surveillance schedule. Adult female abundance was monitored with the same nominal frequency, although sampling dates did not always coincide exactly with ovitrap collections. Baseline (pre-intervention) egg hatch rate data were available for Ascona only, as systematic hatching assays were not performed in Losone in 2024; accordingly, temporal hatch-rate analyses for Losone were restricted to the intervention period and interpreted in relation to spatial gradients and within-season patterns.

#### 2.2.4. Production of Sterile Males

Sterile *Ae. albopictus* males used in this study were produced at the Centro Agricoltura Ambiente “G. Nicoli” (CAA, Crevalcore, Italy), following the same standardized mass-rearing, sex-sorting, and irradiation procedures described in detail in our earlier work [16]. Briefly, the colony originated from eggs collected in Ticino in 2019 and has since been maintained under controlled laboratory conditions. Male pupae were mechanically sex-sorted to achieve a residual female contamination below 1% and irradiated with 30 Gy using an X-ray irradiator. This dose differed from the 40 Gy used in the 2023 field trial [16] and was adopted following updated operational procedures aimed at improving the biological quality and field competitiveness of sterile males while maintaining an induced sterility rate above 99%. A full description of rearing protocols, sex-sorting procedures, irradiation settings, and quality-control metrics is available in our previous publication [16].

#### 2.2.5. Transportation and Release of Sterile Males

After irradiation and emergence, sterile males were cooled, packaged at approximately 1000 individuals per cup, and shipped from the production facility to Switzerland under temperature-controlled conditions to maintain quality during transport [16]. Transport was performed by courier for all shipments except on 19 June 2025, when the material was transported directly by car from the rearing facility. Shipments were typically received on site around 14:00 local time, with releases carried out within approximately one hour of arrival (Table S1). Sterile males were 2–4 days old at the time of release.

Releases were performed manually by two trained operators who moved between release points by car or by walk. The full release procedure required approximately one hour to complete. At each release station, cups were placed in suitable shaded locations in proximity to vegetation that provided favorable microclimatic conditions for male awakening and take-off, while minimizing the risk of ant predation (Figure S1).

The sterility level of released males had been previously assessed in laboratory trials, showing that females mated with irradiated males exhibited residual fertility below 1%. Mortality following transport was assessed at each release event: three cups were inspected at the receiving site, and the proportion of males failing to take off within one hour was recorded (Table S1). Individuals that did not awaken and fly within this interval were counted as dead.

#### 2.2.6. Statistical Analysis

Statistical analyses were designed to quantify temporal and spatial patterns in *Ae. albopictus* egg abundance, egg hatching rate, and adult female abundance. All analyses were conducted separately for the two study sites (Ascona and Losone) using R (version 4.5.1), primarily relying on the mgcv and mgcViz packages. Statistical significance was assessed at the 5% level.

Because the study was conducted in an open urban system without geographically independent control areas, analyses were based on a quasi-experimental framework integrating pre-intervention baseline data and spatial gradients across zones located at increasing distance from the release area.

Temporal dynamics were analyzed using Generalized Additive Mixed-Effects Models (GAMMs) to account for repeated measurements at individual traps. Egg abundance and adult female abundance were modeled using negative binomial distributions to accommodate overdispersion, whereas egg hatching rate was modeled as a binomial response using the numbers of hatched and unhatched eggs, with a quasi-binomial specification. All temporal models included analytical zone (SIT treated, non-SIT buffer, non-SIT outside) as a fixed effect, a zone-specific smooth term for sampling date (expressed as day of year), and trap identity as a random effect. For count-based responses, variation in trap exposure time was accounted for using the logarithm of exposure duration as an offset. Detailed model specifications and diagnostic procedures are provided in Files S1–S3.

To facilitate interpretation and comparison with previous SIT trials, model-based percentage reductions were calculated for egg abundance and egg hatching rate by comparing predictions for the SIT-treated zone with those for the non-SIT buffer and non-SIT outside zones, using predicted values at the seasonal peak for egg abundance and seasonal mean predicted values for egg hatching rate.

Spatial patterns were explored using Generalized Additive Models (GAMs), including a bivariate smooth of trap coordinates, allowing flexible modeling of spatial heterogeneity and gradual changes in entomological indicators with increasing distance from the release center. Spatial models included a smooth term for sampling date to account for seasonal variation and used the same response distributions as the temporal analyses. Offsets were applied to count-based responses as described above.

Model fit and complexity were evaluated using standard diagnostic procedures, including residual analyses, information criteria (AIC and BIC), and likelihood-based comparisons where appropriate. Detailed descriptions of model construction, selection procedures, diagnostics, and supplementary analyses are provided in Files S1–S3.

## 3. Results

### 3.1. Release Logistics and Post-Transport Male Survival

Detailed information on shipment success, transport duration, post-transport mortality, and viable release rates is reported in Table S1. Post-transport mortality of sterile *Ae. albopictus* males was relatively low and stable at the beginning of the release period (mid-June to early July 2025, calendar weeks 25–27), remaining below 13% in most events, with transport durations of approximately 24 h, except for one short direct delivery (≈3 h) associated with minimal mortality. From July to August 2025 (calendar weeks 28–34), mortality showed moderate variability (7–29%), with occasional increases associated with logistical issues, including two prolonged transport events (up to 48 h) and two failed deliveries. During this period, releases resulted on average in approximately 3500–4000 viable sterile males per hectare per week at each study site.

In late August and early September 2025 (calendar weeks 35–36), post-transport mortality increased moderately (23–30%). The second week of September was affected by major logistical disruptions, including one delayed shipment (up to 48 h) with exceptionally high mortality (77%) and one failed delivery. During the final three weeks of the intervention (mid to late September), the number of viable males released per hectare declined sharply, falling below 1000 males/ha in the final release events, with the most pronounced reduction occurring in the last week due to extended transport duration.

### 3.2. Egg Abundance Patterns

Temporal patterns in *Ae. albopictus* egg abundance were analyzed using GAMMs fitted separately for Ascona and Losone, with egg counts standardized by ovitrap exposure time. Predicted egg abundance in the SIT-treated zone was used as the denominator for ratios comparing egg abundance in the surrounding non-SIT buffer zone (200–500 m) and the non-SIT outside area (>500 m) with that in the SIT-treated zone. A ratio of 1 indicates equal predicted egg abundance between zones, while values below or above 1 indicate higher egg counts in the SIT-treated zone or in the comparison zone, respectively.

**Figure 2:**
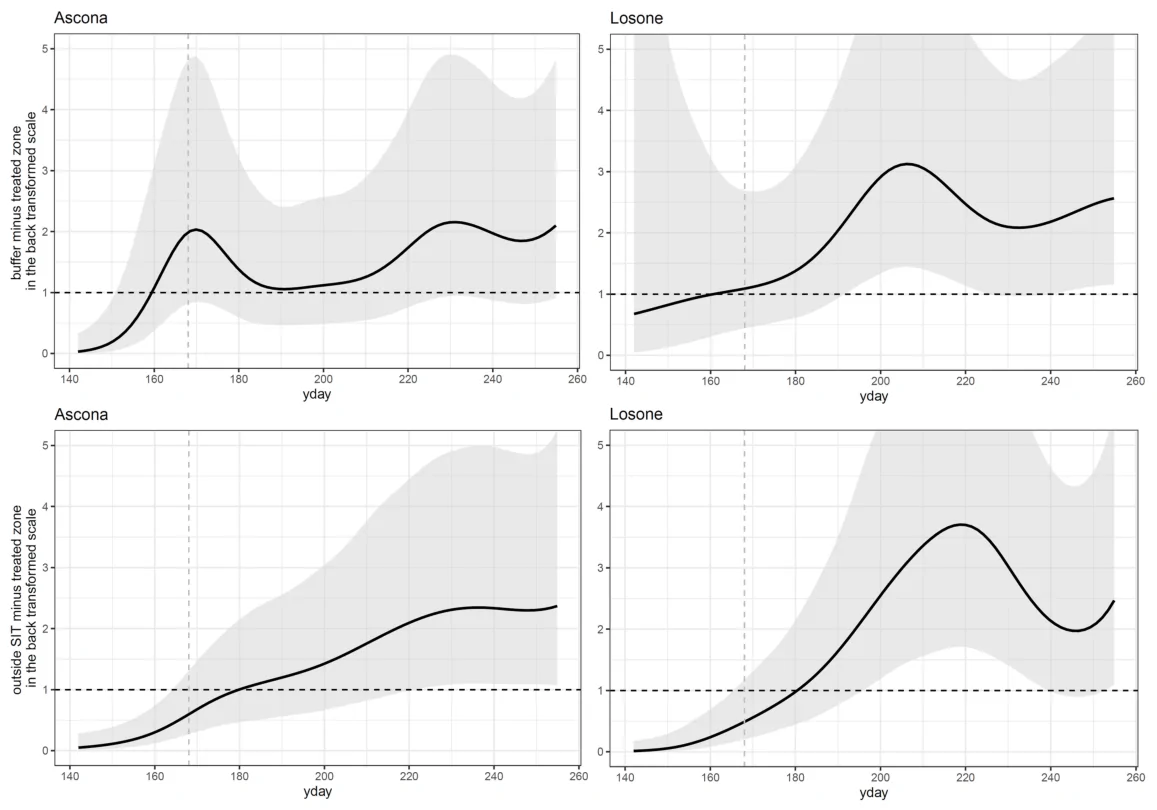
Ratios of model-predicted egg abundance between the non-SIT buffer zone (500 m radius) and the SIT-treated zone (**top panels**) and between the non-SIT outside area (>500 m) and the SIT-treated zone (**bottom panels**) for Ascona (**left**) and Losone (**right**). Curves represent ratios derived from GAMM model predictions, with shaded areas indicating 95% confidence intervals. A value of 1 (horizontal dashed line) indicates equal predicted egg abundance between zones; values greater than 1 indicate higher predicted egg abundance in the non-SIT buffer or outside zones relative to the SIT-treated zone, and values below 1 indicate the opposite. The vertical dashed line marks the start of sterile-male releases (17 June 2025). Ratios are considered statistically different from 1 when the confidence interval does not intersect the horizontal reference line. yday: Day of the year.

In both municipalities, clear temporal changes in these ratios were observed (Figure 2). Prior to the start of SIT releases (before day 168), the to-be SIT-treated zones showed higher predicted egg abundance than both comparison zones, with ratios consistently below 1. This pattern was particularly clear in Ascona, as indicated by confidence intervals entirely below 1, while greater uncertainty was observed in Losone.

Following the start of sterile male releases, the relationship progressively reversed in both sites, with predicted egg abundance becoming lower in the SIT-treated zones than in the surrounding zones and ratios exceeding 1 (Figure 2). This divergence increased over the course of the season and remained clearly separated from early-season patterns, indicating a sustained shift in relative egg abundance.

At the seasonal peak, model-based estimates indicated substantial reductions in egg abundance within the SIT-treated zones compared to surrounding zones. In Losone, predicted egg counts within the SIT-treated zone were approximately 67% lower than in the non-SIT outside area, while in Ascona the corresponding reduction was 57%. These peak values summarize the maximum model-predicted differences observed during the intervention period. The non-SIT buffer zones consistently exhibited intermediate egg abundances between SIT-treated and non-SIT outside zones, suggesting a spatial gradient in SIT effects rather than an abrupt boundary at the edge of the release area.

### 3.3. Egg Hatching Rate Patterns

Egg hatching rate showed clear and consistent patterns across both study sites and proved to be a particularly informative indicator [16]. In contrast to egg abundance and adult female counts, hatching rate exhibited relatively stable values outside the SIT-treated areas and well-defined responses within and around the release zones.

**Figure 3:**
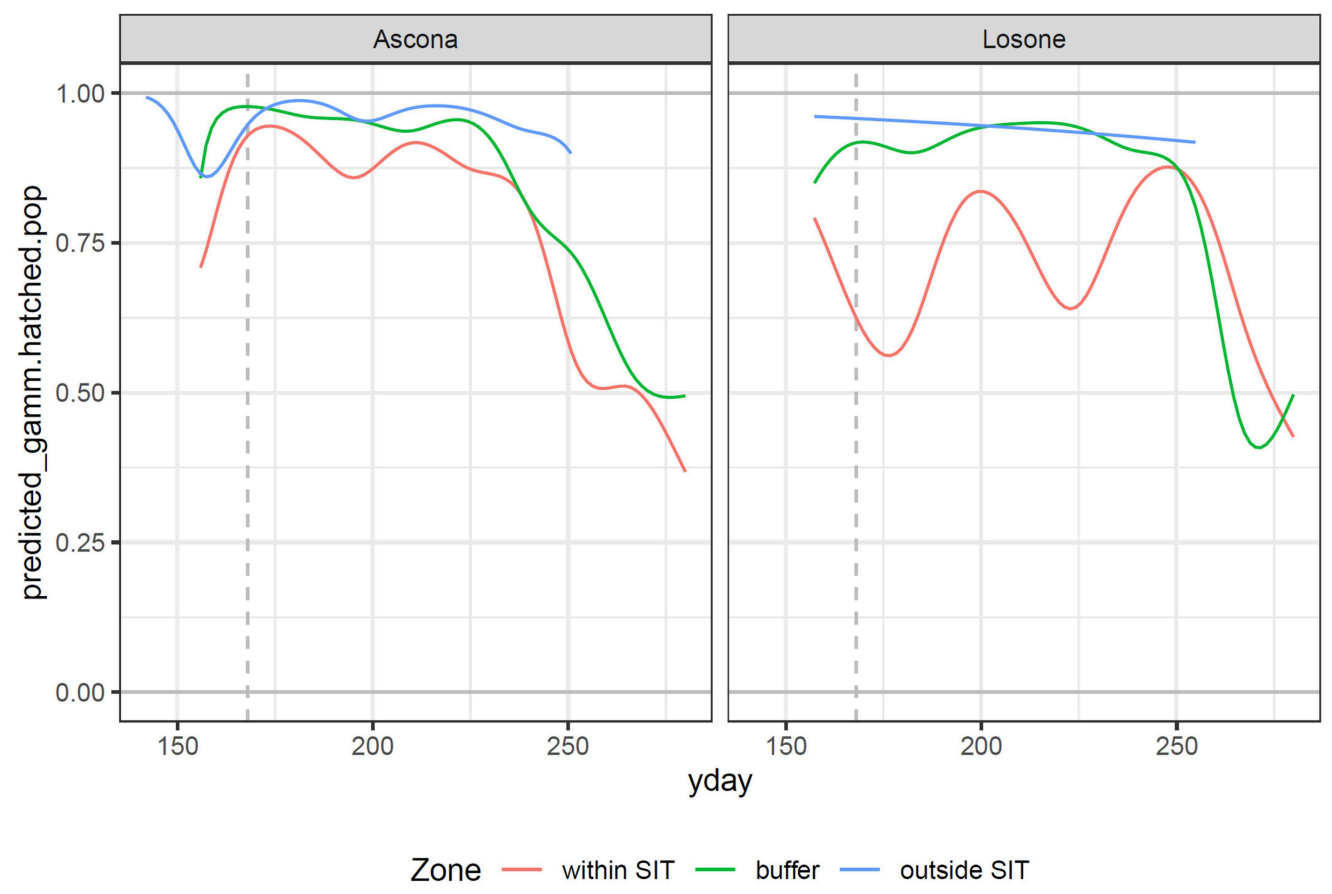
Model-predicted egg hatching rates over time in Ascona (**left**) and Losone (**right**) for the SIT-treated, non-SIT buffer, and non-SIT outside zones. Curves represent population-level predictions derived from GAMMs. The vertical dashed line indicates the start of sterile-male releases (17 June 2025). Confidence intervals are not shown to preserve readability because three predicted trajectories are displayed simultaneously in each panel. yday: Day of the year.

Model-based temporal estimates indicated that, throughout the season, predicted hatching rates remained highest in the non-SIT outside zones, intermediate in the non-SIT buffer zones, and lowest in the SIT-treated zones (Figure 3). This pattern was observed in both Ascona and Losone. Following the start of SIT releases, predicted hatching rates in the SIT-treated areas declined relative to those in the comparison zones, with divergence becoming more pronounced as the season progressed. The reduction in hatching rate was more pronounced in Losone, although estimates at this site displayed greater temporal variability.

**Figure 4:**
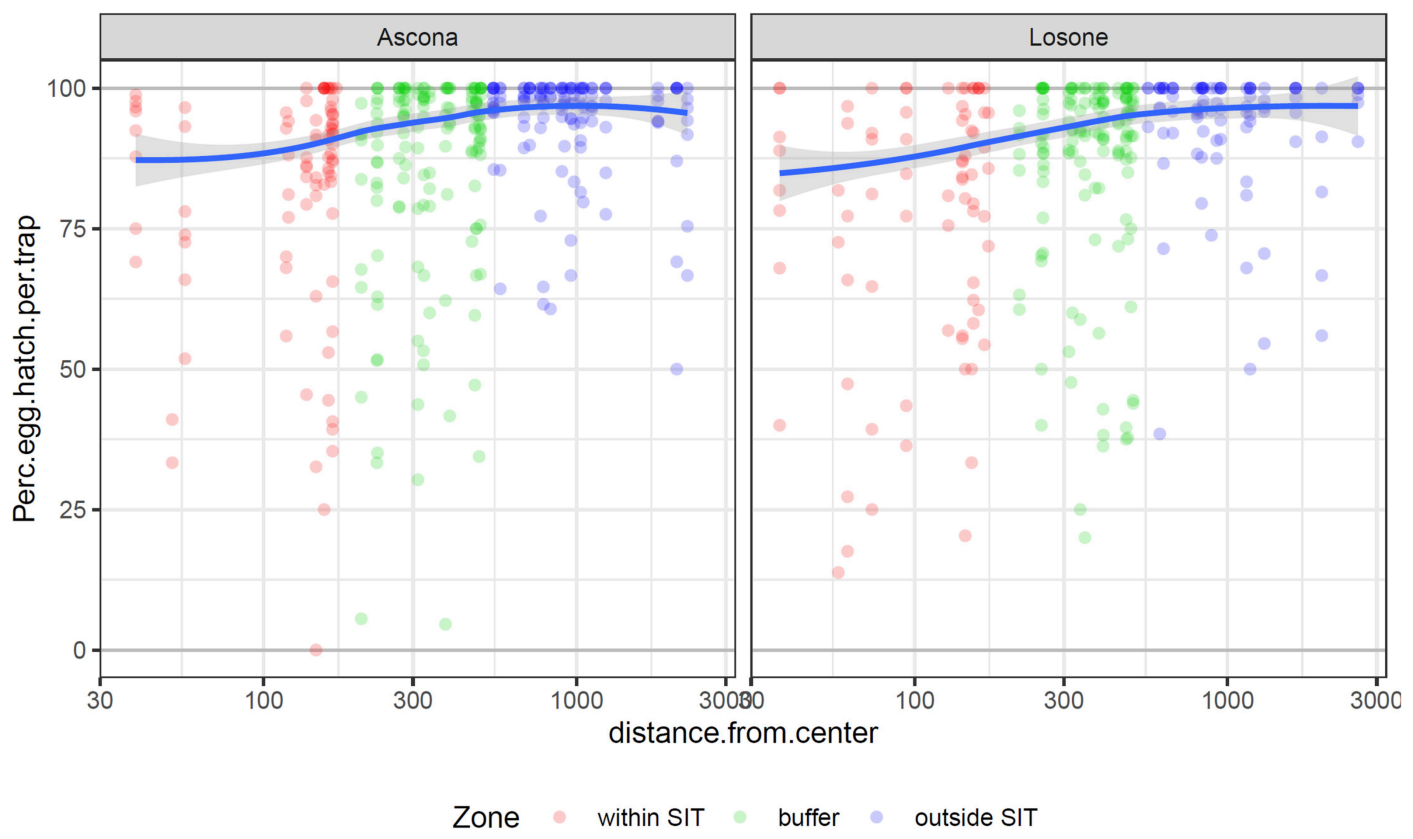
Relationship between egg hatching rate and distance from the SIT release center in Ascona (**left**) and Losone (**right**) during the 2025 SIT intervention period. Points represent individual ovitrap observations and are colored according to the analytical zone (within the SIT-treated zone, non-SIT buffer, and non-SIT outside). Solid lines show locally smoothed trends, while shaded ribbons represent the corresponding 95% confidence intervals. Distance from the release center is displayed on a logarithmic scale to facilitate visualization of observations spanning a wide range of distances.

Observed data and model-based results consistently indicated a spatially continuous response to SIT rather than an abrupt change at the boundary of the release area. When egg hatching rate was examined as a function of distance from the release center, values increased progressively with increasing distance, without any distinct threshold at 200 m corresponding to the edge of the SIT-treated zone (Figure 4). Hatching rates were lowest in the release core, increased gradually across the non-SIT buffer zone, and reached the highest levels in the outer non-SIT areas.

Across both study sites, mean hatching rates within the SIT-treated areas, when averaged over time, converged around ~80%, whereas consistently higher values were observed farther from the release center.

Quantitatively, model-based estimates indicated that mean hatching rates within the SIT-treated areas were approximately 22% lower than in the non-SIT outside zone in Losone and around 9% lower in Ascona over the intervention period, corresponding to absolute reductions of about 21 and 9 percentage points, respectively. Complementary spatial smoothers further supported this interpretation, showing a clear increase in estimated hatching rate with distance from the release center in both municipalities (see Supplementary Figures in File S2, pages 73, 75, 77, and 79).

### 3.4. Adult Female Abundance Patterns

No clear SIT-related effect on adult female abundance could be identified in either study site. Overall female counts were low and characterized by a high proportion of zero values, and the limited number of traps resulted in sparse spatial coverage, with variability among individual trap locations dominating the observed patterns. In Ascona, interpretation was further constrained by the absence of untreated observations. Under these conditions, adult female data did not allow robust inference on SIT effects and are presented for completeness only (File S3).

## 4. Discussion

This study provides a field-based evaluation of the sterile insect technique (SIT) against *Ae. albopictus* under operational conditions that deliberately deviate from the assumptions of current international guidelines [13,14]. By implementing sterile-male releases in very small (≈12 ha), non-isolated, fully urban areas—characterized by predominantly residential land use and a high prevalence of secondary residences—we explored whether SIT could generate detectable entomological responses under realistic operational conditions. Effects were evaluated using a distance-based framework rather than geographically separated control sites.

Despite the challenging context, consistent SIT-associated patterns were observed in both egg abundance and egg hatching rate. Following the onset of releases, SIT-treated areas showed lower egg abundance relative to surrounding zones, and this difference increased over the course of the season. Similar reductions in egg abundance have been reported in previous SIT trials conducted under more favorable conditions, including larger intervention areas and partially isolated settings [11,16,32]. Although the present design does not allow definitive causal attribution, the temporal and spatial consistency of the observed patterns is compatible with an effect of sterile-male releases on local mosquito reproductive dynamics.

Egg hatching rate proved to be a particularly informative indicator, consistent with previous studies showing that fertility-based metrics are among the most sensitive measures of SIT performance [11,33]. In both municipalities, hatching rates were lowest within the release areas, intermediate in adjacent buffer zones, and highest in more distant untreated areas. Moreover, hatching rates increased progressively with distance from the release center, suggesting a gradual spatial decline in SIT-associated effects rather than an abrupt boundary at the edge of the intervention area. While seasonal processes such as diapause induction may influence hatch rates in temperate populations later in the season, they are unlikely to account for the consistent spatial differences observed among zones during the intervention period.

The comparison between the two study sites is particularly informative because they were intentionally selected to represent contrasting operational contexts within the same regional mosquito control program. Losone included premises where recommended IVM measures were implemented consistently, whereas Ascona represented a more heterogeneous residential setting characterized by a high prevalence of private gardens and secondary homes. Although the overall patterns were similar across sites, Losone showed a stronger reduction in egg hatching rate than Ascona. These differences likely reflect, at least in part, the interaction between SIT and local environmental and operational conditions, highlighting the context-dependent nature of SIT performance in real urban environments.

It is also important to emphasize that SIT was evaluated as part of an existing IVM program rather than as a standalone intervention. This reflects its intended operational use, as current recommendations emphasize the integration of SIT with more conventional control measures rather than its replacement of them. Consequently, the present study should be interpreted as an assessment of SIT operating within realistic mosquito management programs rather than under experimentally simplified conditions.

The spatial gradients observed in both egg abundance and egg hatching rate are consistent with the ecology of *Ae. albopictus* in urban environments. Although individual mosquitoes can disperse over several hundred meters, most reproductive activity remains spatially structured around emergence sites [31]. Under such conditions, SIT is expected to produce gradual changes in fertility and oviposition patterns across space rather than sharp discontinuities. This may explain why the observed responses extended beyond the nominal release areas and declined progressively with distance. Importantly, the spatial gradients observed in egg hatching rate represent population-level entomological responses and should not be interpreted as direct proxies for the dispersal patterns of released sterile males, which were not measured in the present study. Instead, these gradients likely reflect the combined effects of sterile and wild mosquito movement, mating dynamics, and local environmental heterogeneity.

An important limitation of this study is the absence of a geographically independent untreated control area. Although this limits causal inference, identifying ecologically comparable and operationally independent control sites in highly connected urban systems is often challenging. For this reason, we adopted a distance-based quasi-experimental framework integrating pre-intervention baseline data and spatial gradients within and around the release areas. This approach does not replace classical experimental designs but provides a complementary perspective that may be particularly useful in open urban environments where strict spatial isolation is difficult to achieve.

Several additional sources of uncertainty should nevertheless be acknowledged. First, no direct measurements of sterile male survival, dispersal, or mating competitiveness were obtained in the present study. As a result, parameters such as sterile-to-wild male ratios remain unknown, and the observed spatial gradients cannot be interpreted as direct proxies of sterile male coverage. Second, release intensity varied over time due to logistical constraints, including delayed shipments and elevated post-transport mortality during some events. Such variability likely influenced the magnitude and temporal stability of observed effects. In addition, fluctuations in the number of viable males released over the curse of the season may have contributed to temporal variation in entomological responses. Although the present study was not designed to formally evaluate dose–response relationships between release intensity and subsequent entomological outcomes, future studies with higher temporal resolution could help clarify how variation in viable male density affects SIT performance under operational conditions.

Furthermore, environmental heterogeneity and variations in local mosquito management practices may have contributed to observed differences between locations and sampling zones. These factors should be considered when interpreting the magnitude of the reported effects. Consistent with these sources of variability, confidence intervals around some model predictions were relatively wide, particularly for specific periods of the season and for certain spatial contrasts. In addition, the spatial distribution of ovitraps was partly constrained by the configuration of the existing surveillance network and was therefore not homogeneous across all analytical zones. Given the known fine-scale heterogeneity of *Ae. albopictus* populations in urban environments, this should be considered when interpreting spatial gradients and differences among zones.

In contrast to egg-based indicators, no clear SIT-associated pattern could be identified for adult female abundance. This result should be interpreted cautiously given the low capture rates, high proportion of zero counts, and limited spatial coverage of the adult monitoring network. Under these conditions, adult female data are best regarded as descriptive and do not permit robust inference regarding potential SIT effects on adult population density.

Overall, the study demonstrates that patterns compatible with SIT activity can be detected in small, non-isolated urban areas operating under routine mosquito control programs. While the magnitude of observed effects was lower than that reported in some larger or more controlled trials, the results suggest that SIT may contribute to modifying local reproductive dynamics of *Ae. albopictus* under realistic operational conditions. Future studies should further explore how release scale, landscape connectivity, monitoring intensity, and local management practices influence SIT performance in complex urban systems.

## 5. Conclusions

This study evaluated the implementation of SIT against *Ae. albopictus* in two small, non-isolated urban areas operating under routine IVM programs. Using a spatially explicit and distance-based framework, we identified consistent spatial and temporal patterns in egg abundance and egg hatching rate that were compatible with the expected effects of sterile-male releases, whereas no clear pattern emerged for adult abundance.

The results indicate that SIT may contribute to modifying reproductive dynamics even under highly connected urban conditions and at spatial scales smaller than those typically considered in conventional SIT field trials. At the same time, the study highlights the importance of operational context, as responses varied between sites characterized by different environmental conditions and levels of implementation of complementary control measures.

Because the study was conducted in an open urban system without geographically independent control areas, the findings should be interpreted as evidence from a quasi-experimental operational evaluation rather than as definitive proof of population suppression. Nevertheless, the consistency of the observed spatial and temporal responses across two contrasting urban scenarios suggests that SIT can represent a valuable component of IVM programs under real-world conditions.

More broadly, this work contributes to the growing body of evidence needed to evaluate SIT outside idealized experimental settings and provides a framework for investigating its performance in the complex and heterogeneous environments where future operational applications are most likely to occur.

## Data Availability

The original data presented in the study are openly available in Zenodo at DOI: 10.5281/zenodo.20080629.

## Supplementary Materials

The following supporting information can be downloaded at: https://www.mdpi.com/article/10.3390/insects17080793/s1.

## Abbreviations

The following abbreviations are used in this manuscript:

FAO	Food and Agriculture Organization
GAMM	Generalized additive mixed-effects model
IAEA	International Atomic Energy Agency
IVM	Integrated vector management
NDVI	Normalized difference vegetation index
OTR-MON	Ovitraps of routine municipal surveillance network
OTR-SIT	Ovitraps deployed specifically for this SIT trial
PCA	Phased conditional approach
SIT	Sterile insect technique
WHO	World Health Organization
