# Supplementary material for: Assessing the Sterile Insect Technique in Small, Non-Isolated Urban Areas: A Field Trial Against Aedes albopictus in Southern Switzerland
Source: Insects. 2026 Jul 31;17(8):793. doi: 10.3390/insects17080793 (PMC13513938; doi:10.3390/insects17080793)
Supplement: Supplementary file 1 [file insects-17-00793-s001.zip › Figure_S1.pdf]

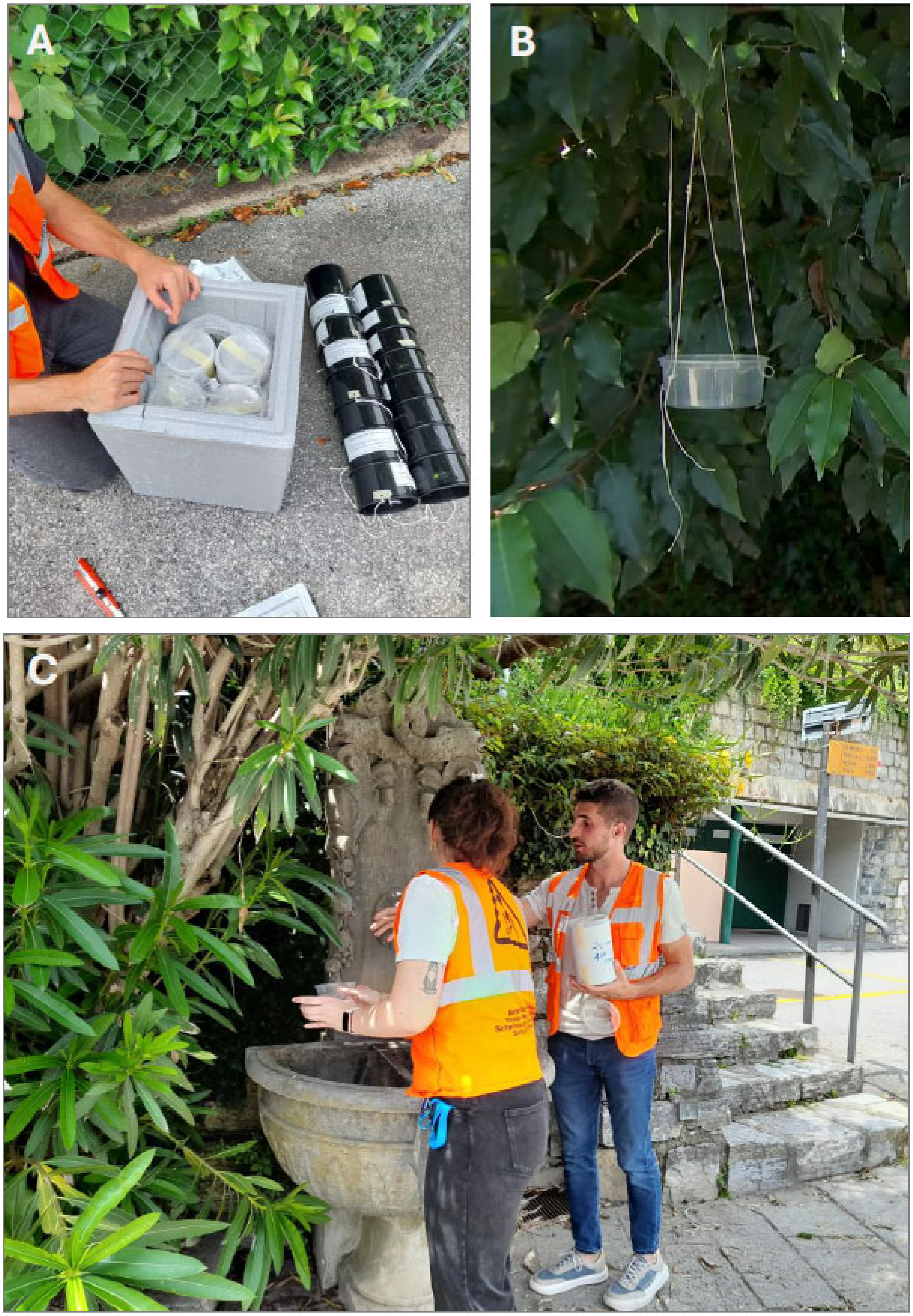

Figure S1. Representative photographs illustrating SIT implementation in Ascona and Losone during the 2025 intervention period. (A) Sterile males upon arrival from the production facility, ready for field deployment. (B) Release cups installed at a release station within the intervention area. (C) Typical urban setting showing the placement of a sterile-male release station.
