# Supplementary material for: Assessing the Sterile Insect Technique in Small, Non-Isolated Urban Areas: A Field Trial Against Aedes albopictus in Southern Switzerland
Source: Insects. 2026 Jul 31;17(8):793. doi: 10.3390/insects17080793 (PMC13513938; doi:10.3390/insects17080793)
Supplement: Supplementary file 1 [file insects-17-00793-s001.zip › File_S1.pdf]

Modelling: SIT Losone & Ascona

Egg counts

Author: Nisia Trisconi | Zurich Data Scientists
Reviewer: Dr. Matteo Tanadini | Zurich Data Scientists

December 5, 2025

Contents

1 Freeze Package versions **3**

2 Settings **3**

3 Getting data **4**

4 Aim and design **6**

5 Generalised Additive Mixed-Effects Model (GAMM) **6**
5.1 Visualising the data . . . 6
5.2 Fitting the models . . . 9
5.2.1 Preparing data set . . . 10
5.2.2 Model for Losone . . . 11
5.2.3 Model for Ascona . . . 13
5.3 Plotting the smoothers . . . 14
5.3.1 Losone . . . 14
5.3.2 Ascona . . . 18
5.4 Model selection . . . 21
5.4.1 Losone . . . 21
5.4.2 Ascona . . . 25
5.5 Fitted values . . . 28
5.6 Predicted values . . . 30
5.6.1 Plotting the differences . . . 35
5.7 Checking the offset . . . 56
5.7.1 Checking the offset for Losone . . . 56
5.7.2 Checking the offset for Ascona . . . 59
5.8 Residual analysis . . . 62

6 Spatial Generalised Additive Model (spatial GAM) **75**
6.1 Visualising the data . . . 75
6.2 Fitting the models . . . 80
6.3 Residual analysis . . . 91

7 Methods **96**
7.1 Generalised Additive Mixed-Effects Model (GAMM) . . . 96
7.2 Spatial Generalised Additive Model (spatial GAM) . . . 96

8 Results **96**

8.1 Generalised Additive Mixed-Effects Model (GAMM) 96
8.2 Spatial Generalised Additive Model (spatial GAM) 98

9 Conclusions **98**

10 References **99**

11 Appendix **100**
11.1 Number of eggs vs distance from the center of the treatment 100
11.1.1 Fitting the models 101
11.1.2 Fitted values 106
11.2 Number of eggs vs distance from center interaction with yday 107
11.2.1 Fitted values 112

12 Session Information **113**

1 Freeze Package versions

```
## (messages are omitted from this chunk)
##
library(groundhog)
pkgs <- c("dplyr",
          "ggplot2",
          "lubridate",
          "mgcViz",
          "readxl",
          "ggpubr",
          "grid",
          "stringr",
          "sf") ## automatically loads mgcv
groundhog.library(pkgs, date = "2025-07-01")
```

2 Settings

Global settings:

```
Sys.setenv(lang = "en_US")
theme_set(theme_bw())

if (!dir.exists("Prepared_data_and_models")) {
  dir.create("Prepared_data_and_models")
}
```

3 Getting data

This analysis concentrates on Losone and Ascona; as control, we use the previous year. **Moreover, we only keep functioning ovitraps**.

We load two datasets: one for temporal analysis (i.e., modelling the evolution of the number of eggs over time), and one for spatial analysis (i.e., modelling the distribution of the number of eggs across the territory).

```
## temporal analysis
d.ovitraps.temporal <- readRDS(file = paste0("Prepared_data_and_models/",
                                             "d.ovitraps.23.24.25_PreparedData.RDS")) %>%
  filter( municipality.fac %in% c("Losone", "Ascona") & status.fac == "F" ) %>%
  droplevels()

## spatial analysis
## Note that this data set is used only for visualising the data.
## For the models, d.ovitraps.temporal is used.
d.ovitraps.spatial <- readRDS(file = paste0("Prepared_data_and_models/",
                                            "d.ovitraps.spatial_PreparedData.RDS")) %>%
  filter( municipality.fac %in% c("Losone", "Ascona") ) %>%
  droplevels()
## Altered traps have already been filtered out when creating this data set
```

Let's check which municipalities we have.

```
d.ovitraps.temporal %>%
  pull(municipality.fac) %>%
  unique()
```

```
[1] Ascona Losone
Levels: Ascona Losone
```

```
## makes sense
##
d.ovitraps.spatial %>%
  pull(municipality.fac) %>%
  unique()
```

```
[1] Ascona Losone
Levels: Ascona Losone
```

```
## makes sense
```

Overview of the data:

```
dim(d.ovitraps.temporal)
```

```
[1] 1180   40
```

```
head(d.ovitraps.temporal)[1:min(ncol(d.ovitraps.temporal), 30)]
```

```
# A tibble: 6 x 30
  OriginalSheet Activation.time total.eggs total.albopictus.egg
  <chr>                   <dbl>      <dbl>                <dbl>
1 1                          14          0                    0
2 1                          14          0                    0
3 1                          14          0                    0
4 1                          14          0                    0
5 1                          14          0                    0
```

```
6 1                           14          0                   0
# i 26 more variables: WHOLE.albopictus.egg <dbl>,
#   DESSICATED.albopictus.egg <dbl>, HATCH.albopictus.egg <dbl>,
#   WHOLE.albopictus.egg..AFTER.HATCHING.PROCEDURE. <dbl>,
#   DESSICATED.albopictus.egg..AFTER.HATCHING.PROCEDURE. <dbl>,
#   HATCH.albopictus.egg..AFTER.HATCHING.PROCEDURE. <dbl>,
#   Perc.egg.hatch.per.trap <dbl>, Year <dbl>, unique.ID <fct>, X.num <dbl>,
#   Y.num <dbl>, round.fac <fct>, Setting_date.date <date>, ...
```

```r
str(d.ovitraps.temporal)
```

```
tibble [1,180 x 40] (S3: tbl_df/tbl/data.frame)
 $ OriginalSheet                                      : chr [1:1180] "1" "1" "1" "1" ...
 $ Activation.time                                    : num [1:1180] 14 14 14 14 14 14 14 14 14 14 ...
 $ total.eggs                                         : num [1:1180] 0 0 0 0 0 0 0 0 0 0 ...
 $ total.albopictus.egg                               : num [1:1180] 0 0 0 0 0 0 0 0 0 0 ...
 $ WHOLE.albopictus.egg                               : num [1:1180] 0 0 0 0 0 0 0 0 0 0 ...
 $ DESSICATED.albopictus.egg                          : num [1:1180] 0 0 0 0 0 0 0 0 0 0 ...
 $ HATCH.albopictus.egg                               : num [1:1180] 0 0 0 0 0 0 0 0 0 0 ...
 $ WHOLE.albopictus.egg..AFTER.HATCHING.PROCEDURE.     : num [1:1180] NA NA NA NA NA NA NA NA NA NA ...
 $ DESSICATED.albopictus.egg..AFTER.HATCHING.PROCEDURE.: num [1:1180] NA NA NA NA NA NA NA NA NA NA ...
 $ HATCH.albopictus.egg..AFTER.HATCHING.PROCEDURE.     : num [1:1180] NA NA NA NA NA NA NA NA NA NA ...
 $ Perc.egg.hatch.per.trap                            : num [1:1180] NA NA NA NA NA NA NA NA NA NA ...
 $ Year                                               : num [1:1180] 2024 2024 2024 2024 2024 ...
 $ unique.ID                                          : Factor w/ 100 levels "Ascona.1","Ascona.10",..
 $ X.num                                              : num [1:1180] 703175 703120 703644 702922 70336
 $ Y.num                                              : num [1:1180] 113224 112967 112960 112519 11251
 $ round.fac                                          : Factor w/ 20 levels "1","2","3","4",..: 12 12 
 $ Setting_date.date                                  : Date[1:1180], format: "2024-05-08" "2024-05-08
 $ Sampling_date.date                                 : Date[1:1180], format: "2024-05-22" "2024-05-22
 $ municipality.ord                                   : Ord.factor w/ 2 levels "Ascona"<"Losone": 1 1 
 $ municipality.fac                                   : Factor w/ 2 levels "Ascona","Losone": 1 1 1 1 
 $ status.fac                                         : Factor w/ 1 level "F": 1 1 1 1 1 1 1 1 1 1 ...
 $ perc.manual                                        : num [1:1180] NA NA NA NA NA NA NA NA NA NA ...
 $ Year.fac                                           : Factor w/ 2 levels "2024","2025": 1 1 1 1 1 1 
 $ hatched.albo.eggs.after.proc                       : num [1:1180] NA NA NA NA NA NA NA NA NA NA ...
 $ daily.albopictus.eggs                              : num [1:1180] 0 0 0 0 0 0 0 0 0 0 ...
 $ yday                                               : num [1:1180] 143 143 143 143 143 143 143 143 14
 $ non.hatched.albo.eggs.after.proc                   : num [1:1180] NA NA NA NA NA NA NA NA NA NA ...
 $ week                                               : num [1:1180] 21 21 21 21 21 21 21 21 21 21 ...
 $ unique.ID.Year                                     : Factor w/ 145 levels "Ascona.10b.2024",..: 21 
 $ super.municipality.year                            : Factor w/ 4 levels "Ascona:2024",..: 1 1 1 1 1
 $ municipality.fac.spatial                           : Factor w/ 2 levels "Ascona","Losone": 1 1 1 1 
 $ zona.SIT.fac                                       : Factor w/ 4 levels "dentro SIT","buffer",..: N
 $ zona.SIT.discrete.fac                              : Factor w/ 3 levels "within SIT","buffer",..: 3
 $ Year.ord                                           : Ord.factor w/ 2 levels "2024"<"2025": 1 1 1 1 
 $ distance.from.center                               : num [1:1180] NA NA NA NA NA NA NA NA NA NA ...
 $ SIT.fac                                            : Factor w/ 2 levels "Pre SIT","SIT": 1 1 1 1 1 
 $ zona.SIT.discrete.ord                              : Ord.factor w/ 3 levels "within SIT"<"buffer"<.
 $ super.zona.SIT.discrete.Year                       : Factor w/ 4 levels "outside SIT.2024",..: 1 1 
 $ super.zona.SIT.discrete.Year.ord                   : Ord.factor w/ 4 levels "outside SIT.2024"<..: 
 $ super.municipality.year.ord                        : Ord.factor w/ 4 levels "Ascona:2024"<..: 1 1 1
```

```r
##
dim(d.ovitraps.spatial)
```

```
[1] 145   9
```

```r
head(d.ovitraps.spatial)[1:min(ncol(d.ovitraps.spatial), 30)]
```

```
# A tibble: 6 x 9
  unique.ID  Year.fac mean.eggs median.eggs  X.num  Y.num municipality.fac.spa~1
  <fct>      <fct>        <dbl>       <dbl>  <dbl>  <dbl> <fct>
1 Ascona.1   2025         189.          215  7.03e5 1.12e5 Ascona
2 Ascona.10  2025         279.          260  7.03e5 1.12e5 Ascona
3 Ascona.10b 2024          93.7         106. 7.03e5 1.12e5 Ascona
4 Ascona.10b 2025          70.4          60  7.03e5 1.12e5 Ascona
5 Ascona.11  2025         128.           99  7.03e5 1.12e5 Ascona
6 Ascona.11a 2024          99.8         118. 7.04e5 1.12e5 Ascona
# i abbreviated name: 1: municipality.fac.spatial
# i 2 more variables: municipality.fac <fct>, zona.SIT.fac <fct>
```

```r
str(d.ovitraps.spatial)
```

```
tibble [145 x 9] (S3: tbl_df/tbl/data.frame)
 $ unique.ID               : Factor w/ 100 levels "Ascona.1","Ascona.10",..: 1 2 3 3 4 5 5 6 7 7 ...
 $ Year.fac                : Factor w/ 2 levels "2024","2025": 2 2 1 2 2 1 2 2 1 2 ...
 $ mean.eggs               : num [1:145] 188.6 278.6 93.7 70.4 128.1 ...
 $ median.eggs             : num [1:145] 215 260 106 60 99 ...
 $ X.num                   : num [1:145] 703393 703328 703474 703474 703434 ...
 $ Y.num                   : num [1:145] 112214 111998 112351 112351 111909 ...
 $ municipality.fac.spatial: Factor w/ 2 levels "Ascona","Losone": 1 1 1 1 1 1 1 1 1 1 ...
 $ municipality.fac        : Factor w/ 2 levels "Ascona","Losone": 1 1 1 1 1 1 1 1 1 1 ...
 $ zona.SIT.fac            : Factor w/ 4 levels "dentro SIT","buffer",..: 1 1 NA 4 1 NA NA 1 NA NA ...
```

4 Aim and design

The aim of this analysis is to determine whether the release of sterilised males in a given area helps reducing the number of laid eggs. With this purpose in mind, we are going to compare the treated, buffer and untreated zones of Losone and Ascona to check whether we see any difference.

In total, the data was collected on `40` different dates; Losone was sampled `26` times, whereas Ascona was sampled `27` times.

5 Generalised Additive Mixed-Effects Model (GAMM)

5.1 Visualising the data

We begin by displaying the number of eggs found in the traps sampled throughout the season for the year 2025. As we are dealing with count data, we apply a square-root transformation to the response variable *total.albopictus.egg* to stabilise variance. We group the observations based on the treatment zone (*zona.SIT.discrete.fac*).

Furthermore, observations from the same ovitrap are connected with a line to visualise trends over time. On top of these lines, we add a smoothed average grouped by the treatment zone, which allows us to check whether a treatment effect is present.

```r
d.ovitraps.temporal %>%
  filter(Year.fac == 2025) %>%
  ggplot(mapping = aes(x = Sampling_date.date,
                       y = total.albopictus.egg,
                       colour = zona.SIT.discrete.fac)) +
```

```r
  geom_vline(xintercept = ymd("2025-06-17"), linetype = "dashed", colour = "grey") +
  geom_hline(yintercept = 0, colour = "grey") +
  geom_point(alpha = 0.2) +
  geom_line(mapping = aes(group = unique.ID), alpha = 0.2) +
  facet_wrap(~ municipality.fac, scales = "free_x") +
  geom_smooth(alpha = 0.3) +
  scale_colour_manual(values = c("blue", "green3", "red")) +
  coord_cartesian(ylim = c(0, NA)) +
  labs(colour = "Zone") +
  theme(legend.position = "bottom")
```

```
`geom_smooth()` using method = 'loess' and formula = 'y ~ x'
```

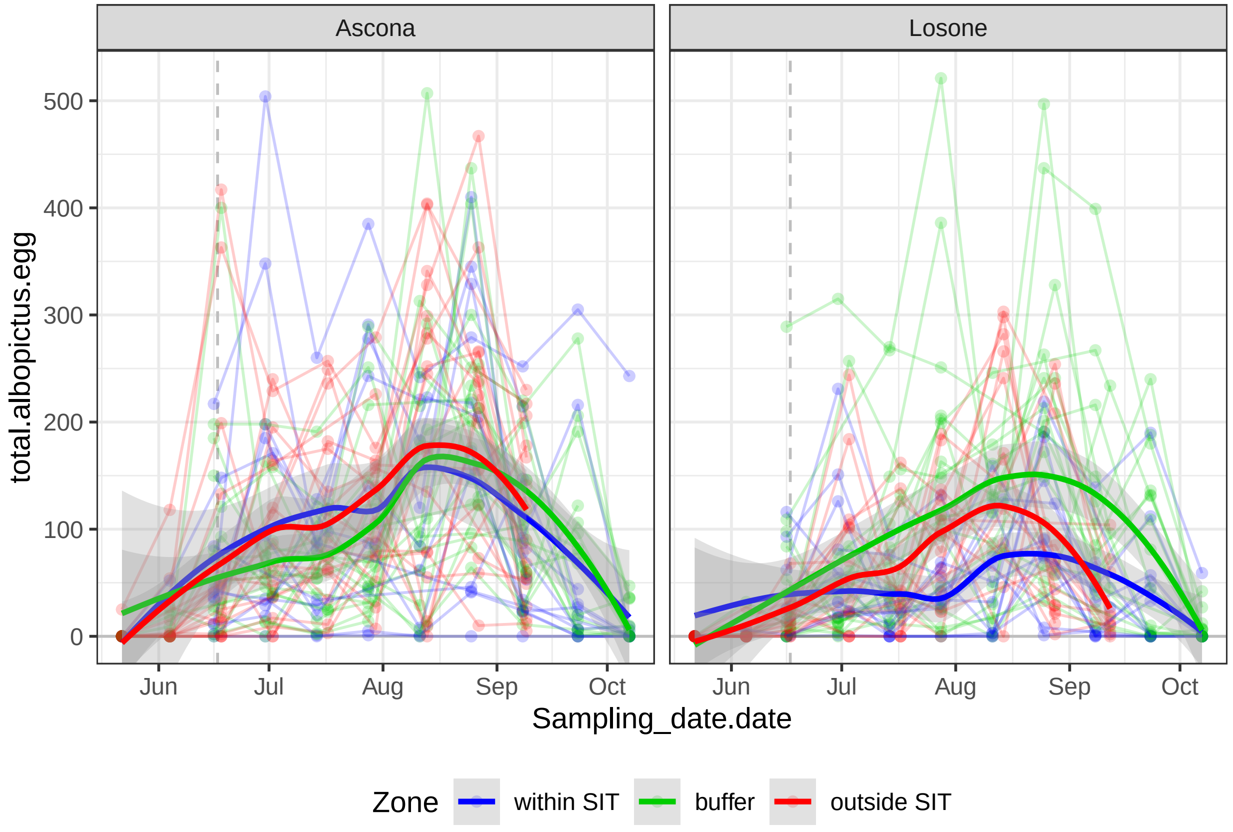

We observe that for Losone there is a difference that increases over time between the treated zone and the other two zones, while for Ascona the difference is not clear.

In particular, for Losone we see a change in the pattern of egg counts after the treatment started (vertical dashed line).

We now compare egg counts over the two measured years 2024 and 2025, differentiating between the treatment zones. The year 2024 was not treated, therefore we consider the entire area of the year 2024 as "outside SIT".

```r
## check year 2024
d.ovitraps.temporal %>%
  filter(Year.fac == 2024) %>%
  pull(zona.SIT.discrete.fac) %>%
  unique()
```

```
[1] outside SIT
```

```
Levels: within SIT buffer outside SIT
```

```r
## makes sense
```

```r
d.ovitraps.temporal %>%
  ggplot(mapping = aes(yday,
                       y = total.albopictus.egg)) +
  geom_hline(yintercept = 0, colour = "grey") +
  geom_vline(xintercept = yday("2025-06-17"), linetype = "dashed", colour = "grey") +
  geom_point(alpha = 0.3) +
  geom_line(mapping = aes(group = interaction(Year.fac, unique.ID)), alpha = 0.1) +
  geom_smooth(mapping = aes(colour = interaction(zona.SIT.discrete.fac, Year.fac)),
              se = FALSE) +
  facet_grid(~ municipality.fac) +
  coord_cartesian(ylim = c(0, NA)) +
  scale_colour_manual(values = c("gold", "blue", "green3", "red")) +
  labs(colour = "Zone") +
  theme(legend.position = "bottom")
```

```
`geom_smooth()` using method = 'loess' and formula = 'y ~ x'
```

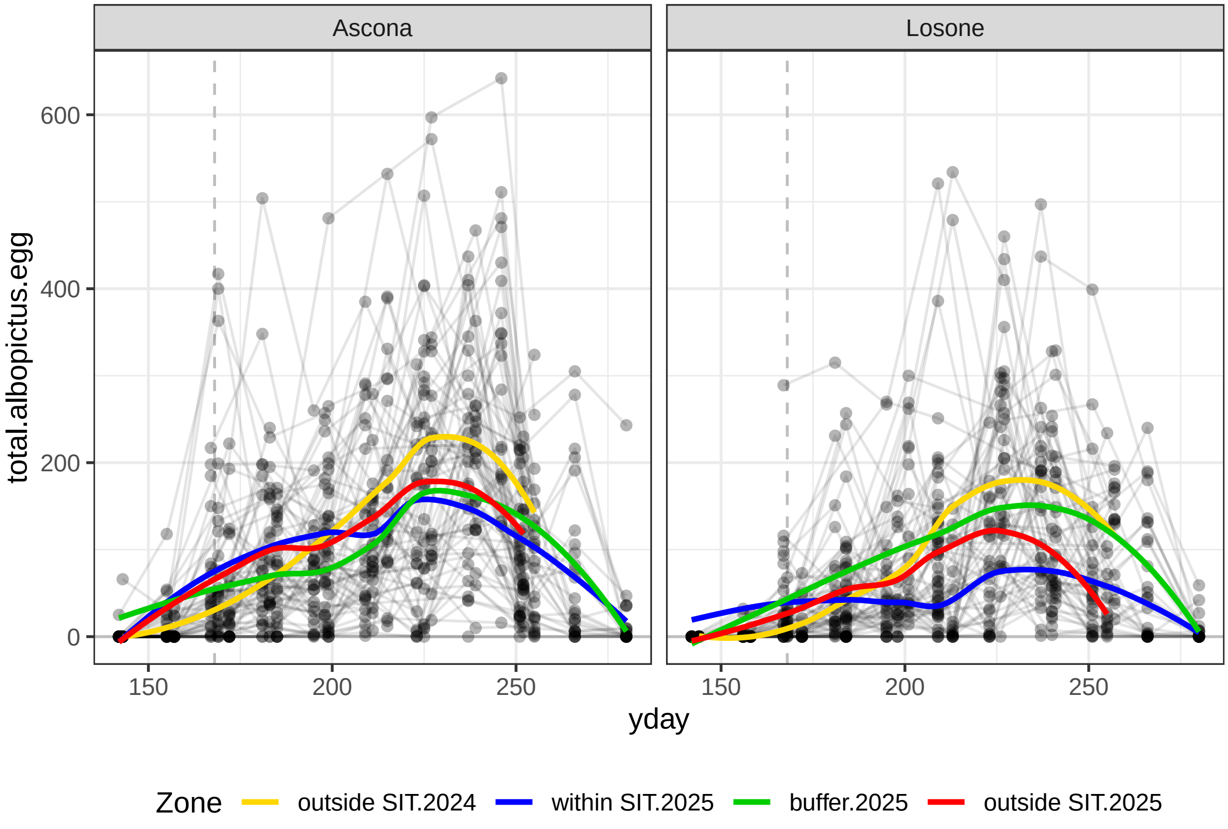

We observe that egg counts in the year 2024 start lower than 2025, but then increase and surpass the counts of 2025.

Another interesting aspect to explore is the relationship between the number of eggs laid and the distance from the release center. The idea behind this is to understand how large the treated area needs to be in order to observe an effect and, if an effect is detected, how far beyond the treated area it propagates.

We create a scatter plot of *total.albopictus.egg* against *distance.from.center*, grouping the points by treatment zone. A smoothed average is added to visualise potential trends.

```r
d.ovitraps.temporal %>%
  filter(Year.fac == 2025) %>%
  ggplot(mapping = aes(x = distance.from.center,
                       y = total.albopictus.egg,
  )) +
  geom_hline(yintercept = 0, colour = "grey") +
  geom_point(alpha = 0.2, mapping = aes(colour = zona.SIT.discrete.fac)) +
  facet_wrap(~ municipality.fac, scales = "free_x") +
  geom_smooth(alpha = 0.3) +
  scale_colour_manual(values = c("blue", "green3", "red")) +
  coord_cartesian(ylim = c(0, NA)) +
  labs(colour = "Zone") +
  theme(legend.position = "bottom")
```

```
`geom_smooth()` using method = 'loess' and formula = 'y ~ x'
```

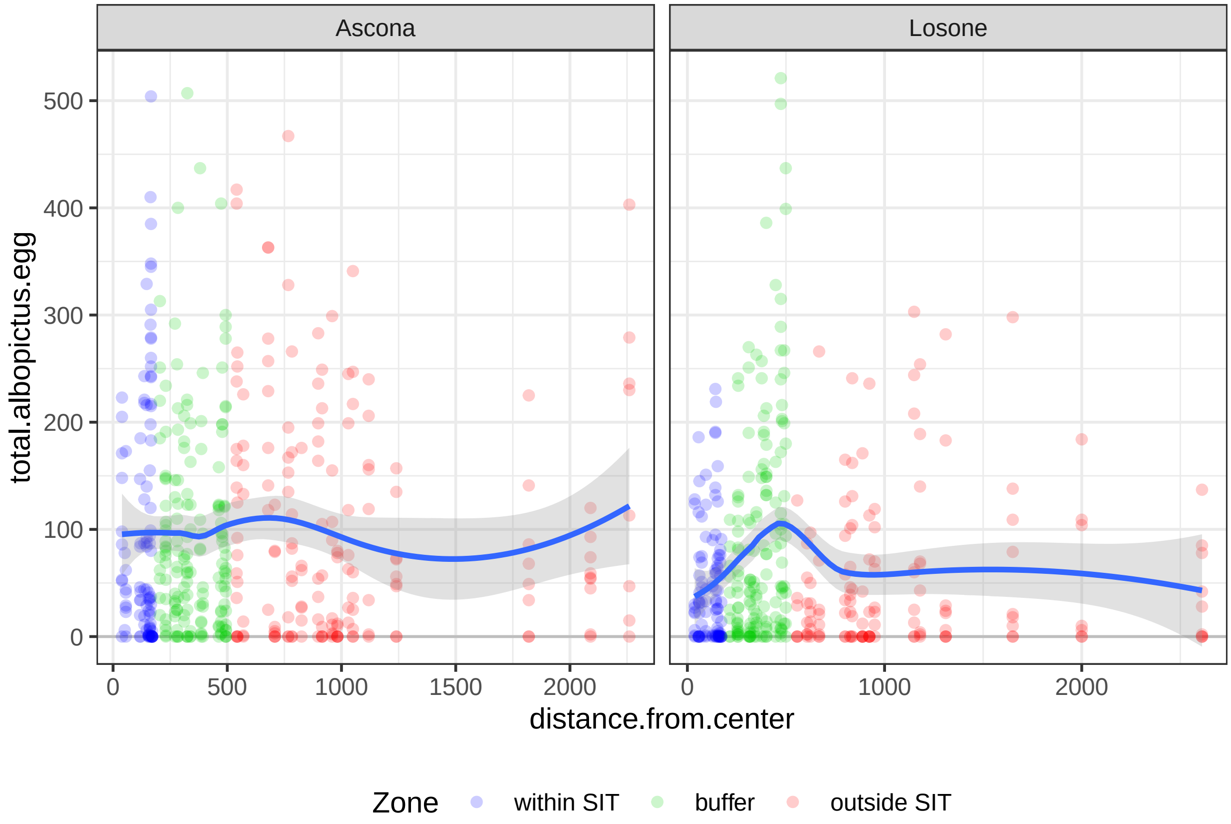

We see an initial positive linear relationship between the two variables up to the limit zone of the buffer area (i.e., 500m) for Losone. Outside of the buffer area, the number of eggs seems to decrease again and then stabilise. However, we know that the outside zone of Losone has different characteristics compared to the other two zones, which could explain this pattern.

In Ascona, no clear relationship is observed.

5.2 Fitting the models

We aim to model the total number of Aedes albopictus eggs (*total.albopictus.eggs*) laid throughout the season, represented by the day of the year (*yday*). Separate models are fitted for the two municipalities, as their

seasonal patterns differ. This approach avoids the need for an overly complex model that would allow different trend shapes depending on the municipality.

Because *total.albopictus.eggs* represents count data, we employ a negative binomial model to account for overdispersion, which is common in such data.

To account for interannual variability, *Year.fac* is included as an additive fixed effect. We do not model it as an interaction term, assuming that the yearly effect influences the overall level of egg counts additively, but does not alter the seasonal pattern. This makes sense from a biological point of view and contributes to a more straightforward model interpretation. Moreover, visual inspection doesn't reveal significant differences in seasonal patterns between the two years.

As already mentioned, our goal is to assess whether egg counts differ among treatment zones. The treatment zone may not only influence the magnitude of egg counts but also modify the shape of their seasonal trend (as observed graphically). To capture this, we treat *yday* as a numeric covariate whose effect is allowed to vary across treatment zones (*zona.SIT.discrete.fac*).

We use a Generalised Additive Model (GAM) to flexibly model seasonal dynamics without imposing a predefined functional form. This approach enhances model flexibility and captures complex temporal patterns, albeit at the cost of some interpretability.

To control for varying exposure times between ovitraps, we include *Activation.time* as an offset in the model. By log-transforming *Activation.time*, we assume that doubling the activation time leads to a proportional doubling in the number of eggs (in fact, having a log-log model corresponds to estimating the percentage increase in the response variable with a 1% increase in the explanatory variable, keeping all the rest constant).

Let's check which values are available for *Activation.time*.

```
d.ovitraps.temporal %>%
  group_by(municipality.fac, Year.fac) %>%
  reframe(range.activation.time.low = range(Activation.time, na.rm = TRUE)[1],
          range.activation.time.up = range(Activation.time, na.rm = TRUE)[2]) %>%
  ungroup()
```

```
# A tibble: 4 x 4
  municipality.fac Year.fac range.activation.time.low range.activation.time.up
  <fct>            <fct>                        <dbl>                    <dbl>
1 Ascona           2024                             9                       19
2 Ascona           2025                            13                       15
3 Losone           2024                            12                       17
4 Losone           2025                            11                       17
```

There are quite some different possible values.

Finally, to account for the lack of independence among observations from the same ovitrap, we include *unique.ID* (the unique ovitrap identifier) as a random effect. This adjustment helps to capture the variability specific to each ovitrap.

5.2.1 Preparing data set

We check if there are any missing values for the relevant variables.

```
d.ovitraps.temporal %>%
  select(total.albopictus.egg,
         municipality.fac,
         Activation.time,
```

```
         unique.ID,
         Sampling_date.date,
         yday,

         zona.SIT.discrete.fac,
         zona.SIT.discrete.ord,

         Year.fac,
         Year.ord) %>%
  na.omit() %>%
  droplevels() %>%
  nrow()
```

```
[1] 1180
```

```
nrow(d.ovitraps.temporal)
```

```
[1] 1180
```

```
## no missing values
```

No missing values are present for the relevant variables.

5.2.2 Model for Losone

We start by modelling Losone.

```
d.ovitraps.temporal.Losone <- d.ovitraps.temporal %>%
  filter(municipality.fac == "Losone")
```

We use the `gamV()` function instead of `gam()`, because it fits the `gam()` model and automatically converts it to a `gamViz` object, making visualisation easier.
The model's estimates are exactly the same.

```
gamm.eggs.Losone <- gamV(total.albopictus.egg ~

                           offset(log(Activation.time)) +
                           s(unique.ID, bs = "re") +

                           ## Fixed effects
                           zona.SIT.discrete.fac +
                           Year.fac +

                           ## Interaction:
                           ## Treatment effect over seasonality
                           s(yday, by = zona.SIT.discrete.fac),

                         family = "nb",
                         method = "GCV.Cp", ## same as gam
                         data = d.ovitraps.temporal.Losone)
summary(gamm.eggs.Losone)
```

```
Family: Negative Binomial(0.574)
Link function: log
```

```
Formula:
total.albopictus.egg ~ offset(log(Activation.time)) + s(unique.ID,
    bs = "re") + zona.SIT.discrete.fac + Year.fac + s(yday, by = zona.SIT.discrete.fac)

Parametric coefficients:
                                       Estimate     Std. Error z value Pr(>|z|)
(Intercept)                        0.528566468021 0.273993240938 1.92912 0.053716
zona.SIT.discrete.facbuffer        0.619452938968 0.262686701018 2.35814 0.018367
zona.SIT.discrete.facoutside SIT 0.305342559936 0.295188117884 1.03440 0.300949
Year.fac2025                       0.124119310306 0.173174029170 0.71673 0.473540

(Intercept)                        .
zona.SIT.discrete.facbuffer        *
zona.SIT.discrete.facoutside SIT
Year.fac2025
---
Signif. codes:  0 '***' 0.001 '**' 0.01 '*' 0.05 '.' 0.1 ' ' 1

Approximate significance of smooth terms:
                                                  edf         Ref.df
s(unique.ID)                              26.02601077679 47.00000000000
s(yday):zona.SIT.discrete.facwithin SIT   4.76592567627  5.84609022032
s(yday):zona.SIT.discrete.facbuffer       4.97904984928  6.07331800085
s(yday):zona.SIT.discrete.facoutside SIT  5.81648313997  6.80658411432
                                            Chi.sq              p-value
s(unique.ID)                               59.82815           0.0000021552 ***
s(yday):zona.SIT.discrete.facwithin SIT   32.00270           0.0000188539 ***
s(yday):zona.SIT.discrete.facbuffer       74.69581 < 0.000000000000000222 ***
s(yday):zona.SIT.discrete.facoutside SIT 321.41109 < 0.000000000000000222 ***
---
Signif. codes:  0 '***' 0.001 '**' 0.01 '*' 0.05 '.' 0.1 ' ' 1

R-sq.(adj) =  0.416   Deviance explained = 46.8%
-REML = 2541.2  Scale est. = 1         n = 552
```

The offset is included in the formula interface to ensure it is properly handled when making predictions. The reason for this is that offsets provided outside the formula are ignored during predictions, unlike those specified within the formula. Hence, we provide the offset through the formula to ensure it is included in the model fitting and predictions.

We extract the standard deviation associated with the random effect.

```r
gam.vcomp(gamm.eggs.Losone) %>%
  head(n = 1)
```

```
Standard deviations and 0.95 confidence intervals:

                                               std.dev                lower
s(unique.ID)                              0.4893876480517871 0.32250706112004740
s(yday):zona.SIT.discrete.facwithin SIT   0.0162675176277378 0.00474330778347075
s(yday):zona.SIT.discrete.facbuffer       0.0140630806649276 0.00492592412435389
s(yday):zona.SIT.discrete.facoutside SIT 0.0237763656131787 0.01053853192471209
                                                 upper
s(unique.ID)                              0.7426202366977329
```

```
s(yday):zona.SIT.discrete.facwithin SIT  0.0557906300516566
s(yday):zona.SIT.discrete.facbuffer      0.0401488599490357
s(yday):zona.SIT.discrete.facoutside SIT 0.0536427242247966

Rank: 4/4
                       std.dev             lower             upper
s(unique.ID) 0.489387648051787 0.322507061120047 0.742620236697733
```

5.2.3 Model for Ascona

We now model Ascona.

```r
d.ovitraps.temporal.Ascona <- d.ovitraps.temporal %>%
  filter(municipality.fac == "Ascona")
```

```r
## with distance from center as discrete variable
gamm.eggs.Ascona <- gamV(total.albopictus.egg ~

                           offset(log(Activation.time)) +
                           s(unique.ID, bs = "re") +

                           ## Fixed effects
                           zona.SIT.discrete.fac +
                           Year.fac +

                           ## Interaction:
                           ## Treatment effect over seasonality
                           s(yday, by = zona.SIT.discrete.fac),

                         family = "nb",
                         method = "GCV.Cp", ## same as gam
                         data = d.ovitraps.temporal.Ascona)
##
summary(gamm.eggs.Ascona)
```

```
Family: Negative Binomial(0.809)
Link function: log

Formula:
total.albopictus.egg ~ offset(log(Activation.time)) + s(unique.ID,
    bs = "re") + zona.SIT.discrete.fac + Year.fac + s(yday, by = zona.SIT.discrete.fac)

Parametric coefficients:
                                        Estimate      Std. Error z value
(Intercept)                      1.0339035829552 0.2970771661256 3.48025
zona.SIT.discrete.facbuffer      0.1386526480395 0.3209619135269 0.43199
zona.SIT.discrete.facoutside SIT 0.0916481430581 0.3179948143611 0.28821
Year.fac2025                     0.2101298564331 0.1303001652755 1.61266
                                   Pr(>|z|)
(Intercept)                      0.00050094 ***
zona.SIT.discrete.facbuffer      0.66574798
zona.SIT.discrete.facoutside SIT 0.77318874
Year.fac2025                     0.10681841
```

```
---
Signif. codes:  0 '***' 0.001 '**' 0.01 '*' 0.05 '.' 0.1 ' ' 1

Approximate significance of smooth terms:
                                                   edf          Ref.df
s(unique.ID)                               43.00630754114 51.00000000000
s(yday):zona.SIT.discrete.facwithin SIT     4.17558258080  5.16801975171
s(yday):zona.SIT.discrete.facbuffer         7.00409764171  8.03333693282
s(yday):zona.SIT.discrete.facoutside SIT    5.59922009349  6.64334882551
                                              Chi.sq                p-value
s(unique.ID)                               340.72148 < 0.0000000000000000222 ***
s(yday):zona.SIT.discrete.facwithin SIT     47.70736 < 0.0000000000000000222 ***
s(yday):zona.SIT.discrete.facbuffer        106.22881 < 0.0000000000000000222 ***
s(yday):zona.SIT.discrete.facoutside SIT   636.78806 < 0.0000000000000000222 ***
---
Signif. codes:  0 '***' 0.001 '**' 0.01 '*' 0.05 '.' 0.1 ' ' 1

R-sq.(adj) =  -3.42   Deviance explained =   53%
-REML = 3184.2  Scale est. = 1         n = 628
```

We extract the standard deviation associated with the random effect.

```r
gam.vcomp(gamm.eggs.Ascona) %>%
  head(n = 1)
```

```
Standard deviations and 0.95 confidence intervals:

                                                   std.dev                 lower
s(unique.ID)                               0.9068198440440697 0.70560363741196819
s(yday):zona.SIT.discrete.facwithin SIT    0.0104319224403510 0.00377528413440824
s(yday):zona.SIT.discrete.facbuffer        0.0294850678925455 0.01432933568651713
s(yday):zona.SIT.discrete.facoutside SIT   0.0144564110233016 0.00746334092725386
                                                     upper
s(unique.ID)                               1.1654166531343946
s(yday):zona.SIT.discrete.facwithin SIT    0.0288256464750982
s(yday):zona.SIT.discrete.facbuffer        0.0606705884799690
s(yday):zona.SIT.discrete.facoutside SIT   0.0280019125096477

Rank: 4/4

                       std.dev             lower            upper
s(unique.ID) 0.90681984404407 0.705603637411968 1.16541665313439
```

5.3 Plotting the smoothers

We now plot the fitted smoothers.

5.3.1 Losone

Let's start with Losone.

```r
plot.gam(gamm.eggs.Losone,
         pages = 1,
```

```
          trans = exp,
          ylim = c(0, 6))
```

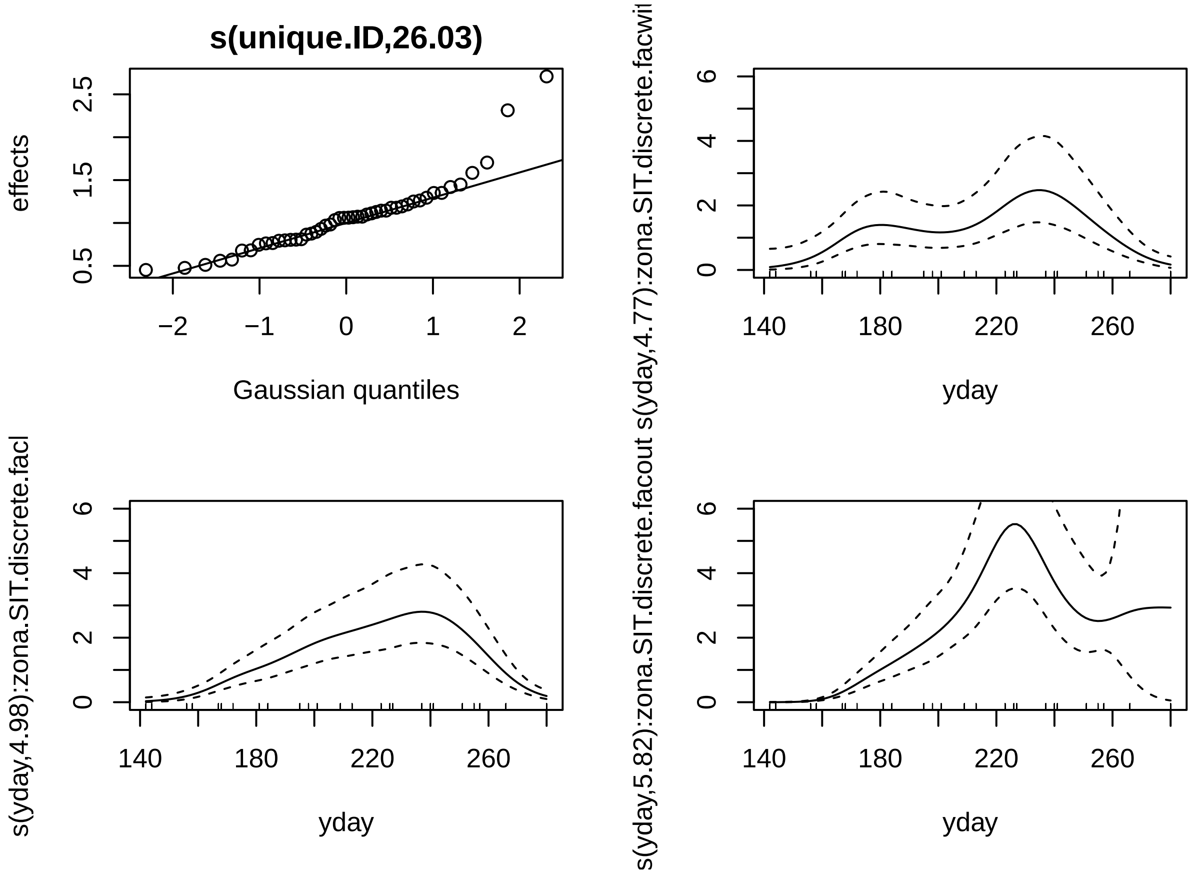

We add the corresponding shift.

```
## Within SIT, 2025
plot.gam(gamm.eggs.Losone,
          select = 2,
          shift = gamm.eggs.Losone$coefficients["(Intercept)"] +
            gamm.eggs.Losone$coefficients["Year.fac2025"],
          trans = exp,
          ylim = c(0, 15))
```

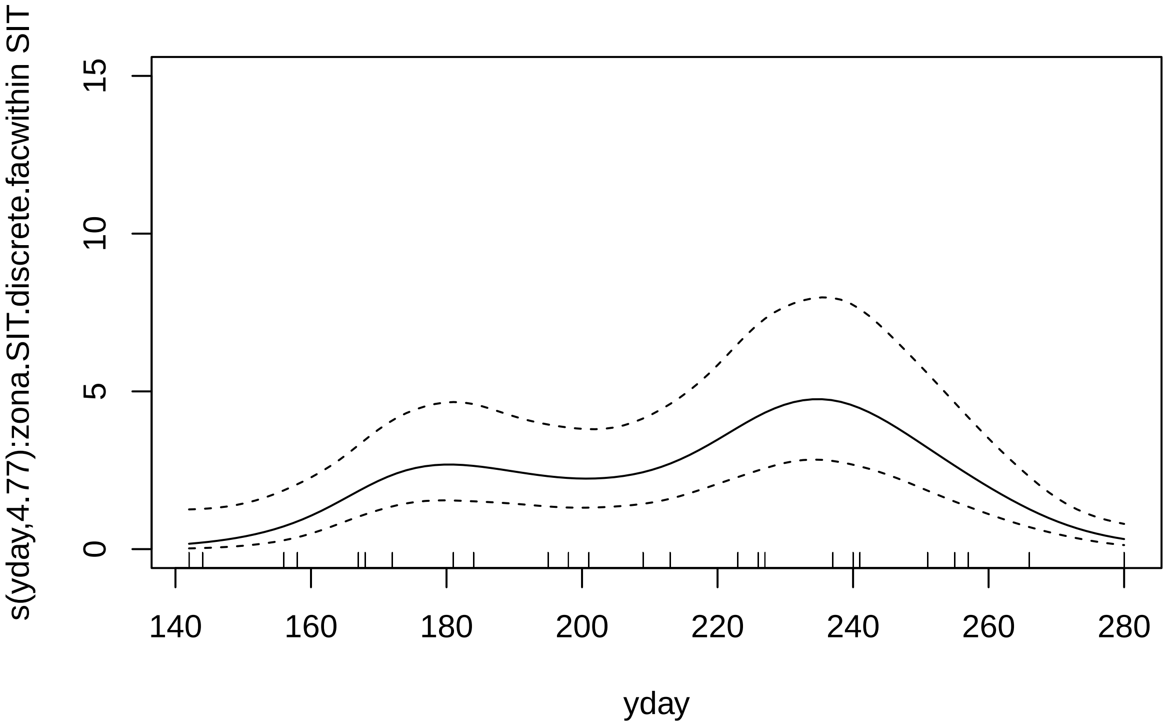


```r
##
## buffer, 2025
plot.gam(gamm.eggs.Losone,
         select = 3,
         shift = gamm.eggs.Losone$coefficients["(Intercept)"] +
           gamm.eggs.Losone$coefficients["zona.SIT.discrete.facbuffer"] +
           gamm.eggs.Losone$coefficients["Year.fac2025"],
         trans = exp,
         ylim = c(0, 15))
```

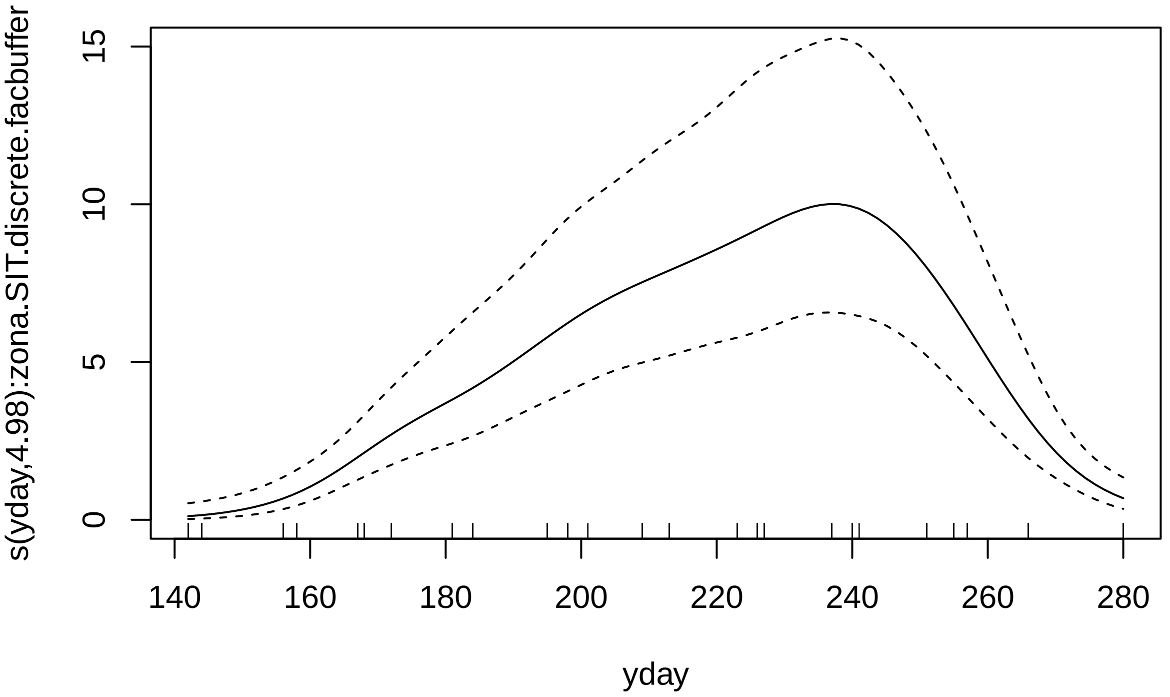


```r
##
## outside SIT, 2025
plot.gam(gamm.eggs.Losone,
         select = 4,
         shift = gamm.eggs.Losone$coefficients["(Intercept)"] +
           gamm.eggs.Losone$coefficients["zona.SIT.discrete.facoutside SIT"] +
           gamm.eggs.Losone$coefficients["Year.fac2025"],
         trans = exp,
         ylim = c(0, 15))
```

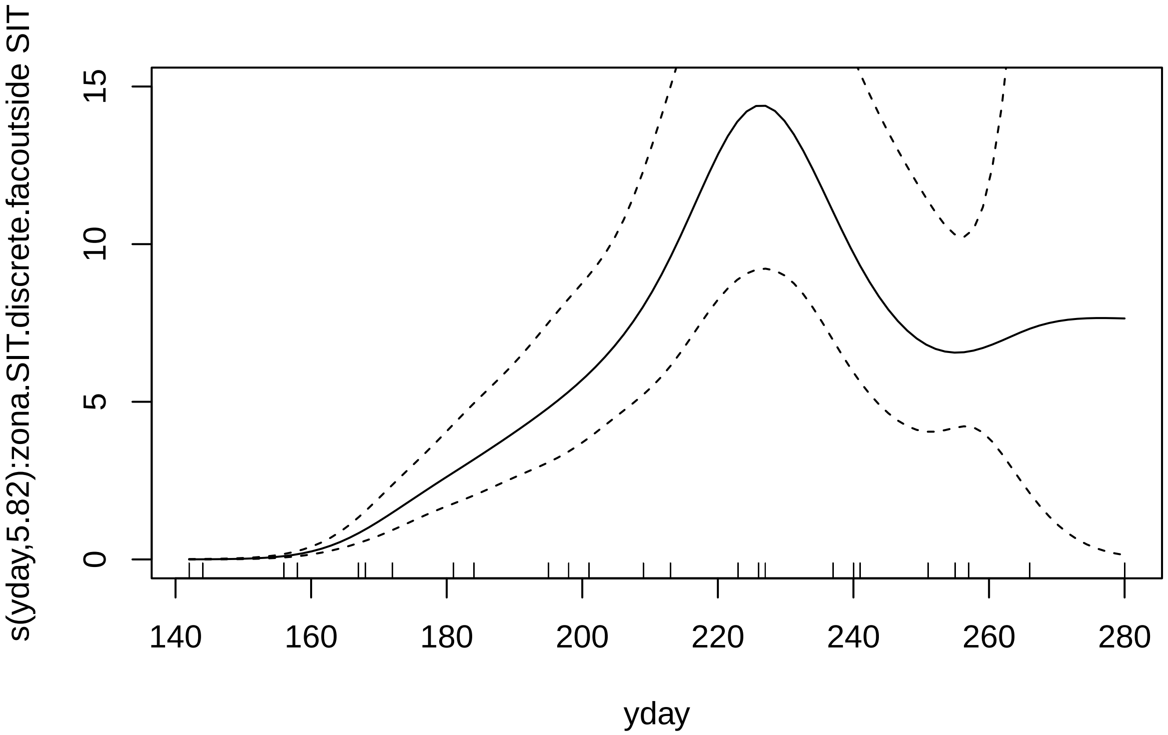

5.3.2 Ascona

We now look at Ascona.

```
plot.gam(gamm.eggs.Ascona,
         pages = 1,
         trans = exp,
         ylim = c(0, 6))
```

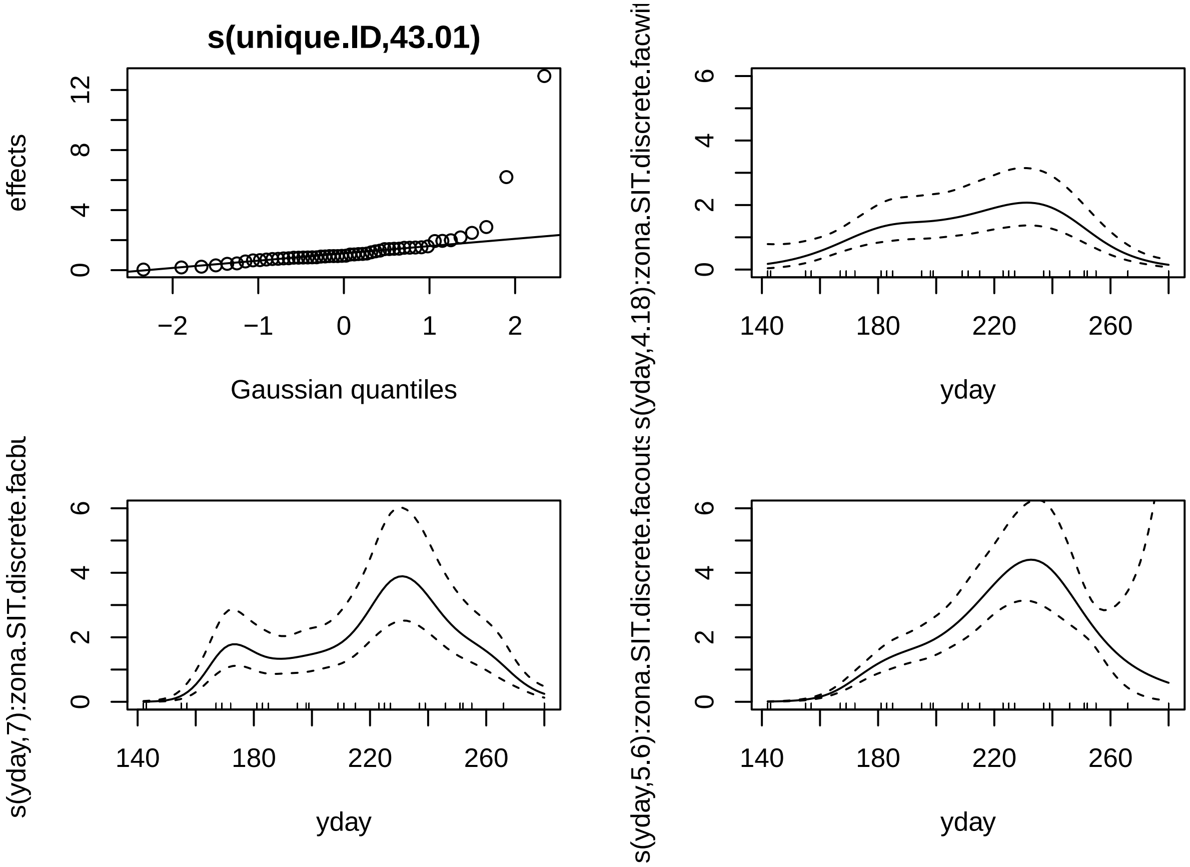

We add the corresponding shift.

```r
## Within SIT, 2025
plot.gam(gamm.eggs.Ascona,
         select = 2,
         shift = gamm.eggs.Ascona$coefficients["(Intercept)"] +
           gamm.eggs.Ascona$coefficients["Year.fac2025"],
         trans = exp,
         ylim = c(0, 20))
```

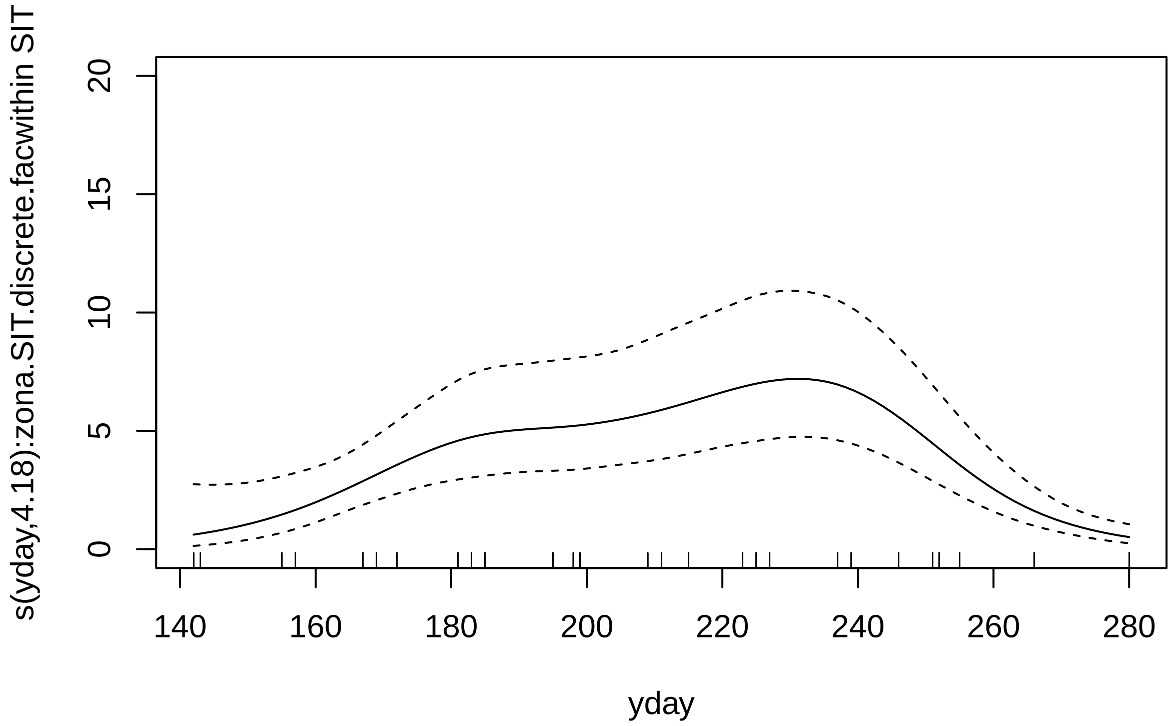


```r
##
## buffer, 2025
plot.gam(gamm.eggs.Ascona,
         select = 3,
         shift = gamm.eggs.Ascona$coefficients["(Intercept)"] +
           gamm.eggs.Ascona$coefficients["zona.SIT.discrete.facbuffer"] +
           gamm.eggs.Ascona$coefficients["Year.fac2025"],
         trans = exp,
         ylim = c(0, 20))
```

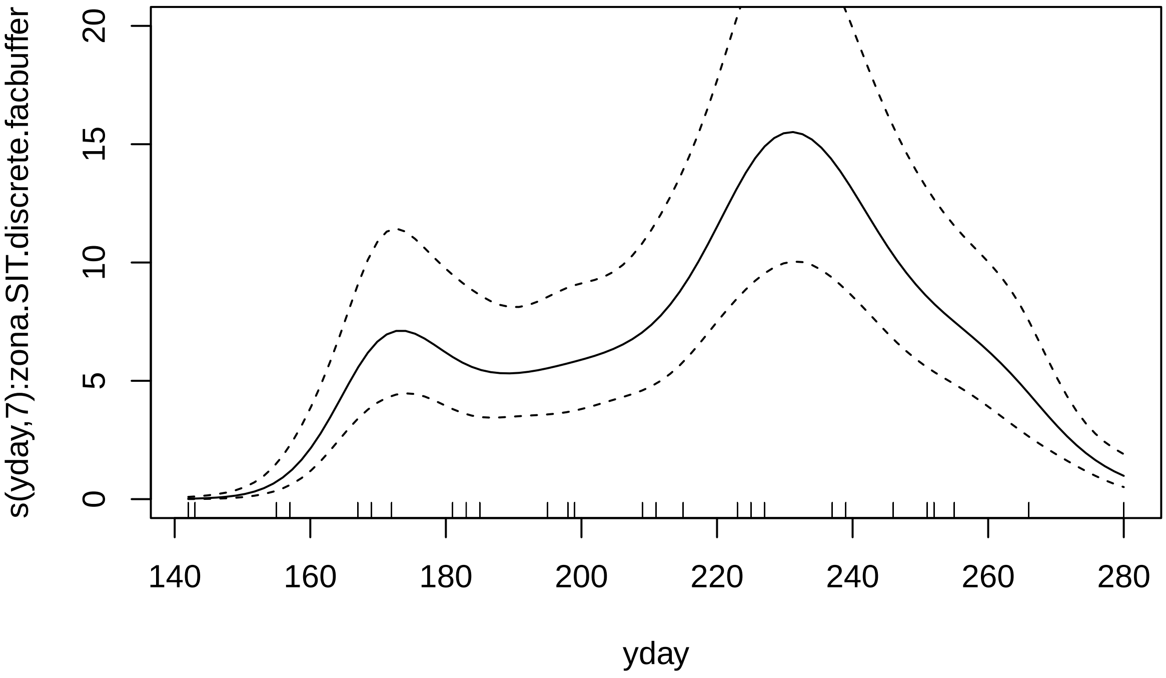

```r
##
## outside SIT, 2025
plot.gam(gamm.eggs.Ascona,
         select = 4,
         shift = gamm.eggs.Ascona$coefficients["(Intercept)"] +
           gamm.eggs.Ascona$coefficients["zona.SIT.discrete.facoutside SIT"] +
           gamm.eggs.Ascona$coefficients["Year.fac2025"],
         trans = exp,
         ylim = c(0, 20))
```

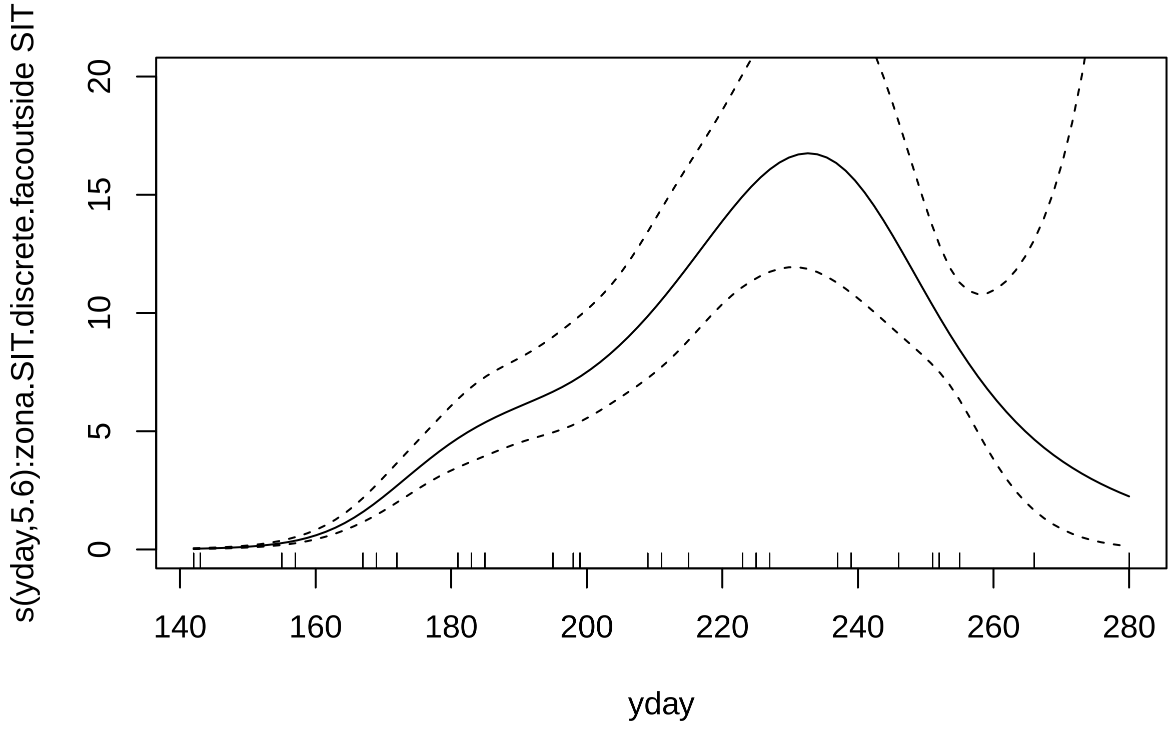

5.4 Model selection

We fitted a model that allows the seasonal trend to vary across treatment zones by including zone-specific smoothers. We now test whether distinct smoothers are necessary.

5.4.1 Losone

For comparing the two models, we need to refit the first model using an ordered factor (*zona.SIT.discrete.ord*) instead of the classical one. This allows us to have nested model matrices and formally compare the two models.

```r
gamm.eggs.Losone_ord <- gamV(total.albopictus.egg ~

                               offset(log(Activation.time)) +
                               s(unique.ID, bs = "re") +

                               ## Fixed effect
                               Year.fac +
                               zona.SIT.discrete.fac +
```

```
                              s(yday) +

                              ## Treatment effect over seasonality
                              s(yday, by = zona.SIT.discrete.ord),


                            family = "nb",
                            method = "GCV.Cp", ## same as gam
                            data = d.ovitraps.temporal.Losone)
##
summary(gamm.eggs.Losone_ord)
```

```
Family: Negative Binomial(0.584)
Link function: log

Formula:
total.albopictus.egg ~ offset(log(Activation.time)) + s(unique.ID,
    bs = "re") + Year.fac + zona.SIT.discrete.fac + s(yday) +
    s(yday, by = zona.SIT.discrete.ord)

Parametric coefficients:
                                      Estimate     Std. Error z value
(Intercept)                     0.289465333087 0.273111677293 1.05988
Year.fac2025                    0.132679385408 0.171964079909 0.77155
zona.SIT.discrete.facbuffer     0.694335268339 0.252424961604 2.75066
zona.SIT.discrete.facoutside SIT 0.374277446573 0.269738523238 1.38756
                                 Pr(>|z|)
(Intercept)                     0.2891996
Year.fac2025                    0.4403793
zona.SIT.discrete.facbuffer     0.0059475 **
zona.SIT.discrete.facoutside SIT 0.1652721
---
Signif. codes:  0 '***' 0.001 '**' 0.01 '*' 0.05 '.' 0.1 ' ' 1

Approximate significance of smooth terms:
                                               edf          Ref.df
s(unique.ID)                          26.93388083319 47.00000000000
s(yday)                                7.38504496484  8.32353510572
s(yday):zona.SIT.discrete.ordbuffer    1.00200639505  1.00384232132
s(yday):zona.SIT.discrete.ordoutside SIT  2.42046348219  3.00524384207
                                        Chi.sq              p-value
s(unique.ID)                          65.75720          0.00000044967 ***
s(yday)                              136.73476 < 0.0000000000000000222 ***
s(yday):zona.SIT.discrete.ordbuffer    1.39462             0.23874152
s(yday):zona.SIT.discrete.ordoutside SIT  17.79147             0.00048488 ***
---
Signif. codes:  0 '***' 0.001 '**' 0.01 '*' 0.05 '.' 0.1 ' ' 1

R-sq.(adj) =   0.39   Deviance explained = 47.4%
-REML = 2533.1  Scale est. = 1         n = 552
```

```
##
gamm.eggs.Losone_one.smooth <- gamV(total.albopictus.egg ~
```

```
                                      offset(log(Activation.time)) +
                                      s(unique.ID, bs = "re") +

                                      ## Fixed effect
                                      Year.fac +
                                      zona.SIT.discrete.fac +

                                      s(yday),# +
                                    ## Treatment effect over seasonality
                                    # s(yday, by = zona.SIT.discrete.ord),


                                    family = "nb",
                                    method = "GCV.Cp", ## same as gam
                                    data = d.ovitraps.temporal.Losone)
##
summary(gamm.eggs.Losone_one.smooth)
```

```
Family: Negative Binomial(0.563)
Link function: log

Formula:
total.albopictus.egg ~ offset(log(Activation.time)) + s(unique.ID,
    bs = "re") + Year.fac + zona.SIT.discrete.fac + s(yday)

Parametric coefficients:
                                          Estimate        Std. Error z value
(Intercept)                       0.285712320859714 0.267175288210523 1.06938
Year.fac2025                      0.000372912890566 0.173568352088332 0.00215
zona.SIT.discrete.facbuffer       0.735066665594993 0.242968273140003 3.02536
zona.SIT.discrete.facoutside SIT 0.405831699044746 0.256367547718064 1.58301
                                  Pr(>|z|)
(Intercept)                       0.2848978
Year.fac2025                      0.9982857
zona.SIT.discrete.facbuffer       0.0024834 **
zona.SIT.discrete.facoutside SIT 0.1134198
---
Signif. codes:  0 '***' 0.001 '**' 0.01 '*' 0.05 '.' 0.1 ' ' 1

Approximate significance of smooth terms:
                        edf          Ref.df    Chi.sq                p-value
s(unique.ID) 25.72173028758 47.00000000000  58.90738           0.0000024937 ***
s(yday)       7.66943689812  8.53007663711 377.43042 < 0.000000000000000222 ***
---
Signif. codes:  0 '***' 0.001 '**' 0.01 '*' 0.05 '.' 0.1 ' ' 1

R-sq.(adj) =   0.368   Deviance explained = 45.3%
-REML = 2539.9  Scale est. = 1          n = 552
```

We check whether the two models are nested. If they are, then we can test whether the most complicated one is necessary, i.e., if the difference of the two models is statistically significant.

```r
## Extract model matrices
m.gamm.eggs.Losone_one.smooth <- model.matrix(gamm.eggs.Losone_one.smooth)
m.gamm.eggs.Losone_ord <- model.matrix(gamm.eggs.Losone_ord)
dim(m.gamm.eggs.Losone_one.smooth)
```

```
[1] 552  61
```

```r
dim(m.gamm.eggs.Losone_ord)
```

```
[1] 552  79
```

```r
##
n1 <- ncol(m.gamm.eggs.Losone_one.smooth)
## check that sum is zero
sum(matrix( m.gamm.eggs.Losone_one.smooth[, 1:n1] - m.gamm.eggs.Losone_ord[, 1:n1] ) != 0)
```

```
[1] 0
```

The two model matrices are nested, therefore we can now compare the two models with the Chi-square test.

```r
chisq.test.Losone <- anova.gam(gamm.eggs.Losone_one.smooth,
                               gamm.eggs.Losone_ord,
                               test = "Chisq") %>%
  print()
```

```
Analysis of Deviance Table

Model 1: total.albopictus.egg ~ offset(log(Activation.time)) + s(unique.ID,
    bs = "re") + Year.fac + zona.SIT.discrete.fac + s(yday)
Model 2: total.albopictus.egg ~ offset(log(Activation.time)) + s(unique.ID,
    bs = "re") + Year.fac + zona.SIT.discrete.fac + s(yday) +
    s(yday, by = zona.SIT.discrete.ord)
       Resid. Df     Resid. Dev             Df       Deviance   Pr(>Chi)
1 500.4284902961 4974.239000398
2 495.0687785113 4951.263617032 5.359711784842 22.97538336608 0.00047048 ***
---
Signif. codes:  0 '***' 0.001 '**' 0.01 '*' 0.05 '.' 0.1 ' ' 1
```

The difference between the two models is statistically significant, which means that the additional flexibility, allowing different shapes for the smoother in each treatment zone, is necessary.

For good practice, we also look at AIC and BIC.

```r
AIC(gamm.eggs.Losone_ord, gamm.eggs.Losone_one.smooth)
```

```
                                          df               AIC
gamm.eggs.Losone_ord        45.6374295959371 5042.53847622357
gamm.eggs.Losone_one.smooth 40.7750761797132 5055.78915275721
```

```r
BIC(gamm.eggs.Losone_ord, gamm.eggs.Losone_one.smooth)
```

```
                                          df               BIC
gamm.eggs.Losone_ord        45.6374295959371 5239.39772149424
gamm.eggs.Losone_one.smooth 40.7750761797132 5231.67440294901
```

AIC and BIC disagree on the need of a more complex model.

5.4.2 Ascona

We do the same but for Ascona.

```
gamm.eggs.Ascona_ord <- gamV(total.albopictus.egg ~

                                 offset(log(Activation.time)) +
                                 s(unique.ID, bs = "re") +

                                 zona.SIT.discrete.fac +
                                 Year.fac +

                                 s(yday) +
                                 ## Treatment effect over seasonality
                                 s(yday, by = zona.SIT.discrete.ord),

                               family = "nb",
                               method = "GCV.Cp", ## same as gam
                               data = d.ovitraps.temporal.Ascona)
summary(gamm.eggs.Ascona_ord)
```

```
Family: Negative Binomial(0.805)
Link function: log

Formula:
total.albopictus.egg ~ offset(log(Activation.time)) + s(unique.ID,
    bs = "re") + zona.SIT.discrete.fac + Year.fac + s(yday) +
    s(yday, by = zona.SIT.discrete.ord)

Parametric coefficients:
                                       Estimate     Std. Error z value
(Intercept)                        0.903245526725 0.296632043286 3.04500
zona.SIT.discrete.facbuffer        0.406134578336 0.315791525511 1.28608
zona.SIT.discrete.facoutside SIT 0.183828040940 0.310546866703 0.59195
Year.fac2025                       0.186301973510 0.130535601089 1.42721
                                    Pr(>|z|)
(Intercept)                        0.0023268 **
zona.SIT.discrete.facbuffer        0.1984136
zona.SIT.discrete.facoutside SIT 0.5538845
Year.fac2025                       0.1535188
---
Signif. codes:  0 '***' 0.001 '**' 0.01 '*' 0.05 '.' 0.1 ' ' 1

Approximate significance of smooth terms:
                                             edf          Ref.df
s(unique.ID)                          43.10477537758 51.00000000000
s(yday)                                7.16551780571  8.18952700659
s(yday):zona.SIT.discrete.ordbuffer       1.00362564966  1.00684322456
s(yday):zona.SIT.discrete.ordoutside SIT  2.43967821865  3.03333978379
                                          Chi.sq                p-value
s(unique.ID)                          348.96861 < 0.000000000000000222 ***
s(yday)                               205.44641 < 0.000000000000000222 ***
s(yday):zona.SIT.discrete.ordbuffer         2.46685                0.11674
s(yday):zona.SIT.discrete.ordoutside SIT  21.87086            0.000069551 ***
```

```
---
Signif. codes:  0 '***' 0.001 '**' 0.01 '*' 0.05 '.' 0.1 ' ' 1

R-sq.(adj) =  -4.67   Deviance explained = 52.5%
-REML = 3177.5  Scale est. = 1         n = 628
```

```r
##
gamm.eggs.Ascona_one.smooth <- gamV(total.albopictus.egg ~

                                      offset(log(Activation.time)) +
                                      s(unique.ID, bs = "re") +

                                      zona.SIT.discrete.fac +
                                      Year.fac +

                                      s(yday), #+
                                    ## Treatment effect over seasonality
                                    # s(yday, by = zona.SIT.discrete.ord),

                                    family = "nb",
                                    method = "GCV.Cp", ## same as gam
                                    data = d.ovitraps.temporal.Ascona)
summary(gamm.eggs.Ascona_one.smooth)
```

```
Family: Negative Binomial(0.777)
Link function: log

Formula:
total.albopictus.egg ~ offset(log(Activation.time)) + s(unique.ID,
    bs = "re") + zona.SIT.discrete.fac + Year.fac + s(yday)

Parametric coefficients:
                                     Estimate     Std. Error z value
(Intercept)                       0.891237145269 0.293056139981 3.04118
zona.SIT.discrete.facbuffer       0.426774470658 0.312302626370 1.36654
zona.SIT.discrete.facoutside SIT  0.175392663722 0.305778572833 0.57359
Year.fac2025                      0.125374977989 0.131861628498 0.95081
                                   Pr(>|z|)
(Intercept)                       0.0023565 **
zona.SIT.discrete.facbuffer       0.1717691
zona.SIT.discrete.facoutside SIT  0.5662428
Year.fac2025                      0.3417023
---
Signif. codes:  0 '***' 0.001 '**' 0.01 '*' 0.05 '.' 0.1 ' ' 1

Approximate significance of smooth terms:
                       edf          Ref.df    Chi.sq                p-value
s(unique.ID) 42.66667673137 51.00000000000 317.27399 < 0.000000000000000222 ***
s(yday)       7.43511687239  8.41192688649 719.26942 < 0.000000000000000222 ***
---
Signif. codes:  0 '***' 0.001 '**' 0.01 '*' 0.05 '.' 0.1 ' ' 1

R-sq.(adj) =  -2.03   Deviance explained = 50.6%
-REML = 3185.2  Scale est. = 1         n = 628
```

We check whether the two models are nested. If they are, then we can test whether the most complicated one is necessary, i.e., if the difference of the two models is statistically significant.

```
## Extract model matrices
m.gamm.eggs.Ascona_one.smooth <- model.matrix(gamm.eggs.Ascona_one.smooth)
m.gamm.eggs.Ascona_ord <- model.matrix(gamm.eggs.Ascona_ord)
dim(m.gamm.eggs.Ascona_one.smooth)
```

```
[1] 628  65
```

```
dim(m.gamm.eggs.Ascona_ord)
```

```
[1] 628  83
```

```
##
n2 <- ncol(m.gamm.eggs.Ascona_one.smooth)
## check that sum is zero
sum(matrix( m.gamm.eggs.Ascona_one.smooth[, 1:n2] - m.gamm.eggs.Ascona_ord[, 1:n2] ) != 0)
```

```
[1] 0
```

The two model matrices are nested, therefore we can now compare the two models with the Chi-square test.

```
chisq.test.Ascona <- anova.gam(gamm.eggs.Ascona_one.smooth,
                               gamm.eggs.Ascona_ord,
                               test = "Chisq") %>%
  print()
```

```
Analysis of Deviance Table

Model 1: total.albopictus.egg ~ offset(log(Activation.time)) + s(unique.ID,
    bs = "re") + zona.SIT.discrete.fac + Year.fac + s(yday)
Model 2: total.albopictus.egg ~ offset(log(Activation.time)) + s(unique.ID,
    bs = "re") + zona.SIT.discrete.fac + Year.fac + s(yday) +
    s(yday, by = zona.SIT.discrete.ord)
      Resid. Df     Resid. Dev             Df       Deviance    Pr(>Chi)
1 565.467851868 6186.302240742
2 560.881961540 6160.655018840 4.585890327943 25.64722190226 0.000069314 ***
---
Signif. codes:  0 '***' 0.001 '**' 0.01 '*' 0.05 '.' 0.1 ' ' 1
```

The difference between the two models is statistically significant, which means that the additional flexibility, allowing different shapes for the smoother in each treatment zone, is necessary.

For good practice, we also look at AIC and BIC.

```
AIC(gamm.eggs.Ascona_ord, gamm.eggs.Ascona_one.smooth)
```

```
                                          df              AIC
gamm.eggs.Ascona_ord        60.5310861841124 6281.71719120798
gamm.eggs.Ascona_one.smooth 56.3044100641073 6298.91106087023
```

```
BIC(gamm.eggs.Ascona_ord, gamm.eggs.Ascona_one.smooth)
```

```
                                          df              BIC
gamm.eggs.Ascona_ord        60.5310861841124 6550.62897290085
gamm.eggs.Ascona_one.smooth 56.3044100641073 6549.04566412933
```

AIC and BIC disagree on the need of a more complex model.

5.5 Fitted values

First, we begin by plotting the fitted values over time, creating a separate graph for each municipality. Observations from the same ovitrap will be connected by lines to visualise the trends and a vertical dashed line is included to show when the SIT releases started (i.e., 17.06.2025).

```
## Losone
d.ovitraps.temporal.Losone$fitted_gamm.eggs <-
  fitted(gamm.eggs.Losone)
## Ascona
d.ovitraps.temporal.Ascona$fitted_gamm.eggs <-
  fitted(gamm.eggs.Ascona)
##
d.ovitraps.fitted <- rbind(d.ovitraps.temporal.Losone,
                           d.ovitraps.temporal.Ascona)
##
ggplot(data = d.ovitraps.fitted,
       mapping = aes(y = fitted_gamm.eggs,
                     x = yday,
                     group = unique.ID,
                     colour = zona.SIT.discrete.fac)) +
  geom_vline(xintercept = yday("2025-06-17"), linetype = "dashed", colour = "grey") +
  geom_hline(yintercept = 0) +
  geom_point(alpha = 0.1) +
  geom_line(alpha = 0.5) +
  scale_y_sqrt() +
  facet_grid(Year.fac ~ municipality.fac)
```

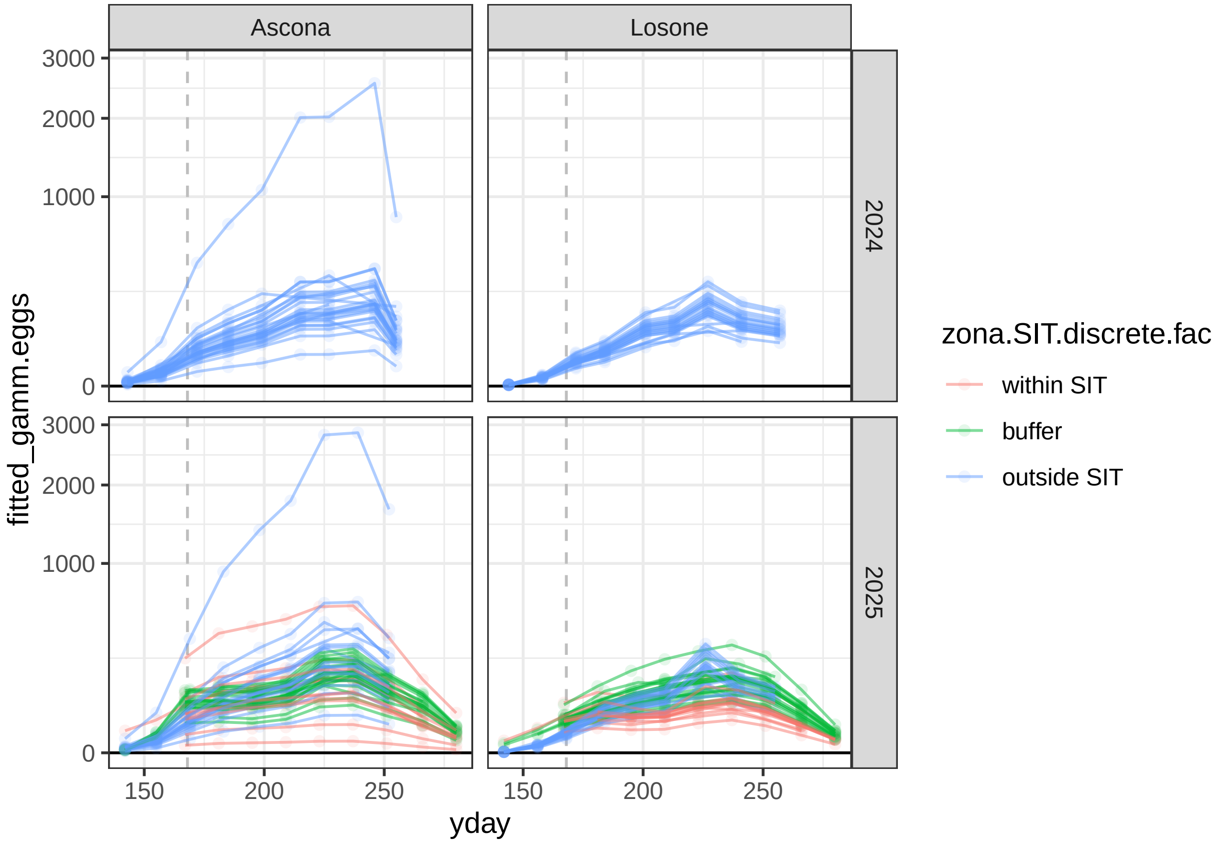

We observe that, in Losone, the fitted values for the level "within SIT" are below those of the other two zones, particularly during the peak season. In Ascona, the differences among zones are less pronounced.

Note that there is significant variability among ovitraps, in particular in Ascona. Specifically, we notice that there is one ovitrap with very high fitted counts. We inspect this observation.

```
d.outlier <- d.ovitraps.temporal.Ascona %>%
  filter(fitted_gamm.eggs > 1000) %>%
  select(unique.ID, zona.SIT.discrete.fac) %>%
  unique() %>%
  print()
```

```
# A tibble: 1 x 2
  unique.ID  zona.SIT.discrete.fac
  <fct>      <fct>
1 Ascona.22a outside SIT
```

```
outlier <- d.outlier$unique.ID
d.ovitraps.temporal.Ascona %>%
  filter(unique.ID == outlier) %>%
  select(Activation.time, yday, Year.fac, total.albopictus.egg, fitted_gamm.eggs) %>%
  arrange(Year.fac, yday)
```

```
# A tibble: 18 x 5
   Activation.time  yday Year.fac total.albopictus.egg fitted_gamm.eggs
             <dbl> <dbl> <fct>                   <dbl>            <dbl>
 1              14   143 2024                       66             5.30
 2              14   157 2024                       24            54.1
 3              15   172 2024                      222           424.
 4              13   185 2024                      146           732.
 5              14   199 2024                      192          1072.
 6              16   215 2024                      297          2011.
 7              12   227 2024                      597          2021.
 8              19   246 2024                      642          2558.
 9               9   255 2024                      255           796.
10              14   142 2025                       25             5.52
11              13   155 2025                      118            44.8
12              14   169 2025                      363           363.
13              14   183 2025                      229           915.
14              15   198 2025                      257          1383.
15              13   211 2025                      176          1771.
16              14   225 2025                      278          2817.
17              14   239 2025                      363          2858.
18              13   252 2025                      141          1653.
```

Let's visualise its trend over the two seasons.

```
ggplot(d.ovitraps.temporal.Ascona %>% filter(zona.SIT.discrete.fac == "outside SIT"),
       mapping = aes(yday, total.albopictus.egg,
                     group = unique.ID,
                     colour = (unique.ID == outlier))) +
  geom_point(alpha = 0.5) +
  geom_line(alpha = 0.5) +
  facet_wrap(~ Year.fac)
```

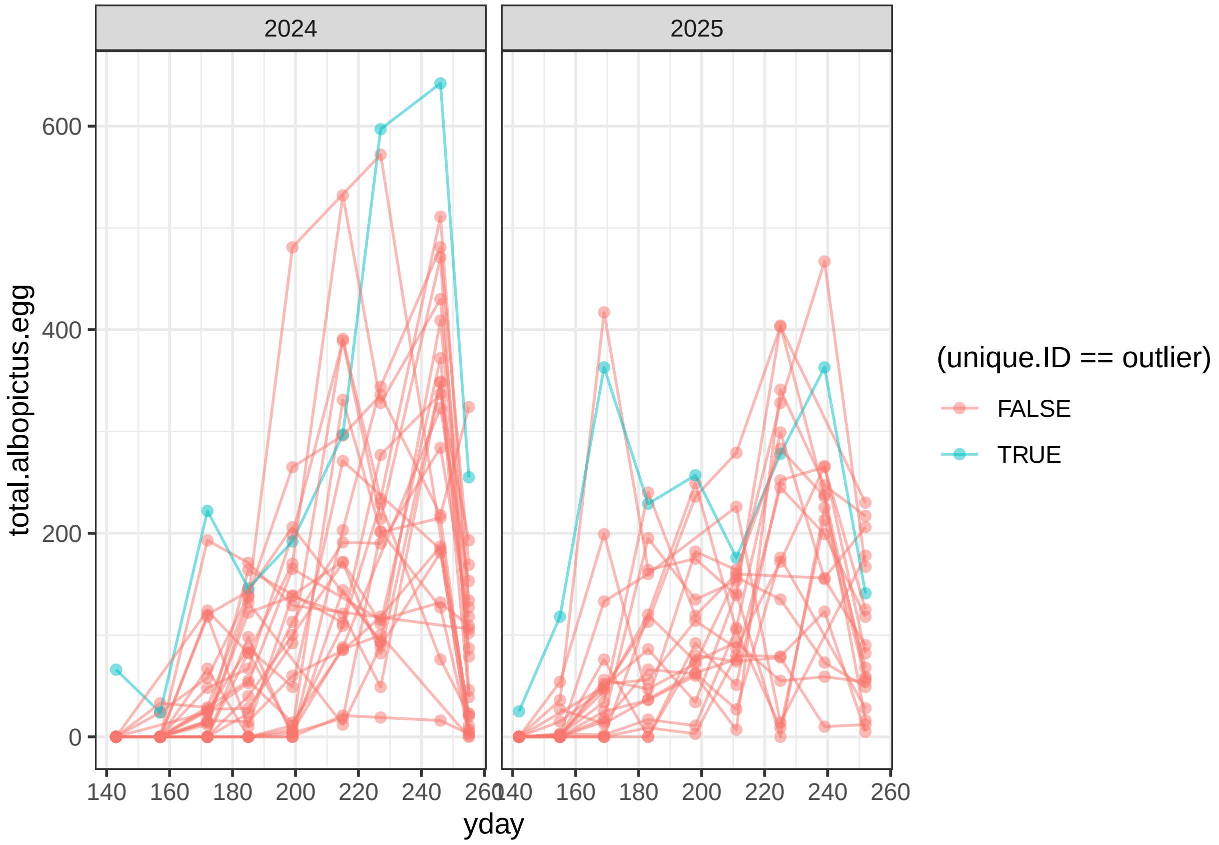

We observe that this ovitrap consistently has high egg counts throughout the season, in particular for the year 2024, which explains the high fitted values. Note, importantly, that this ovitrap has non-zero counts where all other ovitraps do at the very beginning of the season. This is expected to drive the estimation as we deal with a multiplicative model due to the canonical link (i.e., log()). We check the random effect.

```
ranef.gamm.eggs.Ascona <- coef(gamm.eggs.Ascona)[names(coef(gamm.eggs.Ascona)) %>%
                                                   str_detect(pattern = "unique.ID")]

ranef.gamm.eggs.Ascona[levels(d.ovitraps.temporal.Ascona$unique.ID) == outlier]
```

```
 s(unique.ID).28
2.55957820246172
```

```
##
## 0.95 quantile of ranef.ranef.gamm.eggs.Ascona
quantile(ranef.gamm.eggs.Ascona, probs = 0.95)
```

```
              95%
0.974428347331463
```

We see that this random effect is very high compared to the others.

5.6 Predicted values

We will now create the dataset for prediction. Our goal is to predict the total number of eggs laid over the entire season, both at the ovitrap and population levels. This step is necessary to account for differences in activation times among observations, ensuring data comparability.

```r
## (messages are omitted from this chunk)
##
formula(gamm.eggs.Losone)
```

```
total.albopictus.egg ~ offset(log(Activation.time)) + s(unique.ID,
    bs = "re") + zona.SIT.discrete.fac + Year.fac + s(yday, by = zona.SIT.discrete.fac)
```

```r
##
## 1. We create a data set with unique.ID and zona.SIT.discrete.fac.
## We do it separately because not in all zona.SIT.discrete.fac all ovitraps are present.
## We keep municipality to then separate the data set based on this variable.
d.muni.ovitrap.short <- d.ovitraps.fitted %>%
  group_by(municipality.fac, zona.SIT.discrete.fac) %>%
  mutate(yday.min = min(yday, na.rm = TRUE),
         yday.max = max(yday, na.rm = TRUE)) %>%
  select(municipality.fac, unique.ID, Year.fac, zona.SIT.discrete.fac,
         yday.min, yday.max) %>%
  unique()
##
head(d.muni.ovitrap.short)
```

```
# A tibble: 6 x 6
# Groups:   municipality.fac, zona.SIT.discrete.fac [1]
  municipality.fac unique.ID Year.fac zona.SIT.discrete.fac yday.min yday.max
  <fct>            <fct>     <fct>    <fct>                    <dbl>    <dbl>
1 Losone           Losone.1b 2024     outside SIT                142      257
2 Losone           Losone.2a 2024     outside SIT                142      257
3 Losone           Losone.3a 2024     outside SIT                142      257
4 Losone           Losone.4b 2024     outside SIT                142      257
5 Losone           Losone.5a 2024     outside SIT                142      257
6 Losone           Losone.6b 2024     outside SIT                142      257
```

```r
nrow(d.muni.ovitrap.short)
```

```
[1] 145
```

```r
##
## 2. We create a data set with varying yday for each ovitrap ID
d.pred.gamm <- expand.grid(
  yday = seq(from = min(d.ovitraps.fitted$yday),
             to = max(d.ovitraps.fitted$yday),
             length.out = 100),
  unique.ID = d.muni.ovitrap.short$unique.ID,
  Year.fac = levels(d.ovitraps.fitted$Year.fac),
  ## We set the activation time equal to 14 days, i.e. two weeks.
  Activation.time = 14)
##
## 3. We join the two data sets.
## Note that not all ovitraps exist in all municipalities.
## So, we can't simply use expand.grid()
d.pred.gamm_aug <- left_join(x = d.pred.gamm, d.muni.ovitrap.short) %>%
  ## we don't want to predict outside the range of available data for each
  ## municipality, thus we filter the data set based on the min and max yday
  ## available for each municipality.
  filter(yday >= yday.min & yday <= yday.max)
str(d.pred.gamm_aug)
```

```
'data.frame':	20692 obs. of  8 variables:
 $ yday                 : num  142 143 145 146 148 ...
 $ unique.ID            : Factor w/ 100 levels "Ascona.1","Ascona.10",..: 74 74 74 74 74 74 74 74 74 74
 $ Year.fac             : Factor w/ 2 levels "2024","2025": 1 1 1 1 1 1 1 1 1 1 ...
 $ Activation.time      : num  14 14 14 14 14 14 14 14 14 14 ...
 $ municipality.fac     : Factor w/ 2 levels "Ascona","Losone": 2 2 2 2 2 2 2 2 2 2 ...
 $ zona.SIT.discrete.fac: Factor w/ 3 levels "within SIT","buffer",..: 3 3 3 3 3 3 3 3 3 3 ...
 $ yday.min             : num  142 142 142 142 142 142 142 142 142 142 ...
 $ yday.max             : num  257 257 257 257 257 257 257 257 257 257 ...
 - attr(*, "out.attrs")=List of 2
  ..$ dim     : Named int [1:4] 100 145 2 1
  .. ..- attr(*, "names")= chr [1:4] "yday" "unique.ID" "Year.fac" "Activation.time"
  ..$ dimnames:List of 4
  .. ..$ yday           : chr [1:100] "yday=142.000000000000" "yday=143.393939393939" "yday=144.78787878
  .. ..$ unique.ID      : chr [1:145] "unique.ID=Losone.1b" "unique.ID=Losone.2a" "unique.ID=Losone.3a"
  .. ..$ Year.fac       : chr [1:2] "Year.fac=2024" "Year.fac=2025"
  .. ..$ Activation.time: chr "Activation.time=14"
```

We make the predictions on the newly created data set, at ovitrap and population level, and we plot the result.

```r
## prediction at ovitrap level
d.pred.gamm_aug.Losone <- d.pred.gamm_aug %>%
  filter(municipality.fac == "Losone") %>%
  droplevels()
d.pred.gamm_aug.Losone$predicted_gamm.tot.eggs <- predict(
  gamm.eggs.Losone,
  type = "response",
  newdata = d.pred.gamm_aug.Losone)
##
d.pred.gamm_aug.Ascona <- d.pred.gamm_aug %>%
  filter(municipality.fac == "Ascona") %>%
  droplevels()
d.pred.gamm_aug.Ascona$predicted_gamm.tot.eggs <- predict(
  gamm.eggs.Ascona,
  type = "response",
  newdata = d.pred.gamm_aug.Ascona)
##
## prediction at population level
d.pred.gamm_aug.Losone$predicted_gamm.eggs.pop <- predict(
  gamm.eggs.Losone,
  newdata = d.pred.gamm_aug.Losone,
  type = "response",
  exclude = 's(unique.ID)')
##
d.pred.gamm_aug.Ascona$predicted_gamm.eggs.pop <- predict(
  gamm.eggs.Ascona,
  newdata = d.pred.gamm_aug.Ascona,
  type = "response",
  exclude = 's(unique.ID)')
##
## Join the two data sets
d.pred.gamm_aug <- rbind(d.pred.gamm_aug.Losone,
```

```
                              d.pred.gamm_aug.Ascona)
```

```
gg.prediction.gamm.Losone <- ggplot(data = d.pred.gamm_aug.Losone,
                                    mapping = aes(y = predicted_gamm.tot.eggs,
                                                  x = yday,
                                                  group = unique.ID)) +
  geom_vline(xintercept = yday("2025-06-17"), linetype = "dashed", colour = "grey") +
  geom_hline(yintercept = 0) +
  geom_line(alpha = 0.2) +
  scale_y_sqrt() +
  geom_line(mapping = aes(y = predicted_gamm.eggs.pop),
            colour = "red") +
  facet_wrap(zona.SIT.discrete.fac ~ Year.fac) +
  geom_rug(data = d.ovitraps.temporal.Losone, mapping = aes(x = yday, y = NA),
           alpha = 0.5)
gg.prediction.gamm.Losone +
  labs(title = "Losone")
```

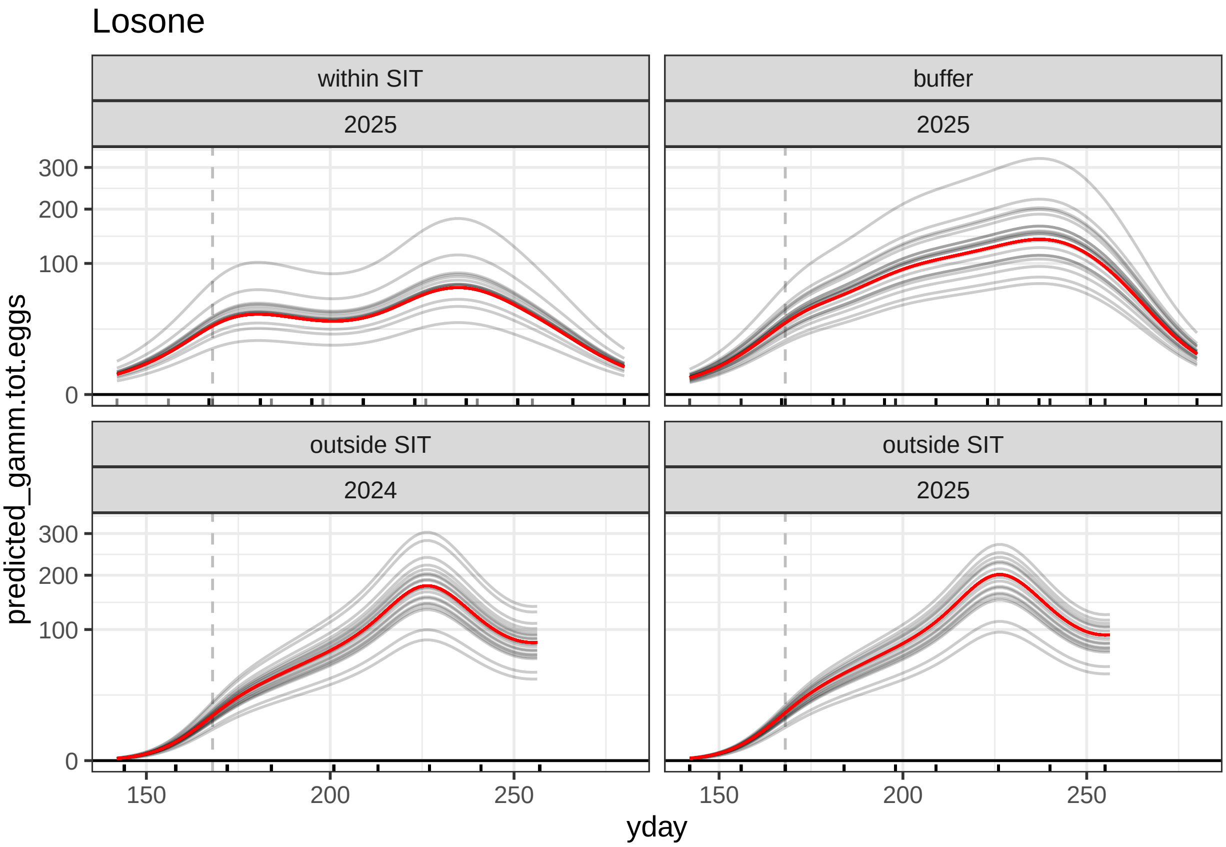


```
gg.prediction.gamm.Ascona <- ggplot(data = d.pred.gamm_aug.Ascona,
                                    mapping = aes(y = predicted_gamm.tot.eggs,
                                                  x = yday,
                                                  group = unique.ID)) +
  geom_vline(xintercept = yday("2025-06-17"), linetype = "dashed", colour = "grey") +
  geom_hline(yintercept = 0) +
  geom_line(alpha = 0.2) +
  scale_y_sqrt() +
  geom_line(mapping = aes(y = predicted_gamm.eggs.pop),
```

```r
          colour = "red") +
  facet_wrap(zona.SIT.discrete.fac ~ Year.fac) +
  geom_rug(data = d.ovitraps.temporal.Ascona, mapping = aes(x = yday, y = NA),
         alpha = 0.5)
gg.prediction.gamm.Ascona +
  labs(title = "Ascona")
```

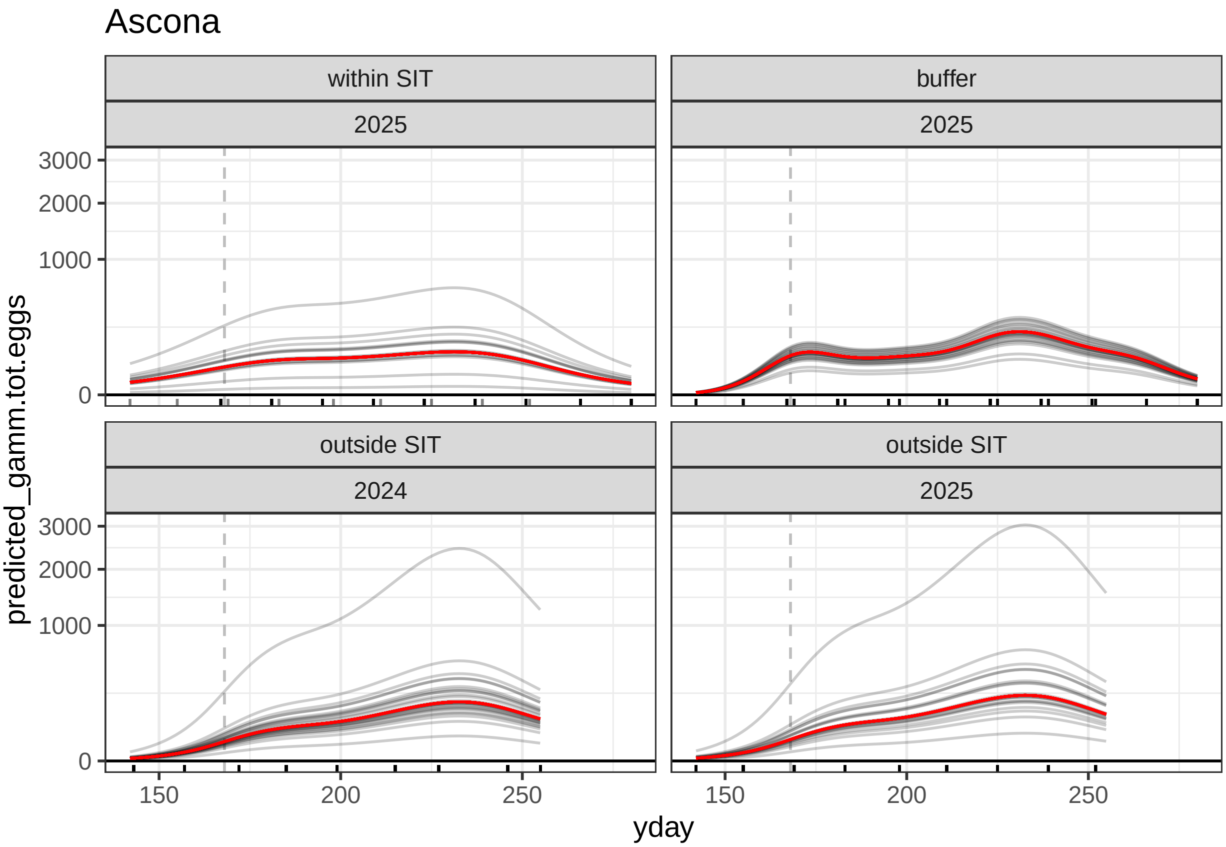

Each black line corresponds to the prediction for a given ovitrap, whereas the red line corresponds to the prediction at population level.

We plot the predictions at population level on the same plot. The year 2024 is only included for a better model fit, thus we can remove it in the following plot because we are only interested in comparing the treatment zones for the year 2025.

```r
gg.prediction.gamm.together <- ggplot(data = filter(d.pred.gamm_aug,
                                                    Year.fac == "2025"),
                                      mapping = aes(y = predicted_gamm.eggs.pop,
                                                    x = yday,
                                                    colour = zona.SIT.discrete.fac)) +
  geom_vline(xintercept = yday(as.Date("2025-06-17")),
             linetype = "dashed", colour = "grey") +
  geom_line() +
  geom_hline(yintercept = 0, colour = "grey") +
  facet_wrap(~ municipality.fac) +
  labs(colour = "Zone") +
  theme(legend.position = "bottom")
gg.prediction.gamm.together
```

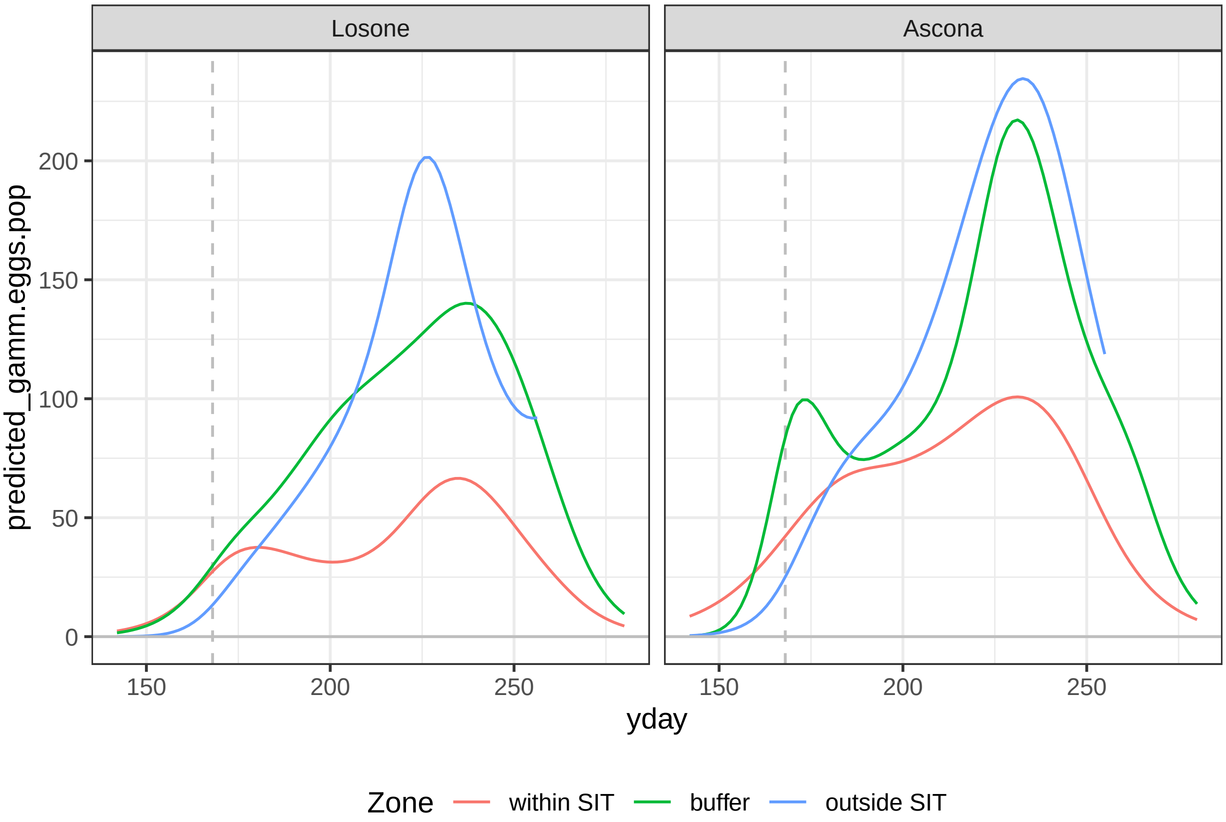

We observe that, in Ascona, the predicted egg counts start out higher in the treated zone than in the other two zones, whereas in Losone they start out lower in the untreated zone than in the others. However, once the treatment begins, the trends clearly diverge, with the treated zone showing consistently lower predicted counts than the other two zones throughout the season.

In particular, we notice that the treated zones in both municipalities begin to show a clear treatment effect only a few days after the start of the intervention. The effect appears to persist until around day 200, after which the egg counts begin to rise again. However, the peak remains much lower compared to that observed in the other two zones.

The treatment also seems to have an effect in the buffer zones. Specifically, in Ascona, the buffer zone starts with much higher egg counts compared to the outside zone, but as soon as the treatment begins, the number of eggs drops. Here as well, after day 200, the egg counts start to increase again, and the seasonal peak is very similar to that of the outside zone. However, based on how the season starts, it seems that the peak could have been much higher without the treatment. In Losone, the buffer zone also starts with higher egg counts compared to the outside zone, but later the peak is lower.

Note that we did not plot the confidence bands here because we already have three curves overlaid. Adding the confidence bands would make the plot too cluttered and difficult to read. Instead, we will focus on plotting the differences between zones in the next section, along with the confidence bands, which will provide a clearer picture of the treatment effects.

5.6.1 Plotting the differences

We now plot the predicted differences between egg counts in the treatment zones throughout the season, separately for each municipality.

We are no longer interested in the prediction at ovitrap level, but only at population level. Thus, we create a new data set containing only the necessary information for this purpose.

```r
## We want to predict at population level, thus we don't care about the random
## effects. This means that we just need one observation per group.
d.pred.gamm_aug_pop <- d.pred.gamm_aug %>%
  group_by(yday, Year.fac, Activation.time, municipality.fac, zona.SIT.discrete.fac) %>%
  slice(1) %>%
  ungroup()
##
## We need to start the comparison where all zones have available data.
d.pred.gamm_aug_pop <- d.pred.gamm_aug_pop %>%
  filter(yday >= max(yday.min) & yday <= min(yday.max)) %>%
  select(-yday.min, -yday.max)
```

5.6.1.1 Buffer versus within SIT zone – Losone We start by looking at the difference between the buffer and the treated zone in Losone.

We create the data set on which we are going to calculate the contrasts. We make sure that we first have all the observations belonging to the "within SIT" level, followed by those of the "buffer" level.

```r
d.pred.gamm_aug.Losone.25.SIT.buffer <- d.pred.gamm_aug_pop %>%
  filter(municipality.fac == "Losone") %>%
  filter(Year.fac == "2025" &
           zona.SIT.discrete.fac %in% c("within SIT", "buffer")) %>%
  droplevels() %>%
  arrange(zona.SIT.discrete.fac, yday)
##
## check
d.pred.gamm_aug.Losone.25.SIT.buffer %>%
  pull(zona.SIT.discrete.fac)
```

```
  [1] within SIT within SIT within SIT within SIT within SIT within SIT
  [7] within SIT within SIT within SIT within SIT within SIT within SIT
 [13] within SIT within SIT within SIT within SIT within SIT within SIT
 [19] within SIT within SIT within SIT within SIT within SIT within SIT
 [25] within SIT within SIT within SIT within SIT within SIT within SIT
 [31] within SIT within SIT within SIT within SIT within SIT within SIT
 [37] within SIT within SIT within SIT within SIT within SIT within SIT
 [43] within SIT within SIT within SIT within SIT within SIT within SIT
 [49] within SIT within SIT within SIT within SIT within SIT within SIT
 [55] within SIT within SIT within SIT within SIT within SIT within SIT
 [61] within SIT within SIT within SIT within SIT within SIT within SIT
 [67] within SIT within SIT within SIT within SIT within SIT within SIT
 [73] within SIT within SIT within SIT within SIT within SIT within SIT
 [79] within SIT within SIT within SIT within SIT buffer     buffer
 [85] buffer     buffer     buffer     buffer     buffer     buffer
 [91] buffer     buffer     buffer     buffer     buffer     buffer
 [97] buffer     buffer     buffer     buffer     buffer     buffer
[103] buffer     buffer     buffer     buffer     buffer     buffer
[109] buffer     buffer     buffer     buffer     buffer     buffer
[115] buffer     buffer     buffer     buffer     buffer     buffer
[121] buffer     buffer     buffer     buffer     buffer     buffer
[127] buffer     buffer     buffer     buffer     buffer     buffer
[133] buffer     buffer     buffer     buffer     buffer     buffer
[139] buffer     buffer     buffer     buffer     buffer     buffer
```

```
[145] buffer     buffer     buffer     buffer     buffer     buffer
[151] buffer     buffer     buffer     buffer     buffer     buffer
[157] buffer     buffer     buffer     buffer     buffer     buffer
[163] buffer     buffer
Levels: within SIT buffer
```

```r
## makes sense
```

Next, we create the model matrix for calculating the contrasts.

```r
## Model matrix
W.predictions.buffer.sit.Losone <- predict(gamm.eggs.Losone,
                                           newdata = d.pred.gamm_aug.Losone.25.SIT.buffer,
                                           type = "lpmatrix",

                                           ## Prediction at population level
                                           exclude = 's(unique.ID)')
```

We also extract the coefficients of the model and the corresponding variance-covariance matrix.

```r
c.coeff.gamm.eggs.Losone <- coef(gamm.eggs.Losone)
vcov.c.coeff.gamm.eggs.Losone <- vcov(gamm.eggs.Losone)
```

We can now compute the contrasts.

```r
## 1) We count the number of rows of the matrix representing the "within SIT" level
n <- d.pred.gamm_aug.Losone.25.SIT.buffer %>%
  filter(zona.SIT.discrete.fac == "within SIT") %>%
  nrow() %>%
  print()
```

```
[1] 82
```

```r
##
## check that "buffer" matrix has the same dimension
d.pred.gamm_aug.Losone.25.SIT.buffer %>%
  filter(zona.SIT.discrete.fac == "buffer") %>%
  nrow()
```

```
[1] 82
```

```r
## makes sense

## 2) We calculate the contrasts, exploiting the model matrix.

##  i) We calculate the contrast matrix L as (X_buffer - X_withinSIT)

## We made sure that we first have all the "within SIT" observations,
## and then all the "buffer" ones., thus we can do as follows:
L.buffer.Losone  <- W.predictions.buffer.sit.Losone[(n + 1):(2*n), , drop = FALSE] -
  W.predictions.buffer.sit.Losone[1:n, , drop = FALSE]

##  ii) multiply L by beta.
## => eta = L * beta
est.buffer.Losone <- (L.buffer.Losone %*% c.coeff.gamm.eggs.Losone) %>%
  as.vector()
```

Now that we have computed the contrasts, we can calculate the corresponding standard error.

```r
## 3) We calculate the corresponding standard error, which is given by
## the variance of L*beta
## Var(L * beta) = diag(L %*% Var(beta) %*% t(L))
## and then we take the sqrt for the standard error
## the following code computes it
se.buffer.Losone  <-
  ( (L.buffer.Losone %*% vcov.c.coeff.gamm.eggs.Losone) * L.buffer.Losone ) %>%
  rowSums() %>%
  sqrt()
## equivalent to
# ( (L.buffer.Losone %*% vcov.c.coeff.gamm.eggs.Losone) %*% t(L.buffer.Losone) ) %>%
#   diag() %>%
#   sqrt()
## but requires less computing power
```

We store everything in a data frame and plot the results.

```r
d.diff.buffer.Losone <- data.frame(
  yday = d.pred.gamm_aug.Losone$yday[1:n],
  diff = est.buffer.Losone,
  lo = est.buffer.Losone - qnorm(0.975) * se.buffer.Losone,
  hi = est.buffer.Losone + qnorm(0.975) * se.buffer.Losone) %>%
  mutate(exp.diff = exp(diff),
         exp.lo = exp(lo),
         exp.hi = exp(hi))

ggplot(d.diff.buffer.Losone, mapping = aes(x = yday, y = diff)) +
  geom_ribbon(aes(ymin = lo, ymax = hi), fill = "lightgray", alpha = 0.5) +
  geom_line(linewidth = 1) +
  geom_hline(yintercept = 0, linetype = "dashed") +
  geom_vline(xintercept = yday("2025-06-17"), linetype = "dashed", colour = "grey") +
  labs(title = "Losone",
       y = "buffer minus treated zone") +
  coord_cartesian(ylim = c(-1, 3))
```

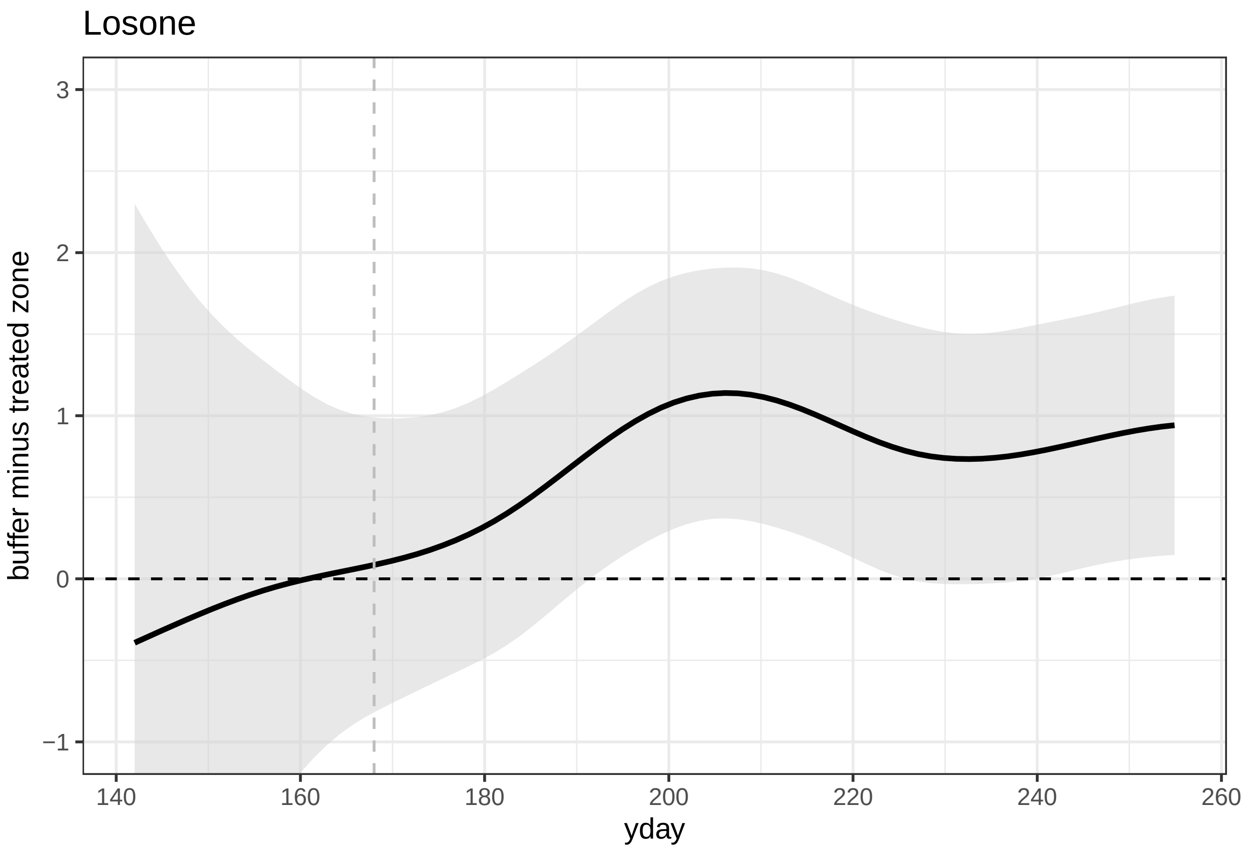

The plot shows the difference between the predicted values on the linear predictor scale for the buffer and treated zones, along with a 95% confidence interval. A horizontal dashed line at 0 indicates no difference between the two zones, while a vertical dashed line marks the start of the treatment on June 17, 2025.

We back-transform the difference by applying the exponential function to return it to the original scale. After this transformation, the values can be interpreted as ratios of predicted egg counts: a value of 1 indicates no difference between zones, values greater than 1 indicate higher predicted egg counts in the buffer zone relative to the treated zone, and values below 1 indicate the opposite.

```
gg.buffer.losone.exp <- ggplot(d.diff.buffer.Losone, mapping = aes(x = yday, y = exp.diff)) +
  geom_ribbon(aes(ymin = exp.lo, ymax = exp.hi), fill = "lightgray", alpha = 0.5) +
  geom_line(linewidth = 1) +
  geom_hline(yintercept = 1, linetype = "dashed") +
  geom_vline(xintercept = yday("2025-06-17"), linetype = "dashed", colour = "grey") +
  labs(title = "Losone",
       y = "buffer minus treated zone \nin the back transformed scale")
gg.buffer.losone.exp
```

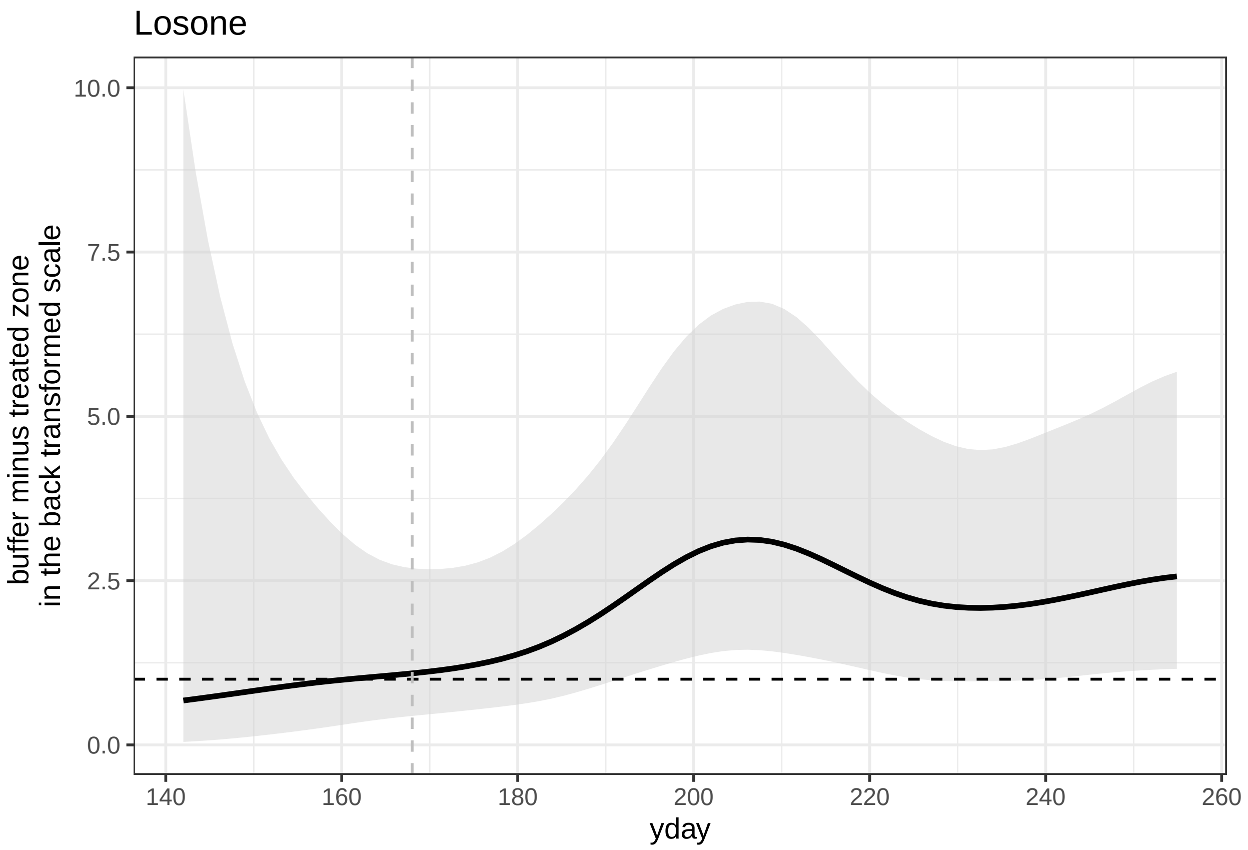

We zoom in to focus on the most relevant part of the graph.

```
## we zoom in
gg.buffer.losone.exp +
  coord_cartesian(ylim = c(0, 5))
```

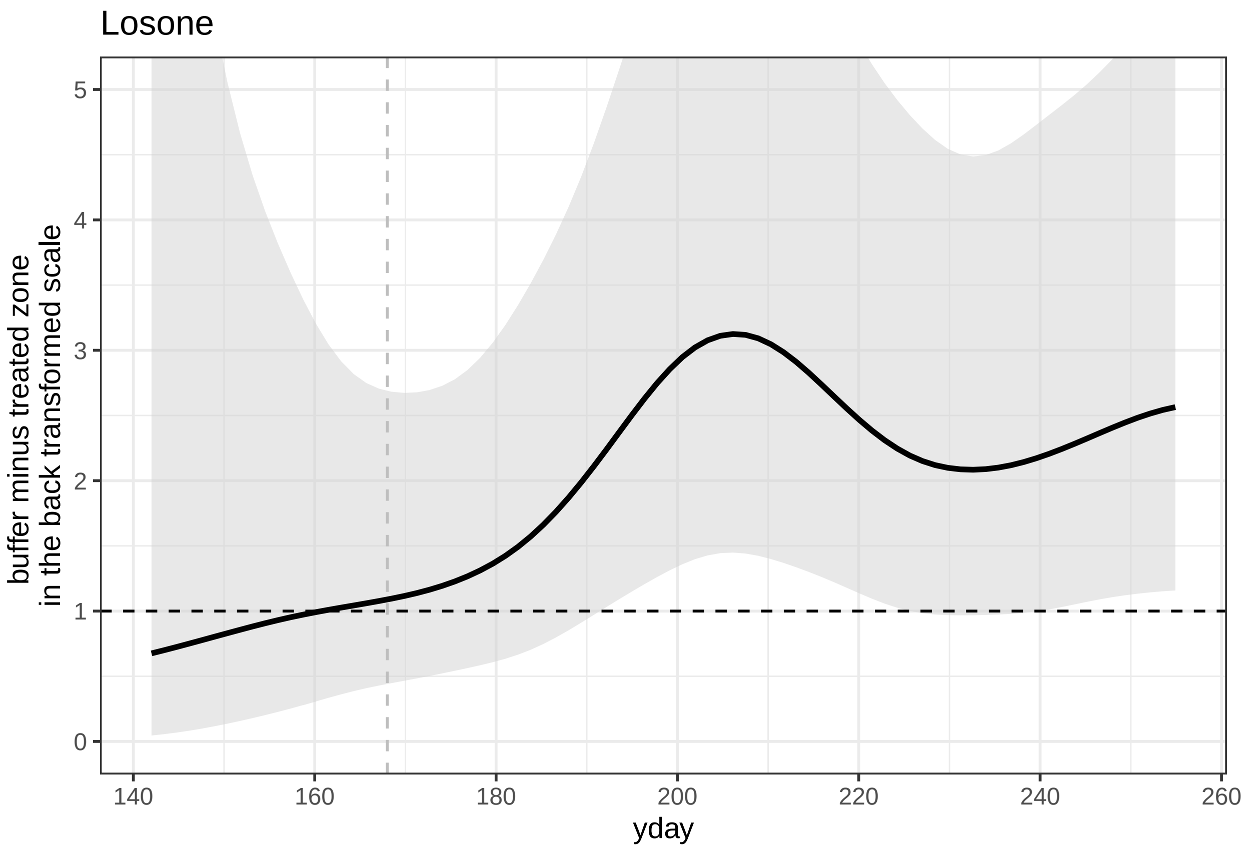

Egg counts start slightly higher in the treated compared to the buffer zone. However, after the treatment begins, the situation reverses, and the buffer zone consistently shows higher predicted egg counts than the treated zone. The difference reaches its peak around day 210, when the predicted egg count in the buffer zone is `3.1` times the egg count predicted in the treated zone.

Note that the difference between the two zones is statistically significant when the grey zone doesn't intersect with the horizontal dashed line at 1.

5.6.1.2 Buffer versus within SIT zone – Ascona

We redo the same exact process, but for Ascona. For the full explanation, look at the section above.

```
d.pred.gamm_aug.Ascona.25.SIT.buffer <- d.pred.gamm_aug_pop %>%
  filter(municipality.fac == "Ascona") %>%
  filter(Year.fac == "2025" &
           zona.SIT.discrete.fac %in% c("within SIT", "buffer")) %>%
  droplevels() %>%
  arrange(zona.SIT.discrete.fac, yday)
##
## check
d.pred.gamm_aug.Ascona.25.SIT.buffer %>%
  pull(zona.SIT.discrete.fac)
```

```
  [1] within SIT within SIT within SIT within SIT within SIT within SIT
  [7] within SIT within SIT within SIT within SIT within SIT within SIT
 [13] within SIT within SIT within SIT within SIT within SIT within SIT
 [19] within SIT within SIT within SIT within SIT within SIT within SIT
 [25] within SIT within SIT within SIT within SIT within SIT within SIT
```

```
 [31] within SIT within SIT within SIT within SIT within SIT within SIT
 [37] within SIT within SIT within SIT within SIT within SIT within SIT
 [43] within SIT within SIT within SIT within SIT within SIT within SIT
 [49] within SIT within SIT within SIT within SIT within SIT within SIT
 [55] within SIT within SIT within SIT within SIT within SIT within SIT
 [61] within SIT within SIT within SIT within SIT within SIT within SIT
 [67] within SIT within SIT within SIT within SIT within SIT within SIT
 [73] within SIT within SIT within SIT within SIT within SIT within SIT
 [79] within SIT within SIT within SIT within SIT buffer     buffer
 [85] buffer     buffer     buffer     buffer     buffer     buffer
 [91] buffer     buffer     buffer     buffer     buffer     buffer
 [97] buffer     buffer     buffer     buffer     buffer     buffer
[103] buffer     buffer     buffer     buffer     buffer     buffer
[109] buffer     buffer     buffer     buffer     buffer     buffer
[115] buffer     buffer     buffer     buffer     buffer     buffer
[121] buffer     buffer     buffer     buffer     buffer     buffer
[127] buffer     buffer     buffer     buffer     buffer     buffer
[133] buffer     buffer     buffer     buffer     buffer     buffer
[139] buffer     buffer     buffer     buffer     buffer     buffer
[145] buffer     buffer     buffer     buffer     buffer     buffer
[151] buffer     buffer     buffer     buffer     buffer     buffer
[157] buffer     buffer     buffer     buffer     buffer     buffer
[163] buffer     buffer
Levels: within SIT buffer
```

```
## makes sense
```

```
## Model matrix
W.predictions.buffer.sit.Ascona <- predict(gamm.eggs.Ascona,
                                           newdata = d.pred.gamm_aug.Ascona.25.SIT.buffer,
                                           type = "lpmatrix",

                                           ## Prediction at population level
                                           exclude = 's(unique.ID)')
##
c.coeff.gamm.eggs.Ascona <- coef(gamm.eggs.Ascona)
vcov.c.coeff.gamm.eggs.Ascona <- vcov(gamm.eggs.Ascona)


## Calculate the contrasts

## First, count the rows of the matrix containing only "within SIT"
n2 <- d.pred.gamm_aug.Ascona.25.SIT.buffer %>%
  filter(zona.SIT.discrete.fac == "within SIT") %>%
  nrow() %>%
  print()
```

```
[1] 82
```

```
##
## check that "buffer" has the same dimension
d.pred.gamm_aug.Ascona.25.SIT.buffer %>%
  filter(zona.SIT.discrete.fac == "buffer") %>%
  nrow()
```

```
[1] 82
```

```r
## makes sense

## Calculate the contrasts
L.buffer.Ascona  <- W.predictions.buffer.sit.Ascona[(n2 + 1):(2*n2), , drop = FALSE] -
  W.predictions.buffer.sit.Ascona[1:n2, , drop = FALSE]
est.buffer.Ascona <- as.vector(L.buffer.Ascona %*% c.coeff.gamm.eggs.Ascona)

## Now, we calculate the corresponding standard error
se.buffer.Ascona  <-
  ( (L.buffer.Ascona %*% vcov.c.coeff.gamm.eggs.Ascona) * L.buffer.Ascona) %>%
  rowSums() %>%
  sqrt()

d.diff.buffer.Ascona <- data.frame(
  yday = d.pred.gamm_aug.Ascona$yday[1:n2],
  diff = est.buffer.Ascona,
  lo = est.buffer.Ascona - qnorm(0.975) * se.buffer.Ascona,
  hi = est.buffer.Ascona + qnorm(0.975) * se.buffer.Ascona
) %>%
  mutate(exp.diff = exp(diff),
         exp.lo = exp(lo),
         exp.hi = exp(hi))
```

We plot the result.

```r
ggplot(d.diff.buffer.Ascona, mapping = aes(x = yday, y = diff)) +
  geom_ribbon(aes(ymin = lo, ymax = hi), fill = "lightgray", alpha = 0.5) +
  geom_line(linewidth = 1) +
  geom_hline(yintercept = 0, linetype = "dashed") +
  geom_vline(xintercept = yday("2025-06-17"), linetype = "dashed", colour = "grey") +
  labs(title = "Ascona",
       y = "buffer minus treated zone")
```

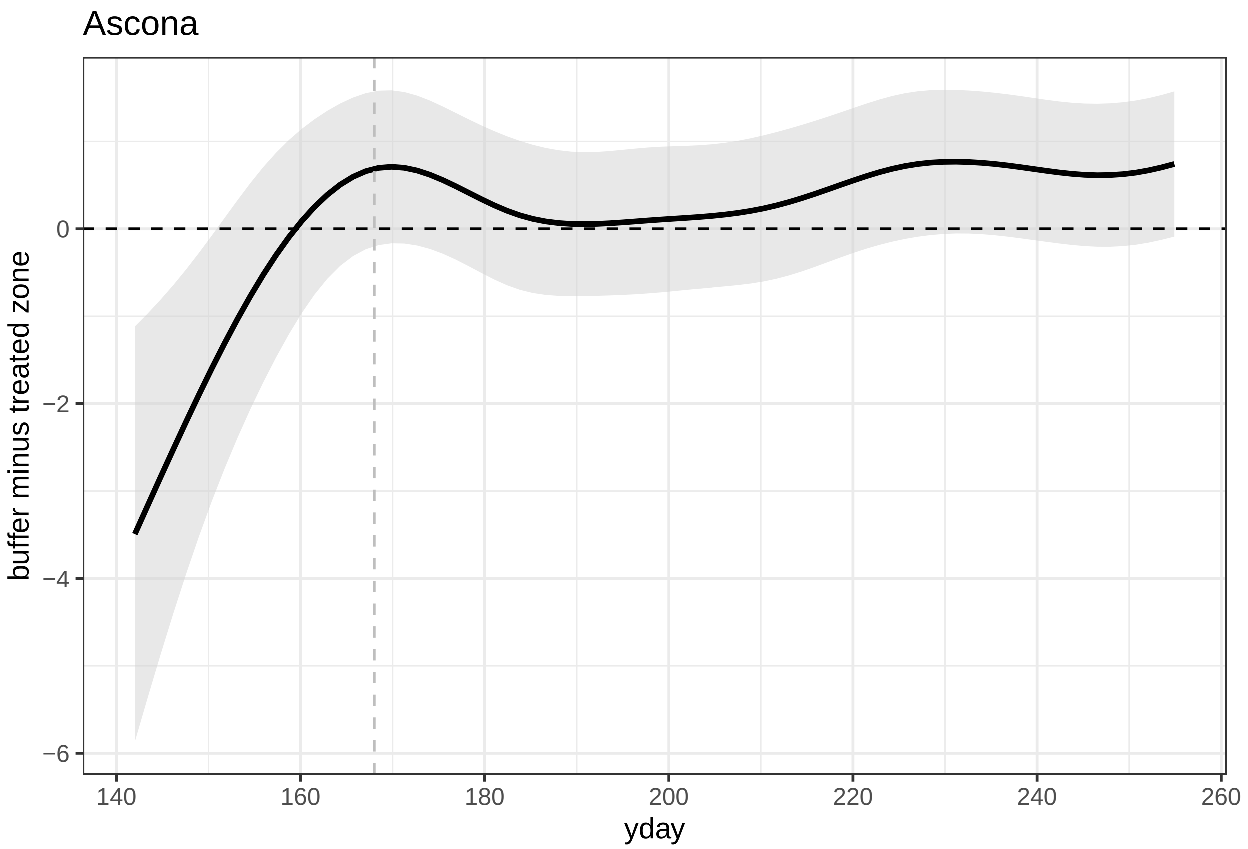

The plot shows the difference between the predicted values on the linear predictor scale for the buffer and treated zones, along with a 95% confidence interval. A horizontal dashed line at 0 indicates no difference between the two zones, while a vertical dashed line marks the start of the treatment on June 17, 2025.

We back-transform the difference by applying the exponential function to return it to the original scale. After this transformation, the values can be interpreted as ratios of predicted egg counts: a value of 1 indicates no difference between zones, values greater than 1 indicate higher predicted egg counts in the buffer zone relative to the treated zone, and values below 1 indicate the opposite.

```
gg.buffer.Ascona <- ggplot(d.diff.buffer.Ascona, mapping = aes(x = yday, y = exp.diff)) +
  geom_ribbon(aes(ymin = exp.lo, ymax = exp.hi), fill = "lightgray", alpha = 0.5) +
  geom_line(linewidth = 1) +
  geom_hline(yintercept = 1, linetype = "dashed") +
  geom_vline(xintercept = yday("2025-06-17"), linetype = "dashed", colour = "grey") +
  labs(title = "Ascona",
       y = "buffer minus treated zone \nin the back transformed scale")
gg.buffer.Ascona
```

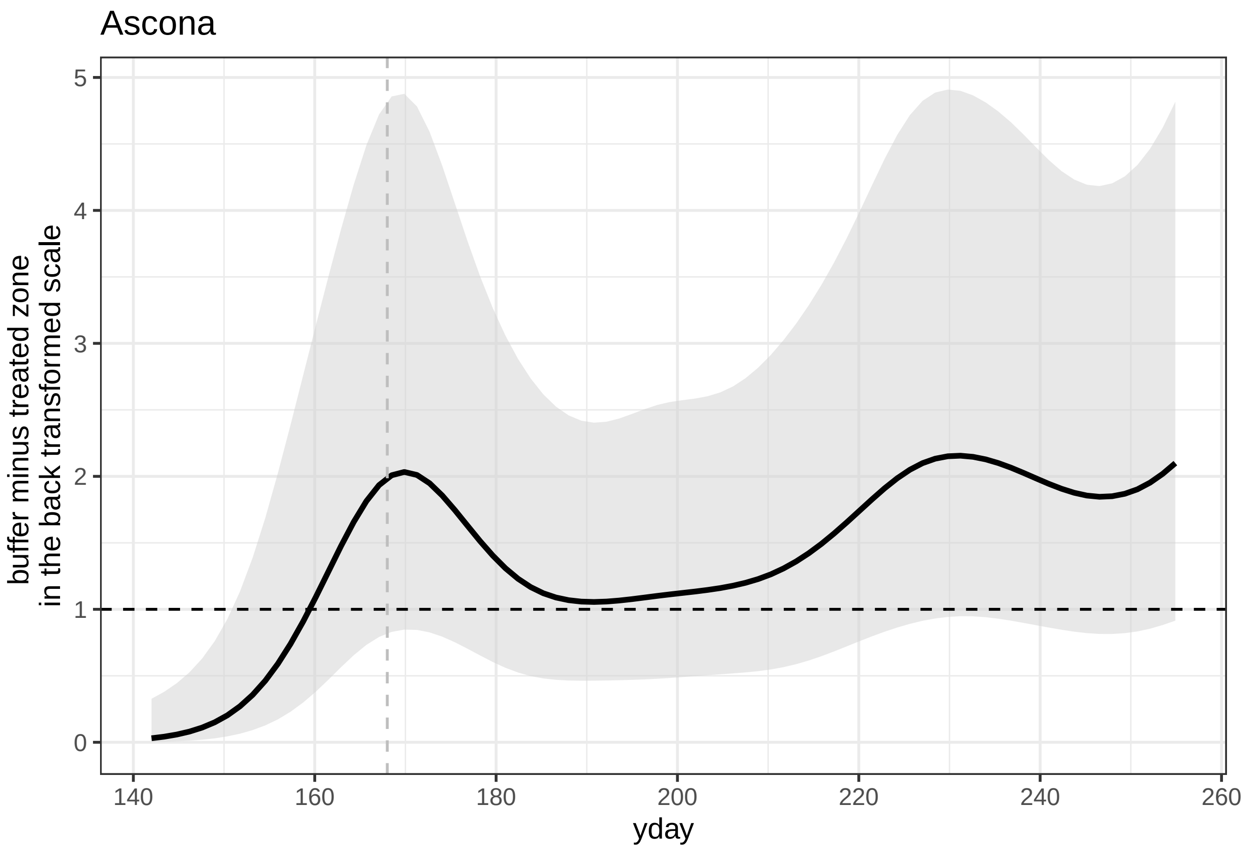

We zoom in to concentrate on the most relevant part of the graph. We use the same zoom as for the previous plot to better compare the plots.

```
## we zoom in
gg.buffer.Ascona +
  coord_cartesian(ylim = c(0, 5))
```

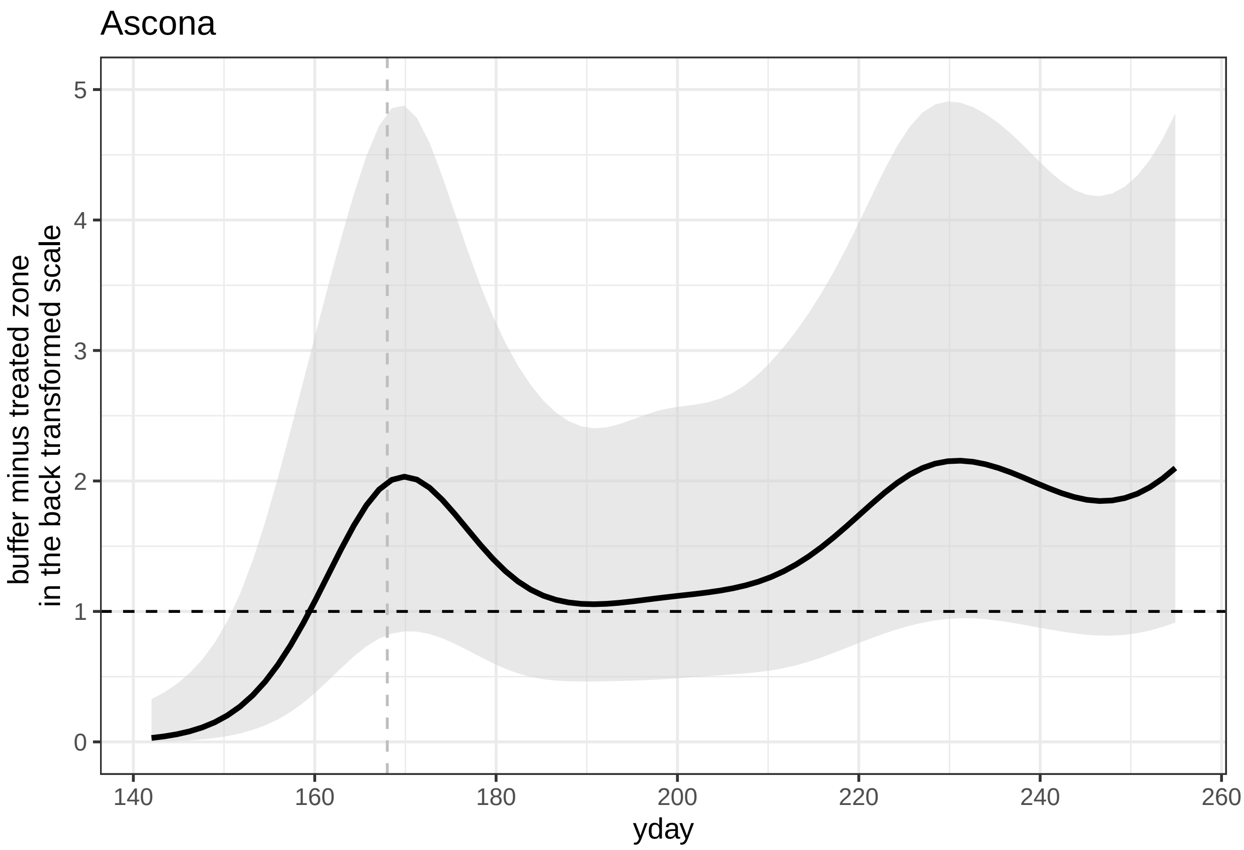

The treated zone starts by having higher predicted egg counts compared to the buffer zone. However, the difference quickly reverses, and after the treatment begins, the buffer zone always exhibits higher predicted egg counts than the treated zone.

Note that the difference between the two zones is statistically significant when the grey zone doesn't intersect with the horizontal dashed line at 1.

5.6.1.3 outside SIT versus within SIT zone – Losone

We now perform the same steps as in the previous two sections, but replacing the buffer area with the untreated zone. We start with Losone.

```
d.pred.gamm_aug.Losone.25.SIT.outside <- d.pred.gamm_aug_pop %>%
  filter(municipality.fac == "Losone") %>%
  filter(Year.fac == "2025" &
           zona.SIT.discrete.fac %in% c("within SIT", "outside SIT")) %>%
  droplevels() %>%
  arrange(zona.SIT.discrete.fac, yday)
##
## check
d.pred.gamm_aug.Losone.25.SIT.outside %>%
  pull(zona.SIT.discrete.fac)
```

```
 [1] within SIT  within SIT  within SIT  within SIT  within SIT  within SIT
 [7] within SIT  within SIT  within SIT  within SIT  within SIT  within SIT
[13] within SIT  within SIT  within SIT  within SIT  within SIT  within SIT
[19] within SIT  within SIT  within SIT  within SIT  within SIT  within SIT
[25] within SIT  within SIT  within SIT  within SIT  within SIT  within SIT
[31] within SIT  within SIT  within SIT  within SIT  within SIT  within SIT
```

```
 [37] within SIT  within SIT  within SIT  within SIT  within SIT  within SIT
 [43] within SIT  within SIT  within SIT  within SIT  within SIT  within SIT
 [49] within SIT  within SIT  within SIT  within SIT  within SIT  within SIT
 [55] within SIT  within SIT  within SIT  within SIT  within SIT  within SIT
 [61] within SIT  within SIT  within SIT  within SIT  within SIT  within SIT
 [67] within SIT  within SIT  within SIT  within SIT  within SIT  within SIT
 [73] within SIT  within SIT  within SIT  within SIT  within SIT  within SIT
 [79] within SIT  within SIT  within SIT  within SIT  outside SIT outside SIT
 [85] outside SIT outside SIT outside SIT outside SIT outside SIT outside SIT
 [91] outside SIT outside SIT outside SIT outside SIT outside SIT outside SIT
 [97] outside SIT outside SIT outside SIT outside SIT outside SIT outside SIT
[103] outside SIT outside SIT outside SIT outside SIT outside SIT outside SIT
[109] outside SIT outside SIT outside SIT outside SIT outside SIT outside SIT
[115] outside SIT outside SIT outside SIT outside SIT outside SIT outside SIT
[121] outside SIT outside SIT outside SIT outside SIT outside SIT outside SIT
[127] outside SIT outside SIT outside SIT outside SIT outside SIT outside SIT
[133] outside SIT outside SIT outside SIT outside SIT outside SIT outside SIT
[139] outside SIT outside SIT outside SIT outside SIT outside SIT outside SIT
[145] outside SIT outside SIT outside SIT outside SIT outside SIT outside SIT
[151] outside SIT outside SIT outside SIT outside SIT outside SIT outside SIT
[157] outside SIT outside SIT outside SIT outside SIT outside SIT outside SIT
[163] outside SIT outside SIT
Levels: within SIT outside SIT
```

```r
## makes sense
```

```r
## Model matrix
W.predictions.outside.sit <- predict(gamm.eggs.Losone,
                                     newdata = d.pred.gamm_aug.Losone.25.SIT.outside,
                                     type = "lpmatrix",

                                     ## Prediction at population level
                                     exclude = 's(unique.ID)')

## Calculate the contrasts

## First, count the rows of the matrix containing only "within SIT"
n3 <- d.pred.gamm_aug.Losone.25.SIT.outside %>%
  filter(zona.SIT.discrete.fac == "within SIT") %>%
  nrow() %>%
  print()
```

```
[1] 82
```

```r
##
## check that "outside SIT" has the same dimension
d.pred.gamm_aug.Losone.25.SIT.outside %>%
  filter(zona.SIT.discrete.fac == "outside SIT") %>%
  nrow()
```

```
[1] 82
```

```r
## makes sense

## Calculate the estimates of the contrasts, starting from the model matrix.
## Proceed as follows: first calculate L, then multiply it by beta.
## L.outside.Losone * beta = (X_outside SIT - X_withinSIT) * beta
```

```r
L.outside.Losone  <- W.predictions.outside.sit[(n3 + 1):(2*n3), , drop = FALSE] -
  W.predictions.outside.sit[1:n3, , drop = FALSE]
est.outside.Losone <- as.vector(L.outside.Losone %*% c.coeff.gamm.eggs.Losone)

## Now, we calculate the corresponding standard error
se.outside.Losone  <-
  ( (L.outside.Losone %*% vcov.c.coeff.gamm.eggs.Losone) * L.outside.Losone ) %>%
  rowSums() %>%
  sqrt()

d.diff.outside.Losone <- data.frame(
  yday = d.pred.gamm_aug.Losone$yday[1:n3],
  diff = est.outside.Losone,
  lo = est.outside.Losone - qnorm(0.975) * se.outside.Losone,
  hi = est.outside.Losone + qnorm(0.975) * se.outside.Losone
) %>%
  mutate(exp.diff = exp(diff),
         exp.lo = exp(lo),
         exp.hi = exp(hi))
```

We plot the result.

```r
ggplot(d.diff.outside.Losone, mapping = aes(x = yday, y = diff)) +
  geom_ribbon(aes(ymin = lo, ymax = hi), fill = "lightgray", alpha = 0.5) +
  geom_line(linewidth = 1) +
  geom_hline(yintercept = 0, linetype = "dashed") +
  geom_vline(xintercept = yday("2025-06-17"), linetype = "dashed", colour = "grey") +
  labs(title = "Losone",
       y = "outside SIT minus treated zone")
```

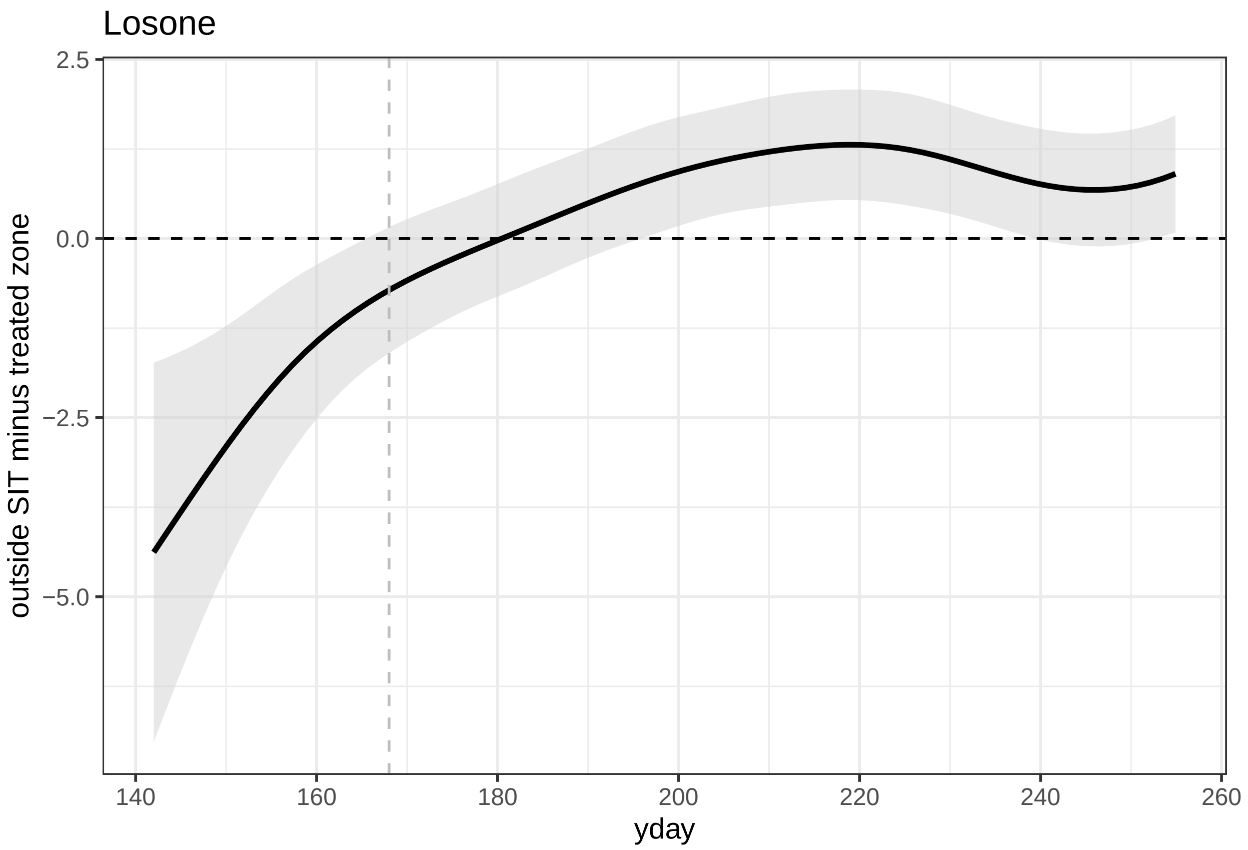

The plot shows the difference between the predicted values on the linear predictor scale for the untreated and treated zones, along with a 95% confidence interval. A horizontal dashed line at 0 indicates no difference between the two zones, while a vertical dashed line marks the start of the treatment on June 17, 2025.

We back-transform the difference by applying the exponential function to return it to the original scale. After this transformation, the values can be interpreted as ratios of predicted egg counts: a value of 1 indicates no difference between zones, values greater than 1 indicate higher predicted egg counts in the untreated zone relative to the treated zone, and values below 1 indicate the opposite.

```
##
gg.outside.Losone <- ggplot(d.diff.outside.Losone, mapping = aes(x = yday, y = exp.diff)) +
  geom_ribbon(aes(ymin = exp.lo, ymax = exp.hi), fill = "lightgray", alpha = 0.5) +
  geom_line(linewidth = 1) +
  geom_hline(yintercept = 1, linetype = "dashed") +
  geom_vline(xintercept = yday("2025-06-17"), linetype = "dashed", colour = "grey") +
  labs(title = "Losone",
       y = "outside SIT minus treated zone \nin the back transformed scale")
gg.outside.Losone
```

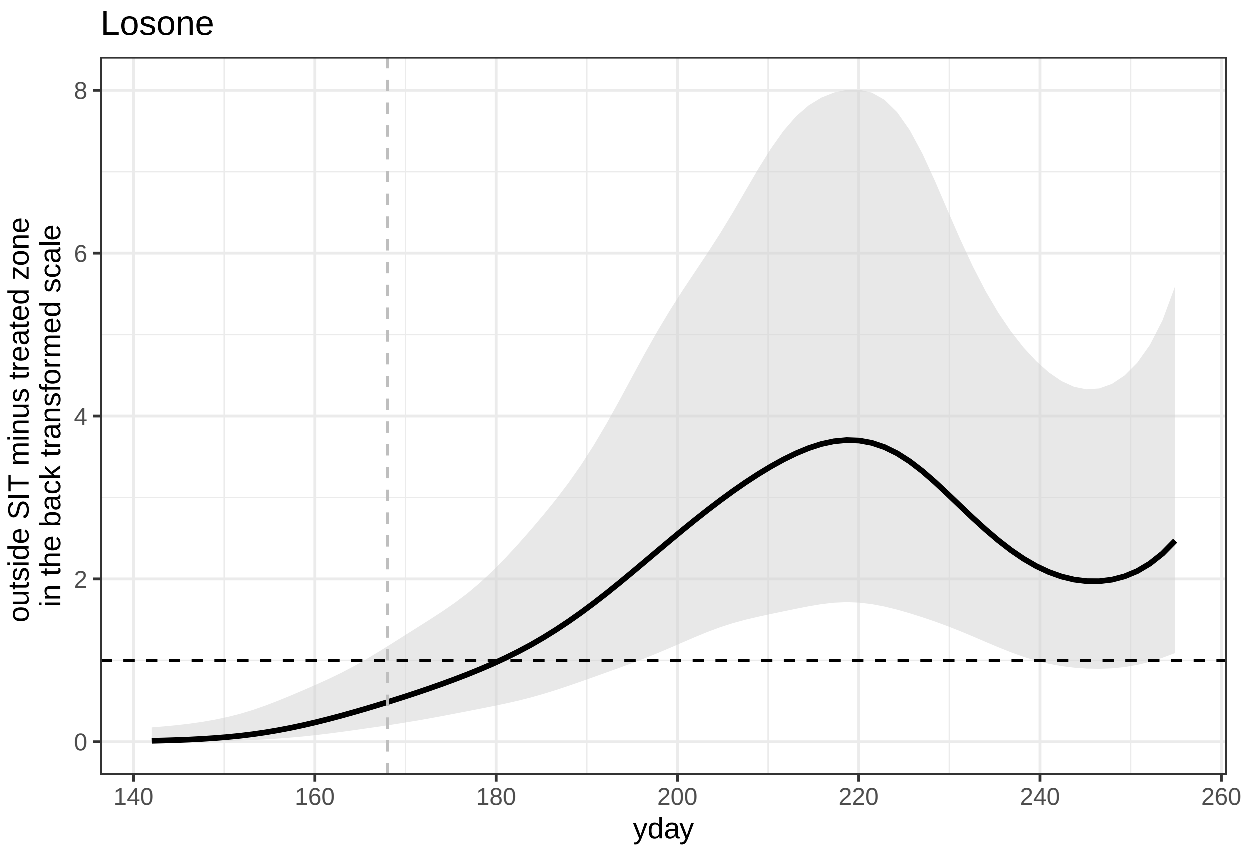

We zoom in to focus on the most relevant part of the graph. We use the same zoom as for the previous plots to better compare them.

```
## we zoom in
gg.outside.Losone +
  coord_cartesian(ylim = c(0, 5))
```

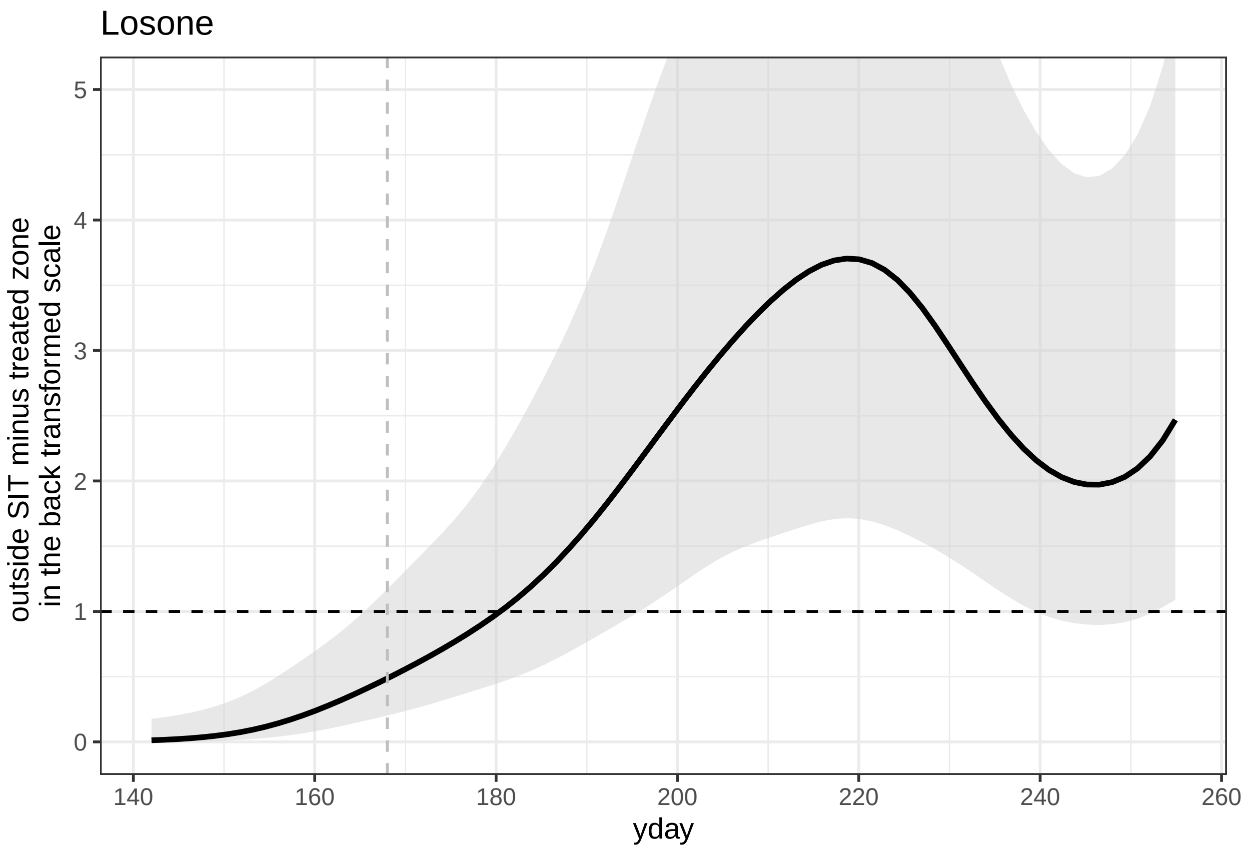

The treated zone initially shows higher predicted egg counts than the untreated zone. However, this difference quickly reverses, and after the treatment begins, the untreated zone consistently exhibits higher predicted egg counts than the treated zone. This difference grows over time, reaching its peak around day 220, when the predicted egg count in the untreated zone is `3.7` times the egg count in the treated zone.

Note that the difference between the two zones is statistically significant when the grey zone doesn't intersect with the horizontal dashed line at 1.

5.6.1.4 outside SIT versus within SIT zone – Ascona

We calculate the last contrast, comparing the treated and untreated zones in Ascona.

```
d.pred.gamm_aug.Ascona.25.SIT.outside <- d.pred.gamm_aug_pop %>%
  filter(municipality.fac == "Ascona") %>%
  filter(Year.fac == "2025" &
           zona.SIT.discrete.fac %in% c("within SIT", "outside SIT")) %>%
  droplevels() %>%
  arrange(zona.SIT.discrete.fac, yday)
##
## check
d.pred.gamm_aug.Ascona.25.SIT.outside %>%
  pull(zona.SIT.discrete.fac)
```

```
 [1] within SIT  within SIT  within SIT  within SIT  within SIT  within SIT
 [7] within SIT  within SIT  within SIT  within SIT  within SIT  within SIT
[13] within SIT  within SIT  within SIT  within SIT  within SIT  within SIT
[19] within SIT  within SIT  within SIT  within SIT  within SIT  within SIT
[25] within SIT  within SIT  within SIT  within SIT  within SIT  within SIT
```

```
 [31] within SIT  within SIT  within SIT  within SIT  within SIT  within SIT 
 [37] within SIT  within SIT  within SIT  within SIT  within SIT  within SIT 
 [43] within SIT  within SIT  within SIT  within SIT  within SIT  within SIT 
 [49] within SIT  within SIT  within SIT  within SIT  within SIT  within SIT 
 [55] within SIT  within SIT  within SIT  within SIT  within SIT  within SIT 
 [61] within SIT  within SIT  within SIT  within SIT  within SIT  within SIT 
 [67] within SIT  within SIT  within SIT  within SIT  within SIT  within SIT 
 [73] within SIT  within SIT  within SIT  within SIT  within SIT  within SIT 
 [79] within SIT  within SIT  within SIT  within SIT  outside SIT outside SIT
 [85] outside SIT outside SIT outside SIT outside SIT outside SIT outside SIT
 [91] outside SIT outside SIT outside SIT outside SIT outside SIT outside SIT
 [97] outside SIT outside SIT outside SIT outside SIT outside SIT outside SIT
[103] outside SIT outside SIT outside SIT outside SIT outside SIT outside SIT
[109] outside SIT outside SIT outside SIT outside SIT outside SIT outside SIT
[115] outside SIT outside SIT outside SIT outside SIT outside SIT outside SIT
[121] outside SIT outside SIT outside SIT outside SIT outside SIT outside SIT
[127] outside SIT outside SIT outside SIT outside SIT outside SIT outside SIT
[133] outside SIT outside SIT outside SIT outside SIT outside SIT outside SIT
[139] outside SIT outside SIT outside SIT outside SIT outside SIT outside SIT
[145] outside SIT outside SIT outside SIT outside SIT outside SIT outside SIT
[151] outside SIT outside SIT outside SIT outside SIT outside SIT outside SIT
[157] outside SIT outside SIT outside SIT outside SIT outside SIT outside SIT
[163] outside SIT outside SIT
Levels: within SIT outside SIT
```

```
## makes sense
```

```
## Model matrix
W.predictions.outside.sit <- predict(gamm.eggs.Ascona,
                                     newdata = d.pred.gamm_aug.Ascona.25.SIT.outside,
                                     type = "lpmatrix",

                                     ## Prediction at population level
                                     exclude = 's(unique.ID)')

## Calculate the contrasts

## First, extract the dimension of the matrix containing only "within SIT"
n4 <- d.pred.gamm_aug.Ascona.25.SIT.outside %>%
  filter(zona.SIT.discrete.fac == "within SIT") %>%
  nrow() %>%
  print()
```

```
[1] 82
```

```
##
## check that "outside SIT" has the same dimension
d.pred.gamm_aug.Ascona.25.SIT.outside %>%
  filter(zona.SIT.discrete.fac == "outside SIT") %>%
  nrow()
```

```
[1] 82
```

```
## makes sense

## Calculate the estimates of the contrasts
L.outside.Ascona  <- W.predictions.outside.sit[(n4 + 1):(2*n4), , drop = FALSE] -
```

```
  W.predictions.outside.sit[1:n4, , drop = FALSE]
est.outside.Ascona <- as.vector(L.outside.Ascona %*% c.coeff.gamm.eggs.Ascona)

## Now, we calculate the corresponding standard error
se.outside.Ascona  <-
  ( (L.outside.Ascona %*% vcov.c.coeff.gamm.eggs.Ascona) * L.outside.Ascona ) %>%
  rowSums() %>%
  sqrt()

d.diff.outside.Ascona <- data.frame(
  yday = d.pred.gamm_aug.Ascona$yday[1:n4],
  diff = est.outside.Ascona,
  lo = est.outside.Ascona - qnorm(0.975) * se.outside.Ascona,
  hi = est.outside.Ascona + qnorm(0.975) * se.outside.Ascona
) %>%
  mutate(exp.diff = exp(diff),
         exp.lo = exp(lo),
         exp.hi = exp(hi))
```

We plot the results.

```
ggplot(d.diff.outside.Ascona, mapping = aes(x = yday, y = diff)) +
  geom_ribbon(aes(ymin = lo, ymax = hi), fill = "lightgray", alpha = 0.5) +
  geom_line(linewidth = 1) +
  geom_hline(yintercept = 0, linetype = "dashed") +
  geom_vline(xintercept = yday("2025-06-17"), linetype = "dashed", colour = "grey") +
  labs(title = "Ascona",
       y = "outside SIT minus treated zone")
```

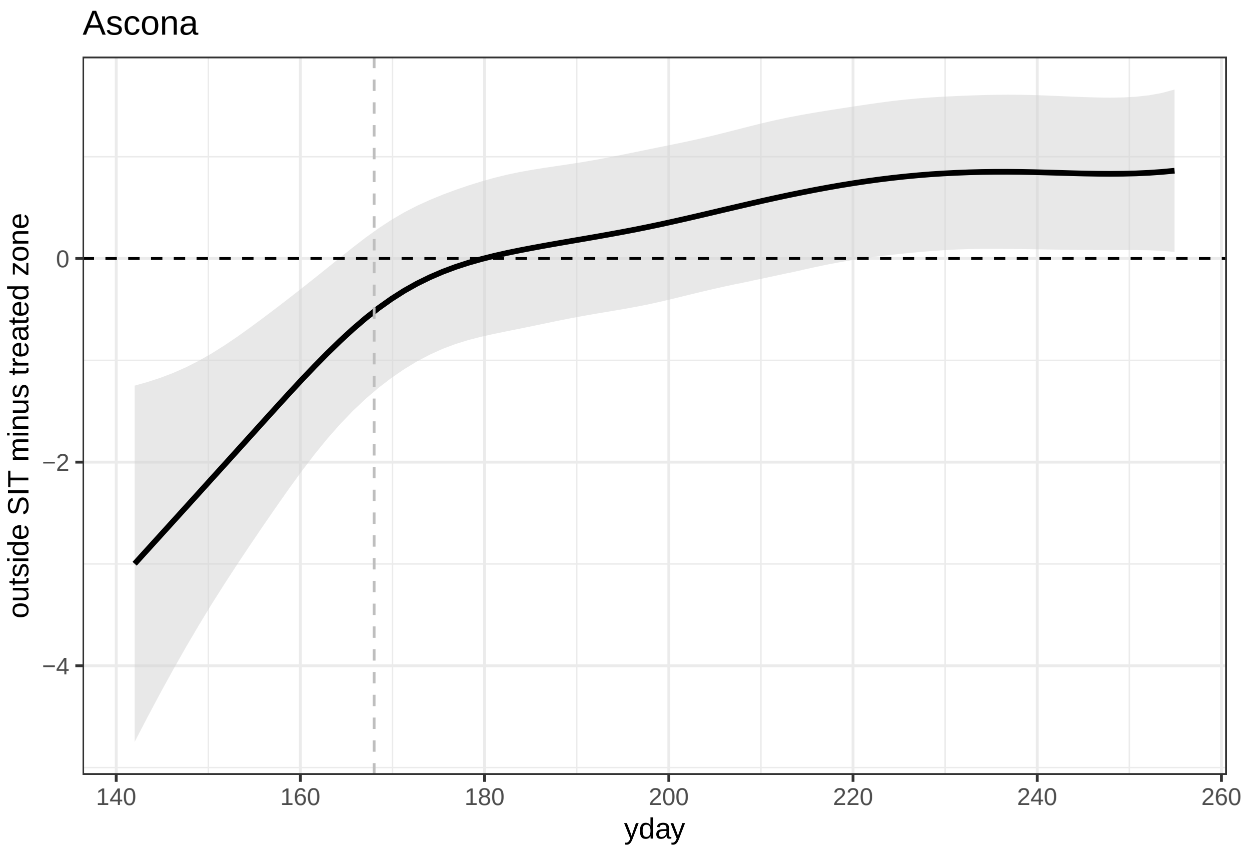

The plot shows the difference between the predicted values on the linear predictor scale for the untreated and treated zones, along with a 95% confidence interval. A horizontal dashed line at 0 indicates no difference between the two zones, while a vertical dashed line marks the start of the treatment on June 17, 2025.

We back-transform the difference by applying the exponential function to return it to the original scale. After this transformation, the values can be interpreted as ratios of predicted egg counts: a value of 1 indicates no difference between zones, values greater than 1 indicate higher predicted egg counts in the untreated zone relative to the treated zone, and values below 1 indicate the opposite.

```
gg.outside.Ascona <- ggplot(d.diff.outside.Ascona, mapping = aes(x = yday, y = exp.diff)) +
  geom_ribbon(aes(ymin = exp.lo, ymax = exp.hi), fill = "lightgray", alpha = 0.5) +
  geom_line(linewidth = 1) +
  geom_hline(yintercept = 1, linetype = "dashed") +
  geom_vline(xintercept = yday("2025-06-17"), linetype = "dashed", colour = "grey") +
  labs(title = "Ascona",
       y = "outside SIT minus treated zone \nin the back transformed scale")
gg.outside.Ascona
```

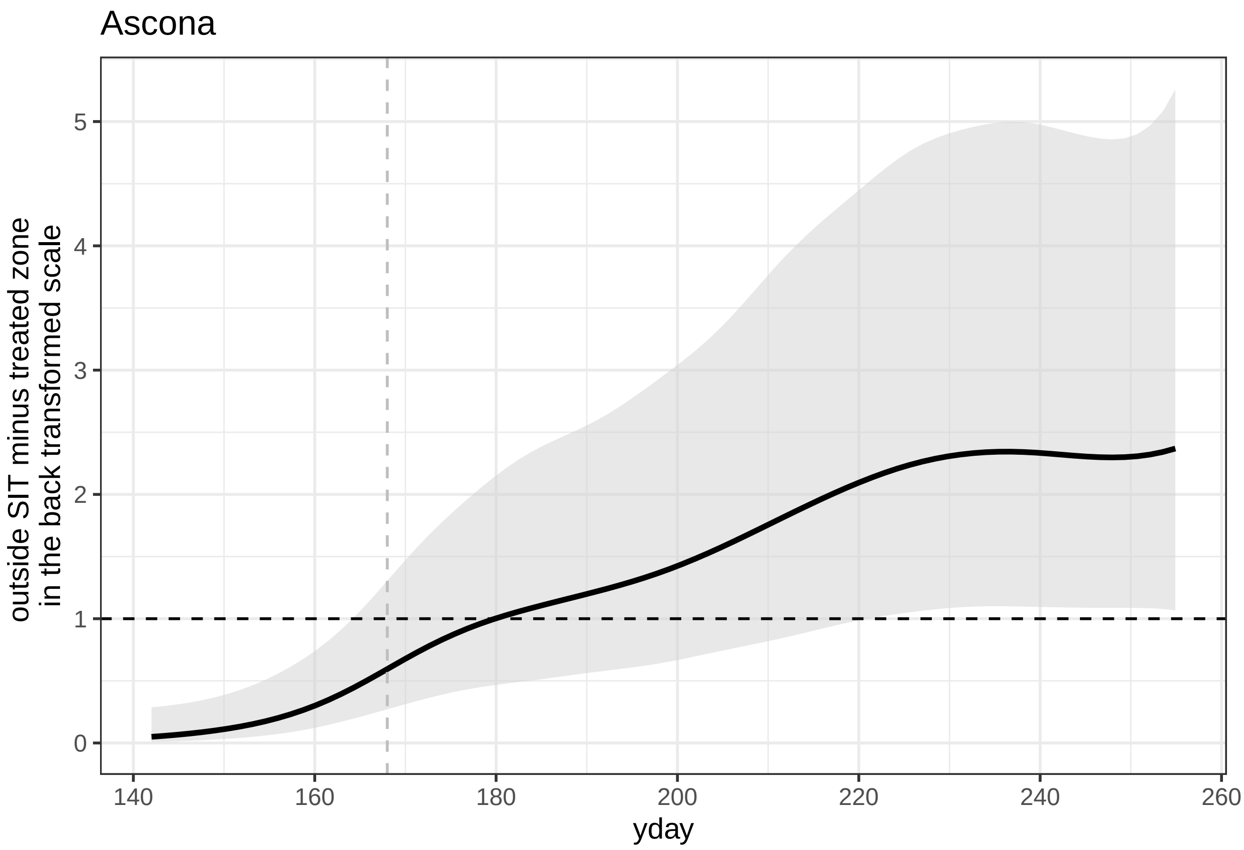

We zoom in to focus on the most relevant part of the graph. We use the same zoom as for the previous plots to better compare them.

```
## we zoom in
gg.outside.Ascona +
  coord_cartesian(ylim = c(0, 5))
```

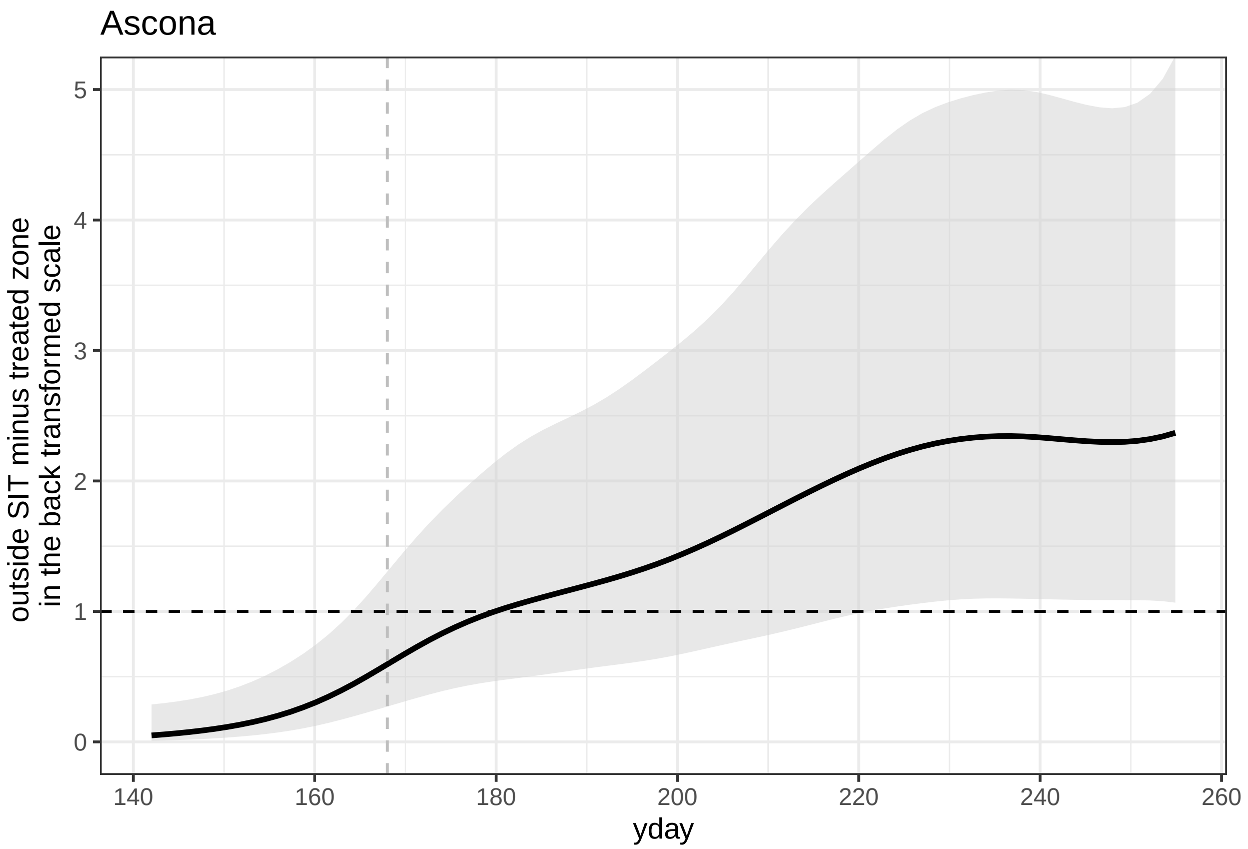

The treated zone initially shows higher predicted egg counts than the untreated zone. However, this difference quickly reverses, and after the treatment begins, the untreated zone consistently exhibits higher predicted egg counts than the treated zone; this difference increases constantly over time.

Note that the difference between the two zones is statistically significant when the grey zone doesn't intersect with the horizontal dashed line at 1.

An important point to note is that the plots generally show higher predicted egg counts in the treated zones compared to the other zones when no treatment is applied. This suggests that the control zones were not perfectly selected. For a proper comparison, the zones should be similar under the same conditions; that is, they should start with no difference (or a ratio of 1 on the back-transformed scale). However, once the treatment begins, the treated zones consistently show lower predicted egg counts than the other zones, indicating that the treatment had a positive effect.

5.7 Checking the offset

5.7.1 Checking the offset for Losone

The offset makes the assumption that number of eggs is proportional to effort (i.e. "activation time"). Let's formally check this assumption by re-fitting the model without the offset.

If this assumption holds true, one can also show graphs with e.g. the "daily number of eggs".

```
gamm.eggs.Losone_effortSmooth <- gam(total.albopictus.egg ~
                                       s(unique.ID, bs = "re") +
                                       s(log(Activation.time), k = 6) +
```

```r
                              zona.SIT.discrete.fac +
                              Year.fac +

                              s(yday, by = zona.SIT.discrete.fac),

                            family = "nb",
                            method = "GCV.Cp",
                            data = d.ovitraps.temporal.Losone)
```

```r
summary(gamm.eggs.Losone_effortSmooth)
```

```
Family: Negative Binomial(0.577)
Link function: log

Formula:
total.albopictus.egg ~ s(unique.ID, bs = "re") + s(log(Activation.time),
    k = 6) + zona.SIT.discrete.fac + Year.fac + s(yday, by = zona.SIT.discrete.fac)

Parametric coefficients:
                                        Estimate      Std. Error  z value
(Intercept)                        3.2388976406064 0.2813335384497 11.51266
zona.SIT.discrete.facbuffer        0.6207166050365 0.2630624208021  2.35958
zona.SIT.discrete.facoutside SIT 0.1842049934883 0.3088855780013  0.59635
Year.fac2025                       0.0853838793921 0.1767761014503  0.48301
                                             Pr(>|z|)
(Intercept)                        < 0.0000000000000002 ***
zona.SIT.discrete.facbuffer                   0.018296 *
zona.SIT.discrete.facoutside SIT              0.550939
Year.fac2025                                  0.629092
---
Signif. codes:  0 '***' 0.001 '**' 0.01 '*' 0.05 '.' 0.1 ' ' 1

Approximate significance of smooth terms:
                                                    edf         Ref.df
s(unique.ID)                              26.26883665774 47.00000000000
s(log(Activation.time))                    1.83677857146  2.22055390495
s(yday):zona.SIT.discrete.facwithin SIT    4.72802401499  5.79914787780
s(yday):zona.SIT.discrete.facbuffer        4.87593868974  5.95029522191
s(yday):zona.SIT.discrete.facoutside SIT  5.77444023189  6.72263535844
                                             Chi.sq               p-value
s(unique.ID)                              60.97225          0.0000016131 ***
s(log(Activation.time))                    8.33224              0.026131 *
s(yday):zona.SIT.discrete.facwithin SIT   32.90731          0.0000120544 ***
s(yday):zona.SIT.discrete.facbuffer       74.36059 < 0.0000000000000000222 ***
s(yday):zona.SIT.discrete.facoutside SIT 282.56495 < 0.0000000000000000222 ***
---
Signif. codes:  0 '***' 0.001 '**' 0.01 '*' 0.05 '.' 0.1 ' ' 1

R-sq.(adj) =  0.364   Deviance explained = 47.5%
-REML = 2542.3  Scale est. = 1         n = 552
```

The estimated degree of freedom for the effect of effort (i.e., *Activation.time*) is estimated to be 1.837, i.e., not exactly linear. Let's visualise it.

```r
plot.gam(gamm.eggs.Losone_effortSmooth, select = 2,
         ylim = c(-1, 1))
```

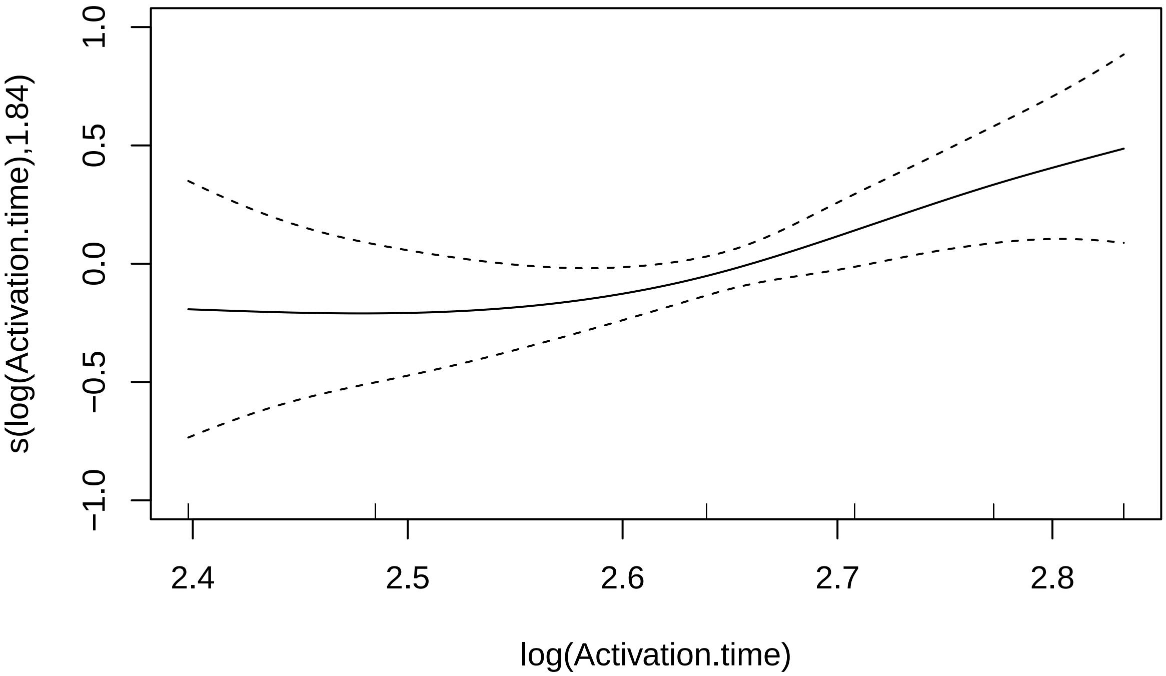

A flat line could pass through the confidence band, indicating that a linear effect might be sufficient. Therefore, we can remove *Activation.time* from the smooth terms. Note that we take it's log simply because the link function implicitly used here is the log. So, the results will be interpretable in the %-% scale.

```r
gamm.eggs.Losone_effort.lin <- gam(total.albopictus.egg ~
                                      log(Activation.time) +
                                      s(unique.ID, bs = "re") +

                                      zona.SIT.discrete.fac +
                                      Year.fac +

                                      s(yday, by = zona.SIT.discrete.fac),

                                    family = "nb",
                                    method = "GCV.Cp",
                                    data = d.ovitraps.temporal.Losone)
summary(gamm.eggs.Losone_effort.lin)
```

```
Family: Negative Binomial(0.574)
Link function: log

Formula:
total.albopictus.egg ~ log(Activation.time) + s(unique.ID, bs = "re") +
    zona.SIT.discrete.fac + Year.fac + s(yday, by = zona.SIT.discrete.fac)

Parametric coefficients:
                                        Estimate      Std. Error  z value
(Intercept)                        -1.494125128172  1.913924631001 -0.78066
log(Activation.time)                1.760586599108  0.715558470321  2.46044
```

```
zona.SIT.discrete.facbuffer       0.620073846963  0.262520490374  2.36200
zona.SIT.discrete.facoutside SIT  0.294403185725  0.297004029613  0.99124
Year.fac2025                      0.138984969819  0.173228642885  0.80232
                                 Pr(>|z|)
(Intercept)                      0.435002
log(Activation.time)             0.013877 *
zona.SIT.discrete.facbuffer      0.018177 *
zona.SIT.discrete.facoutside SIT 0.321567
Year.fac2025                     0.422367
---
Signif. codes:  0 '***' 0.001 '**' 0.01 '*' 0.05 '.' 0.1 ' ' 1

Approximate significance of smooth terms:
                                                edf         Ref.df
s(unique.ID)                             26.04685367097 47.00000000000
s(yday):zona.SIT.discrete.facwithin SIT   4.72040295264  5.79486258596
s(yday):zona.SIT.discrete.facbuffer       4.93805648587  6.02619166719
s(yday):zona.SIT.discrete.facoutside SIT  5.77870774070  6.73485692312
                                          Chi.sq                p-value
s(unique.ID)                              59.92019           0.0000021109 ***
s(yday):zona.SIT.discrete.facwithin SIT   32.13085           0.0000175039 ***
s(yday):zona.SIT.discrete.facbuffer       74.86710 < 0.0000000000000000222 ***
s(yday):zona.SIT.discrete.facoutside SIT 314.25744 < 0.0000000000000000222 ***
---
Signif. codes:  0 '***' 0.001 '**' 0.01 '*' 0.05 '.' 0.1 ' ' 1

R-sq.(adj) =  0.392   Deviance explained = 47.2%
-REML = 2540.1  Scale est. = 1         n = 552
```

We calculate the confidence interval for the coefficient of log(*Activation.time*).

```
( gamm.eggs.Losone_effort.lin$coefficients["log(Activation.time)"] +
    c(-1, 1) * 2 *summary(gamm.eggs.Losone_effort.lin)$se["log(Activation.time)"] )
```

```
[1] 0.329469658465264 3.191703539750079
```

The value 1 is contained in the confidence interval for the log of *Activation.time*; this means that assuming proportionality is consistent with the data. Note that this is true regardless of the p-value of the summary above being significant. Indeed, here we are not interested in testing whether the variable has no effect (what the p-value refers to), but rather whether the coefficient could be 1 (which is the case based on the CI).

5.7.2 Checking the offset for Ascona

We perform the same check for Ascona.

```
gamm.eggs.Ascona_effortSmooth <- gam(total.albopictus.egg ~
                                       s(unique.ID, bs = "re") +
                                       s(log(Activation.time), k = 6) +


                                       zona.SIT.discrete.fac +
                                       Year.fac +

                                       s(yday, by = zona.SIT.discrete.fac),

                                     family = "nb",
```

```r
                                method = "GCV.Cp",
                                data = d.ovitraps.temporal.Ascona)
```

```r
summary(gamm.eggs.Ascona_effortSmooth)
```

```
Family: Negative Binomial(0.817)
Link function: log

Formula:
total.albopictus.egg ~ s(unique.ID, bs = "re") + s(log(Activation.time),
    k = 6) + zona.SIT.discrete.fac + Year.fac + s(yday, by = zona.SIT.discrete.fac)

Parametric coefficients:
                                        Estimate      Std. Error  z value
(Intercept)                         3.5409606635778 0.3015141608627 11.74393
zona.SIT.discrete.facbuffer         0.1263517766819 0.3216975387211  0.39277
zona.SIT.discrete.facoutside SIT 0.0343793653145 0.3256265789073  0.10558
Year.fac2025                        0.3987164310824 0.1510935064141  2.63887
                                              Pr(>|z|)
(Intercept)                         < 0.000000000000000222 ***
zona.SIT.discrete.facbuffer                      0.6944925
zona.SIT.discrete.facoutside SIT                 0.9159163
Year.fac2025                                     0.0083182 **
---
Signif. codes:  0 '***' 0.001 '**' 0.01 '*' 0.05 '.' 0.1 ' ' 1

Approximate significance of smooth terms:
                                            edf          Ref.df
s(unique.ID)                           43.12124586011 51.00000000000
s(log(Activation.time))                 2.63449179650  3.14945596707
s(yday):zona.SIT.discrete.facwithin SIT   4.22238267298  5.21909164242
s(yday):zona.SIT.discrete.facbuffer       7.00395668454  8.02439789598
s(yday):zona.SIT.discrete.facoutside SIT  5.43792049359  6.37084039835
                                          Chi.sq                 p-value
s(unique.ID)                           348.06646 < 0.000000000000000222 ***
s(log(Activation.time))                 12.54534              0.0068682 **
s(yday):zona.SIT.discrete.facwithin SIT   49.10007 < 0.000000000000000222 ***
s(yday):zona.SIT.discrete.facbuffer      106.93675 < 0.000000000000000222 ***
s(yday):zona.SIT.discrete.facoutside SIT 550.48550 < 0.000000000000000222 ***
---
Signif. codes:  0 '***' 0.001 '**' 0.01 '*' 0.05 '.' 0.1 ' ' 1

R-sq.(adj) =  -3.76   Deviance explained =   54%
-REML = 3184.5  Scale est. = 1         n = 628
```

Here, the estimated degree of freedom for the effect of effort (i.e., *Activation.time*) is 2.634, i.e., not linear. Let's visualise it.

```r
plot.gam(gamm.eggs.Ascona_effortSmooth, select = 2,
         ylim = c(-1, 1))
```

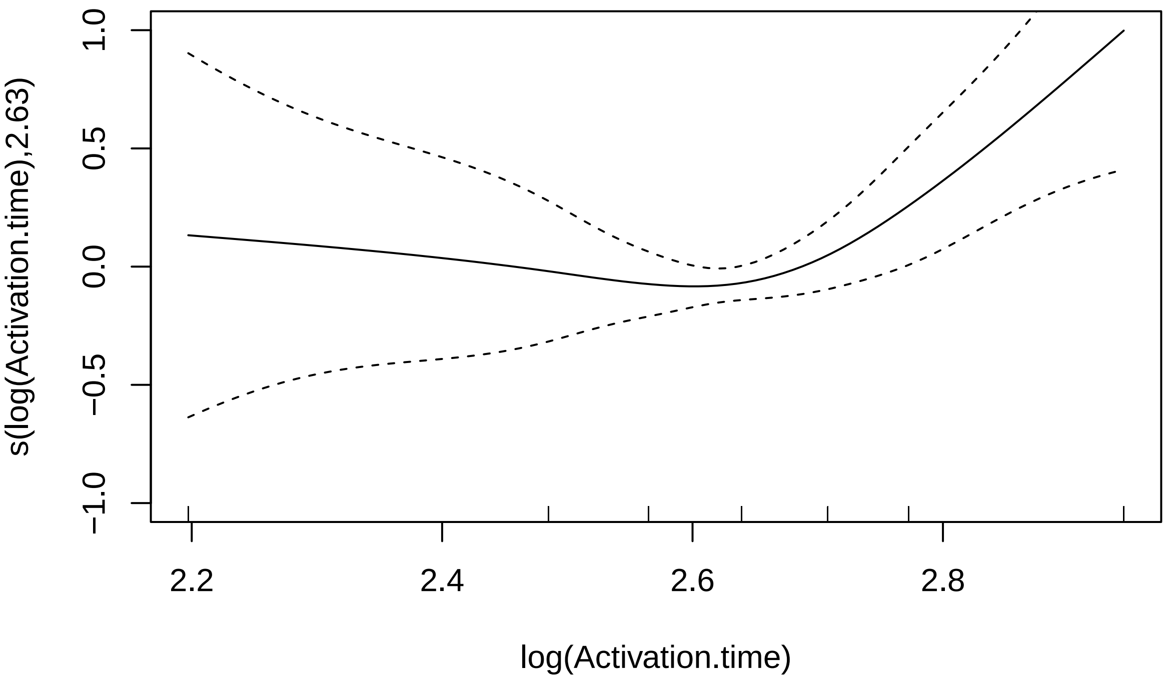

The estimated degrees of freedom is not 1, but, looking at the smoother, a straight line could pass through the confidence band, indicating that a linear effect might be sufficient. Therefore, we can remove *Activation.time* from the smooth terms.

```
gamm.eggs.Ascona_effort.lin <- gam(total.albopictus.egg ~
                                     log(Activation.time) +
                                     s(unique.ID, bs = "re") +

                                     zona.SIT.discrete.fac +
                                     Year.fac +

                                     s(yday, by = zona.SIT.discrete.fac),

                                   family = "nb",
                                   method = "GCV.Cp",
                                   data = d.ovitraps.temporal.Ascona)

summary(gamm.eggs.Ascona_effort.lin)
```

```
Family: Negative Binomial(0.808)
Link function: log

Formula:
total.albopictus.egg ~ log(Activation.time) + s(unique.ID, bs = "re") +
    zona.SIT.discrete.fac + Year.fac + s(yday, by = zona.SIT.discrete.fac)

Parametric coefficients:
                                       Estimate     Std. Error z value Pr(>|z|)
(Intercept)                         0.173620507902 1.344840012428 0.12910 0.897278
log(Activation.time)                1.324163917585 0.494173064460 2.67956 0.007372
zona.SIT.discrete.facbuffer         0.137379130723 0.321094393497 0.42785 0.668763
zona.SIT.discrete.facoutside SIT 0.124834719067 0.322322996581 0.38730 0.698536
```

```
Year.fac2025                     0.218606697738 0.130603538200 1.67382 0.094166

(Intercept)
log(Activation.time)             **
zona.SIT.discrete.facbuffer
zona.SIT.discrete.facoutside SIT
Year.fac2025                     .
---
Signif. codes:  0 '***' 0.001 '**' 0.01 '*' 0.05 '.' 0.1 ' ' 1

Approximate significance of smooth terms:
                                               edf         Ref.df
s(unique.ID)                         43.01152158627 51.00000000000
s(yday):zona.SIT.discrete.facwithin SIT   4.16869075009  5.15961419842
s(yday):zona.SIT.discrete.facbuffer       6.98671795989  8.01856277795
s(yday):zona.SIT.discrete.facoutside SIT  5.39330129840  6.36449152262
                                            Chi.sq                p-value
s(unique.ID)                         341.06896 < 0.000000000000000222 ***
s(yday):zona.SIT.discrete.facwithin SIT   47.82091 < 0.000000000000000222 ***
s(yday):zona.SIT.discrete.facbuffer      106.54156 < 0.000000000000000222 ***
s(yday):zona.SIT.discrete.facoutside SIT 635.47363 < 0.000000000000000222 ***
---
Signif. codes:  0 '***' 0.001 '**' 0.01 '*' 0.05 '.' 0.1 ' ' 1

R-sq.(adj) =  -3.52   Deviance explained = 53.4%
-REML = 3183.8  Scale est. = 1         n = 628
```

We calculate the confidence interval for the coefficient of log(*Activation.time*).

```
( gamm.eggs.Ascona_effort.lin$coefficients["log(Activation.time)"] +
    c(-1, 1) * 2 *summary(gamm.eggs.Ascona_effort.lin)$se["log(Activation.time)"] )
```

```
[1] 0.335817788666178 2.312510046504213
```

The value 1 is contained in the confidence interval for the log of *Activation.time*; this means that assuming proportionality is consistent with the data.

5.8 Residual analysis

First of all, we apply the `gam.check()` function to the model, which produces some diagnostic information.

```
par(mfrow = c(2, 2))
gam.check(gamm.eggs.Losone)
```

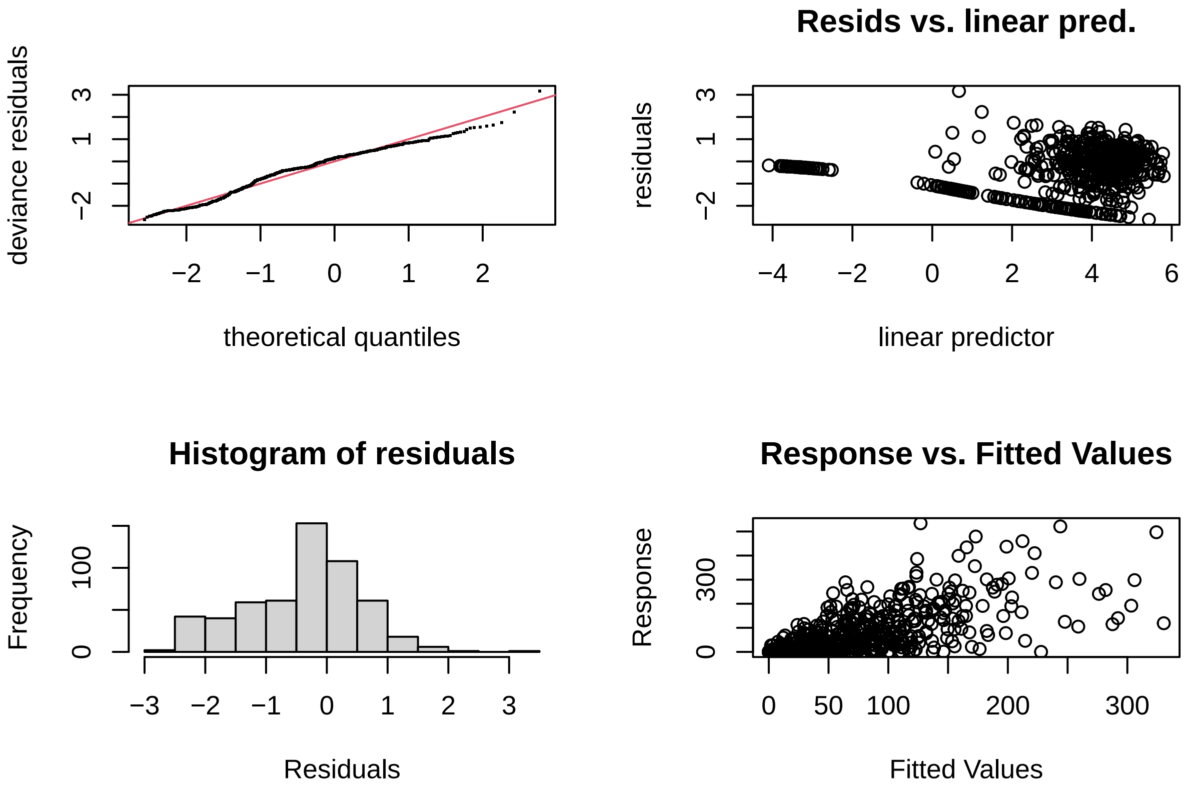


```
Method: REML   Optimizer: outer newton
full convergence after 4 iterations.
Gradient range [-0.0000000261602370876801,0.0000887509690752353]
(score 2541.22545401228 & scale 1).
Hessian positive definite, eigenvalue range [0.619480298501473,210.656763376283].
Model rank =  79 / 79

Basis dimension (k) checking results. Low p-value (k-index<1) may
indicate that k is too low, especially if edf is close to k'.

                                         k'   edf k-index
s(unique.ID)                          48.00 26.03      NA
s(yday):zona.SIT.discrete.facwithin SIT  9.00  4.77    0.75
s(yday):zona.SIT.discrete.facbuffer      9.00  4.98    0.75
s(yday):zona.SIT.discrete.facoutside SIT  9.00  5.82    0.75
                                                 p-value
s(unique.ID)                                          NA
s(yday):zona.SIT.discrete.facwithin SIT  <0.0000000000000002 ***
s(yday):zona.SIT.discrete.facbuffer      <0.0000000000000002 ***
s(yday):zona.SIT.discrete.facoutside SIT <0.0000000000000002 ***
---
Signif. codes:  0 '***' 0.001 '**' 0.01 '*' 0.05 '.' 0.1 ' ' 1
```

```r
par(mfrow = c(1, 1))

par(mfrow = c(2, 2))
gam.check(gamm.eggs.Ascona)
```

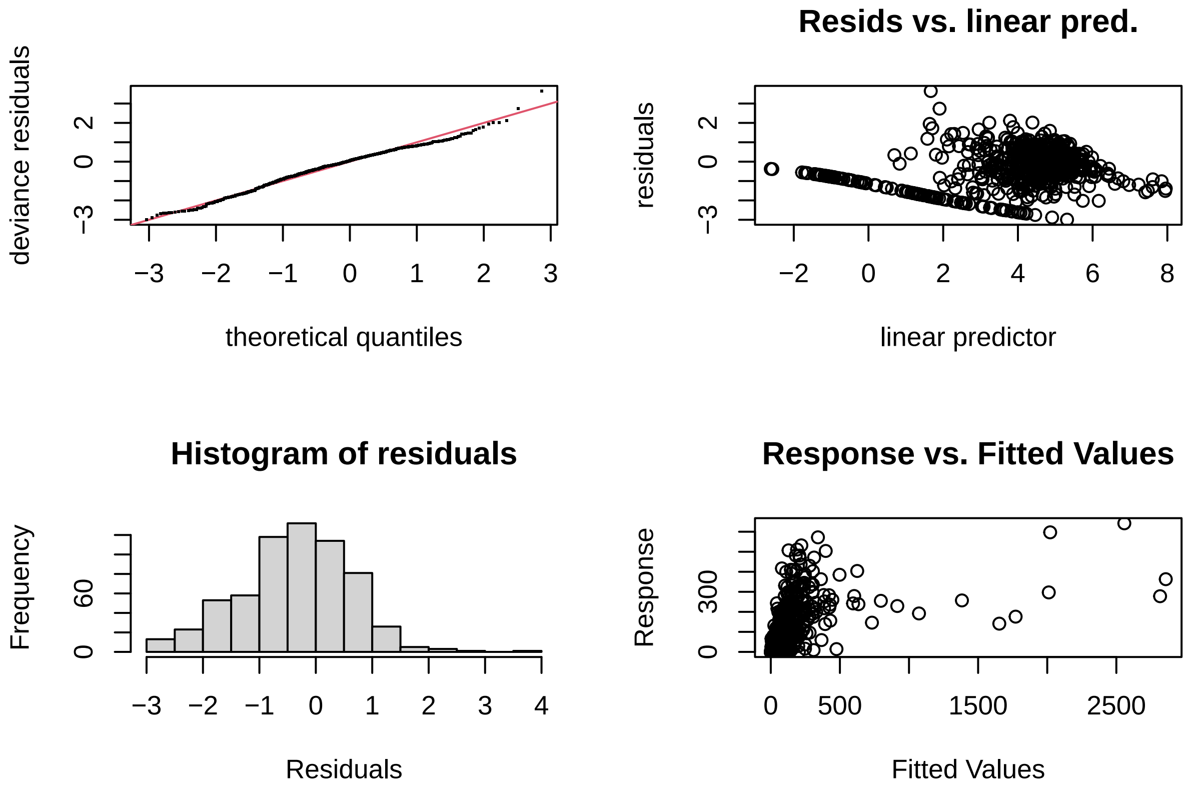


```
Method: REML   Optimizer: outer newton
full convergence after 6 iterations.
Gradient range [-0.0000741911184505284,0.000722883951429054]
(score 3184.18094590396 & scale 1).
Hessian positive definite, eigenvalue range [0.927877292784042,240.02871972052].
Model rank =  83 / 83

Basis dimension (k) checking results. Low p-value (k-index<1) may
indicate that k is too low, especially if edf is close to k'.

                                          k'   edf k-index p-value
s(unique.ID)                           52.00 43.01      NA      NA
s(yday):zona.SIT.discrete.facwithin SIT  9.00  4.18    0.85    0.17
s(yday):zona.SIT.discrete.facbuffer      9.00  7.00    0.85    0.19
s(yday):zona.SIT.discrete.facoutside SIT  9.00  5.60    0.85    0.23
```

```r
par(mfrow = c(1, 1))
```

Then, we store the pearson residuals in the original data frame, and we plot the residuals against the fitted values to see whether there is still structure in the data.

```r
d.ovitraps.temporal.Losone$resid_gamm.tot.eggs <- resid(gamm.eggs.Losone,
                                                        type = "pearson")
##
ggplot(data = d.ovitraps.temporal.Losone,
       mapping = aes(y = resid_gamm.tot.eggs,
                     x = fitted_gamm.eggs)) +
  geom_hline(yintercept = 0) +
  geom_point(alpha = 0.2) +
```

```r
  geom_smooth()
```

```
`geom_smooth()` using method = 'loess' and formula = 'y ~ x'
```

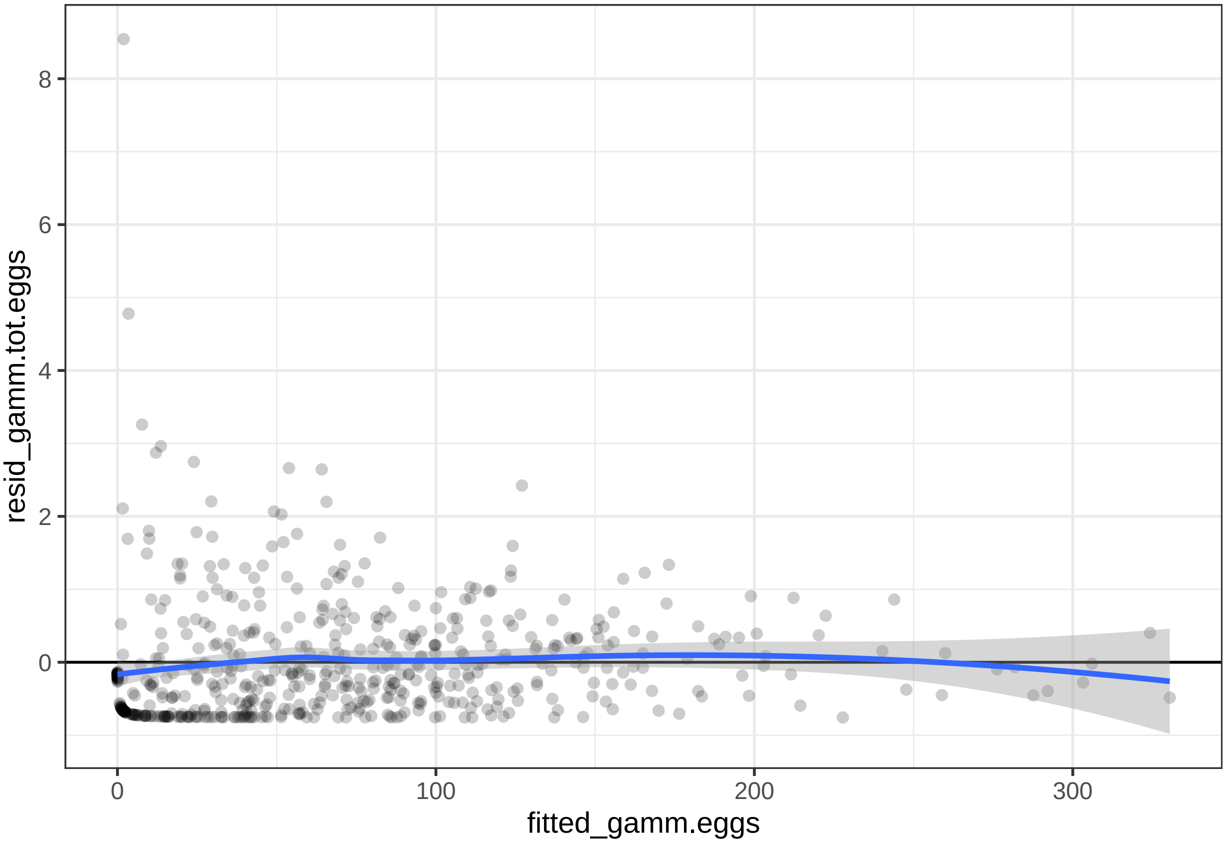

The blue line is located on the x-axis and it is quite straight, indicating that there doesn't seem to be much structure left in the residuals.

```r
d.ovitraps.temporal.Ascona$resid_gamm.tot.eggs <- resid(gamm.eggs.Ascona,
                                                        type = "pearson")
##
ggplot(data = d.ovitraps.temporal.Ascona,
       mapping = aes(y = resid_gamm.tot.eggs,
                     x = fitted_gamm.eggs)) +
  geom_hline(yintercept = 0) +
  geom_point(alpha = 0.2) +
  geom_smooth()
```

```
`geom_smooth()` using method = 'loess' and formula = 'y ~ x'
```

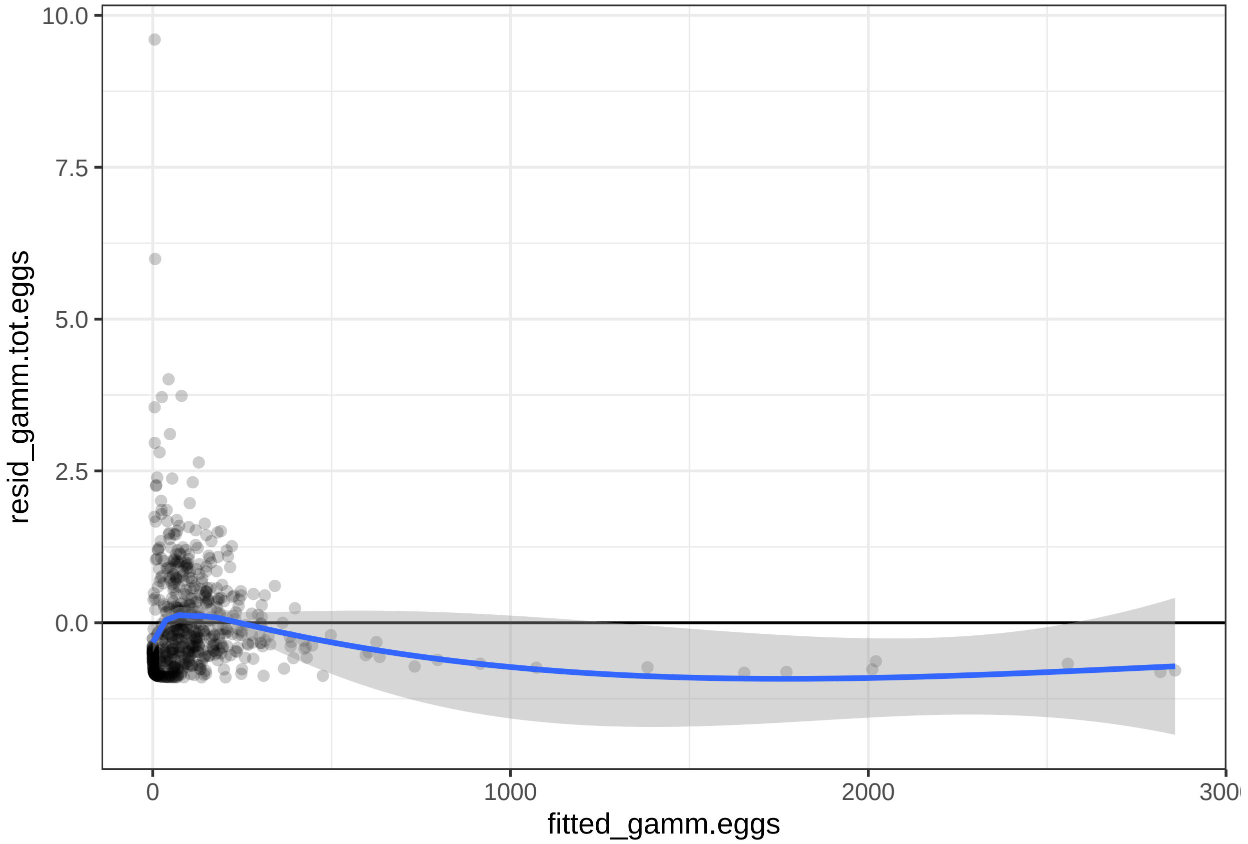

The blue line is not located far from the x-axis and it is quite straight, indicating that there doesn't seem to be much structure left in the residuals.

We plot the qq-plot for the random effects.

```
## QQ for random effects
plot(sm(gamm.eggs.Losone, 1))
```

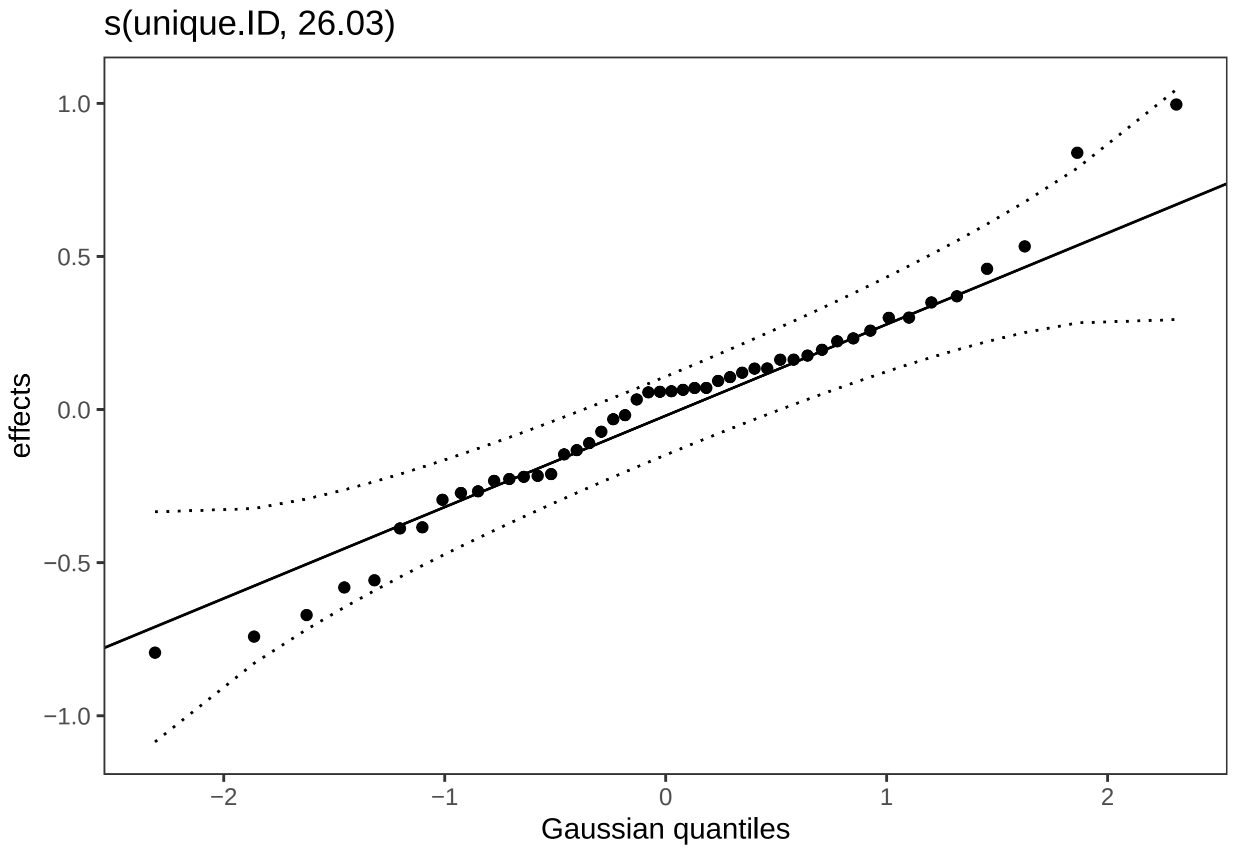


```
##
plot(sm(gamm.eggs.Ascona, 1))
```

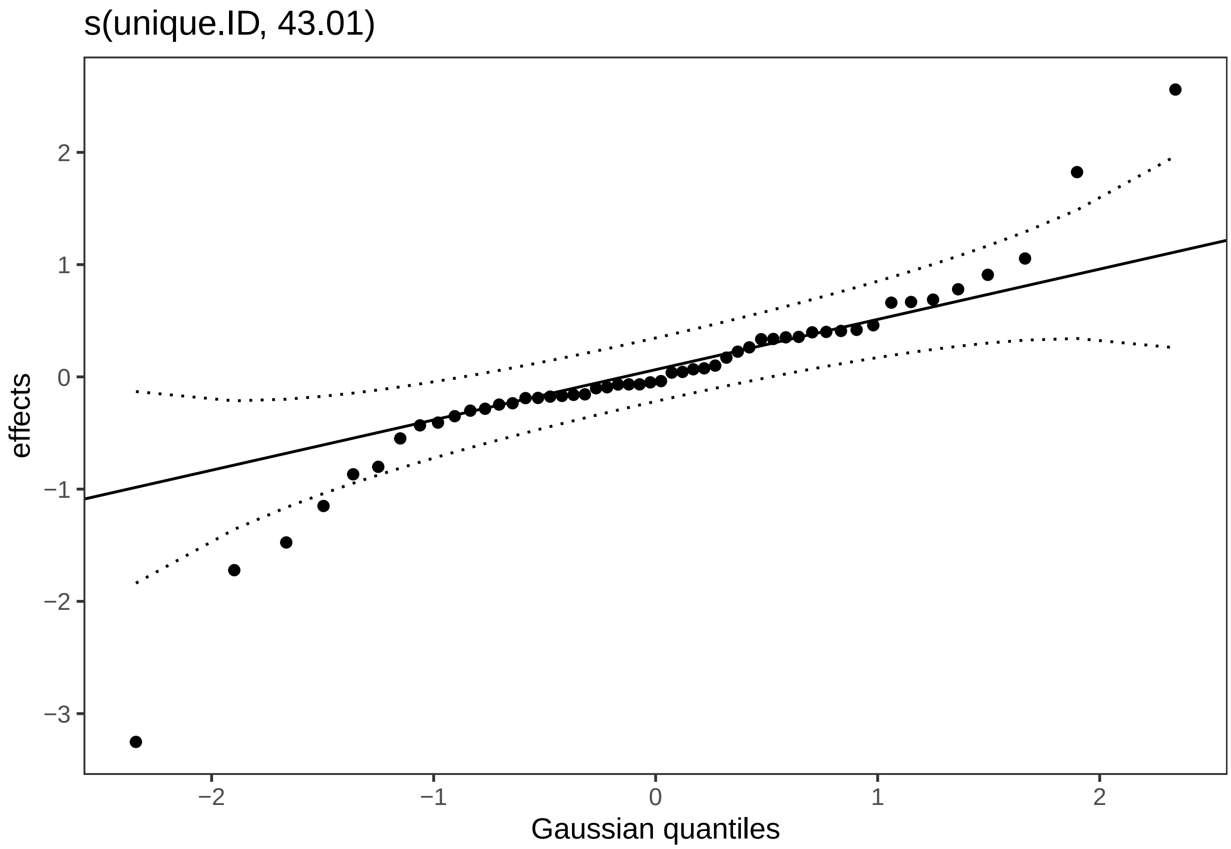

There doesn't seem to be a significant departure from the normality assumption for either models.

We now plot the residuals over time to further check whether there is any structure left in the data.

```
ggplot(data = d.ovitraps.temporal.Losone,
       mapping = aes(y = resid_gamm.tot.eggs,
                     x = yday)) +
  geom_hline(yintercept = 0) +
  geom_point(alpha = 0.2) +
  geom_smooth(method = "loess")
```

```
`geom_smooth()` using formula = 'y ~ x'
```

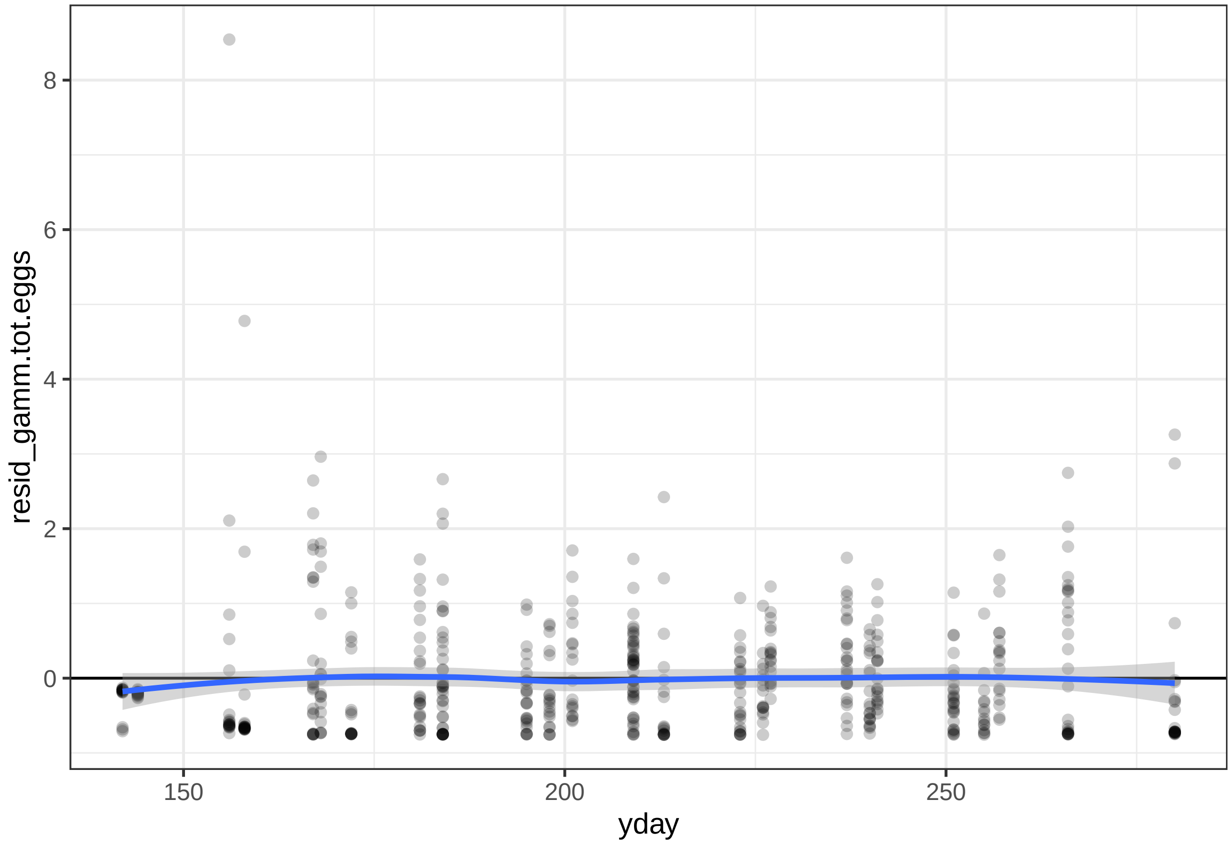


```
##
ggplot(data = d.ovitraps.temporal.Ascona,
       mapping = aes(y = resid_gamm.tot.eggs,
                     x = yday)) +
  geom_hline(yintercept = 0) +
  geom_point(alpha = 0.2) +
  geom_smooth(method = "loess")
```

```
`geom_smooth()` using formula = 'y ~ x'
```

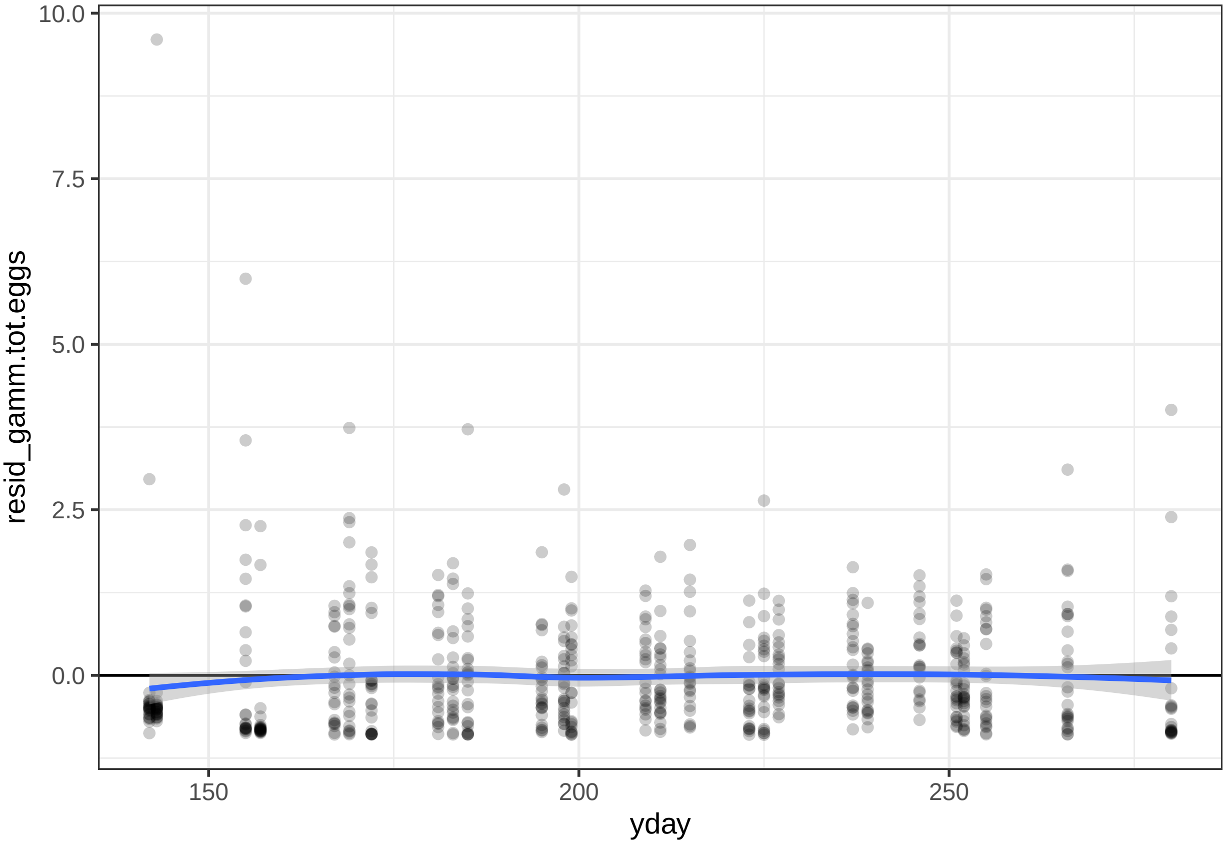

There does not seem to be any structure left in the data.

The following two plots display the residuals in Losone and Ascona, respectively.
The observations belonging to the same ovitrap are connected with a line. These plots are additionally drawn to check temporal correlation and variability.

```
## for Losone
ggplot(data = d.ovitraps.temporal.Losone,
       mapping = aes(y = resid_gamm.tot.eggs,
                     x = yday,
                     group = unique.ID)) +
  geom_hline(yintercept = 0) +
  geom_point(alpha = 0.2) +
  geom_line(alpha = 0.2) +
  labs(title = "Losone")
```

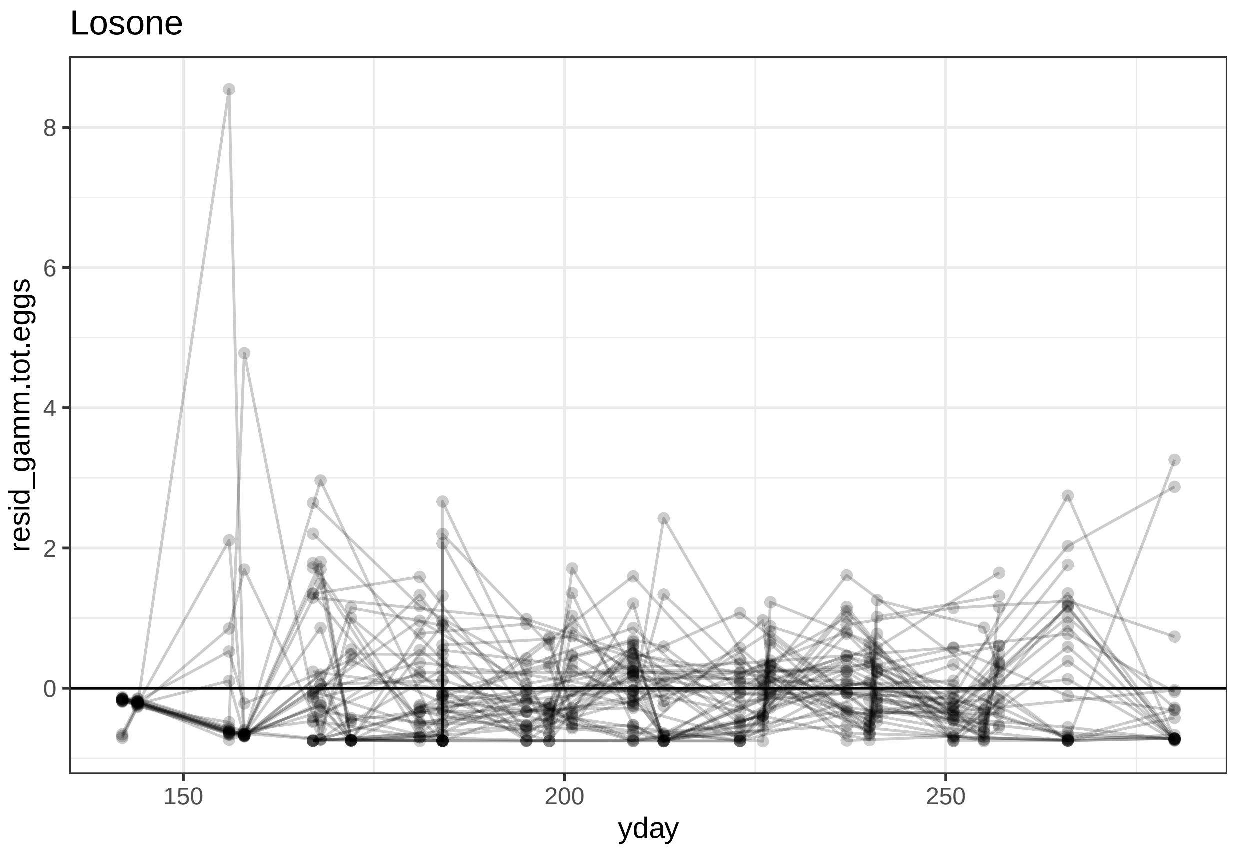


```
##
## For Ascona
ggplot(data = d.ovitraps.temporal.Ascona,
       mapping = aes(y = resid_gamm.tot.eggs,
                     x = yday,
                     group = unique.ID)) +
  geom_hline(yintercept = 0) +
  geom_point(alpha = 0.2) +
  geom_line(alpha = 0.2) +
  labs(title = "Ascona")
```

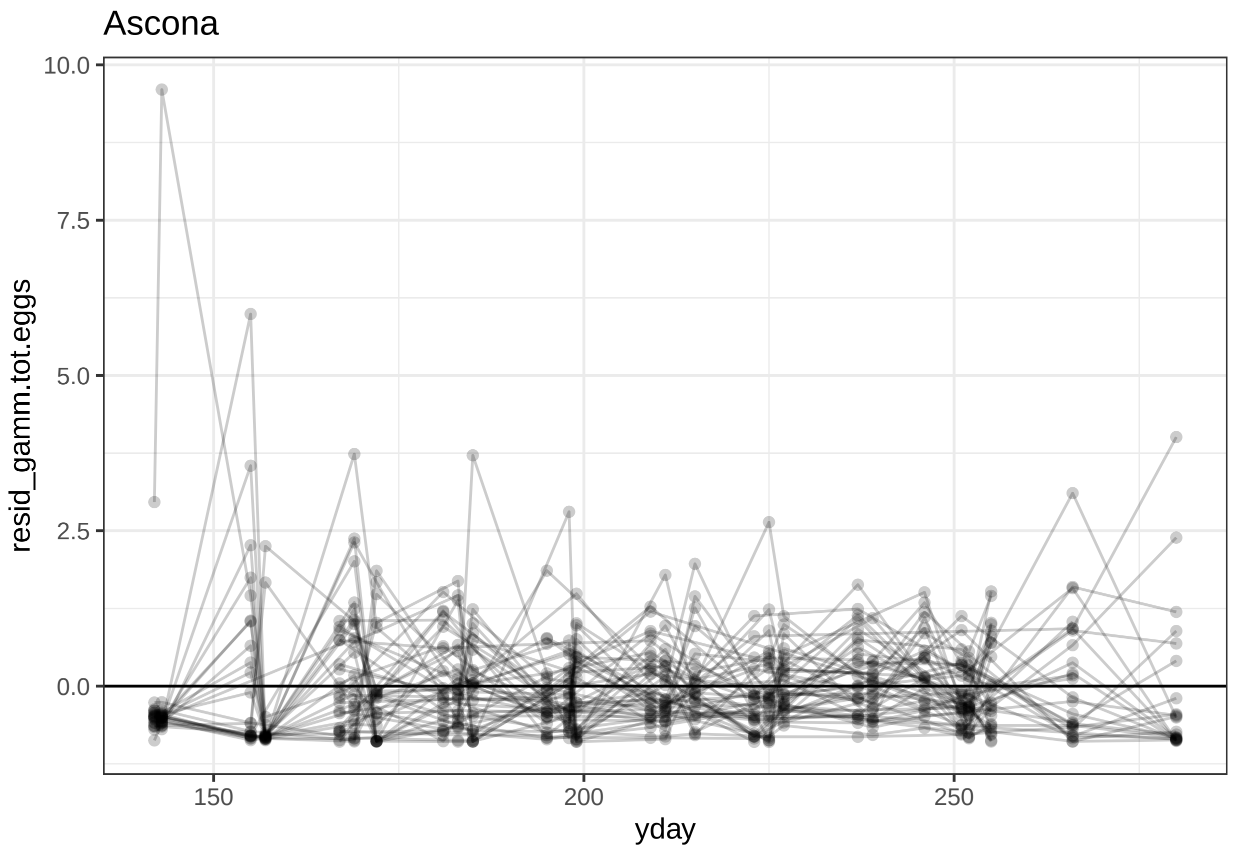

There doesn't seem to be any structure left in the data.

Let's look at the single ovitraps.

```
## for Losone
ggplot(data = d.ovitraps.temporal.Losone,
       mapping = aes(y = resid_gamm.tot.eggs,
                     x = yday,
                     group = unique.ID)) +
  geom_hline(yintercept = 0) +
  geom_point() +
  geom_line() +
  facet_wrap(~ unique.ID) +
  theme(
    strip.background = element_blank(),
    strip.text.x = element_blank()) +
  coord_cartesian(ylim = c(-1,4)) +
  labs(title = "Losone")
```

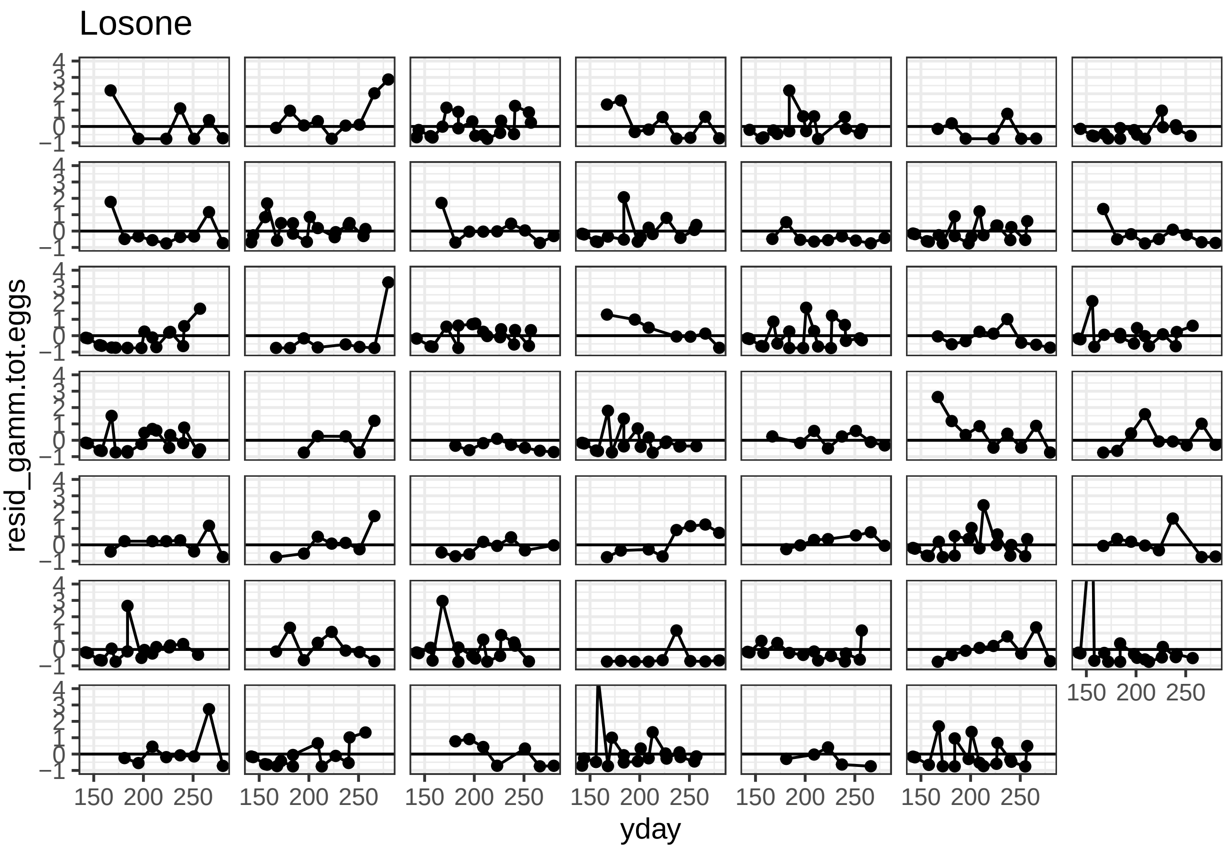


```r
##
## for Ascona
ggplot(data = filter(d.ovitraps.temporal.Ascona,
                     municipality.fac == "Ascona"),
       mapping = aes(y = resid_gamm.tot.eggs,
                     x = yday,
                     group = unique.ID)) +
  geom_hline(yintercept = 0) +
  geom_point() +
  geom_line() +
  facet_wrap(~ unique.ID) +
  theme(
    strip.background = element_blank(),
    strip.text.x = element_blank()) +
  coord_cartesian(ylim = c(-1, 4)) +
  labs(title = "Ascona")
```

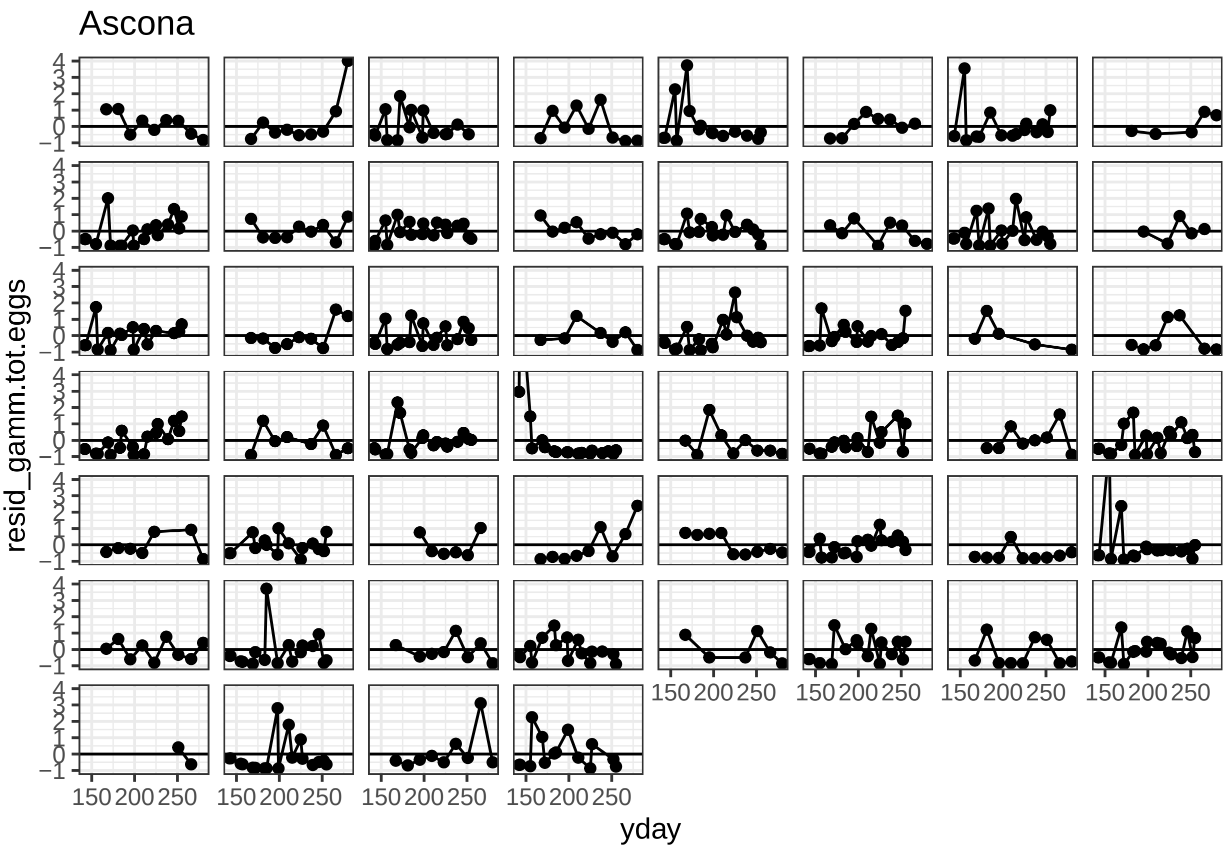

In both cases, there does not seem to be much structure left in the data.

Note that we set the limits at -1 and 4 to zoom in on the graphs, because there are a couple of ovitraps for which the residuals have some big jumps.

6 Spatial Generalised Additive Model (spatial GAM)

We are now interested in testing whether geographical location plays a role in determining the number of eggs laid.

6.1 Visualising the data

Let us first plot the geographical distribution of the median number of eggs in Ascona and Losone for the year 2025. We observe the median because we are dealing with skewed data.

For this (and subsequent graphs) we use the `d.ovitraps.spatial` data set, created during data preparation. This data set contains the median number of eggs for the municipalities of interest during the season. However, we will use `d.ovitraps.temporal` for modelling it.

We also mark the center of the treated zone with a cross.

```
d.coord.center.losone <- data.frame(X = 2702682.04, Y = 1113837.30) %>%
  st_as_sf(
    coords = c("X", "Y"),
    crs = 2056,
    remove = FALSE) %>%
  st_transform(crs = 21781) %>%
  st_coordinates() %>%
  as.data.frame()
d.coord.center.ascona <- data.frame(X = 2703480.39, Y = 1112071.09) %>%
  st_as_sf(
    coords = c("X", "Y"),
    crs = 2056,
    remove = FALSE) %>%
  st_transform(crs = 21781) %>%
  st_coordinates() %>%
  as.data.frame()
```

```
## (warnings are omitted from this chunk)
##
## Create two separate data sets, one for each municipality
d.ovitraps.spatial.Ascona.25 <- d.ovitraps.spatial %>%
  filter(municipality.fac.spatial == "Ascona" &
           ## There are traps in Ascona that are sampled bi-weekly
           ## (they came from a different data set).
           ## We filter those observations out.
           !is.na(X.num) &
           !is.na(Y.num) &
           Year.fac == 2025)
##
d.ovitraps.spatial.Losone.25 <- d.ovitraps.spatial %>%
  filter(municipality.fac.spatial %in% c("Losone") &
           Year.fac == 2025)
##
## We calculate the min and max for the median number of eggs.
## This is used in the plots, to maintain the same colour scale for the two
## municipalities and compare them more easily.
max.eggs.median.25 <- max(c(d.ovitraps.spatial.Ascona.25$median.eggs,
                            d.ovitraps.spatial.Losone.25$median.eggs))
```

```r
##
min.eggs.median.25 <- min(c(d.ovitraps.spatial.Ascona.25$median.eggs,
                            d.ovitraps.spatial.Losone.25$median.eggs))
##
## Plot Losone
gg.obs.L.25 <- ggplot(d.ovitraps.spatial.Losone.25,
                      mapping = aes(y = Y.num,
                                    x = X.num,
                                    colour = median.eggs)) +
  geom_point(size = 3) +
  geom_point(d.coord.center.losone, mapping = aes(x = X, y = Y),
             colour = "black", shape = 4, size = 4) +
  scale_color_gradientn(colours = c("blue", "gold"),
                        values = scales::rescale(c(min.eggs.median.25,
                                                   max.eggs.median.25)),
                        limits = c(min.eggs.median.25, max.eggs.median.25)) +
  # theme(aspect.ratio = 1) +
  coord_fixed() +
  labs(title = "Losone") +
  theme(legend.position = "bottom")
gg.obs.L.25
```

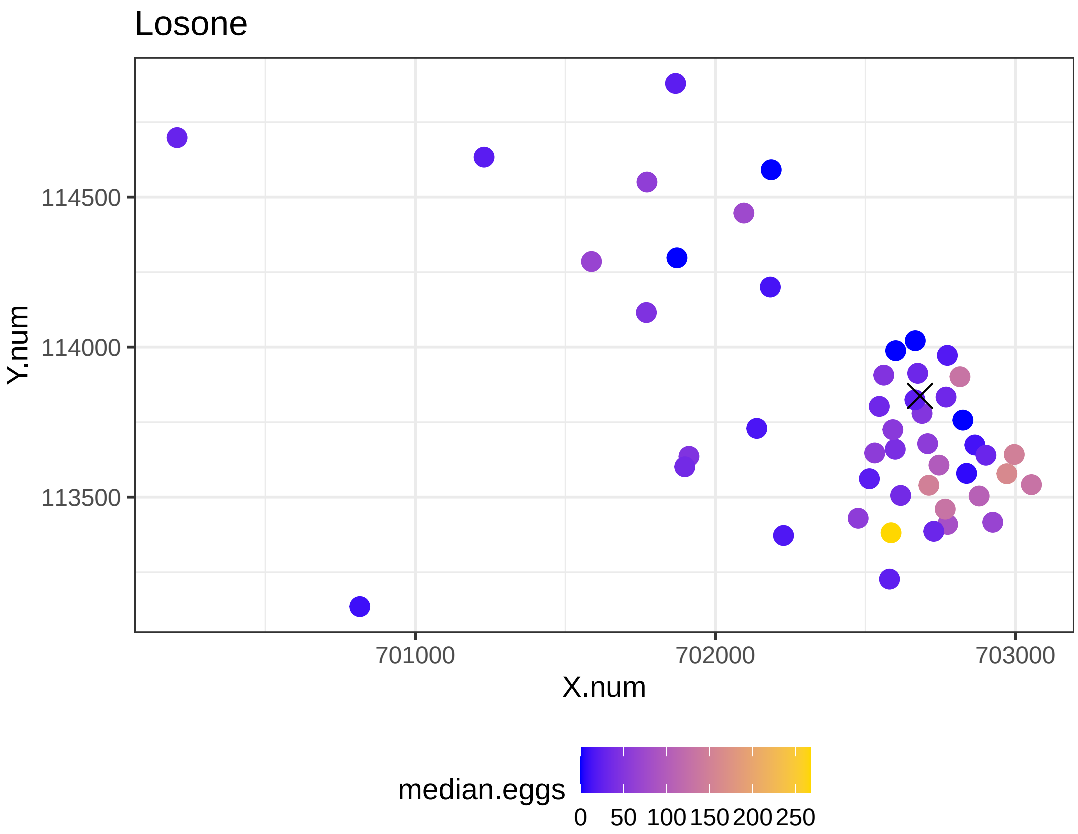

The spatial pattern is quite clear: closer to the treated zone, there seems to be a lower median egg count, whereas further away from it the counts seem to be higher. However, in the left-upper part of the map, there seems to be low counts as well, despite being far from the treated zone. However, that area has different characteristics, thus it's not really comparable to the treated zone.

The treated area is very small compared to the entire surface of the municipality. Therefore, we create a second data set where we focus on this area.

```
d.ovitraps.spatial.Losone.25.zoom <- d.ovitraps.spatial.Losone.25 %>%
  filter(## ovitraps with letters after the number are not part of the SIT
    ## design, thus we drop them.
    ## (see "istruzioni tabella ASC-LOS.docx")
    str_detect(unique.ID, pattern = "[a-zA-Z]\\.\\d+[a-z]", negate = TRUE)) %>%
  droplevels()
##
## check
unique(d.ovitraps.spatial.Losone.25.zoom$unique.ID)
```

```
 [1] Losone.1  Losone.10 Losone.11 Losone.12 Losone.13 Losone.14 Losone.15
 [8] Losone.16 Losone.17 Losone.18 Losone.19 Losone.2  Losone.20 Losone.21
[15] Losone.22 Losone.23 Losone.24 Losone.25 Losone.26 Losone.27 Losone.28
[22] Losone.3  Losone.4  Losone.5  Losone.6  Losone.7  Losone.8  Losone.9
28 Levels: Losone.1 Losone.10 Losone.11 Losone.12 Losone.13 ... Losone.9
```

```
## makes sense
##
## Set min / max for colour scale
max.eggs.median.25.zoom.Losone <- max(d.ovitraps.spatial.Losone.25.zoom$median.eggs)
min.eggs.median.25.zoom.Losone <- min(d.ovitraps.spatial.Losone.25.zoom$median.eggs)
##
```

```
gg.obs.L.25.zoom <- ggplot(d.ovitraps.spatial.Losone.25.zoom,
                           mapping = aes(y = Y.num,
                                         x = X.num,
                                         colour = median.eggs)) +
  geom_point(size = 3) +
  geom_point(d.coord.center.losone, mapping = aes(x = X, y = Y),
             colour = "black", shape = 4, size = 4) +
  scale_color_gradientn(colours = c("blue", "gold"),
                        values = scales::rescale(c(min.eggs.median.25.zoom.Losone,
                                                   max.eggs.median.25.zoom.Losone)),
                        limits = c(min.eggs.median.25.zoom.Losone,
                                   max.eggs.median.25.zoom.Losone)) +
  # theme(aspect.ratio = 1) +
  coord_fixed() +
  labs(title = "Losone") +
  theme(legend.position = "bottom")
gg.obs.L.25.zoom
```

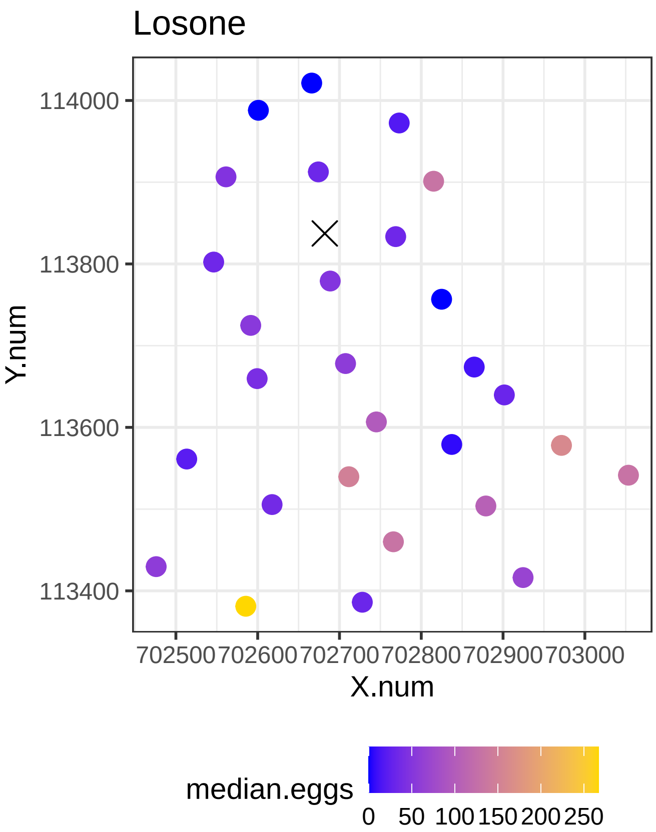

With this graph, the spatial pattern is a bit clearer.

We draw the same plots for Ascona.

```
## (warnings are omitted from this chunk)
##
gg.obs.A.25 <- ggplot(d.ovitraps.spatial.Ascona.25,
                      mapping = aes(y = Y.num,
                                    x = X.num,
                                    colour = median.eggs)) +
  geom_point(size = 3) +
  geom_point(d.coord.center.ascona, mapping = aes(x = X, y = Y),
             colour = "black", shape = 4, size = 4) +
  scale_color_gradientn(colours = c("blue", "gold"),
                        values = scales::rescale(c(min.eggs.median.25,
                                                   max.eggs.median.25)),
                        limits = c(min.eggs.median.25, max.eggs.median.25)) +
  # theme(aspect.ratio = 1) +
  coord_fixed() +
  labs(title = "Ascona") +
  theme(legend.position = "bottom")
gg.obs.A.25
```

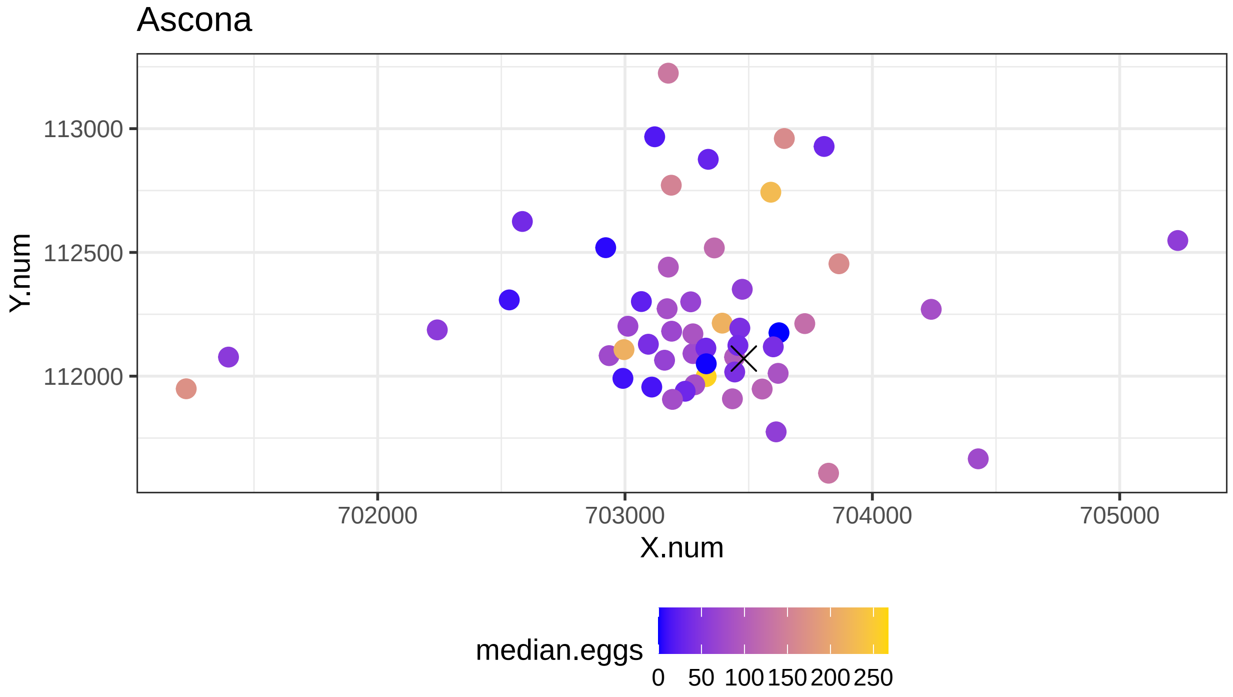

A spatial pattern is not as evident as in Losone.

We again concentrate on the treated zone and its neighbour area.

```
d.ovitraps.spatial.Ascona.25.zoom <- d.ovitraps.spatial.Ascona.25 %>%
  filter(## ovitraps with letters after the number are not part of the SIT
    ## design, thus we drop them.
    ## (see "istruzioni tabella ASC-LOS.docx")
    str_detect(unique.ID, pattern = "[a-zA-Z]\\.\\d+[a-z]", negate = TRUE)) %>%
  droplevels()
##
## check
unique(d.ovitraps.spatial.Ascona.25.zoom$unique.ID)
```

```
 [1] Ascona.1  Ascona.10 Ascona.11 Ascona.12 Ascona.13 Ascona.14 Ascona.15
 [8] Ascona.16 Ascona.17 Ascona.18 Ascona.19 Ascona.2  Ascona.20 Ascona.21
[15] Ascona.23 Ascona.24 Ascona.25 Ascona.26 Ascona.27 Ascona.28 Ascona.3
[22] Ascona.4  Ascona.5  Ascona.6  Ascona.7  Ascona.8  Ascona.9
27 Levels: Ascona.1 Ascona.10 Ascona.11 Ascona.12 Ascona.13 ... Ascona.9
```

```
## makes sense
## Set min / max for colour scale
max.eggs.median.25.zoom.Ascona <- max(d.ovitraps.spatial.Ascona.25.zoom$median.eggs)
min.eggs.median.25.zoom.Ascona <- min(d.ovitraps.spatial.Ascona.25.zoom$median.eggs)
```

```
gg.obs.A.25.zoom <- ggplot(d.ovitraps.spatial.Ascona.25.zoom,
                           mapping = aes(y = Y.num,
                                         x = X.num,
                                         colour = median.eggs)) +
  geom_point(size = 3) +
  geom_point(d.coord.center.ascona, mapping = aes(x = X, y = Y),
             colour = "black", shape = 4, size = 4) +
  scale_color_gradientn(colours = c("blue", "gold"),
```

```
                          values = scales::rescale(c(min.eggs.median.25.zoom.Ascona,
                                                     max.eggs.median.25.zoom.Ascona)),
                          limits = c(min.eggs.median.25.zoom.Ascona,
                                     max.eggs.median.25.zoom.Ascona)) +
  # theme(aspect.ratio = 1) +
  coord_fixed() +
  labs(title = "Ascona") +
  theme(legend.position = "bottom")
gg.obs.A.25.zoom
```

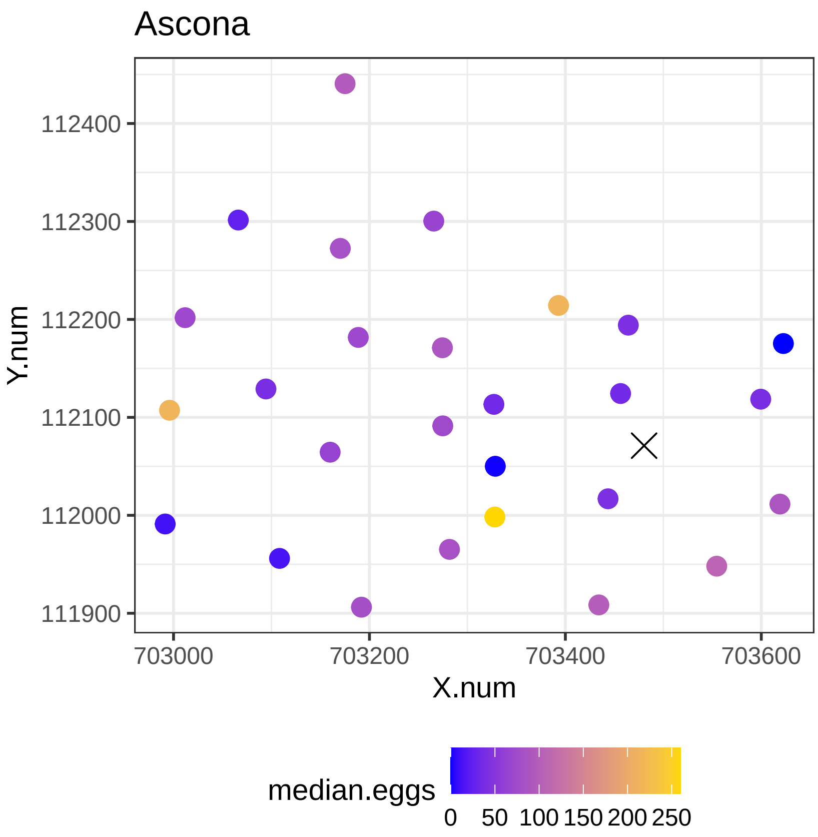

The spatial pattern is not so clear.

6.2 Fitting the models

We create two data sets containing only Ascona and Losone for the year 2025, respectively.

```
d.ovitraps.temporal %>%
  select(`total.albopictus.egg`, `Activation.time`,
         municipality.fac.spatial, unique.ID,
         Sampling_date.date, yday, Year.fac,
         X.num, Y.num) %>%
  na.omit() %>%
  droplevels() %>%
  nrow()
```

```
[1] 1180
```

```r
nrow(d.ovitraps.temporal)
```

```
[1] 1180
```

```r
## No missing values
##
d.ovitraps.temporal.Ascona.25 <- d.ovitraps.temporal %>%
  filter(municipality.fac.spatial == "Ascona" & Year.fac == "2025") %>%
  droplevels()
##
d.ovitraps.temporal.Losone.25 <- d.ovitraps.temporal %>%
  filter(municipality.fac.spatial == "Losone" & Year.fac == "2025") %>%
  droplevels()
##
## check
unique(d.ovitraps.temporal.Ascona.25$municipality.fac.spatial)
```

```
[1] Ascona
Levels: Ascona
```

```r
unique(d.ovitraps.temporal.Losone.25$municipality.fac.spatial)
```

```
[1] Losone
Levels: Losone
```

We also create two data sets where we zoom in the treated zone with its neighbour area, for both municipalities.

```r
d.ovitraps.temporal.Ascona.25.zoom <- d.ovitraps.temporal.Ascona.25 %>%
  filter(## ovitraps with letters after the number are not part of the SIT
    ## design, thus we drop them.
    ## (see "istruzioni tabella ASC-LOS.docx")
    str_detect(unique.ID, pattern = "[a-zA-Z]\\.\\d+[a-z]", negate = TRUE)) %>%
  droplevels()
## check
unique(d.ovitraps.temporal.Ascona.25.zoom$unique.ID)
```

```
 [1] Ascona.1  Ascona.2  Ascona.3  Ascona.4  Ascona.5  Ascona.6  Ascona.7
 [8] Ascona.9  Ascona.10 Ascona.11 Ascona.12 Ascona.14 Ascona.15 Ascona.16
[15] Ascona.18 Ascona.19 Ascona.21 Ascona.23 Ascona.25 Ascona.27 Ascona.28
[22] Ascona.13 Ascona.20 Ascona.24 Ascona.17 Ascona.26 Ascona.8
27 Levels: Ascona.1 Ascona.10 Ascona.11 Ascona.12 Ascona.13 ... Ascona.9
```

```r
## makes sense
##
d.ovitraps.temporal.Losone.25.zoom <- d.ovitraps.temporal.Losone.25 %>%
  filter(## ovitraps with letters after the number are not part of the SIT
    ## design, thus we drop them.
    ## (see "istruzioni tabella ASC-LOS.docx")
    str_detect(unique.ID, pattern = "[a-zA-Z]\\.\\d+[a-z]", negate = TRUE)) %>%
  droplevels()
## check
unique(d.ovitraps.temporal.Losone.25.zoom$unique.ID)
```

```
 [1] Losone.1  Losone.3  Losone.4  Losone.5  Losone.6  Losone.10 Losone.11
 [8] Losone.12 Losone.13 Losone.14 Losone.15 Losone.16 Losone.17 Losone.18
[15] Losone.19 Losone.21 Losone.22 Losone.23 Losone.24 Losone.25 Losone.26
```

```
[22] Losone.27 Losone.7  Losone.8  Losone.9  Losone.20 Losone.28 Losone.2
28 Levels: Losone.1 Losone.10 Losone.11 Losone.12 Losone.13 ... Losone.9
```

```
## makes sense
```

We fit two separate models, one for Losone and one for Ascona.

We aim to model the total number of laid *Aedes albopictus* eggs (*total.albopictus.eggs*) over the season (*yday*) and territory (*X.num*, *Y.num*).

yday will be included as numeric variable; the same holds true for *X.num* and *Y.num*, which represent the swiss coordinates.

Given that *total.albopictus.eggs* represents count data, we will use a negative binomial model, which is appropriate for handling overdispersion in count data.

We don't want to assume any specific seasonal pattern, therefore we will fit a Generalised Additive Model (GAM), which provides flexibility in determining the best shape for the seasonal trend in the given municipality. However, this flexibility comes at the cost of interpretability.

As before, we include *Activation.time* as an offset.

Let's fit the model for Losone.

Note that we use the element `s(X.num, Y.num)` to plot spatial data. We can use this isotropic smoothing because both predictors are spatial coordinates and we assume the spatial effect is isotropic (i.e., the same in all directions) and the two variables are on the same scale.

```
gam.tot.eggs_space.Losone.25 <- gamV(total.albopictus.egg ~
                                       s(yday) +
                                       offset(log(Activation.time)) +

                                       s(X.num, Y.num), ## new element!

                                     method = "GCV.Cp", ## as in gam
                                     family = "nb",
                                     data = d.ovitraps.temporal.Losone.25)
##
summary(gam.tot.eggs_space.Losone.25)
```

```
Family: Negative Binomial(0.557)
Link function: log

Formula:
total.albopictus.egg ~ s(yday) + offset(log(Activation.time)) +
    s(X.num, Y.num)

Parametric coefficients:
                   Estimate      Std. Error  z value               Pr(>|z|)
(Intercept) 0.9823146788188 0.0792561979375 12.39417 < 0.000000000000000222 ***
---
Signif. codes:  0 '***' 0.001 '**' 0.01 '*' 0.05 '.' 0.1 ' ' 1

Approximate significance of smooth terms:
                         edf          Ref.df    Chi.sq              p-value
s(yday)        7.41866423429  8.37648692713 201.52521 < 0.0000000000000002 ***
s(X.num,Y.num) 7.96549692764 10.79527396081  22.76449             0.018167 *
```

```
---
Signif. codes:  0 '***' 0.001 '**' 0.01 '*' 0.05 '.' 0.1 ' ' 1

R-sq.(adj) =   0.29   Deviance explained = 34.5%
-REML = 1828.1  Scale est. = 1         n = 386
```

```
##
## We also fit a model on the treated zone and its neighbour area
gam.tot.eggs_space.Losone.25_zoom <- gamV(total.albopictus.egg ~
                                            ## We need to specify k
                                            s(yday, k = 9) +
                                            offset(log(Activation.time)) +

                                            ## If we don't specify k, the
                                            ## model doesn't converge
                                            s(X.num, Y.num, k = 25), ## new element!

                                          method = "GCV.Cp", ## as in gam
                                          family = "nb",
                                          data = d.ovitraps.temporal.Losone.25.zoom)
summary(gam.tot.eggs_space.Losone.25_zoom)
```

```
Family: Negative Binomial(0.574)
Link function: log

Formula:
total.albopictus.egg ~ s(yday, k = 9) + offset(log(Activation.time)) +
    s(X.num, Y.num, k = 25)

Parametric coefficients:
                  Estimate     Std. Error  z value               Pr(>|z|)
(Intercept) 1.209898905746 0.089340555612 13.54255 < 0.0000000000000000222 ***
---
Signif. codes:  0 '***' 0.001 '**' 0.01 '*' 0.05 '.' 0.1 ' ' 1

Approximate significance of smooth terms:
                         edf         Ref.df   Chi.sq                 p-value
s(yday)         4.72935873171  5.75612566078 71.83570 < 0.0000000000000000222
s(X.num,Y.num) 14.41643497772 18.56934235649 59.39709            0.0000038032

s(yday)        ***
s(X.num,Y.num) ***
---
Signif. codes:  0 '***' 0.001 '**' 0.01 '*' 0.05 '.' 0.1 ' ' 1

R-sq.(adj) =  0.458   Deviance explained = 31.8%
-REML = 1106.3  Scale est. = 1         n = 223
```

Let's visualise the results.

```
plot.gam(gam.tot.eggs_space.Losone.25,
         ## Adding the shift would not give the expected result (i.e., direct
         ## interpretation). In fact, the intercept also contains the
         ## estimate for the coordinates, thus adding the shift would
```

```
    ## change the interpretation, providing an estimated shift when
    ## s(X,Y) = 0. In any case, we are interested
    ## in the spatial smooth. We include the seasonal pattern
    ## to control for it when estimating the spatial pattern.
    # shift = coef(gam.tot.eggs_space.Losone.25)["(Intercept)"],
    select = 1,
    trans = exp)
```

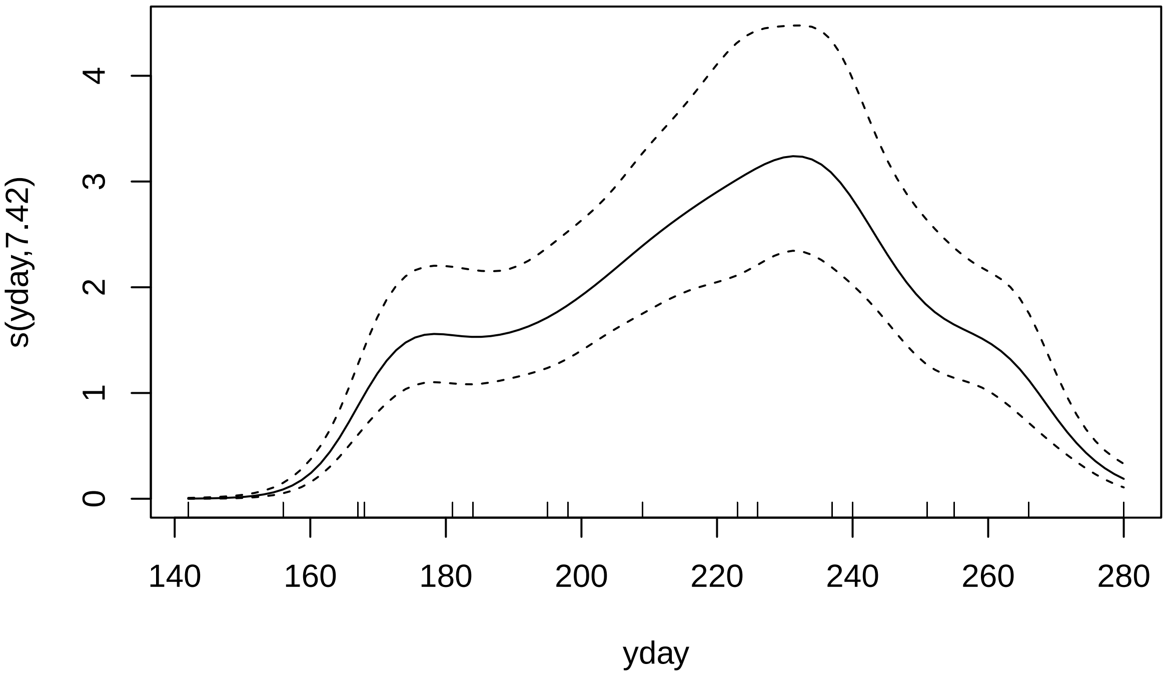


```
##
gg.gamViz.L.25 <- plot.gamViz(gam.tot.eggs_space.Losone.25,
                              ## Adding the shift doesn't change anything
                              ## for the spatial, because the legend is
                              ## relative to the mean value and it is
                              ## multiplicative. Thus, even if we shift everything
                              ## by the intercept and then back-transform,
                              ## this relationship doesn't change.
                              # shift = coef(gam.tot.eggs_space.Losone.25)["(Intercept)"],
                              select = 2,
                              trans = exp) +
  l_fitRaster() + l_fitContour() +
  l_points(shape = 16, col = "black", size = 2) +
  l_points(x = d.coord.center.losone$X, y = d.coord.center.losone$Y,
           colour = "red", size = 3, shape = 4) +
  coord_fixed() +
  labs(title = "Losone")
gg.gamViz.L.25
```

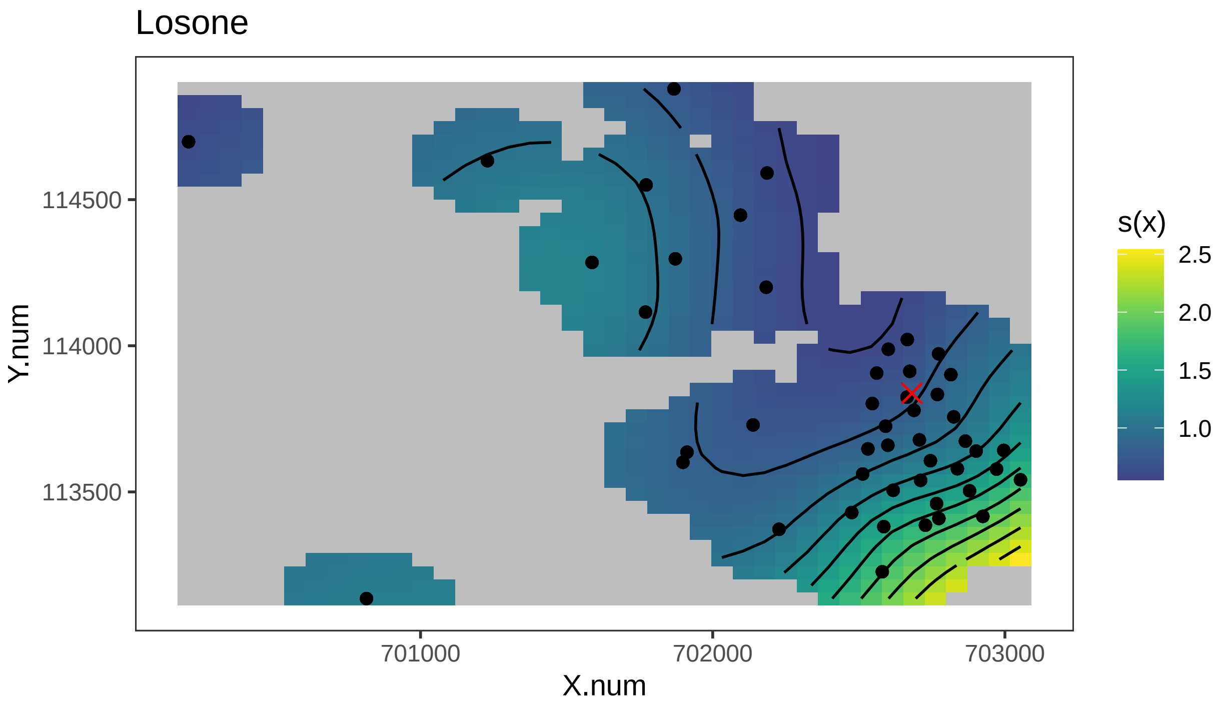

The spatial graph shows a small treatment effect. In fact, we observe that as we move away from the center of the treated zone (marked with a red cross), the number of eggs laid increases. In particular, this pattern is evident when moving toward the lower right corner of the plot. The upper right part has also low egg counts but, as previously noted, has different characteristics and is therefore not directly comparable to the treated zone.

Let's visualise the results of the second model, with data only on the treated and neighbour zones.

```
plot.gam(gam.tot.eggs_space.Losone.25_zoom,
         ## Here we don't add the shift because it would be wrong.
         ## In fact the intercept also contains the coordinates.
         select = 1,
         trans = exp)
```

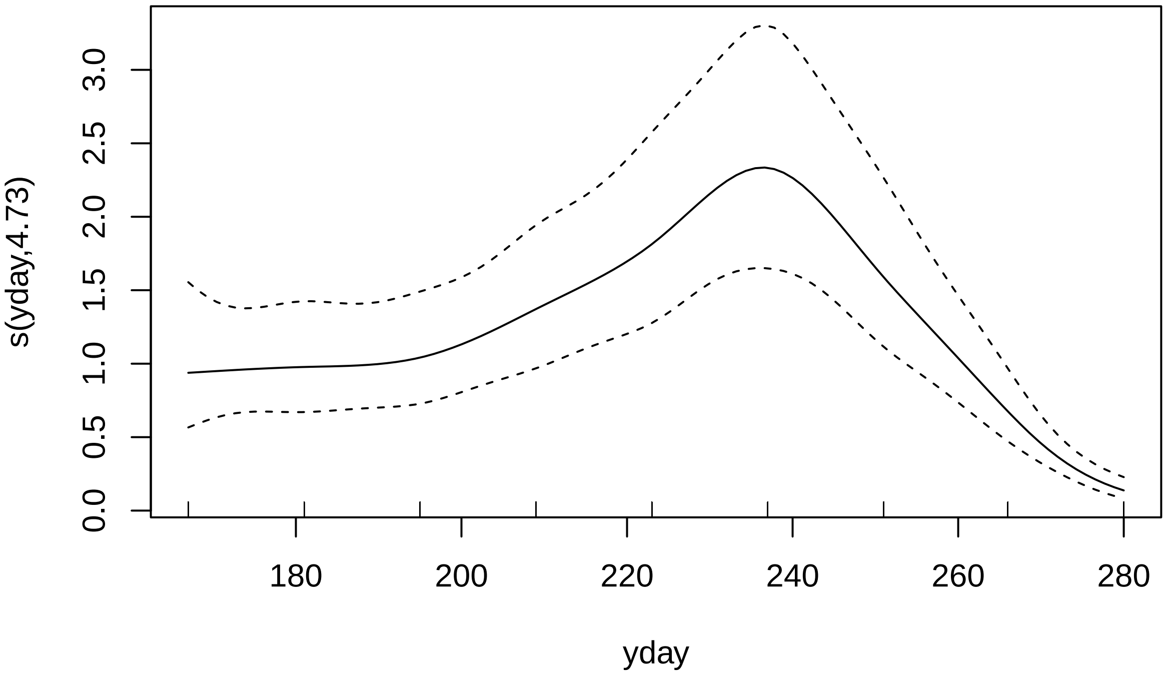


```r
##
gg.gamViz.L.25_zoom <- plot.gamViz(gam.tot.eggs_space.Losone.25_zoom,
                                   select = 2,
                                   trans = exp) +
  l_fitRaster() + l_fitContour() +
  l_points(shape = 16, col = "black", size = 2) +
  l_points(x = d.coord.center.losone$X, y = d.coord.center.losone$Y,
           colour = "red", size = 3, shape = 4) +
  coord_fixed() +
  labs(title = "Losone")
gg.gamViz.L.25_zoom
```

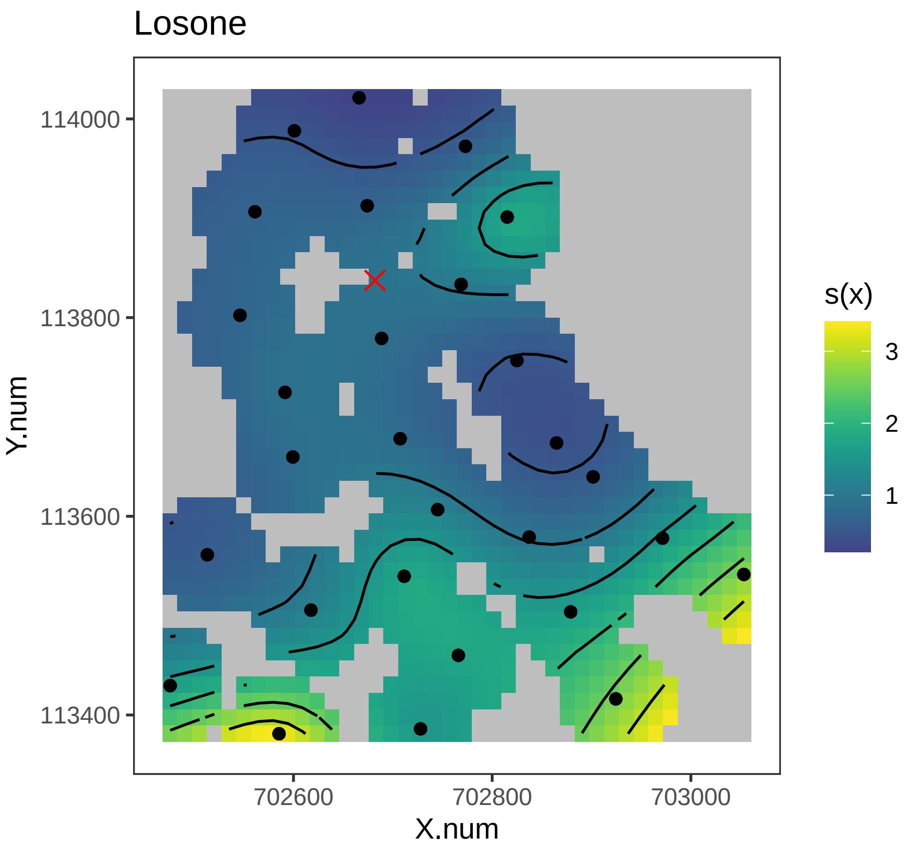

We have less data, therefore the model is not able to estimate a continuous smooth effect. However, we still see some spatial pattern, with increasing egg counts as we move away from the treated zone.

We refit the same model for Ascona.

```
gam.tot.eggs_space.Ascona.25 <- gamV(total.albopictus.egg ~
                                        s(yday) +
                                        offset(log(Activation.time)) +
                                        s(X.num, Y.num),

                                      method = "GCV.Cp", ## as in gam
                                      family = "nb",
                                      data = d.ovitraps.temporal.Ascona.25) ## new element!
##
summary(gam.tot.eggs_space.Ascona.25)
```

```
Family: Negative Binomial(0.706)
Link function: log

Formula:
total.albopictus.egg ~ s(yday) + offset(log(Activation.time)) +
    s(X.num, Y.num)

Parametric coefficients:
                   Estimate      Std. Error  z value              Pr(>|z|)
(Intercept) 1.5319116698509 0.0596786951745 25.66932 < 0.0000000000000000222 ***
---
```

```
Signif. codes:  0 '***' 0.001 '**' 0.01 '*' 0.05 '.' 0.1 ' ' 1

Approximate significance of smooth terms:
                          edf         Ref.df    Chi.sq              p-value
s(yday)          7.18106306575  8.23485080497 331.30449 < 0.0000000000000002 ***
s(X.num,Y.num) 12.44290372871 16.57757964797  25.41347              0.07404 .
---
Signif. codes:  0 '***' 0.001 '**' 0.01 '*' 0.05 '.' 0.1 ' ' 1

R-sq.(adj) =  0.266   Deviance explained = 33.6%
-REML = 2219.3  Scale est. = 1         n = 422
```

```
##
## We also fit a model on the treated zone and its neighbour area
gam.tot.eggs_space.Ascona.25_zoom <- gamV(total.albopictus.egg ~
                                            ## We need to specify k
                                            s(yday, k = 9) +
                                            offset(log(Activation.time)) +

                                            ## If we don't specify k, the
                                            ## model doesn't converge
                                            s(X.num, Y.num, k = 25), ## new element!

                                          method = "GCV.Cp", ## as in gam
                                          family = "nb",
                                          data = d.ovitraps.temporal.Ascona.25.zoom)
summary(gam.tot.eggs_space.Ascona.25_zoom)
```

```
Family: Negative Binomial(0.782)
Link function: log

Formula:
total.albopictus.egg ~ s(yday, k = 9) + offset(log(Activation.time)) +
    s(X.num, Y.num, k = 25)

Parametric coefficients:
                   Estimate      Std. Error  z value               Pr(>|z|)
(Intercept) 1.4922310545764 0.0801212612705 18.62466 < 0.000000000000000222 ***
---
Signif. codes:  0 '***' 0.001 '**' 0.01 '*' 0.05 '.' 0.1 ' ' 1

Approximate significance of smooth terms:
                          edf         Ref.df    Chi.sq                p-value
s(yday)          4.29676034819  5.26477985739  74.82051 < 0.000000000000000222
s(X.num,Y.num) 18.82421993375 22.13508550045 122.04327 < 0.000000000000000222

s(yday)        ***
s(X.num,Y.num) ***
---
Signif. codes:  0 '***' 0.001 '**' 0.01 '*' 0.05 '.' 0.1 ' ' 1

R-sq.(adj) =  0.234   Deviance explained = 38.1%
-REML = 1108.8  Scale est. = 1         n = 207
```

Let's visualise the results.

```
plot.gam(gam.tot.eggs_space.Ascona.25,
         ## Here we don't add the shift because it would be wrong.
         ## In fact the intercept also contains the coordinates.
         select = 1,
         trans = exp)
```

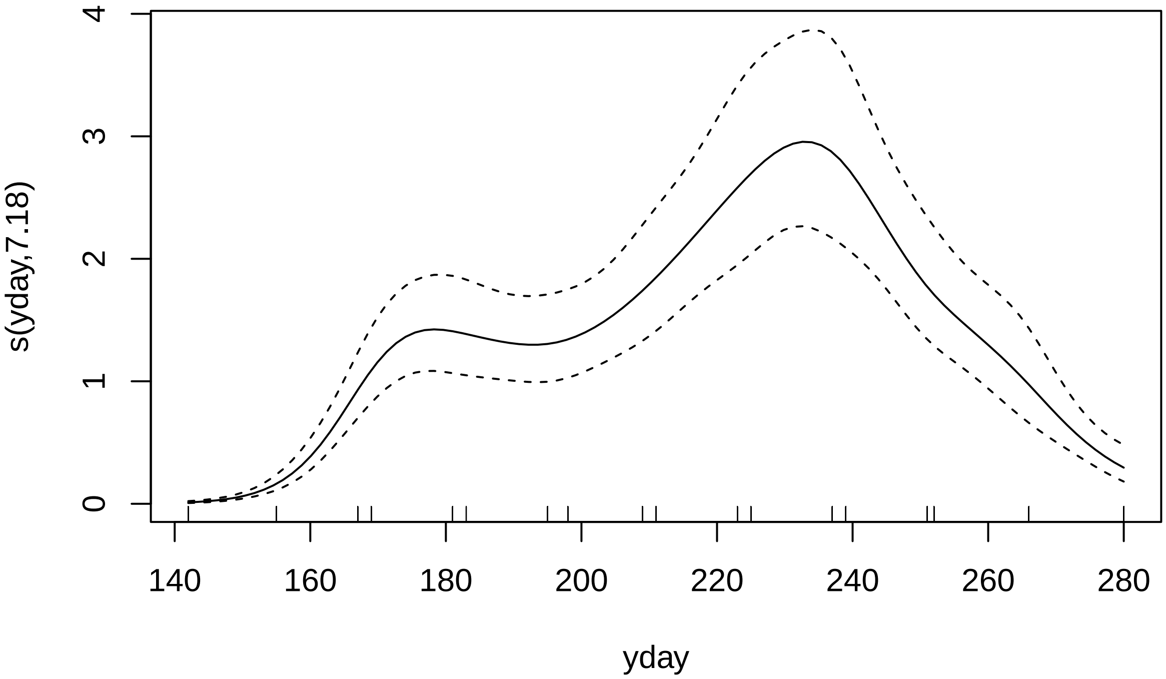


```
##
gg.gamViz.A.25 <- plot.gamViz(gam.tot.eggs_space.Ascona.25,
                              select = 2,
                              trans = exp) +
  l_fitRaster() + l_fitContour() +
  l_points(shape = 16, col = "black", size = 2) +
  l_points(x = d.coord.center.ascona$X, y = d.coord.center.ascona$Y,
           colour = "red", size = 3, shape = 4) +
  coord_fixed() +
  labs(title = "Ascona")
gg.gamViz.A.25
```

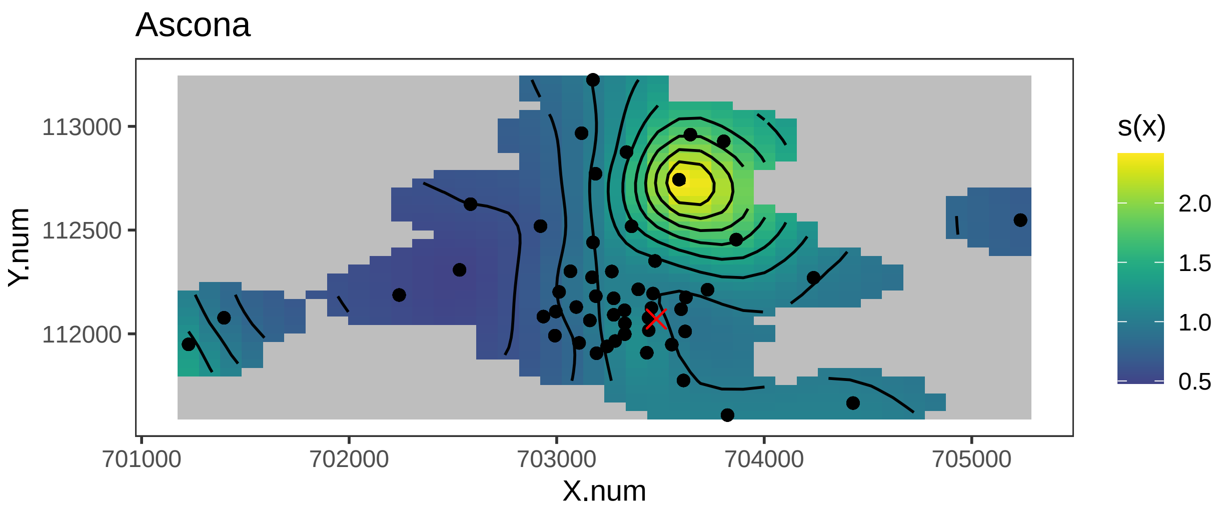

The spatial graph does not show a clear treatment effect. In fact, there is no evident pattern of increasing egg counts as we move away from the center of the treated zone (marked with a red cross).

Let's plot the results of the model fitted on the treated zone and its neighbour area.

```
plot.gam(gam.tot.eggs_space.Ascona.25_zoom,
         ## Here we don't add the shift because it would be wrong.
         ## In fact the intercept also contains the coordinates.
         select = 1,
         trans = exp)
```

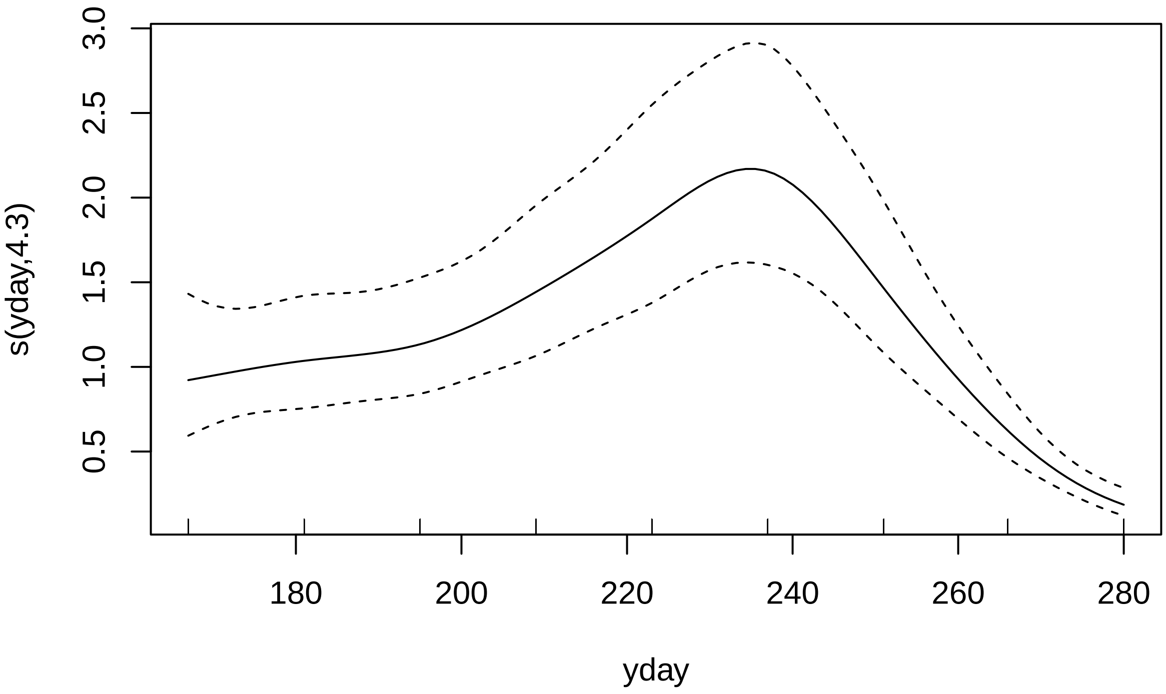


```
##
gg.gamViz.A.25_zoom <- plot.gamViz(gam.tot.eggs_space.Ascona.25_zoom,
                                   select = 2,
                                   trans = exp) +
  l_fitRaster() + l_fitContour() +
```

```r
  l_points(shape = 16, col = "black", size = 2) +
  l_points(x = d.coord.center.ascona$X, y = d.coord.center.ascona$Y,
           colour = "red", size = 3, shape = 4) +
  coord_fixed() +
  labs(title = "Ascona")
gg.gamViz.A.25_zoom
```

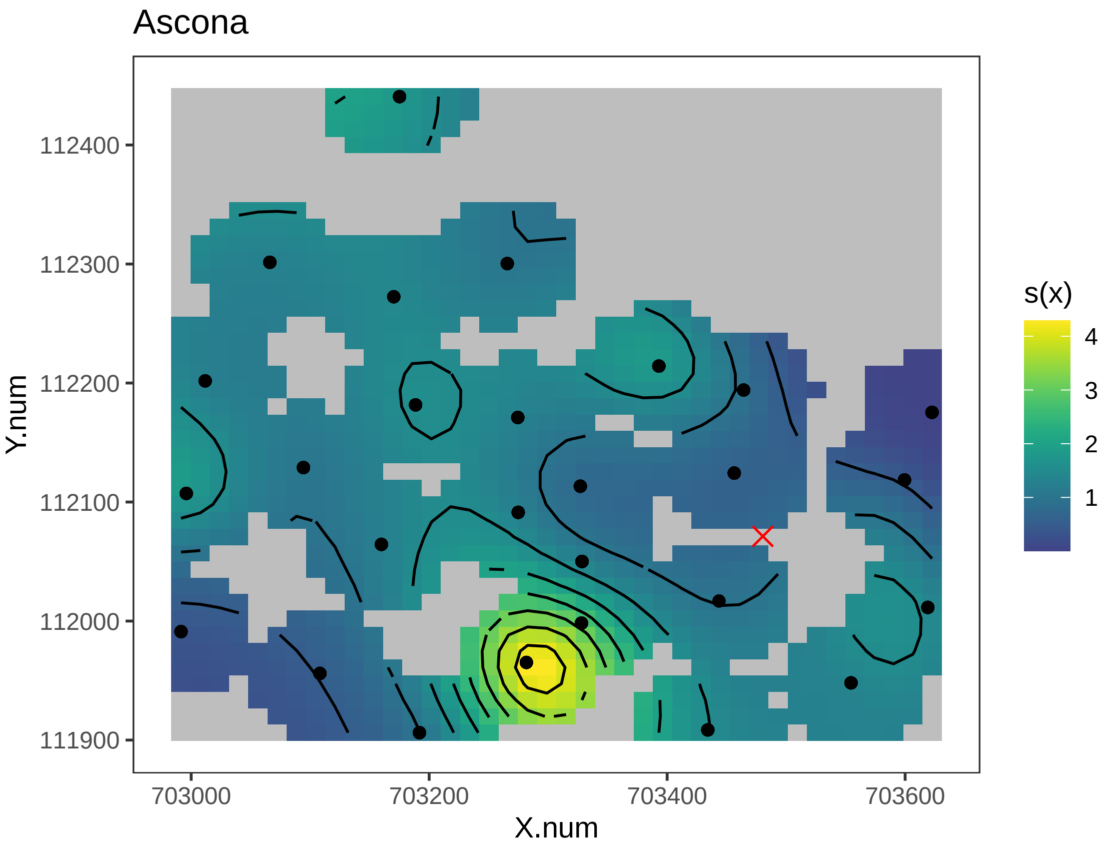

We have less data, therefore the model is not able to estimate a continuous smooth effect. However, from the available information, we don't see any clear spatial pattern.

6.3 Residual analysis

First of all, we apply the `gam.check()` function to the models, which produces some diagnostic information.

```r
## Losone
par(mfrow = c(2, 2))
gam.check(gam.tot.eggs_space.Losone.25)
```

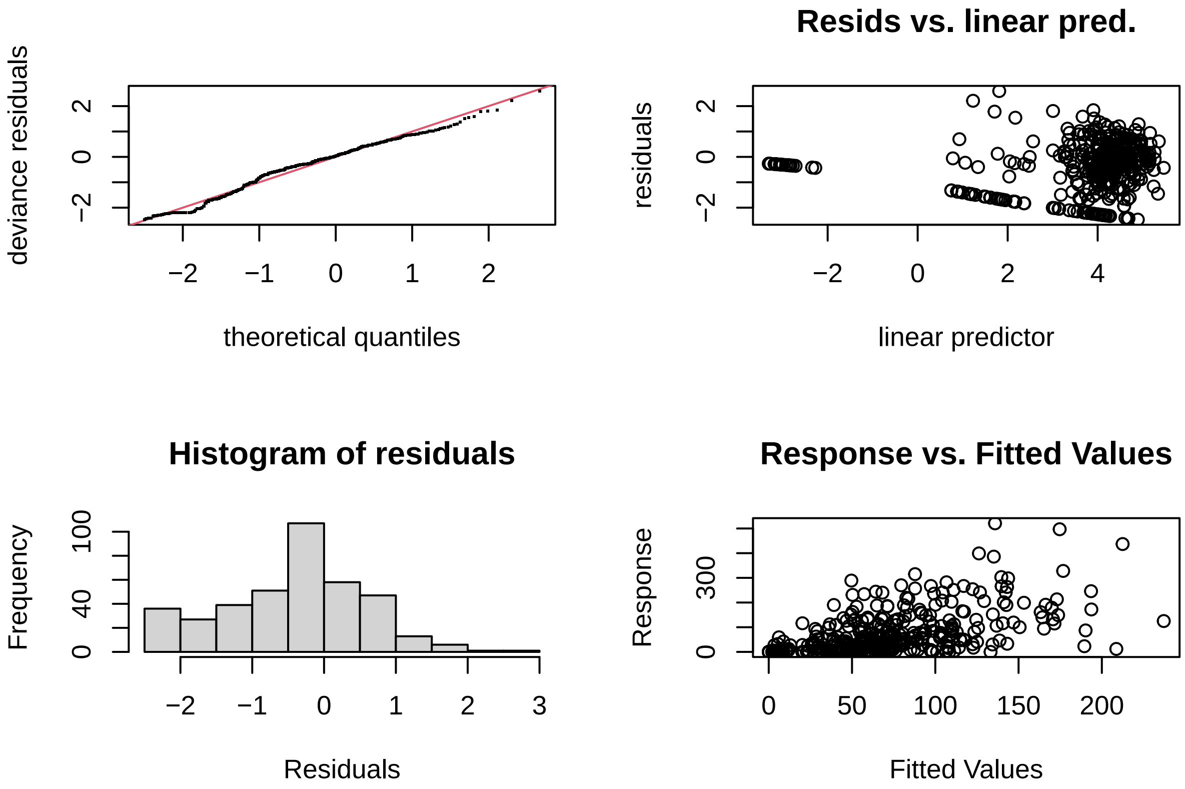


```
Method: REML   Optimizer: outer newton
full convergence after 4 iterations.
Gradient range [-0.000000394762429856144,0.00000871909394017933]
(score 1828.11963091439 & scale 1).
Hessian positive definite, eigenvalue range [1.24735682593573,173.635866478589].
Model rank =  39 / 39

Basis dimension (k) checking results. Low p-value (k-index<1) may
indicate that k is too low, especially if edf is close to k'.

                   k'  edf k-index p-value
s(yday)          9.00 7.42    0.80   0.100 .
s(X.num,Y.num) 29.00 7.97    0.79   0.095 .
---
Signif. codes:  0 '***' 0.001 '**' 0.01 '*' 0.05 '.' 0.1 ' ' 1
```

```r
par(mfrow = c(1, 1))
##
## Ascona
par(mfrow = c(2, 2))
gam.check(gam.tot.eggs_space.Ascona.25)
```

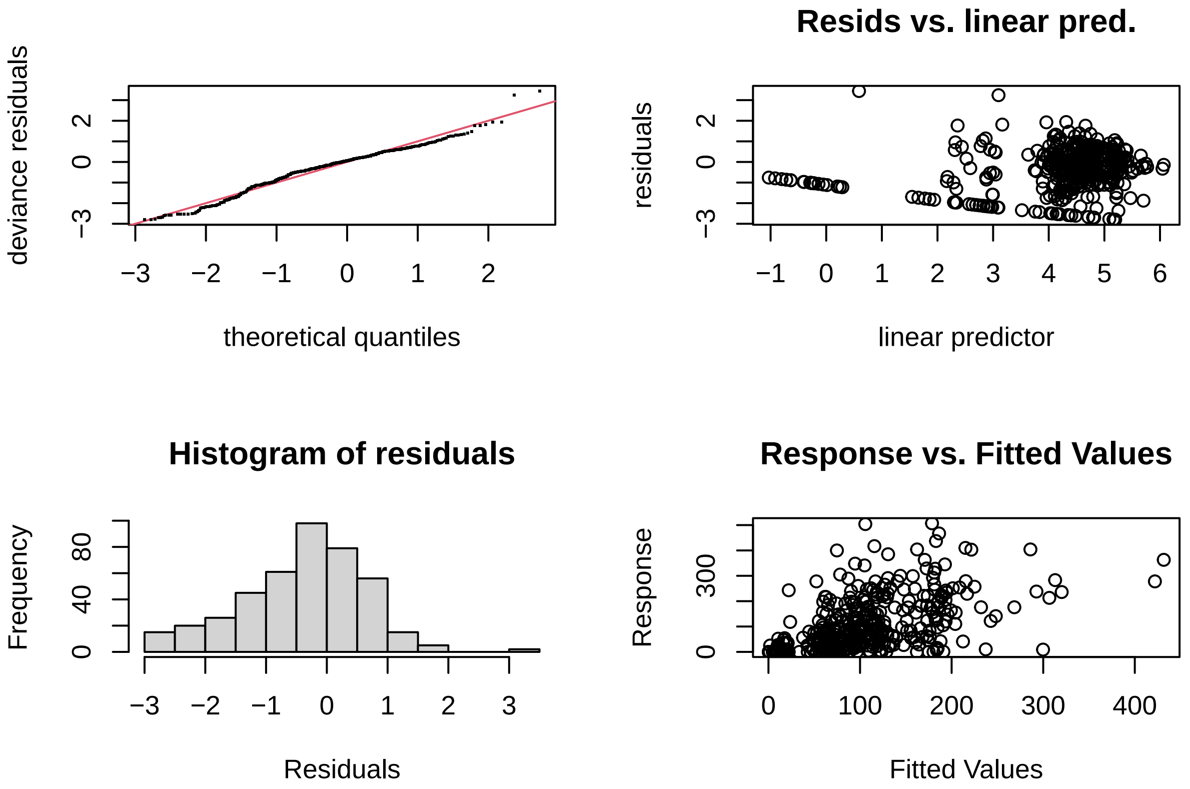


```
Method: REML   Optimizer: outer newton
full convergence after 6 iterations.
Gradient range [-0.000000382254432551576,0.000000167773843706698]
(score 2219.31642426853 & scale 1).
Hessian positive definite, eigenvalue range [0.758467315119842,194.228423362053].
Model rank =  39 / 39

Basis dimension (k) checking results. Low p-value (k-index<1) may
indicate that k is too low, especially if edf is close to k'.

                  k'   edf k-index              p-value
s(yday)         9.00  7.18    0.86                 0.34
s(X.num,Y.num) 29.00 12.44    0.68 <0.0000000000000002 ***
---
Signif. codes:  0 '***' 0.001 '**' 0.01 '*' 0.05 '.' 0.1 ' ' 1
```

```
par(mfrow = c(1, 1))
```

Then, we store the pearson residuals in the original data frame, and we plot the residuals against the fitted values to see whether there is still structure in the data.

```
## Losone
d.ovitraps.temporal.Losone.25$resid_gam.tot.eggs_space.Losone <-
  resid(gam.tot.eggs_space.Losone.25, type = "pearson")
##
d.ovitraps.temporal.Losone.25$fitted_gam.tot.eggs_space.Losone <-
  fitted(gam.tot.eggs_space.Losone.25)
##
ggplot(data = d.ovitraps.temporal.Losone.25,
```

```
       mapping = aes(y = resid_gam.tot.eggs_space.Losone,
                     x = fitted_gam.tot.eggs_space.Losone)) +
  geom_hline(yintercept = 0) +
  geom_point(alpha = 0.2) +
  geom_smooth()
```

```
`geom_smooth()` using method = 'loess' and formula = 'y ~ x'
```

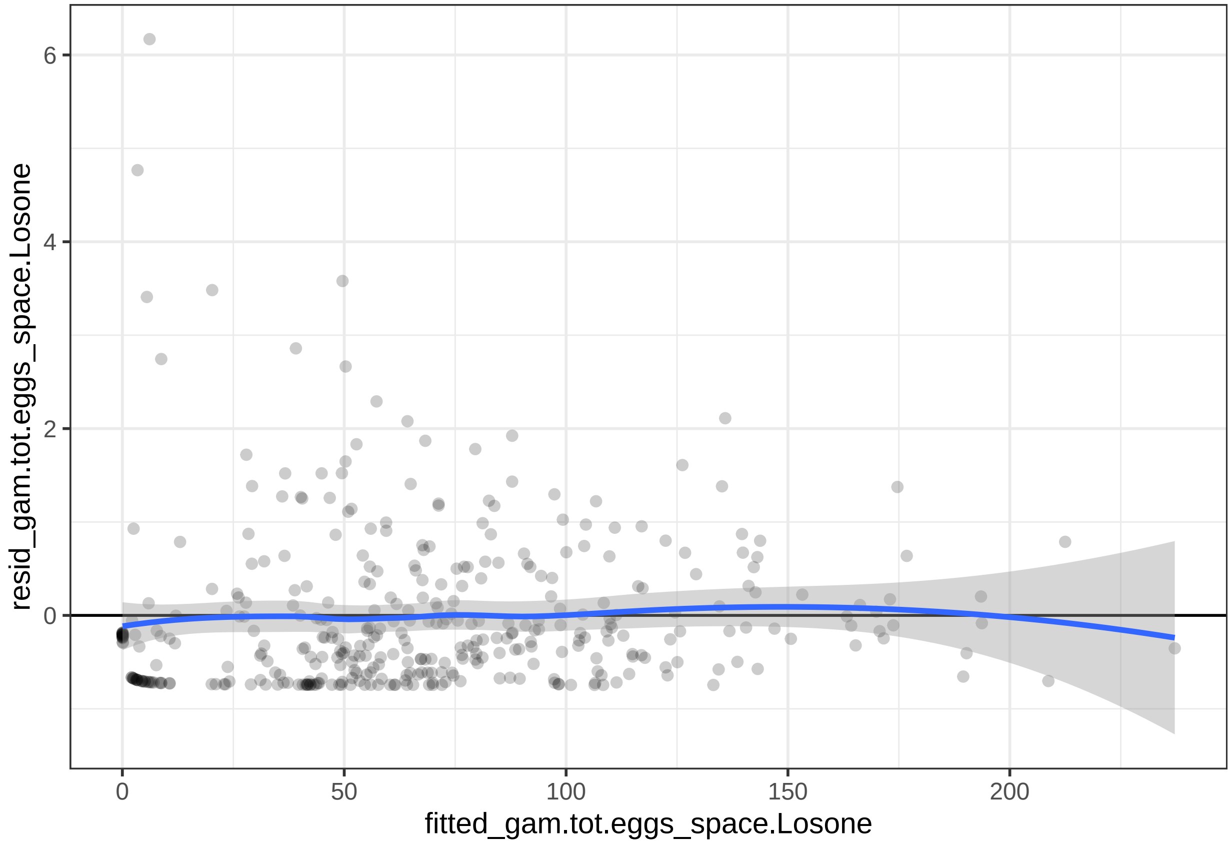


```
##
## Ascona
d.ovitraps.temporal.Ascona.25$resid_gam.tot.eggs_space.Ascona <-
  resid(gam.tot.eggs_space.Ascona.25, type = "pearson")
##
##
d.ovitraps.temporal.Ascona.25$fitted_gam.tot.eggs_space.Ascona <-
  fitted(gam.tot.eggs_space.Ascona.25)
##
ggplot(data = d.ovitraps.temporal.Ascona.25,
       mapping = aes(y = resid_gam.tot.eggs_space.Ascona,
                     x = fitted_gam.tot.eggs_space.Ascona)) +
  geom_hline(yintercept = 0) +
  geom_point(alpha = 0.2) +
  geom_smooth()
```

```
`geom_smooth()` using method = 'loess' and formula = 'y ~ x'
```

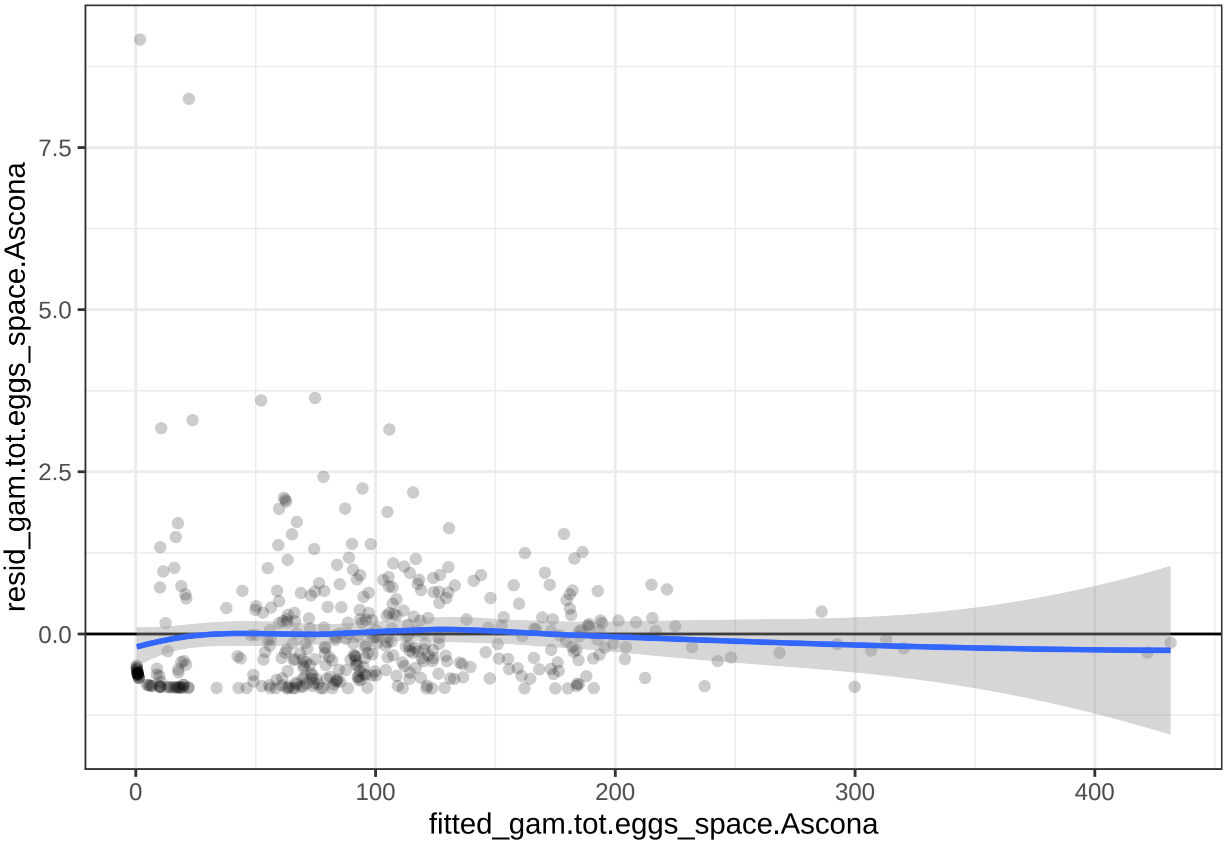

The blue lines are located on the x-axis (or the confidence band contains it), indicating that there doesn't seem to be structure left in the residuals.

7 Methods

7.1 Generalised Additive Mixed-Effects Model (GAMM)

We modelled the response variable “total number of Ae. albopictus eggs” (*total.albopictus.egg*) using a Generalised Additive Mixed Model (GAMM) with a negative binomial distribution to account for overdispersion. The model included a smooth term for sampling date, expressed as day of the year (*yday*), and we allowed this smooth to vary by treatment zone (*zona.SIT.discrete.fac*), resulting in zone-specific smooth functions. Treatment zone, a categorical variable with three levels, was included as a fixed effect. Differences in trap exposure time were accounted for by including *Activation.time* (in days) as an offset, using its logarithm so that egg counts were assumed proportional to exposure duration. To account for repeated measurements of the same trap, we included trap identity (*unique.ID*) as a random intercept.

This modelling procedure was applied separately for the municipalities of Ascona and Losone.

Model complexity was evaluated using Likelihood-Ratio (Chi-square) tests as well as AIC and BIC.

All analyses were performed in `R` using the packages `{mgcv}` and `{mgcViz}`, with statistical significance assessed at the 5% level. For additional details, see Section 5 of the Appendix.

@Diego: You can find the citations for the packages in the section "References"

7.2 Spatial Generalised Additive Model (spatial GAM)

We modelled the response variable “total number of Ae. albopictus eggs” (*total.albopictus.egg*) using a Generalised Additive Model (GAM) with a negative binomial distribution to account for overdispersion. The model included a smooth term for sampling date, expressed as day of the year (*yday*), and a bivariate smooth for geographic coordinates to capture spatial variation in egg counts. Differences in trap exposure time were accounted for by including *Activation.time* (in days) as an offset, using its logarithm so that egg counts were assumed proportional to exposure duration.

This modelling procedure was applied separately for the municipalities of Ascona and Losone.

Model complexity was evaluated using Likelihood-Ratio (Chi-square) tests as well as AIC and BIC.

All analyses were performed in `R` using the packages `{mgcv}` and `{mgcViz}`, with statistical significance assessed at the 5% level. For additional details, see Section 6 of the Appendix.

@Diego: You can find the citations for the packages in the section References

8 Results

8.1 Generalised Additive Mixed-Effects Model (GAMM)

All models converge without numerical issues. The proportion of deviance explained is 46.8 % for Losone and 53 % for Ascona, indicating a satisfactory fit for both municipalities.

Model comparison shows that the more complex models improve model performance. In particular, the models including the smooth–by–treatment interaction provide a significantly better fit than the ones without the interaction (Likelihood-Ratio Chi-square test, p= 0.000470484083753 $ <0.001$ for Losone, p= 0.000069314450677 $ <0.001$ for Ascona). This confirms that allowing for treatment-specific smooth functions captures additional structure in the data and contributes independently to explaining variability in egg counts.

The modelling results show that, once the treatment begins, egg counts in the treated zone are consistently lower than in the untreated (outside) zone. The buffer zone exhibits intermediate values.

These results are best summarised in Figure XYZ, which illustrates the seasonal dynamics across the three zones.

Alternatively, the differences in egg counts between the treated and untreated zones can be examined directly. This is shown in Figure WXZ, which clearly confirms that, after the treatment kicks in, egg counts in the treated area remain consistently lower than those in the untreated area.

The magnitude of the reduction is not constant but varies over time. At its peak, the reduction in Losone is predicted to be 67.0%, whereas in Ascona it is predicted to be 57.0%.

@Diego: For the p-values, choose the notation the journal prefers.

@Diego: The percentage reductions at peak are calculated in the document "3 tables SIT 2025 losone ascona.pdf"

@Diego: Please insert the correct number for the figures

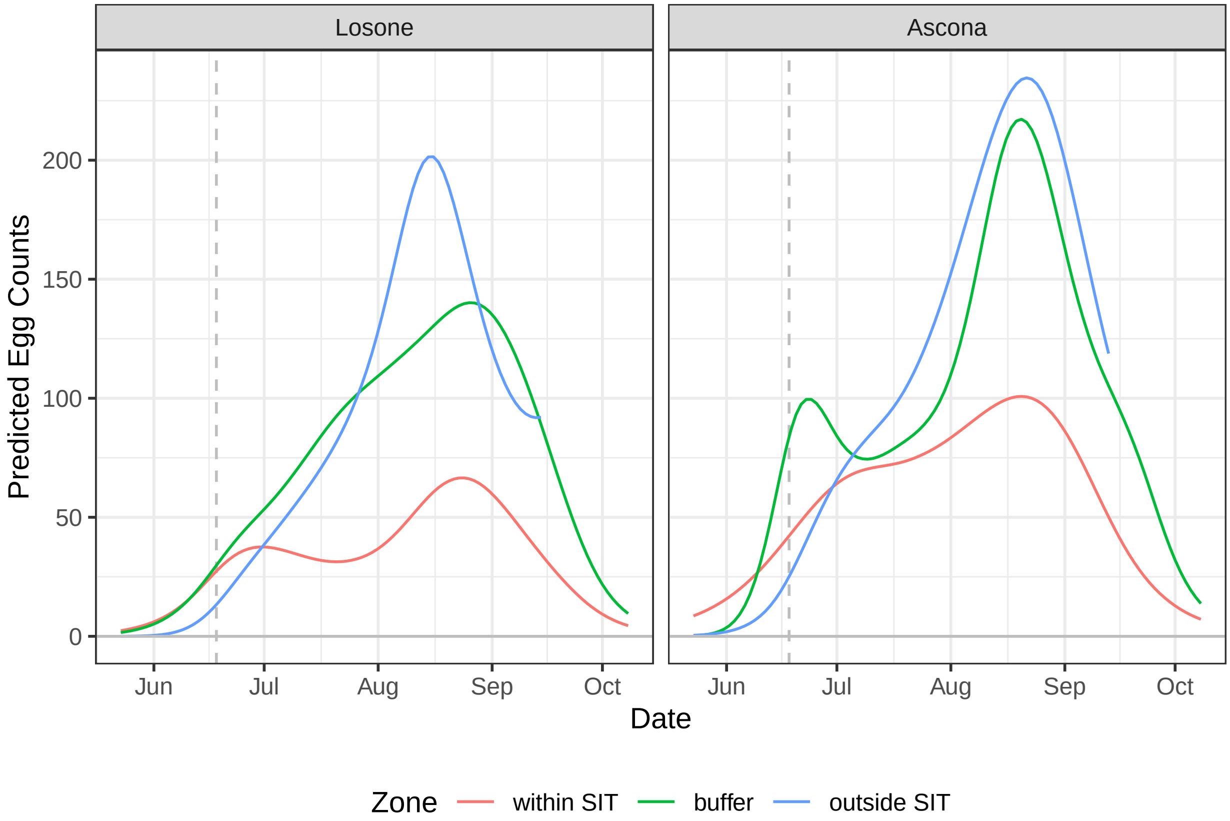

Figure 1: This figure shows the predicted egg counts over time for Losone (left) and Ascona (right). Each plot displays three lines: blue for the untreated zone, red for the treated zone, and green for the buffer zone. The dashed vertical line at 17.06.2025 marks the start of the treatment. In both municipalities, egg counts in the treated zone decrease relative to the untreated zone after the treatment begins, while the buffer zone shows intermediate values.

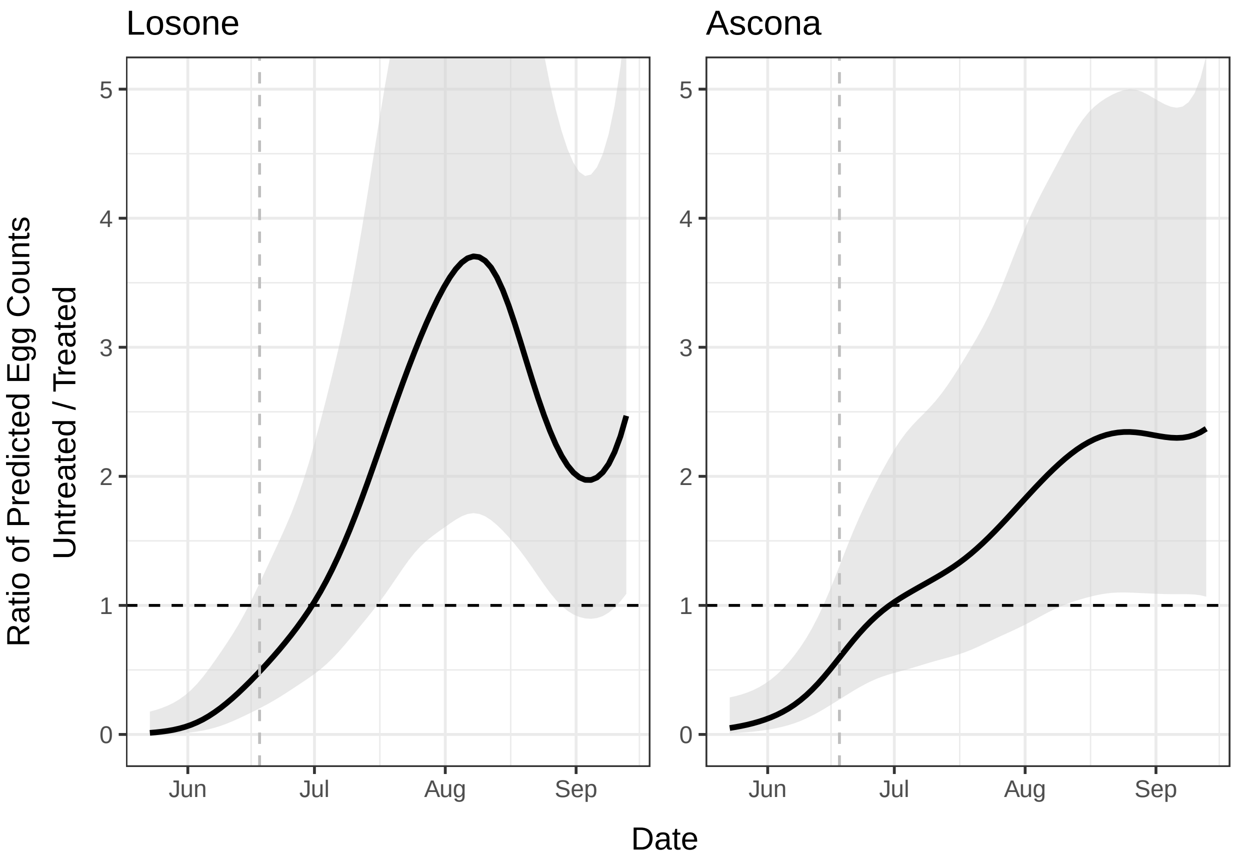

Figure 2: This figure shows the ratio of the predicted egg counts between the untreated zone and the treated zone over time for Losone (left) and Ascona (right). The solid line represents the predicted ratio, while the shaded area indicates the 95% confidence interval. The dashed horizontal line at 1 indicates no difference between zones. Values above this line indicate higher egg counts in the untreated zone, while values below it indicate higher egg counts in the treated zone. The vertical dashed line marks the start of the treatment on 17.06.2025. After the treatment kicks in, egg counts in the treated area remain consistently lower than those in the untreated area.

8.2 Spatial Generalised Additive Model (spatial GAM)

All models converge without numerical issues. The proportion of deviance explained is 34.5 % for Losone and 33.6 % for Ascona, indicating a sufficiently good fit for both municipalities.

@Diego: We suggest to not put the spatial part in the paper, as no clear spatial pattern emerges. Therefore, this part does not add much to the discussion.

9 Conclusions

The GAMM results indicate a clear treatment effect in both Losone and Ascona. After the intervention begins, the estimated differences between untreated and treated zones become consistently positive, with several periods in which these differences are statistically significant. The difference between the buffer zone and the treated zone is also positive, though less pronounced than the difference with the untreated area.

These findings indicate that, following the treatment, egg counts in the treated zones are estimated to be lower than in the other zones.

Spatial patterns are less clear. In Ascona, the spatial smooth surface does not display a distinct structure associated with the treatment zone. In Losone, there is some indication of a spatial gradient consistent with lower values in treated areas, although this pattern is weaker and less consistent than the temporal effect.

@Diego: You can omit the conclusion about spatial patterns if you don't include this chapter in the paper.

@Diego: Overall, the results highlight the importance of collecting pre-treatment data from the same zones to reliably quantify treatment effects. Indeed, the temporal patterns show that treated zones exhibit higher egg counts prior to the intervention compared to the other zones, indicating that the control areas are not directly comparable to the treated ones.

@Diego: We motivate the choice of these controls as follows: We treated the areas that the municipality allowed us to access, selecting only those large enough to include both the buffer zone and the outer area. As a result, the available control sites were simply those located within these permitted areas, without the possibility of achieving a more balanced selection. The fact that the controls are lower is therefore not a methodological issue: the key comparison is not only between inside and outside areas, but also within each site relative to its own pre-treatment trends. In this way, each area effectively serves as its own control over time, reducing the risk of bias due to initial structural differences.

10 References

```r
## Packages
citation("mgcViz")
```

```
To cite the mgcViz package in publications use:

  Fasiolo, M., Nedellec, R., Goude, Y. and Wood, S.N., 2020. Scalable
  visualization methods for modern generalized additive models. Journal
  of computational and Graphical Statistics, 29(1), pp.78-86.

A BibTeX entry for LaTeX users is

  @Article{,
    title = {Scalable visualisation methods for modern Generalized Additive Models.},
    journal = {Journal of the Royal Statistical Society (B)},
    volume = {29},
    number = {1},
    pages = {78-86},
    year = {2020},
    author = {{Fasiolo} and {Matteo} and {Nedellec} and {Rapha{"e}l} and {Goude} and {Yannig} and {Wood[illegible]
  }

As mgcViz is often updated, you may want to cite its version number.
Find it with 'help(package=mgcViz)'.
```

```r
citation("mgcv")[[1]] ## return several papers
```

```
Wood, S.N. (2011) Fast stable restricted maximum likelihood and
marginal likelihood estimation of semiparametric generalized linear
models. Journal of the Royal Statistical Society (B) 73(1):3-36

A BibTeX entry for LaTeX users is
```

```
  @Article{,
    title = {Fast stable restricted maximum likelihood and marginal
likelihood estimation of semiparametric generalized linear models},
    journal = {Journal of the Royal Statistical Society (B)},
    volume = {73},
    number = {1},
    pages = {3--36},
    year = {2011},
    author = {S. N. Wood},
    doi = {10.1111/j.1467-9868.2010.00749.x},
  }
```

```r
##
## R itself
citation()
```

```
To cite R in publications use:

  R Core Team (2025). _R: A Language and Environment for Statistical
  Computing_. R Foundation for Statistical Computing, Vienna, Austria.
  <https://www.R-project.org/>.

A BibTeX entry for LaTeX users is

  @Manual{,
    title = {R: A Language and Environment for Statistical Computing},
    author = {{R Core Team}},
    organization = {R Foundation for Statistical Computing},
    address = {Vienna, Austria},
    year = {2025},
    url = {https://www.R-project.org/},
  }

We have invested a lot of time and effort in creating R, please cite it
when using it for data analysis. See also 'citation("pkgname")' for
citing R packages.
```

11 Appendix

11.1 Number of eggs vs distance from the center of the treatment

We are going to model the distance from the centre of the treatment as continuous variable. Note this model does not discretise the ovitraps into three groups (i.e., within SIT, buffer and outside SIT) as the models above, but it accounts for the treatment effect via the distance to the centre of the treated zone. So, in a sense, it better uses the spatial information.

On the other hand, this model assumes that the effect of seasonality is the same for all ovitraps regardless of in which zone they are located. We saw in the previous models that the seasonality shapes seem to differ.

Therefore, a-priori, it is hard to say whether this or the models above make better use of the information available.

11.1.1 Fitting the models

```r
d.ovitraps.temporal <- d.ovitraps.temporal %>%
  group_by(municipality.fac) %>%
  mutate(distance.from.center = case_when(
    Year.fac == 2024 ~  max(distance.from.center, na.rm = TRUE),
    .default = distance.from.center
  )) %>%
  ungroup()
##
## check
d.ovitraps.temporal %>%
  filter(Year.fac == 2024 & municipality.fac == "Ascona") %>%
  pull(zona.SIT.discrete.fac) %>%
  unique()
```

```
[1] outside SIT
Levels: within SIT buffer outside SIT
```

```r
## only outside SIT, makes sense
##
d.ovitraps.temporal %>%
  filter(Year.fac == 2024 & municipality.fac == "Ascona") %>%
  pull(distance.from.center) %>%
  unique()
```

```
[1] 2260
```

```r
d.ovitraps.temporal %>%
  filter(Year.fac == 2025 & municipality.fac == "Ascona") %>%
  pull(distance.from.center) %>%
  max()
```

```
[1] 2260
```

```r
## single distance, equal to max distance for 2025 in Ascona, makes sense
##
d.ovitraps.temporal %>%
  filter(Year.fac == 2024 & municipality.fac == "Losone") %>%
  pull(zona.SIT.discrete.fac) %>%
  unique()
```

```
[1] outside SIT
Levels: within SIT buffer outside SIT
```

```r
## only outside SIT, makes sense
##
d.ovitraps.temporal %>%
  filter(Year.fac == 2024 & municipality.fac == "Losone") %>%
  pull(distance.from.center) %>%
  unique()
```

```
[1] 2610
```

```r
d.ovitraps.temporal %>%
  filter(Year.fac == 2025 & municipality.fac == "Losone") %>%
  pull(distance.from.center) %>%
  max()
```

```
[1] 2610
```

```r
## single distance, equal to max distance fro 2025 in Losone, makes sense
##
## We create two separate data sets for the two municipalities
d.ovitraps.temporal.Losone.new <- d.ovitraps.temporal %>%
  filter(municipality.fac == "Losone")
##
d.ovitraps.temporal.Ascona.new <- d.ovitraps.temporal %>%
  filter(municipality.fac == "Ascona")
```

We fit the models.

```r
## with distance from center as continuous variable
##
gamm.eggs.Losone.continuous <- gamV(total.albopictus.egg ~

                                      offset(log(Activation.time)) +
                                      s(unique.ID, bs = "re") +

                                      Year.fac +

                                      s(yday) +
                                      s(distance.from.center),

                                    family = "nb",
                                    method = "GCV.Cp", ## same as gam
                                    data = d.ovitraps.temporal.Losone.new)
summary(gamm.eggs.Losone.continuous)
```

```

Family: Negative Binomial(0.56)
Link function: log

Formula:
total.albopictus.egg ~ offset(log(Activation.time)) + s(unique.ID,
    bs = "re") + Year.fac + s(yday) + s(distance.from.center)

Parametric coefficients:
                    Estimate      Std. Error  z value Pr(>|z|)
(Intercept)    0.861186667900  0.371013296536  2.32117 0.020277 *
Year.fac2025 -0.199340075776  0.509663331716 -0.39112 0.695708
---
Signif. codes:  0 '***' 0.001 '**' 0.01 '*' 0.05 '.' 0.1 ' ' 1

Approximate significance of smooth terms:
                                 edf          Ref.df    Chi.sq
s(unique.ID)            26.47055690866 47.00000000000  62.59172
s(yday)                  7.68518821805  8.53789997327 373.12688
s(distance.from.center)  2.20250004484  2.60012296281   2.20014
                                     p-value
s(unique.ID)                    0.0000015943 ***
s(yday)                 < 0.0000000000000222 ***
s(distance.from.center)              0.31802
---
```

```
Signif. codes:  0 '***' 0.001 '**' 0.01 '*' 0.05 '.' 0.1 ' ' 1

R-sq.(adj) =  0.369   Deviance explained = 45.2%
-REML =   2543  Scale est. = 1         n = 552
```

```r
##
##
gamm.eggs.Ascona.continuous <- gamV(total.albopictus.egg ~

                                      offset(log(Activation.time)) +
                                      s(unique.ID, bs = "re") +

                                      Year.fac +

                                      s(yday) +
                                      s(distance.from.center),

                                    family = "nb",
                                    method = "GCV.Cp", ## same as gam
                                    data = d.ovitraps.temporal.Ascona.new)
summary(gamm.eggs.Ascona.continuous)
```

```
Family: Negative Binomial(0.774)
Link function: log

Formula:
total.albopictus.egg ~ offset(log(Activation.time)) + s(unique.ID,
    bs = "re") + Year.fac + s(yday) + s(distance.from.center)

Parametric coefficients:
                   Estimate     Std. Error z value     Pr(>|z|)
(Intercept)  1.041299894742 0.232826600937 4.47243 0.0000077337 ***
Year.fac2025 0.231231337517 0.299423459543 0.77226      0.43996
---
Signif. codes:  0 '***' 0.001 '**' 0.01 '*' 0.05 '.' 0.1 ' ' 1

Approximate significance of smooth terms:
                                  edf         Ref.df    Chi.sq
s(unique.ID)            43.20908043926 51.00000000000 317.99488
s(yday)                  7.44591282197  8.41900456838 717.17078
s(distance.from.center)  1.01767537179  1.03150298233   0.04294
                                    p-value
s(unique.ID)            < 0.0000000000000002 ***
s(yday)                 < 0.0000000000000002 ***
s(distance.from.center)             0.86302
---
Signif. codes:  0 '***' 0.001 '**' 0.01 '*' 0.05 '.' 0.1 ' ' 1

R-sq.(adj) =  -2.07   Deviance explained = 50.5%
-REML = 3186.3  Scale est. = 1         n = 628
```

```r
plot.gam(gamm.eggs.Losone.continuous, pages = 1)
```

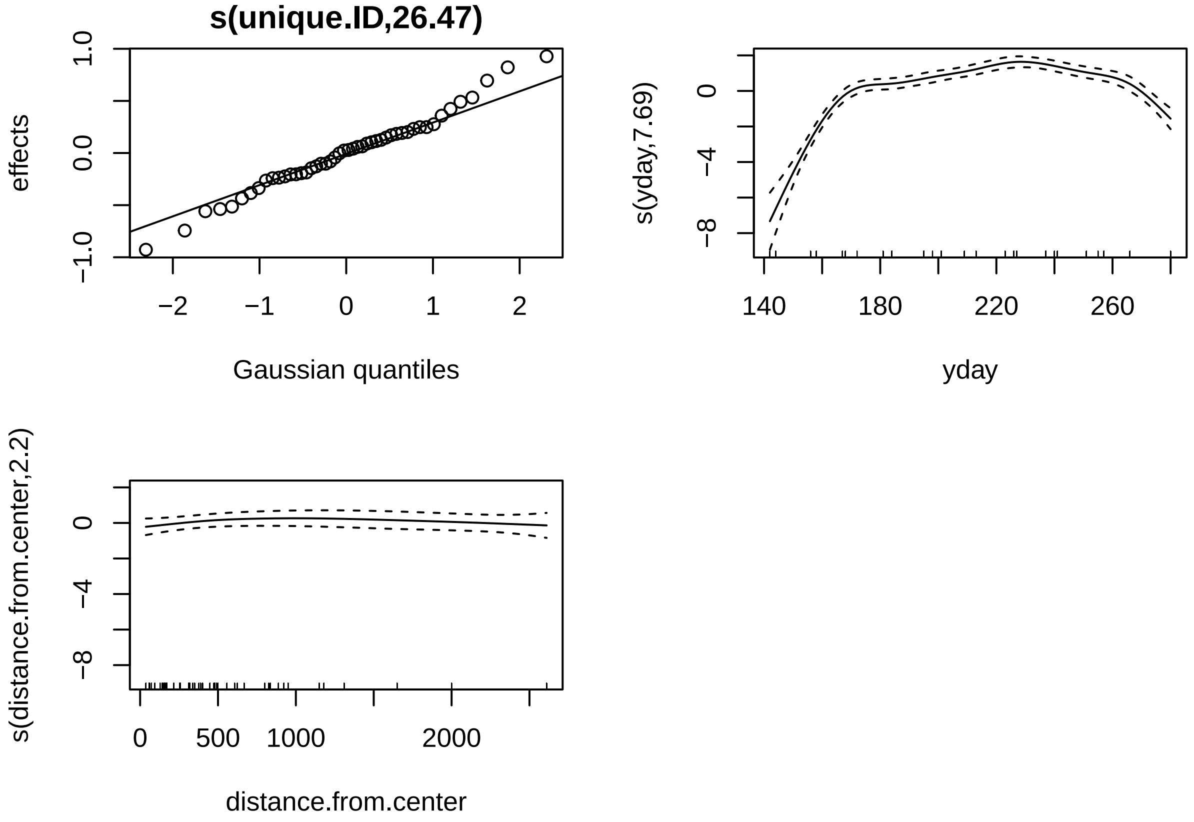


```r
plot.gam(gamm.eggs.Losone.continuous,
         select = 3,
         trans = exp,
         ylim = c(0, 5),
         shift = gamm.eggs.Losone.continuous$coefficients["(Intercept)"])
abline(h = 1, col = "lightgray")
```

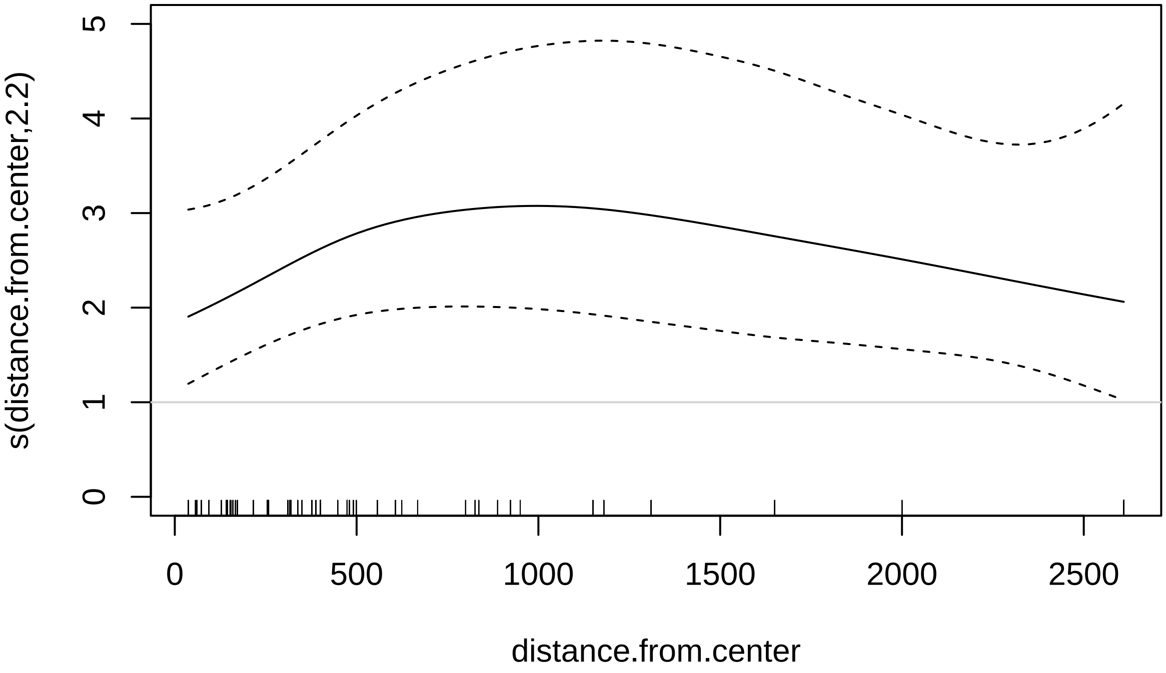

```
plot.gam(gamm.eggs.Ascona.continuous, pages = 1)
```

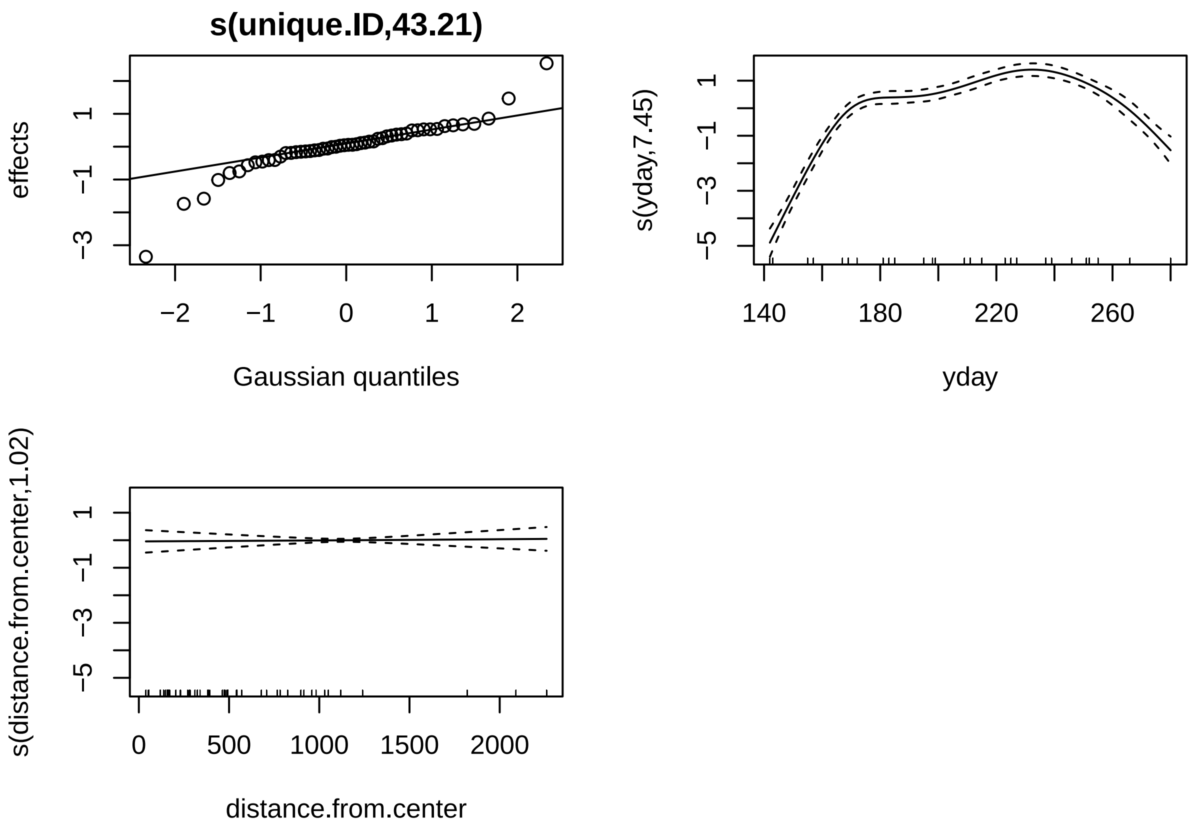


```
plot.gam(gamm.eggs.Ascona.continuous,
         select = 3,
         trans = exp,
         ylim = c(0, 5),
         shift = gamm.eggs.Ascona.continuous$coefficients["(Intercept)"])
abline(h = 1, col = "lightgray")
```

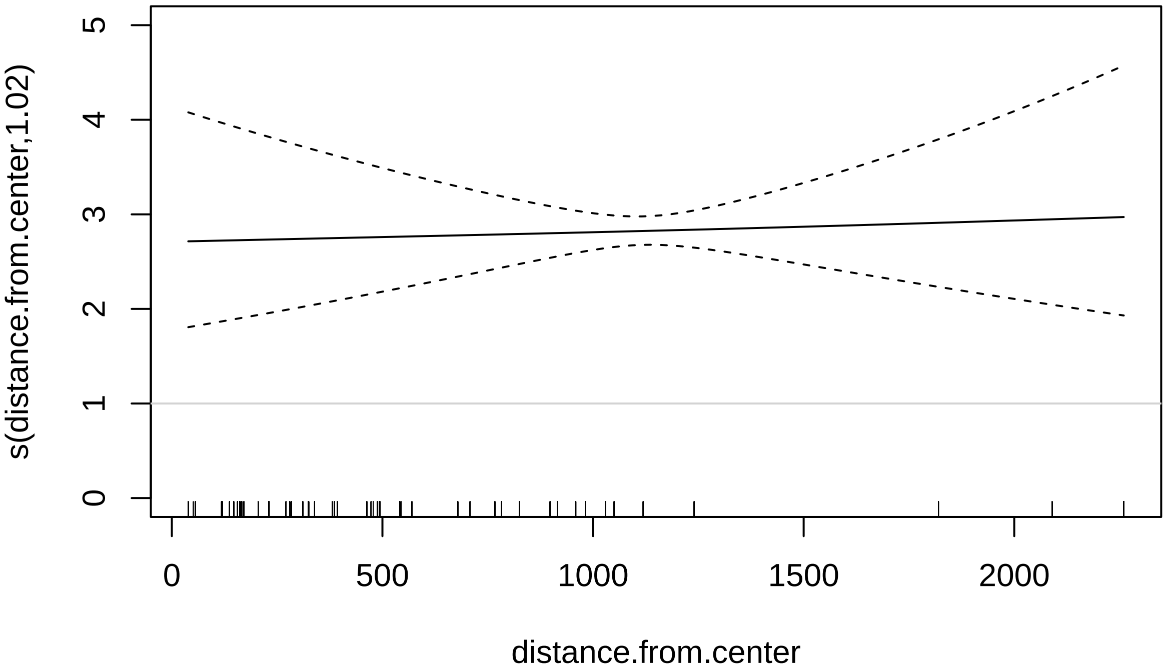

11.1.2 Fitted values

```
## Losone
d.ovitraps.temporal.Losone.new$fitted_gamm.eggs <-
  fitted(gamm.eggs.Losone.continuous)
## Ascona
d.ovitraps.temporal.Ascona.new$fitted_gamm.eggs <-
  fitted(gamm.eggs.Ascona.continuous)
##
d.ovitraps.fitted.new <- rbind(d.ovitraps.temporal.Losone.new,
                               d.ovitraps.temporal.Ascona.new)
##
## we include distance.from.center as percentiles for better visualisation
d.ovitraps.fitted.new <- d.ovitraps.fitted.new %>%
  mutate(percentile.distance.from.center = percent_rank(distance.from.center))
##
ggplot(data = d.ovitraps.fitted.new,
       mapping = aes(y = fitted_gamm.eggs,
                     x = yday,
                     group = unique.ID,
                     colour = percentile.distance.from.center)) +
  geom_vline(xintercept = yday("2025-06-17"), linetype = "dashed", colour = "grey") +
  geom_hline(yintercept = 0) +
  geom_point(alpha = 0.2) +
  geom_line(alpha = 0.2) +
  scale_y_sqrt() +
  facet_grid(Year.fac ~ municipality.fac) +
  theme(legend.position = "bottom")
```

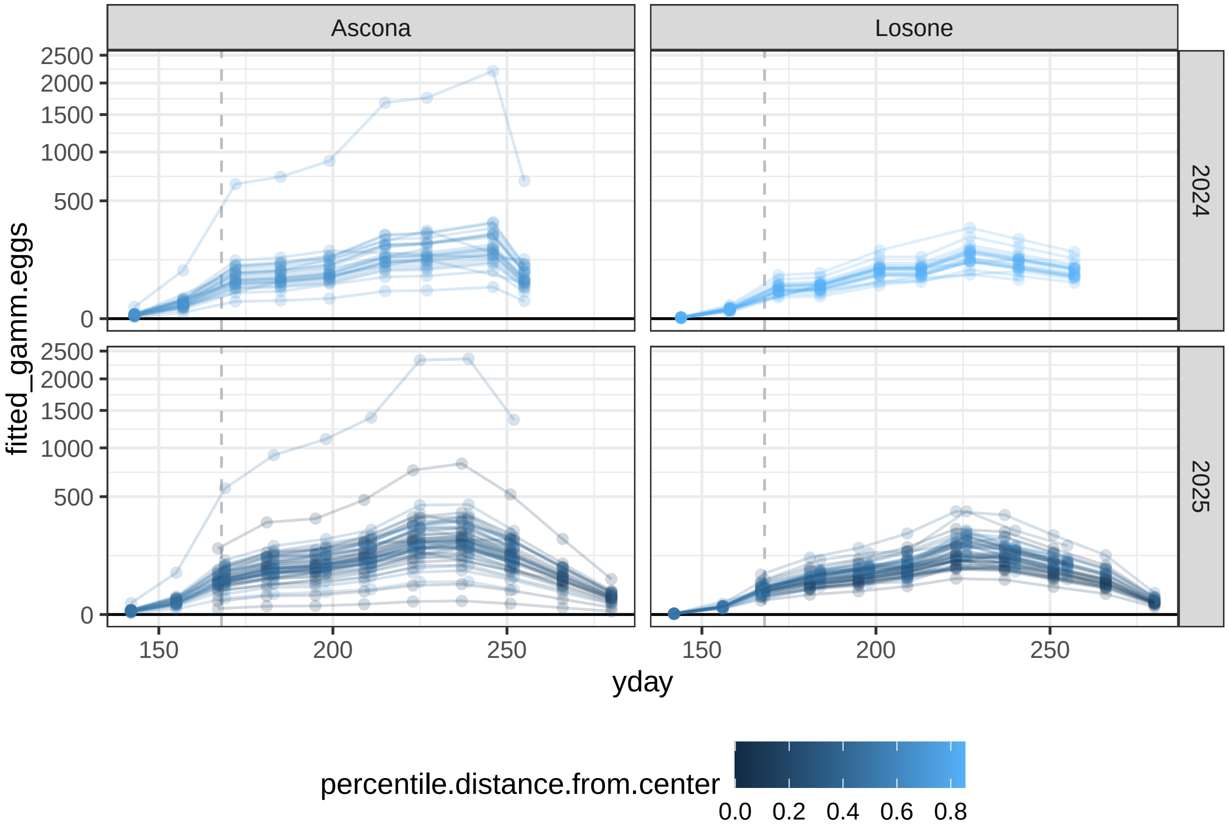

A pattern is not clear from this plot.

11.2 Number of eggs vs distance from center interaction with yday

We now model *total.albopictus.egg* as a function of *yday* and *distance.from.center*, including an interaction between the two variables. We fit separate models for Losone and Ascona.

```
gamm.eggs.Losone.te <- gamV(total.albopictus.egg ~
                             te(yday, distance.from.center,
                                by = Year.fac
                             ) +
                             Year.fac +
                             offset(log(Activation.time)) +
                             s(unique.ID, bs = "re"),
                           family = "nb",
                           data = d.ovitraps.temporal.Losone.new)
summary(gamm.eggs.Losone.te)
```

```
Family: Negative Binomial(0.59)
Link function: log

Formula:
total.albopictus.egg ~ te(yday, distance.from.center, by = Year.fac) +
    Year.fac + offset(log(Activation.time)) + s(unique.ID, bs = "re")

Parametric coefficients:
                    Estimate      Std. Error  z value Pr(>|z|)
```

```
(Intercept)  -0.253968932879 13.080415418401 -0.01942  0.98451
Year.fac2025  1.054949472498 13.080800109510  0.08065  0.93572

Approximate significance of smooth terms:
                                                     edf         Ref.df
te(yday,distance.from.center):Year.fac2024  3.53143696688  3.87682623063
te(yday,distance.from.center):Year.fac2025  9.20655888495 10.31721463338
s(unique.ID)                               30.16136917283 47.00000000000
                                              Chi.sq               p-value
te(yday,distance.from.center):Year.fac2024 255.63993 < 0.000000000000000222 ***
te(yday,distance.from.center):Year.fac2025 263.98653 < 0.000000000000000222 ***
s(unique.ID)                                85.98763 < 0.000000000000000222 ***
---
Signif. codes:  0 '***' 0.001 '**' 0.01 '*' 0.05 '.' 0.1 ' ' 1

Rank: 96/98
R-sq.(adj) =  0.394   Deviance explained =   48%
-REML = 2528.6  Scale est. = 1         n = 552
```

```r
##
##
gamm.eggs.Ascona.te <- gamV(total.albopictus.egg ~
                              te(yday, distance.from.center,
                                 by = Year.fac
                              ) +
                              Year.fac +
                              offset(log(Activation.time)) +
                              s(unique.ID, bs = "re"),
                            family = "nb",
                            data = d.ovitraps.temporal.Ascona.new)
summary(gamm.eggs.Ascona.te)
```

```
Family: Negative Binomial(0.806)
Link function: log

Formula:
total.albopictus.egg ~ te(yday, distance.from.center, by = Year.fac) +
    Year.fac + offset(log(Activation.time)) + s(unique.ID, bs = "re")

Parametric coefficients:
                    Estimate      Std. Error z value Pr(>|z|)
(Intercept)   0.840023016729 33.408331889770 0.02514  0.97994
Year.fac2025  0.505484201433 33.408218865479 0.01513  0.98793

Approximate significance of smooth terms:
                                                     edf         Ref.df
te(yday,distance.from.center):Year.fac2024  3.59702406296  3.88928533004
te(yday,distance.from.center):Year.fac2025 10.08297065860 11.38450039100
s(unique.ID)                               43.38122368273 51.00000000000
                                              Chi.sq               p-value
te(yday,distance.from.center):Year.fac2024 391.16459 < 0.000000000000000222 ***
te(yday,distance.from.center):Year.fac2025 435.08927 < 0.000000000000000222 ***
s(unique.ID)                               328.64452 < 0.000000000000000222 ***
---
```

```
Signif. codes:  0 '***' 0.001 '**' 0.01 '*' 0.05 '.' 0.1 ' ' 1

Rank: 100/102
R-sq.(adj) =   -1.9   Deviance explained = 52.3%
-REML = 3175.4  Scale est. = 1          n = 628
```

```r
plot.gam(gamm.eggs.Losone.te, pages = 1)
```

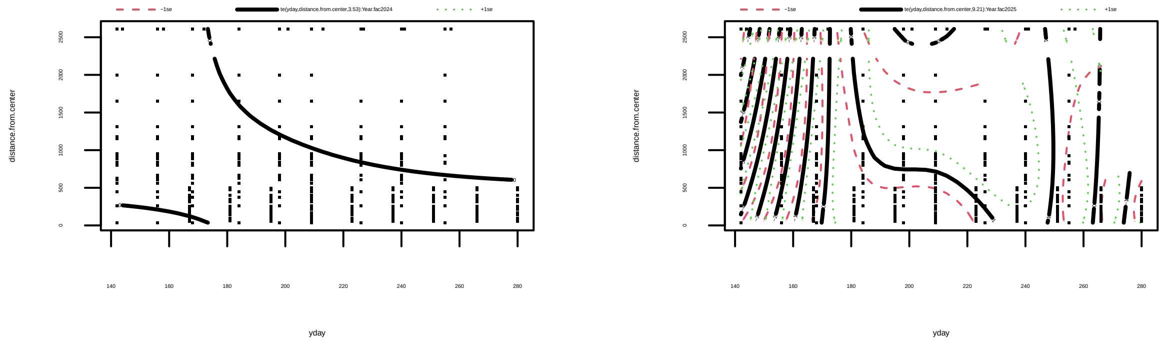

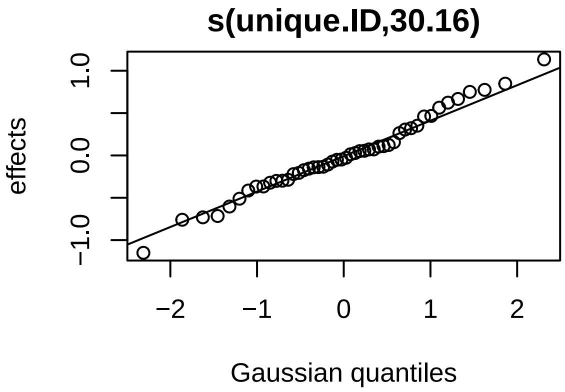


```r
plot.gamViz(gamm.eggs.Losone.te, select = 2)
```

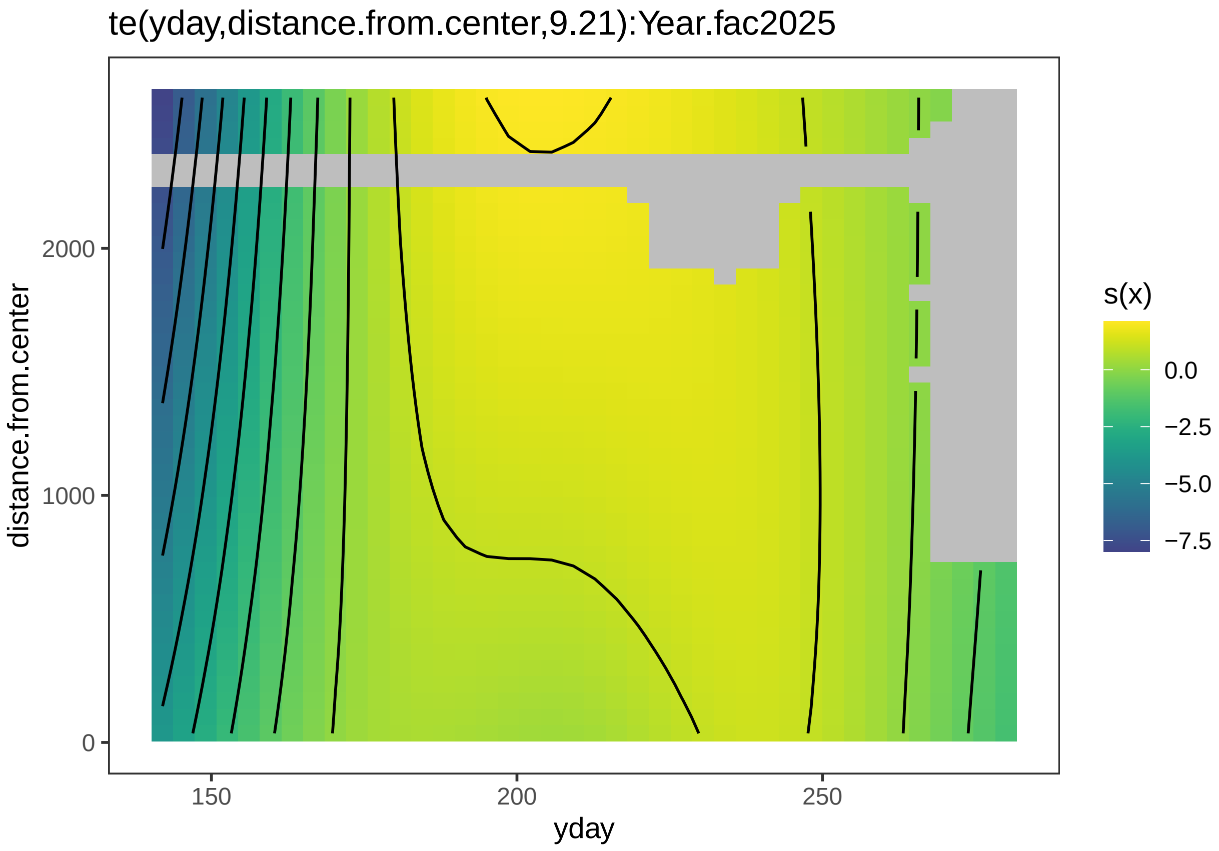


```
##
plot.gam(gamm.eggs.Ascona.te, pages = 1)
```

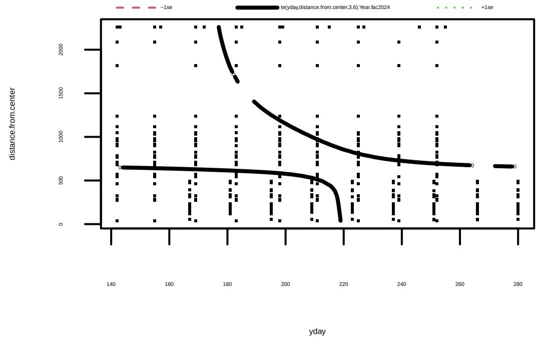

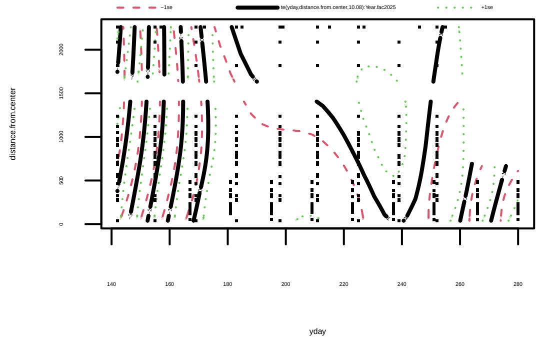

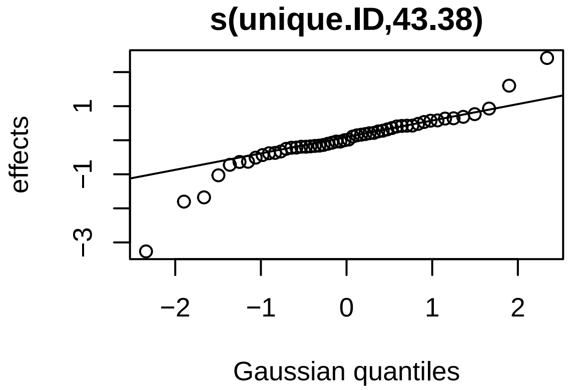


```
plot.gamViz(gamm.eggs.Ascona.te, select = 2)
```

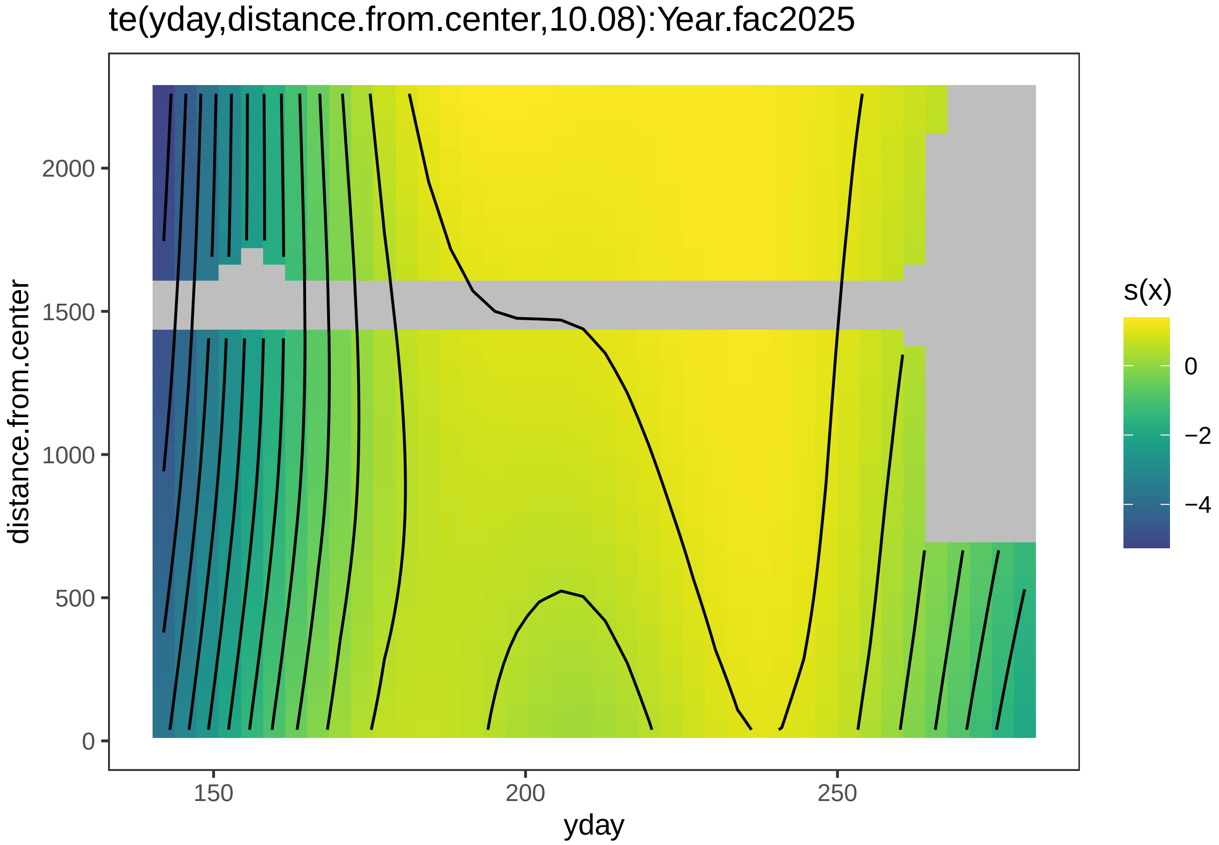

11.2.1 Fitted values

```
## Losone
d.ovitraps.temporal.Losone.new$fitted_gamm.eggs.te <-
  fitted(gamm.eggs.Losone.te)
## Ascona
d.ovitraps.temporal.Ascona.new$fitted_gamm.eggs.te <-
  fitted(gamm.eggs.Ascona.te)
##
d.ovitraps.fitted.new <- rbind(d.ovitraps.temporal.Losone.new,
                               d.ovitraps.temporal.Ascona.new)
##
ggplot(data = d.ovitraps.fitted.new,
       mapping = aes(y = fitted_gamm.eggs.te,
                     x = yday,
                     group = unique.ID,
                     colour = distance.from.center)) +
  geom_vline(xintercept = yday("2025-06-17"), linetype = "dashed", colour = "grey") +
  geom_hline(yintercept = 0) +
  geom_point(alpha = 0.2) +
  geom_line(alpha = 0.2) +
  scale_y_sqrt() +
  facet_grid(Year.fac ~ municipality.fac)
```

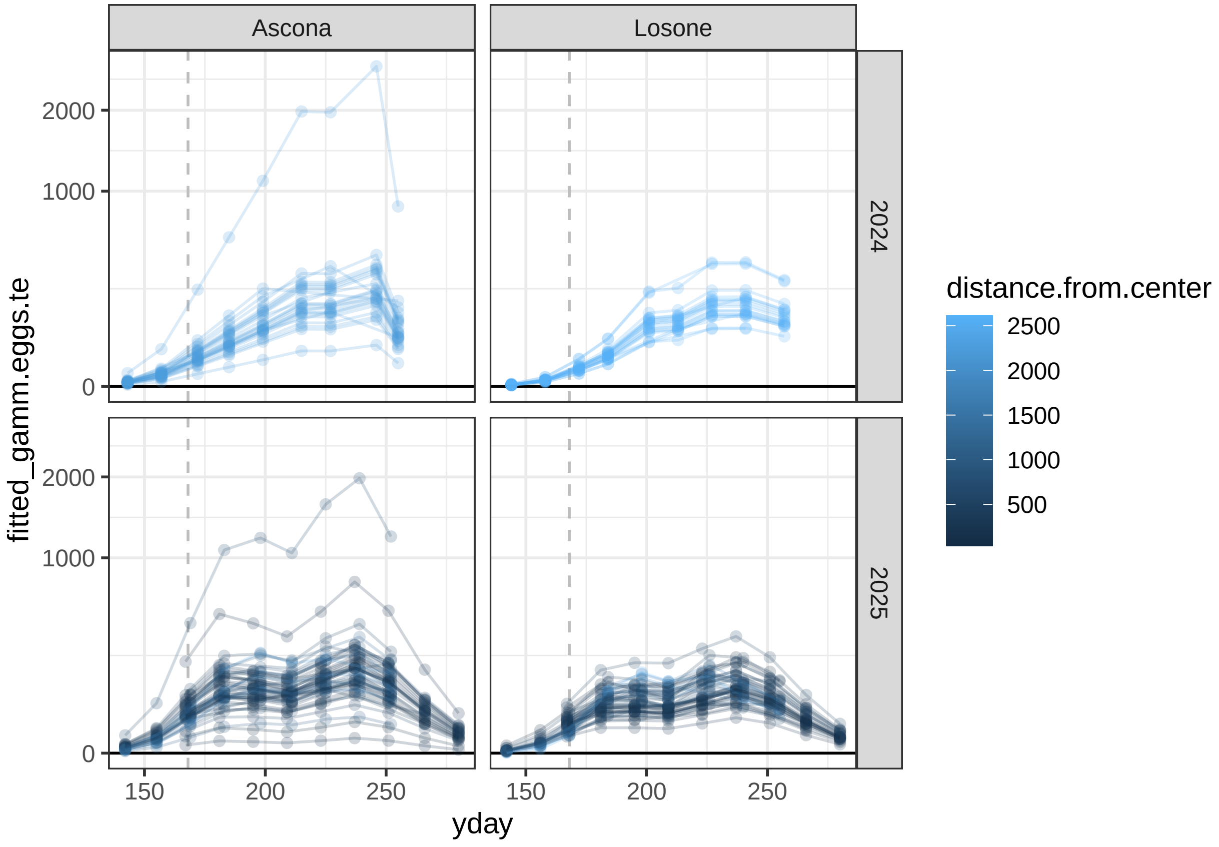

12 Session Information

```r
sessionInfo()
```

```
R version 4.5.1 (2025-06-13)
Platform: aarch64-apple-darwin20
Running under: macOS Tahoe 26.1

Matrix products: default
BLAS:   /Library/Frameworks/R.framework/Versions/4.5-arm64/Resources/lib/libRblas.0.dylib
LAPACK: /Library/Frameworks/R.framework/Versions/4.5-arm64/Resources/lib/libRlapack.dylib;  LAPACK vers

locale:
[1] en_US.UTF-8/en_US.UTF-8/en_US.UTF-8/C/en_US.UTF-8/en_US.UTF-8

time zone: Europe/Zurich
tzcode source: internal

attached base packages:
[1] grid      stats     graphics  grDevices utils     datasets  methods
[8] base

other attached packages:
 [1] sf_1.0-21       stringr_1.5.1   ggpubr_0.6.1    readxl_1.4.5
 [5] mgcViz_0.2.0    qgam_2.0.0      mgcv_1.9-3      nlme_3.1-168
 [9] lubridate_1.9.4 ggplot2_3.5.2   dplyr_1.1.4     groundhog_3.2.3
[13] knitr_1.50

loaded via a namespace (and not attached):
 [1] tidyselect_1.2.1   viridisLite_0.4.2  farver_2.1.2       viridis_0.6.5
 [5] fastmap_1.2.0      GGally_2.2.1       promises_1.3.3     digest_0.6.37
 [9] timechange_0.3.0   mime_0.13          lifecycle_1.0.4    magrittr_2.0.3
[13] compiler_4.5.1     rlang_1.1.6        tools_4.5.1        utf8_1.2.6
[17] yaml_2.3.10        ggsignif_0.6.4     labeling_0.4.3     classInt_0.4-11
[21] plyr_1.8.9         RColorBrewer_1.1-3 abind_1.4-8        KernSmooth_2.23-26
[25] gamm4_0.2-7        miniUI_0.1.2       withr_3.0.2        purrr_1.0.4
[29] xtable_1.8-4       e1071_1.7-16       scales_1.4.0       iterators_1.0.14
[33] MASS_7.3-65        isoband_0.2.7      tinytex_0.57       cli_3.6.5
[37] rmarkdown_2.29     reformulas_0.4.1   generics_0.1.4     rstudioapi_0.17.1
[41] minqa_1.2.8        DBI_1.2.3          proxy_0.4-27       splines_4.5.1
[45] parallel_4.5.1     cellranger_1.1.0   matrixStats_1.5.0  vctrs_0.6.5
[49] boot_1.3-31        Matrix_1.7-3       carData_3.0-5      car_3.1-3
[53] rstatix_0.7.2      Formula_1.2-5      foreach_1.5.2      tidyr_1.3.1
[57] units_0.8-7        glue_1.8.0         nloptr_2.2.1       ggstats_0.9.0
[61] codetools_0.2-20   cowplot_1.1.3      stringi_1.8.7      gtable_0.3.6
[65] later_1.4.2        lme4_1.1-37        tibble_3.3.0       pillar_1.10.2
[69] htmltools_0.5.8.1  R6_2.6.1           Rdpack_2.6.4       doParallel_1.0.17
[73] evaluate_1.0.4     shiny_1.11.0       lattice_0.22-7     rbibutils_2.3
[77] backports_1.5.0    broom_1.0.8        httpuv_1.6.16      class_7.3-23
[81] Rcpp_1.0.14        gridExtra_2.3      xfun_0.52          pkgconfig_2.0.3
```
