# Supplementary material for: Assessing the Sterile Insect Technique in Small, Non-Isolated Urban Areas: A Field Trial Against Aedes albopictus in Southern Switzerland
Source: Insects. 2026 Jul 31;17(8):793. doi: 10.3390/insects17080793 (PMC13513938; doi:10.3390/insects17080793)
Supplement: Supplementary file 1 [file insects-17-00793-s001.zip › File_S2.pdf]

Modelling: SIT Losone & Ascona

Hatched eggs

Author: Nisia Trisconi | Zurich Data Scientists
Reviewer: Dr. Matteo Tanadini | Zurich Data Scientists

December 5, 2025

Contents

1 Freeze Package versions **3**

2 Settings **3**

3 Getting data **4**

4 Aim and design **5**

5 Generalised Additive Mixed-Effects Model (GAMM) **5**
5.1 Visualising the data 6
5.2 Fitting the models 11
5.2.1 Preparing data set 12
5.2.2 Model for Losone 12
5.2.3 Model for Ascona 14
5.3 Plotting the smoothers 15
5.3.1 Losone 15
5.3.2 Ascona 18
5.4 Model selection 21
5.4.1 Losone 21
5.4.2 Ascona 24
5.5 Fitted values 27
5.6 Predicted values 28
5.6.1 Plotting the differences 33
5.7 Residual analysis 51

6 Spatial Generalised Additive Model (spatial GAM) **63**
6.1 Visualising the data 63
6.2 Fitting the models 68
6.3 Residual analysis 79

7 Methods **84**
7.1 Generalised Additive Mixed-Effects Model (GAMM) 84
7.2 Spatial Generalised Additive Model (spatial GAM) 84

8 Results **84**
8.1 Generalised Additive Mixed-Effects Model (GAMM) 84
8.2 Spatial Generalised Additive Model (spatial GAM) 86

9 Conclusions **88**

10 References **88**

11 Appendix **89**

11.1 hatched eggs vs distance from the centre of the treatment 89

11.1.1 Fitting the models 89

11.1.2 Fitted values 94

11.2 hatched eggs vs distance from centre interaction with yday 95

11.2.1 Fitted values 99

12 Session Information **100**

1 Freeze Package versions

```r
## (messages are omitted from this chunk)
##
library(groundhog)
pkgs <- c("dplyr",
          "ggplot2",
          "lubridate",
          "mgcViz",
          "readxl",
          "grid",
          "ggplotify",
          "ggpubr",
          "stringr",
          "sf") ## automatically loads mgcv
groundhog.library(pkgs, date = "2025-07-01")
```

2 Settings

Global settings:

```r
Sys.setenv(lang = "en_US")
theme_set(theme_bw())

if (!dir.exists("Prepared_data_and_models")) {
  dir.create("Prepared_data_and_models")
}
```

3 Getting data

This analysis concentrates on Losone and Ascona. Hatching rates remain constant over the years and across municipalities in the untreated zones, therefore we use these zones as control and don't include the data of previous years or different municipalities. This is done also because we don't have any information about hatching rates in Losone for the year 2024.

Moreover, we only keep functioning ovitraps.

We load the data set.

```
d.ovitraps.temporal.tmp <- readRDS(file = paste0("Prepared_data_and_models/",
                                                 "d.ovitraps.23.24.25_PreparedData.RDS")) %>%
  filter(status.fac == "F") %>%
  droplevels()
##
d.ovitraps.temporal <- d.ovitraps.temporal.tmp %>%
  filter(municipality.fac %in% c("Losone", "Ascona")) %>%
  droplevels()
```

Let's check which municipalities we have.

```
d.ovitraps.temporal %>%
  pull(municipality.fac) %>%
  unique()
```

```
[1] Ascona Losone
Levels: Ascona Losone
```

```
## makes sense
```

Overview of the data:

```
dim(d.ovitraps.temporal)
```

```
[1] 1180   40
```

```
head(d.ovitraps.temporal)[1:min(ncol(d.ovitraps.temporal), 30)]
```

```
# A tibble: 6 x 30
  OriginalSheet Activation.time total.eggs total.albopictus.egg
  <chr>                   <dbl>      <dbl>                <dbl>
1 1                          14          0                    0
2 1                          14          0                    0
3 1                          14          0                    0
4 1                          14          0                    0
5 1                          14          0                    0
6 1                          14          0                    0
# i 26 more variables: WHOLE.albopictus.egg <dbl>,
#   DESSICATED.albopictus.egg <dbl>, HATCH.albopictus.egg <dbl>,
#   WHOLE.albopictus.egg..AFTER.HATCHING.PROCEDURE. <dbl>,
#   DESSICATED.albopictus.egg..AFTER.HATCHING.PROCEDURE. <dbl>,
#   HATCH.albopictus.egg..AFTER.HATCHING.PROCEDURE. <dbl>,
#   Perc.egg.hatch.per.trap <dbl>, Year <dbl>, unique.ID <fct>, X.num <dbl>,
#   Y.num <dbl>, round.fac <fct>, Setting_date.date <date>, ...
```

```
str(d.ovitraps.temporal)
```

```
tibble [1,180 x 40] (S3: tbl_df/tbl/data.frame)
 $ OriginalSheet                                      : chr [1:1180] "1" "1" "1" "1" ...
```

```
 $ Activation.time                                    : num [1:1180] 14 14 14 14 14 14 14 14 14 14 ...
 $ total.eggs                                         : num [1:1180] 0 0 0 0 0 0 0 0 0 0 ...
 $ total.albopictus.egg                               : num [1:1180] 0 0 0 0 0 0 0 0 0 0 ...
 $ WHOLE.albopictus.egg                               : num [1:1180] 0 0 0 0 0 0 0 0 0 0 ...
 $ DESSICATED.albopictus.egg                          : num [1:1180] 0 0 0 0 0 0 0 0 0 0 ...
 $ HATCH.albopictus.egg                               : num [1:1180] 0 0 0 0 0 0 0 0 0 0 ...
 $ WHOLE.albopictus.egg..AFTER.HATCHING.PROCEDURE.     : num [1:1180] NA NA NA NA NA NA NA NA NA NA ...
 $ DESSICATED.albopictus.egg..AFTER.HATCHING.PROCEDURE.: num [1:1180] NA NA NA NA NA NA NA NA NA NA ...
 $ HATCH.albopictus.egg..AFTER.HATCHING.PROCEDURE.     : num [1:1180] NA NA NA NA NA NA NA NA NA NA ...
 $ Perc.egg.hatch.per.trap                            : num [1:1180] NA NA NA NA NA NA NA NA NA NA ...
 $ Year                                               : num [1:1180] 2024 2024 2024 2024 2024 ...
 $ unique.ID                                          : Factor w/ 100 levels "Ascona.1","Ascona.10",..
 $ X.num                                              : num [1:1180] 703175 703120 703644 702922 70336
 $ Y.num                                              : num [1:1180] 113224 112967 112960 112519 11251
 $ round.fac                                          : Factor w/ 20 levels "1","2","3","4",..: 12 12
 $ Setting_date.date                                  : Date[1:1180], format: "2024-05-08" "2024-05-08
 $ Sampling_date.date                                 : Date[1:1180], format: "2024-05-22" "2024-05-22
 $ municipality.ord                                   : Ord.factor w/ 2 levels "Ascona"<"Losone": 1 1
 $ municipality.fac                                   : Factor w/ 2 levels "Ascona","Losone": 1 1 1 1
 $ status.fac                                         : Factor w/ 1 level "F": 1 1 1 1 1 1 1 1 1 1 ...
 $ perc.manual                                        : num [1:1180] NA NA NA NA NA NA NA NA NA NA ...
 $ Year.fac                                           : Factor w/ 2 levels "2024","2025": 1 1 1 1 1 1
 $ hatched.albo.eggs.after.proc                       : num [1:1180] NA NA NA NA NA NA NA NA NA NA ...
 $ daily.albopictus.eggs                              : num [1:1180] 0 0 0 0 0 0 0 0 0 0 ...
 $ yday                                               : num [1:1180] 143 143 143 143 143 143 143 143 1
 $ non.hatched.albo.eggs.after.proc                   : num [1:1180] NA NA NA NA NA NA NA NA NA NA ...
 $ week                                               : num [1:1180] 21 21 21 21 21 21 21 21 21 21 ...
 $ unique.ID.Year                                     : Factor w/ 145 levels "Ascona.10b.2024",..: 21
 $ super.municipality.year                            : Factor w/ 4 levels "Ascona:2024",..: 1 1 1 1
 $ municipality.fac.spatial                           : Factor w/ 2 levels "Ascona","Losone": 1 1 1 1
 $ zona.SIT.fac                                       : Factor w/ 4 levels "dentro SIT","buffer",..: N
 $ zona.SIT.discrete.fac                              : Factor w/ 3 levels "within SIT","buffer",..: 3
 $ Year.ord                                           : Ord.factor w/ 2 levels "2024"<"2025": 1 1 1 1
 $ distance.from.center                               : num [1:1180] NA NA NA NA NA NA NA NA NA NA ...
 $ SIT.fac                                            : Factor w/ 2 levels "Pre SIT","SIT": 1 1 1 1 1
 $ zona.SIT.discrete.ord                              : Ord.factor w/ 3 levels "within SIT"<"buffer"<.
 $ super.zona.SIT.discrete.Year                       : Factor w/ 4 levels "outside SIT.2024",..: 1 1
 $ super.zona.SIT.discrete.Year.ord                   : Ord.factor w/ 4 levels "outside SIT.2024"<..:
 $ super.municipality.year.ord                        : Ord.factor w/ 4 levels "Ascona:2024"<..: 1 1 1
```

4 Aim and design

The aim of this analysis is to determine whether the release of sterilised males in a given area helps reducing the percentage of hatched eggs. With this purpose in mind, we are going to compare the treated, buffer and untreated zones of Losone and Ascona to check whether we see any difference.

In total, the data was collected on `40` different dates; Losone was sampled `26` times, whereas Ascona was sampled `27` times.

5 Generalised Additive Mixed-Effects Model (GAMM)

5.1 Visualising the data

The hatching rate is a quantity that remains quite constant over the years and across municipalities. For this reason, we compare hatching rates between the treatment zones by simply looking at the year 2025; the untreated zones will serve as control.

We begin by displaying hatching rates throughout the season. We group the observations based on the treatment zone (*zona.SIT.discrete.fac*).

Furthermore, observations from the same ovitrap are connected with a line to visualise trends over time. On top of these lines, we add a smoothed average grouped by the treatment zone, which allows us to check whether a treatment effect is present.

We also include a dashed line indicating when the treatment started, i.e., on date 2025-06-17.

```
## (warnings are omitted from this chunk)
##
d.ovitraps.temporal %>%
  filter(Year.fac == 2025) %>%
  ggplot(mapping = aes(x = Sampling_date.date,
                       y = Perc.egg.hatch.per.trap,
                       colour = zona.SIT.discrete.fac)) +
  geom_hline(yintercept = c(0, 100), colour = "grey") +
  geom_vline(xintercept = ymd("2025-06-17"), linetype = "dashed", colour = "grey") +
  geom_point(alpha = 0.2) +
  geom_line(mapping = aes(group = unique.ID), alpha = 0.2) +
  facet_wrap(~ municipality.fac, scales = "free_x") +
  ## robust smoother
  geom_smooth(alpha = 0.3, method = "loess",
              method.args = list(family = "symmetric")) +
  scale_colour_manual(values = c("blue", "green3", "red")) +
  coord_cartesian(ylim = c(0, 100)) +
  labs(colour = "Zone") +
  theme(legend.position = "bottom")
```

```
`geom_smooth()` using formula = 'y ~ x'
```

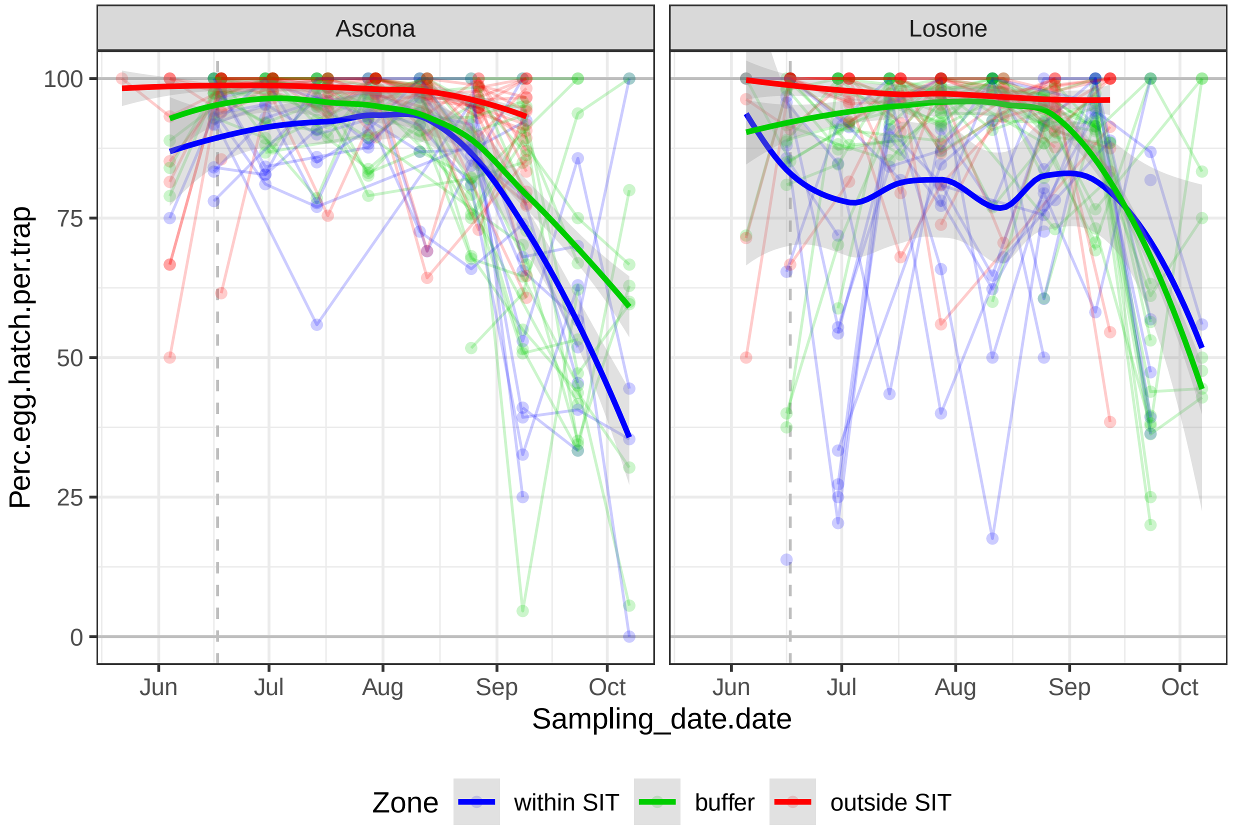

In general, we observe clear differences between the treated, buffer, and untreated zones. Specifically, hatching rates appear to be quite low in the treated zones, increase in the buffer areas, and reach very high levels in the untreated zones. The contrast between zones is more pronounced in Losone than in Ascona.

In Losone, hatching rates start off very high across all zones, but the treated zone drops rapidly after the beginning of the treatment, while the buffer and untreated zones remain at consistently high levels.

In Ascona, the treated zone starts with already lower hatching rated compared to the other zones, and then slightly increases over time. However, the treated zone has only a single observation before the treatment, which is relatively low and strongly pulls the smoother downward.

We show that hatching rates don't differ between years in the untreated zones.

```
## check that the year 2024 only has "outside SIT" values.
d.ovitraps.temporal.tmp %>%
  filter(Year.fac == 2024) %>%
  select(municipality.fac, zona.SIT.discrete.fac) %>%
  unique()
```

```
# A tibble: 17 x 2
   municipality.fac  zona.SIT.discrete.fac
   <fct>             <fct>
 1 Ascona            outside SIT
 2 Melide            outside SIT
 3 Vico Morcote      outside SIT
 4 Balerna           outside SIT
 5 Canobbio          outside SIT
 6 Caslano           outside SIT
 7 Chiasso           outside SIT
```

```
 8 Gordola           outside SIT
 9 Gravesano         outside SIT
10 Losone            outside SIT
11 Lugano            outside SIT
12 Manno             outside SIT
13 TaverneTorricella outside SIT
14 Bellinzona (GIU)  outside SIT
15 buffer area       outside SIT
16 Magliaso          outside SIT
17 Morcote           outside SIT
```

```
## makes sense
```

```
d.ovitraps.temporal.tmp %>%
  ## we remove Morcote, Vico Morcote and "buffer area" because they are
  ## treated or near treated regions
  filter( !(municipality.fac %in% c("Morcote", "Vico Morcote", "buffer area")) &
            !is.na(Perc.egg.hatch.per.trap) &
            zona.SIT.discrete.fac == "outside SIT") %>%
  droplevels() %>%
  ggplot(mapping = aes(x = yday,
                       y = Perc.egg.hatch.per.trap,
                       colour = Year.fac)) +
  geom_hline(yintercept = c(0, 100), colour = "grey") +
  geom_point(alpha = 0.2) +
  geom_line(mapping = aes(group = unique.ID), alpha = 0.2) +
  facet_wrap(~ municipality.fac, scales = "free_x") +
  ## robust smoother
  geom_smooth(alpha = 0.3, method = "loess",
            method.args = list(family = "symmetric")) +
  coord_cartesian(ylim = c(0, 100)) +
  labs(colour = "Year") +
  theme(legend.position = "bottom")
```

```
`geom_smooth()` using formula = 'y ~ x'
```

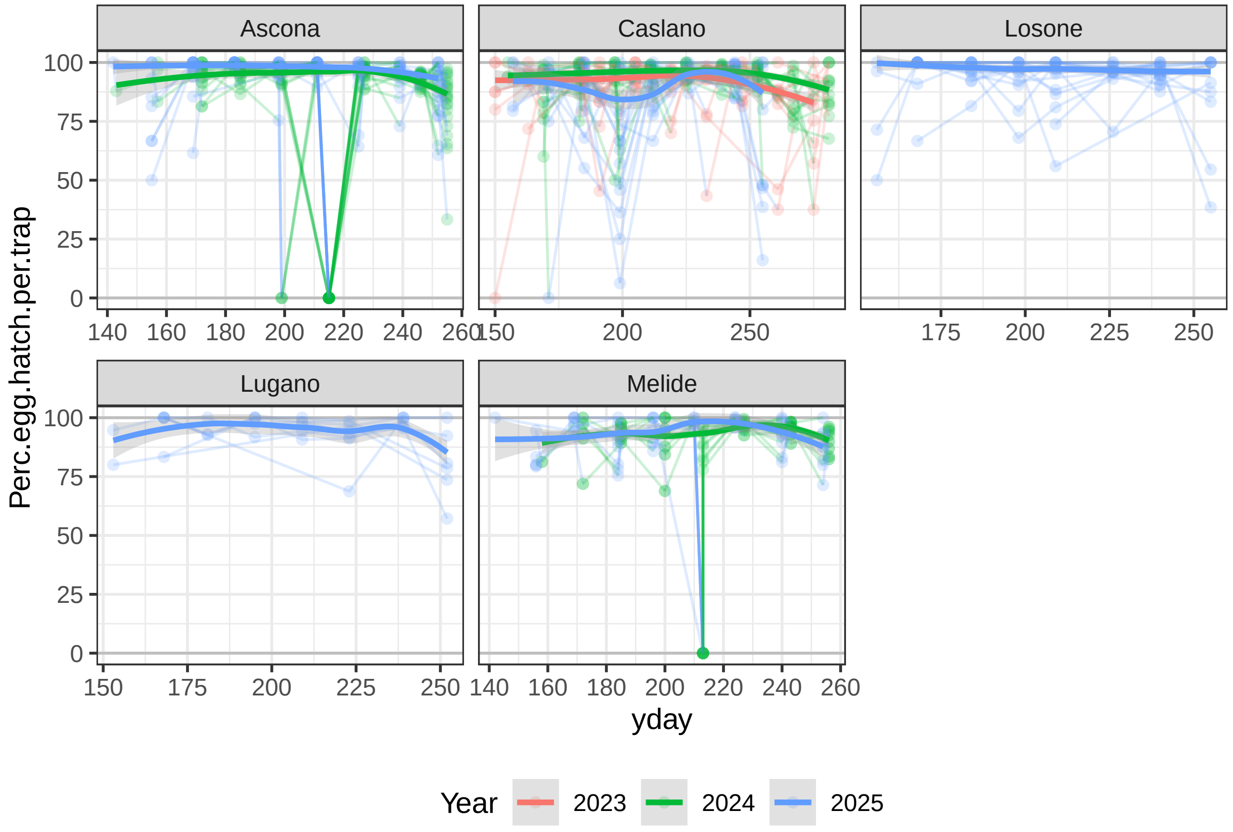

We observe that hatching rates are in general very high in all the untreated regions, and constant over the years.

Another interesting aspect to explore is the relationship between hatching rates and the distance from the release center. The idea behind this is to understand how large the treated area needs to be in order to observe an effect and, if an effect is detected, how far beyond the treated area it propagates.

We create a scatter plot of *Perc.egg.hatch.per.trap* against *distance.from.center*, grouping the points by treatment zone. A smoothed average is added to visualise potential trends.

```
## (warnings are omitted from this chunk)
##
gg.distance <- d.ovitraps.temporal %>%
  filter(Year.fac == 2025) %>%
  ggplot(mapping = aes(x = distance.from.center,
                       y = Perc.egg.hatch.per.trap,
  )) +
  geom_hline(yintercept = c(0, 100), colour = "grey") +
  geom_point(alpha = 0.2, mapping = aes(colour = zona.SIT.discrete.fac)) +
  facet_wrap(~ municipality.fac) +
  geom_smooth(alpha = 0.3, method = "loess",
              method.args = list(family = "symmetric")) +
  scale_colour_manual(values = c("blue", "green3", "red")) +
  coord_cartesian(ylim = c(0, 100)) +
  labs(colour = "Zone") +
  theme(legend.position = "bottom")
gg.distance
```

```
`geom_smooth()` using formula = 'y ~ x'
```

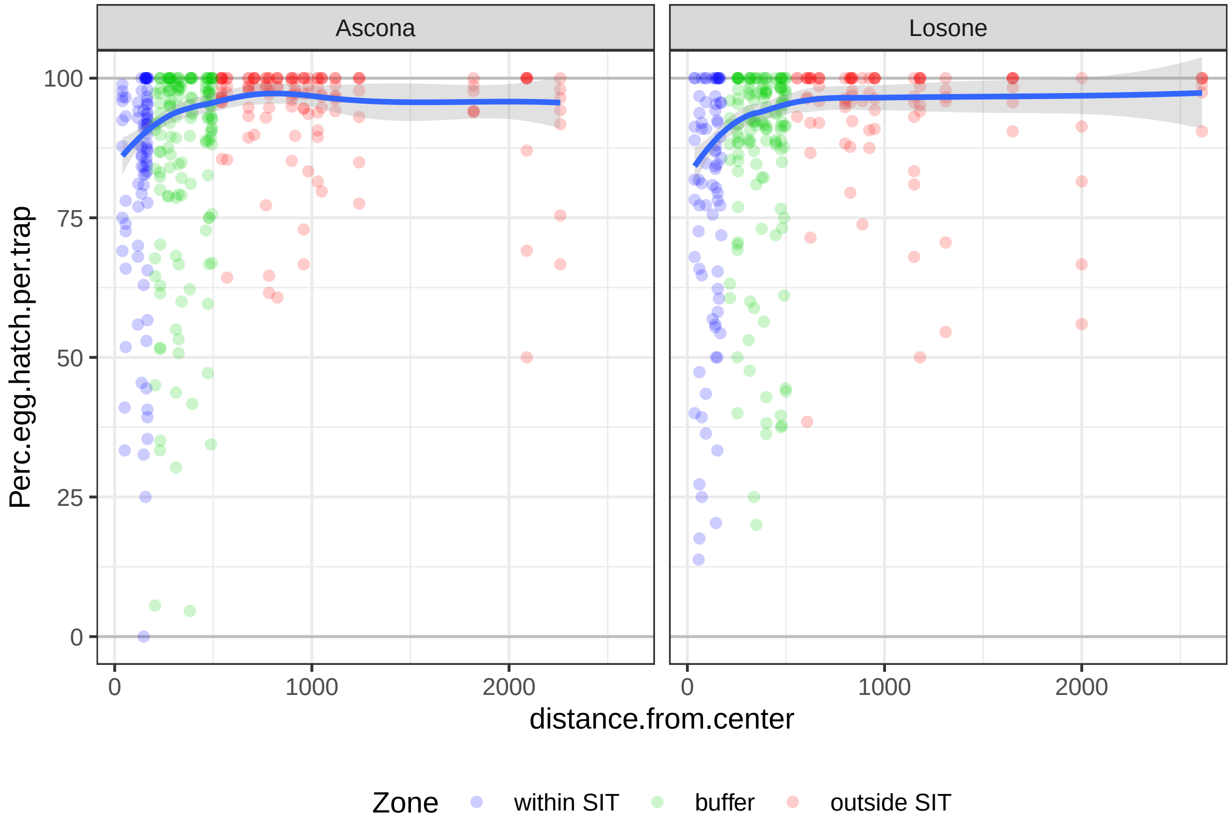


```r
##
## apply log
gg.distance +
  scale_x_log10()
```

```
`geom_smooth()` using formula = 'y ~ x'
```

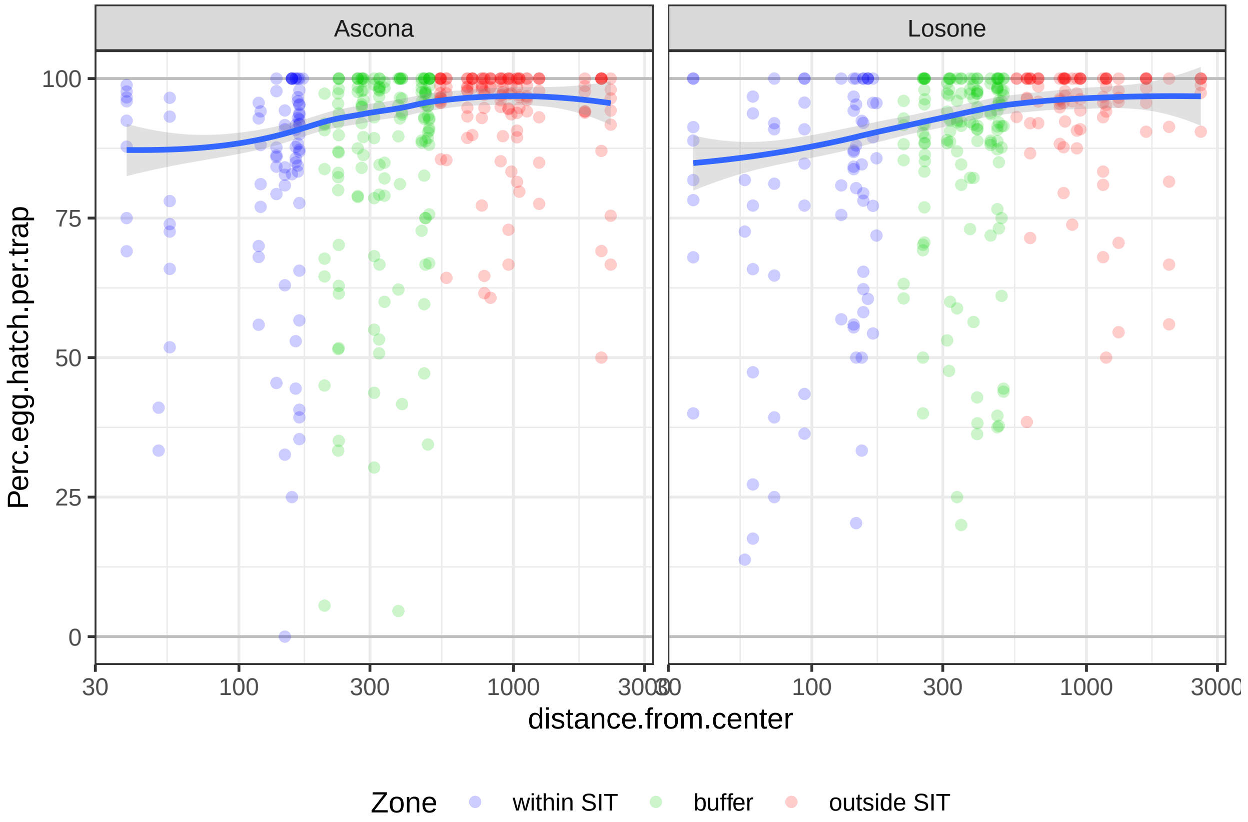

We see a strong initial positive relationship between the two variables up to the limit zone of the buffer area (i.e., 500m) for Losone. Outside of the buffer area, hatching rates seem to stabilise at very high values.

5.2 Fitting the models

We aim to model hatching rates of Aedes albopictus eggs (*Perc.egg.hatch.per.trap*) throughout the season, represented by the day of the year (*yday*). Separate models are fitted for the two municipalities, as their seasonal patterns might differ. This approach avoids the need for an overly complex model that would allow different trend shapes depending on the municipality.

The response variable is binomial, representing the counts of hatched versus non-hatched eggs (quantified by the variables *hatched.albo.eggs.after.proc* and *non.hatched.albo.eggs.after.proc*). To account for overdispersion, the quasi-binomial family is used for formal modelling.

As already mentioned, our goal is to assess whether hatching rates differ among treatment zones. The treatment zone may not only influence the magnitude of hatching rates but also modify the shape of their seasonal trend. To capture this, we treat *yday* as a numeric covariate whose effect is allowed to vary across treatment zones (*zona.SIT.discrete.fac*).

We use a Generalised Additive Model (GAM) to flexibly model seasonal dynamics without imposing a predefined functional form. This approach enhances model flexibility and captures complex temporal patterns, albeit at the cost of some interpretability.

Finally, to account for the lack of independence among observations from the same ovitrap, we include *unique.ID* (the unique ovitrap identifier) as a random effect. This adjustment helps to capture the variability specific to each ovitrap.

5.2.1 Preparing data set

We retain only the data from 2025 because hatching rates in the untreated zones (which serve as controls) remain constant across years. In addition, for Losone, data are available only for 2025. Including the 2024 data for Ascona would serve merely to improve coefficient estimation, but the 2025 dataset alone provides sufficient information.

```
d.hatching.tmp <- d.ovitraps.temporal %>%
  filter(Year.fac == 2025) %>%
  droplevels()
```

We check if there are any missing values for the relevant variables.

```
d.hatching <- d.hatching.tmp %>%
  select(Perc.egg.hatch.per.trap,
         hatched.albo.eggs.after.proc,
         non.hatched.albo.eggs.after.proc,

         municipality.fac,
         Activation.time,
         unique.ID,
         Sampling_date.date,
         yday,

         X.num, ## If we don't include them, the number of observations
         Y.num, ## dropped doesn't change. Thus we include them as we are going
         ## to need them in the spatial analysis.

         zona.SIT.discrete.fac,
         zona.SIT.discrete.ord,
         distance.from.center ## We'll use this variable in the appendix,
         ## but since it doesn't create any missing values,
         ## we include it now.
  ) %>%
  na.omit() %>%
  droplevels()
d.hatching.tmp %>%
  nrow()
```

```
[1] 808
```

```
nrow(d.ovitraps.temporal)
```

```
[1] 1180
```

```
## missing values are present
```

We rename *hatched.albo.eggs.after.proc* and *non.hatched.albo.eggs.after.proc* to have simpler and smaller variable names.

```
d.hatching <- d.hatching %>%
  rename(hatched = hatched.albo.eggs.after.proc,
         non.hatched = non.hatched.albo.eggs.after.proc)
```

5.2.2 Model for Losone

We start by modelling Losone.

```r
d.hatching.Losone <- d.hatching %>%
  filter(municipality.fac == "Losone")
```

We use the `gamV()` function instead of `gam()`, because it fits the `gam()` model and automatically converts it to a `gamViz` object, making visualisation easier.
The model's estimates are exactly the same.

```r
gamm.hatched.Losone <- gamV(cbind(hatched, non.hatched) ~

                              s(unique.ID, bs = "re") +

                              ## Fixed effects
                              zona.SIT.discrete.fac +
                              ## Losone doesn't have the year 2024
                              # Year.fac +

                              ## Interaction:
                              ## Treatment effect over seasonality
                              s(yday, by = zona.SIT.discrete.fac),

                            family = "quasibinomial",
                            method = "GCV.Cp", ## same as gam
                            data = d.hatching.Losone)
summary(gamm.hatched.Losone)
```

```
Family: quasibinomial
Link function: logit

Formula:
cbind(hatched, non.hatched) ~ s(unique.ID, bs = "re") + zona.SIT.discrete.fac +
    s(yday, by = zona.SIT.discrete.fac)

Parametric coefficients:
                                      Estimate     Std. Error t value
(Intercept)                        1.062992197067 0.169998346860 6.25296
zona.SIT.discrete.facbuffer        1.112277310123 0.219851504013 5.05922
zona.SIT.discrete.facoutside SIT 1.668463332558 0.244348827693 6.82820
                                          Pr(>|t|)
(Intercept)                        0.000000001612803 ***
zona.SIT.discrete.facbuffer        0.000000789343735 ***
zona.SIT.discrete.facoutside SIT 0.000000000058948 ***
---
Signif. codes:  0 '***' 0.001 '**' 0.01 '*' 0.05 '.' 0.1 ' ' 1

Approximate significance of smooth terms:
                                                 edf          Ref.df        F
s(unique.ID)                               23.50689028559 45.00000000000  1.23454
s(yday):zona.SIT.discrete.facwithin SIT     6.92824926487  7.86245735541  4.37449
s(yday):zona.SIT.discrete.facbuffer         7.60018900915  8.45162468592 26.41036
s(yday):zona.SIT.discrete.facoutside SIT  1.00010945612  1.00021423091  1.86067
                                                   p-value
s(unique.ID)                                   0.000030684 ***
s(yday):zona.SIT.discrete.facwithin SIT        0.000068759 ***
```

```
s(yday):zona.SIT.discrete.facbuffer      < 0.0000000000000000222 ***
s(yday):zona.SIT.discrete.facoutside SIT                0.17369
---
Signif. codes:  0 '***' 0.001 '**' 0.01 '*' 0.05 '.' 0.1 ' ' 1

R-sq.(adj) =  0.625   Deviance explained = 64.4%
GCV = 8.3038  Scale est. = 7.0173    n = 306
```

We extract the standard deviation associated with the random effect.

```r
gam.vcomp(gamm.hatched.Losone) %>%
  head(n = 1)
```

```
     s(unique.ID)
0.441668623210452
```

5.2.3 Model for Ascona

We now model Ascona.

```r
d.hatching.Ascona <- d.hatching %>%
  filter(municipality.fac == "Ascona")
```

```r
## with distance from center as discrete variable
gamm.hatched.Ascona <-  gamV(cbind(hatched, non.hatched) ~

                               s(unique.ID, bs = "re") +

                               ## Fixed effects
                               zona.SIT.discrete.fac +
                               ## Kept only 2025
                               # Year.fac +

                               ## Interaction:
                               ## Treatment effect over seasonality
                               s(yday, by = zona.SIT.discrete.fac),

                             family = "quasibinomial",
                             method = "GCV.Cp", ## same as gam
                             data = d.hatching.Ascona)
##
summary(gamm.hatched.Ascona)
```

```
Family: quasibinomial
Link function: logit

Formula:
cbind(hatched, non.hatched) ~ s(unique.ID, bs = "re") + zona.SIT.discrete.fac +
    s(yday, by = zona.SIT.discrete.fac)

Parametric coefficients:
                                      Estimate     Std. Error  t value
(Intercept)                        1.603755151547 0.154970898742 10.34875
zona.SIT.discrete.facbuffer        0.661214460903 0.210533233016  3.14067
```

```
zona.SIT.discrete.facoutside SIT 1.091870897092 0.885827181978  1.23260
                                              Pr(>|t|)
(Intercept)                      < 0.0000000000000000222 ***
zona.SIT.discrete.facbuffer                  0.0018492 **
zona.SIT.discrete.facoutside SIT             0.2186642
---
Signif. codes:  0 '***' 0.001 '**' 0.01 '*' 0.05 '.' 0.1 ' ' 1

Approximate significance of smooth terms:
                                                   edf         Ref.df        F
s(unique.ID)                            23.78710153466 49.00000000000  1.07175
s(yday):zona.SIT.discrete.facwithin SIT   8.31803495887  8.76759152056 16.22329
s(yday):zona.SIT.discrete.facbuffer       8.78705389765  8.96862850966 22.01714
s(yday):zona.SIT.discrete.facoutside SIT  6.85921468737  7.23800116619  4.89053
                                                       p-value
s(unique.ID)                                       0.000068020 ***
s(yday):zona.SIT.discrete.facwithin SIT  < 0.0000000000000000222 ***
s(yday):zona.SIT.discrete.facbuffer      < 0.0000000000000000222 ***
s(yday):zona.SIT.discrete.facoutside SIT           0.000019532 ***
---
Signif. codes:  0 '***' 0.001 '**' 0.01 '*' 0.05 '.' 0.1 ' ' 1

R-sq.(adj) =    0.7   Deviance explained = 72.3%
GCV = 8.4166  Scale est. = 7.657     n = 359
```

We extract the standard deviation associated with the random effect.

```r
gam.vcomp(gamm.hatched.Ascona) %>%
  head(n = 1)
```

```
     s(unique.ID)
0.384295191487195
```

5.3 Plotting the smoothers

We now plot the fitted smoothers.

5.3.1 Losone

Let's start with Losone.

```r
plot.gam(gamm.hatched.Losone,
         pages = 1,
         trans = plogis,
         ylim = c(0, 1))
```

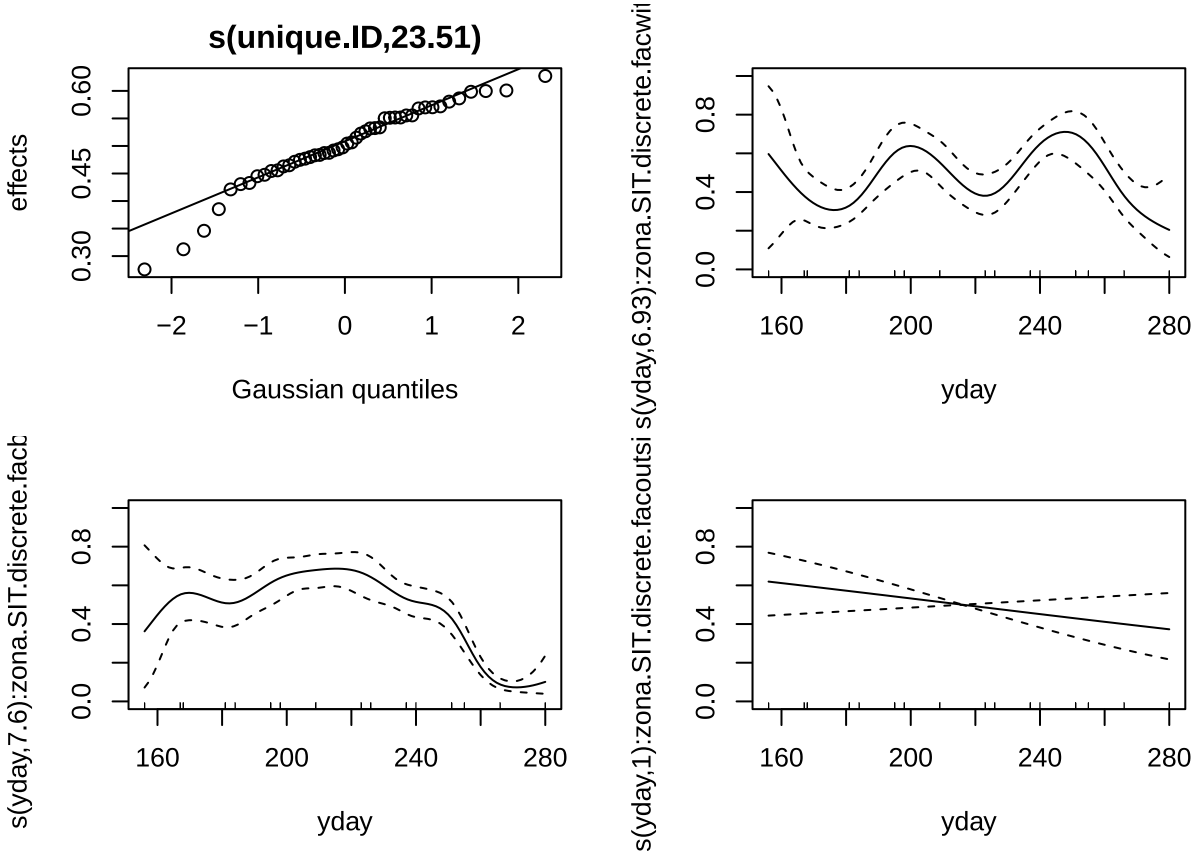

We add the corresponding shift.

```
## Within SIT
plot.gam(gamm.hatched.Losone,
         select = 2,
         shift = gamm.hatched.Losone$coefficients["(Intercept)"],
         trans = plogis,
         ylim = c(0, 1))
abline(h = 0:1)
```

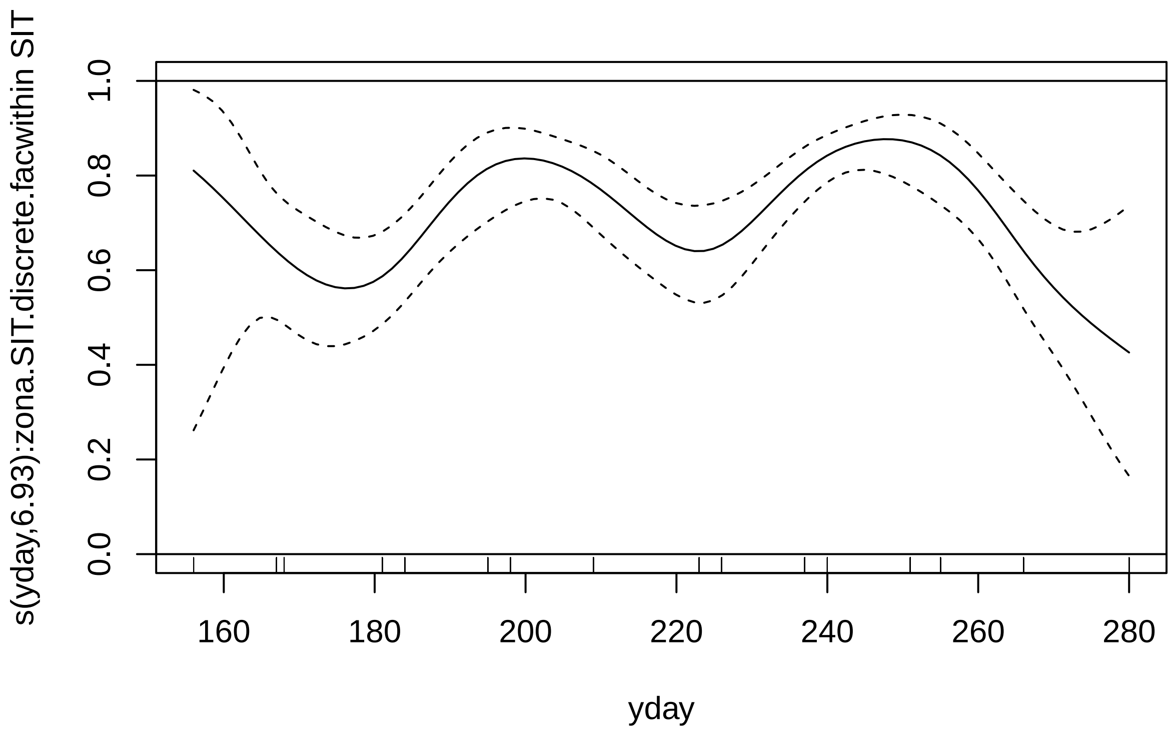


```r
##
## buffer
plot.gam(gamm.hatched.Losone,
         select = 3,
         shift = gamm.hatched.Losone$coefficients["(Intercept)"] +
           gamm.hatched.Losone$coefficients["zona.SIT.discrete.facbuffer"],
         trans = plogis,
         ylim = c(0, 1))
abline(h = 0:1)
```

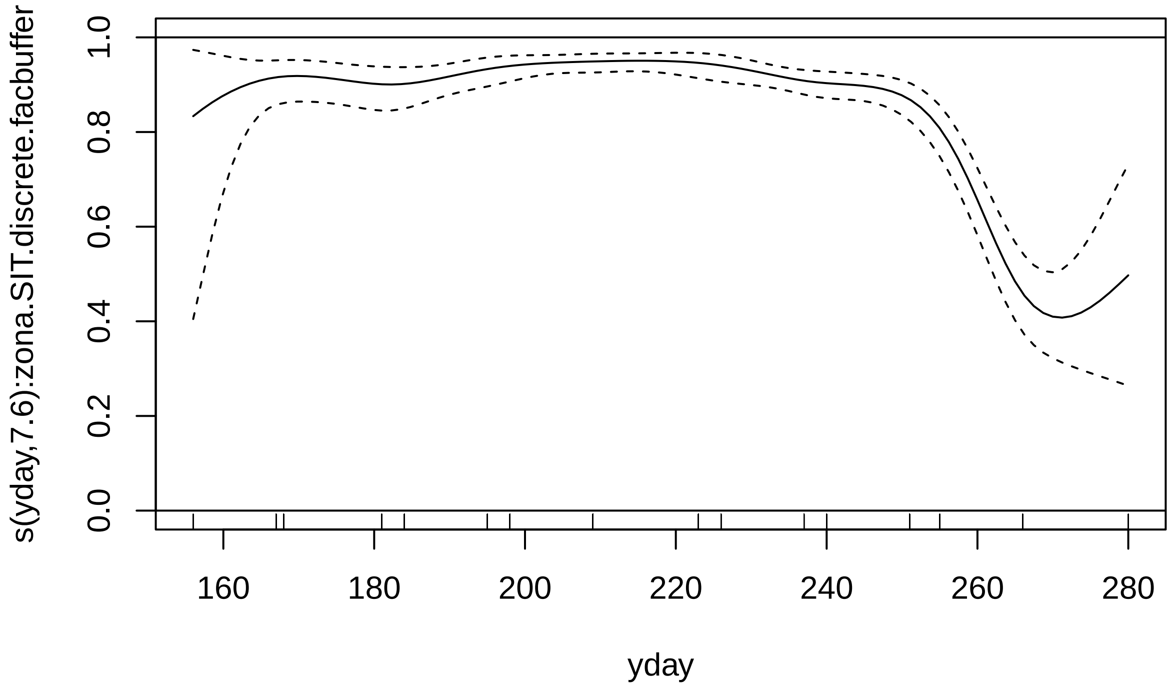

```
##
## outside SIT
plot.gam(gamm.hatched.Losone,
         select = 4,
         shift = gamm.hatched.Losone$coefficients["(Intercept)"] +
           gamm.hatched.Losone$coefficients["zona.SIT.discrete.facoutside SIT"],
         trans = plogis,
         ylim = c(0, 1))
abline(h = 0:1)
```

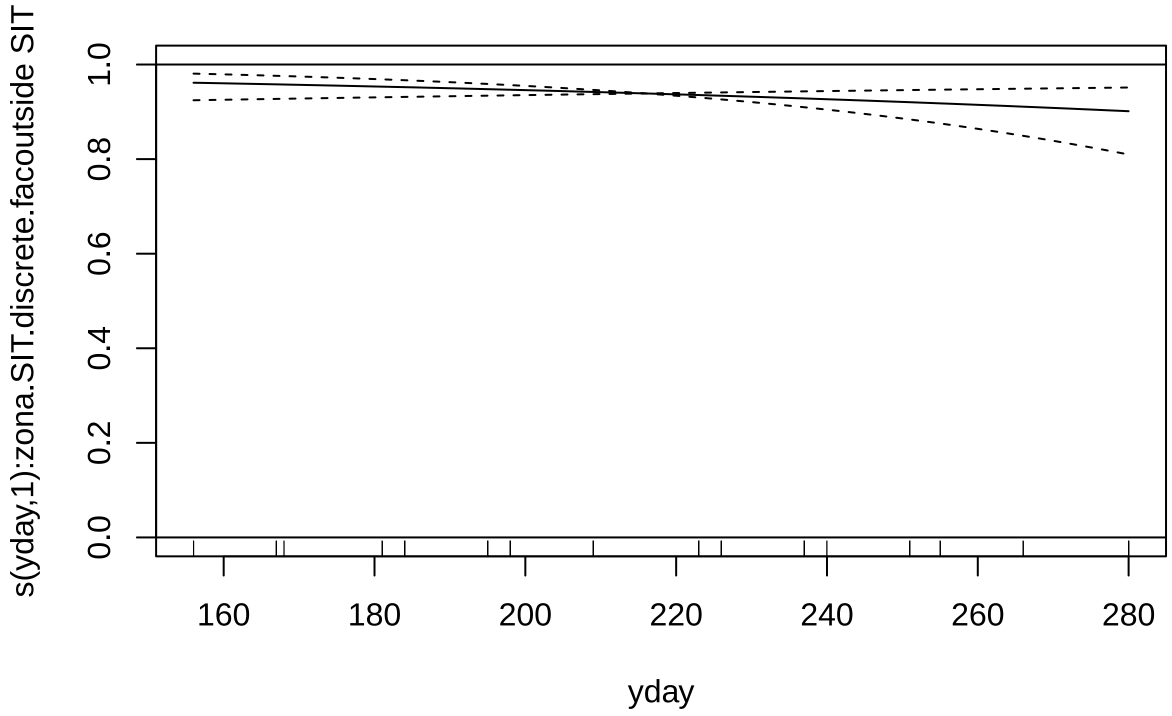

5.3.2 Ascona

We now look at Ascona.

```
plot.gam(gamm.hatched.Ascona,
         pages = 1,
         trans = plogis,
         ylim = c(0, 1))
```

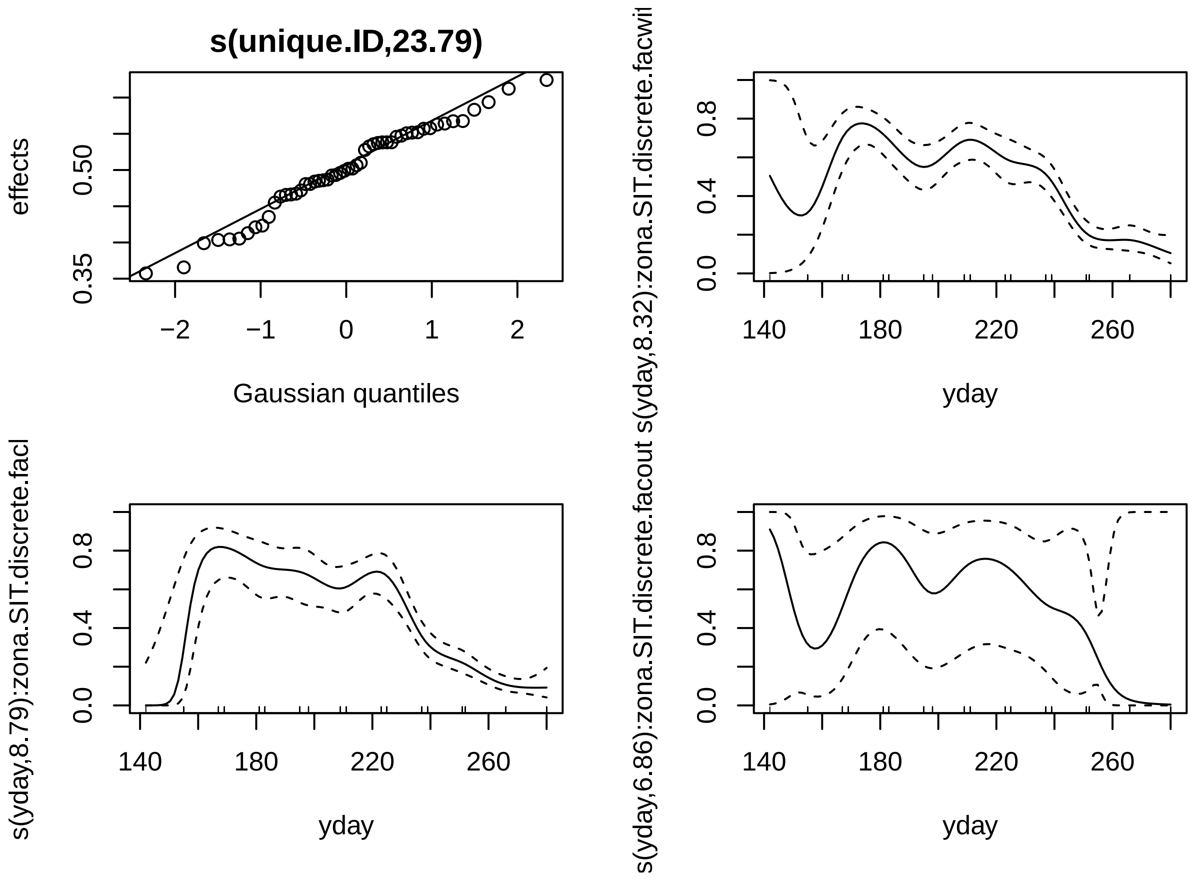

We add the corresponding shift.

```
## Within SIT
plot.gam(gamm.hatched.Ascona,
         select = 2,
         shift = gamm.hatched.Ascona$coefficients["(Intercept)"],
         trans = plogis,
         ylim = c(0, 1))
```

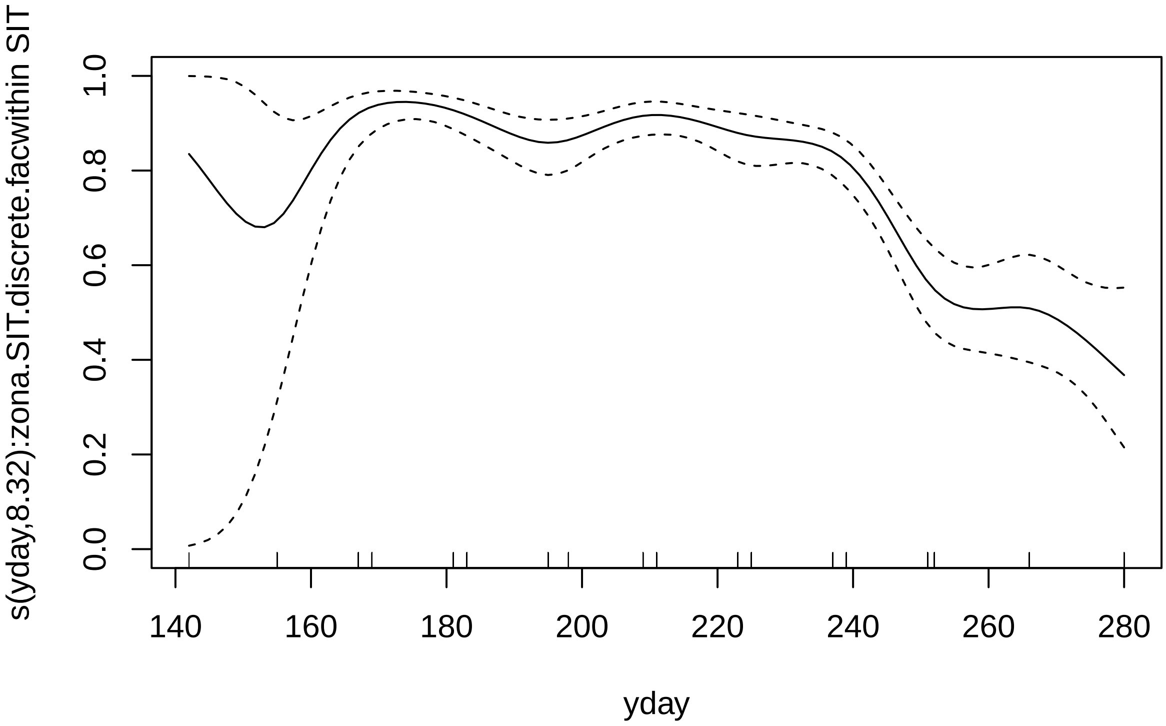


```r
##
## buffer
plot.gam(gamm.hatched.Ascona,
         select = 3,
         shift = gamm.hatched.Ascona$coefficients["(Intercept)"] +
           gamm.hatched.Ascona$coefficients["zona.SIT.discrete.facbuffer"],
         trans = plogis,
         ylim = c(0, 1))
```

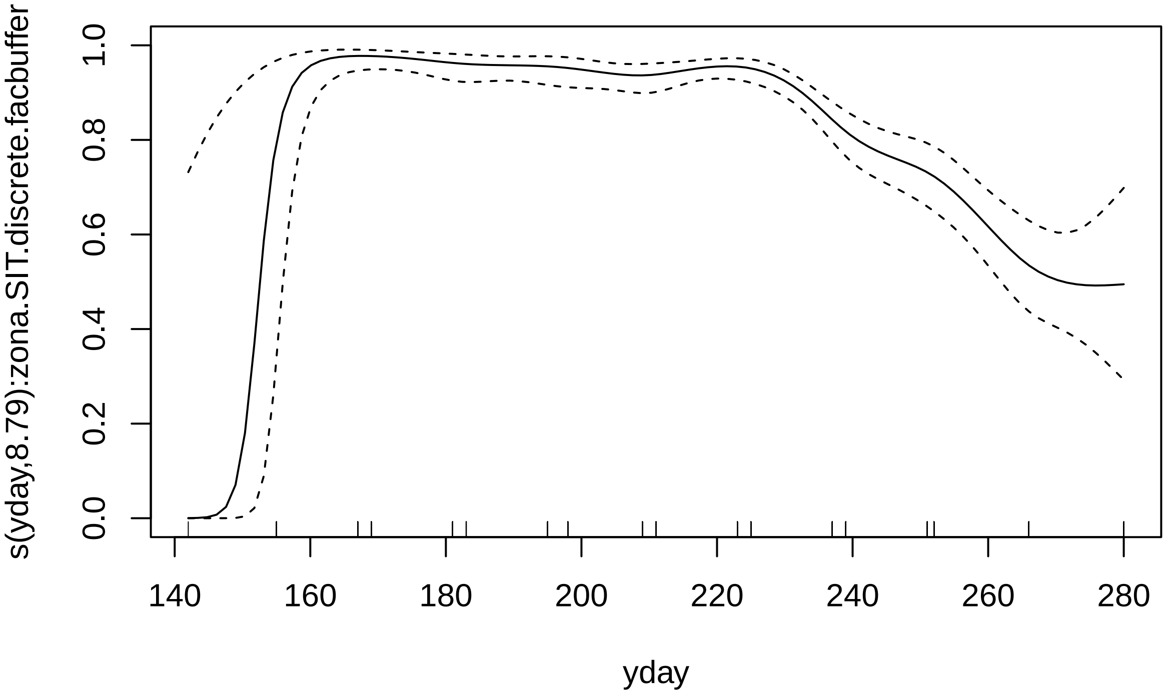

```r
##
## outside SIT
plot.gam(gamm.hatched.Ascona,
         select = 4,
         shift = gamm.hatched.Ascona$coefficients["(Intercept)"] +
           gamm.hatched.Ascona$coefficients["zona.SIT.discrete.facoutside SIT"],
         trans = plogis,
         ylim = c(0, 1))
```

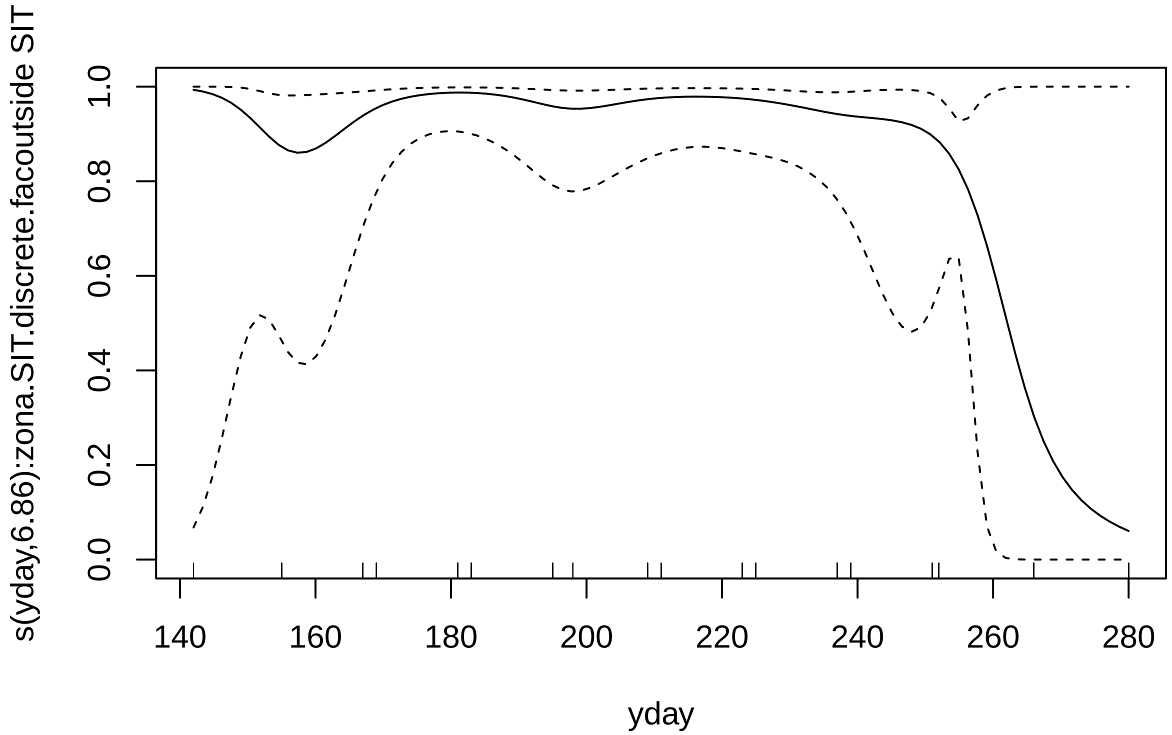


```r
## The CI towards the end of the plot is strange, but for the outside zone
## we only have data until yday 252, thus the CI is not reliably there.
```

5.4 Model selection

We fitted a model that allows the seasonal trend to vary across treatment zones by including zone-specific smoothers. We now test whether distinct smoothers are necessary.

5.4.1 Losone

For comparing the two models, we need to refit the first model using an ordered factor (*zona.SIT.discrete.ord*) instead of the classical one. This allows us to have nested model matrices and formally compare the two models.

```r
gamm.hatched.Losone_ord <- gamV(cbind(hatched, non.hatched)  ~

                                  s(unique.ID, bs = "re") +

                                  ## Fixed effect
                                  zona.SIT.discrete.fac +
```

```r
                                    s(yday) +

                                    ## Treatment effect over seasonality
                                    s(yday, by = zona.SIT.discrete.ord),


                                  family = "quasibinomial",
                                  method = "GCV.Cp", ## same as gam
                                  data = d.hatching.Losone)

##
summary(gamm.hatched.Losone_ord)
```

```

Family: quasibinomial
Link function: logit

Formula:
cbind(hatched, non.hatched) ~ s(unique.ID, bs = "re") + zona.SIT.discrete.fac +
    s(yday) + s(yday, by = zona.SIT.discrete.ord)

Parametric coefficients:
                                       Estimate     Std. Error t value
(Intercept)                      0.982919266839 0.155437542876 6.32356
zona.SIT.discrete.facbuffer      1.190858187082 0.203754746013 5.84457
zona.SIT.discrete.facoutside SIT 1.680803384778 0.232753053560 7.22140
                                           Pr(>|t|)
(Intercept)                      0.0000000010664145 ***
zona.SIT.discrete.facbuffer      0.0000000147249842 ***
zona.SIT.discrete.facoutside SIT 0.0000000000053417 ***
---
Signif. codes:  0 '***' 0.001 '**' 0.01 '*' 0.05 '.' 0.1 ' ' 1

Approximate significance of smooth terms:
                                              edf          Ref.df       F
s(unique.ID)                          21.67370006836 45.00000000000 1.08409
s(yday)                                7.50586385077  8.28926305177 3.72361
s(yday):zona.SIT.discrete.ordbuffer    5.15372291313  6.20934798587 8.80549
s(yday):zona.SIT.discrete.ordoutside SIT  1.00018950574  1.00036376216 8.53823
                                                  p-value
s(unique.ID)                                  0.000090246 ***
s(yday)                                        0.00029826 ***
s(yday):zona.SIT.discrete.ordbuffer      < 0.0000000000000000222 ***
s(yday):zona.SIT.discrete.ordoutside SIT           0.00377191 **
---
Signif. codes:  0 '***' 0.001 '**' 0.01 '*' 0.05 '.' 0.1 ' ' 1

R-sq.(adj) =  0.601   Deviance explained = 62.3%
GCV = 8.5512  Scale est. = 7.1742    n = 306
```

```r
##
gamm.hatched.Losone_one.smooth <- gamV(cbind(hatched, non.hatched)  ~

                                         s(unique.ID, bs = "re") +
```

```
                                          ## Fixed effect
                                          zona.SIT.discrete.fac +

                                          s(yday),# +

                                        ## Treatment effect over seasonality
                                        # s(yday, by = zona.SIT.discrete.ord),


                                        family = "quasibinomial",
                                        method = "GCV.Cp", ## same as gam
                                        data = d.hatching.Losone)
##
summary(gamm.hatched.Losone_one.smooth)
```

```

Family: quasibinomial
Link function: logit

Formula:
cbind(hatched, non.hatched) ~ s(unique.ID, bs = "re") + zona.SIT.discrete.fac +
    s(yday)

Parametric coefficients:
                                       Estimate     Std. Error t value
(Intercept)                       1.037992915422 0.148531321975 6.98838
zona.SIT.discrete.facbuffer       1.007825773562 0.186415053377 5.40635
zona.SIT.discrete.facoutside SIT 1.445448120910 0.227262205173 6.36027
                                          Pr(>|t|)
(Intercept)                       0.000000000020213 ***
zona.SIT.discrete.facbuffer       0.000000137525163 ***
zona.SIT.discrete.facoutside SIT 0.000000000813641 ***
---
Signif. codes:  0 '***' 0.001 '**' 0.01 '*' 0.05 '.' 0.1 ' ' 1

Approximate significance of smooth terms:
                        edf        Ref.df        F               p-value
s(unique.ID) 14.45917618690 45.0000000000  0.52716              0.014705 *
s(yday)       7.53686557096  8.4069909147 17.25682 < 0.0000000000000002 ***
---
Signif. codes:  0 '***' 0.001 '**' 0.01 '*' 0.05 '.' 0.1 ' ' 1

R-sq.(adj) =  0.469   Deviance explained = 51.3%
GCV = 10.021  Scale est. = 9.6238    n = 306
```

We check whether the two models are nested. If they are, then we can test whether the most complicated one is necessary, i.e., if the difference of the two models is statistically significant.

```
## Extract model matrices
m.gamm.hatched.Losone_one.smooth <- model.matrix(gamm.hatched.Losone_one.smooth)
m.gamm.hatched.Losone_ord <- model.matrix(gamm.hatched.Losone_ord)
dim(m.gamm.hatched.Losone_one.smooth)
```

```
[1] 306  60
```

```r
dim(m.gamm.hatched.Losone_ord)
```

```
[1] 306  78
```

```r
##
n1 <- ncol(m.gamm.hatched.Losone_one.smooth)
## check that sum is zero
sum(matrix( m.gamm.hatched.Losone_one.smooth[, 1:n1] - m.gamm.hatched.Losone_ord[, 1:n1] ) != 0)
```

```
[1] 0
```

The two model matrices are nested, therefore we can now compare the two models with the Chi-square test.

```r
chisq.test.Losone <- anova.gam(gamm.hatched.Losone_one.smooth,
                               gamm.hatched.Losone_ord,
                               test = "Chisq") %>%
  print()
```

```
Analysis of Deviance Table

Model 1: cbind(hatched, non.hatched) ~ s(unique.ID, bs = "re") + zona.SIT.discrete.fac +
    s(yday)
Model 2: cbind(hatched, non.hatched) ~ s(unique.ID, bs = "re") + zona.SIT.discrete.fac +
    s(yday) + s(yday, by = zona.SIT.discrete.ord)
       Resid. Df     Resid. Dev              Df       Deviance        Pr(>Chi)
1 271.3316861266 2585.790484334
2 255.9596470629 2002.143542802 15.37203906369 583.6469415323 0.0000000000549

1
2 ***
---
Signif. codes:  0 '***' 0.001 '**' 0.01 '*' 0.05 '.' 0.1 ' ' 1
```

The difference between the two models is statistically significant, which means that the additional flexibility, allowing different shapes for the smoother in each treatment zone, is necessary.

Please note that AIC and BIC cannot be calculated for models having a "quasi" family, thus we cannot double check the anova result with these additional tools.

5.4.2 Ascona

We do the same but for Ascona.

```r
gamm.hatched.Ascona_ord <- gamV(cbind(hatched, non.hatched)  ~

                                  s(unique.ID, bs = "re") +

                                  ## Fixed effect
                                  zona.SIT.discrete.fac +

                                  s(yday) +

                                  ## Treatment effect over seasonality
                                  s(yday, by = zona.SIT.discrete.ord),
```

```r
                                family = "quasibinomial",
                                method = "GCV.Cp", ## same as gam
                                data = d.hatching.Ascona)

##
summary(gamm.hatched.Ascona_ord)
```

```

Family: quasibinomial
Link function: logit

Formula:
cbind(hatched, non.hatched) ~ s(unique.ID, bs = "re") + zona.SIT.discrete.fac +
    s(yday) + s(yday, by = zona.SIT.discrete.ord)

Parametric coefficients:
                                       Estimate     Std. Error  t value
(Intercept)                        1.642400507255 0.150468181105 10.91527
zona.SIT.discrete.facbuffer        0.626123112727 0.199950585363  3.13139
zona.SIT.discrete.facoutside SIT 1.290949909277 0.207662273744  6.21658
                                               Pr(>|t|)
(Intercept)                        < 0.000000000000000222 ***
zona.SIT.discrete.facbuffer                     0.0018985 **
zona.SIT.discrete.facoutside SIT        0.0000000015648 ***
---
Signif. codes:  0 '***' 0.001 '**' 0.01 '*' 0.05 '.' 0.1 ' ' 1

Approximate significance of smooth terms:
                                                   edf         Ref.df        F
s(unique.ID)                              22.70075168684 49.00000000000  0.95268
s(yday)                                    7.58245320893  8.41069919163 20.43995
s(yday):zona.SIT.discrete.ordbuffer        1.00001903318  1.00003749144  3.64646
s(yday):zona.SIT.discrete.ordoutside SIT  1.00010554475  1.00020692014  2.46328
                                                       p-value
s(unique.ID)                                        0.00027136 ***
s(yday)                                   < 0.000000000000000222 ***
s(yday):zona.SIT.discrete.ordbuffer                 0.05707114 .
s(yday):zona.SIT.discrete.ordoutside SIT            0.11750111
---
Signif. codes:  0 '***' 0.001 '**' 0.01 '*' 0.05 '.' 0.1 ' ' 1

R-sq.(adj) =  0.689   Deviance explained = 69.8%
GCV = 8.3429  Scale est. = 8.023     n = 359
```

```r
##
gamm.hatched.Ascona_one.smooth <- gamV(cbind(hatched, non.hatched)  ~

                                         s(unique.ID, bs = "re") +

                                         ## Fixed effect
                                         zona.SIT.discrete.fac +

                                         s(yday),# +

```

```
                                              ## Treatment effect over seasonality
                                              # s(yday, by = zona.SIT.discrete.ord),


                                              family = "quasibinomial",
                                              method = "GCV.Cp", ## same as gam
                                              data = d.hatching.Ascona)
summary(gamm.hatched.Ascona_one.smooth)
```

```

Family: quasibinomial
Link function: logit

Formula:
cbind(hatched, non.hatched) ~ s(unique.ID, bs = "re") + zona.SIT.discrete.fac +
    s(yday)

Parametric coefficients:
                                       Estimate     Std. Error  t value
(Intercept)                      1.652718031582 0.148047598698 11.16342
zona.SIT.discrete.facbuffer      0.462935808059 0.183615353563  2.52123
zona.SIT.discrete.facoutside SIT 1.365858593821 0.203120264520  6.72438
                                         Pr(>|t|)
(Intercept)                      < 0.000000000000000222 ***
zona.SIT.discrete.facbuffer                   0.01217 *
zona.SIT.discrete.facoutside SIT      0.000000000078913 ***
---
Signif. codes:  0 '***' 0.001 '**' 0.01 '*' 0.05 '.' 0.1 ' ' 1

Approximate significance of smooth terms:
                        edf         Ref.df        F                 p-value
s(unique.ID) 22.01078831686 49.00000000000  0.86449              0.00090512 ***
s(yday)       7.44292253643  8.31297744614 40.85495 < 0.000000000000000222 ***
---
Signif. codes:  0 '***' 0.001 '**' 0.01 '*' 0.05 '.' 0.1 ' ' 1

R-sq.(adj) =  0.682   Deviance explained = 68.6%
GCV = 8.5088  Scale est. = 8.4763    n = 359
```

We check whether the two models are nested. If they are, then we can test whether the most complicated one is necessary, i.e., if the difference of the two models is statistically significant.

```
## Extract model matrices
m.gamm.hatched.Ascona_one.smooth <- model.matrix(gamm.hatched.Ascona_one.smooth)
m.gamm.hatched.Ascona_ord <- model.matrix(gamm.hatched.Ascona_ord)
dim(m.gamm.hatched.Ascona_one.smooth)
```

```
[1] 359  64
```

```
dim(m.gamm.hatched.Ascona_ord)
```

```
[1] 359  82
```

```
##
n2 <- ncol(m.gamm.hatched.Ascona_one.smooth)
## check that sum is zero
```

```r
sum(matrix( m.gamm.hatched.Ascona_one.smooth[, 1:n2] - m.gamm.hatched.Ascona_ord[, 1:n2] ) != 0)
```

```
[1] 0
```

The two model matrices are nested, therefore we can now compare the two models with the Chi-square test.

```r
chisq.test.Ascona <- anova.gam(gamm.hatched.Ascona_one.smooth,
                               gamm.hatched.Ascona_ord,
                               test = "Chisq") %>%
  print()
```

```
Analysis of Deviance Table

Model 1: cbind(hatched, non.hatched) ~ s(unique.ID, bs = "re") + zona.SIT.discrete.fac +
    s(yday)
Model 2: cbind(hatched, non.hatched) ~ s(unique.ID, bs = "re") + zona.SIT.discrete.fac +
    s(yday) + s(yday, by = zona.SIT.discrete.ord)
       Resid. Df      Resid. Dev              Df        Deviance  Pr(>Chi)
1 314.9854385029 2527.333921661
2 312.1382116333 2435.313066805 2.847226869634 92.02085485659 0.0081602 **
---
Signif. codes:  0 '***' 0.001 '**' 0.01 '*' 0.05 '.' 0.1 ' ' 1
```

The difference between the two models is statistically significant, which means that the additional flexibility, allowing different shapes for the smoother in each treatment zone, is necessary.

Please note that AIC and BIC cannot be calculated for models having a "quasi" family, thus we cannot double check the anova result with these additional tools.

5.5 Fitted values

First, we begin by plotting the fitted values over time, creating a separate graph for each municipality. Observations from the same ovitrap will be connected by lines to visualise the trends and a vertical dashed line is included to show when the SIT releases started (i.e., 17.06.2025).

```r
## Losone
d.hatching.Losone$fitted_gamm.hatching <-
  fitted(gamm.hatched.Losone)
## Ascona
d.hatching.Ascona$fitted_gamm.hatching <-
  fitted(gamm.hatched.Ascona)
##
d.ovitraps.fitted <- rbind(d.hatching.Losone,
                           d.hatching.Ascona)
##
ggplot(data = d.ovitraps.fitted,
       mapping = aes(y = fitted_gamm.hatching,
                     x = yday,
                     group = unique.ID,
                     colour = zona.SIT.discrete.fac)) +
  geom_vline(xintercept = yday("2025-06-17"), linetype = "dashed", colour = "grey") +
  geom_hline(yintercept = 0) +
  geom_point(alpha = 0.1) +
  geom_line(alpha = 0.5) +
  scale_y_sqrt() +
  facet_grid(~ municipality.fac)
```

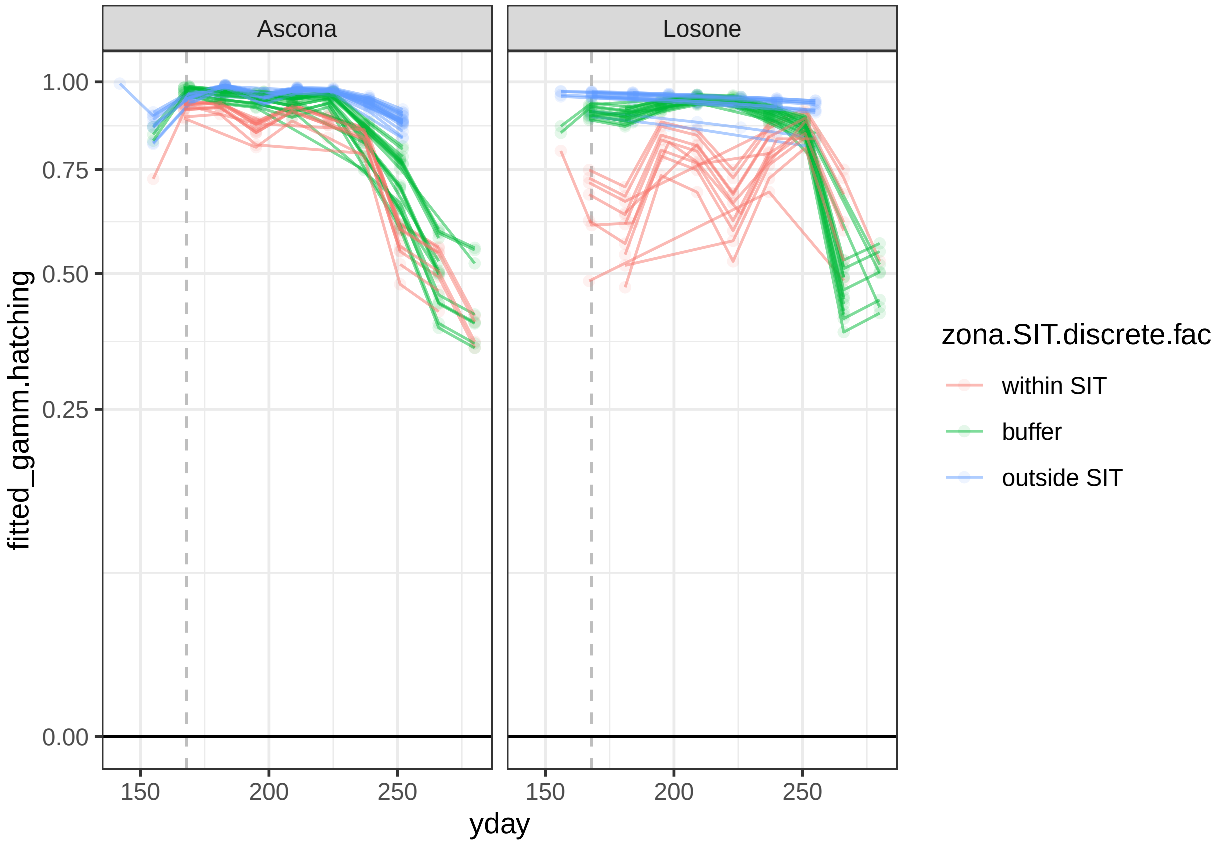

We observe that, in Losone, the fitted values for the level "within SIT" are below those of the other two zones. In Ascona, the differences among zones are less pronounced, but we still see that the "within SIT" zone has lower fitted values compared to the other zones.

Note that there is quite some variability among ovitraps in the treated zone for Losone.

5.6 Predicted values

We will now create the dataset for prediction. Our goal is to predict the hatching rates over the entire season, both at the ovitrap and population levels.

```
## (messages are omitted from this chunk)
##
formula(gamm.hatched.Losone)
```

```
cbind(hatched, non.hatched) ~ s(unique.ID, bs = "re") + zona.SIT.discrete.fac +
    s(yday, by = zona.SIT.discrete.fac)
```

```
##
## 1. We create a data set with unique.ID and zona.SIT.discrete.fac.
## We do it separately because not in all zona.SIT.discrete.fac all ovitraps are present.
## We keep municipality to then separate the data set based on this variable.
d.muni.ovitrap.short <- d.ovitraps.fitted %>%
  group_by(municipality.fac, zona.SIT.discrete.fac) %>%
  mutate(yday.min = min(yday, na.rm = TRUE),
         yday.max = max(yday, na.rm = TRUE)) %>%
  ungroup() %>%
  select(municipality.fac, unique.ID, zona.SIT.discrete.fac, yday.min, yday.max) %>%
```

```r
  unique()
##
head(d.muni.ovitrap.short)
```

```
# A tibble: 6 x 5
  municipality.fac unique.ID  zona.SIT.discrete.fac yday.min yday.max
  <fct>            <fct>      <fct>                    <dbl>    <dbl>
1 Losone           Losone.4b  outside SIT                156      255
2 Losone           Losone.5a  outside SIT                156      255
3 Losone           Losone.6b  outside SIT                156      255
4 Losone           Losone.8a  within SIT                 156      280
5 Losone           Losone.10b buffer                     156      280
6 Losone           Losone.13a buffer                     156      280
```

```r
nrow(d.muni.ovitrap.short)
```

```
[1] 100
```

```r
##
## 2. We create a data set with varying yday for each ovitrap ID
d.pred.gamm <- expand.grid(
  yday = seq(from = min(d.ovitraps.fitted$yday),
             to = max(d.ovitraps.fitted$yday),
             length.out = 100),
  unique.ID = d.muni.ovitrap.short$unique.ID)
##
## 3. We join the two data sets.
## Note that not all ovitraps exist in all municipalities.
## So, we can't simply use expand.grid()
d.pred.gamm_aug <- left_join(x = d.pred.gamm, y = d.muni.ovitrap.short) %>%
  ## we don't want to predict outside the range of available data
  filter(yday >= yday.min & yday <= yday.max)
str(d.pred.gamm_aug)
```

```
'data.frame':   8455 obs. of  6 variables:
 $ yday                 : num  157 159 160 162 163 ...
 $ unique.ID            : Factor w/ 100 levels "Ascona.1","Ascona.10",..: 90 90 90 90 90 90 90 90 90 90
 $ municipality.fac     : Factor w/ 2 levels "Ascona","Losone": 2 2 2 2 2 2 2 2 2 2 ...
 $ zona.SIT.discrete.fac: Factor w/ 3 levels "within SIT","buffer",..: 3 3 3 3 3 3 3 3 3 3 ...
 $ yday.min             : num  156 156 156 156 156 156 156 156 156 156 ...
 $ yday.max             : num  255 255 255 255 255 255 255 255 255 255 ...
 - attr(*, "out.attrs")=List of 2
  ..$ dim     : Named int [1:2] 100 100
  .. ..- attr(*, "names")= chr [1:2] "yday" "unique.ID"
  ..$ dimnames:List of 2
  .. ..$ yday     : chr [1:100] "yday=142.000000000000" "yday=143.393939393939" "yday=144.787878787879"
  .. ..$ unique.ID: chr [1:100] "unique.ID=Losone.4b" "unique.ID=Losone.5a" "unique.ID=Losone.6b" "uniq
```

We make the predictions on the newly created data set, at ovitrap and population level, and we plot the result.

```r
## prediction at ovitrap level
d.pred.gamm_aug.Losone <- d.pred.gamm_aug %>%
  filter(municipality.fac == "Losone") %>%
  droplevels()
d.pred.gamm_aug.Losone$predicted_gamm.hatched <- predict(
```

```r
  gamm.hatched.Losone,
  type = "response",
  newdata = d.pred.gamm_aug.Losone)
##
d.pred.gamm_aug.Ascona <- d.pred.gamm_aug %>%
  filter(municipality.fac == "Ascona") %>%
  droplevels()
d.pred.gamm_aug.Ascona$predicted_gamm.hatched <- predict(
  gamm.hatched.Ascona,
  type = "response",
  newdata = d.pred.gamm_aug.Ascona)
##
## prediction at population level
d.pred.gamm_aug.Losone$predicted_gamm.hatched.pop <- predict(
  gamm.hatched.Losone,
  newdata = d.pred.gamm_aug.Losone,
  type = "response",
  exclude = 's(unique.ID)')
##
d.pred.gamm_aug.Ascona$predicted_gamm.hatched.pop <- predict(
  gamm.hatched.Ascona,
  newdata = d.pred.gamm_aug.Ascona,
  type = "response",
  exclude = 's(unique.ID)')
##
## Join the two data sets
d.pred.gamm_aug <- rbind(d.pred.gamm_aug.Losone,
                         d.pred.gamm_aug.Ascona)
```

```r
gg.prediction.gamm.Losone <- ggplot(data = d.pred.gamm_aug.Losone,
                                    mapping = aes(y = predicted_gamm.hatched,
                                                  x = yday,
                                                  group = unique.ID)) +
  geom_vline(xintercept = yday("2025-06-17"), linetype = "dashed", colour = "grey") +
  geom_hline(yintercept = c(0, 1), colour = "grey") +
  geom_line(alpha = 0.2) +
  geom_line(mapping = aes(y = predicted_gamm.hatched.pop),
            colour = "red") +
  facet_wrap(~ zona.SIT.discrete.fac) +
  geom_rug(data = d.hatching.Losone, mapping = aes(x = yday),
           sides = "b",
           inherit.aes = FALSE,
           alpha = 0.5)
gg.prediction.gamm.Losone +
  labs(title = "Losone")
```

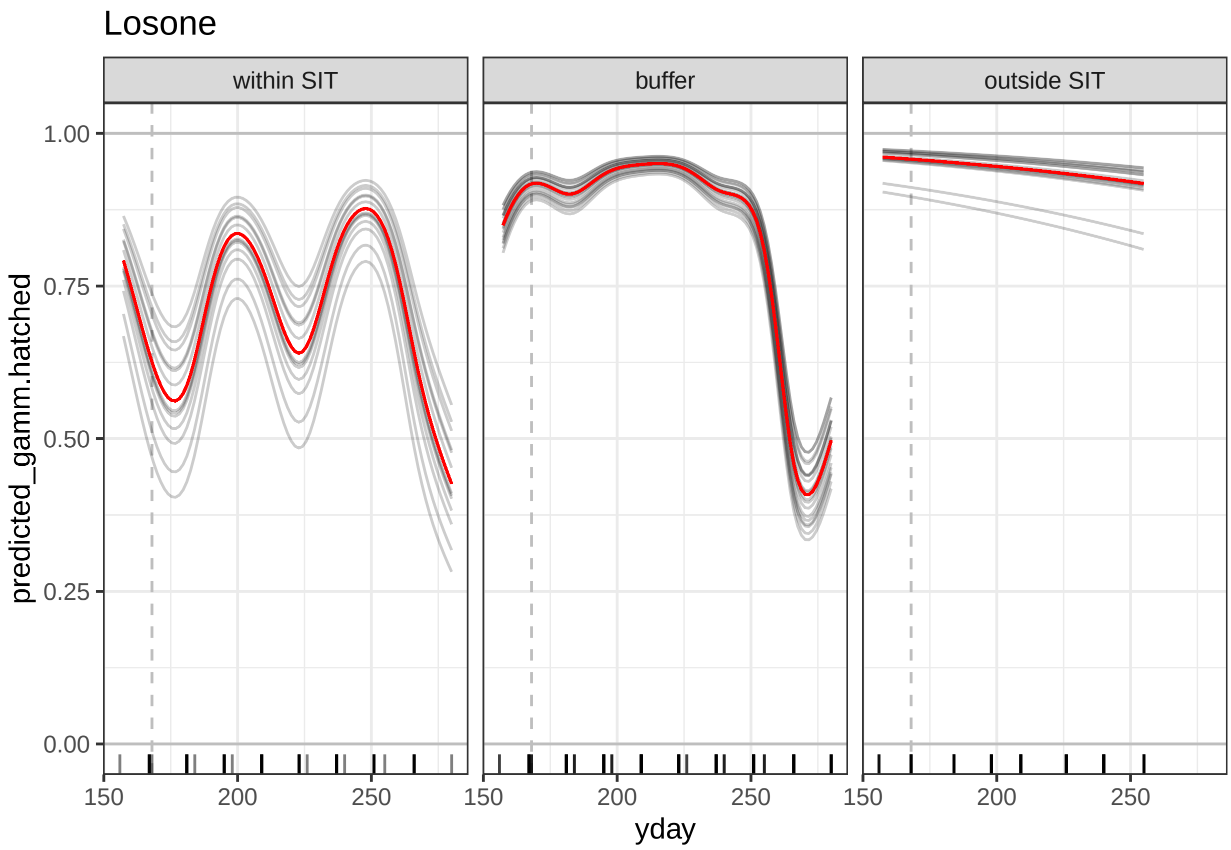


```
gg.prediction.gamm.Ascona <- ggplot(data = d.pred.gamm_aug.Ascona,
                                    mapping = aes(y = predicted_gamm.hatched,
                                                  x = yday,
                                                  group = unique.ID)) +
  geom_vline(xintercept = yday("2025-06-17"), linetype = "dashed", colour = "grey") +
  geom_hline(yintercept = c(0, 1), colour = "grey") +
  geom_line(alpha = 0.2) +
  geom_line(mapping = aes(y = predicted_gamm.hatched.pop),
            colour = "red") +
  facet_wrap(~ zona.SIT.discrete.fac) +
  geom_rug(data = d.hatching.Ascona, mapping = aes(x = yday),
           sides = "b",
           inherit.aes = FALSE,
           alpha = 0.5)
gg.prediction.gamm.Ascona +
  labs(title = "Ascona")
```

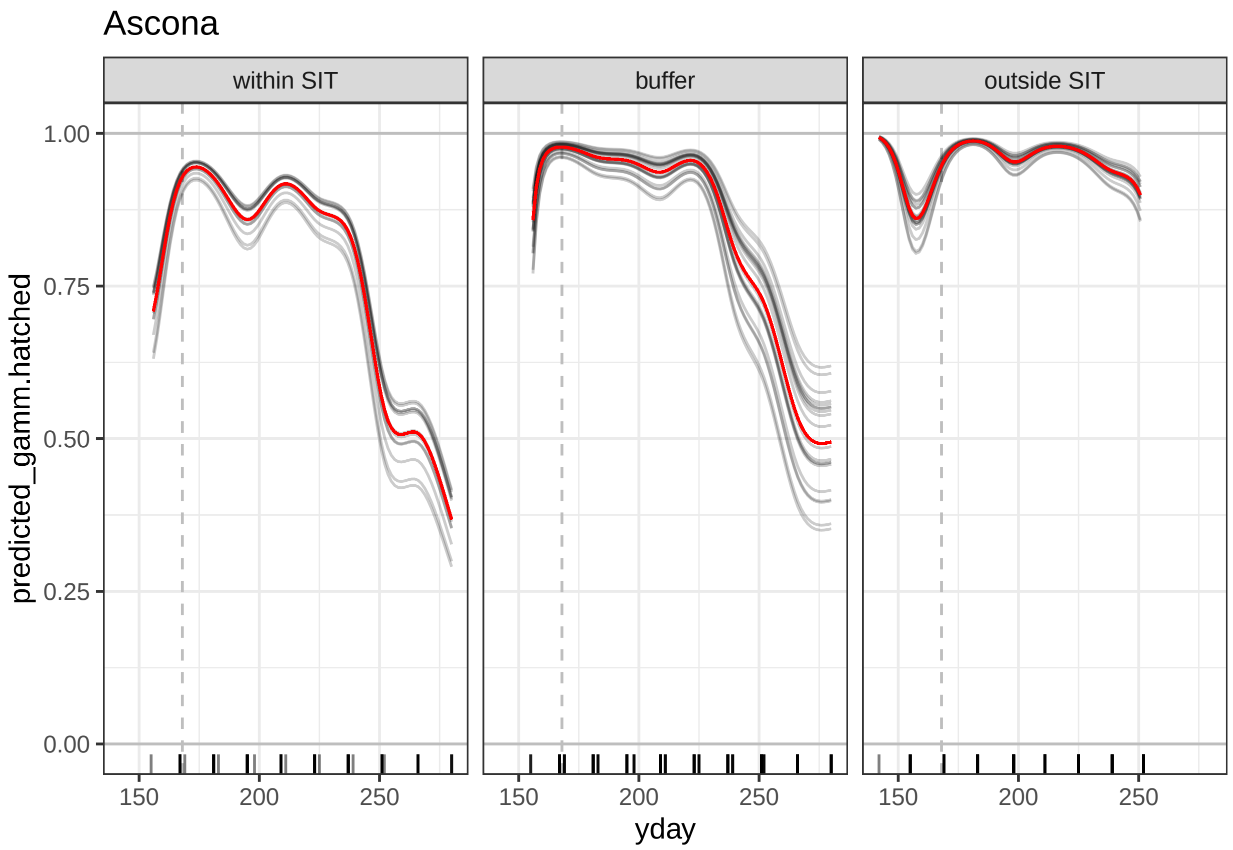

Each black line corresponds to the prediction for a given ovitrap, whereas the red line corresponds to the prediction at population level.

We plot the predictions at population level on the same plot.

```
gg.prediction.gamm.together <- ggplot(data = d.pred.gamm_aug,
                                      mapping = aes(y = predicted_gamm.hatched.pop,
                                                    x = yday,
                                                    colour = zona.SIT.discrete.fac)) +
  geom_vline(xintercept = yday(as.Date("2025-06-17")),
             linetype = "dashed", colour = "grey") +
  geom_line() +
  geom_hline(yintercept = c(0, 1), colour = "grey") +
  facet_wrap(~ municipality.fac) +
  labs(colour = "Zone") +
  theme(legend.position = "bottom")
gg.prediction.gamm.together
```

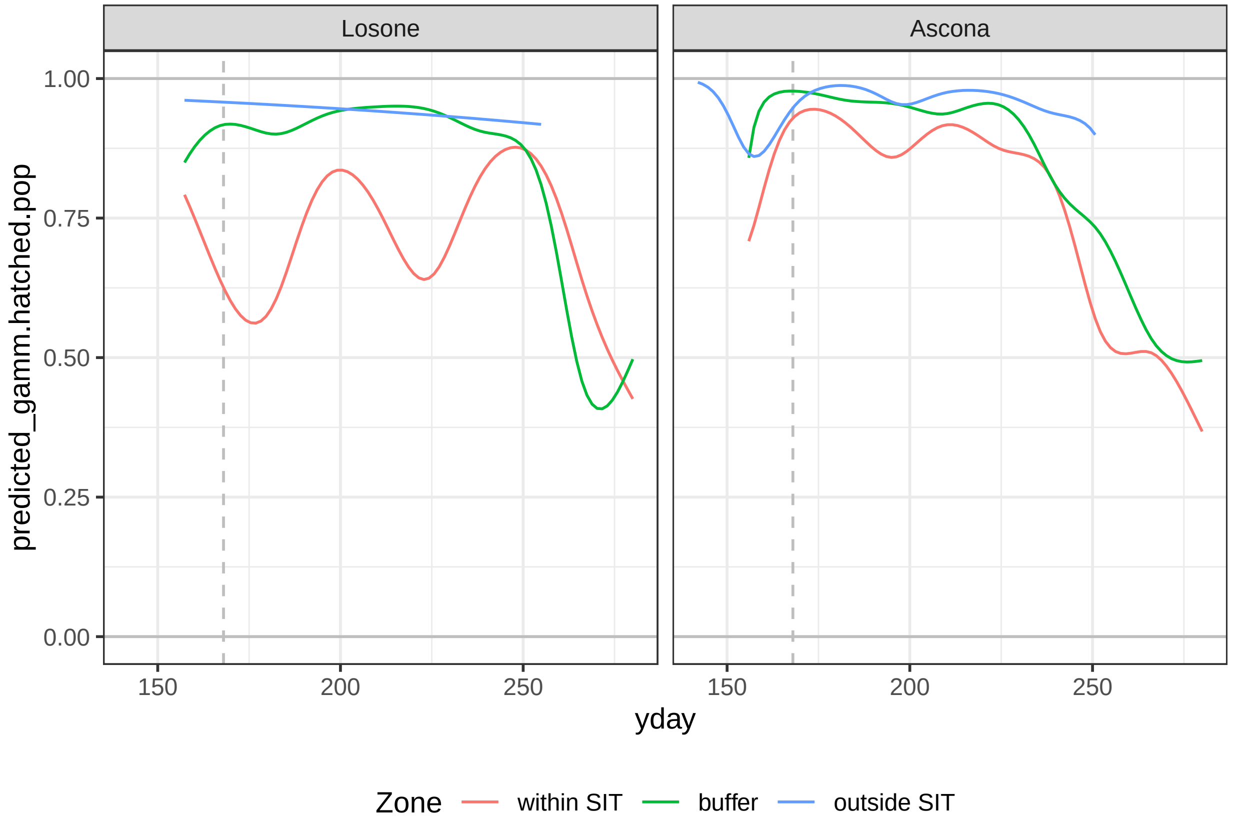

We observe that, in both municipalities, the predicted hatching rates in the untreated zones remain generally high throughout the season.

In both municipalities, the treated zones show lower predicted hatching rates than the others, with a stronger decrease (and also greater variability) in Losone compared to Ascona. Specifically, the smoother for the treated zone in Losone is quite wiggly, driven by a few particular dates.

The buffer zones display intermediate predicted values, lying between those of the treated and untreated areas.

Note that we did not plot the confidence bands here because we already have three curves overlaid. Adding the confidence bands would make the plot too cluttered and difficult to read. Instead, we will focus on plotting the differences between zones in the next section, along with the confidence bands, which will provide a clearer picture of the treatment effects.

5.6.1 Plotting the differences

We now plot the predicted differences between hatching rates in the treatment zones throughout the season, separately for each municipality.

We are no longer interested in the prediction at ovitrap level, but only at population level. Thus, we create a new data set containing only the necessary information for this purpose.

```
## We want to predict at population level, thus we don't care about the random
## effects. This means that we just need one observation per group.
d.pred.gamm_aug_pop <- d.pred.gamm_aug %>%
  group_by(yday, municipality.fac, zona.SIT.discrete.fac) %>%
  slice(1) %>%
  ungroup()
```

```r
##
## We need to start the comparison where all zones have available data.
d.pred.gamm_aug_pop <- d.pred.gamm_aug_pop %>%
  filter(yday >= max(yday.min) & yday <= min(yday.max)) %>%
  select(-yday.min, -yday.max)
```

5.6.1.1 Buffer versus within SIT zone – Losone

We start by looking at the difference between the buffer and the treated zone in Losone.

We create the data set on which we are going to calculate the contrasts. We make sure that we first have all the observations belonging to the "within SIT" level, followed by those of the "buffer" level.

```r
d.pred.gamm_aug.Losone.25.SIT.buffer <- d.pred.gamm_aug_pop %>%
  filter(municipality.fac == "Losone") %>%
  filter(zona.SIT.discrete.fac %in% c("within SIT", "buffer")) %>%
  droplevels() %>%
  arrange(zona.SIT.discrete.fac, yday)
##
## check
d.pred.gamm_aug.Losone.25.SIT.buffer %>%
  pull(zona.SIT.discrete.fac)
```

```
  [1] within SIT within SIT within SIT within SIT within SIT within SIT
  [7] within SIT within SIT within SIT within SIT within SIT within SIT
 [13] within SIT within SIT within SIT within SIT within SIT within SIT
 [19] within SIT within SIT within SIT within SIT within SIT within SIT
 [25] within SIT within SIT within SIT within SIT within SIT within SIT
 [31] within SIT within SIT within SIT within SIT within SIT within SIT
 [37] within SIT within SIT within SIT within SIT within SIT within SIT
 [43] within SIT within SIT within SIT within SIT within SIT within SIT
 [49] within SIT within SIT within SIT within SIT within SIT within SIT
 [55] within SIT within SIT within SIT within SIT within SIT within SIT
 [61] within SIT within SIT within SIT within SIT within SIT within SIT
 [67] within SIT within SIT buffer     buffer     buffer     buffer
 [73] buffer     buffer     buffer     buffer     buffer     buffer
 [79] buffer     buffer     buffer     buffer     buffer     buffer
 [85] buffer     buffer     buffer     buffer     buffer     buffer
 [91] buffer     buffer     buffer     buffer     buffer     buffer
 [97] buffer     buffer     buffer     buffer     buffer     buffer
[103] buffer     buffer     buffer     buffer     buffer     buffer
[109] buffer     buffer     buffer     buffer     buffer     buffer
[115] buffer     buffer     buffer     buffer     buffer     buffer
[121] buffer     buffer     buffer     buffer     buffer     buffer
[127] buffer     buffer     buffer     buffer     buffer     buffer
[133] buffer     buffer     buffer     buffer
Levels: within SIT buffer
```

```r
## makes sense
```

Next, we create the model matrix for calculating the contrasts.

```r
## Model matrix
W.predictions.buffer.sit.Losone <- predict(gamm.hatched.Losone,
                                           newdata = d.pred.gamm_aug.Losone.25.SIT.buffer,
```

```
                                        type = "lpmatrix",

                                        ## Prediction at population level
                                        exclude = 's(unique.ID)')
```

We also extract the coefficients of the model and the corresponding variance-covariance matrix.

```
c.coeff.gamm.hatched.Losone <- coef(gamm.hatched.Losone)
vcov.c.coeff.gamm.hatched.Losone <- vcov(gamm.hatched.Losone)
```

We can now compute the contrasts.

```
## 1) We count the number of rows of the matrix representing the "within SIT" level
n <- d.pred.gamm_aug.Losone.25.SIT.buffer %>%
  filter(zona.SIT.discrete.fac == "within SIT") %>%
  nrow() %>%
  print()
```

```
[1] 68
```

```
##
## check that "buffer" matrix has the same number of rows
d.pred.gamm_aug.Losone.25.SIT.buffer %>%
  filter(zona.SIT.discrete.fac == "buffer") %>%
  nrow()
```

```
[1] 68
```

```
## makes sense

## 2) We calculate the contrasts, exploiting the model matrix.

##  i) We calculate the contrast matrix L as (X_buffer - X_withinSIT)

## We made sure that we first have all the "within SIT" observations,
## and then all the "buffer" ones, thus we can do as follows:
L.buffer.Losone  <- W.predictions.buffer.sit.Losone[(n + 1):(2*n), , drop = FALSE] -
  W.predictions.buffer.sit.Losone[1:n, , drop = FALSE]

##  ii) multiply L by beta to calculate the difference between the predicted values
## => eta = L * beta
est.buffer.Losone <- (L.buffer.Losone %*% c.coeff.gamm.hatched.Losone) %>%
  as.vector()
```

Now that we have computed the contrasts, we can calculate the corresponding standard error.

```
## 3) We calculate the corresponding standard error, which is given by
## the variance of L*beta
## Var(L * beta) = diag( L %*% Var(beta) %*% t(L) )
## and then we take the sqrt for the standard error
## the following code computes it
se.buffer.Losone  <-
  ( (L.buffer.Losone %*% vcov.c.coeff.gamm.hatched.Losone) * L.buffer.Losone ) %>%
  rowSums() %>%
  sqrt()
## equivalent to
# se.buffer.Losone.2 <-
```

```r
# ( (L.buffer.Losone %*% vcov.c.coeff.gamm.hatched.Losone) %*% t(L.buffer.Losone) ) %>%
#   diag() %>%
#   sqrt()
## but requires less computing power
```

We store everything in a data frame and plot the results.

```r
d.diff.buffer.Losone <- data.frame(
  yday = d.pred.gamm_aug.Losone$yday[1:n],
  diff = est.buffer.Losone,
  lo = est.buffer.Losone - qnorm(0.975) * se.buffer.Losone,
  hi = est.buffer.Losone + qnorm(0.975) * se.buffer.Losone) %>%
  ##
  ## back transform the estimated values
  mutate(exp.diff = exp(diff),
         exp.lo = exp(lo),
         exp.hi = exp(hi))
```

We plot the result.

```r
ggplot(d.diff.buffer.Losone, mapping = aes(x = yday, y = diff)) +
  geom_ribbon(aes(ymin = lo, ymax = hi), fill = "lightgray", alpha = 0.5) +
  geom_line(linewidth = 1) +
  geom_hline(yintercept = 0, linetype = "dashed") +
  geom_vline(xintercept = yday("2025-06-17"), linetype = "dashed", colour = "grey") +
  labs(title = "Losone",
       y = "buffer minus treated zone") +
  coord_cartesian(ylim = c(-3, 3))
```

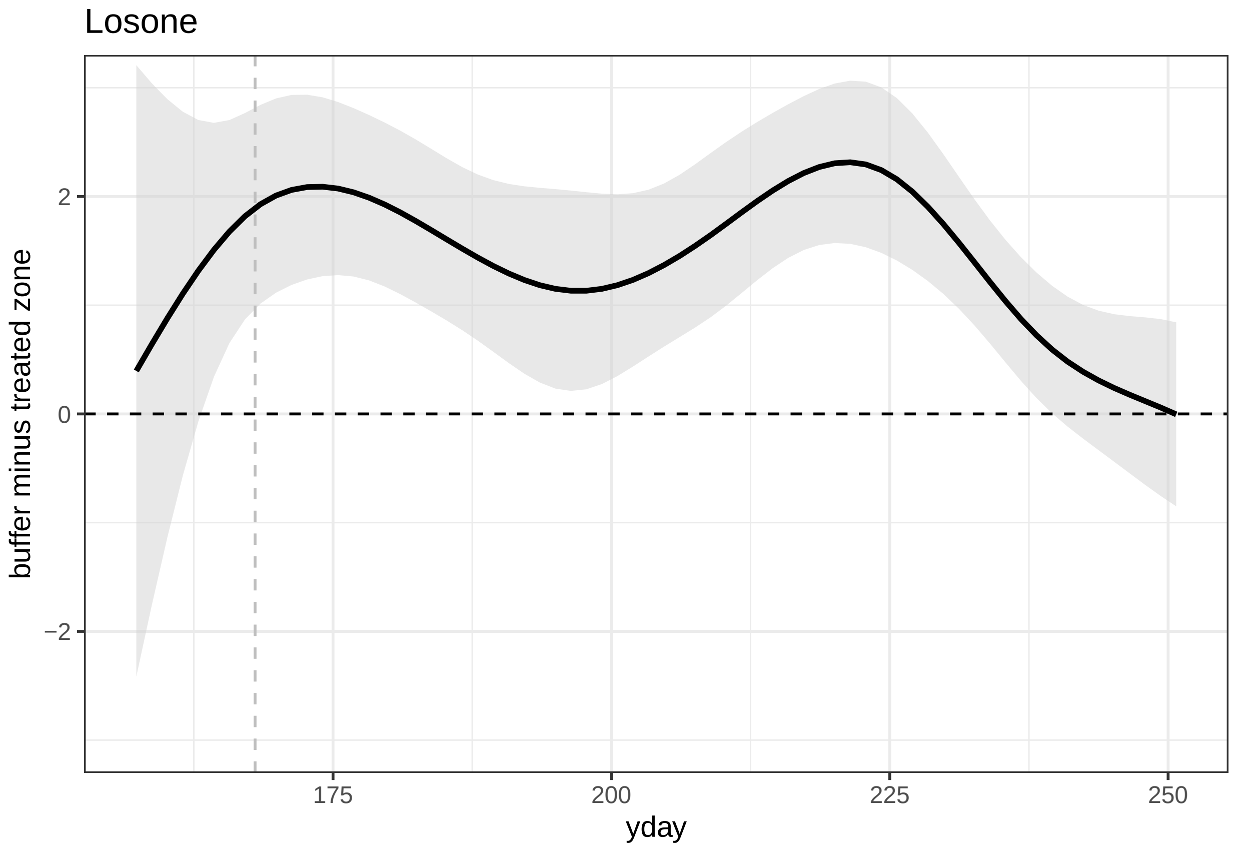

The plot shows the difference between the predicted values on the linear predictor scale for the buffer and treated zones, along with a 95% confidence interval. A horizontal dashed line at 0 indicates no difference between the two zones, while a vertical dashed line marks the start of the treatment on June 17, 2025.

We back-transform the difference by applying the exponential function, which returns the values to the odds scale. After this transformation, the values can be interpreted as ratios of predicted odds of hatching: a value of 1 indicates no difference between zones, values greater than 1 indicate higher predicted odds of hatching in the buffer zone relative to the treated zone, and values below 1 indicate the opposite.

```r
## In the back transformed scale
gg.buffer.losone.exp <- ggplot(d.diff.buffer.Losone,
                              mapping = aes(x = yday, y = exp.diff)) +
  geom_ribbon(aes(ymin = exp.lo, ymax = exp.hi), fill = "lightgray", alpha = 0.5) +
  geom_line(linewidth = 1) +
  geom_hline(yintercept = 1, linetype = "dashed") +
  geom_vline(xintercept = yday("2025-06-17"), linetype = "dashed", colour = "grey") +
  labs(title = "Losone",
       y = "buffer minus treated zone \nin the back transformed scale")
gg.buffer.losone.exp
```

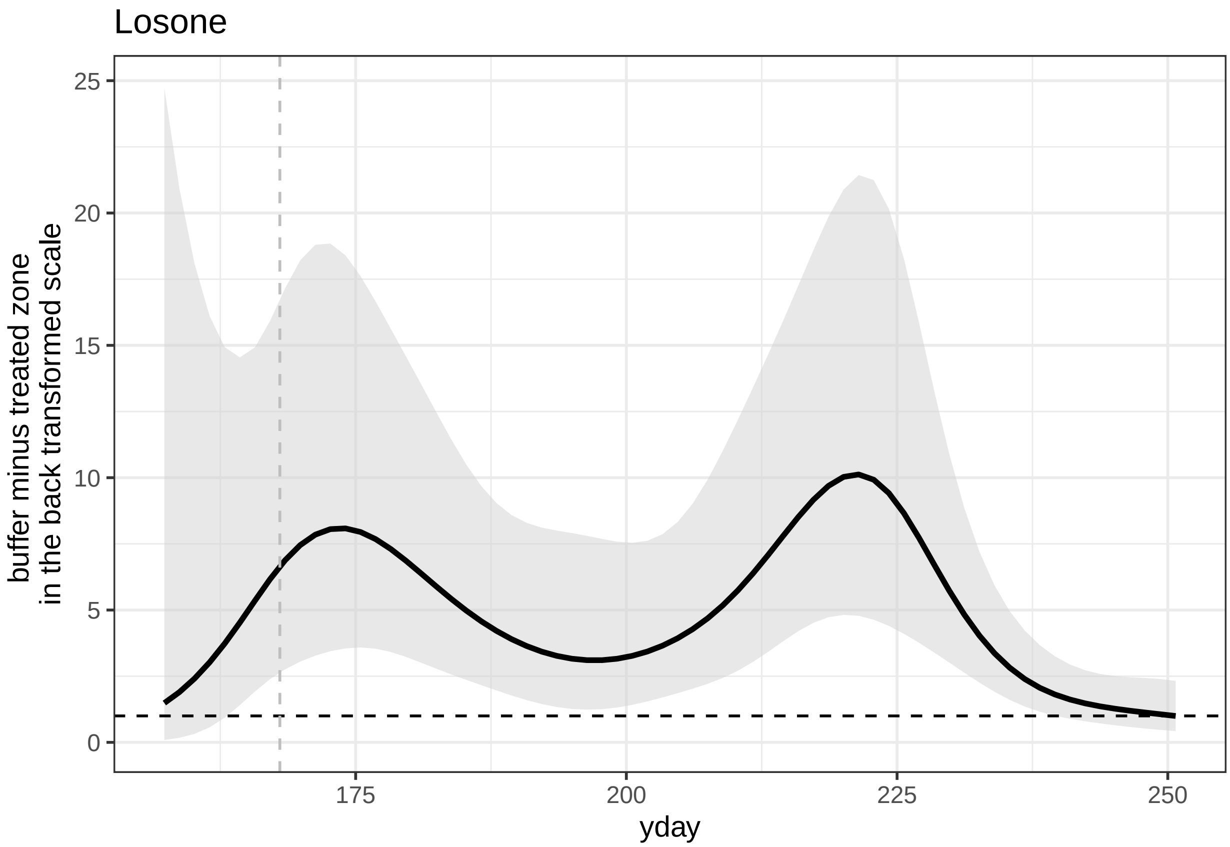

We zoom in to focus on the most relevant part of the graph.

```r
## we zoom in
gg.buffer.losone.exp +
  coord_cartesian(ylim = c(0, 20))
```

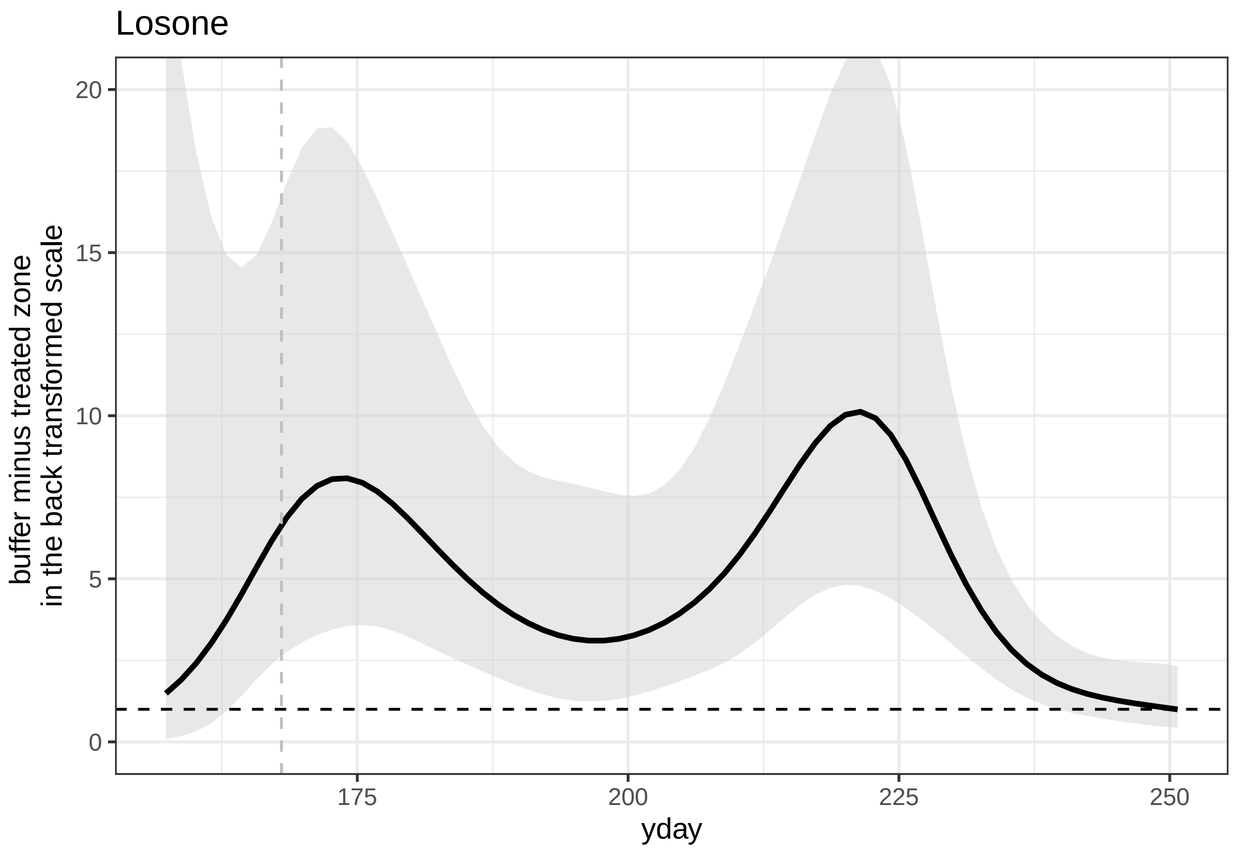

The predicted odds of an egg hatching are initially quite similar in the two zones (note that the confidence band is very large, showing high uncertainty). Once the treatment begins, the odds in the buffer zone become higher than those in the treated zone, with some variability over time. On day `221`, this difference reaches its maximum: the predicted odds of hatching in the buffer zone are `10.1` times higher than in the treated zone.

Note that the difference between the two zones is statistically significant when the grey zone doesn't intersect with the horizontal dashed line at 1.

We can better understand this with an example.

Let's calculate the predicted hatching rate in the treated zone for day `221`.

```
d.max.diff <- data.frame(yday = yday.max.diff,
                         zona.SIT.discrete.fac = "within SIT",
                         unique.ID = "Losone.16a")
predicted.hatching.treated <- predict(gamm.hatched.Losone, newdata = d.max.diff,
                                      type = "response",
                                      exclude = 's(unique.ID)') %>%
  print()
```

```
                1
0.642872433388271
```

The predicted hatching rate in the treated zone is `64.3` %. This means that, out of 100 eggs, about `64` are expected to hatch and `36` are not. The corresponding odds of hatching are therefore: probability hatching / probability not hatching = `1.8`. In other words, an egg is almost twice as likely to hatch than to not hatch.

Now, if the model predicts that the odds of hatching in the buffer zone are 10.1 times higher than in the treated zone, we can multiply 1.8 by this value. In other words, the odds in the buffer zone are:

```
odds.untreated <- ( predicted.hatching.treated / (1 - predicted.hatching.treated) *
                      max(d.diff.buffer.Losone$exp.diff) ) %>%
  round(digits = 2) %>%
  print()
```

```
    1
18.22
```

Converting these odds back into a probability, we get:

```
prop.untreated <- round(odds.untreated / (1 + odds.untreated) * 100, digits = 1) %>%
  print()
```

```
   1
94.8
```

This means that the predicted hatching rate in the buffer zone is about 94.8 %, compared to 64.3 % in the treated zone.

5.6.1.2 Buffer versus within SIT zone – Ascona

We redo the same exact process, but for Ascona. For the full explanation, look at the section above.

```
d.pred.gamm_aug.Ascona.25.SIT.buffer <- d.pred.gamm_aug_pop %>%
  filter(municipality.fac == "Ascona") %>%
  filter(zona.SIT.discrete.fac %in% c("within SIT", "buffer")) %>%
  droplevels() %>%
  arrange(zona.SIT.discrete.fac, yday)
##
## check
d.pred.gamm_aug.Ascona.25.SIT.buffer %>%
  pull(zona.SIT.discrete.fac)
```

```
  [1] within SIT within SIT within SIT within SIT within SIT within SIT
  [7] within SIT within SIT within SIT within SIT within SIT within SIT
 [13] within SIT within SIT within SIT within SIT within SIT within SIT
 [19] within SIT within SIT within SIT within SIT within SIT within SIT
 [25] within SIT within SIT within SIT within SIT within SIT within SIT
 [31] within SIT within SIT within SIT within SIT within SIT within SIT
 [37] within SIT within SIT within SIT within SIT within SIT within SIT
 [43] within SIT within SIT within SIT within SIT within SIT within SIT
 [49] within SIT within SIT within SIT within SIT within SIT within SIT
 [55] within SIT within SIT within SIT within SIT within SIT within SIT
 [61] within SIT within SIT within SIT within SIT within SIT within SIT
 [67] within SIT within SIT buffer     buffer     buffer     buffer
 [73] buffer     buffer     buffer     buffer     buffer     buffer
 [79] buffer     buffer     buffer     buffer     buffer     buffer
 [85] buffer     buffer     buffer     buffer     buffer     buffer
 [91] buffer     buffer     buffer     buffer     buffer     buffer
 [97] buffer     buffer     buffer     buffer     buffer     buffer
[103] buffer     buffer     buffer     buffer     buffer     buffer
[109] buffer     buffer     buffer     buffer     buffer     buffer
[115] buffer     buffer     buffer     buffer     buffer     buffer
[121] buffer     buffer     buffer     buffer     buffer     buffer
```

```
[127] buffer     buffer     buffer     buffer     buffer     buffer
[133] buffer     buffer     buffer     buffer
Levels: within SIT buffer
```

```r
## makes sense
```

```r
## Model matrix
W.predictions.buffer.sit.Ascona <- predict(gamm.hatched.Ascona,
                                           newdata = d.pred.gamm_aug.Ascona.25.SIT.buffer,
                                           type = "lpmatrix",

                                           ## Prediction at population level
                                           exclude = 's(unique.ID)')
##
c.coeff.gamm.hatched.Ascona <- coef(gamm.hatched.Ascona)
vcov.c.coeff.gamm.hatched.Ascona <- vcov(gamm.hatched.Ascona)


## Calculate the contrasts

## First, count the rows of the matrix containing only "within SIT"
n2 <- d.pred.gamm_aug.Ascona.25.SIT.buffer %>%
  filter(zona.SIT.discrete.fac == "within SIT") %>%
  nrow() %>%
  print()
```

```
[1] 68
```

```r
##
## check that "buffer" has the same dimension
d.pred.gamm_aug.Ascona.25.SIT.buffer %>%
  filter(zona.SIT.discrete.fac == "buffer") %>%
  nrow()
```

```
[1] 68
```

```r
## makes sense

## Calculate the contrasts
L.buffer.Ascona  <- W.predictions.buffer.sit.Ascona[(n2 + 1):(2*n2), , drop = FALSE] -
  W.predictions.buffer.sit.Ascona[1:n2, , drop = FALSE]
est.buffer.Ascona <- as.vector(L.buffer.Ascona %*% c.coeff.gamm.hatched.Ascona)

## Now, we calculate the corresponding standard error
se.buffer.Ascona  <-
  ( (L.buffer.Ascona %*% vcov.c.coeff.gamm.hatched.Ascona) * L.buffer.Ascona) %>%
  rowSums() %>%
  sqrt()

d.diff.buffer.Ascona <- data.frame(
  yday = d.pred.gamm_aug.Ascona$yday[1:n2],
  diff = est.buffer.Ascona,
  lo = est.buffer.Ascona - qnorm(0.975) * se.buffer.Ascona,
  hi = est.buffer.Ascona + qnorm(0.975) * se.buffer.Ascona
) %>%
  mutate(exp.diff = exp(diff),
         exp.lo = exp(lo),
```

```
        exp.hi = exp(hi))
```

We plot the result.

```
ggplot(d.diff.buffer.Ascona, mapping = aes(x = yday, y = diff)) +
  geom_ribbon(aes(ymin = lo, ymax = hi), fill = "lightgray", alpha = 0.5) +
  geom_line(linewidth = 1) +
  geom_hline(yintercept = 0, linetype = "dashed") +
  geom_vline(xintercept = yday("2025-06-17"), linetype = "dashed", colour = "grey") +
  labs(title = "Ascona",
       y = "buffer minus treated zone")
```

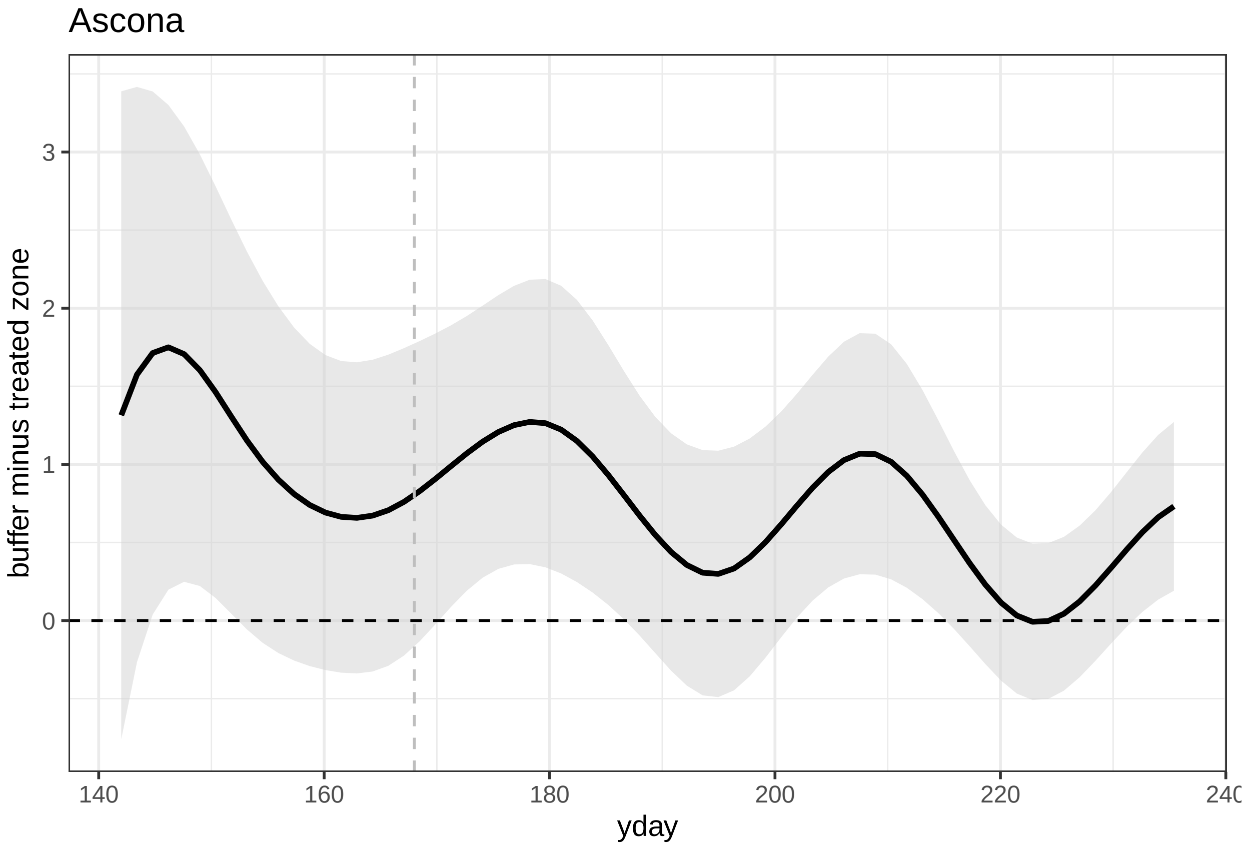

The plot shows the difference between the predicted values on the linear predictor scale for the buffer and treated zones, along with a 95% confidence interval. A horizontal dashed line at 0 indicates no difference between the two zones, while a vertical dashed line marks the start of the treatment on June 17, 2025.

We back-transform the difference by applying the exponential function, which returns the values to the odds scale. After this transformation, the values can be interpreted as ratios of predicted odds of hatching: a value of 1 indicates no difference between zones, values greater than 1 indicate higher predicted odds of hatching in the buffer zone relative to the treated zone, and values below 1 indicate the opposite.

```
## in the back transformed scale
gg.buffer.Ascona <- ggplot(d.diff.buffer.Ascona, mapping = aes(x = yday, y = exp.diff)) +
  geom_ribbon(aes(ymin = exp.lo, ymax = exp.hi), fill = "lightgray", alpha = 0.5) +
  geom_line(linewidth = 1) +
  geom_hline(yintercept = 1, linetype = "dashed") +
  geom_vline(xintercept = yday("2025-06-17"), linetype = "dashed", colour = "grey") +
  labs(title = "Ascona",
```

```
      y = "buffer minus treated zone \nin the back transformed scale")
gg.buffer.Ascona
```

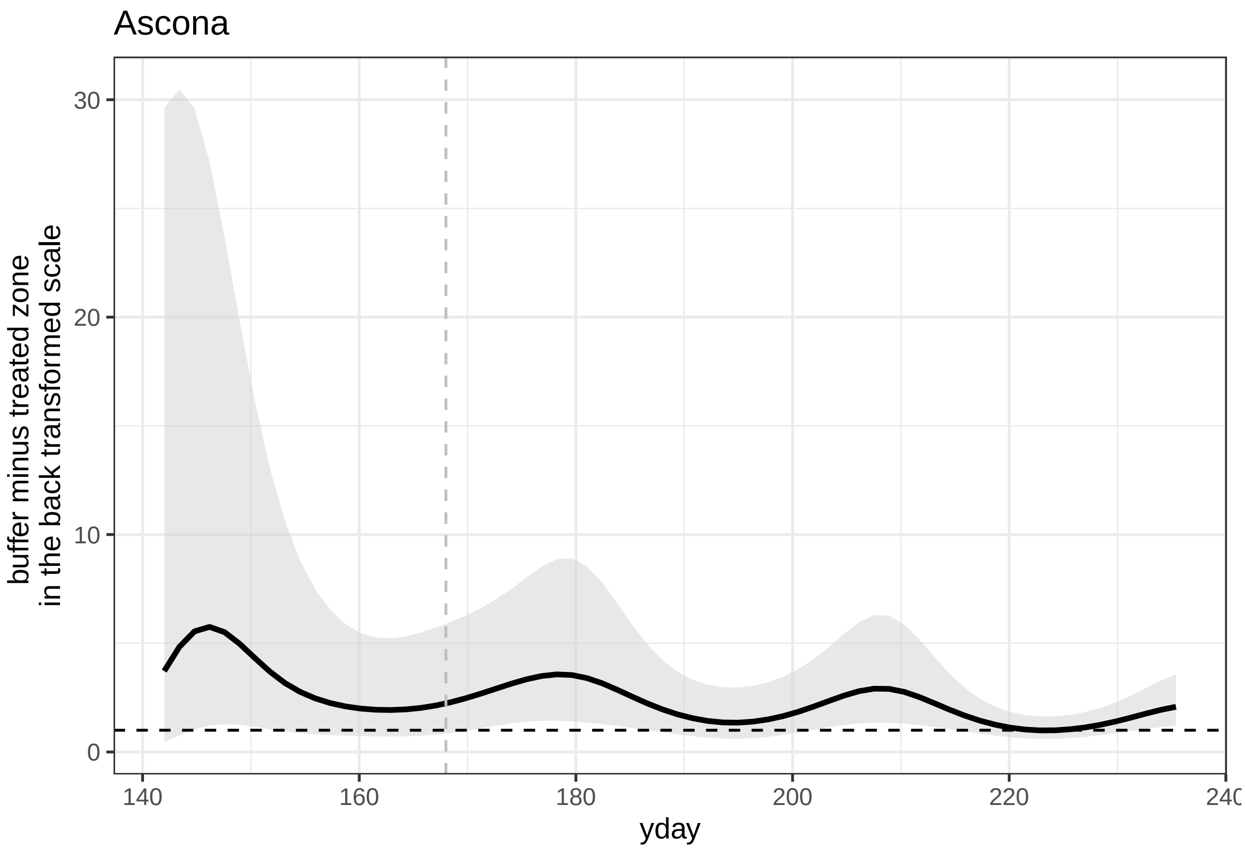

We zoom in to focus on the most relevant part of the graph. We use the same zoom as for Losone to facilitate comparison.

```
## we zoom in, using the same zoom as for Losone
gg.buffer.Ascona +
  coord_cartesian(ylim = c(0, 20))
```

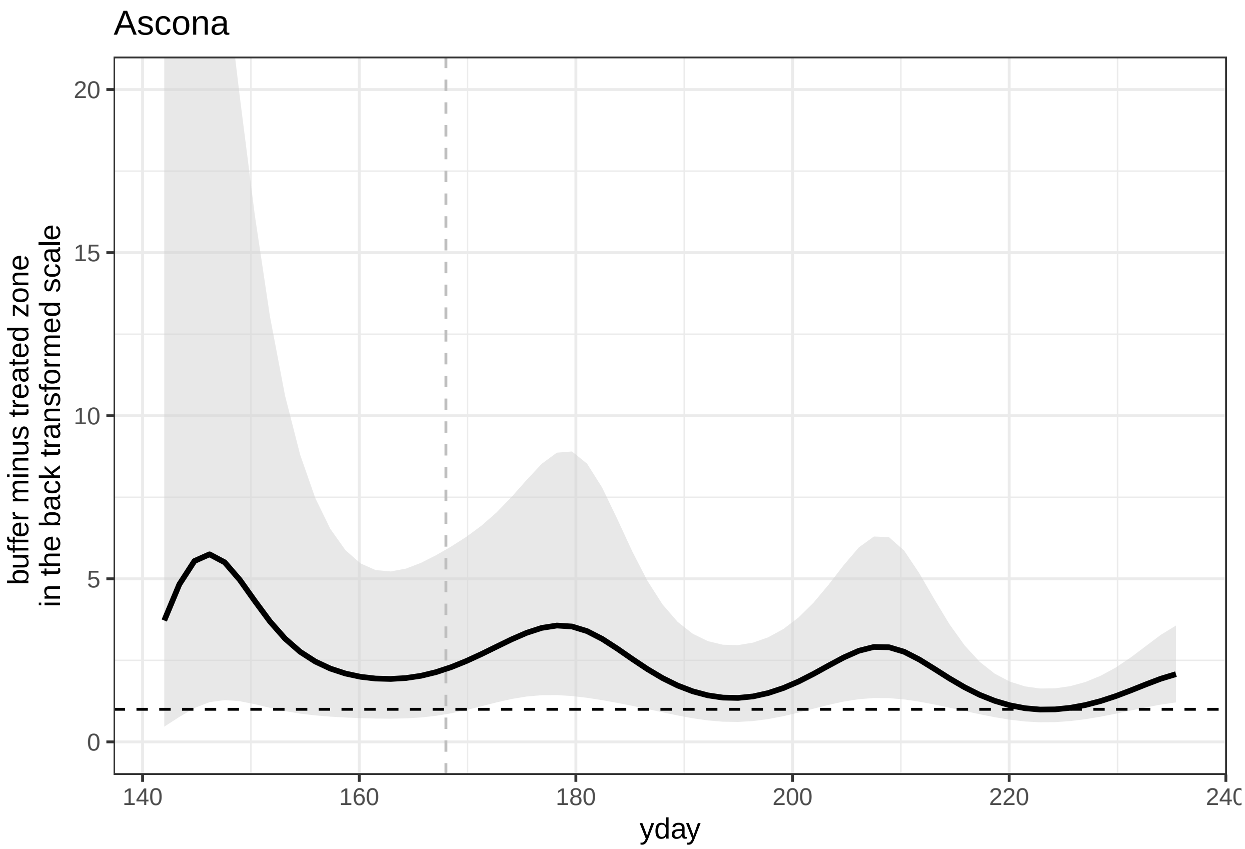

The buffer zone starts by having higher hatching odds compared to the untreated area (the confidence band is very large, showing high uncertainty). This difference remains quite constant over time, i.e., the buffer zone has higher predicted hatching rates compared to the treated zone.

Note that the difference between the two zones is statistically significant when the grey zone doesn't intersect with the horizontal dashed line at 1.

5.6.1.3 outside SIT versus within SIT zone – Losone We now perform the same steps as in the previous two sections, but replacing the buffer area with the untreated zone. We start with Losone.

```
d.pred.gamm_aug.Losone.25.SIT.outside <- d.pred.gamm_aug_pop %>%
  filter(municipality.fac == "Losone") %>%
  filter(zona.SIT.discrete.fac %in% c("within SIT", "outside SIT")) %>%
  droplevels() %>%
  arrange(zona.SIT.discrete.fac, yday)
##
## check
d.pred.gamm_aug.Losone.25.SIT.outside %>%
  pull(zona.SIT.discrete.fac)
```

```
  [1] within SIT  within SIT  within SIT  within SIT  within SIT  within SIT
  [7] within SIT  within SIT  within SIT  within SIT  within SIT  within SIT
 [13] within SIT  within SIT  within SIT  within SIT  within SIT  within SIT
 [19] within SIT  within SIT  within SIT  within SIT  within SIT  within SIT
 [25] within SIT  within SIT  within SIT  within SIT  within SIT  within SIT
 [31] within SIT  within SIT  within SIT  within SIT  within SIT  within SIT
 [37] within SIT  within SIT  within SIT  within SIT  within SIT  within SIT
```

```
 [43] within SIT  within SIT  within SIT  within SIT  within SIT  within SIT
 [49] within SIT  within SIT  within SIT  within SIT  within SIT  within SIT
 [55] within SIT  within SIT  within SIT  within SIT  within SIT  within SIT
 [61] within SIT  within SIT  within SIT  within SIT  within SIT  within SIT
 [67] within SIT  within SIT  outside SIT outside SIT outside SIT outside SIT
 [73] outside SIT outside SIT outside SIT outside SIT outside SIT outside SIT
 [79] outside SIT outside SIT outside SIT outside SIT outside SIT outside SIT
 [85] outside SIT outside SIT outside SIT outside SIT outside SIT outside SIT
 [91] outside SIT outside SIT outside SIT outside SIT outside SIT outside SIT
 [97] outside SIT outside SIT outside SIT outside SIT outside SIT outside SIT
[103] outside SIT outside SIT outside SIT outside SIT outside SIT outside SIT
[109] outside SIT outside SIT outside SIT outside SIT outside SIT outside SIT
[115] outside SIT outside SIT outside SIT outside SIT outside SIT outside SIT
[121] outside SIT outside SIT outside SIT outside SIT outside SIT outside SIT
[127] outside SIT outside SIT outside SIT outside SIT outside SIT outside SIT
[133] outside SIT outside SIT outside SIT outside SIT
Levels: within SIT outside SIT
```

```r
## makes sense
```

```r
## Model matrix
W.predictions.outside.sit <- predict(gamm.hatched.Losone,
                                     newdata = d.pred.gamm_aug.Losone.25.SIT.outside,
                                     type = "lpmatrix",

                                     ## Prediction at population level
                                     exclude = 's(unique.ID)')

## Calculate the contrasts

## First, count the rows of the matrix containing only "within SIT"
n3 <- d.pred.gamm_aug.Losone.25.SIT.outside %>%
  filter(zona.SIT.discrete.fac == "within SIT") %>%
  nrow() %>%
  print()
```

```
[1] 68
```

```r
##
## check that "outside SIT" has the same dimension
d.pred.gamm_aug.Losone.25.SIT.outside %>%
  filter(zona.SIT.discrete.fac == "outside SIT") %>%
  nrow()
```

```
[1] 68
```

```r
## makes sense

## Calculate the estimates of the contrasts, starting from the model matrix.
## Proceed as follows: first calculate L, then multiply it by beta.
## L.outside.Losone * beta = (X_outside SIT - X_withinSIT) * beta
L.outside.Losone  <- W.predictions.outside.sit[(n3 + 1):(2*n3), , drop = FALSE] -
  W.predictions.outside.sit[1:n3, , drop = FALSE]
est.outside.Losone <- as.vector(L.outside.Losone %*% c.coeff.gamm.hatched.Losone)

## Now, we calculate the corresponding standard error
se.outside.Losone  <-
```

```
  ( (L.outside.Losone %*% vcov.c.coeff.gamm.hatched.Losone) * L.outside.Losone ) %>%
  rowSums() %>%
  sqrt()

d.diff.outside.Losone <- data.frame(
  yday = d.pred.gamm_aug.Losone$yday[1:n3],
  diff = est.outside.Losone,
  lo = est.outside.Losone - qnorm(0.975) * se.outside.Losone,
  hi = est.outside.Losone + qnorm(0.975) * se.outside.Losone
) %>%
  mutate(exp.diff = exp(diff),
         exp.lo = exp(lo),
         exp.hi = exp(hi))
```

We plot the result.

```
ggplot(d.diff.outside.Losone, mapping = aes(x = yday, y = diff)) +
  geom_ribbon(aes(ymin = lo, ymax = hi), fill = "lightgray", alpha = 0.5) +
  geom_line(linewidth = 1) +
  geom_hline(yintercept = 0, linetype = "dashed") +
  geom_vline(xintercept = yday("2025-06-17"), linetype = "dashed", colour = "grey") +
  labs(title = "Losone",
       y = "outside SIT minus treated zone")
```

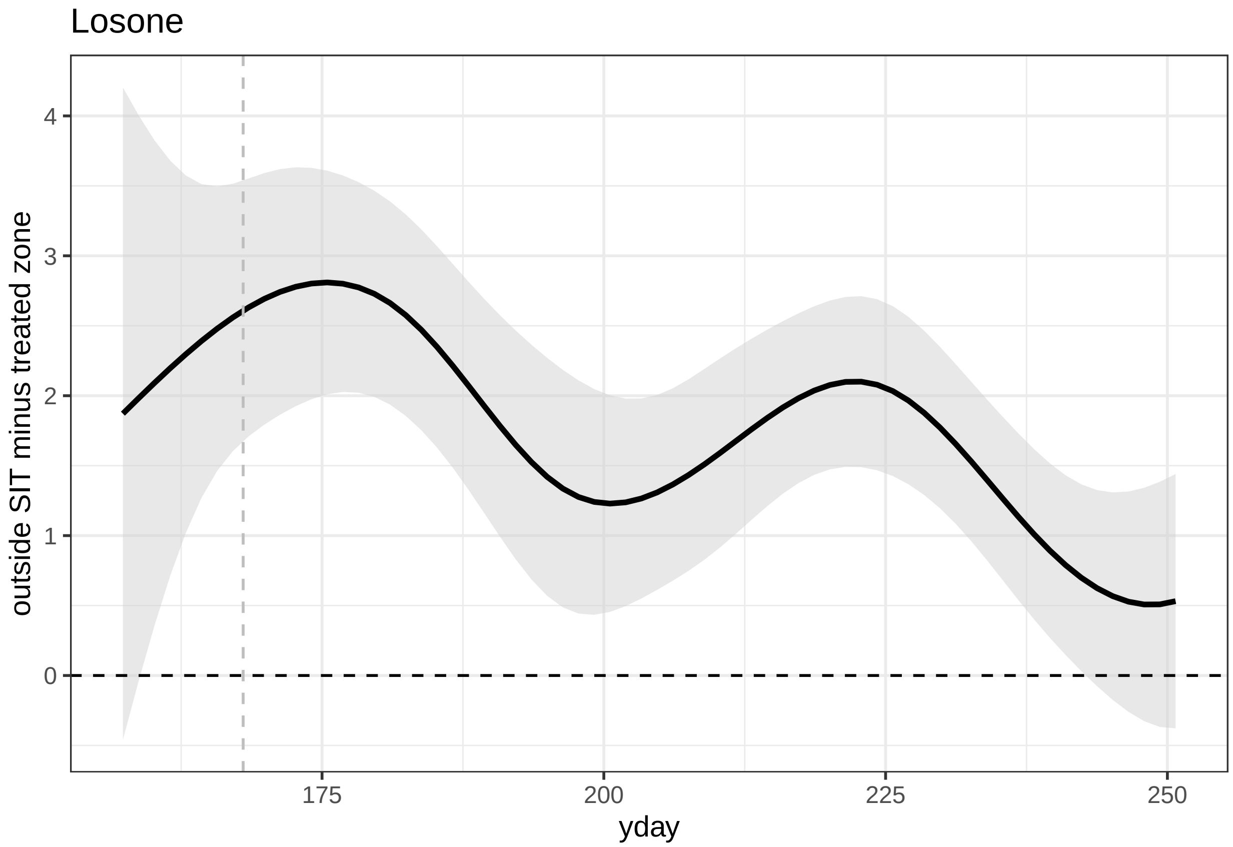

The plot shows the difference between the predicted values on the linear predictor scale for the untreated and treated zones, along with a 95% confidence interval. A horizontal dashed line at 0 indicates no difference between the two zones, while a vertical dashed line marks the start of the treatment on June 17, 2025.

We back-transform the difference by applying the exponential function, which returns the values to the odds scale. After this transformation, the values can be interpreted as ratios of predicted odds of hatching: a value of 1 indicates no difference between zones, values greater than 1 indicate higher predicted odds of hatching in the untreated zone relative to the treated zone, and values below 1 indicate the opposite.

```
gg.outside.Losone <- ggplot(d.diff.outside.Losone, mapping = aes(x = yday, y = exp.diff)) +
  geom_ribbon(aes(ymin = exp.lo, ymax = exp.hi), fill = "lightgray", alpha = 0.5) +
  geom_line(linewidth = 1) +
  geom_hline(yintercept = 1, linetype = "dashed") +
  geom_vline(xintercept = yday("2025-06-17"), linetype = "dashed", colour = "grey") +
  labs(title = "Losone",
       y = "outside SIT minus treated zone \nin the back transformed scale")
gg.outside.Losone
```

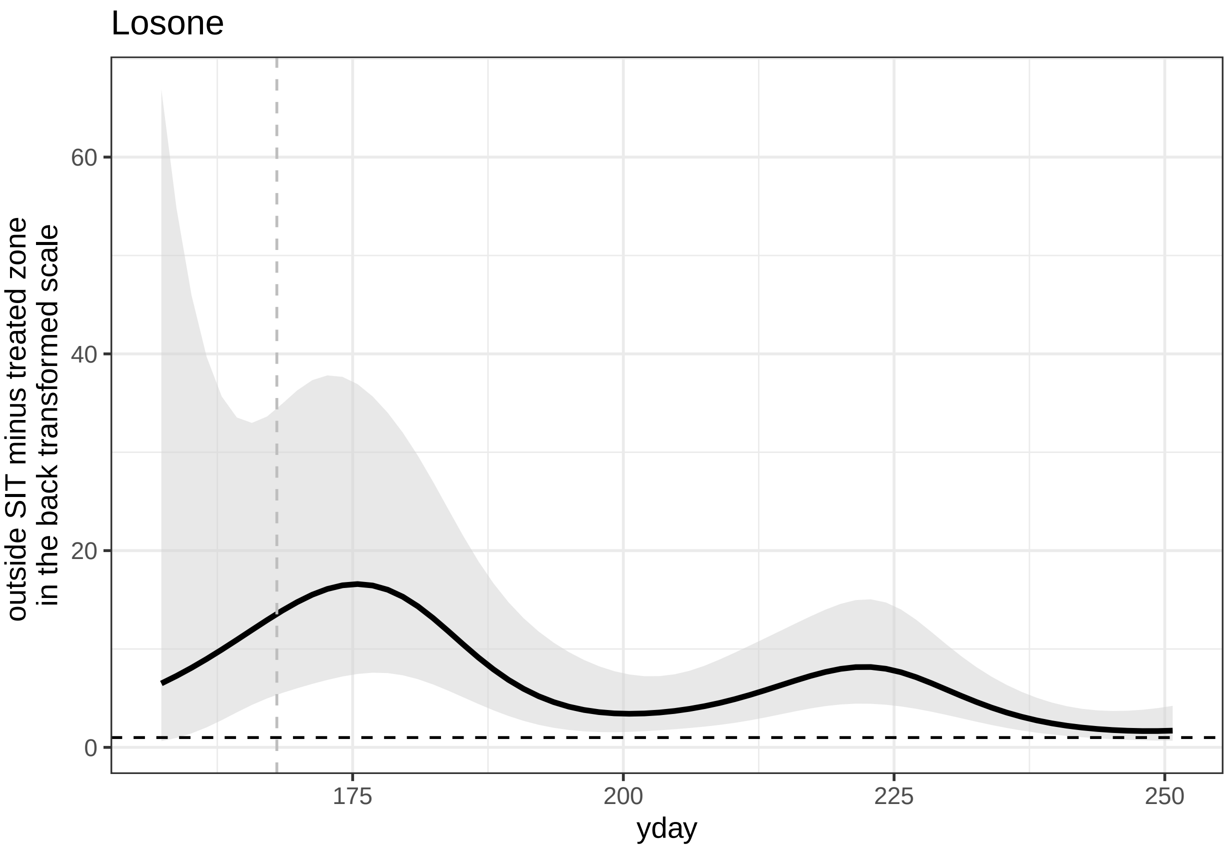

We zoom in to focus on the most relevant part of the graph. We use the same zoom as for the previous plots to facilitate comparison.

```
## we zoom in
gg.outside.Losone +
  coord_cartesian(ylim = c(0, 20))
```

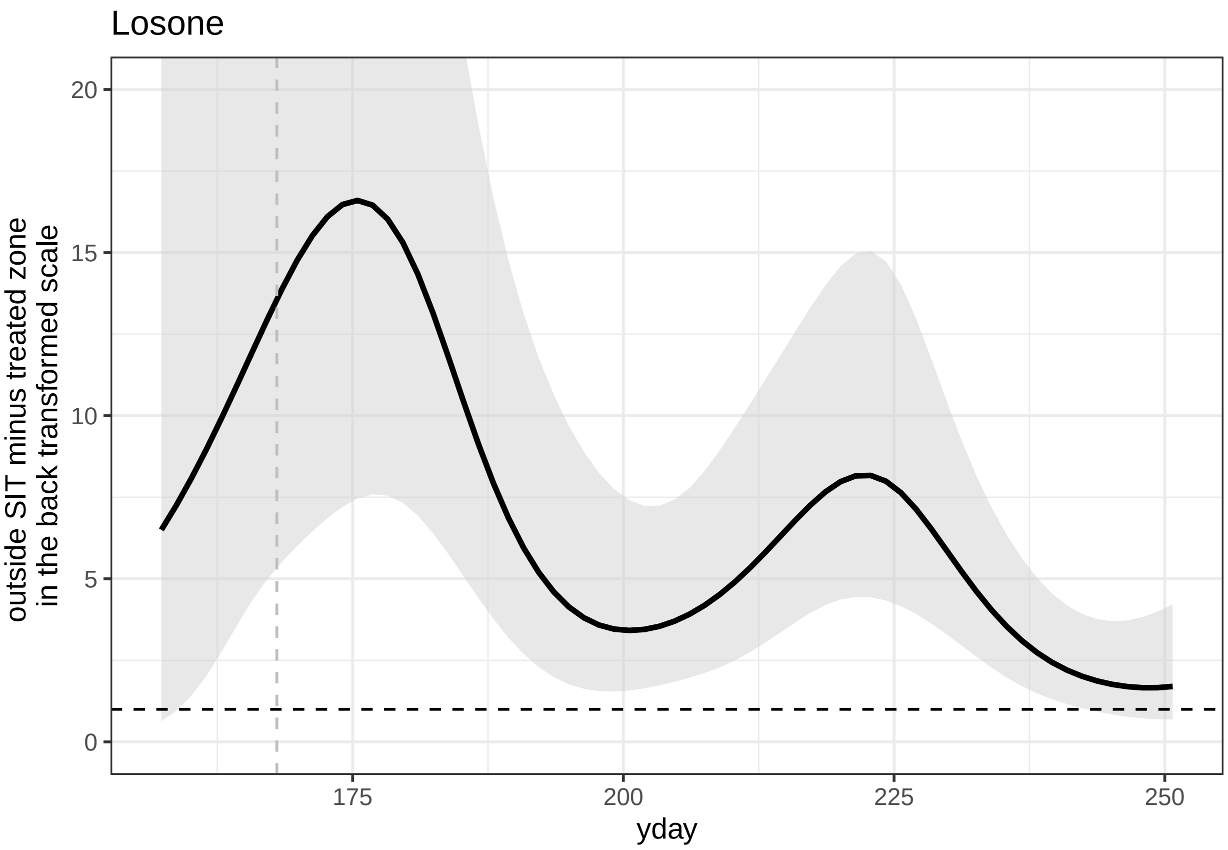

The untreated zone shows higher predicted odds of hatching compared to the treated zone. This difference increases sharply after the treatment begins, reaching a peak around day 175, when the predicted odds of hatching in the untreated zone are about `16.6` times higher than in the treated zone. The difference then decreases slightly but remains statistically significantly positive.

Note that the difference between the two zones is statistically significant when the grey zone doesn't intersect with the horizontal dashed line at 1.

5.6.1.4 outside SIT versus within SIT zone – Ascona We calculate the last contrast, comparing the treated and untreated zones in Ascona.

```
d.pred.gamm_aug.Ascona.25.SIT.outside <- d.pred.gamm_aug_pop %>%
  filter(municipality.fac == "Ascona") %>%
  filter(zona.SIT.discrete.fac %in% c("within SIT", "outside SIT")) %>%
  droplevels() %>%
  arrange(zona.SIT.discrete.fac, yday)
##
## check
d.pred.gamm_aug.Ascona.25.SIT.outside %>%
  pull(zona.SIT.discrete.fac)
```

```
 [1] within SIT  within SIT  within SIT  within SIT  within SIT  within SIT
 [7] within SIT  within SIT  within SIT  within SIT  within SIT  within SIT
[13] within SIT  within SIT  within SIT  within SIT  within SIT  within SIT
[19] within SIT  within SIT  within SIT  within SIT  within SIT  within SIT
[25] within SIT  within SIT  within SIT  within SIT  within SIT  within SIT
[31] within SIT  within SIT  within SIT  within SIT  within SIT  within SIT
```

```
 [37] within SIT  within SIT  within SIT  within SIT  within SIT  within SIT 
 [43] within SIT  within SIT  within SIT  within SIT  within SIT  within SIT 
 [49] within SIT  within SIT  within SIT  within SIT  within SIT  within SIT 
 [55] within SIT  within SIT  within SIT  within SIT  within SIT  within SIT 
 [61] within SIT  within SIT  within SIT  within SIT  within SIT  within SIT 
 [67] within SIT  within SIT  outside SIT outside SIT outside SIT outside SIT
 [73] outside SIT outside SIT outside SIT outside SIT outside SIT outside SIT
 [79] outside SIT outside SIT outside SIT outside SIT outside SIT outside SIT
 [85] outside SIT outside SIT outside SIT outside SIT outside SIT outside SIT
 [91] outside SIT outside SIT outside SIT outside SIT outside SIT outside SIT
 [97] outside SIT outside SIT outside SIT outside SIT outside SIT outside SIT
[103] outside SIT outside SIT outside SIT outside SIT outside SIT outside SIT
[109] outside SIT outside SIT outside SIT outside SIT outside SIT outside SIT
[115] outside SIT outside SIT outside SIT outside SIT outside SIT outside SIT
[121] outside SIT outside SIT outside SIT outside SIT outside SIT outside SIT
[127] outside SIT outside SIT outside SIT outside SIT outside SIT outside SIT
[133] outside SIT outside SIT outside SIT outside SIT
Levels: within SIT outside SIT
```

```r
## makes sense
```

```r
## Model matrix
W.predictions.outside.sit <- predict(gamm.hatched.Ascona,
                                     newdata = d.pred.gamm_aug.Ascona.25.SIT.outside,
                                     type = "lpmatrix",

                                     ## Prediction at population level
                                     exclude = 's(unique.ID)')

## Calculate the contrasts

## First, extract the dimension of the matrix containing only "within SIT"
n4 <- d.pred.gamm_aug.Ascona.25.SIT.outside %>%
  filter(zona.SIT.discrete.fac == "within SIT") %>%
  nrow() %>%
  print()
```

```
[1] 68
```

```r
##
## check that "outside SIT" has the same dimension
d.pred.gamm_aug.Ascona.25.SIT.outside %>%
  filter(zona.SIT.discrete.fac == "outside SIT") %>%
  nrow()
```

```
[1] 68
```

```r
## makes sense

## Calculate the estimates of the contrasts
L.outside.Ascona  <- W.predictions.outside.sit[(n4 + 1):(2*n4), , drop = FALSE] -
  W.predictions.outside.sit[1:n4, , drop = FALSE]
est.outside.Ascona <- as.vector(L.outside.Ascona %*% c.coeff.gamm.hatched.Ascona)

## Now, we calculate the corresponding standard error
se.outside.Ascona  <-
  ( (L.outside.Ascona %*% vcov.c.coeff.gamm.hatched.Ascona) * L.outside.Ascona ) %>%
```

```
  rowSums() %>%
  sqrt()

d.diff.outside.Ascona <- data.frame(
  yday = d.pred.gamm_aug.Ascona$yday[1:n4],
  diff = est.outside.Ascona,
  lo = est.outside.Ascona - qnorm(0.975) * se.outside.Ascona,
  hi = est.outside.Ascona + qnorm(0.975) * se.outside.Ascona
) %>%
  mutate(exp.diff = exp(diff),
         exp.lo = exp(lo),
         exp.hi = exp(hi))
```

We plot the result.

```
ggplot(d.diff.outside.Ascona, mapping = aes(x = yday, y = diff)) +
  geom_ribbon(aes(ymin = lo, ymax = hi), fill = "lightgray", alpha = 0.5) +
  geom_line(linewidth = 1) +
  geom_hline(yintercept = 0, linetype = "dashed") +
  geom_vline(xintercept = yday("2025-06-17"), linetype = "dashed", colour = "grey") +
  labs(title = "Ascona",
       y = "outside SIT minus treated zone")
```

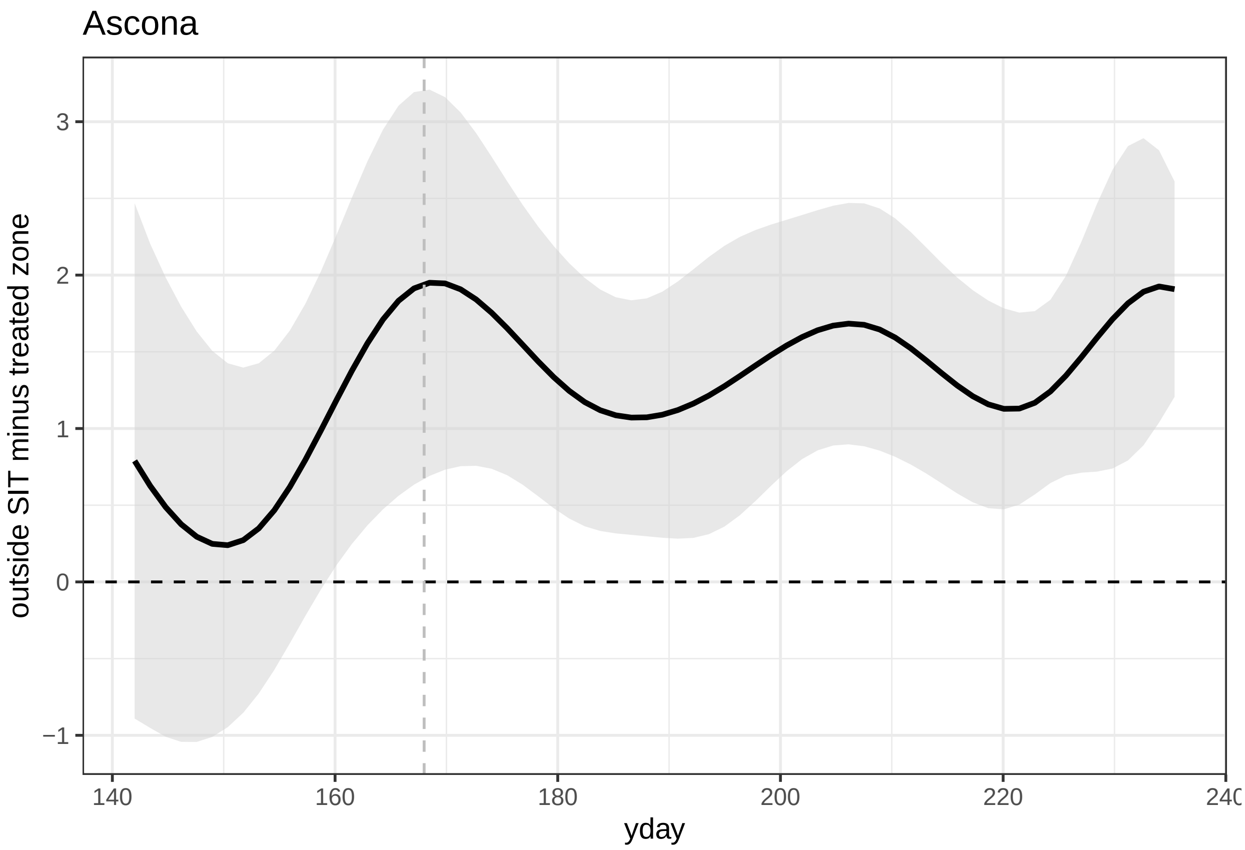

The plot shows the difference between the predicted values on the linear predictor scale for the untreated and treated zones, along with a 95% confidence interval. A horizontal dashed line at 0 indicates no difference between the two zones, while a vertical dashed line marks the start of the treatment on June 17, 2025.

We back-transform the difference by applying the exponential function, which returns the values to the odds

scale. After this transformation, the values can be interpreted as ratios of predicted odds of hatching: a value of 1 indicates no difference between zones, values greater than 1 indicate higher predicted odds of hatching in the untreated zone relative to the treated zone, and values below 1 indicate the opposite.

```
gg.outside.Ascona <- ggplot(d.diff.outside.Ascona, mapping = aes(x = yday, y = exp.diff)) +
  geom_ribbon(aes(ymin = exp.lo, ymax = exp.hi), fill = "lightgray", alpha = 0.5) +
  geom_line(linewidth = 1) +
  geom_hline(yintercept = 1, linetype = "dashed") +
  geom_vline(xintercept = yday("2025-06-17"), linetype = "dashed", colour = "grey") +
  labs(title = "Ascona",
       y = "outside SIT minus treated zone \nin the back transformed scale")
```

We zoom in to focus on the most relevant part of the graph. We use the same zoom as for the previous plots to facilitate comparison.

```
## we zoom in
gg.outside.Ascona +
  coord_cartesian(ylim = c(0, 20))
```

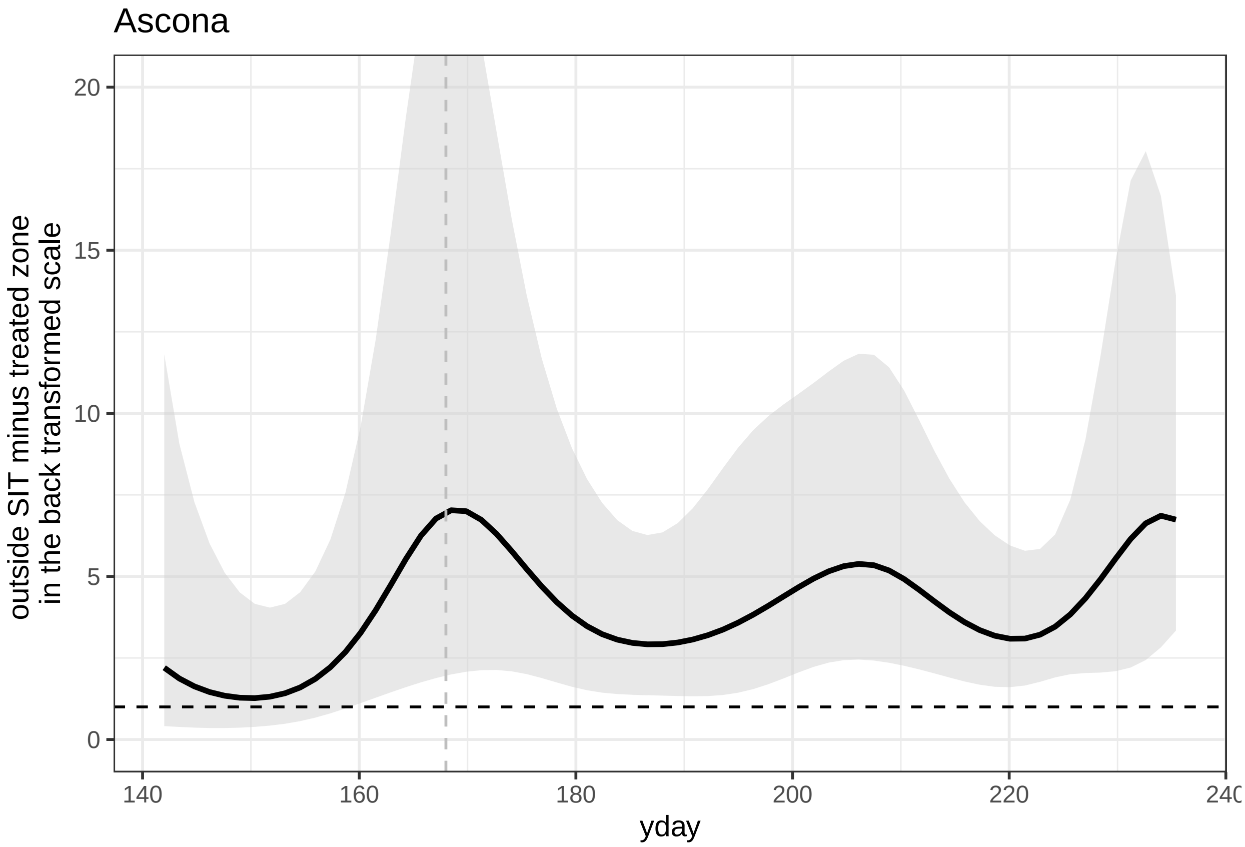

We observe that the predicted odds of hatching in the two zones start at similar levels. After the treatment begins, the difference increases, with higher predicted odds in the untreated zone. This difference reaches its peak around day 180 and then stabilises. At that point, the predicted odds of hatching in the untreated zone are about `7` times higher than in the treated zone.

Note that the difference between the two zones is statistically significant when the grey zone doesn't intersect with the horizontal dashed line at 1.

5.7 Residual analysis

First of all, we apply the `gam.check()` function to the model, which produces some diagnostic information.

```
par(mfrow = c(2, 2))
gam.check(gamm.hatched.Losone)
```

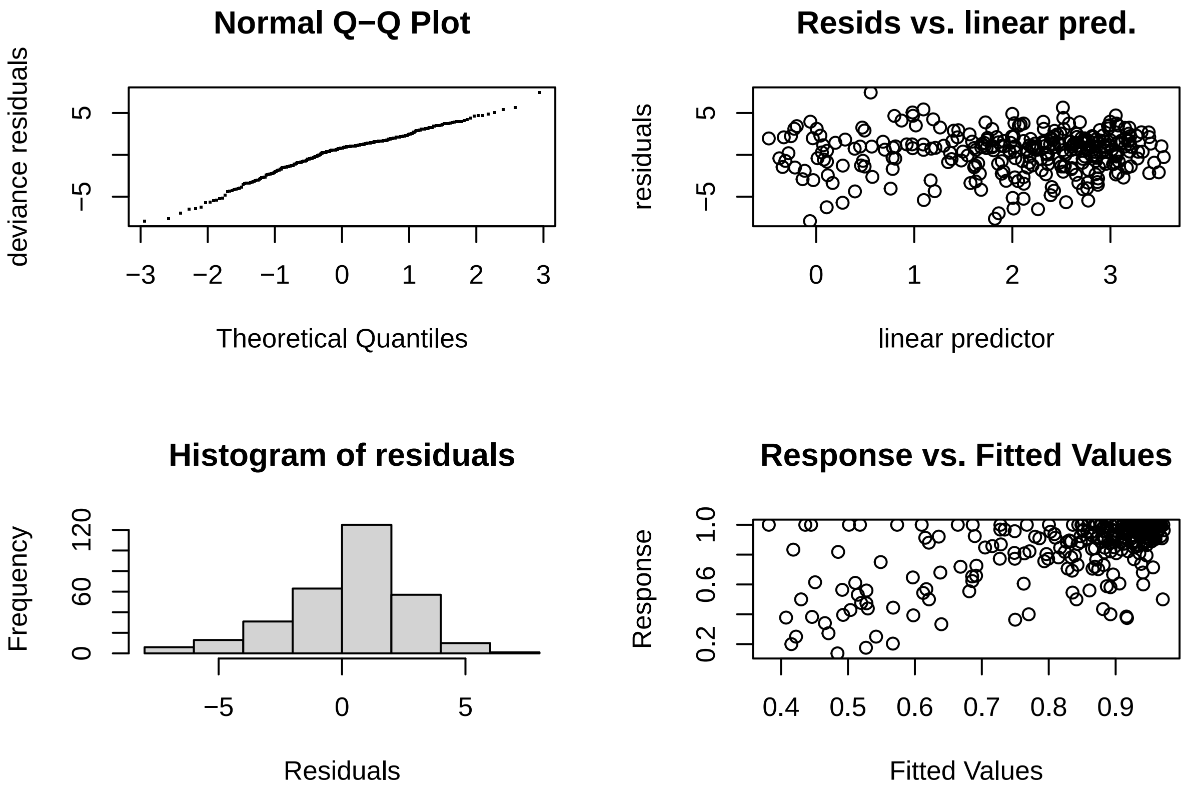


```
Method: GCV   Optimizer: outer newton
full convergence after 8 iterations.
Gradient range [-0.00000494944192659815,0.000000982515895753266]
(score 8.30384729265633 & scale 7.01729245934651).
Hessian positive definite, eigenvalue range [0.0000049486997049877,0.288149928167348].
Model rank =  78 / 78

Basis dimension (k) checking results. Low p-value (k-index<1) may
indicate that k is too low, especially if edf is close to k'.

                                         k'   edf k-index p-value
s(unique.ID)                          48.00 23.51      NA      NA
s(yday):zona.SIT.discrete.facwithin SIT  9.00  6.93    1.04    0.80
s(yday):zona.SIT.discrete.facbuffer      9.00  7.60    1.04    0.77
s(yday):zona.SIT.discrete.facoutside SIT  9.00  1.00    1.04    0.83
```

```
par(mfrow = c(1, 1))

par(mfrow = c(2, 2))
gam.check(gamm.hatched.Ascona)
```

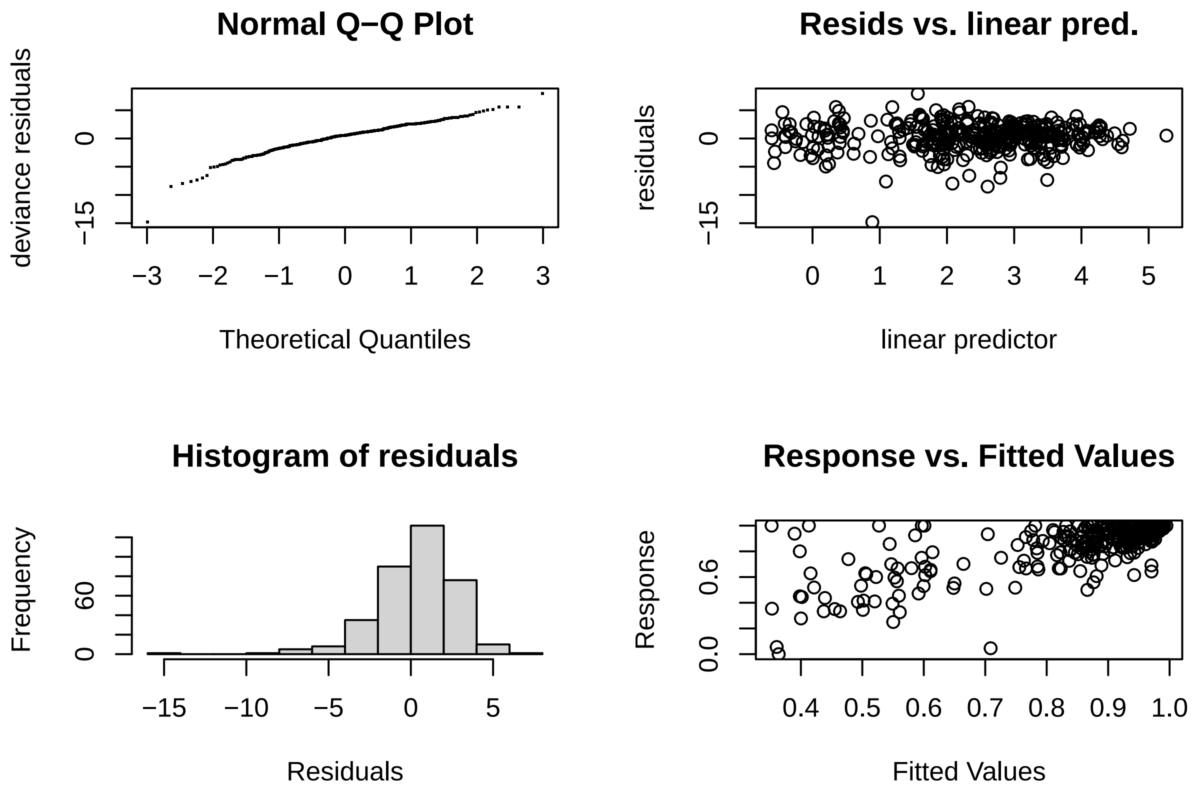


```
Method: GCV   Optimizer: outer newton
full convergence after 18 iterations.
Gradient range [-0.000000104830737535089,0.000000630915116664466]
(score 8.41661831152048 & scale 7.65701971448096).
Hessian positive definite, eigenvalue range [0.00532323756507765,0.284360953137854].
Model rank =  82 / 82

Basis dimension (k) checking results. Low p-value (k-index<1) may
indicate that k is too low, especially if edf is close to k'.

                                           k'   edf k-index p-value
s(unique.ID)                            52.00 23.79      NA      NA
s(yday):zona.SIT.discrete.facwithin SIT  9.00  8.32    1.01    0.67
s(yday):zona.SIT.discrete.facbuffer      9.00  8.79    1.01    0.70
s(yday):zona.SIT.discrete.facoutside SIT  9.00  6.86    1.01    0.62
```

```
par(mfrow = c(1, 1))
```

Then, we store the pearson residuals in the original data frame, and we plot the residuals against the fitted values to see whether there is still structure in the data.

```
d.hatching.Losone$resid_gamm.tot.eggs <- resid(gamm.hatched.Losone,
                                               type = "pearson")
##
ggplot(data = d.hatching.Losone,
       mapping = aes(y = resid_gamm.tot.eggs,
                     x = fitted_gamm.hatching)) +
  geom_hline(yintercept = 0) +
  geom_point(alpha = 0.2) +
```

```
  geom_smooth()
```

```
`geom_smooth()` using method = 'loess' and formula = 'y ~ x'
```

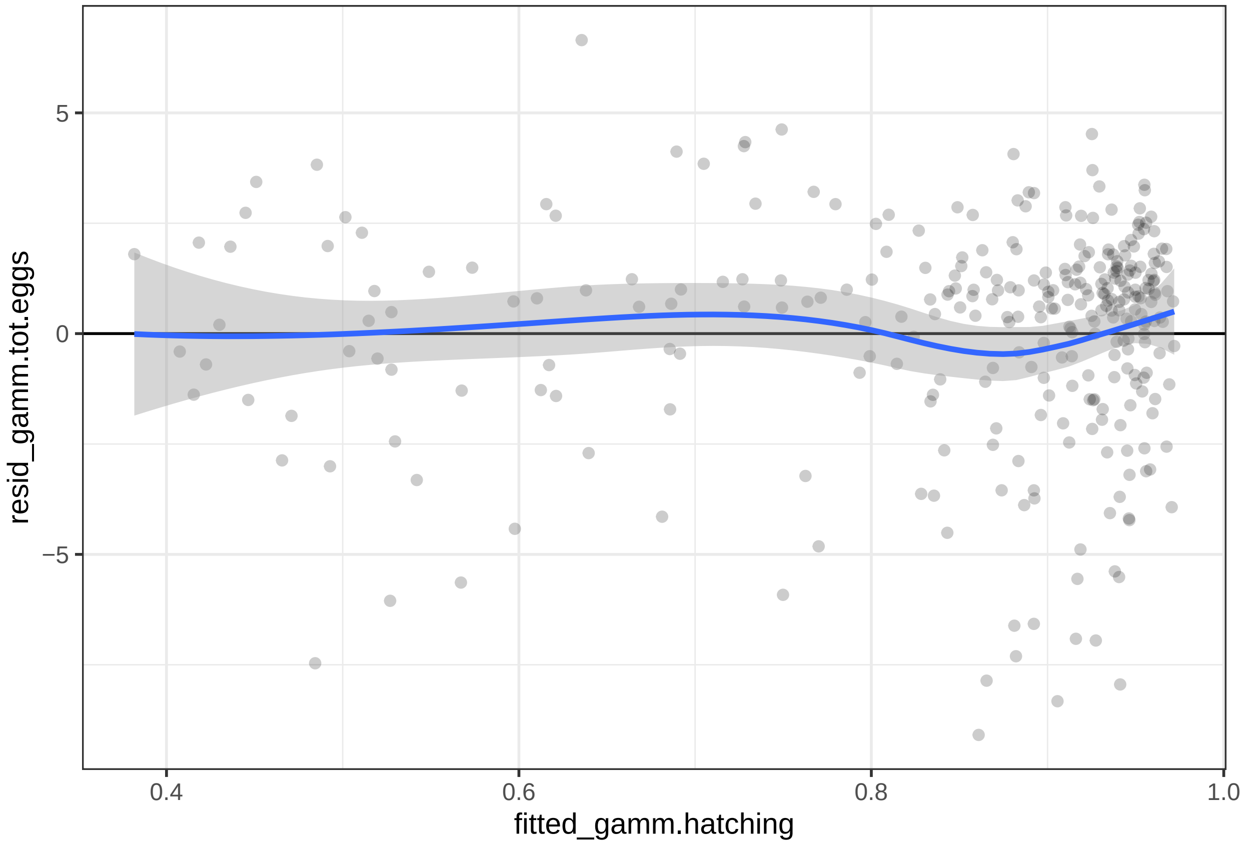

The blue line is located on the x-axis and it is quite straight, indicating that there doesn't seem to be much structure left in the residuals.

```
d.hatching.Ascona$resid_gamm.tot.eggs <- resid(gamm.hatched.Ascona,
                                               type = "pearson")
##
ggplot(data = d.hatching.Ascona,
       mapping = aes(y = resid_gamm.tot.eggs,
                     x = fitted_gamm.hatching)) +
  geom_hline(yintercept = 0) +
  geom_point(alpha = 0.2) +
  geom_smooth()
```

```
`geom_smooth()` using method = 'loess' and formula = 'y ~ x'
```

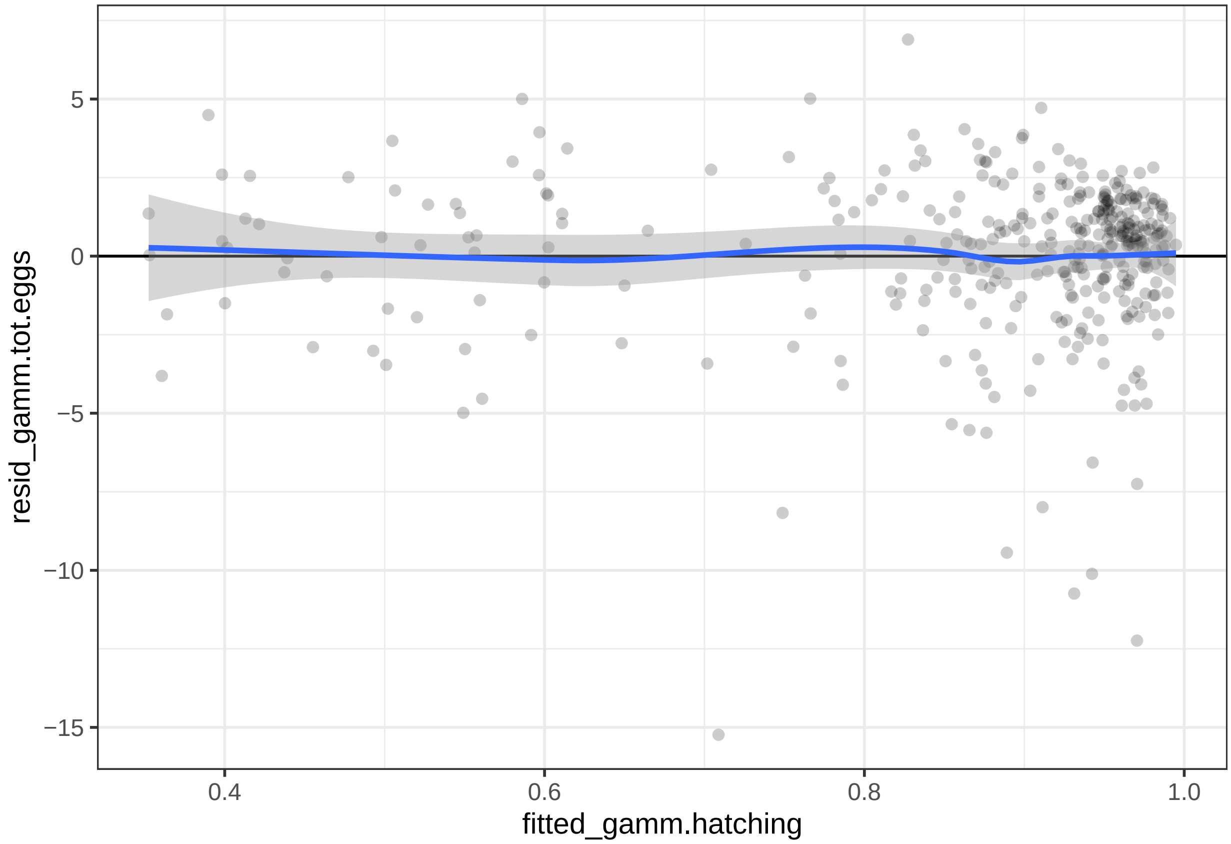

The blue line is not located far from the x-axis and it is quite straight, indicating that there doesn't seem to be much structure left in the residuals.

We plot the qq-plot for the random effects.

```
## QQ for random effects
plot(sm(gamm.hatched.Losone, 1))
```

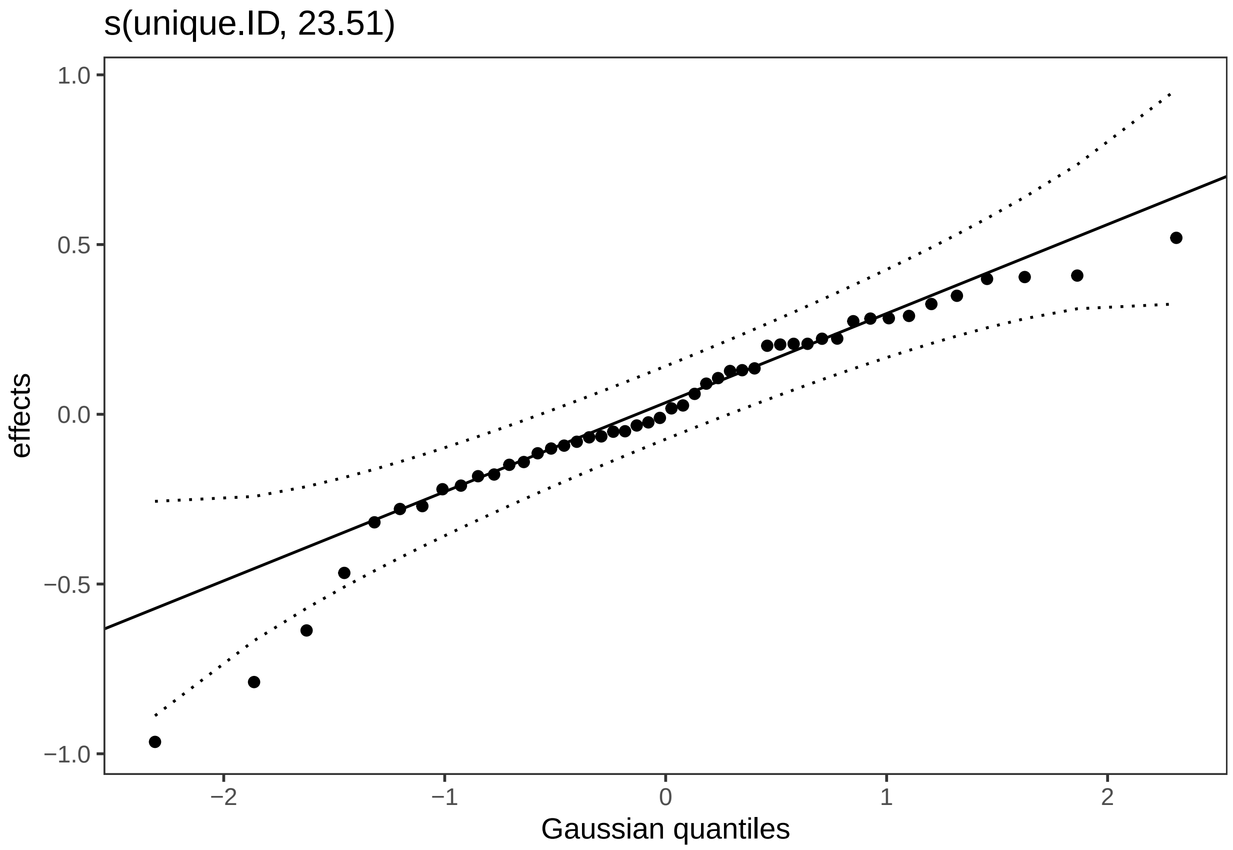


```
##
plot(sm(gamm.hatched.Ascona, 1))
```

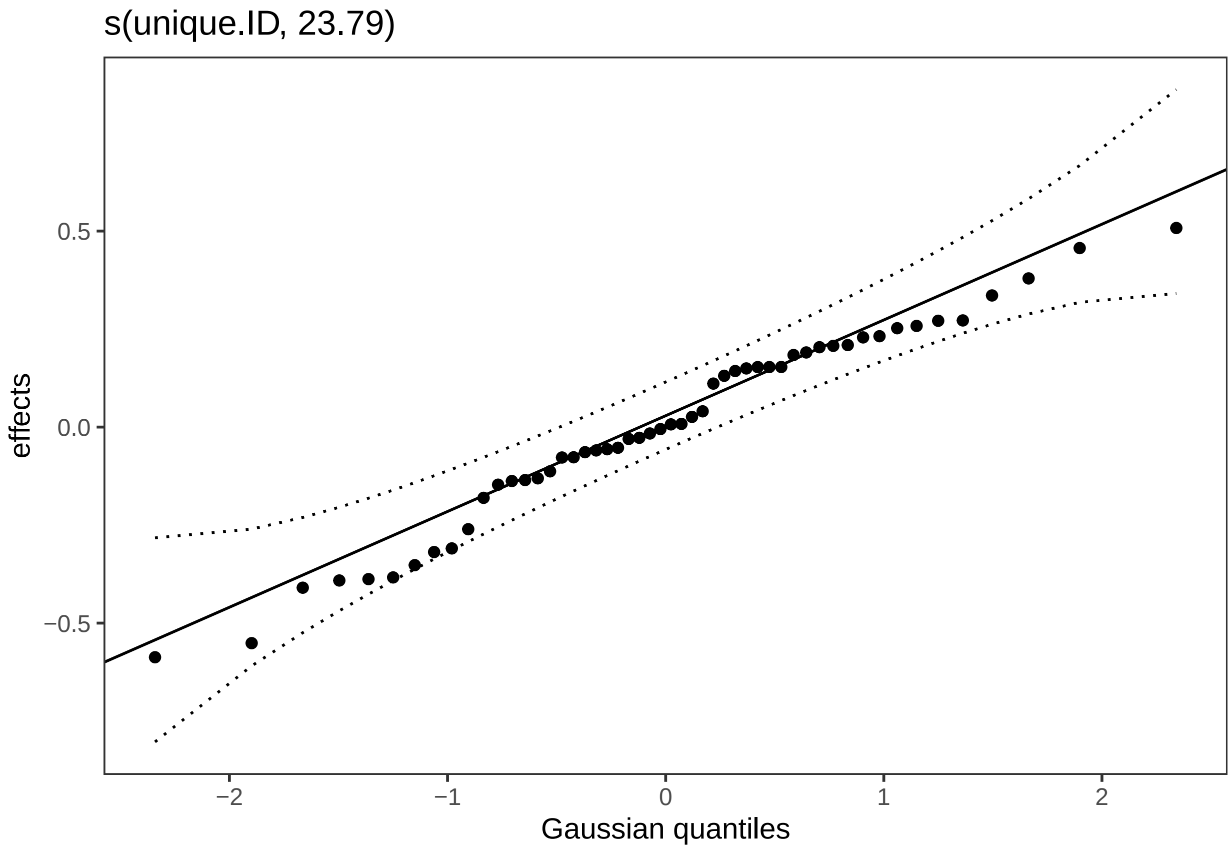

There doesn't seem to be a significant departure from the normality assumption for either models.

We now plot the residuals over time to further check whether there is any structure left in the data.

```
ggplot(data = d.hatching.Losone,
       mapping = aes(y = resid_gamm.tot.eggs,
                     x = yday)) +
  geom_hline(yintercept = 0) +
  geom_point(alpha = 0.2) +
  geom_smooth(method = "loess")
```

```
`geom_smooth()` using formula = 'y ~ x'
```

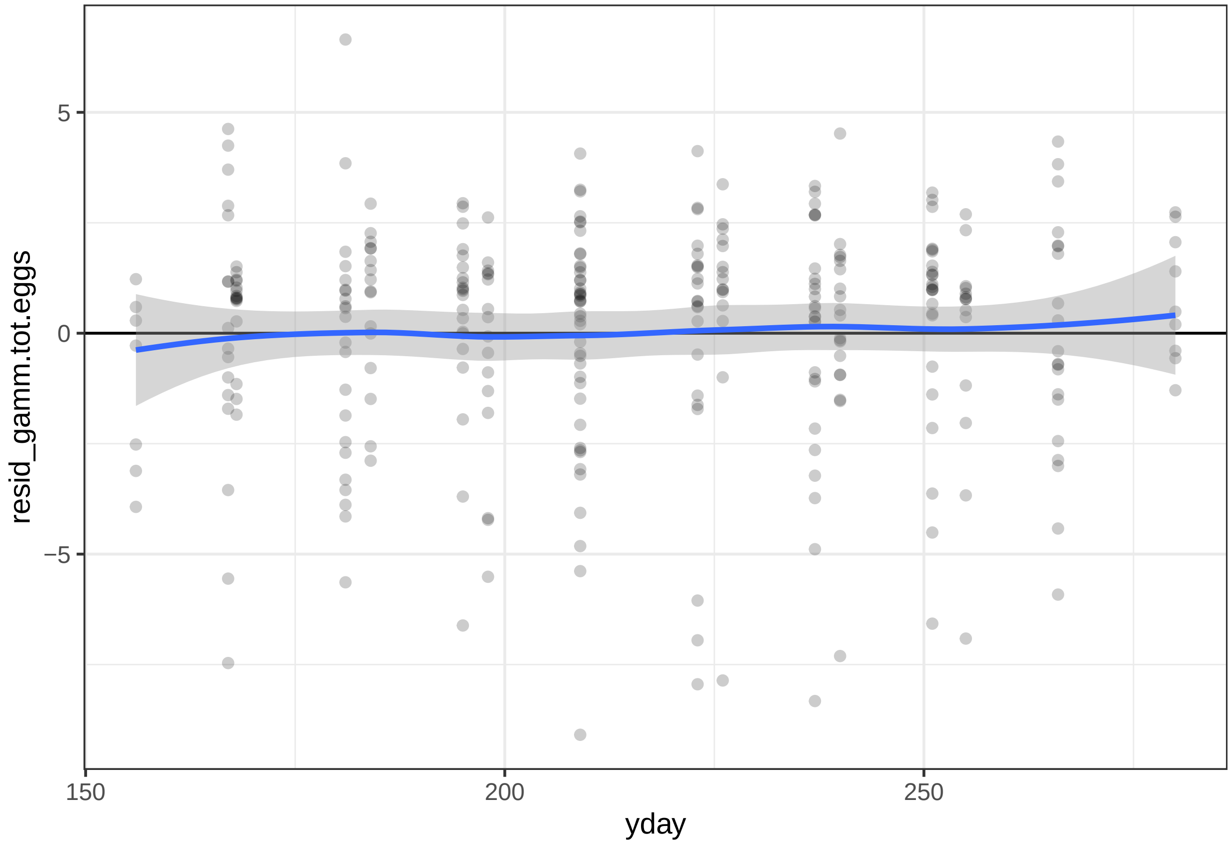


```
##
ggplot(data = d.hatching.Ascona,
       mapping = aes(y = resid_gamm.tot.eggs,
                     x = yday)) +
  geom_hline(yintercept = 0) +
  geom_point(alpha = 0.2) +
  geom_smooth(method = "loess")
```

```
`geom_smooth()` using formula = 'y ~ x'
```

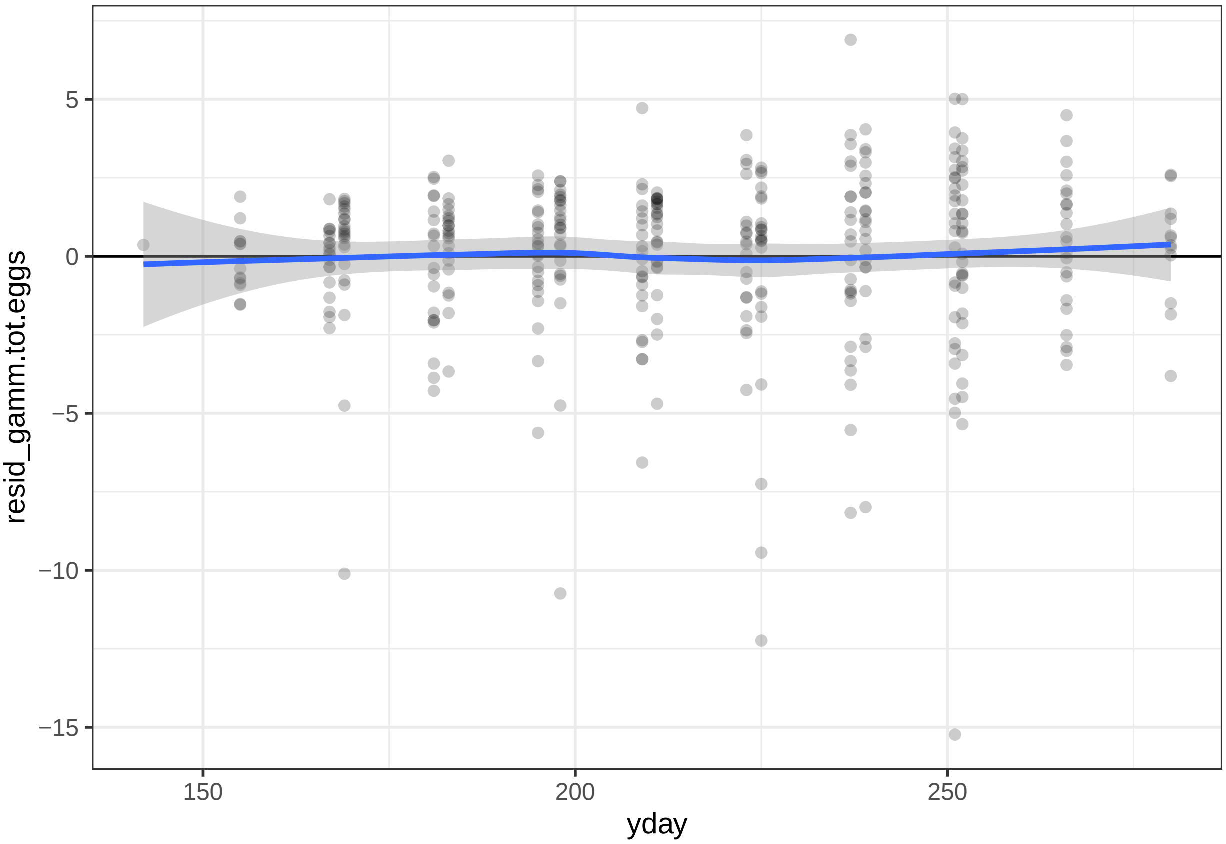

There does not seem to be any structure left in the data.

The following two plots display the residuals in Losone and Ascona, respectively.
The observations belonging to the same ovitrap are connected with a line. These plots are additionally drawn to check temporal correlation and variability.

```
## for Losone
ggplot(data = d.hatching.Losone,
       mapping = aes(y = resid_gamm.tot.eggs,
                     x = yday,
                     group = unique.ID)) +
  geom_hline(yintercept = 0) +
  geom_point(alpha = 0.2) +
  geom_line(alpha = 0.2) +
  labs(title = "Losone")
```

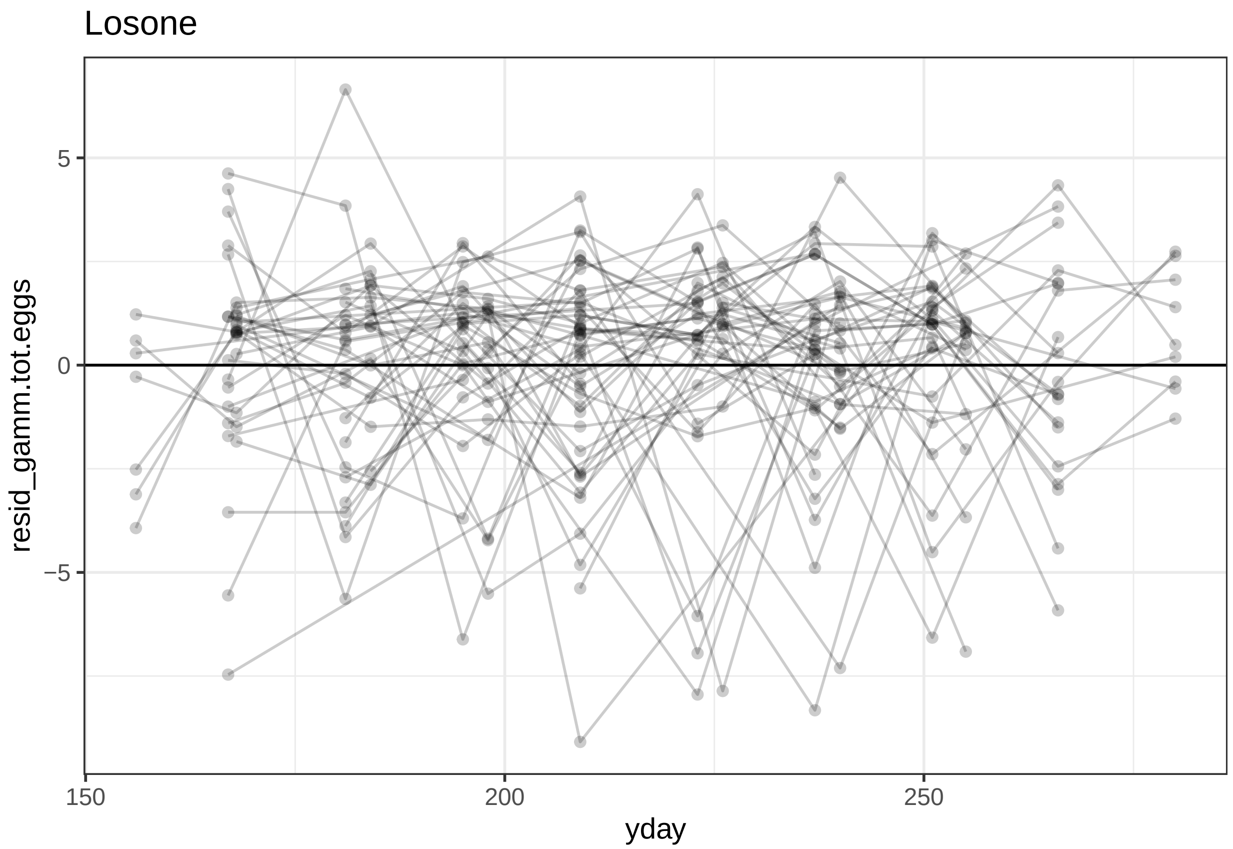


```
##
## For Ascona
ggplot(data = d.hatching.Ascona,
       mapping = aes(y = resid_gamm.tot.eggs,
                     x = yday,
                     group = unique.ID)) +
  geom_hline(yintercept = 0) +
  geom_point(alpha = 0.2) +
  geom_line(alpha = 0.2) +
  labs(title = "Ascona")
```

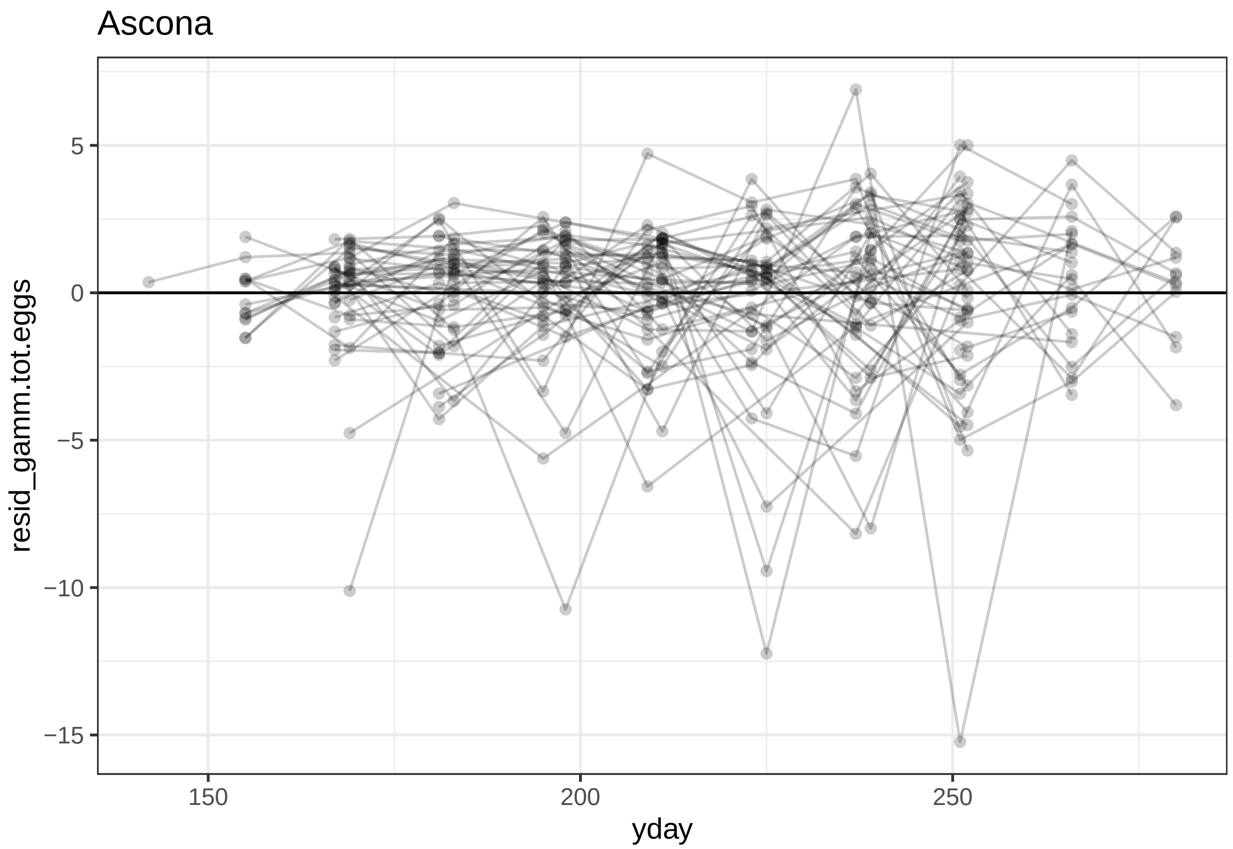

There doesn't seem to be any structure left in the data.

Let's look at the single ovitraps.

```
## for Losone
ggplot(data = d.hatching.Losone,
       mapping = aes(y = resid_gamm.tot.eggs,
                     x = yday,
                     group = unique.ID)) +
  geom_hline(yintercept = 0) +
  geom_point() +
  geom_line() +
  facet_wrap(~ unique.ID) +
  theme(
    strip.background = element_blank(),
    strip.text.x = element_blank()) +
  coord_cartesian(ylim = c(-4,4)) +
  labs(title = "Losone")
```

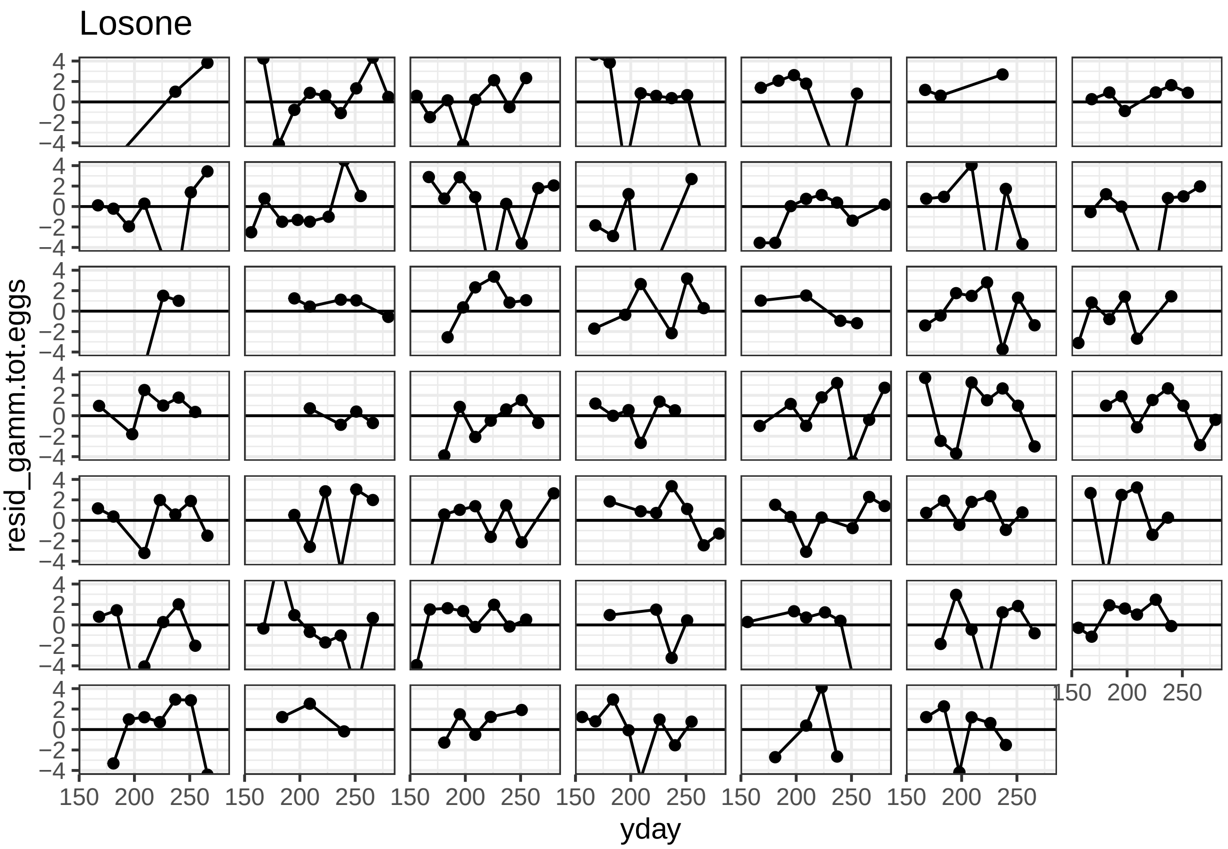


```
##
## for Ascona
ggplot(data = filter(d.hatching.Ascona,
                     municipality.fac == "Ascona"),
       mapping = aes(y = resid_gamm.tot.eggs,
                     x = yday,
                     group = unique.ID)) +
  geom_hline(yintercept = 0) +
  geom_point() +
  geom_line() +
  facet_wrap(~ unique.ID) +
  theme(
    strip.background = element_blank(),
    strip.text.x = element_blank()) +
  coord_cartesian(ylim = c(-4, 4)) +
  labs(title = "Ascona")
```

```
`geom_line()`: Each group consists of only one observation.
i Do you need to adjust the group aesthetic?
```

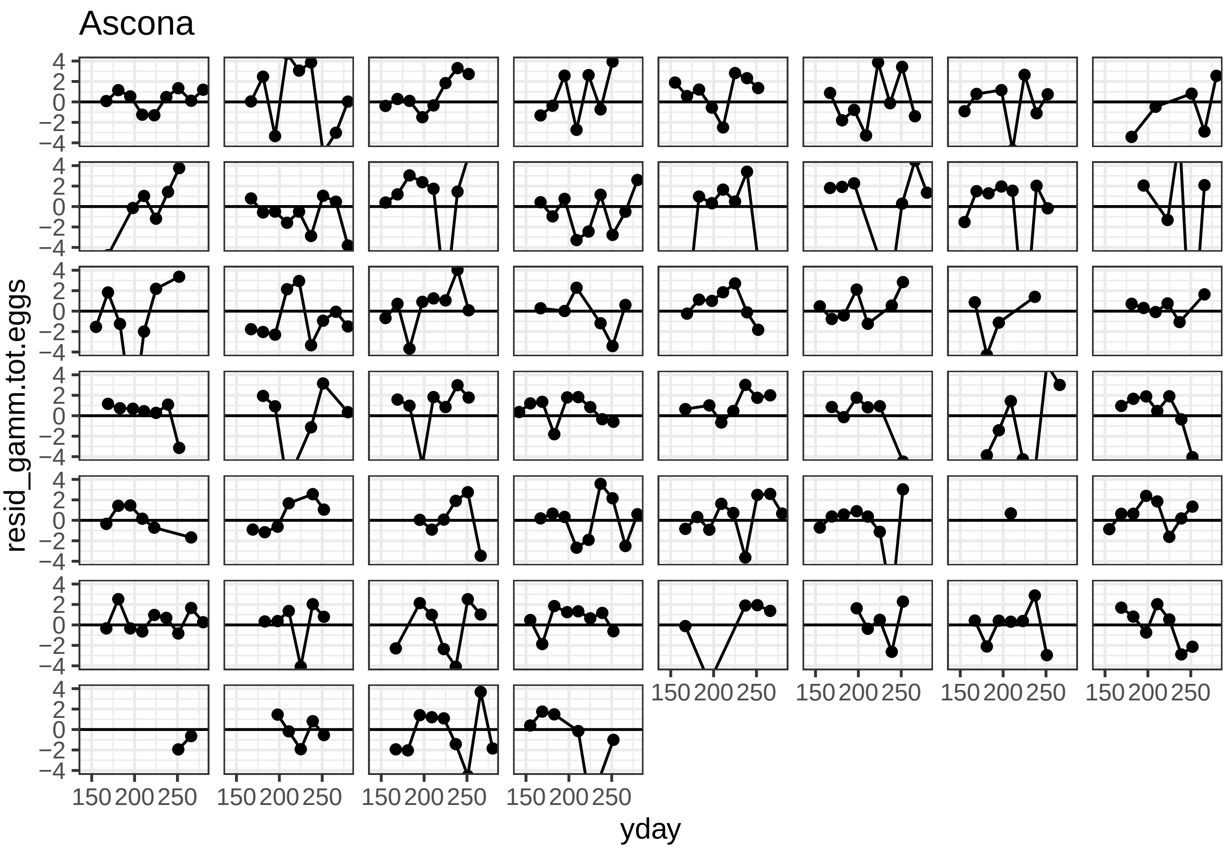

In both cases, there does not seem to be much structure left in the data.

6 Spatial Generalised Additive Model (spatial GAM)

We are now interested in testing whether geographical location plays a role in determining hatching rates.

6.1 Visualising the data

Let us first plot the geographical distribution of the mean hatching rates in Ascona and Losone for the year 2025. To achieve this, we first calculate the average hatching rates over the season.

```
d.hatching.agg <- d.hatching %>%
  group_by(unique.ID) %>%
  summarise(mean.hatched = mean(Perc.egg.hatch.per.trap,
                                na.rm = TRUE),
            X.num = first(X.num),
            Y.num = first(Y.num),
            municipality.fac = first(municipality.fac))
d.hatching.agg %>%
  head(n = 10)
```

```
# A tibble: 10 x 5
   unique.ID  mean.hatched   X.num   Y.num municipality.fac
   <fct>             <dbl>   <dbl>   <dbl> <fct>
 1 Ascona.1           85.3 703393. 112214. Ascona
 2 Ascona.10          73.7 703328  111998. Ascona
 3 Ascona.10b         94.9 703474  112351  Ascona
 4 Ascona.11          91.7 703434. 111909. Ascona
 5 Ascona.11a         98.0 703865. 112454. Ascona
 6 Ascona.12          83.3 703555. 111948. Ascona
 7 Ascona.12a         93.4 704428. 111666. Ascona
 8 Ascona.13          68.9 703282. 111965. Ascona
 9 Ascona.13b         95.7 702936  112083  Ascona
10 Ascona.14          70.9 703275. 112091. Ascona
```

We mark the center of the treated zone with a cross.

```
d.coord.center.losone <- data.frame(X = 2702682.04, Y = 1113837.30) %>%
  st_as_sf(
    coords = c("X", "Y"),
    crs = 2056,
    remove = FALSE) %>%
  st_transform(crs = 21781) %>%
  st_coordinates() %>%
  as.data.frame()
d.coord.center.ascona <- data.frame(X = 2703480.39, Y = 1112071.09) %>%
  st_as_sf(
    coords = c("X", "Y"),
    crs = 2056,
    remove = FALSE) %>%
  st_transform(crs = 21781) %>%
  st_coordinates() %>%
  as.data.frame()
```

```
## (warnings are omitted from this chunk)
##
## Create two separate data sets, one for each municipality
```

```r
d.hatching.agg.Ascona.25 <- d.hatching.agg %>%
  filter(municipality.fac == "Ascona")
##
d.hatching.agg.Losone.25 <- d.hatching.agg %>%
  filter(municipality.fac =="Losone")
##
## We calculate the min and max for the median hatched eggs.
## This is used in the plots, to maintain the same colour scale for the two
## municipalities and compare them more easily.
max.hatch.mean.25 <- max(c(d.hatching.agg.Ascona.25$mean.hatched,
                           d.hatching.agg.Losone.25$mean.hatched))
##
min.hatch.mean.25 <- min(c(d.hatching.agg.Ascona.25$mean.hatched,
                           d.hatching.agg.Losone.25$mean.hatched))
```

```r
## Plot Losone
gg.obs.L.25 <- ggplot(d.hatching.agg.Losone.25,
                      mapping = aes(y = Y.num,
                                    x = X.num,
                                    colour = mean.hatched)) +
  geom_point(size = 3) +
  geom_point(d.coord.center.losone, mapping = aes(x = X, y = Y),
             colour = "black", shape = 4, size = 4) +
  scale_color_gradientn(colours = c("blue", "gold"),
                        values = scales::rescale(c(min.hatch.mean.25,
                                                   max.hatch.mean.25)),
                        limits = c(min.hatch.mean.25, max.hatch.mean.25)) +
  # theme(aspect.ratio = 1) +
  coord_fixed() +
  labs(title = "Losone")
gg.obs.L.25
```

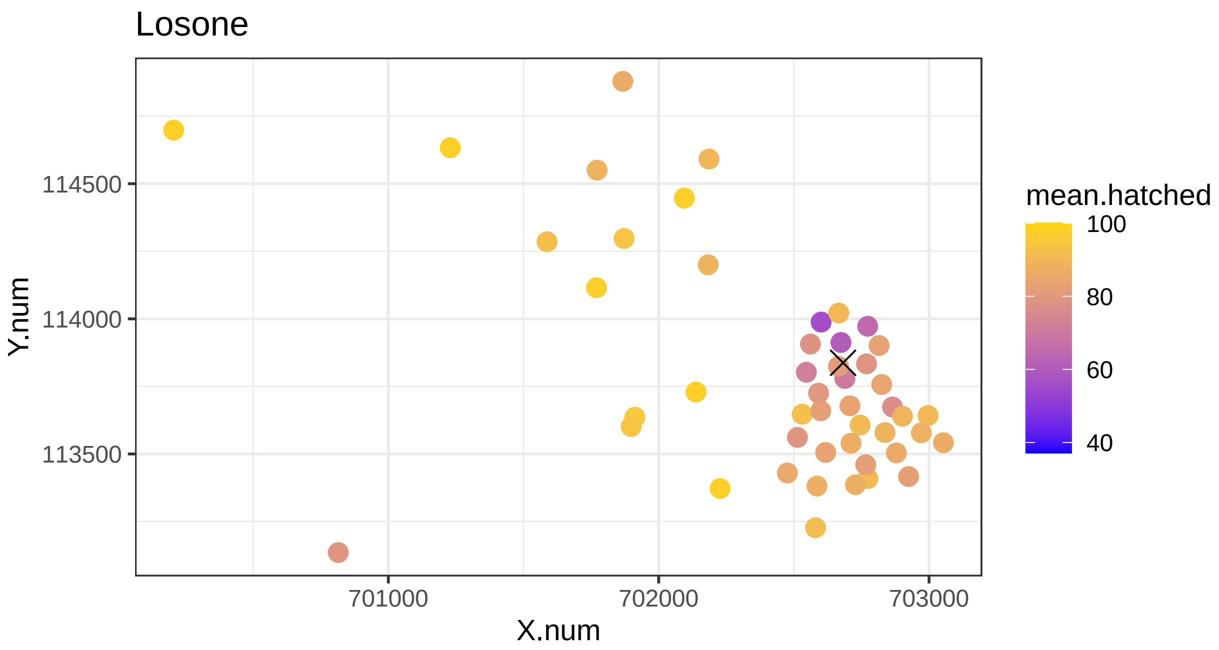

The spatial pattern is quite clear: closer to the treated zone, there seems to be a lower mean of hatching

rates, whereas further away from it the rates seem to be higher.

The treated area is very small compared to the entire surface of the municipality. Therefore, we create a second data set where we focus on this area.

```
d.hatching.agg.Losone.25.zoom <- d.hatching.agg.Losone.25 %>%
  filter(## ovitraps with letters after the number are not part of the SIT
    ## design, thus we drop them.
    ## (see "istruzioni tabella ASC-LOS.docx")
    str_detect(unique.ID, pattern = "[a-zA-Z]\\.\\d+[a-z]", negate = TRUE)) %>%
  droplevels()
##
## check
unique(d.hatching.agg.Losone.25.zoom$unique.ID)
```

```
 [1] Losone.1  Losone.10 Losone.11 Losone.12 Losone.13 Losone.14 Losone.15
 [8] Losone.16 Losone.17 Losone.18 Losone.19 Losone.2  Losone.20 Losone.21
[15] Losone.22 Losone.23 Losone.24 Losone.25 Losone.26 Losone.27 Losone.28
[22] Losone.3  Losone.4  Losone.5  Losone.6  Losone.7  Losone.8  Losone.9
28 Levels: Losone.1 Losone.10 Losone.11 Losone.12 Losone.13 ... Losone.9
```

```
## makes sense
##
## Set min / max for colour scale
max.hatch.mean.25.zoom.Losone <- max(d.hatching.agg.Losone.25.zoom$mean.hatched)
min.hatch.mean.25.zoom.Losone <- min(d.hatching.agg.Losone.25.zoom$mean.hatched)
```

```
gg.obs.L.25_zoom <- ggplot(d.hatching.agg.Losone.25.zoom,
                           mapping = aes(y = Y.num,
                                         x = X.num,
                                         colour = mean.hatched)) +
  geom_point(size = 3) +
  geom_point(d.coord.center.losone, mapping = aes(x = X, y = Y),
             colour = "black", shape = 4, size = 4) +
  scale_color_gradientn(colours = c("blue", "gold"),
                        values = scales::rescale(c(min.hatch.mean.25.zoom.Losone,
                                                   max.hatch.mean.25.zoom.Losone)),
                        limits = c(min.hatch.mean.25.zoom.Losone,
                                   max.hatch.mean.25.zoom.Losone)) +
  # theme(aspect.ratio = 1) +
  coord_fixed() +
  labs(title = "Losone")
gg.obs.L.25_zoom
```

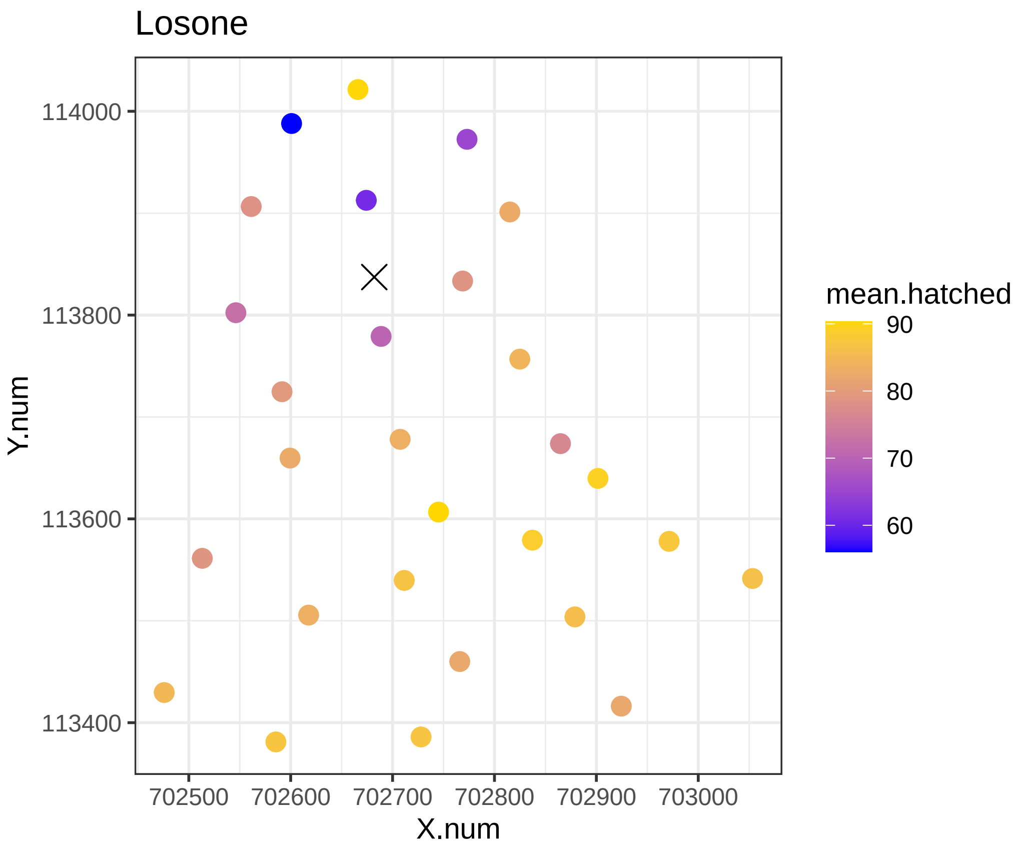

The spatial pattern is clear: closer to the treated zone, there seems to be a lower mean of hatching rates, whereas further away from it the rates seem to be higher.

We draw the same plots for Ascona.

```
## (warnings are omitted from this chunk)
##
gg.obs.A.25 <- ggplot(d.hatching.agg.Ascona.25,
                      mapping = aes(y = Y.num,
                                    x = X.num,
                                    colour = mean.hatched)) +
  geom_point(size = 3) +
  geom_point(d.coord.center.ascona, mapping = aes(x = X, y = Y),
             colour = "black", shape = 4, size = 4) +
  scale_color_gradientn(colours = c("blue", "gold"),
                        values = scales::rescale(c(min.hatch.mean.25,
                                                   max.hatch.mean.25)),
                        limits = c(min.hatch.mean.25, max.hatch.mean.25)) +
  # theme(aspect.ratio = 1) +
  coord_fixed() +
  labs(title = "Ascona")
gg.obs.A.25
```

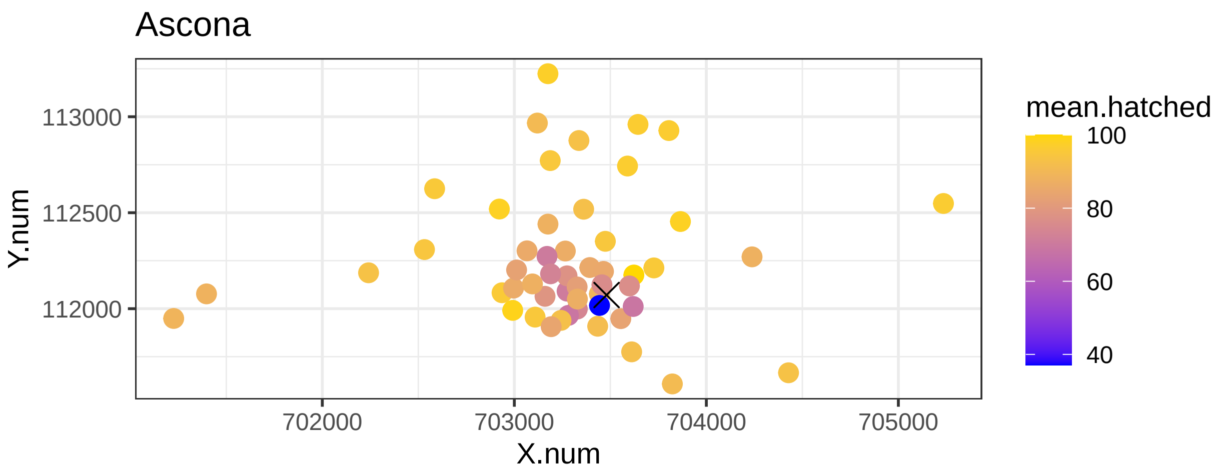

Here as well we notice a similar patter: closer to the treatment center, the mean hatching rates seem to be lower, whereas further away from it the rates seem to be higher.

Let's zoom in again in the treated zone and its neighbour area.

```
d.hatching.agg.Ascona.25.zoom <- d.hatching.agg.Ascona.25 %>%
  filter(## ovitraps with letters after the number are not part of the SIT
    ## design, thus we drop them.
    ## (see "istruzioni tabella ASC-LOS.docx")
    str_detect(unique.ID, pattern = "[a-zA-Z]\\.\\d+[a-z]", negate = TRUE)) %>%
  droplevels()
##
## check
unique(d.hatching.agg.Ascona.25.zoom$unique.ID)
```

```
 [1] Ascona.1  Ascona.10 Ascona.11 Ascona.12 Ascona.13 Ascona.14 Ascona.15
 [8] Ascona.16 Ascona.17 Ascona.18 Ascona.19 Ascona.2  Ascona.20 Ascona.21
[15] Ascona.23 Ascona.24 Ascona.25 Ascona.26 Ascona.27 Ascona.28 Ascona.3
[22] Ascona.4  Ascona.5  Ascona.6  Ascona.7  Ascona.8  Ascona.9
27 Levels: Ascona.1 Ascona.10 Ascona.11 Ascona.12 Ascona.13 ... Ascona.9
```

```
## makes sense
##
## Set min / max for colour scale
max.hatch.mean.25.zoom.Ascona <- max(d.hatching.agg.Ascona.25.zoom$mean.hatched)
min.hatch.mean.25.zoom.Ascona <- min(d.hatching.agg.Ascona.25.zoom$mean.hatched)
```

```
gg.obs.A.25_zoom <- ggplot(d.hatching.agg.Ascona.25.zoom,
                           mapping = aes(y = Y.num,
                                         x = X.num,
                                         colour = mean.hatched)) +
  geom_point(size = 3) +
  geom_point(d.coord.center.ascona, mapping = aes(x = X, y = Y),
             colour = "black", shape = 4, size = 4) +
  scale_color_gradientn(colours = c("blue", "gold"),
                        values = scales::rescale(c(min.hatch.mean.25.zoom.Ascona,
                                                   max.hatch.mean.25.zoom.Ascona)),
                        limits = c(min.hatch.mean.25.zoom.Ascona,
                                   max.hatch.mean.25.zoom.Ascona)) +
  # theme(aspect.ratio = 1) +
```

```
  coord_fixed() +
  labs(title = "Ascona")
gg.obs.A.25_zoom
```

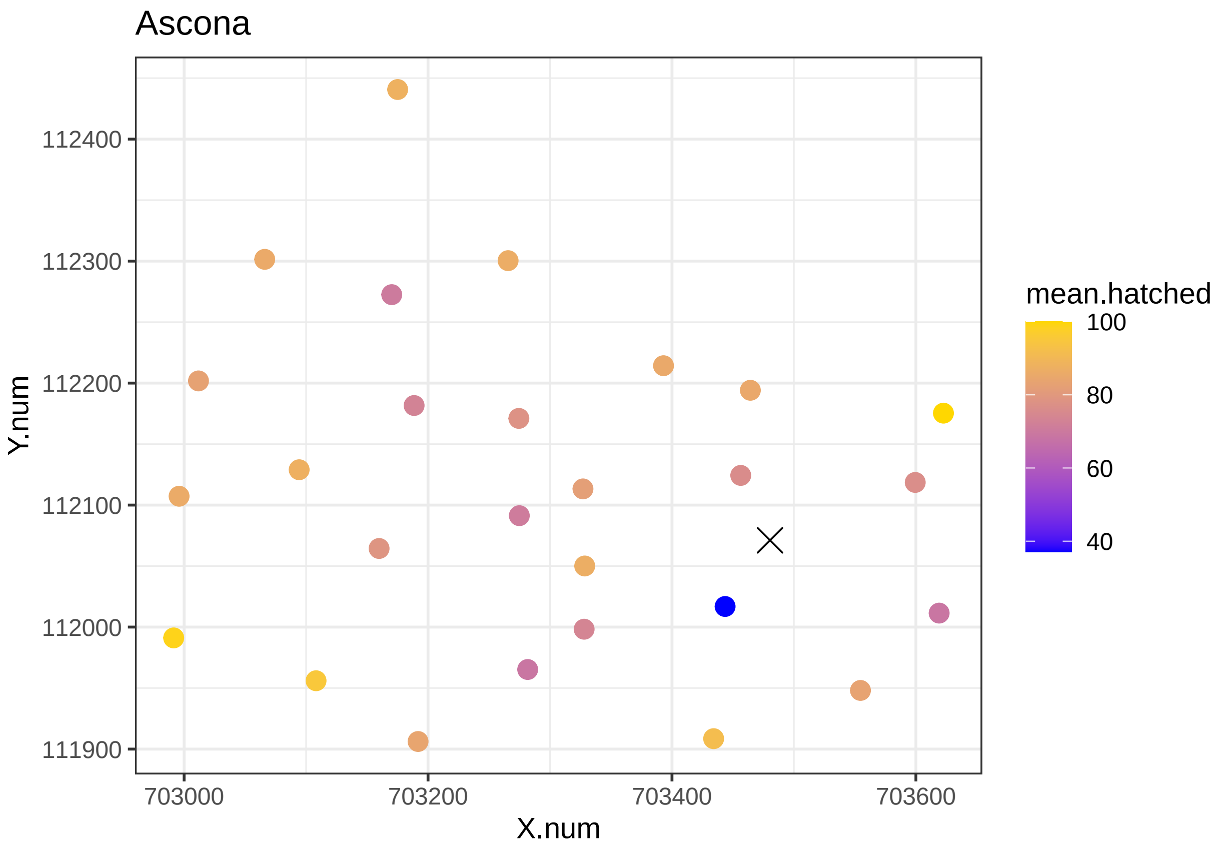

This plot is less clear than the previous one. This is because hatching rates appear to be quite low everywhere, so the contrast between the different zones is no longer visible.

6.2 Fitting the models

We create two data sets containing only Ascona and Losone for the year 2025, respectively.

```
d.hatching %>%
  select(Perc.egg.hatch.per.trap, hatched, non.hatched,
         municipality.fac,
         unique.ID,
         Sampling_date.date, yday,
         zona.SIT.discrete.fac,
         zona.SIT.discrete.ord,
         X.num, Y.num) %>%
  na.omit() %>%
  droplevels() %>%
  nrow()
```

```
[1] 665
```

```
nrow(d.hatching)
```

```
[1] 665
```

```r
## No missing values
##
d.hatching.Ascona.25 <- d.hatching %>%
  filter(municipality.fac == "Ascona") %>%
  droplevels()
##
d.hatching.Losone.25 <- d.hatching %>%
  filter(municipality.fac == "Losone") %>%
  droplevels()
##
## check
unique(d.hatching.Ascona.25$municipality.fac)
```

```
[1] Ascona
Levels: Ascona
```

```r
unique(d.hatching.Losone.25$municipality.fac)
```

```
[1] Losone
Levels: Losone
```

We also create two data sets where we zoom in the treated zone with its neighbour area, for both municipalities.

```r
d.hatching.Ascona.25.zoom <- d.hatching.Ascona.25 %>%
  filter(## ovitraps with letters after the number are not part of the SIT
    ## design, thus we drop them.
    ## (see "istruzioni tabella ASC-LOS.docx")
    str_detect(unique.ID, pattern = "[a-zA-Z]\\.\\d+[a-z]", negate = TRUE)) %>%
  droplevels()
## check
unique(d.hatching.Ascona.25.zoom$unique.ID)
```

```
 [1] Ascona.1  Ascona.2  Ascona.4  Ascona.5  Ascona.6  Ascona.7  Ascona.9
 [8] Ascona.10 Ascona.11 Ascona.12 Ascona.14 Ascona.15 Ascona.16 Ascona.18
[15] Ascona.19 Ascona.23 Ascona.25 Ascona.27 Ascona.28 Ascona.13 Ascona.20
[22] Ascona.21 Ascona.24 Ascona.17 Ascona.26 Ascona.3  Ascona.8
27 Levels: Ascona.1 Ascona.10 Ascona.11 Ascona.12 Ascona.13 ... Ascona.9
```

```r
## makes sense
##
d.hatching.Losone.25.zoom <- d.hatching.Losone.25 %>%
  filter(## ovitraps with letters after the number are not part of the SIT
    ## design, thus we drop them.
    ## (see "istruzioni tabella ASC-LOS.docx")
    str_detect(unique.ID, pattern = "[a-zA-Z]\\.\\d+[a-z]", negate = TRUE)) %>%
  droplevels()
## check
unique(d.hatching.Losone.25.zoom$unique.ID)
```

```
 [1] Losone.1  Losone.3  Losone.4  Losone.10 Losone.11 Losone.12 Losone.13
 [8] Losone.14 Losone.15 Losone.16 Losone.18 Losone.19 Losone.21 Losone.22
[15] Losone.24 Losone.26 Losone.5  Losone.6  Losone.7  Losone.8  Losone.9
[22] Losone.20 Losone.23 Losone.27 Losone.28 Losone.17 Losone.25 Losone.2
28 Levels: Losone.1 Losone.10 Losone.11 Losone.12 Losone.13 ... Losone.9
```

```r
## makes sense
```

We fit two separate models, one for Losone and one for Ascona.

We aim to model the hatching rates of *Aedes albopictus* eggs (*Perc.egg.hatch.per.trap*) over the season (*yday*) and territory (*X.num*, *Y.num*).

yday will be included as numeric variable; the same holds true for *X.num* and *Y.num*, which represent the swiss coordinates.

The response variable is binomial, representing the counts of hatched versus non-hatched eggs (quantified by the variables *hatched* and *non.hatched*). To account for overdispersion, the quasi-binomial family is used for formal modelling.

We don't want to assume any specific seasonal pattern, therefore we will fit a Generalised Additive Model (GAM), which provides flexibility in determining the best shape for the seasonal trend in the given municipality. However, this flexibility comes at the cost of interpretability.

Let's fit the model for Losone.

Note that we use the element `s(X.num, Y.num)` to plot spatial data. We can use this isotropic smoothing because both predictors are spatial coordinates and we assume the spatial effect is isotropic (i.e., the same in all directions) and the two variables are on the same scale.

```
gam.hatched_space.Losone.25 <- gamV(cbind(hatched, non.hatched) ~
                                      s(yday) +

                                      s(X.num, Y.num), ## new element!

                                    method = "GCV.Cp", ## as in gam
                                    family = "quasibinomial",
                                    data = d.hatching.Losone.25)
##
summary(gam.hatched_space.Losone.25)
```

```

Family: quasibinomial
Link function: logit

Formula:
cbind(hatched, non.hatched) ~ s(yday) + s(X.num, Y.num)

Parametric coefficients:
                  Estimate     Std. Error  t value               Pr(>|t|)
(Intercept) 1.969174026670 0.071716875321 27.45761 < 0.0000000000000000222 ***
---
Signif. codes:  0 '***' 0.001 '**' 0.01 '*' 0.05 '.' 0.1 ' ' 1

Approximate significance of smooth terms:
                          edf         Ref.df       F                p-value
s(yday)         7.52111955201  8.39567598688 18.7517 < 0.0000000000000000222 ***
s(X.num,Y.num) 14.95776507877 19.32786113742  5.4252 < 0.0000000000000000222 ***
---
Signif. codes:  0 '***' 0.001 '**' 0.01 '*' 0.05 '.' 0.1 ' ' 1

R-sq.(adj) =  0.472   Deviance explained = 52.6%
GCV = 9.6545  Scale est. = 8.7999    n = 306
```

```
##
## We also fit a model on the treated zone and its neighbour area
```

```r
gam.hatched_space.Losone.25_zoom <- gamV(cbind(hatched, non.hatched) ~
                                         ## Need to specify k
                                         s(yday, k = 9) +
                                         ##
                                         ## Need to specify k
                                         s(X.num, Y.num, k = 25), ## new element!

                                       method = "GCV.Cp", ## as in gam
                                       family = "quasibinomial",
                                       data = d.hatching.Losone.25.zoom)
##
summary(gam.hatched_space.Losone.25_zoom)
```

```
Family: quasibinomial
Link function: logit

Formula:
cbind(hatched, non.hatched) ~ s(yday, k = 9) + s(X.num, Y.num,
    k = 25)

Parametric coefficients:
                   Estimate      Std. Error  t value               Pr(>|t|)
(Intercept) 1.5556476005956 0.0776163813712 20.04277 < 0.0000000000000000222 ***
---
Signif. codes:  0 '***' 0.001 '**' 0.01 '*' 0.05 '.' 0.1 ' ' 1

Approximate significance of smooth terms:
                         edf        Ref.df        F                 p-value
s(yday)        7.18558842365 7.80699694962 18.63794 < 0.0000000000000000222 ***
s(X.num,Y.num) 5.02103277814 6.94560890279  7.25232          0.00000056189 ***
---
Signif. codes:  0 '***' 0.001 '**' 0.01 '*' 0.05 '.' 0.1 ' ' 1

R-sq.(adj) =  0.491   Deviance explained = 54.5%
GCV =  10.81  Scale est. = 9.8952    n = 184
```

Let's visualise the results.

```r
plot.gam(gam.hatched_space.Losone.25,
         ## Adding the shift would not give the expected result (i.e., direct
         ## interpretation). In fact, the intercept also contains the
         ## estimate for the coordinates, thus adding the shift would
         ## change the interpretation, providing an estimated shift when
         ## s(X,Y) = 0. In any case, we are interested
         ## in the spatial smooth. We include the seasonal pattern
         ## to control for it when estimating the spatial pattern.
         # shift = coef(gam.hatched_space.Losone.25)["(Intercept)"],
         select = 1,
         trans = plogis)
```

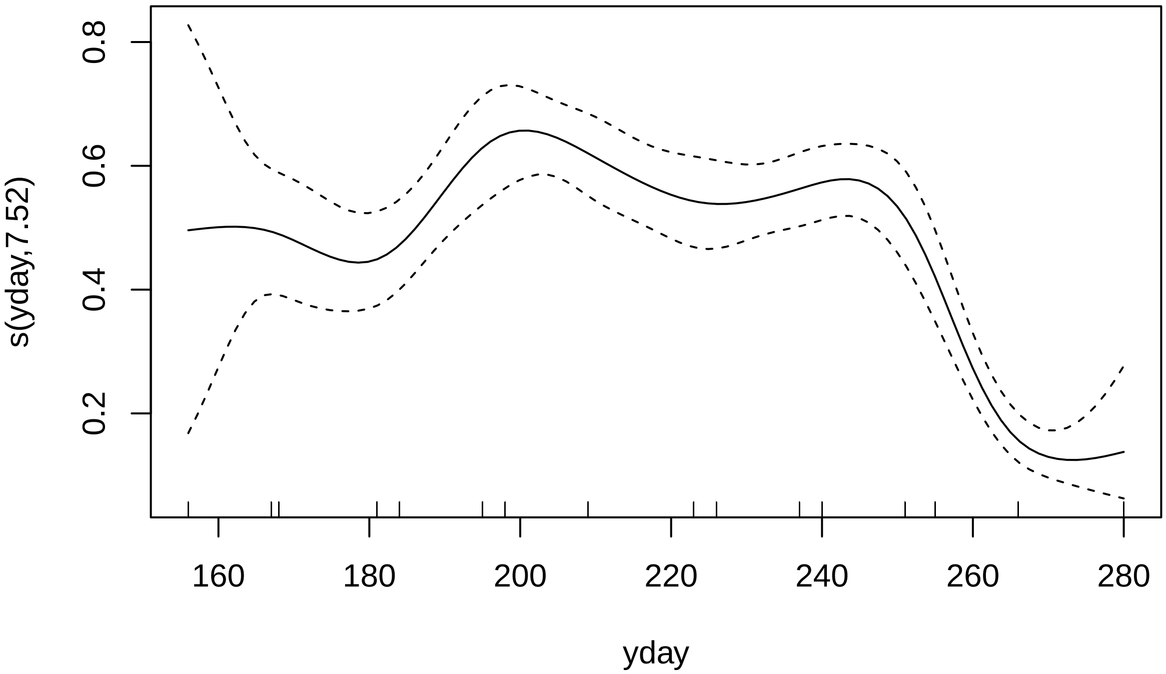


```
##
gg.gamViz.L.25 <- plot.gamViz(gam.hatched_space.Losone.25,
                              select = 2,
                              ## Adding the shift doesn't change anything
                              ## for the spatial, because the legend is
                              ## relative to the mean value and it is
                              ## multiplicative. Thus, even if we shift everything
                              ## by the intercept and then back-transform,
                              ## this relationship doesn't change.
                              # shift = coef(gam.hatched_space.Losone.25)["(Intercept)"],
                              trans = plogis) +
  l_fitRaster() + l_fitContour() +
  l_points(shape = 16, col = "black", size = 2) +
  l_points(x = d.coord.center.losone$X, y = d.coord.center.losone$Y,
           colour = "red", size = 3, shape = 4) +
  coord_fixed() +
  labs(title = "Losone")
gg.gamViz.L.25
```

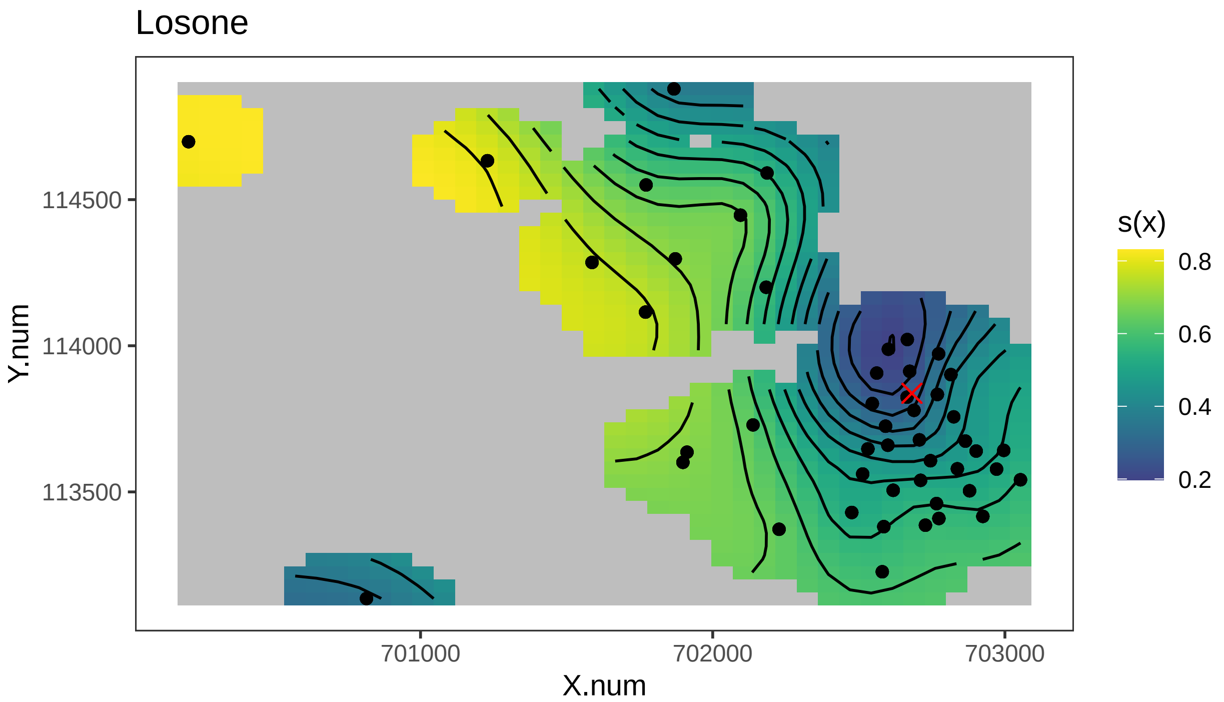

We can interpret the spatial plot as follows: the map represents the relative spatial contribution to hatching probability, derived from the spatial smooth of the GAM and displayed on the probability scale. Values around 0.5 correspond to locations with an average spatial effect, whereas lower or higher values indicate areas that are relatively less or more favourable for egg hatching.

These values reflect spatial deviations around the model's mean effect and should not be interpreted as absolute hatching probabilities.

The spatial graph shows a clear treatment effect. In fact, we observe that as we move away from the center of the treated zone (marked with a red cross), relative hatching rates increase.

Let's visualise the results of the second model, with data only on the treated and neighbour zones.

```
plot.gam(gam.hatched_space.Losone.25_zoom,
         ## Here we don't add the shift because it would be wrong.
         ## In fact the intercept also contains the coordinates.
         select = 1,
         trans = plogis)
```

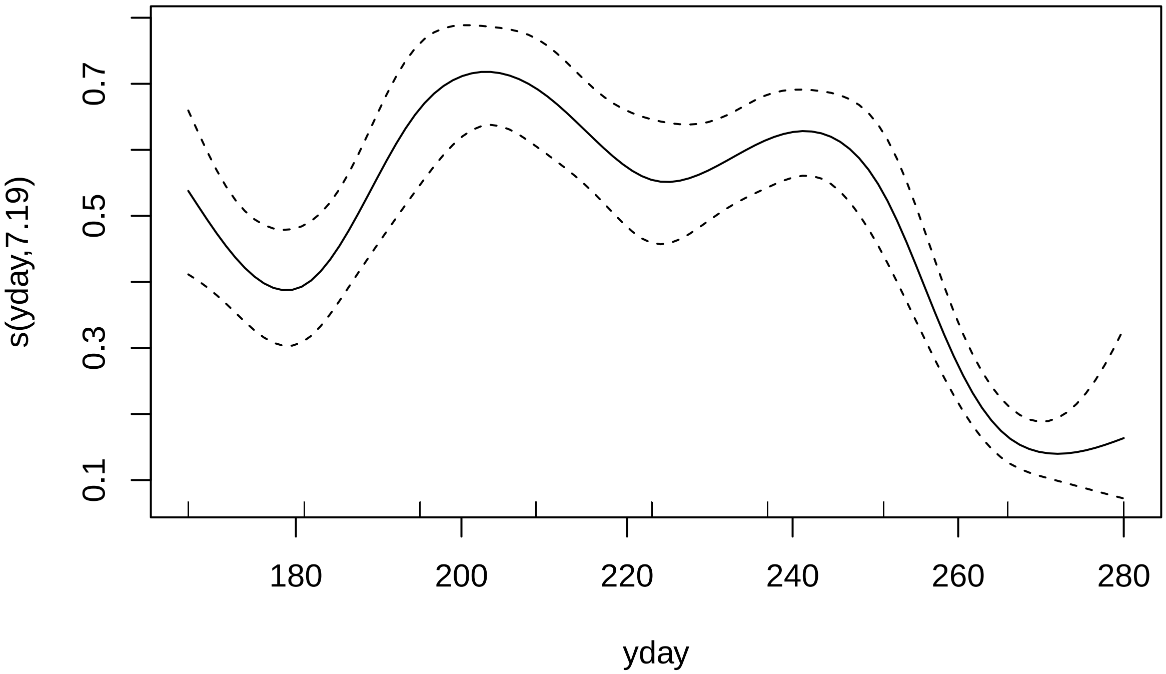


```
##
gg.gamViz.L.25_zoom <- plot.gamViz(gam.hatched_space.Losone.25_zoom,
                                   select = 2,
                                   trans = plogis) +
  l_fitRaster() + l_fitContour() +
  l_points(shape = 16, col = "black", size = 2) +
  l_points(x = d.coord.center.losone$X, y = d.coord.center.losone$Y,
           colour = "red", size = 3, shape = 4) +
  coord_fixed() +
  labs(title = "Losone")
gg.gamViz.L.25_zoom
```

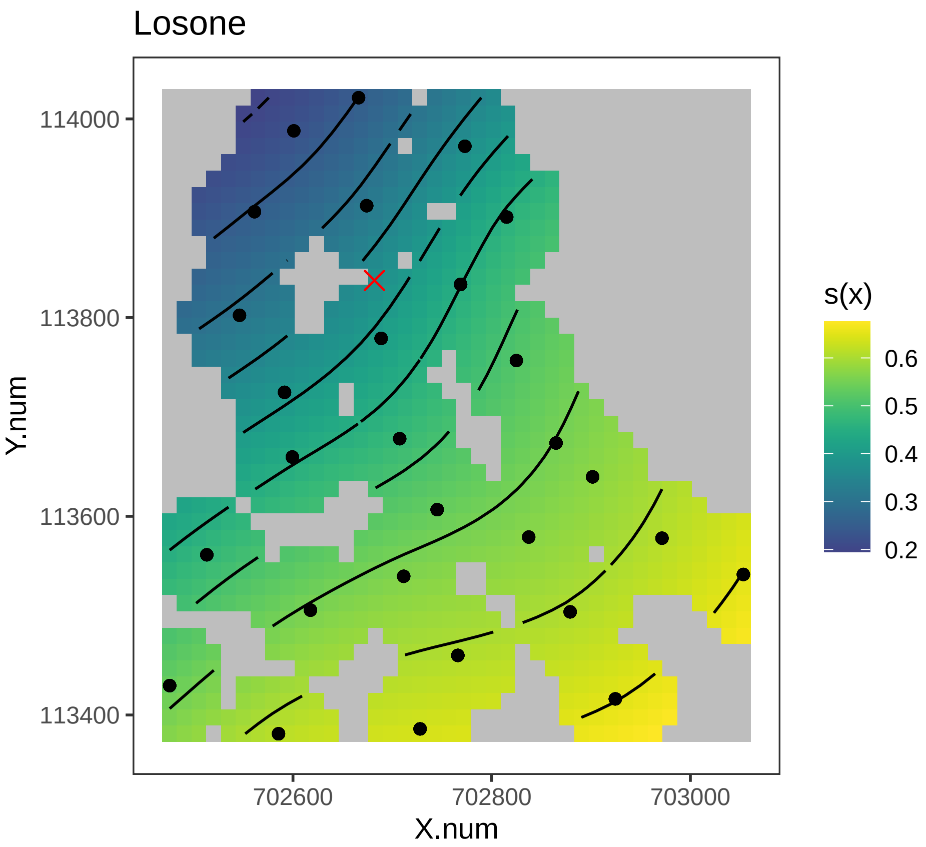

The spatial graph shows a clear pattern: as we move toward the lower right corner of the plot (i.e., away from the center of the treated zone marked with a red cross), hatching rates increase.

We refit the same model for Ascona.

```
gam.hatched_space.Ascona.25 <- gamV(cbind(hatched, non.hatched) ~
                                      s(yday) +

                                      s(X.num, Y.num),

                                    method = "GCV.Cp", ## as in gam
                                    family = "quasibinomial",
                                    data = d.hatching.Ascona.25) ## new element!
##
summary(gam.hatched_space.Ascona.25)
```

```
Family: quasibinomial
Link function: logit

Formula:
cbind(hatched, non.hatched) ~ s(yday) + s(X.num, Y.num)

Parametric coefficients:
                   Estimate      Std. Error  t value                 Pr(>|t|)
(Intercept) 2.3456121584567 0.0651520086999 36.00215 < 0.000000000000000222 ***
---
Signif. codes:  0 '***' 0.001 '**' 0.01 '*' 0.05 '.' 0.1 ' ' 1
```

```
Approximate significance of smooth terms:
                            edf          Ref.df        F                 p-value
s(yday)          7.32984260935  8.23648045001 44.44592 < 0.000000000000000222
s(X.num,Y.num) 15.98440064960 20.47258536938  5.86516 < 0.000000000000000222

s(yday)        ***
s(X.num,Y.num) ***
---
Signif. codes:  0 '***' 0.001 '**' 0.01 '*' 0.05 '.' 0.1 ' ' 1

R-sq.(adj) =  0.669   Deviance explained = 66.9%
GCV = 8.5382  Scale est. = 8.4075    n = 359
```

```
##
## We also fit a model on the treated zone and its neighbour area
gam.hatched_space.Ascona.25_zoom <- gamV(cbind(hatched, non.hatched) ~
                                           ## Need to specify k
                                           s(yday, k = 9) +
                                           ## Need to specify k
                                           s(X.num, Y.num, k = 25),

                                         method = "GCV.Cp", ## as in gam
                                         family = "quasibinomial",
                                         data = d.hatching.Ascona.25.zoom) ## new element!
##
summary(gam.hatched_space.Ascona.25_zoom)
```

```
Family: quasibinomial
Link function: logit

Formula:
cbind(hatched, non.hatched) ~ s(yday, k = 9) + s(X.num, Y.num,
    k = 25)

Parametric coefficients:
                   Estimate      Std. Error  t value               Pr(>|t|)
(Intercept) 1.7003442742026 0.0724054452499 23.48365 < 0.000000000000000222 ***
---
Signif. codes:  0 '***' 0.001 '**' 0.01 '*' 0.05 '.' 0.1 ' ' 1

Approximate significance of smooth terms:
                          edf         Ref.df        F               p-value
s(yday)        4.99002025780 6.05816879619 48.61010 < 0.0000000000000002 ***
s(X.num,Y.num) 5.83278051852 8.05773171567  2.48213             0.014374 *
---
Signif. codes:  0 '***' 0.001 '**' 0.01 '*' 0.05 '.' 0.1 ' ' 1

R-sq.(adj) =  0.675   Deviance explained = 67.6%
GCV =  9.179  Scale est. = 8.335     n = 183
```

Let's visualise the results.

```
plot.gam(gam.hatched_space.Ascona.25,
         ## Here we don't add the shift because it would be wrong.
```

```
## In fact the intercept also contains the coordinates.
select = 1,
trans = plogis)
```

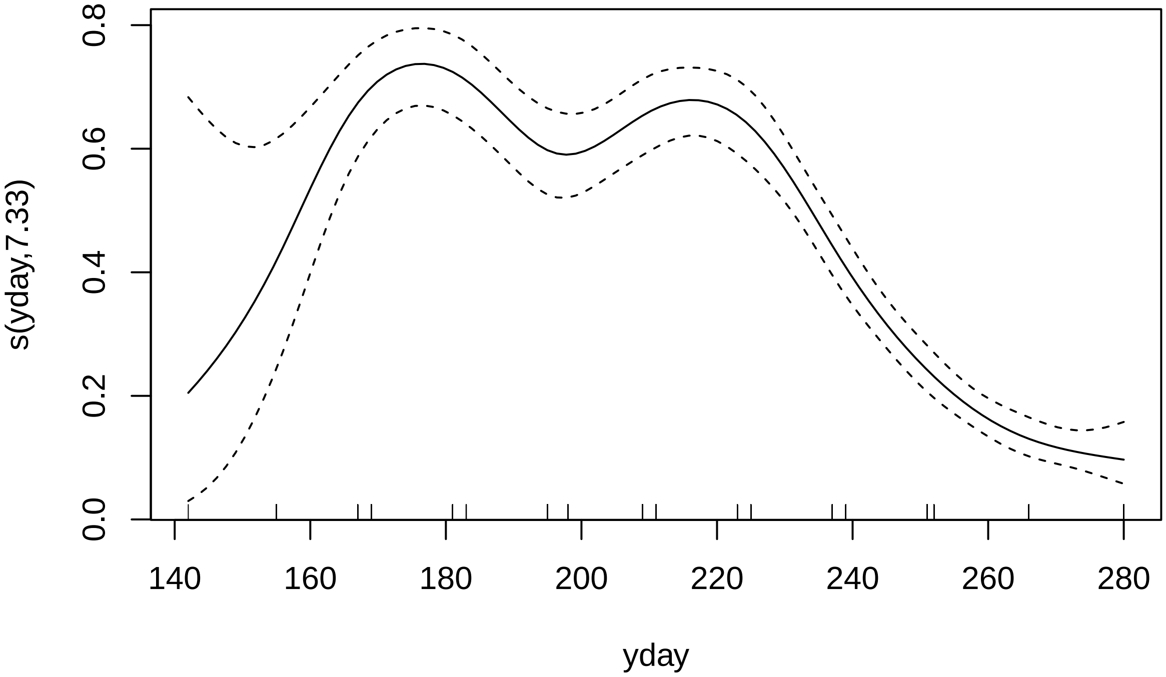


```
##
gg.gamViz.A.25 <- plot.gamViz(gam.hatched_space.Ascona.25,
                              select = 2,
                              trans = plogis) +
  l_fitRaster() + l_fitContour() +
  l_points(shape = 16, col = "black", size = 2) +
  l_points(x = d.coord.center.ascona$X, y = d.coord.center.ascona$Y,
           colour = "red", size = 3, shape = 4) +
  coord_fixed() +
  labs(title = "Ascona")
gg.gamViz.A.25
```

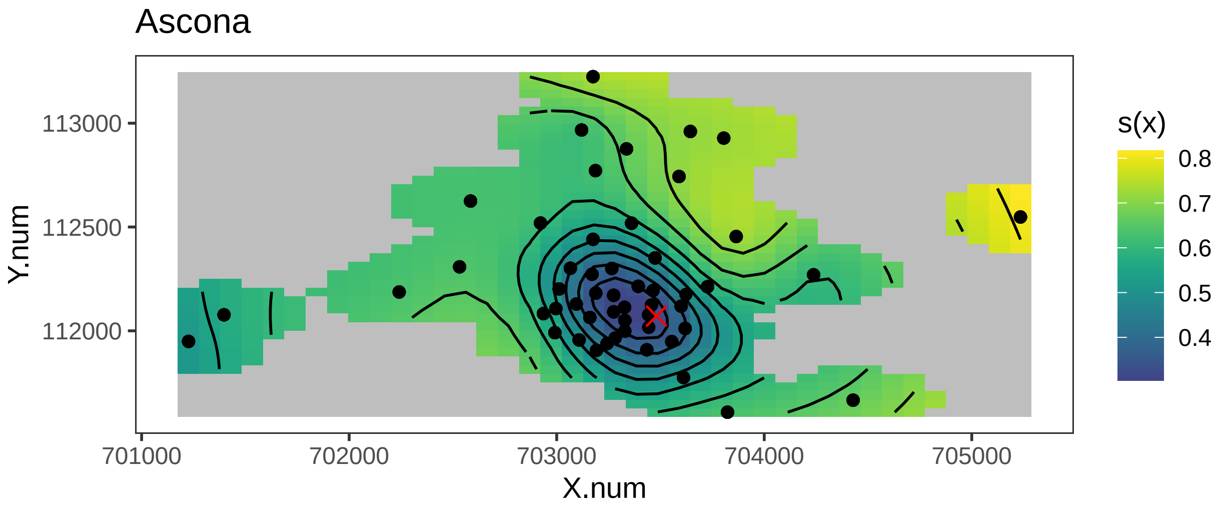

The spatial graph shows a clear treatment effect. In fact, we observe that as we move away from the center of the treated zone (marked with a red cross), hatching rates increase.

Let's visualise the results of the second model, with data only on the treated and neighbour zones.

```
plot.gam(gam.hatched_space.Ascona.25_zoom,
         ## Here we don't add the shift because it would be wrong.
         ## In fact the intercept also contains the coordinates.
         select = 1,
         trans = plogis)
```

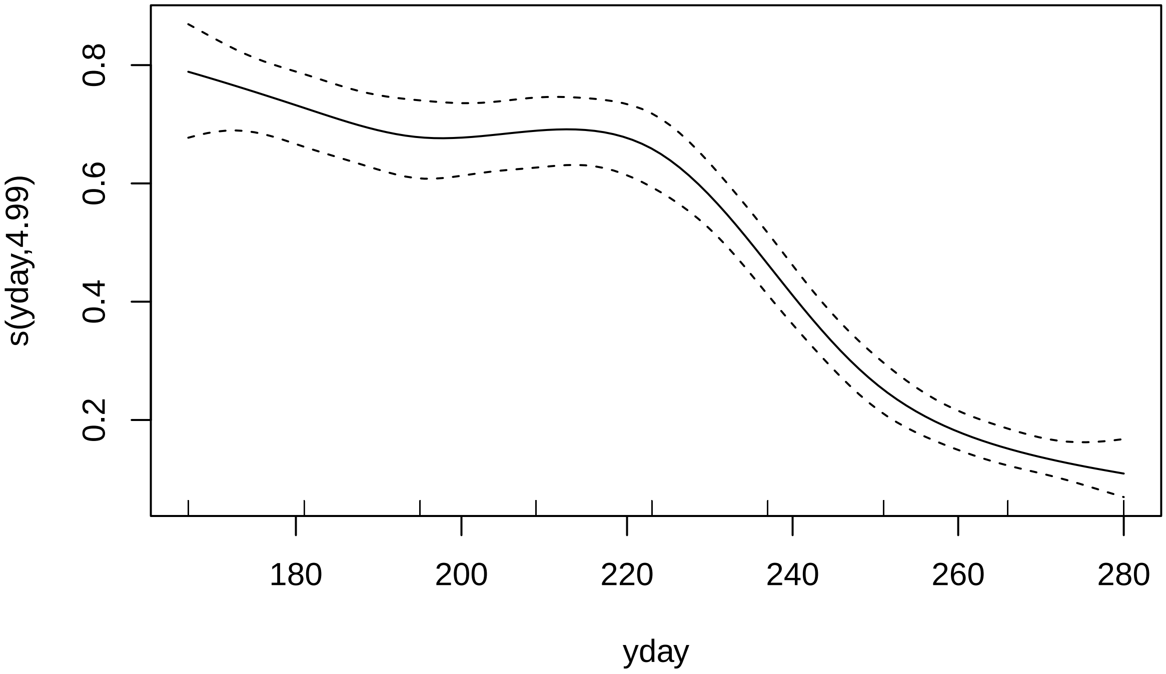


```
##
gg.gamViz.A.25_zoom <- plot.gamViz(gam.hatched_space.Ascona.25_zoom,
                                   select = 2,
                                   trans = plogis) +
  l_fitRaster() + l_fitContour() +
  l_points(shape = 16, col = "black", size = 2) +
  l_points(x = d.coord.center.ascona$X, y = d.coord.center.ascona$Y,
           colour = "red", size = 3, shape = 4) +
  coord_fixed() +
  labs(title = "Ascona")
gg.gamViz.A.25_zoom
```

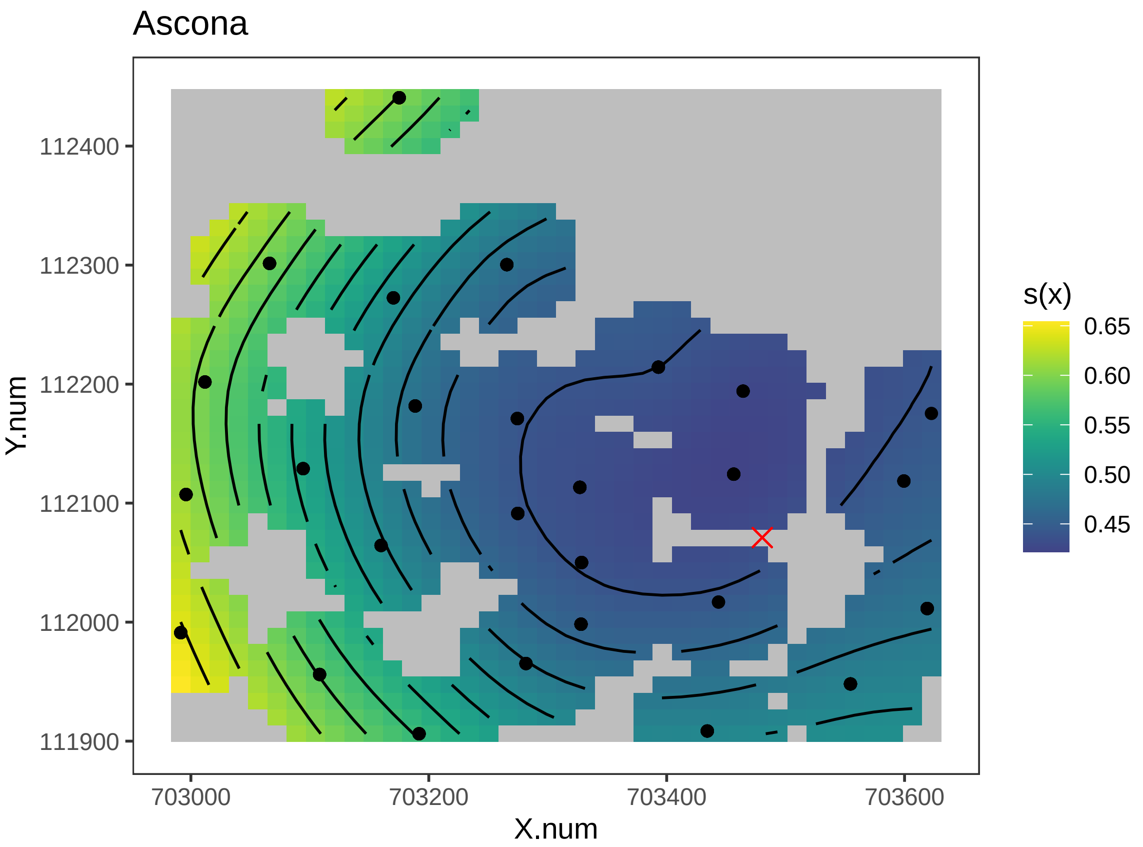

Again, the spatial graph shows a clear treatment effect. In fact, we observe that as we move away from the center of the treated zone (marked with a red cross), hatching rates increase.

6.3 Residual analysis

The residual analysis is performed on the models including all ovitraps in each municipality.

First of all, we apply the `gam.check()` function to the models, which produces some diagnostic information.

```
## Losone
par(mfrow = c(2, 2))
gam.check(gam.hatched_space.Losone.25)
```

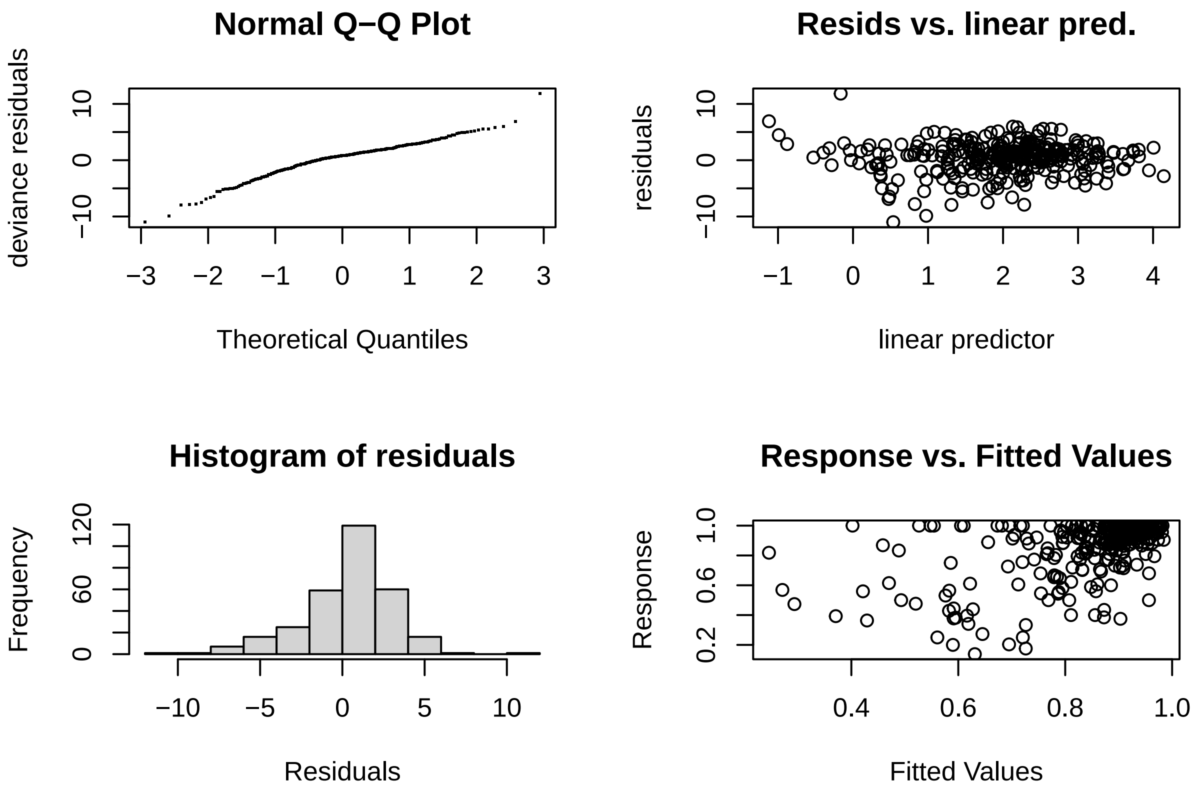


```
Method: GCV   Optimizer: outer newton
full convergence after 6 iterations.
Gradient range [-0.0000000001430718876704870,0.0000000190669725771975]
(score 9.6544519277629 & scale 8.79993035398424).
Hessian positive definite, eigenvalue range [0.0507926439004419,0.152790607333398].
Model rank =  39 / 39

Basis dimension (k) checking results. Low p-value (k-index<1) may
indicate that k is too low, especially if edf is close to k'.

                  k'   edf k-index p-value
s(yday)         9.00  7.52    0.87    0.02 *
s(X.num,Y.num) 29.00 14.96    1.12    1.00
---
Signif. codes:  0 '***' 0.001 '**' 0.01 '*' 0.05 '.' 0.1 ' ' 1
```

```r
par(mfrow = c(1, 1))
##
## Ascona
par(mfrow = c(2, 2))
gam.check(gam.hatched_space.Ascona.25)
```

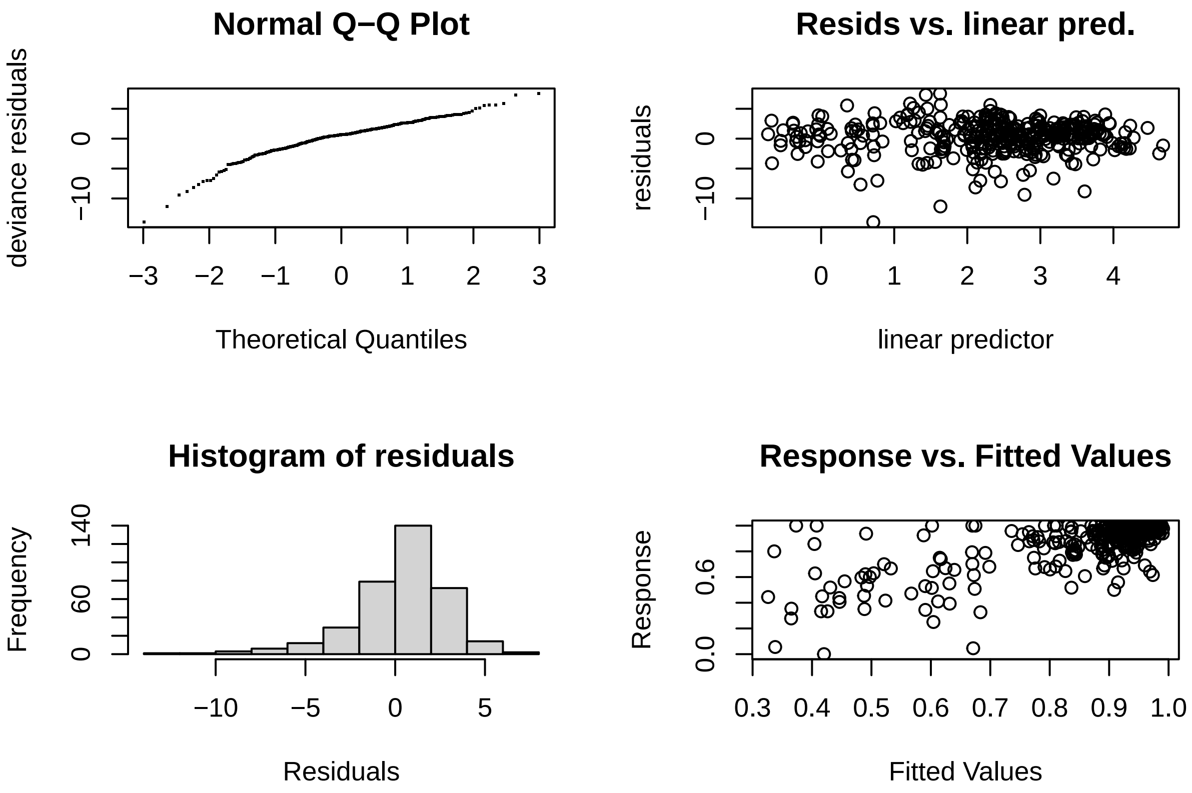


```
Method: GCV   Optimizer: outer newton
full convergence after 4 iterations.
Gradient range [-0.000000020693553121065,0.0000025990208410015]
(score 8.53822491748922 & scale 8.40748014963331).
Hessian positive definite, eigenvalue range [0.0272863755145113,0.119062213789452].
Model rank =  39 / 39

Basis dimension (k) checking results. Low p-value (k-index<1) may
indicate that k is too low, especially if edf is close to k'.

                   k'   edf k-index p-value
s(yday)          9.00  7.33    0.96    0.23
s(X.num,Y.num) 29.00 15.98    1.13    1.00
```

```
par(mfrow = c(1, 1))
```

Then, we store the pearson residuals in the original data frame, and we plot the residuals against the fitted values to see whether there is still structure in the data.

```
## Losone
d.hatching.Losone.25$resid_gam.tot.eggs_space.Losone <-
  resid(gam.hatched_space.Losone.25, type = "pearson")
##
d.hatching.Losone.25$fitted_gam.tot.eggs_space.Losone <-
  fitted(gam.hatched_space.Losone.25)
##
ggplot(data = d.hatching.Losone.25,
       mapping = aes(y = resid_gam.tot.eggs_space.Losone,
                     x = fitted_gam.tot.eggs_space.Losone)) +
```

```
  geom_hline(yintercept = 0) +
  geom_point(alpha = 0.2) +
  geom_smooth()
```

```
`geom_smooth()` using method = 'loess' and formula = 'y ~ x'
```

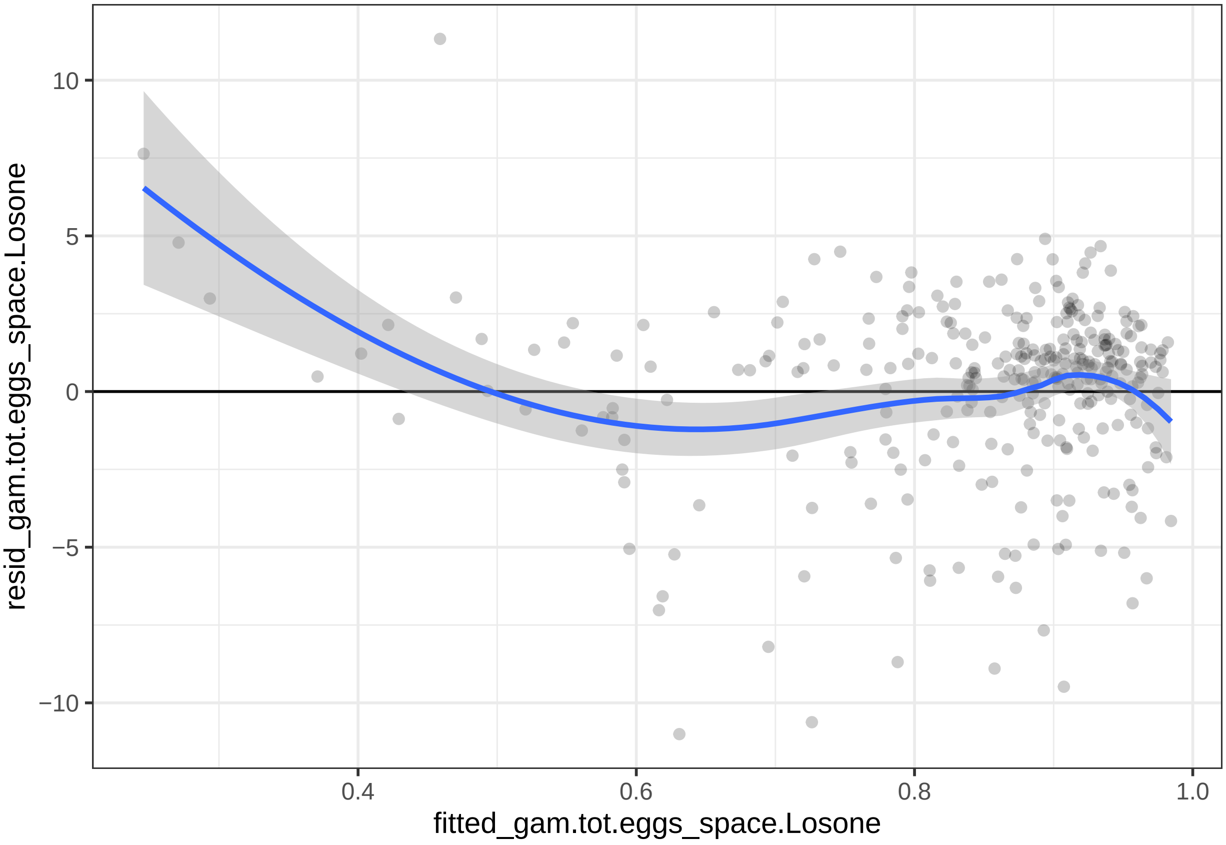


```
##
## Ascona
d.hatching.Ascona.25$resid_gam.tot.eggs_space.Ascona <-
  resid(gam.hatched_space.Ascona.25, type = "pearson")
##
##
d.hatching.Ascona.25$fitted_gam.tot.eggs_space.Ascona <-
  fitted(gam.hatched_space.Ascona.25)
##
ggplot(data = d.hatching.Ascona.25,
       mapping = aes(y = resid_gam.tot.eggs_space.Ascona,
                     x = fitted_gam.tot.eggs_space.Ascona)) +
  geom_hline(yintercept = 0) +
  geom_point(alpha = 0.2) +
  geom_smooth()
```

```
`geom_smooth()` using method = 'loess' and formula = 'y ~ x'
```

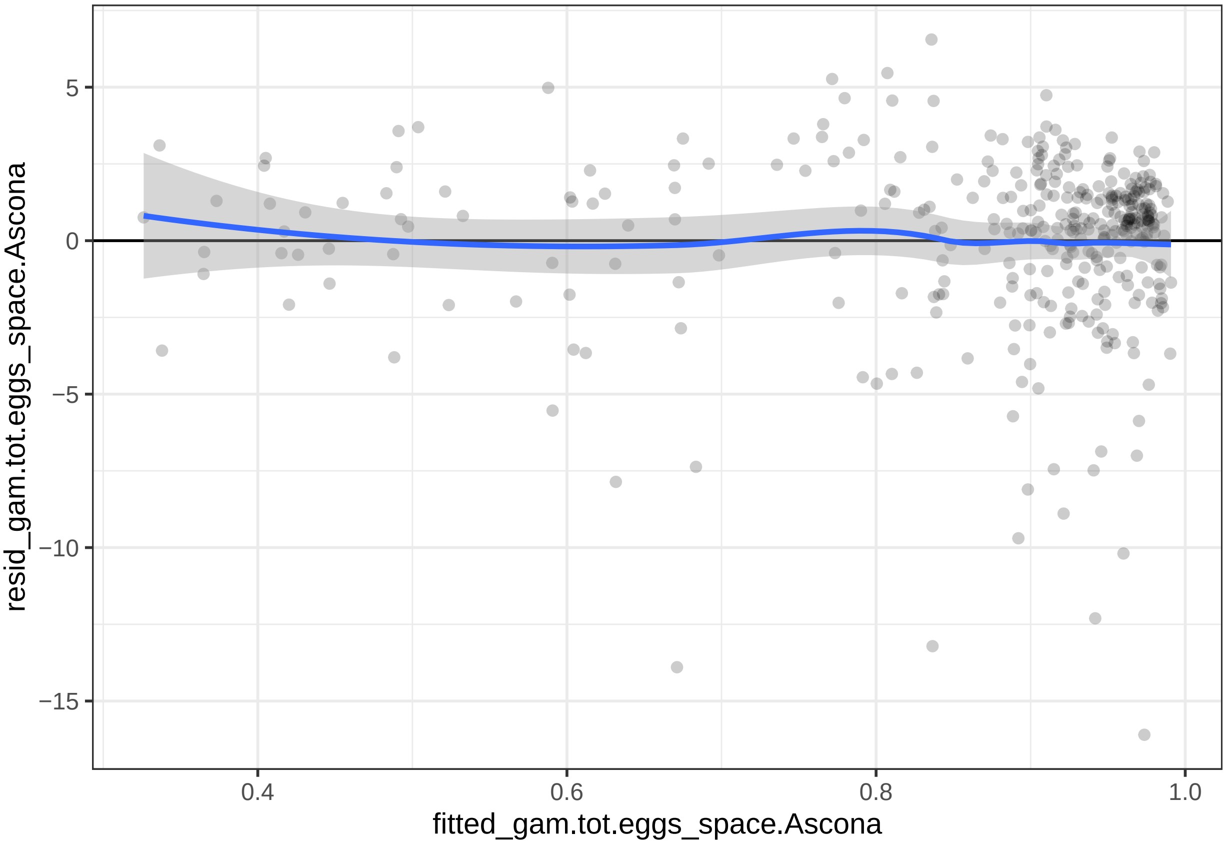

The blue lines are located on the x-axis (or the confidence band contains it), indicating that there doesn't seem to be structure left in the residuals.

7 Methods

7.1 Generalised Additive Mixed-Effects Model (GAMM)

We modelled the percentage of hatched Ae. albopictus eggs (combining the variables *hatched.albo.eggs.after.proc* and *non.hatched.albo.eggs.after.proc* to a binomial response variable) using a Generalised Additive Mixed Model (GAMM) with a quasi binomial distribution to account for overdispersion. The model included a smooth term for sampling date, expressed as day of the year (*yday*), and we allowed this smooth to vary by treatment zone (*zona.SIT.discrete.fac*), resulting in zone-specific smooth functions. Treatment zone, a categorical variable with three levels, was included as a fixed effect. To account for repeated measurements of the same trap, we included trap identity (*unique.ID*) as a random intercept.

This modelling procedure was applied separately for the municipalities of Ascona and Losone.

Model complexity was evaluated using Likelihood-Ratio (Chi-square) tests. All analyses were performed in `R` using the packages `{mgcv}` and `{mgcViz}`, with statistical significance assessed at the 5% level. For additional details, see Section 5 of the Appendix.

@Diego: You can find the citations for the packages in the section "References"

7.2 Spatial Generalised Additive Model (spatial GAM)

We modelled the percentage of hatched Ae. albopictus eggs (combining the variables *hatched.albo.eggs.after.proc* and *non.hatched.albo.eggs.after.proc* to a binomial response variable) using a Generalised Additive Model (GAM) with a quasi binomial distribution to account for overdispersion. The model included a smooth term for sampling date, expressed as day of the year (*yday*), and a bivariate smooth for geographic coordinates to capture spatial variation in hatching rates.

This modelling procedure was applied separately for the municipalities of Ascona and Losone.

Model complexity was evaluated using Likelihood-Ratio (Chi-square) tests as well as AIC and BIC. All analyses were performed in `R` using the packages `{mgcv}` and `{mgcViz}`, with statistical significance assessed at the 5% level. For additional details, see Section 6 of the Appendix.

@Diego: You can find the citations for the packages in the section "References"

8 Results

8.1 Generalised Additive Mixed-Effects Model (GAMM)

All models converge without numerical issues. The proportion of deviance explained is 64.4 % for Losone and 72.3 % for Ascona, indicating a satisfactory fit for both municipalities.

Model comparison shows that the more complex models improve model performance. In particular, the models including the smooth–by–treatment interaction provide a significantly better fit than the ones without the interaction (Likelihood-Ratio Chi-square test, p= 0.0000000000549 =< 0.001 for Losone, p= 0.008160211411218 =< 0.001 for Ascona). This confirms that allowing for treatment-specific smooth functions capture additional structure in the data and contribute independently to explaining variability in hatching rates.

The modelling results show that hatching rates in the treated zone are consistently lower than in the untreated (outside) zone. The buffer zone exhibits intermediate values.

These results are best summarised in Figure XYZ, which illustrates the seasonal dynamics across the three zones.

Alternatively, the differences in hatching rates between the treated and untreated zones can be examined directly. This is shown in Figure WXZ, which clearly confirms that the odds of hatching in the treated area remain consistently lower than those in the untreated area.

The magnitude of the reduction is not constant but varies over time. The average reduction in hatching rates in Losone is predicted to be 22.3%, whereas in Ascona it is predicted to be 9.5%.

@Diego: Choose the notation the journal prefers for the p-values.

@Diego: The average percentage reductions are calculated in the document "3 tables SIT 2025 losone ascona.pdf"

@Diego: Please insert the correct number for the figures

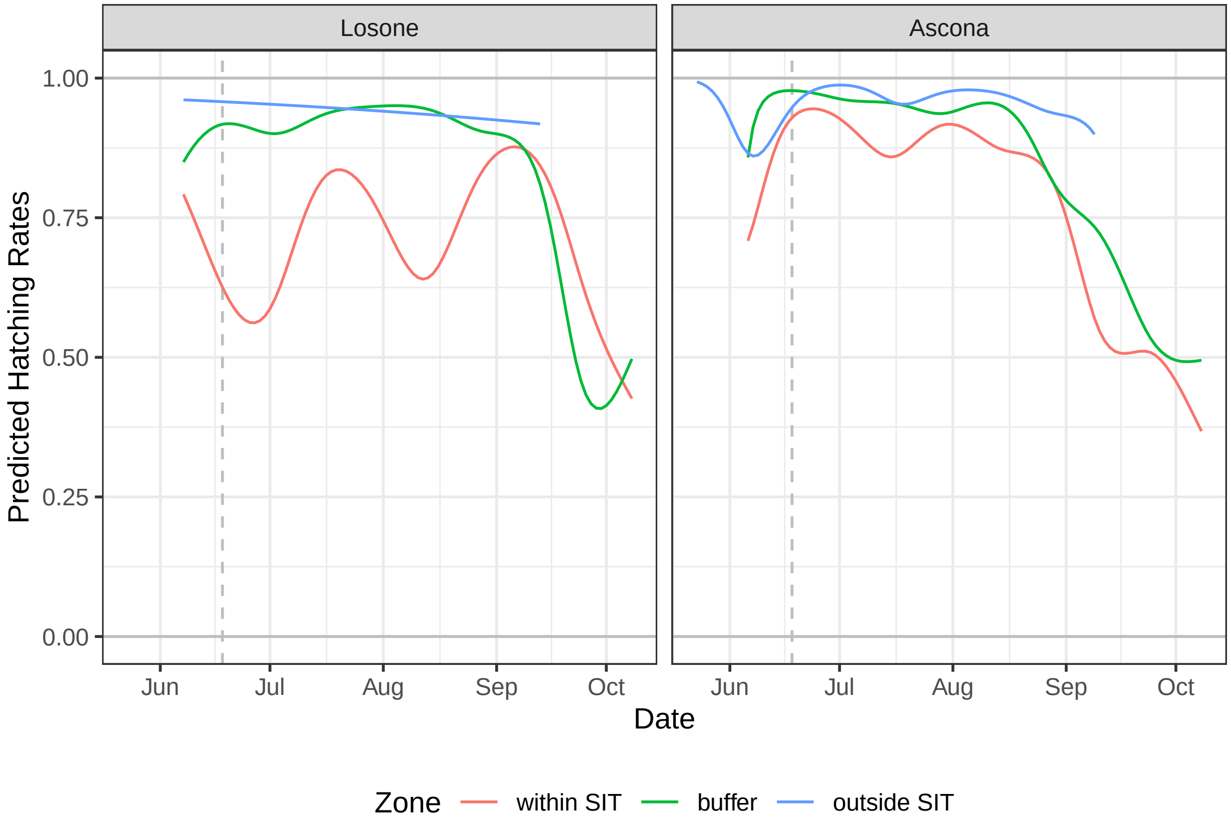

Figure 1: This figure shows the predicted hatching rates over time for Losone (left) and Ascona (right). Each plot displays three lines: blue for the untreated zone, red for the treated zone, and green for the buffer zone. The dashed vertical line at 17.06.2025 marks the start of the treatment. In both municipalities, hatching rates in the treated zone are lower relative to the untreated zone, while the buffer zone shows intermediate values.

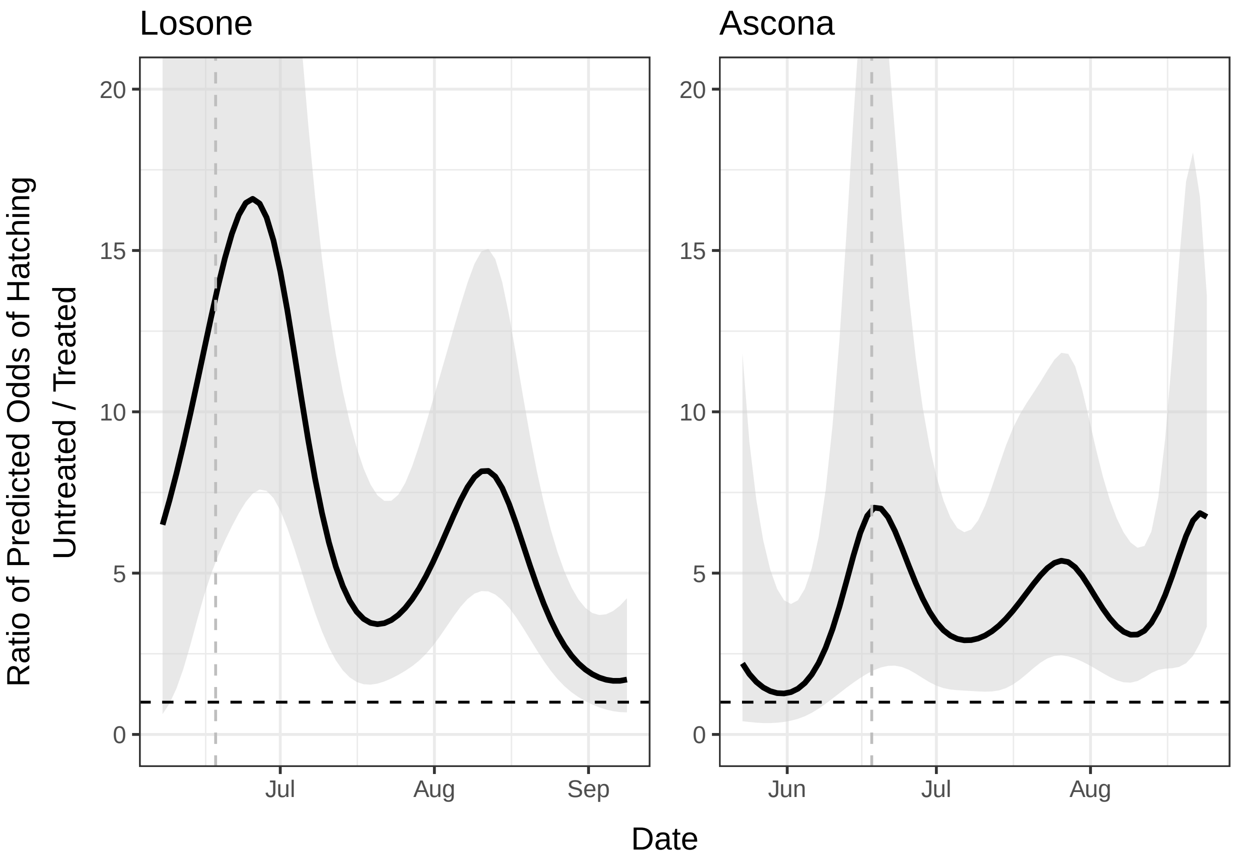

Figure 2: This figure shows the ratio of predicted odds of hatching between the untreated zone and the treated zone over time for Losone (left) and Ascona (right). The solid line represents the predicted ratio, while the shaded area indicates the 95% confidence interval. The dashed horizontal line at 1 indicates no difference between zones. Values above this line indicate higher odds of hatching in the untreated zone, while values below it indicate higher odds of hatching in the treated zone. The vertical dashed line marks the start of the treatment on 17.06.2025. The odds of hatching in the treated area remain consistently lower than those in the untreated area.

8.2 Spatial Generalised Additive Model (spatial GAM)

All models converge without numerical issues. The proportion of deviance explained is 52.6 % for Losone and 66.9 % for Ascona, indicating a satisfactory fit for both municipalities.

The modelling results reveal a clear gradient, with hatching rates increasing as the distance from the treated zone grows. These results are effectively illustrated in Figure ABC, which displays the spatial distribution of predicted hatching rates and highlights the smooth increase from the treated zone outward.

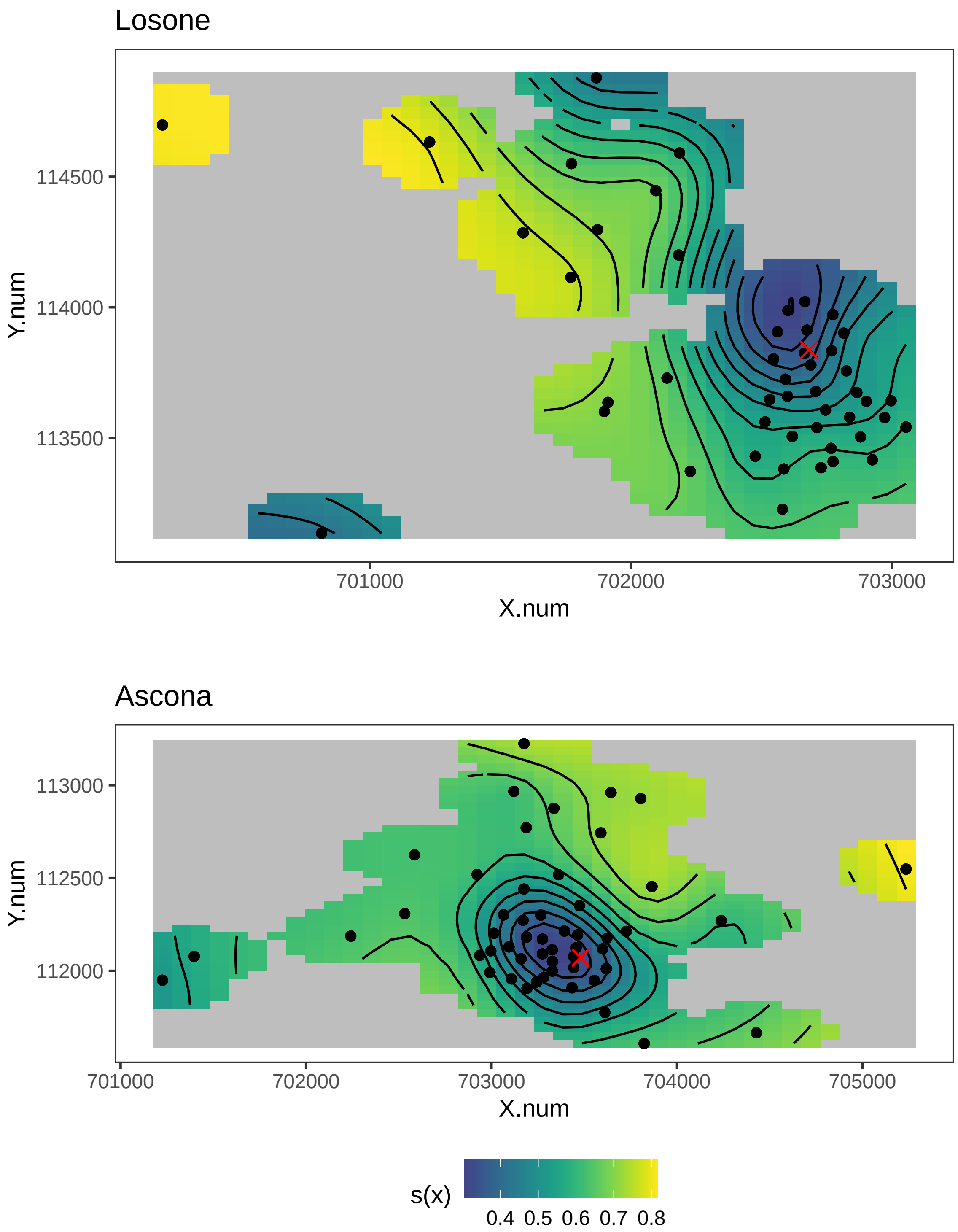

Figure 3: This figure shows the spatial distribution of predicted hatching rates for Losone (top) and Ascona (bottom) in 2025. Values around 0.5 correspond to locations with an average spatial effect, whereas lower or higher values indicate areas that are relatively less or more favourable for egg hatching. The red cross indicates the center of the treated zone. In both municipalities, hatching rates increase as the distance from the treated zone grows.

9 Conclusions

The GAMM results indicate a clear treatment effect in both Losone and Ascona. In fact, the estimated differences between untreated and treated zones is consistently positive, with several periods in which these differences are statistically significant. The difference between the buffer zone and the treated zone is also positive, though less pronounced than the difference with the untreated area.

From a spatial perspective, the evidence is also very strong. In particular, we observe that hatching rates increase as the distance from the treated zone grows.

These findings indicate that, following the treatment, hatching rates in the treated zones are estimated to be lower than in the other zones.

10 References

```r
## Packages
citation("mgcViz")
```

```
To cite the mgcViz package in publications use:

  Fasiolo, M., Nedellec, R., Goude, Y. and Wood, S.N., 2020. Scalable
  visualization methods for modern generalized additive models. Journal
  of computational and Graphical Statistics, 29(1), pp.78-86.

A BibTeX entry for LaTeX users is

  @Article{,
    title = {Scalable visualisation methods for modern Generalized Additive Models.},
    journal = {Journal of the Royal Statistical Society (B)},
    volume = {29},
    number = {1},
    pages = {78-86},
    year = {2020},
    author = {{Fasiolo} and {Matteo} and {Nedellec} and {Rapha{"e}l} and {Goude} and {Yannig} and {Wood
  }

As mgcViz is often updated, you may want to cite its version number.
Find it with 'help(package=mgcViz)'.
```

```r
citation("mgcv")[[1]] ## return several papers
```

```
Wood, S.N. (2011) Fast stable restricted maximum likelihood and
marginal likelihood estimation of semiparametric generalized linear
models. Journal of the Royal Statistical Society (B) 73(1):3-36

A BibTeX entry for LaTeX users is

  @Article{,
    title = {Fast stable restricted maximum likelihood and marginal
likelihood estimation of semiparametric generalized linear models},
    journal = {Journal of the Royal Statistical Society (B)},
    volume = {73},
    number = {1},
```

```
    pages = {3--36},
    year = {2011},
    author = {S. N. Wood},
    doi = {10.1111/j.1467-9868.2010.00749.x},
  }
```

```r
##
## R itself
citation()
```

```
To cite R in publications use:

  R Core Team (2025). _R: A Language and Environment for Statistical
  Computing_. R Foundation for Statistical Computing, Vienna, Austria.
  <https://www.R-project.org/>.

A BibTeX entry for LaTeX users is

  @Manual{,
    title = {R: A Language and Environment for Statistical Computing},
    author = {{R Core Team}},
    organization = {R Foundation for Statistical Computing},
    address = {Vienna, Austria},
    year = {2025},
    url = {https://www.R-project.org/},
  }

We have invested a lot of time and effort in creating R, please cite it
when using it for data analysis. See also 'citation("pkgname")' for
citing R packages.
```

11 Appendix

11.1 hatched eggs vs distance from the centre of the treatment

We are going to model the distance from the centre of the treatment as continuous variable. Note this model does not discretise the ovitraps into three groups (i.e., within SIT, buffer and outside SIT) as the models above, but it accounts for the treatment effect via the distance to the centre of the treated zone. So, in a sense, it better uses the spatial information.

On the other hand, this model assumes that the effect of seasonality is the same for all ovitraps regardless of in which zone they are located. We saw in the previous models that the seasonality shapes seem to differ.

Therefore, a-priori, it is hard to say whether this or the models above make better use of the information available.

11.1.1 Fitting the models

```r
## with distance from centre as continuous variable
##
gamm.hatched.Losone.continuous <- gamV(cbind(hatched, non.hatched) ~

                                          s(unique.ID, bs = "re") +
```

```r
                                          s(yday) +
                                          s(distance.from.center),

                                        family = "quasibinomial",
                                        method = "GCV.Cp", ## same as gam
                                        data = d.hatching.Losone)
summary(gamm.hatched.Losone.continuous)
```

```

Family: quasibinomial
Link function: logit

Formula:
cbind(hatched, non.hatched) ~ s(unique.ID, bs = "re") + s(yday) +
    s(distance.from.center)

Parametric coefficients:
                  Estimate      Std. Error  t value               Pr(>|t|)
(Intercept) 1.9598731680825 0.0768748177927 25.49435 < 0.000000000000000222 ***
---
Signif. codes:  0 '***' 0.001 '**' 0.01 '*' 0.05 '.' 0.1 ' ' 1

Approximate significance of smooth terms:
                                edf         Ref.df        F
s(unique.ID)            4.81486888984 46.00000000000  0.12445
s(yday)                 7.52782974903  8.40251964560 17.69702
s(distance.from.center) 7.41045819694  8.22582096648  9.32854
                                   p-value
s(unique.ID)                       0.20573
s(yday)                 < 0.0000000000000002 ***
s(distance.from.center) < 0.0000000000000002 ***
---
Signif. codes:  0 '***' 0.001 '**' 0.01 '*' 0.05 '.' 0.1 ' ' 1

R-sq.(adj) =  0.457   Deviance explained = 50.5%
GCV = 9.8766  Scale est. = 9.3681    n = 306
```

```r
##
##
gamm.hatched.Ascona.continuous <- gamV(cbind(hatched, non.hatched) ~

                                          s(unique.ID, bs = "re") +

                                          s(yday) +
                                          s(distance.from.center),

                                        family = "quasibinomial",
                                        method = "GCV.Cp", ## same as gam
                                        data = d.hatching.Ascona)
summary(gamm.hatched.Ascona.continuous)
```

```

Family: quasibinomial
Link function: logit
```

```
Formula:
cbind(hatched, non.hatched) ~ s(unique.ID, bs = "re") + s(yday) +
    s(distance.from.center)

Parametric coefficients:
                   Estimate      Std. Error  t value               Pr(>|t|)
(Intercept) 2.3404899740767 0.0825456326988 28.35389 < 0.0000000000000000222 ***
---
Signif. codes:  0 '***' 0.001 '**' 0.01 '*' 0.05 '.' 0.1 ' ' 1

Approximate significance of smooth terms:
                                   edf         Ref.df        F
s(unique.ID)            20.67553980522 50.00000000000  0.70026
s(yday)                  7.38565910323  8.27282620201 42.75836
s(distance.from.center)  2.95201415213  3.34832742662 15.24543
                                       p-value
s(unique.ID)                         0.0053004 **
s(yday)                 < 0.0000000000000000222 ***
s(distance.from.center) < 0.0000000000000000222 ***
---
Signif. codes:  0 '***' 0.001 '**' 0.01 '*' 0.05 '.' 0.1 ' ' 1

R-sq.(adj) =  0.678   Deviance explained = 68.5%
GCV = 8.5223  Scale est. = 8.3309    n = 359
```

Note that the models fitted here cannot be formally compared to the above ones via e.g., AIC or BIC (due to the fact that they are quasi-models). However, the deviance explained indicates that the models with a different seasonality smoother for each zone is superior.

Note, however, that these models very clearly show that the gradient of the ovitrap estimated hatching rates nicely correlates with distance. In other words, the 2 groups of models are complementary.

```r
plot.gam(gamm.hatched.Losone.continuous, pages = 1)
```

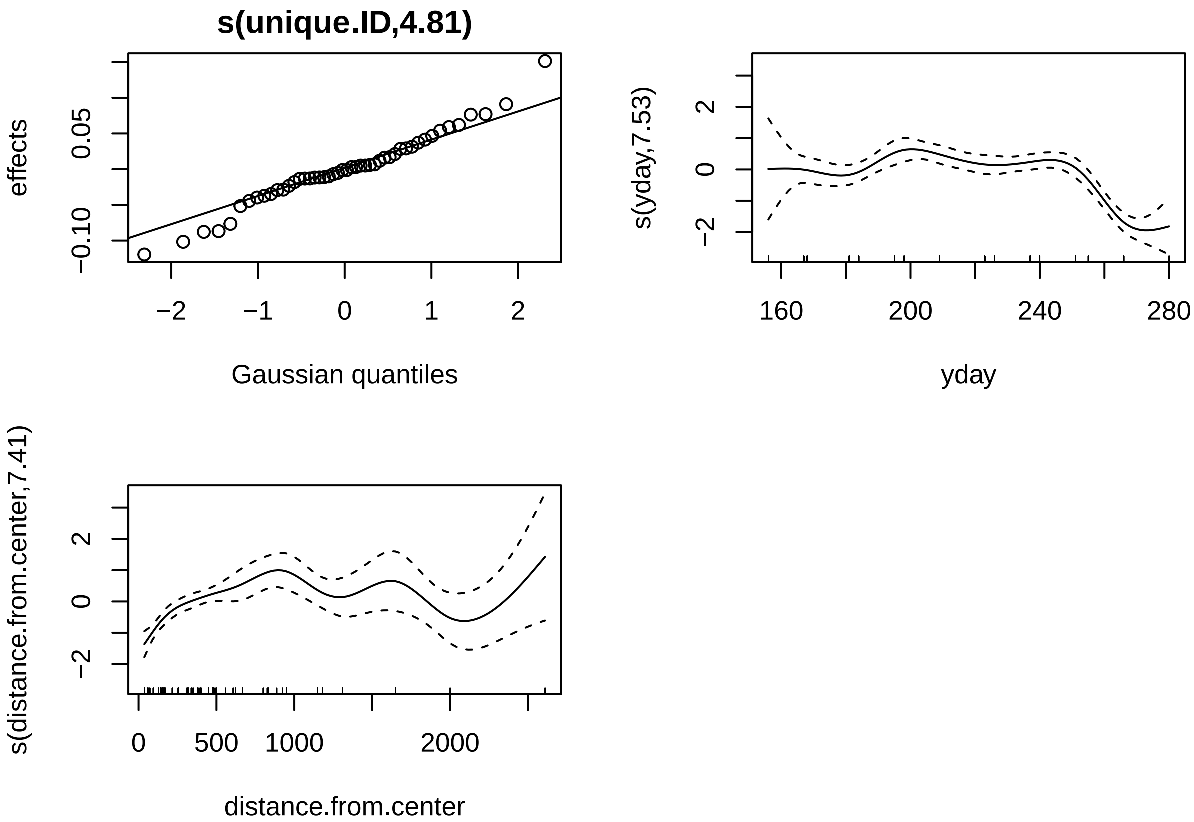


```r
plot.gam(gamm.hatched.Losone.continuous,
         select = 3,
         trans = exp,
         shift = gamm.hatched.Losone.continuous$coefficients["(Intercept)"],
         ylim = c(0, 30))
abline(h = 1, col = "grey")
```

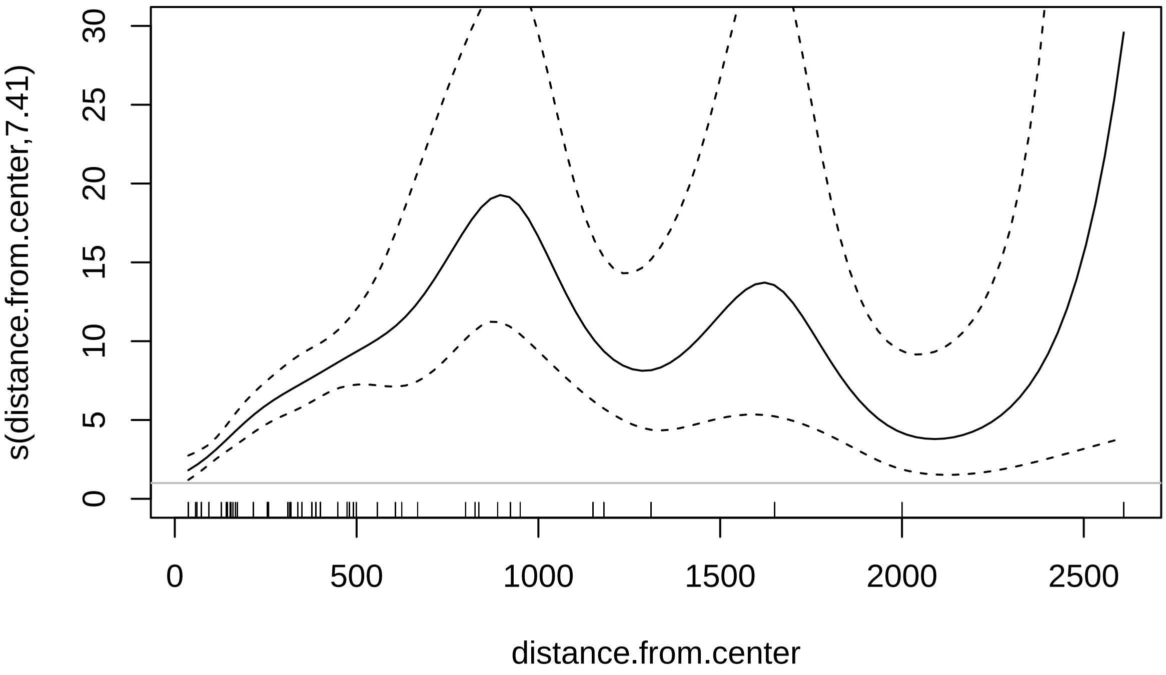

```
plot.gam(gamm.hatched.Ascona.continuous, pages = 1)
```

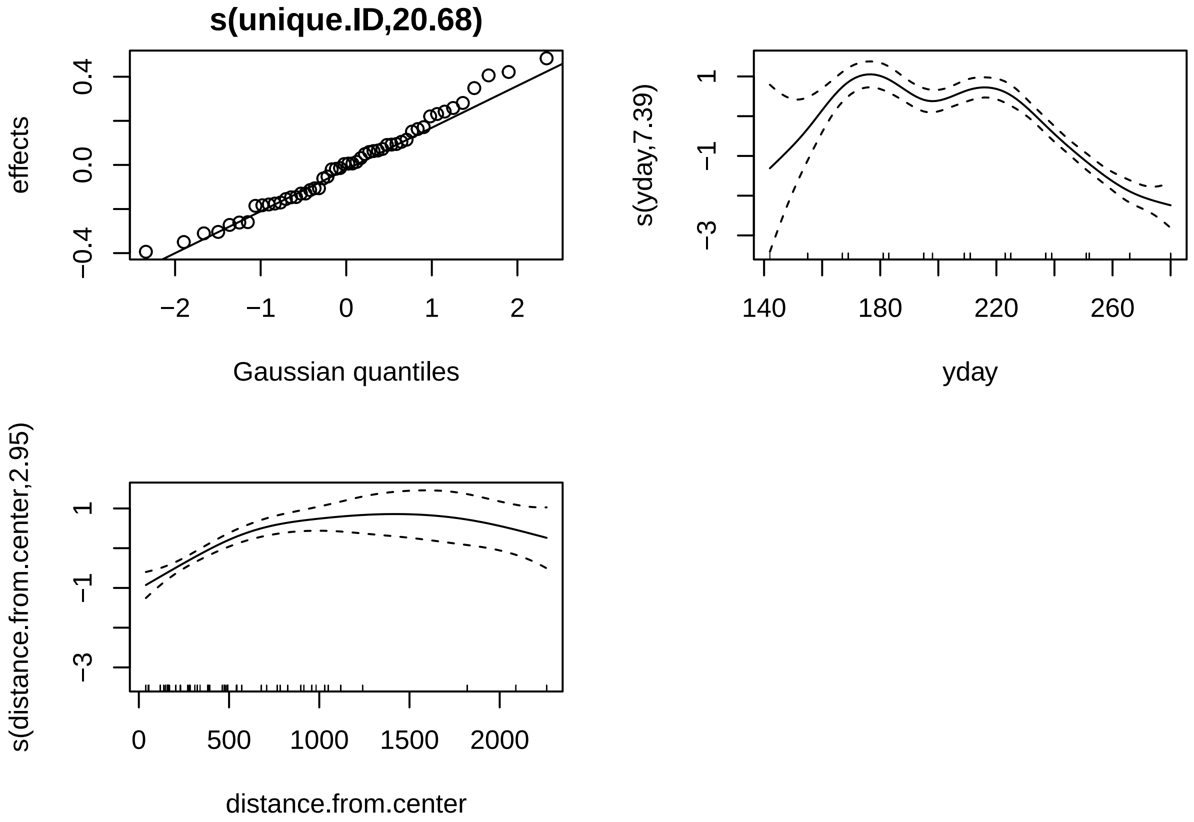


```
plot.gam(gamm.hatched.Ascona.continuous,
         select = 3,
         trans = exp,
         shift = gamm.hatched.Ascona.continuous$coefficients["(Intercept)"],
         ylim = c(0, 30))
abline(h = 1, col = "grey")
```

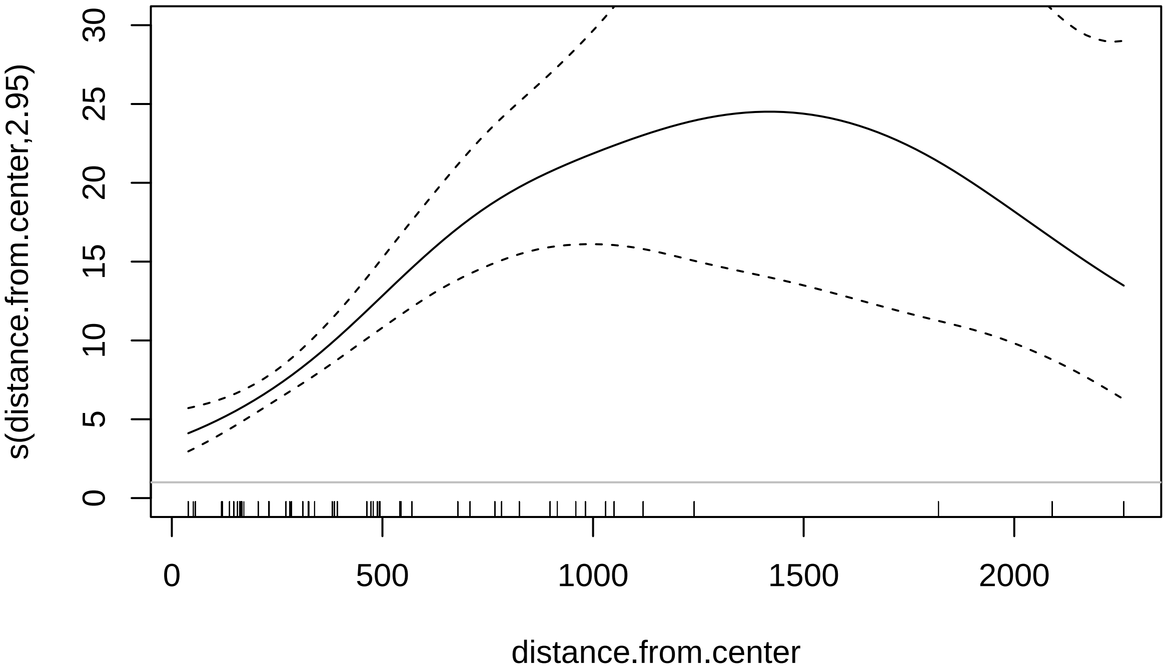

11.1.2 Fitted values

```
## Losone
d.hatching.Losone$fitted_gamm.hatching <-
  fitted(gamm.hatched.Losone.continuous)
## Ascona
d.hatching.Ascona$fitted_gamm.hatching <-
  fitted(gamm.hatched.Ascona.continuous)
##
d.ovitraps.fitted.new <- rbind(d.hatching.Losone,
                               d.hatching.Ascona)
##
## we include the distance as percentiles
d.ovitraps.fitted.new <- d.ovitraps.fitted.new %>%
  mutate(percentile.distance.from.center = percent_rank(distance.from.center))
ggplot(data = d.ovitraps.fitted.new,
       mapping = aes(y = fitted_gamm.hatching,
                     x = yday,
                     group = unique.ID,
                     colour = percentile.distance.from.center)) +
  geom_vline(xintercept = yday("2025-06-17"), linetype = "dashed", colour = "grey") +
  geom_hline(yintercept = c(0, 1), colour = "grey") +
  geom_point(alpha = 0.2) +
  geom_line(alpha = 0.2) +
  scale_y_sqrt() +
  facet_grid(~ municipality.fac) +
  theme(legend.position = "bottom")
```

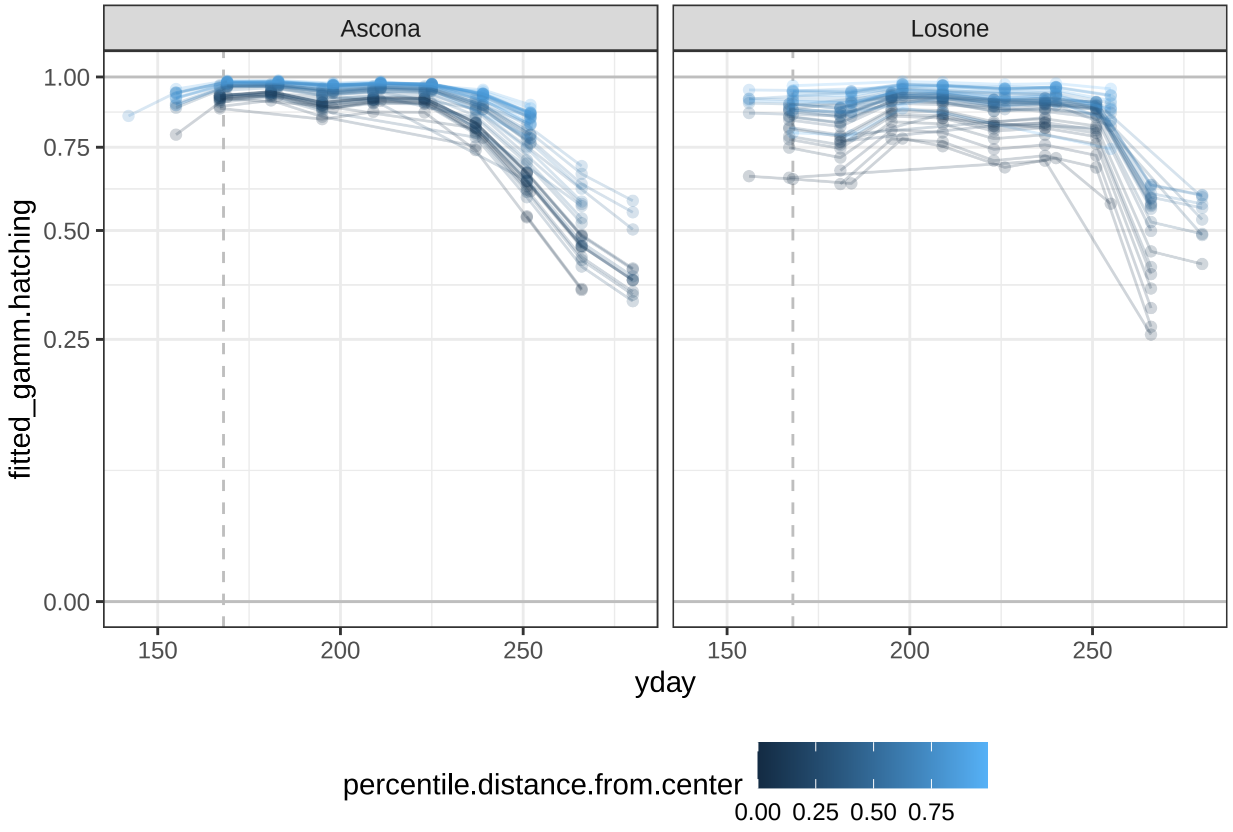

We can observe a clear pattern: the further away from the treatment centre, the higher the predicted hatching rates.

11.2 hatched eggs vs distance from centre interaction with yday

We now model hatching rates as a function of *yday* and *distance.from.center*, including an interaction between the two variables. We fit separate models for Losone and Ascona.

```
gamm.hatched.Losone.te <- gamV(cbind(hatched, non.hatched) ~
                                 te(yday, distance.from.center) +

                                 s(unique.ID, bs = "re"),
                               family = "quasibinomial",
                               data = d.hatching.Losone)
summary(gamm.hatched.Losone.te)
```

```
Family: quasibinomial
Link function: logit

Formula:
cbind(hatched, non.hatched) ~ te(yday, distance.from.center) +
    s(unique.ID, bs = "re")

Parametric coefficients:
                  Estimate      Std. Error  t value               Pr(>|t|)
(Intercept) 1.9485387378418 0.0823555707141 23.66007 < 0.0000000000000000222 ***
---
```

```
Signif. codes:  0 '***' 0.001 '**' 0.01 '*' 0.05 '.' 0.1 ' ' 1

Approximate significance of smooth terms:
                                        edf        Ref.df        F
te(yday,distance.from.center) 14.0095460435 16.6276937548 16.80337
s(unique.ID)                  14.9862729549 46.0000000000  0.58680
                                             p-value
te(yday,distance.from.center) < 0.000000000000000222 ***
s(unique.ID)                             0.0040725 **
---
Signif. codes:  0 '***' 0.001 '**' 0.01 '*' 0.05 '.' 0.1 ' ' 1

R-sq.(adj) =  0.543   Deviance explained = 57.9%
-REML = -88.193  Scale est. = 7.4144    n = 306
```

```r
##
##
gamm.hatched.Ascona.te <- gamV(cbind(hatched, non.hatched) ~
                                 te(yday, distance.from.center) +

                                 s(unique.ID, bs = "re"),
                               family = "quasibinomial",
                               data = d.hatching.Ascona)
summary(gamm.hatched.Ascona.te)
```

```
Family: quasibinomial
Link function: logit

Formula:
cbind(hatched, non.hatched) ~ te(yday, distance.from.center) +
    s(unique.ID, bs = "re")

Parametric coefficients:
                   Estimate      Std. Error  t value               Pr(>|t|)
(Intercept) 2.3620713402989 0.0791073084754 29.85908 < 0.000000000000000222 ***
---
Signif. codes:  0 '***' 0.001 '**' 0.01 '*' 0.05 '.' 0.1 ' ' 1

Approximate significance of smooth terms:
                                        edf       Ref.df        F
te(yday,distance.from.center) 11.0489879861 13.501670299 31.85989
s(unique.ID)                  18.3846964791 50.000000000  0.65609
                                             p-value
te(yday,distance.from.center) < 0.000000000000000222 ***
s(unique.ID)                             0.0028517 **
---
Signif. codes:  0 '***' 0.001 '**' 0.01 '*' 0.05 '.' 0.1 ' ' 1

R-sq.(adj) =   0.67   Deviance explained = 67.5%
-REML = -179.71  Scale est. = 7.8897    n = 359
```

```r
plot.gam(gamm.hatched.Losone.te, pages = 1)
```

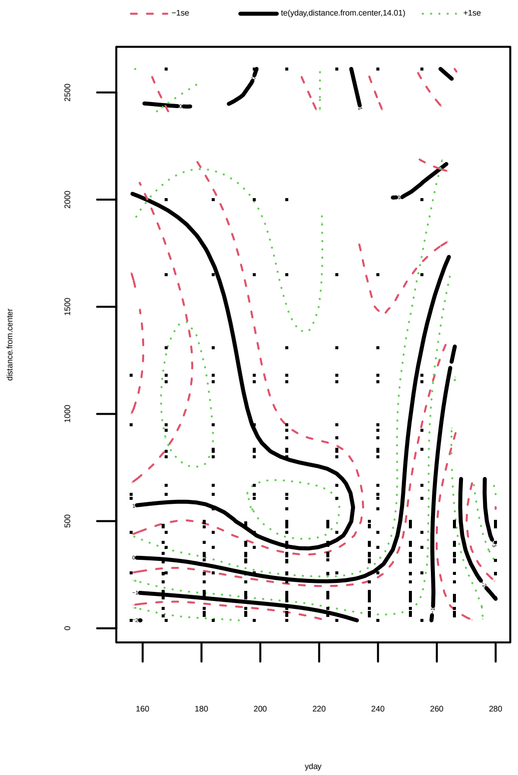

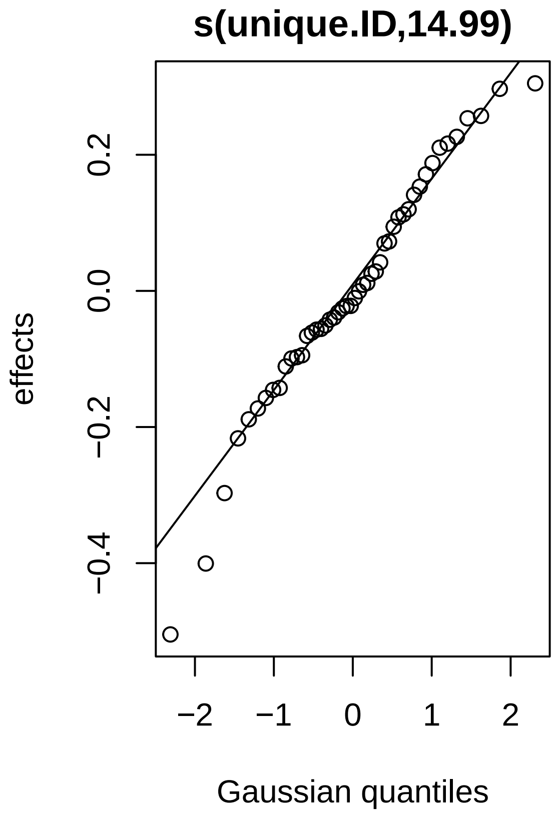


```r
plot.gamViz(gamm.hatched.Losone.te, select = 1)
```

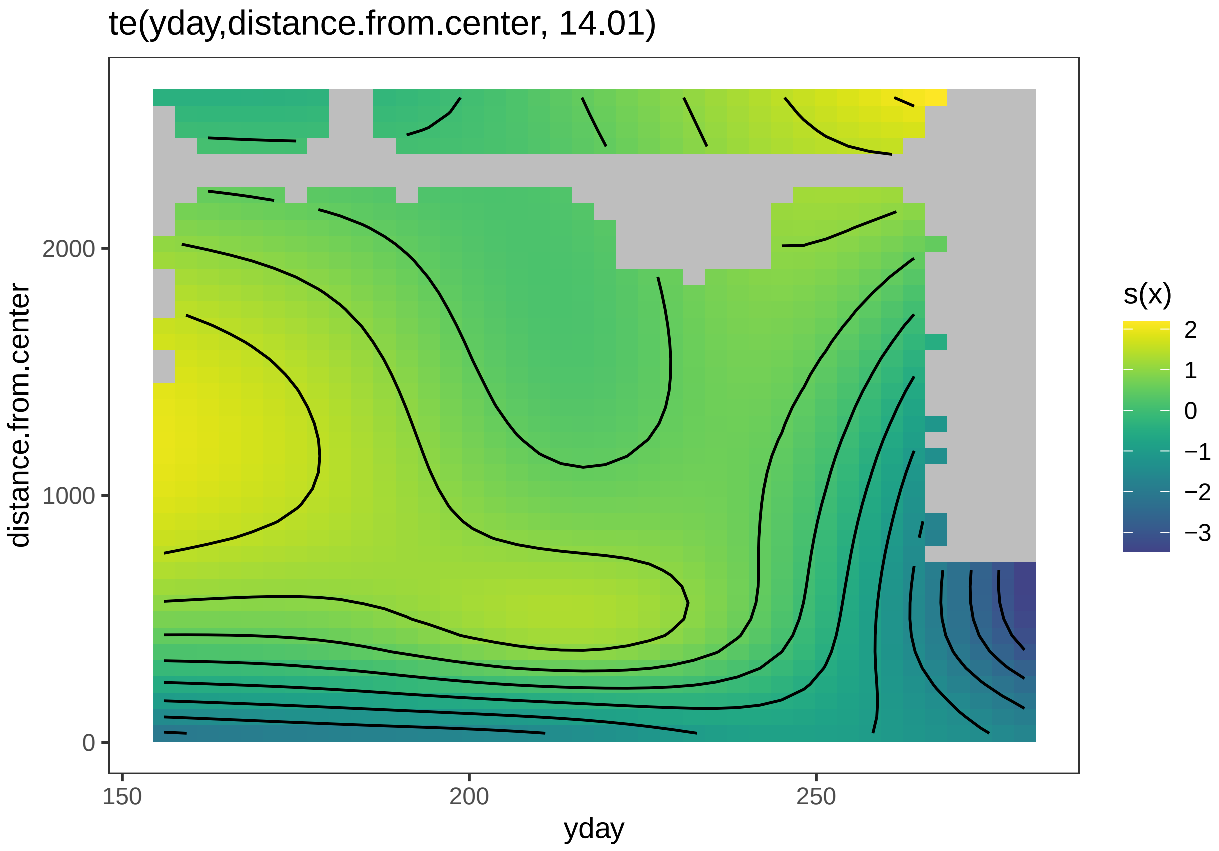

```
##
plot.gam(gamm.hatched.Ascona.te, pages = 1)
```

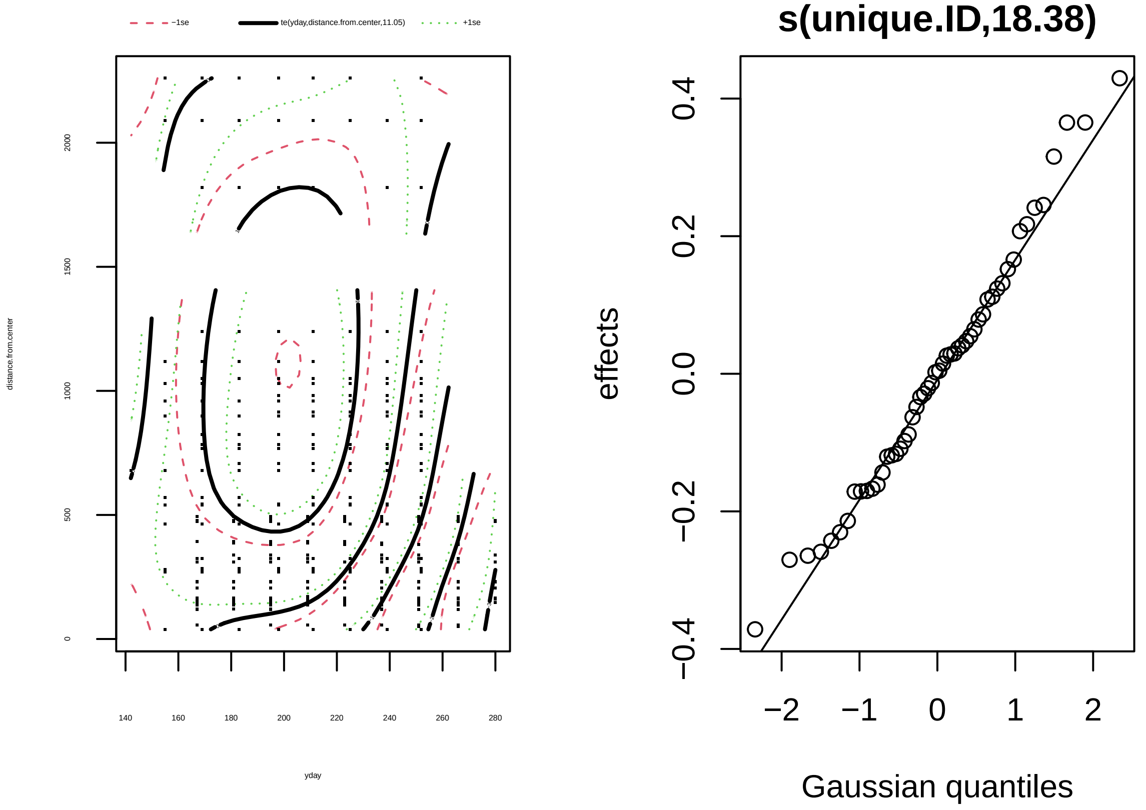


```
plot.gamViz(gamm.hatched.Ascona.te, select = 1)
```

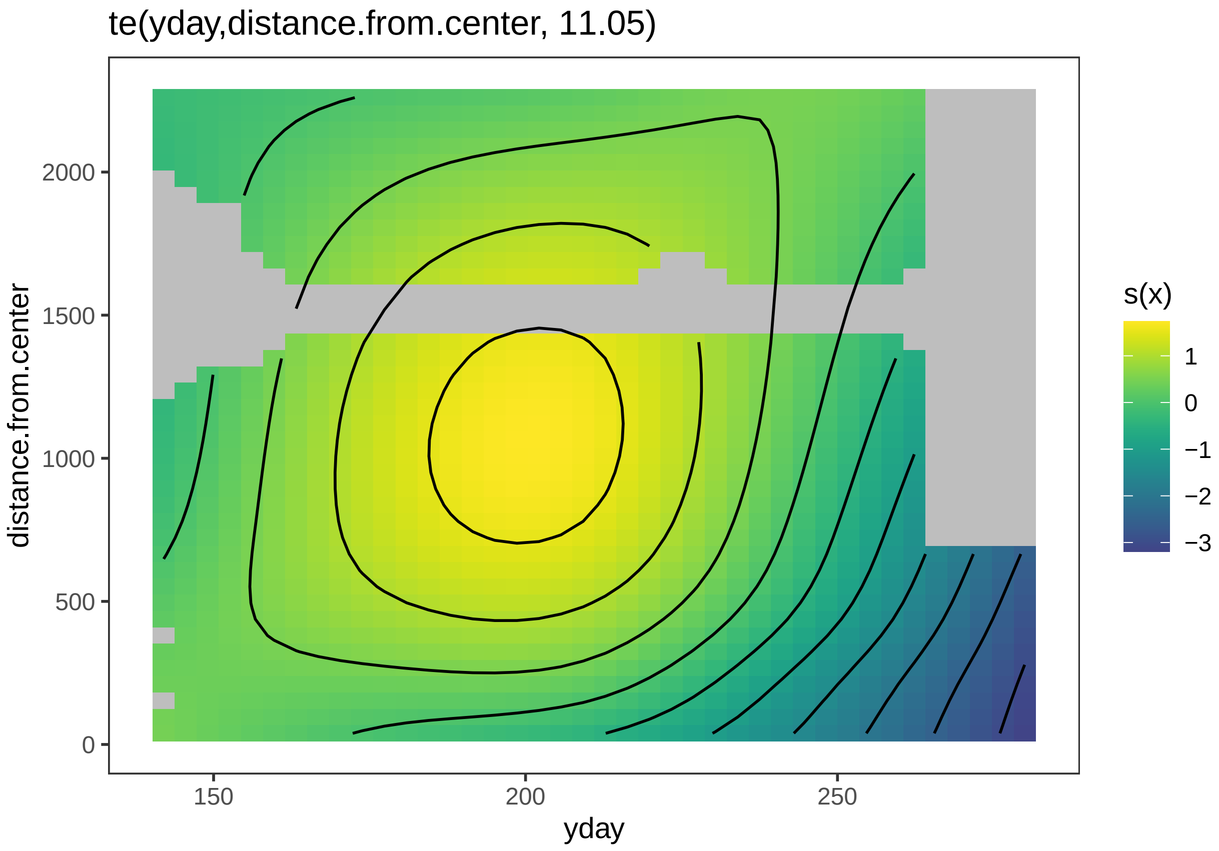

11.2.1 Fitted values

```
## Losone
d.hatching.Losone$fitted_gamm.hatching.te <-
  fitted(gamm.hatched.Losone.te)
## Ascona
d.hatching.Ascona$fitted_gamm.hatching.te <-
  fitted(gamm.hatched.Ascona.te)
##
d.ovitraps.fitted.new <- rbind(d.hatching.Losone,
                               d.hatching.Ascona) %>%
  mutate(percentile.distance.from.center = percent_rank(distance.from.center))
##
ggplot(data = d.ovitraps.fitted.new,
       mapping = aes(y = fitted_gamm.hatching.te,
                     x = yday,
                     group = unique.ID,
                     colour = percentile.distance.from.center)) +
  geom_vline(xintercept = yday("2025-06-17"), linetype = "dashed", colour = "grey") +
  geom_hline(yintercept = 0) +
  geom_point(alpha = 0.2) +
  geom_line(alpha = 0.2) +
  scale_y_sqrt() +
  facet_grid( ~ municipality.fac)
```

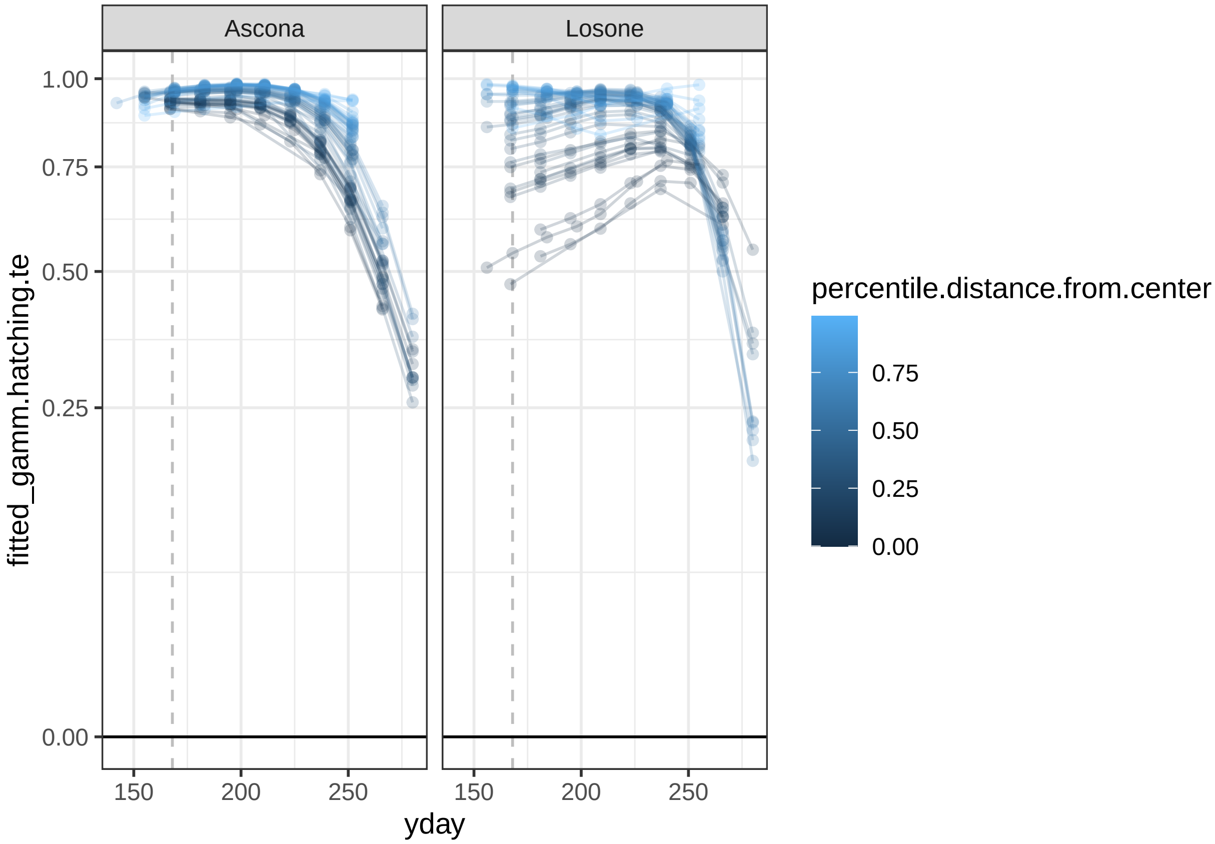

12 Session Information

```
sessionInfo()
```

```
R version 4.5.1 (2025-06-13)
Platform: aarch64-apple-darwin20
Running under: macOS Tahoe 26.1

Matrix products: default
BLAS:   /Library/Frameworks/R.framework/Versions/4.5-arm64/Resources/lib/libRblas.0.dylib
LAPACK: /Library/Frameworks/R.framework/Versions/4.5-arm64/Resources/lib/libRlapack.dylib;  LAPACK vers

locale:
[1] en_US.UTF-8/en_US.UTF-8/en_US.UTF-8/C/en_US.UTF-8/en_US.UTF-8

time zone: Europe/Zurich
tzcode source: internal

attached base packages:
[1] grid      stats     graphics  grDevices utils     datasets  methods
[8] base

other attached packages:
 [1] sf_1.0-21       stringr_1.5.1   ggpubr_0.6.1    ggplotify_0.1.2
 [5] readxl_1.4.5    mgcViz_0.2.0    qgam_2.0.0      mgcv_1.9-3
```

```
 [9] nlme_3.1-168    lubridate_1.9.4 ggplot2_3.5.2   dplyr_1.1.4
[13] groundhog_3.2.3 knitr_1.50

loaded via a namespace (and not attached):
 [1] tidyselect_1.2.1   viridisLite_0.4.2  farver_2.1.2       viridis_0.6.5
 [5] fastmap_1.2.0      GGally_2.2.1       promises_1.3.3     digest_0.6.37
 [9] timechange_0.3.0   mime_0.13          lifecycle_1.0.4    magrittr_2.0.3
[13] compiler_4.5.1     rlang_1.1.6        tools_4.5.1        utf8_1.2.6
[17] yaml_2.3.10        ggsignif_0.6.4     labeling_0.4.3     classInt_0.4-11
[21] plyr_1.8.9         RColorBrewer_1.1-3 abind_1.4-8        KernSmooth_2.23-26
[25] gamm4_0.2-7        miniUI_0.1.2       withr_3.0.2        purrr_1.0.4
[29] e1071_1.7-16       xtable_1.8-4       scales_1.4.0       iterators_1.0.14
[33] MASS_7.3-65        isoband_0.2.7      tinytex_0.57       cli_3.6.5
[37] rmarkdown_2.29     reformulas_0.4.1   generics_0.1.4     rstudioapi_0.17.1
[41] proxy_0.4-27       DBI_1.2.3          minqa_1.2.8        splines_4.5.1
[45] parallel_4.5.1     cellranger_1.1.0   matrixStats_1.5.0  vctrs_0.6.5
[49] yulab.utils_0.2.0  boot_1.3-31        Matrix_1.7-3       carData_3.0-5
[53] car_3.1-3          gridGraphics_0.5-1 rstatix_0.7.2      Formula_1.2-5
[57] foreach_1.5.2      tidyr_1.3.1        units_0.8-7        glue_1.8.0
[61] nloptr_2.2.1       ggstats_0.9.0      codetools_0.2-20   cowplot_1.1.3
[65] stringi_1.8.7      gtable_0.3.6       later_1.4.2        lme4_1.1-37
[69] tibble_3.3.0       pillar_1.10.2      htmltools_0.5.8.1  R6_2.6.1
[73] Rdpack_2.6.4       doParallel_1.0.17  evaluate_1.0.4     shiny_1.11.0
[77] lattice_0.22-7     rbibutils_2.3      backports_1.5.0    broom_1.0.8
[81] httpuv_1.6.16      class_7.3-23       Rcpp_1.0.14        gridExtra_2.3
[85] xfun_0.52          fs_1.6.6           pkgconfig_2.0.3
```
