# Supplementary material for: Assessing the Sterile Insect Technique in Small, Non-Isolated Urban Areas: A Field Trial Against Aedes albopictus in Southern Switzerland
Source: Insects. 2026 Jul 31;17(8):793. doi: 10.3390/insects17080793 (PMC13513938; doi:10.3390/insects17080793)
Supplement: Supplementary file 1 [file insects-17-00793-s001.zip › File_S3.pdf]

Modelling: SIT Losone & Ascona

Female counts

Author: Nisia Trisconi | Zurich Data Scientists
Reviewer: Dr. Matteo Tanadini | Zurich Data Scientists

December 5, 2025

Contents

1 Freeze Package versions **2**

2 Settings **2**

3 Getting data **3**

4 Aim and design **4**

5 Generalised Additive Mixed-Effects Model – Females (GAMM) **5**
5.1 Visualising the data 5
5.2 Fitting the models 10
5.2.1 Model for Losone 11
5.2.2 Model for Ascona 13
5.3 Plotting the smoothers 14
5.3.1 Losone 15
5.3.2 Ascona 18
5.4 Model selection 21
5.4.1 Losone 21
5.4.2 Ascona 25
5.5 Fitted values 29
5.6 Predicted values 30
5.6.1 Plotting the differences 35
5.7 Residual analysis 51

6 Methods **64**

7 Results **64**

8 Conclusions **64**

9 References **66**

1 Freeze Package versions

```
## (messages are omitted from this chunk)
##
library(groundhog)
pkgs <- c("dplyr",
          "ggplot2",
          "lubridate",
          "mgcViz")
groundhog.library(pkgs, date = "2025-07-01")
```

2 Settings

Global settings:

```
Sys.setenv(lang = "en_US")
theme_set(theme_bw())

if (!dir.exists("Prepared_data_and_models")) {
  dir.create("Prepared_data_and_models")
}
```

3 Getting data

This analysis concentrates on female counts in Losone and Ascona for the year 2025. We only keep functioning BG traps.

We load the data set.

```
d.adults.tmp <- readRDS(file = paste0("Prepared_data_and_models/",
                                      "d.adults.23.24.25_PreparedData.RDS")) %>%
  filter(status.fac == "F") %>%
  droplevels()
##
d.adults <- d.adults.tmp %>%
  filter(municipality.fac %in% c("Losone", "Ascona")) %>%
  droplevels()
```

Let's check which municipalities we have.

```
d.adults %>%
  pull(municipality.fac) %>%
  unique()
```

```
[1] Ascona Losone
Levels: Ascona Losone
```

```
## makes sense
```

Overview of the data:

```
dim(d.adults)
```

```
[1] 90 36
```

```
head(d.adults)[1:min(ncol(d.adults), 30)]
```

```
# A tibble: 6 x 30
  Activation.time Ae.albopictus.female Ae.albopictus.male Cx.pipiens.female
            <dbl>                <dbl>              <dbl>             <dbl>
1               1                    0                  0                 1
2               1                    0                  0                 2
3               1                    1                  0                 1
4               1                    0                  0                 6
5               1                    0                  1                 2
6               1                    0                  0                 2
# i 26 more variables: Cx.pipiens.male <dbl>, Ae.japonicus.female <dbl>,
#   Ae.japonicus.male <dbl>, Ae.koreicus.female <dbl>, Ae.koreicus.male <dbl>,
#   Year <dbl>, X.num <dbl>, Y.num <dbl>, BG_round.fac <fct>,
#   Setting_date.date <date>, Sampling_date.date <date>,
#   municipality.fac <fct>, municipality.ord <ord>, ID_BG_trap.fac <fct>,
#   status.fac <fct>, Year.fac <fct>, Ae.albopictus.tot <dbl>,
#   Ae.albopictus.sex.ratio <dbl>, Ae.albopictus.male.prop <dbl>, ...
```

```
str(d.adults)
```

```
tibble [90 x 36] (S3: tbl_df/tbl/data.frame)
 $ Activation.time          : num [1:90] 1 1 1 1 1 1 1 1 1 1 ...
 $ Ae.albopictus.female     : num [1:90] 0 0 1 0 0 0 0 0 1 0 ...
 $ Ae.albopictus.male       : num [1:90] 0 0 0 0 1 0 0 0 0 0 ...
 $ Cx.pipiens.female        : num [1:90] 1 2 1 6 2 2 4 3 4 5 ...
 $ Cx.pipiens.male          : num [1:90] 1 2 1 0 0 1 0 4 2 0 ...
```

```
 $ Ae.japonicus.female        : num [1:90] 0 0 0 0 0 0 0 0 0 0 ...
 $ Ae.japonicus.male          : num [1:90] 0 0 0 0 0 0 0 0 0 0 ...
 $ Ae.koreicus.female         : num [1:90] 0 0 0 0 0 0 0 0 0 0 ...
 $ Ae.koreicus.male           : num [1:90] 0 0 0 0 0 0 0 0 0 0 ...
 $ Year                       : num [1:90] 2025 2025 2025 2025 2025 ...
 $ X.num                      : num [1:90] 703474 703366 703276 703132 703009 ...
 $ Y.num                      : num [1:90] 112071 112108 112130 112141 112158 ...
 $ BG_round.fac               : Factor w/ 9 levels "1","2","3","4",..: 1 1 1 1 1 1 1 1 1 1 ...
 $ Setting_date.date          : Date[1:90], format: "2025-06-16" "2025-06-16" ...
 $ Sampling_date.date         : Date[1:90], format: "2025-06-17" "2025-06-17" ...
 $ municipality.fac           : Factor w/ 2 levels "Ascona","Losone": 1 1 1 1 1 2 2 2 2 2 ...
 $ municipality.ord           : Ord.factor w/ 2 levels "Ascona"<"Losone": 1 1 1 1 1 2 2 2 2 2 ...
 $ ID_BG_trap.fac             : Factor w/ 10 levels "1","10","2","3",..: 1 3 4 5 6 7 8 9 10 2 ...
 $ status.fac                 : Factor w/ 1 level "F": 1 1 1 1 1 1 1 1 1 1 ...
 $ Year.fac                   : Factor w/ 1 level "2025": 1 1 1 1 1 1 1 1 1 1 ...
 $ Ae.albopictus.tot          : num [1:90] 0 0 1 0 1 0 0 0 1 0 ...
 $ Ae.albopictus.sex.ratio    : num [1:90] NaN NaN 0 NaN Inf ...
 $ Ae.albopictus.male.prop    : num [1:90] NaN NaN 0 NaN 1 NaN NaN NaN 0 NaN ...
 $ yday                       : num [1:90] 168 168 168 168 168 168 168 168 168 168 ...
 $ week                       : num [1:90] 24 24 24 24 24 24 24 24 24 24 ...
 $ unique.ID                  : Factor w/ 10 levels "Ascona.1","Ascona.2",..: 1 2 3 4 5 6 7 8 9 10 ...
 $ unique.ID.Year             : Factor w/ 10 levels "Ascona.1.2025",..: 1 2 3 4 5 6 7 8 9 10 ...
 $ super.municipality.year    : Factor w/ 2 levels "Ascona:2025",..: 1 1 1 1 1 2 2 2 2 2 ...
 $ osservazioni.adults        : chr [1:90] NA NA NA NA ...
 $ osservazioni.coordinates   : chr [1:90] "dentro area sit" "dentro area sit" "confine area sit" "area
 $ distance.from.center       : num [1:90] 8 115 205 329 499 5 97 217 346 504 ...
 $ zona.SIT.discrete.fac      : Factor w/ 3 levels "within SIT","buffer",..: 1 1 2 2 2 1 1 2 2 3 ...
 $ SIT.fac                    : Factor w/ 1 level "SIT": 1 1 1 1 1 1 1 1 1 1 ...
 $ zona.SIT.discrete.ord      : Ord.factor w/ 3 levels "within SIT"<"buffer"<..: 1 1 2 2 2 1 1 2 2 3 ..
 $ Year.ord                   : Ord.factor w/ 1 level "2025": 1 1 1 1 1 1 1 1 1 1 ...
 $ super.municipality.year.ord: Ord.factor w/ 2 levels "Ascona:2025"<..: 1 1 1 1 1 2 2 2 2 2 ...
```

4 Aim and design

The aim of this analysis is to determine whether the release of sterilised males in a given area helps reducing the number of mosquitoes. With this purpose in mind, we are going to compare the treated, buffer and untreated zones of Losone and Ascona to check whether we see any difference in the number of females.

In total, the data was collected on `9` different dates; Losone was sampled `9` times, whereas Ascona was sampled `9` times.

In each municipality there are 5 traps that were measured 9 times.

```r
table(d.adults$Setting_date.date, d.adults$municipality.fac)
```

```
            Ascona Losone
  2025-06-16      5      5
  2025-06-30      5      5
  2025-07-15      5      5
  2025-07-28      5      5
  2025-08-11      5      5
  2025-08-25      5      5
  2025-09-08      5      5
  2025-09-24      5      5
```

```
  2025-10-06       5       5
```

5 Generalised Additive Mixed-Effects Model – Females (GAMM)

5.1 Visualising the data

A large number of males were released by the researchers, so we cannot include the male count in the analysis when assessing the reduction in mosquito populations, as this would lead to inaccurate results. Therefore, only the number of females is monitored to evaluate whether the procedure is effective.

With this purpose in mind, we begin by displaying the number of Aa albopictus females found in the traps sampled throughout the season for the year 2025 (note that we have data only for this year). As we are dealing with count data, we apply a square-root transformation to the response variable *Ae.albopictus.female* to stabilise variance.

Furthermore, observations from the same BG trap are connected with a line to visualise trends over time. On top of these lines, we add a smoothed average that allows us to see the global trend.

We divide the observations based on the municipality and add a dashed vertical line indicating when the treatment started (i.e., 17.06.2025).

```r
## (messages and warnings are excluded from this chunk)
##
## check that we have data only for the year 2025
unique(d.adults$Year.fac)
```

```
[1] 2025
Levels: 2025
```

```r
## makes sense
##
gg.adults.Losone <- ggplot(
  d.adults,
  mapping = aes(y = `Ae.albopictus.female`,
              x = Sampling_date.date)) +
  geom_hline(yintercept = 0, colour = "grey") +
  geom_vline(xintercept = ymd("2025-06-17"),
           linetype = "dashed", colour = "grey") +
  geom_point(alpha = 0.2,
           mapping = aes(group = ID_BG_trap.fac)) +
  geom_line(alpha = 0.2,
          mapping = aes(group = ID_BG_trap.fac)) +
  scale_y_sqrt(breaks = c(0, 2, 5, 10)) +
  coord_cartesian(ylim = c(0, NA)) +
  facet_wrap(~ municipality.fac)
##
gg.adults.Losone +
  geom_smooth(mapping = aes(group = NULL))
```

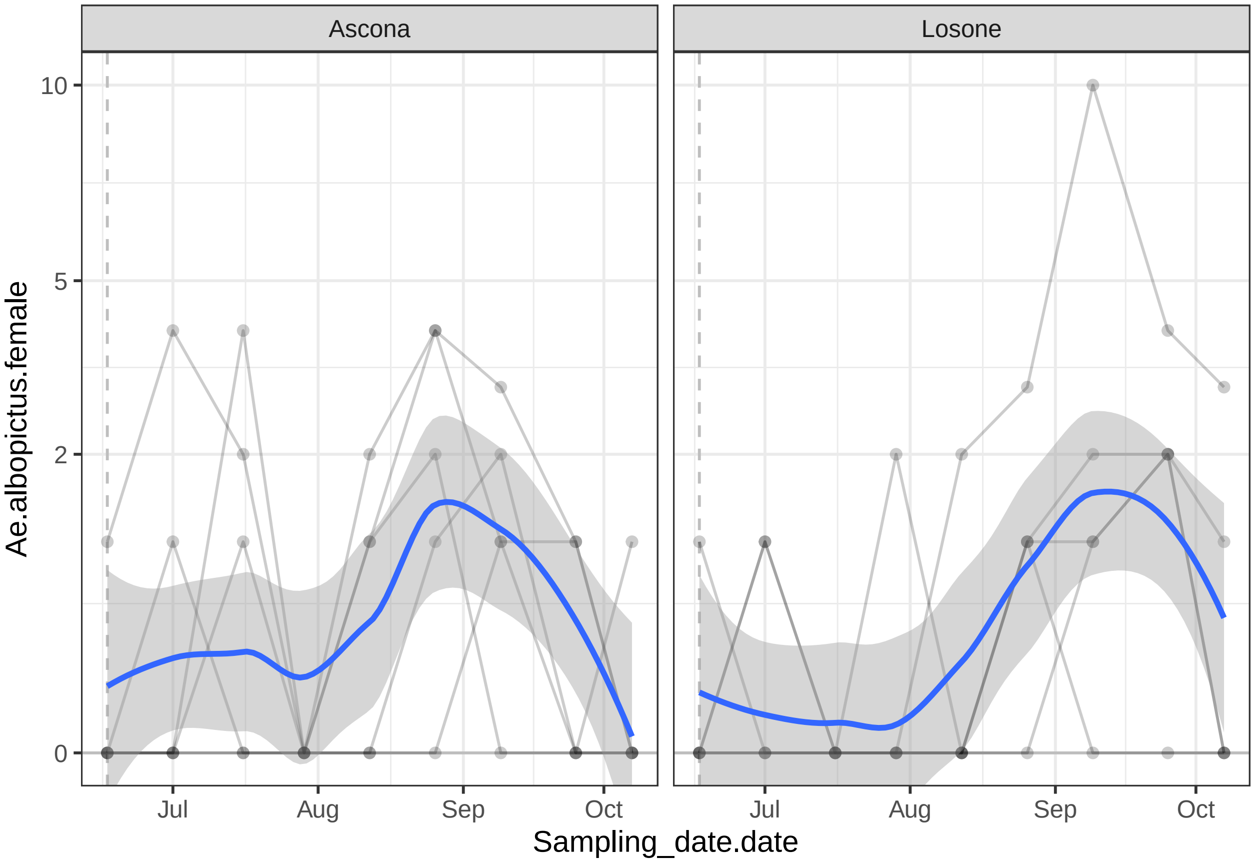

The number of Ae albopictus females seems to be constantly low until August for both municipalities, and then increase.

We also plot the 3 different zones separately as we will then model.

```
gg.adults.Losone +
  geom_smooth(mapping = aes(colour = zona.SIT.discrete.fac)) +
  theme(legend.position = "bottom")
```

```
`geom_smooth()` using method = 'loess' and formula = 'y ~ x'
```

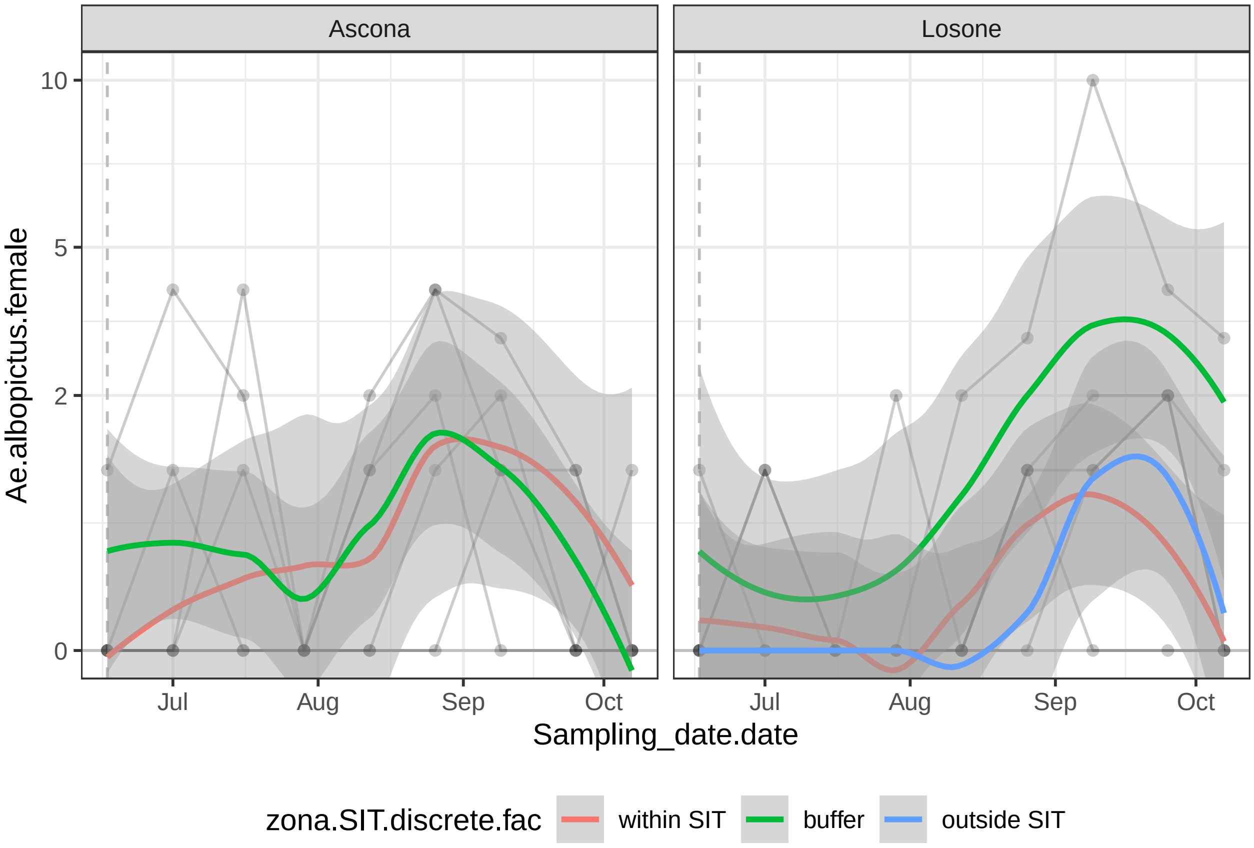

We can already see that the smoothers (loess here) cannot be estimated with high precision. On the one hand due to the low counts and on the other hand due to the low number of GAT used (5 in each municipality). Note also that Ascona has only observations within the SIT zone and in the buffer, not outside the SIT zone.

Note that there are only a few BG traps in each zone and municipality combination.

```
d.adults %>%
  select(municipality.fac, zona.SIT.discrete.fac, unique.ID) %>%
  unique()
```

```
# A tibble: 10 x 3
   municipality.fac zona.SIT.discrete.fac unique.ID
   <fct>            <fct>                 <fct>
 1 Ascona           within SIT            Ascona.1
 2 Ascona           within SIT            Ascona.2
 3 Ascona           buffer                Ascona.3
 4 Ascona           buffer                Ascona.4
 5 Ascona           buffer                Ascona.5
 6 Losone           within SIT            Losone.6
 7 Losone           within SIT            Losone.7
 8 Losone           buffer                Losone.8
 9 Losone           buffer                Losone.9
10 Losone           outside SIT           Losone.10
```

Losone outside SIT has only one BG trap. Let's visualise the data per zone and municipality combination in separate panels.

```
gg.adults.Losone +
  geom_smooth(mapping = aes(colour = zona.SIT.discrete.fac))  +
  facet_grid(zona.SIT.discrete.fac ~ municipality.fac)
```

```
`geom_smooth()` using method = 'loess' and formula = 'y ~ x'
```

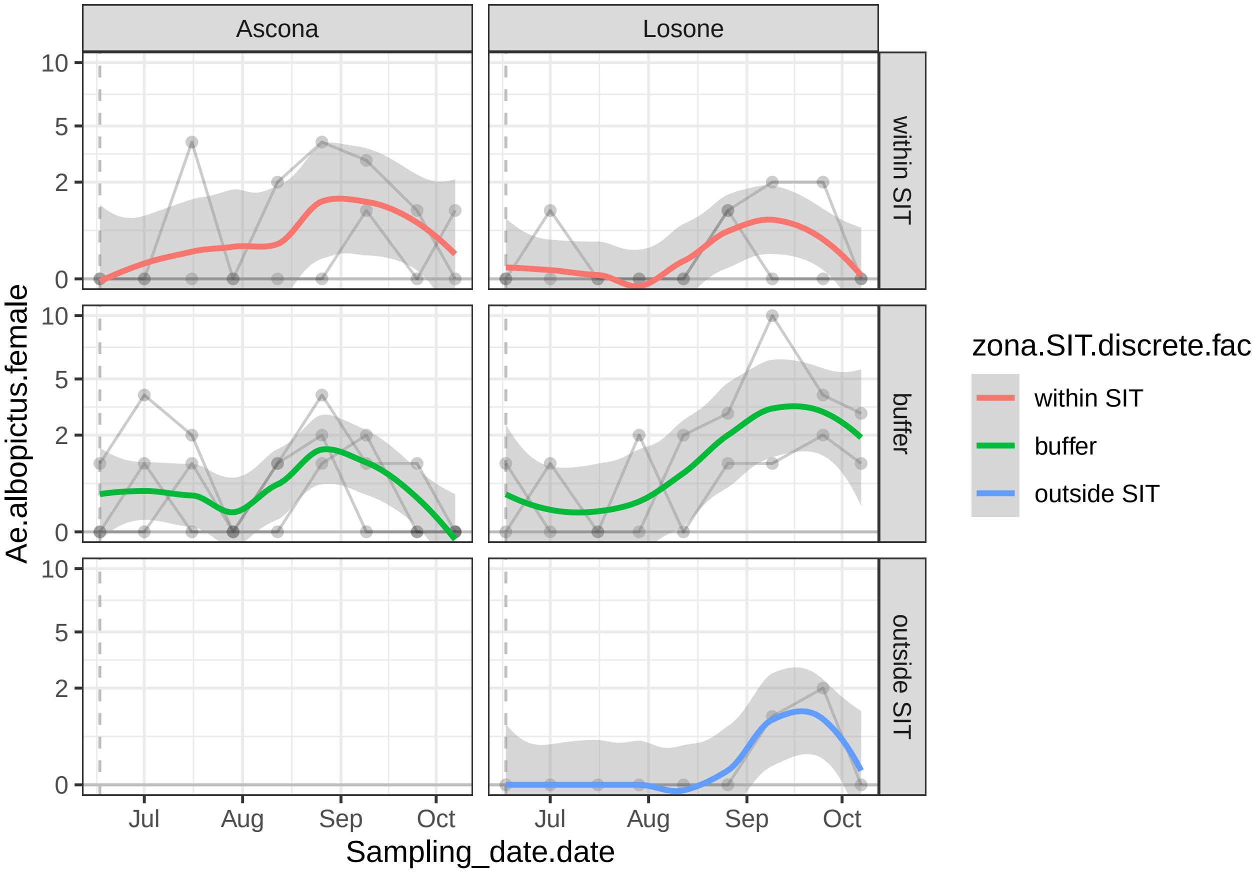

Last but not least, note that adults are surveyed only once the experiment has begun. Starting the monitoring earlier would have allowed us to establish a better baseline control (the same for all conditions). However, it is also true that early in the season the adult counts are zero or close to zero anyway, which would not provide a substantial amount of additional information to better characterise each BG-trap location.

Another interesting aspect to explore is the relationship between number of females and the distance from the release centre. The idea behind this is to understand how large the treated area needs to be in order to observe an effect and, if an effect is detected, how far beyond the treated area it propagates.

We create a scatter plot of *Ae.albopictus.female* against *distance.from.center*, grouping the points by treatment zone. A smoothed average is added to visualise potential trends.

```
## (warnings are omitted from this chunk)
##
gg.distance <- d.adults %>%
  ggplot(mapping = aes(x = distance.from.center,
                       y = Ae.albopictus.female,
  )) +
  geom_hline(yintercept = c(0), colour = "grey") +
  geom_point(alpha = 0.2, mapping = aes(colour = zona.SIT.discrete.fac)) +
  facet_wrap(~ municipality.fac) +
```

```r
  geom_smooth(alpha = 0.3, method = "loess",
              method.args = list(family = "symmetric")) +
  scale_colour_manual(values = c("blue", "green3", "red")) +
  coord_cartesian(ylim = c(0, NA)) +
  labs(colour = "Zone") +
  theme(legend.position = "bottom")
gg.distance
```

```
`geom_smooth()` using formula = 'y ~ x'
```

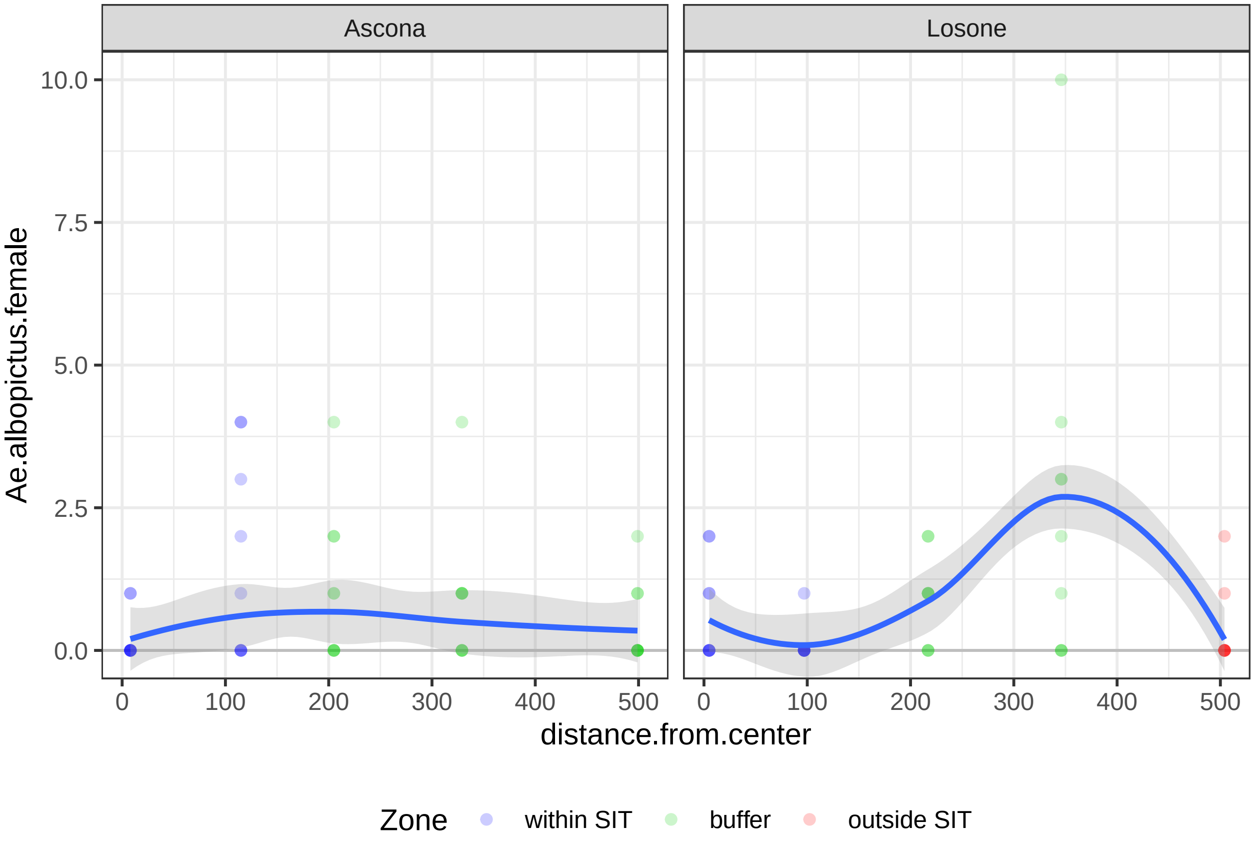


```r
##
## apply sqrt to both axes
gg.distance +
  scale_x_sqrt() + ## log(x) would be theory, but leads to a bad plot here
  scale_y_sqrt(breaks = c(0, 2, 5, 10))
```

```
`geom_smooth()` using formula = 'y ~ x'
```

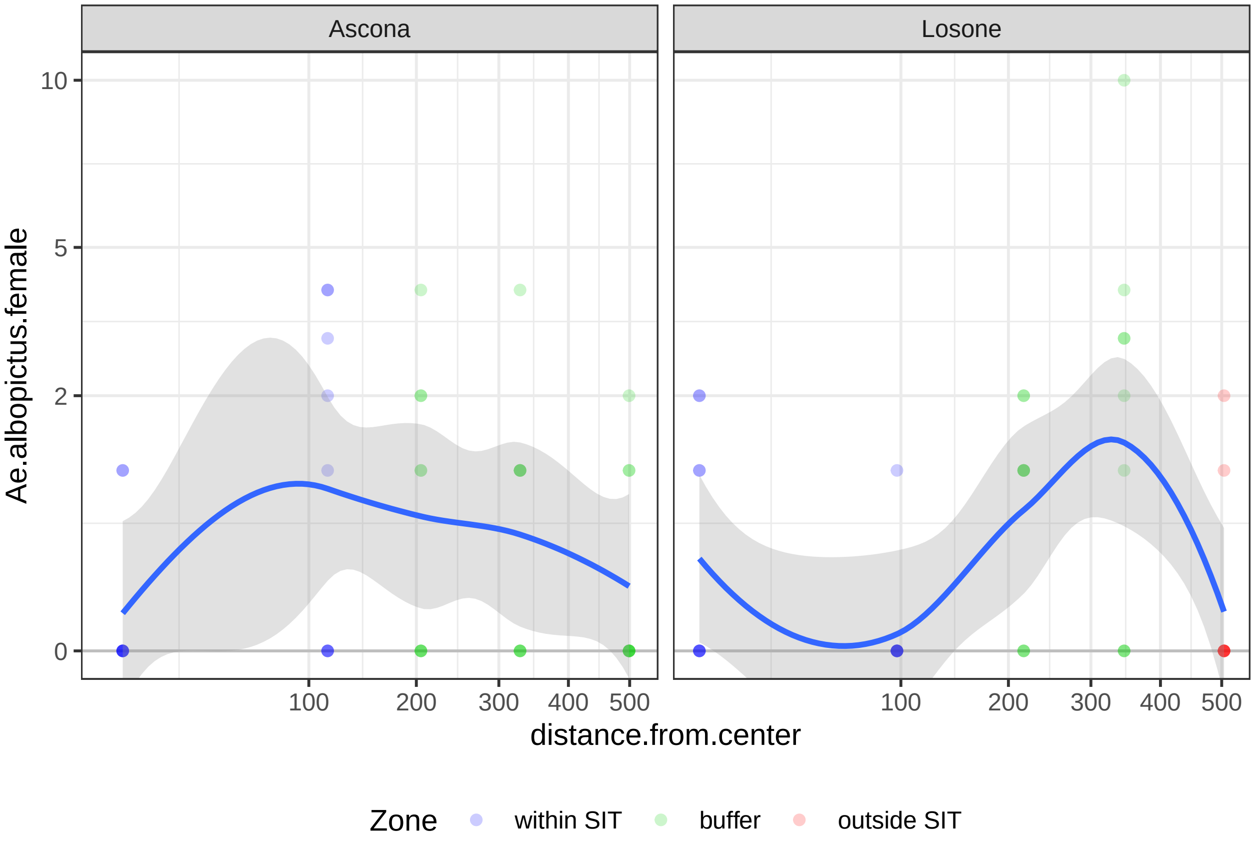

The effect is not clear from these plots.

5.2 Fitting the models

We aim to model the total number of *Aedes albopictus* females (*Ae.albopictus.females*) over the season (*yday*), represented by the day of the year (*yday*). Separate models are fitted for the two municipalities, as their seasonal patterns might differ. This approach avoids the need for an overly complex model that would allow different trend shapes depending on the municipality.

Because *Ae.albopictus.females* represents count data, we employ a negative binomial model to account for overdispersion, which is common in such data.

As already mentioned, our goal is to assess whether female counts differ among treatment zones. The treatment zone may not only influence the magnitude of these counts but also modify the shape of their seasonal trend. To capture this, we treat *yday* as a numeric covariate whose effect is allowed to vary across treatment zones (*zona.SIT.discrete.fac*).

We use a Generalised Additive Model (GAM) to flexibly model seasonal dynamics without imposing a predefined functional form. This approach enhances model flexibility and captures complex temporal patterns, albeit at the cost of some interpretability.

Finally, to account for the lack of independence among observations from the same BG trap, we include *ID_BG_trap.fac* (the unique BG trap identifier) as a random effect. This adjustment helps to capture the variability specific to each BG trap.

Let's check how many sampling dates there are for each municipality.

```
d.adults %>%
  group_by(municipality.fac) %>%
```

```
  summarise(nr.knots = n_distinct(yday)) %>%
  arrange(nr.knots)
```

```
# A tibble: 2 x 2
  municipality.fac nr.knots
  <fct>               <int>
1 Ascona                  9
2 Losone                  9
```

We also check how many different activation times are present for each municipality.

```
table(d.adults$Activation.time, d.adults$municipality.fac)
```

```

    Ascona Losone
  1     45     45
```

```
## both municipalities have a single activation time
```

Both municipalities have a single activation time, equal to 1, therefore we don't need to include it in the model.

Let's check if we have any missing value for the relevant variables.

```
d.adults %>%
  select(Ae.albopictus.female,
         municipality.fac,
         ID_BG_trap.fac,
         yday,
         municipality.ord) %>%
  na.omit() %>%
  droplevels() %>%
  nrow()
```

```
[1] 90
```

```
##
## check
nrow(d.adults)
```

```
[1] 90
```

```
## no missing values
```

5.2.1 Model for Losone

We start by modelling Losone.

```
d.adults.Losone <- d.adults %>%
  filter(municipality.fac == "Losone")
```

We use the `gamV()` function instead of `gam()`, because it fits the `gam()` model and automatically converts it to a `gamViz` object, making visualisation easier.
The model's estimates are exactly the same.

```r
gamm.tot.females.Losone <- gamV(Ae.albopictus.female ~
                                  ## ranef
                                  s(ID_BG_trap.fac, bs = "re") +

                                  ## fixed effect
                                  zona.SIT.discrete.fac +

                                  ## smooth term with interaction
                                  s(yday, by = zona.SIT.discrete.fac, k = 9),

                                family = "nb",
                                method = "GCV.Cp", ## as gam function
                                data = d.adults.Losone)
summary(gamm.tot.females.Losone)
```

```
Family: Negative Binomial(4.042)
Link function: log

Formula:
Ae.albopictus.female ~ s(ID_BG_trap.fac, bs = "re") + zona.SIT.discrete.fac +
    s(yday, by = zona.SIT.discrete.fac, k = 9)

Parametric coefficients:
                                       Estimate      Std. Error  z value
(Intercept)                       -1.247248580718  0.757204569760 -1.64718
zona.SIT.discrete.facbuffer        1.394824612116  0.993020161898  1.40463
zona.SIT.discrete.facoutside SIT -9.451106623307 31.303012958819 -0.30192
                                  Pr(>|z|)
(Intercept)                       0.099522 .
zona.SIT.discrete.facbuffer       0.160132
zona.SIT.discrete.facoutside SIT 0.762711
---
Signif. codes:  0 '***' 0.001 '**' 0.01 '*' 0.05 '.' 0.1 ' ' 1

Approximate significance of smooth terms:
                                            edf        Ref.df  Chi.sq
s(ID_BG_trap.fac)                      1.47310888533 2.00000000000 5.52779
s(yday):zona.SIT.discrete.facwithin SIT  1.41430991036 1.71728930418 1.23820
s(yday):zona.SIT.discrete.facbuffer      1.73091594428 2.15602405738 7.96927
s(yday):zona.SIT.discrete.facoutside SIT 2.44520650479 2.85846234291 1.20080
                                         p-value
s(ID_BG_trap.fac)                      0.024412 *
s(yday):zona.SIT.discrete.facwithin SIT  0.399042
s(yday):zona.SIT.discrete.facbuffer      0.021106 *
s(yday):zona.SIT.discrete.facoutside SIT 0.765976
---
Signif. codes:  0 '***' 0.001 '**' 0.01 '*' 0.05 '.' 0.1 ' ' 1

R-sq.(adj) =  0.458   Deviance explained = 63.9%
-REML = 43.749  Scale est. = 1         n = 45
```

We also extract the standard deviation corresponding to the random effect.

```r
gam.vcomp(gamm.tot.females.Losone) %>%
  tail(n = 1)
```

```
Standard deviations and 0.95 confidence intervals:

                                               std.dev
s(ID_BG_trap.fac)                       0.81765052805156324
s(yday):zona.SIT.discrete.facwithin SIT  0.00498067991821263
s(yday):zona.SIT.discrete.facbuffer      0.00443489568170773
s(yday):zona.SIT.discrete.facoutside SIT 0.25981055951918491
                                                    lower
s(ID_BG_trap.fac)                       0.17917615558845381707
s(yday):zona.SIT.discrete.facwithin SIT  0.00000399033445083647
s(yday):zona.SIT.discrete.facbuffer      0.00013455105663798180
s(yday):zona.SIT.discrete.facoutside SIT 0.00823638142519204051
                                               upper
s(ID_BG_trap.fac)                       3.731257565089102
s(yday):zona.SIT.discrete.facwithin SIT  6.216815345512305
s(yday):zona.SIT.discrete.facbuffer      0.146177222231325
s(yday):zona.SIT.discrete.facoutside SIT 8.195531915412486

Rank: 4/4

                                             std.dev              lower
s(yday):zona.SIT.discrete.facoutside SIT 0.259810559519185 0.00823638142519204
                                              upper
s(yday):zona.SIT.discrete.facoutside SIT 8.19553191541249
```

5.2.2 Model for Ascona

We now model Ascona.

```r
d.adults.Ascona <- d.adults %>%
  filter(municipality.fac == "Ascona")
```

```r
gamm.tot.females.Ascona <- gamV(Ae.albopictus.female ~
                                  ## ranef
                                  s(ID_BG_trap.fac, bs = "re") +

                                  ## fixed effect
                                  zona.SIT.discrete.fac +

                                  ## smooth term with interaction
                                  s(yday, by = zona.SIT.discrete.fac, k = 9),

                                family = "nb",
                                method = "GCV.Cp", ## as gam function
                                data = d.adults.Ascona)
summary(gamm.tot.females.Ascona)
```

```
Family: Negative Binomial(1.66)
Link function: log
```

```
Formula:
Ae.albopictus.female ~ s(ID_BG_trap.fac, bs = "re") + zona.SIT.discrete.fac +
    s(yday, by = zona.SIT.discrete.fac, k = 9)

Parametric coefficients:
                                    Estimate       Std. Error  z value Pr(>|z|)
(Intercept)                  -0.5129403504876  0.6388168479098 -0.80295  0.42200
zona.SIT.discrete.facbuffer  0.0419793419666  0.8238263884093  0.05096  0.95936

Approximate significance of smooth terms:
                                               edf        Ref.df  Chi.sq
s(ID_BG_trap.fac)                        2.04977646448 3.00000000000 5.30897
s(yday):zona.SIT.discrete.facwithin SIT 1.95590789903 2.44868112287 2.47122
s(yday):zona.SIT.discrete.facbuffer     3.26250971296 4.06605489211 4.59474
                                         p-value
s(ID_BG_trap.fac)                        0.051069 .
s(yday):zona.SIT.discrete.facwithin SIT 0.362485
s(yday):zona.SIT.discrete.facbuffer     0.369346
---
Signif. codes:  0 '***' 0.001 '**' 0.01 '*' 0.05 '.' 0.1 ' ' 1

R-sq.(adj) =  0.212   Deviance explained = 37.5%
-REML = 55.675  Scale est. = 1         n = 45
```

We also extract the standard deviation corresponding to the random effect.

```r
gam.vcomp(gamm.tot.females.Ascona) %>%
  tail(n = 1)
```

```
Standard deviations and 0.95 confidence intervals:

                                                 std.dev               lower
s(ID_BG_trap.fac)                        0.72320523377763424 0.19603364969507103
s(yday):zona.SIT.discrete.facwithin SIT 0.00813247937619777 0.00103418300176683
s(yday):zona.SIT.discrete.facbuffer     0.01877299426875149 0.00150966039570510
                                                 upper
s(ID_BG_trap.fac)                        2.6680409765207429
s(yday):zona.SIT.discrete.facwithin SIT 0.0639511775878074
s(yday):zona.SIT.discrete.facbuffer     0.2334467505521158

Rank: 3/3

                                              std.dev              lower
s(yday):zona.SIT.discrete.facbuffer 0.0187729942687515 0.0015096603957051
                                               upper
s(yday):zona.SIT.discrete.facbuffer 0.233446750552116
```

5.3 Plotting the smoothers

We now plot the fitted smoothers.

5.3.1 Losone

```
plot.gam(gamm.tot.females.Losone,
         pages = 1,
         trans = exp,
         ylim = c(0, 10))
```

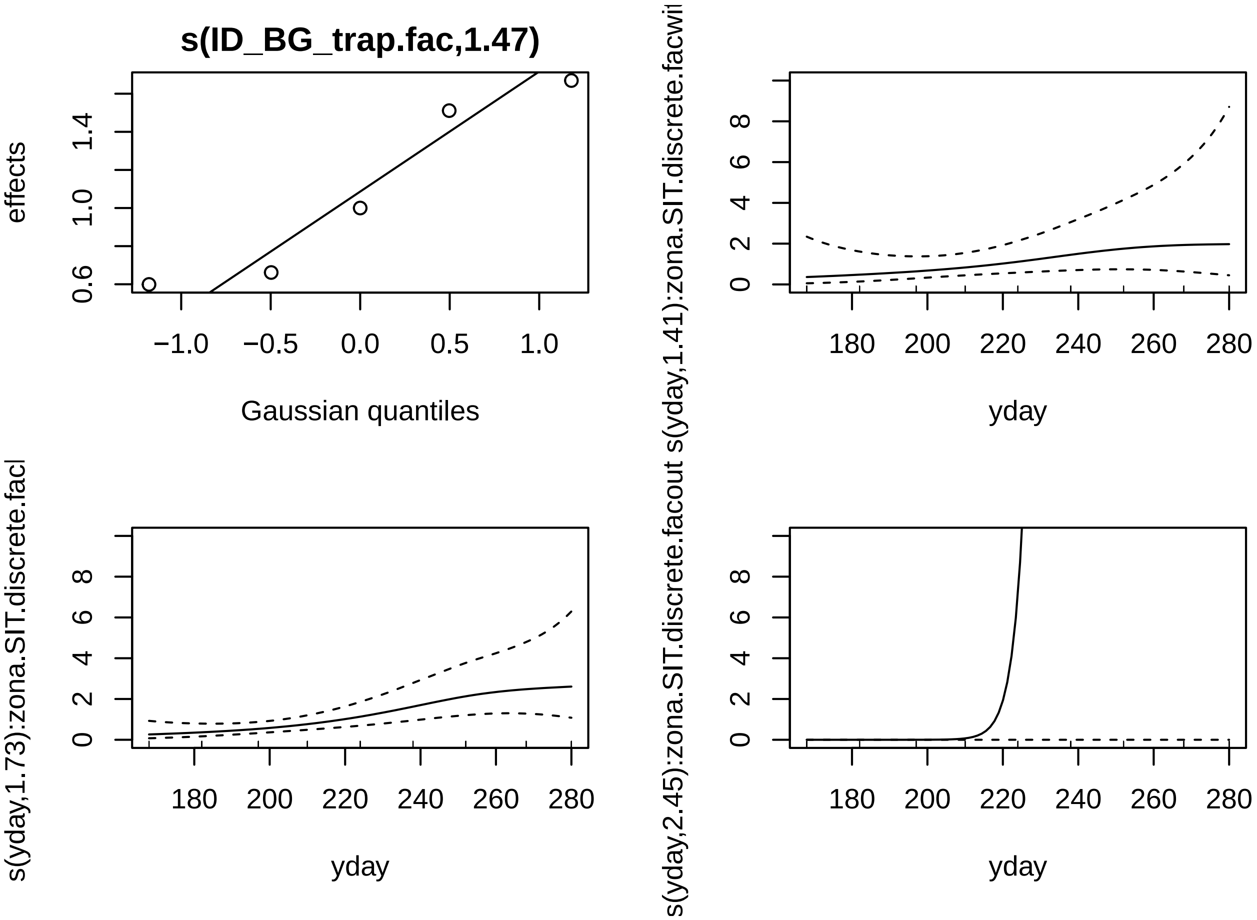

We add the corresponding shift.

```
## Within SIT
plot.gam(gamm.tot.females.Losone,
         select = 2,
         shift = gamm.tot.females.Losone$coefficients["(Intercept)"],
         trans = exp,
         ylim = c(0, 4))
```

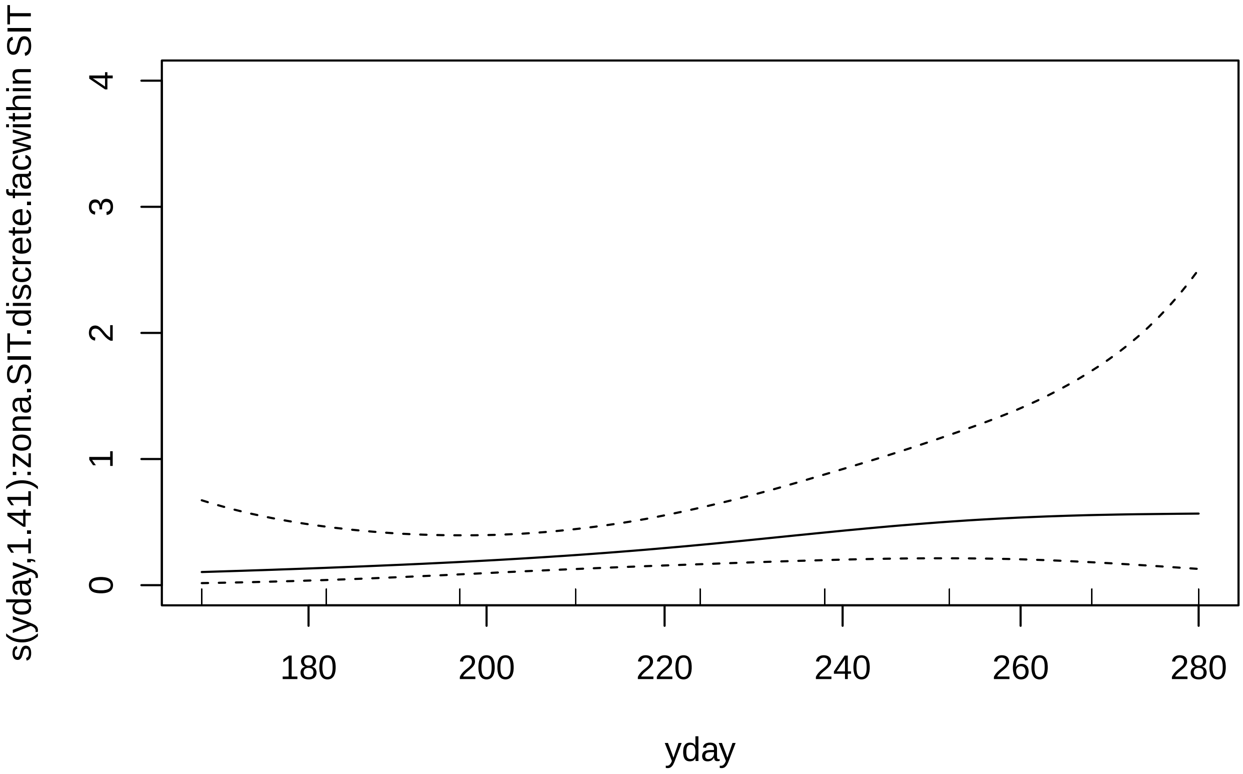


```r
##
## buffer
plot.gam(gamm.tot.females.Losone,
         select = 3,
         shift = gamm.tot.females.Losone$coefficients["(Intercept)"] +
           gamm.tot.females.Losone$coefficients["zona.SIT.discrete.facbuffer"],
         trans = exp,
         ylim = c(0, 4))
```

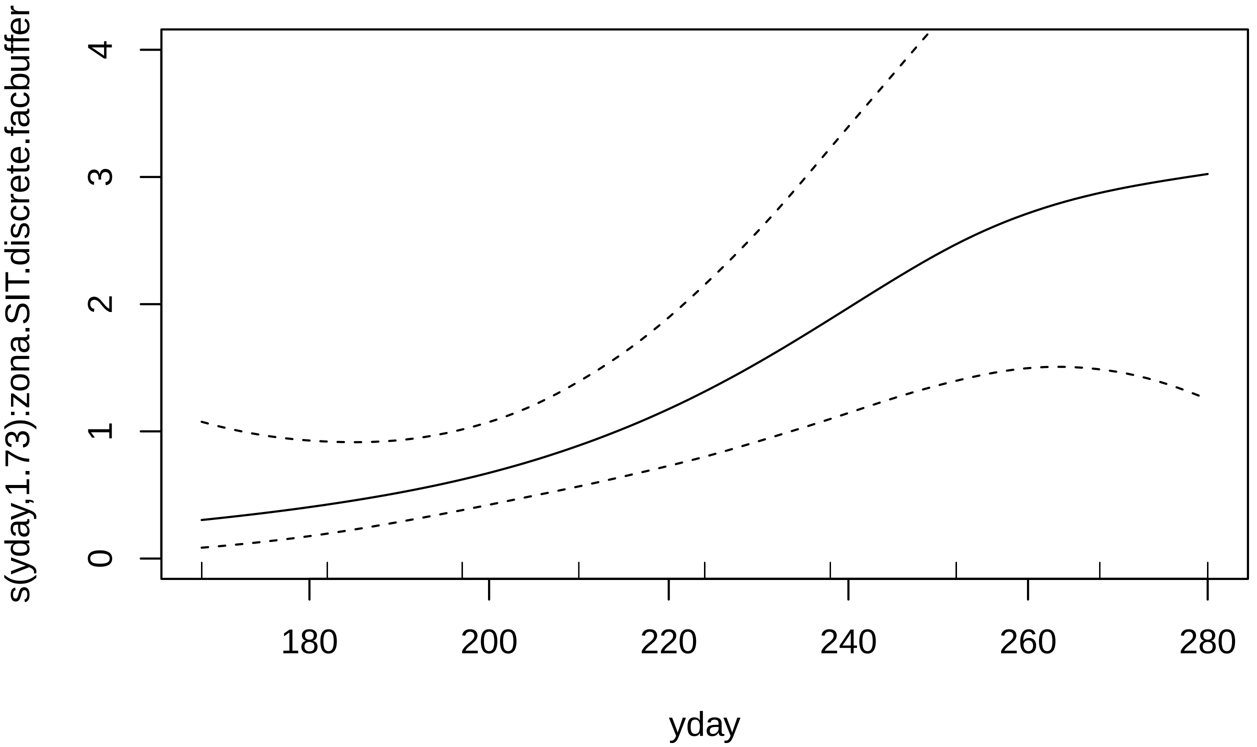


```
##
## outside SIT
plot.gam(gamm.tot.females.Losone,
         select = 4,
         shift = gamm.tot.females.Losone$coefficients["(Intercept)"] +
           gamm.tot.females.Losone$coefficients["zona.SIT.discrete.facoutside SIT"],
         trans = exp,
         ylim = c(0, 4))
```

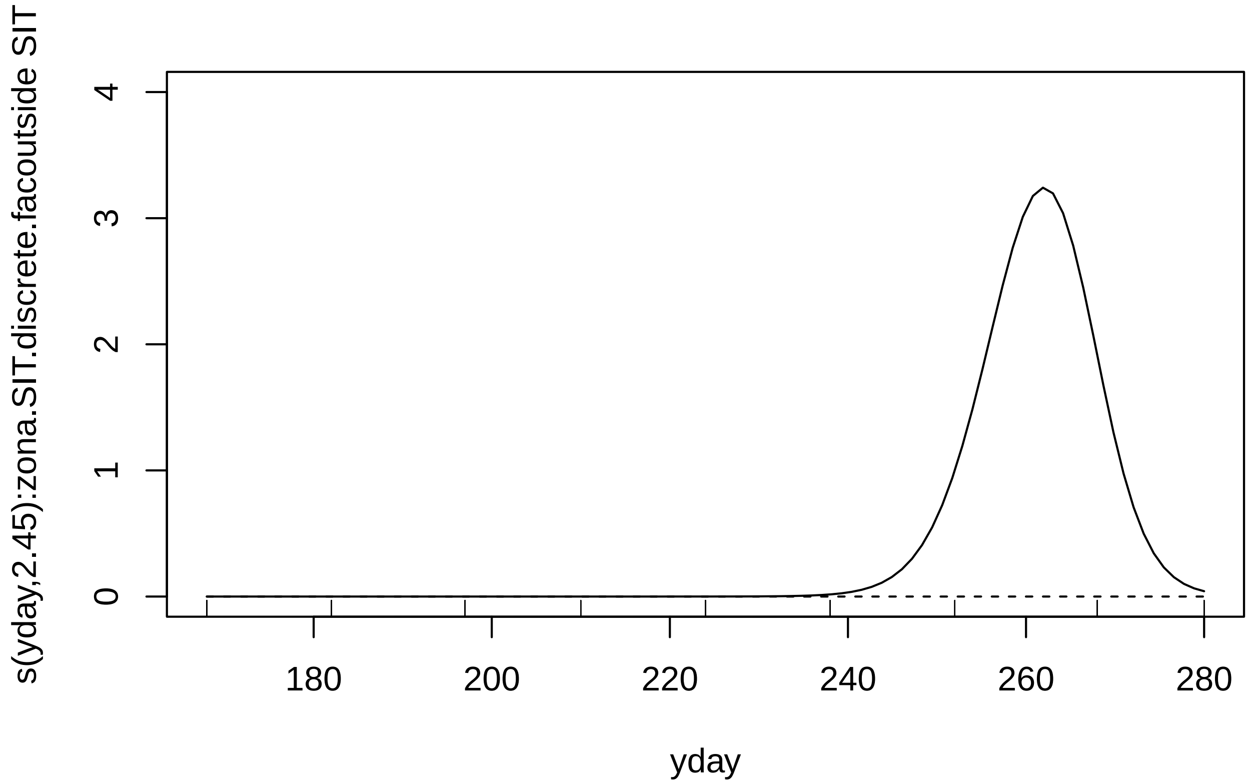

The smoother for Losone "outside SIT" is estimate poorly, due to the fact that most of it observations are zero and that it only contains as single BG trap.

5.3.2 Ascona

```
plot.gam(gamm.tot.females.Ascona,
         pages = 1,
         trans = exp,
         ylim = c(0, 10))
```

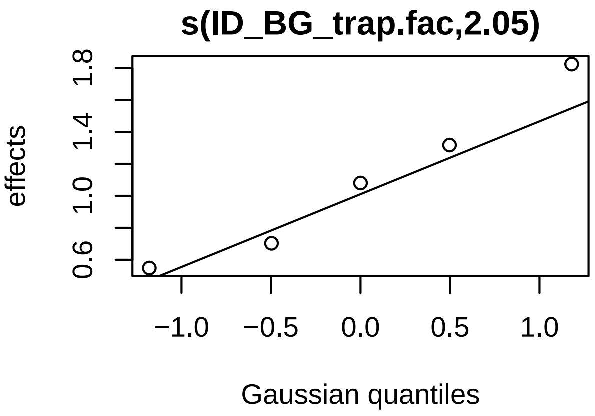

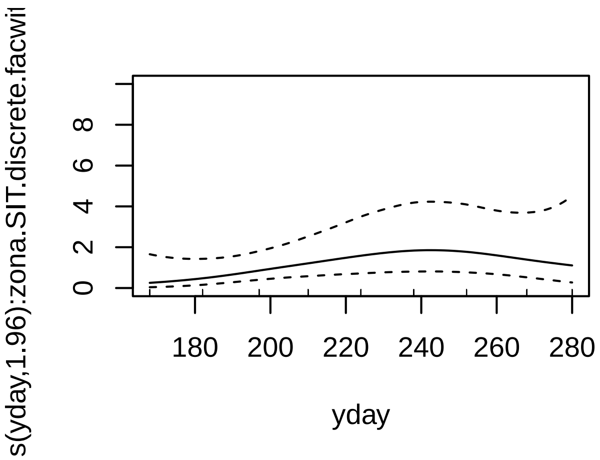

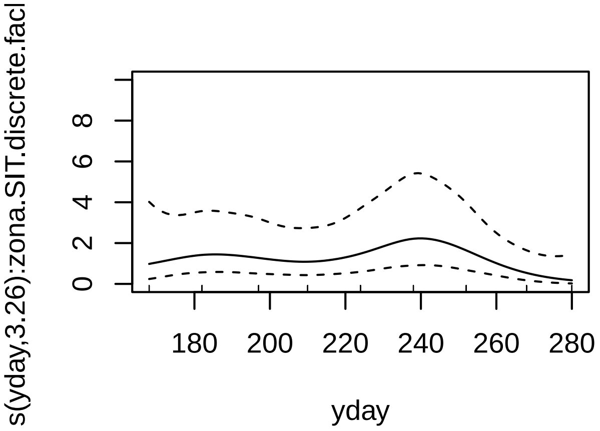

We add the corresponding shift.

```
## Within SIT
plot.gam(gamm.tot.females.Ascona,
         select = 2,
         shift = gamm.tot.females.Ascona$coefficients["(Intercept)"],
         trans = exp,
         ylim = c(0, 4))
```

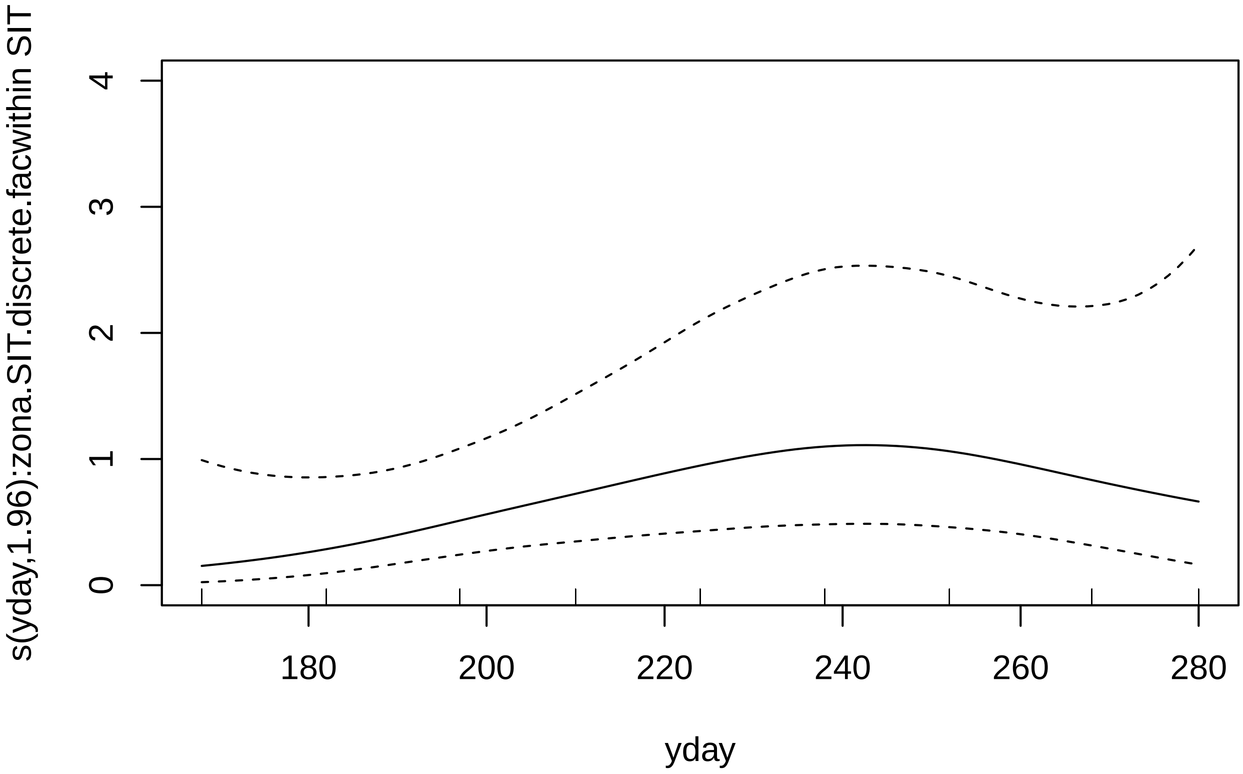


```r
##
## buffer
plot.gam(gamm.tot.females.Ascona,
         select = 3,
         shift = gamm.tot.females.Ascona$coefficients["(Intercept)"] +
           gamm.tot.females.Ascona$coefficients["zona.SIT.discrete.facbuffer"],
         trans = exp,
         ylim = c(0, 4))
```

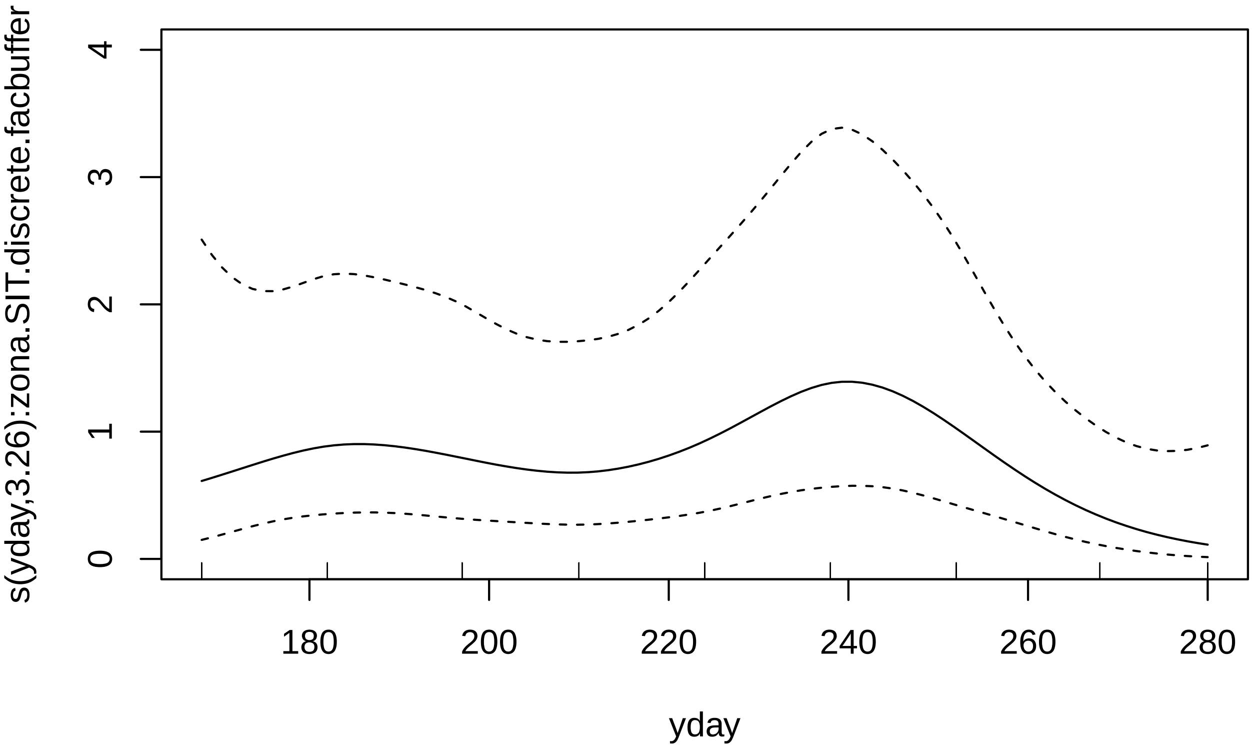


```
##
## outside is not available for Ascona
```

5.4 Model selection

We fitted a model that allows the seasonal trend to vary across treatment zones by including zone-specific smoothers. We now test whether distinct smoothers are necessary.

5.4.1 Losone

For comparing the two models, we need to refit the first model using an ordered factor (*zona.SIT.discrete.ord*) instead of the classical one. This allows us to have nested model matrices and formally compare the two models.

```
## (this chunk is not evaluated)
gamm.tot.females.Losone.ord <- gamV(Ae.albopictus.female ~

                                      ## Ranef
                                      s(ID_BG_trap.fac, bs = "re") +

                                      ## smooth term
                                      s(yday, k = 9) +

                                      ## Fixed effect
                                      zona.SIT.discrete.fac +


                                      ## smooth term with interaction
                                      s(yday, by = zona.SIT.discrete.ord, k = 9),
```

```
                                    family = "nb",
                                    method = "GCV.Cp", ## as gam function
                                    data = d.adults.Losone)
##
gamm.tot.females.Losone_one.smooth <- gamV(Ae.albopictus.female ~

                                             ## Ranef
                                             s(ID_BG_trap.fac, bs = "re") +

                                             ## smooth term
                                             s(yday, k = 9) +

                                             ## Fixed effect
                                             zona.SIT.discrete.fac, # +

                                           ## smooth term with interaction
                                           # s(yday, by = zona.SIT.discrete.ord, k = 9),
                                           family = "nb",
                                           method = "GCV.Cp", ## as gam function
                                           data = d.adults.Losone)
```

```
summary(gamm.tot.females.Losone.ord)
```

```
Family: Negative Binomial(74.47)
Link function: log

Formula:
Ae.albopictus.female ~ s(ID_BG_trap.fac, bs = "re") + s(yday,
    k = 9) + zona.SIT.discrete.fac + s(yday, by = zona.SIT.discrete.ord,
    k = 9)

Parametric coefficients:
                                        Estimate      Std. Error  z value
(Intercept)                        -1.336980060593  0.782726012851 -1.70811
zona.SIT.discrete.facbuffer         1.394738398609  1.024501907742  1.36138
zona.SIT.discrete.facoutside SIT -8.590747007010 24.478586487291 -0.35095
                                 Pr(>|z|)
(Intercept)                      0.087616 .
zona.SIT.discrete.facbuffer      0.173393
zona.SIT.discrete.facoutside SIT 0.725626
---
Signif. codes:  0 '***' 0.001 '**' 0.01 '*' 0.05 '.' 0.1 ' ' 1

Approximate significance of smooth terms:
                                            edf         Ref.df    Chi.sq
s(ID_BG_trap.fac)                   1.56212008428 2.00000000000  8.31918
s(yday)                             3.31900108223 4.11819782918 10.87082
s(yday):zona.SIT.discrete.ordbuffer      1.00004631471 1.00008309872  0.18009
s(yday):zona.SIT.discrete.ordoutside SIT 2.32391723459 2.76683876869  1.60120
                                      p-value
s(ID_BG_trap.fac)                   0.0053692 **
s(yday)                             0.0309787 *
s(yday):zona.SIT.discrete.ordbuffer 0.6713421
```

```
s(yday):zona.SIT.discrete.ordoutside SIT 0.7057884
---
Signif. codes:  0 '***' 0.001 '**' 0.01 '*' 0.05 '.' 0.1 ' ' 1

R-sq.(adj) =  0.722   Deviance explained = 72.9%
-REML =  42.44  Scale est. = 1         n = 45
```

```
summary(gamm.tot.females.Losone_one.smooth)
```

```
Family: Negative Binomial(7728.626)
Link function: log

Formula:
Ae.albopictus.female ~ s(ID_BG_trap.fac, bs = "re") + s(yday,
    k = 9) + zona.SIT.discrete.fac

Parametric coefficients:
                                          Estimate        Std. Error  z value
(Intercept)                        -1.48505326695399  0.76391954268267 -1.94399
zona.SIT.discrete.facbuffer         1.52625908046192  0.98912319998697  1.54304
zona.SIT.discrete.facoutside SIT -0.00642377858389  1.28608299557842 -0.00499
                                 Pr(>|z|)
(Intercept)                      0.051896 .
zona.SIT.discrete.facbuffer      0.122820
zona.SIT.discrete.facoutside SIT 0.996015
---
Signif. codes:  0 '***' 0.001 '**' 0.01 '*' 0.05 '.' 0.1 ' ' 1

Approximate significance of smooth terms:
                          edf       Ref.df   Chi.sq    p-value
s(ID_BG_trap.fac) 1.57012903330 2.0000000000  8.67927 0.00442194 **
s(yday)           3.49905057681 4.3344531263 23.82115 0.00014887 ***
---
Signif. codes:  0 '***' 0.001 '**' 0.01 '*' 0.05 '.' 0.1 ' ' 1

R-sq.(adj) =  0.746   Deviance explained = 68.7%
-REML =  45.76  Scale est. = 1         n = 45
```

We check whether the two models are nested. If they are, then we can test whether the most complex one is necessary, i.e., if we have evidence indicating that the more complex model improves the more simple model.

```
## Extract model matrices
m.gamm.tot.females.Losone.ord <- model.matrix(gamm.tot.females.Losone.ord)
m.gamm.tot.females.Losone_one.smooth <- model.matrix(gamm.tot.females.Losone_one.smooth)
dim(m.gamm.tot.females.Losone.ord)
```

```
[1] 45 32
```

```
dim(m.gamm.tot.females.Losone_one.smooth)
```

```
[1] 45 16
```

```
##
n1 <- ncol(m.gamm.tot.females.Losone_one.smooth)
## check that sum is zero
sum(matrix( m.gamm.tot.females.Losone_one.smooth[, 1:n1] - m.gamm.tot.females.Losone.ord[, 1:n1] ) != 0)
```

```
[1] 0
```

The two model matrices are nested, therefore we can now compare the two models with the Chi-square test.

```
chisq.test.Losone.slope <- anova.gam(gamm.tot.females.Losone_one.smooth,
                                     gamm.tot.females.Losone.ord,
                                     test = "Chisq") %>%
  print()
```

```
Analysis of Deviance Table

Model 1: Ae.albopictus.female ~ s(ID_BG_trap.fac, bs = "re") + s(yday,
    k = 9) + zona.SIT.discrete.fac
Model 2: Ae.albopictus.female ~ s(ID_BG_trap.fac, bs = "re") + s(yday,
    k = 9) + zona.SIT.discrete.fac + s(yday, by = zona.SIT.discrete.ord,
    k = 9)
       Resid. Df      Resid. Dev               Df      Deviance Pr(>Chi)
1 34.64238648263 76.68752019330
2 30.68411219558 72.81520728338 3.958274287055 3.87231290992  0.41733
```

We have no evidence that the more complex model improves the simpler one. This means that the additional flexibility, allowing different shapes for the smoother in each treatment zone, is not necessary. This is not surprising given the low sample size and the close-to-zero counts.

For good practice, we also look at AIC and BIC.

```
AIC(gamm.tot.females.Losone.ord, gamm.tot.females.Losone_one.smooth)
```

```
                                                df             AIC
gamm.tot.females.Losone.ord        13.7604862601121 100.336179803604
gamm.tot.females.Losone_one.smooth 10.2133965637380  97.114313320776
```

```
BIC(gamm.tot.females.Losone.ord, gamm.tot.females.Losone_one.smooth)
```

```
                                                df             BIC
gamm.tot.females.Losone.ord        13.7604862601121 125.196734170748
gamm.tot.females.Losone_one.smooth 10.2133965637380 115.566473785631
```

AIC and BIC agree that allowing different shapes for the smoother in each treatment zone is not necessary.

5.4.1.1 Shift We have found that allowing different shapes for the smoothers is not necessary. Therefore, we can keep the simpler model and test whether different intercepts for the treatment zones are necessary.

This can be performed by looking at the summary of the model with a different intercept for each treatment zone.

```
summary(gamm.tot.females.Losone_one.smooth)
```

```
Family: Negative Binomial(7728.626)
Link function: log

Formula:
Ae.albopictus.female ~ s(ID_BG_trap.fac, bs = "re") + s(yday,
    k = 9) + zona.SIT.discrete.fac

Parametric coefficients:
```

```
                                        Estimate        Std. Error  z value
(Intercept)                        -1.48505326695399  0.76391954268267 -1.94399
zona.SIT.discrete.facbuffer         1.52625908046192  0.98912319998697  1.54304
zona.SIT.discrete.facoutside SIT -0.00642377858389  1.28608299557842 -0.00499
                                   Pr(>|z|)
(Intercept)                        0.051896 .
zona.SIT.discrete.facbuffer        0.122820
zona.SIT.discrete.facoutside SIT 0.996015
---
Signif. codes:  0 '***' 0.001 '**' 0.01 '*' 0.05 '.' 0.1 ' ' 1

Approximate significance of smooth terms:
                            edf       Ref.df    Chi.sq    p-value
s(ID_BG_trap.fac) 1.57012903330 2.0000000000  8.67927 0.00442194 **
s(yday)           3.49905057681 4.3344531263 23.82115 0.00014887 ***
---
Signif. codes:  0 '***' 0.001 '**' 0.01 '*' 0.05 '.' 0.1 ' ' 1

R-sq.(adj) =  0.746   Deviance explained = 68.7%
-REML =  45.76  Scale est. = 1         n = 45
```

The difference between the buffer and outside zones relative to the treated zone is not significant. We now formally test whether including this variable as a fixed effect in the model is necessary by using the `anova.gam` function , which performs type III Anova test (rather then sequential type I Anova).

```r
test.Losone.shift <- anova.gam(gamm.tot.females.Losone_one.smooth) %>%
  print()
```

```
Family: Negative Binomial(7728.626)
Link function: log

Formula:
Ae.albopictus.female ~ s(ID_BG_trap.fac, bs = "re") + s(yday,
    k = 9) + zona.SIT.discrete.fac

Parametric Terms:
                      df  Chi.sq p-value
zona.SIT.discrete.fac  2 2.95475 0.22824

Approximate significance of smooth terms:
                            edf        Ref.df    Chi.sq    p-value
s(ID_BG_trap.fac) 1.57012903330 2.00000000000  8.67927 0.00442194
s(yday)           3.49905057681 4.33445312630 23.82115 0.00014887
```

Removing the variable *zona.SIT.discrete.fac* from the model does not significantly affect the model.

Note that we will keep both the fixed-effect and the interaction in the smooth terms for the variable *zona.SIT.discrete.fac* in the model, as it is a design variable.

5.4.2 Ascona

We now check the model for Ascona.

```r
## (this chunk is not evaluated)
gamm.tot.females.Ascona.ord <- gamV(Ae.albopictus.female ~
```

```r
                                      ## Ranef
                                      s(ID_BG_trap.fac, bs = "re") +

                                      ## smooth term
                                      s(yday, k = 9) +

                                      ## Fixed effect
                                      zona.SIT.discrete.fac +


                                      ## smooth term with interaction
                                      s(yday, by = zona.SIT.discrete.ord, k = 9),
                                    family = "nb",
                                    method = "GCV.Cp", ## as gam function
                                    data = d.adults.Ascona)
##
gamm.tot.females.Ascona_one.smooth <- gamV(Ae.albopictus.female ~

                                             ## Ranef
                                             s(ID_BG_trap.fac, bs = "re") +

                                             ## smooth term
                                             s(yday, k = 9) +

                                             ## Fixed effect
                                             zona.SIT.discrete.fac, # +

                                           ## smooth term with interaction
                                           # s(yday, by = zona.SIT.discrete.ord, k = 9),
                                           family = "nb",
                                           method = "GCV.Cp", ## as gam function
                                           data = d.adults.Ascona)
```

```r
summary(gamm.tot.females.Ascona.ord)
```

```

Family: Negative Binomial(3.01)
Link function: log

Formula:
Ae.albopictus.female ~ s(ID_BG_trap.fac, bs = "re") + s(yday,
    k = 9) + zona.SIT.discrete.fac + s(yday, by = zona.SIT.discrete.ord,
    k = 9)

Parametric coefficients:
                                   Estimate      Std. Error  z value Pr(>|z|)
(Intercept)                 -0.612838234858  0.627916543620 -0.97599  0.32907
zona.SIT.discrete.facbuffer  0.129901201906  0.789018061561  0.16464  0.86923

Approximate significance of smooth terms:
                                           edf        Ref.df   Chi.sq
s(ID_BG_trap.fac)                  2.13794175635 3.00000000000  6.08948
s(yday)                            4.12219293161 5.10205337314 10.97789
s(yday):zona.SIT.discrete.ordbuffer 1.00008832730 1.00016895149  2.09766
```

```
                                    p-value
s(ID_BG_trap.fac)                  0.036089 *
s(yday)                            0.052642 .
s(yday):zona.SIT.discrete.ordbuffer 0.147549
---
Signif. codes:  0 '***' 0.001 '**' 0.01 '*' 0.05 '.' 0.1 ' ' 1

R-sq.(adj) =  0.345   Deviance explained = 43.1%
-REML = 54.687  Scale est. = 1         n = 45
```

```r
summary(gamm.tot.females.Ascona_one.smooth)
```

```

Family: Negative Binomial(2.73)
Link function: log

Formula:
Ae.albopictus.female ~ s(ID_BG_trap.fac, bs = "re") + s(yday,
    k = 9) + zona.SIT.discrete.fac

Parametric coefficients:
                                    Estimate       Std. Error  z value Pr(>|z|)
(Intercept)                 -0.5173240922761  0.6104744182307 -0.84741  0.39676
zona.SIT.discrete.facbuffer  0.0900677379769  0.7744221031590  0.11630  0.90741

Approximate significance of smooth terms:
                            edf        Ref.df  Chi.sq  p-value
s(ID_BG_trap.fac) 2.11388537897 3.00000000000 5.99948 0.036590 *
s(yday)           3.94955256956 4.89503384209 9.74051 0.099633 .
---
Signif. codes:  0 '***' 0.001 '**' 0.01 '*' 0.05 '.' 0.1 ' ' 1

R-sq.(adj) =  0.307   Deviance explained =   38%
-REML = 56.139  Scale est. = 1         n = 45
```

We check whether the two models are nested. If they are, then we can test whether the most complex one is necessary, i.e., if we have evidence indicating that the more complex model improves the more simple model.

```r
## Extract model matrices
m.gamm.tot.females.Ascona.ord <- model.matrix(gamm.tot.females.Ascona.ord)
m.gamm.tot.females.Ascona_one.smooth <- model.matrix(gamm.tot.females.Ascona_one.smooth)
dim(m.gamm.tot.females.Ascona.ord)
```

```
[1] 45 23
```

```r
dim(m.gamm.tot.females.Ascona_one.smooth)
```

```
[1] 45 15
```

```r
##
n1 <- ncol(m.gamm.tot.females.Ascona_one.smooth)
## check that sum is zero
sum(matrix( m.gamm.tot.females.Ascona_one.smooth[, 1:n1] - m.gamm.tot.females.Ascona.ord[, 1:n1] ) != 0
```

```
[1] 0
```

The two model matrices are nested, therefore we can now compare the two models with the Chi-square test.

```r
chisq.test.Ascona.slope <- anova.gam(gamm.tot.females.Ascona_one.smooth,
                                     gamm.tot.females.Ascona.ord,
                                     test = "Chisq") %>%
  print()
```

```
Analysis of Deviance Table

Model 1: Ae.albopictus.female ~ s(ID_BG_trap.fac, bs = "re") + s(yday,
    k = 9) + zona.SIT.discrete.fac
Model 2: Ae.albopictus.female ~ s(ID_BG_trap.fac, bs = "re") + s(yday,
    k = 9) + zona.SIT.discrete.fac + s(yday, by = zona.SIT.discrete.ord,
    k = 9)
       Resid. Df      Resid. Dev              Df        Deviance Pr(>Chi)
1 33.80285881012 94.96905888586
2 32.55750315369 91.87017221060 1.245355656438 3.098886675258  0.10683
```

We have no evidence that the more complex model improves the simpler one. This means that the additional flexibility, allowing different shapes for the smoother in each treatment zone, is not necessary.

For good practice, we also look at AIC and BIC.

```r
AIC(gamm.tot.females.Ascona.ord, gamm.tot.females.Ascona_one.smooth)
```

```
                                                df             AIC
gamm.tot.females.Ascona.ord        11.8513599307861 115.572892072170
gamm.tot.females.Ascona_one.smooth 10.6302895692001 116.229638024256
```

```r
BIC(gamm.tot.females.Ascona.ord, gamm.tot.females.Ascona_one.smooth)
```

```
                                                df             BIC
gamm.tot.females.Ascona.ord        11.8513599307861 136.984299511888
gamm.tot.females.Ascona_one.smooth 10.6302895692001 135.434983444326
```

AIC and BIC disagree on the need for different smoother shapes across treatment zones. For this reason, we follow the result of the ANOVA test.

5.4.2.1 Shift We have found that allowing different shapes for the smoothers is not necessary. Therefore, we can keep the simpler model and test whether different intercepts for the treatment zones are necessary.

This can be performed by looking at the summary of the model with a different intercept for each treatment zone.

```r
test.Ascona.shift <- summary(gamm.tot.females.Ascona_one.smooth) %>%
  print()
```

```
Family: Negative Binomial(2.73)
Link function: log

Formula:
Ae.albopictus.female ~ s(ID_BG_trap.fac, bs = "re") + s(yday,
    k = 9) + zona.SIT.discrete.fac

Parametric coefficients:
                                    Estimate       Std. Error  z value Pr(>|z|)
(Intercept)                  -0.5173240922761   0.6104744182307 -0.84741   0.39676
```

```
zona.SIT.discrete.facbuffer  0.0900677379769  0.7744221031590  0.11630  0.90741

Approximate significance of smooth terms:
                            edf        Ref.df  Chi.sq  p-value
s(ID_BG_trap.fac) 2.11388537897 3.00000000000 5.99948 0.036590 *
s(yday)           3.94955256956 4.89503384209 9.74051 0.099633 .
---
Signif. codes:  0 '***' 0.001 '**' 0.01 '*' 0.05 '.' 0.1 ' ' 1

R-sq.(adj) =  0.307   Deviance explained =   38%
-REML = 56.139  Scale est. = 1         n = 45
```

The difference between the buffer zone relative to the treated zone is not statistically significant.

Note that we will keep both the fixed-effect and the interaction in the smooth terms for the variable *zona.SIT.discrete.fac* in the model, as it is a design variable.

5.5 Fitted values

First, we begin by plotting the fitted values over time, creating a separate graph for each municipality. Observations from the same BG trap will be connected by lines to visualise the trends and a vertical dashed line is included to show when the SIT releases started (i.e., 17.06.2025).

```r
## Losone
d.adults.Losone$fitted_gamm.hatching <-
  fitted(gamm.tot.females.Losone)
## Ascona
d.adults.Ascona$fitted_gamm.hatching <-
  fitted(gamm.tot.females.Ascona)
##
d.adults.fitted <- rbind(d.adults.Losone,
                         d.adults.Ascona)
##
ggplot(data = d.adults.fitted,
       mapping = aes(y = fitted_gamm.hatching,
                     x = yday,
                     group = ID_BG_trap.fac,
                     colour = zona.SIT.discrete.fac)) +
  geom_vline(xintercept = yday("2025-06-17"), linetype = "dashed", colour = "grey") +
  geom_hline(yintercept = 0) +
  geom_point(alpha = 0.1) +
  geom_line(alpha = 0.5) +
  scale_y_sqrt() +
  facet_grid(~ municipality.fac) +
  theme(legend.position = "bottom")
```

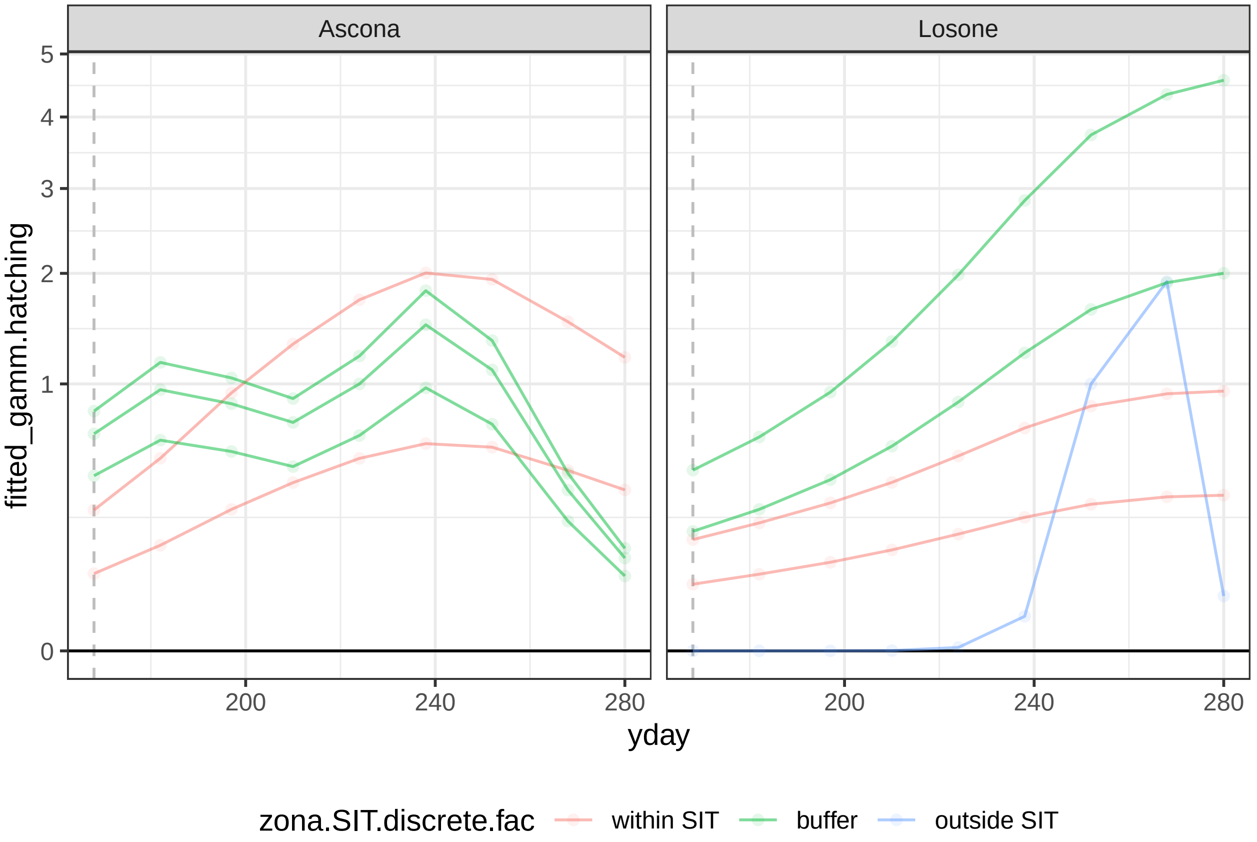

Note that there is significant variability in both municipalities, and we don't have many observations. However, we can observe that, in Losone, the treated zone has lower fitted values compared to the buffer zone, for all BG traps. For Ascona, this is not true.

5.6 Predicted values

We will now create the dataset for prediction. Our goal is to predict the female count over the entire season, both at the BG trap and population levels.

```
## (messages are omitted from this chunk)
##
formula(gamm.tot.females.Losone)
```

```
Ae.albopictus.female ~ s(ID_BG_trap.fac, bs = "re") + zona.SIT.discrete.fac +
    s(yday, by = zona.SIT.discrete.fac, k = 9)
```

```
##
## 1. We create a data set with ID_BG_trap.fac and zona.SIT.discrete.fac.
## We do it separately because not in all zona.SIT.discrete.fac all BG traps are present.
## We keep municipality to then separate the data set based on this variable.
d.muni.adults.short <- d.adults %>%
  group_by(municipality.fac, zona.SIT.discrete.fac) %>%
  mutate(yday.min = min(yday, na.rm = TRUE),
         yday.max = max(yday, na.rm = TRUE)) %>%
  select(municipality.fac, ID_BG_trap.fac, zona.SIT.discrete.fac,
         yday.min, yday.max) %>%
  unique()
```

```
##
head(d.muni.adults.short)
```

```
# A tibble: 6 x 5
# Groups:   municipality.fac, zona.SIT.discrete.fac [3]
  municipality.fac ID_BG_trap.fac zona.SIT.discrete.fac yday.min yday.max
  <fct>            <fct>          <fct>                    <dbl>    <dbl>
1 Ascona           1              within SIT                 168      280
2 Ascona           2              within SIT                 168      280
3 Ascona           3              buffer                     168      280
4 Ascona           4              buffer                     168      280
5 Ascona           5              buffer                     168      280
6 Losone           6              within SIT                 168      280
```

```
nrow(d.muni.adults.short)
```

```
[1] 10
```

```
##
## 2. We create a data set with varying yday for each BG trap ID
d.pred.gamm <- expand.grid(
  yday = seq(from = min(d.adults$yday),
             to = max(d.adults$yday),
             length.out = 100),
  ID_BG_trap.fac = d.muni.adults.short$ID_BG_trap.fac)
##
## 3. We join the two data sets.
## Note that not all BG traps exist in all municipalities.
## So, we can't simply use expand.grid()
d.pred.gamm_aug <- left_join(d.pred.gamm, d.muni.adults.short) %>%
  ## we don't want to predict outside the range of available data for each
  ## municipality, thus we filter the data set based on the min and max yday
  ## available for each municipality.
  filter(yday >= yday.min & yday <= yday.max)
str(d.pred.gamm_aug)
```

```
'data.frame':   1000 obs. of  6 variables:
 $ yday                 : num  168 169 170 171 173 ...
 $ ID_BG_trap.fac       : Factor w/ 10 levels "1","10","2","3",..: 1 1 1 1 1 1 1 1 1 1 ...
 $ municipality.fac     : Factor w/ 2 levels "Ascona","Losone": 1 1 1 1 1 1 1 1 1 1 ...
 $ zona.SIT.discrete.fac: Factor w/ 3 levels "within SIT","buffer",..: 1 1 1 1 1 1 1 1 1 1 ...
 $ yday.min             : num  168 168 168 168 168 168 168 168 168 168 ...
 $ yday.max             : num  280 280 280 280 280 280 280 280 280 280 ...
 - attr(*, "out.attrs")=List of 2
  ..$ dim     : Named int [1:2] 100 10
  .. ..- attr(*, "names")= chr [1:2] "yday" "ID_BG_trap.fac"
  ..$ dimnames:List of 2
  .. ..$ yday          : chr [1:100] "yday=168.000000000000" "yday=169.131313131313" "yday=170.26262626
  .. ..$ ID_BG_trap.fac: chr [1:10] "ID_BG_trap.fac=1" "ID_BG_trap.fac=2" "ID_BG_trap.fac=3" "ID_BG_trap
```

We make the predictions on the newly created data set, at BG trap and population level, and we plot the result.

```
d.pred.gamm_aug.Losone <- d.pred.gamm_aug %>%
  filter(municipality.fac == "Losone") %>%
  droplevels()
```

```r
## prediction at BG trap level
d.pred.gamm_aug.Losone$predicted_gamm.tot.females <- predict(
  gamm.tot.females.Losone,
  newdata = d.pred.gamm_aug.Losone,
  type = "response")
##
## prediction at population level
d.pred.gamm_aug.Losone$predicted_gamm.tot.females.pop <- predict(
  gamm.tot.females.Losone,
  newdata = d.pred.gamm_aug.Losone,
  type = "response",
  exclude = 's(ID_BG_trap.fac)')
##
## For Ascona
d.pred.gamm_aug.Ascona <- d.pred.gamm_aug %>%
  filter(municipality.fac == "Ascona") %>%
  droplevels()

## prediction at BG trap level
d.pred.gamm_aug.Ascona$predicted_gamm.tot.females <- predict(
  gamm.tot.females.Ascona,
  newdata = d.pred.gamm_aug.Ascona,
  type = "response")
##
## prediction at population level
d.pred.gamm_aug.Ascona$predicted_gamm.tot.females.pop <- predict(
  gamm.tot.females.Ascona,
  newdata = d.pred.gamm_aug.Ascona,
  type = "response",
  exclude = 's(ID_BG_trap.fac)')
##
## Join the two data sets
d.pred.gamm_aug <- rbind(d.pred.gamm_aug.Losone,
                         d.pred.gamm_aug.Ascona)
```

```r
gg.prediction.gamm.Losone <- ggplot(data = d.pred.gamm_aug.Losone,
                                    mapping = aes(y = predicted_gamm.tot.females,
                                                  x = yday,
                                                  group = ID_BG_trap.fac)) +
  geom_vline(xintercept = yday("2025-06-17"), linetype = "dashed", colour = "grey") +
  geom_hline(yintercept = c(0), colour = "grey") +
  geom_line(alpha = 0.5) +
  geom_line(mapping = aes(y = predicted_gamm.tot.females.pop),
            colour = "red") +
  facet_wrap(~ zona.SIT.discrete.fac) +
  geom_rug(data = d.adults.Losone, mapping = aes(x = yday),
           sides = "b",
           inherit.aes = FALSE,
           alpha = 0.5)
gg.prediction.gamm.Losone +
  labs(title = "Losone")
```

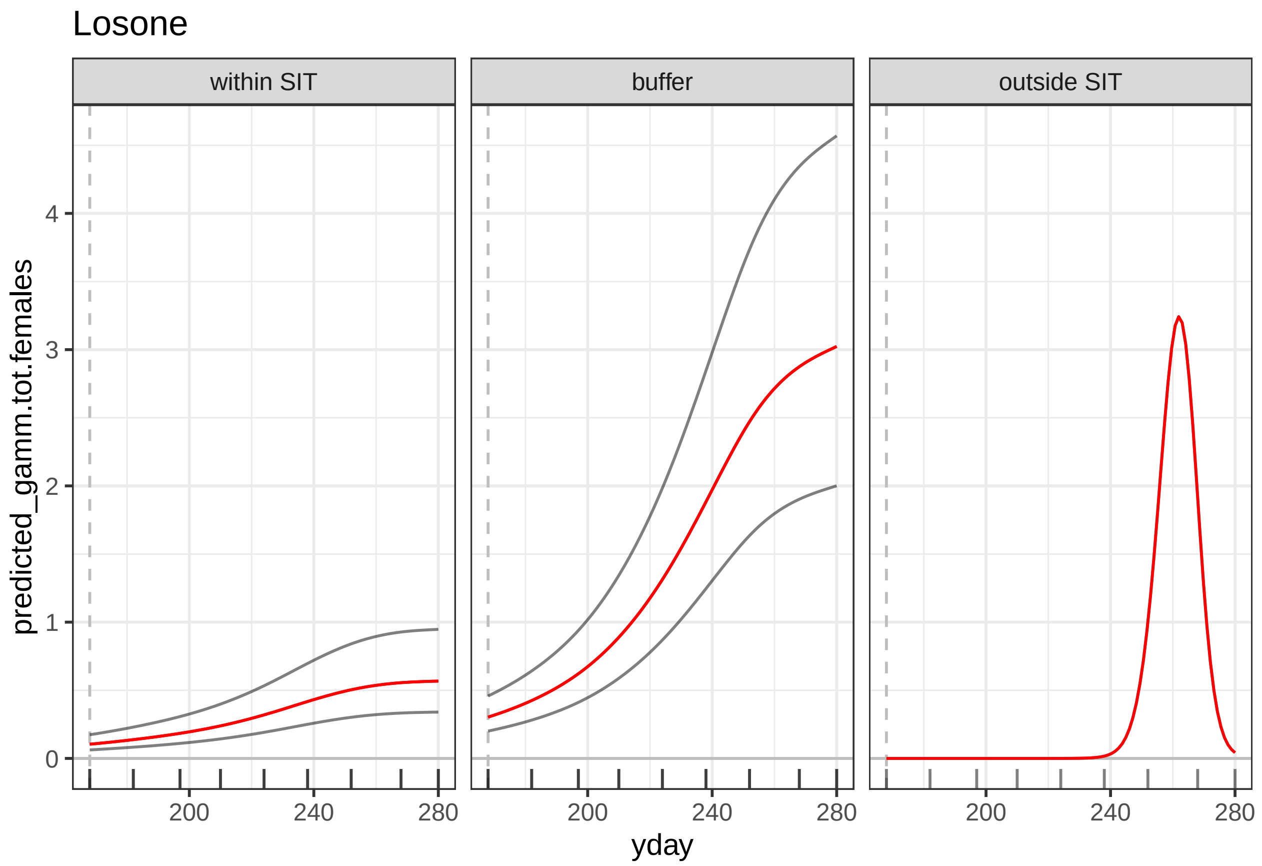


```r
gg.prediction.gamm.Ascona <- ggplot(data = d.pred.gamm_aug.Ascona,
                                    mapping = aes(y = predicted_gamm.tot.females,
                                                  x = yday,
                                                  group = ID_BG_trap.fac)) +
  geom_vline(xintercept = yday("2025-06-17"), linetype = "dashed", colour = "grey") +
  geom_hline(yintercept = c(0), colour = "grey") +
  geom_line(alpha = 0.5) +
  geom_line(mapping = aes(y = predicted_gamm.tot.females.pop),
            colour = "red") +
  facet_wrap(~ zona.SIT.discrete.fac) +
  geom_rug(data = d.adults.Ascona, mapping = aes(x = yday),
           sides = "b",
           inherit.aes = FALSE,
           alpha = 0.5)
gg.prediction.gamm.Ascona +
  labs(title = "Ascona")
```

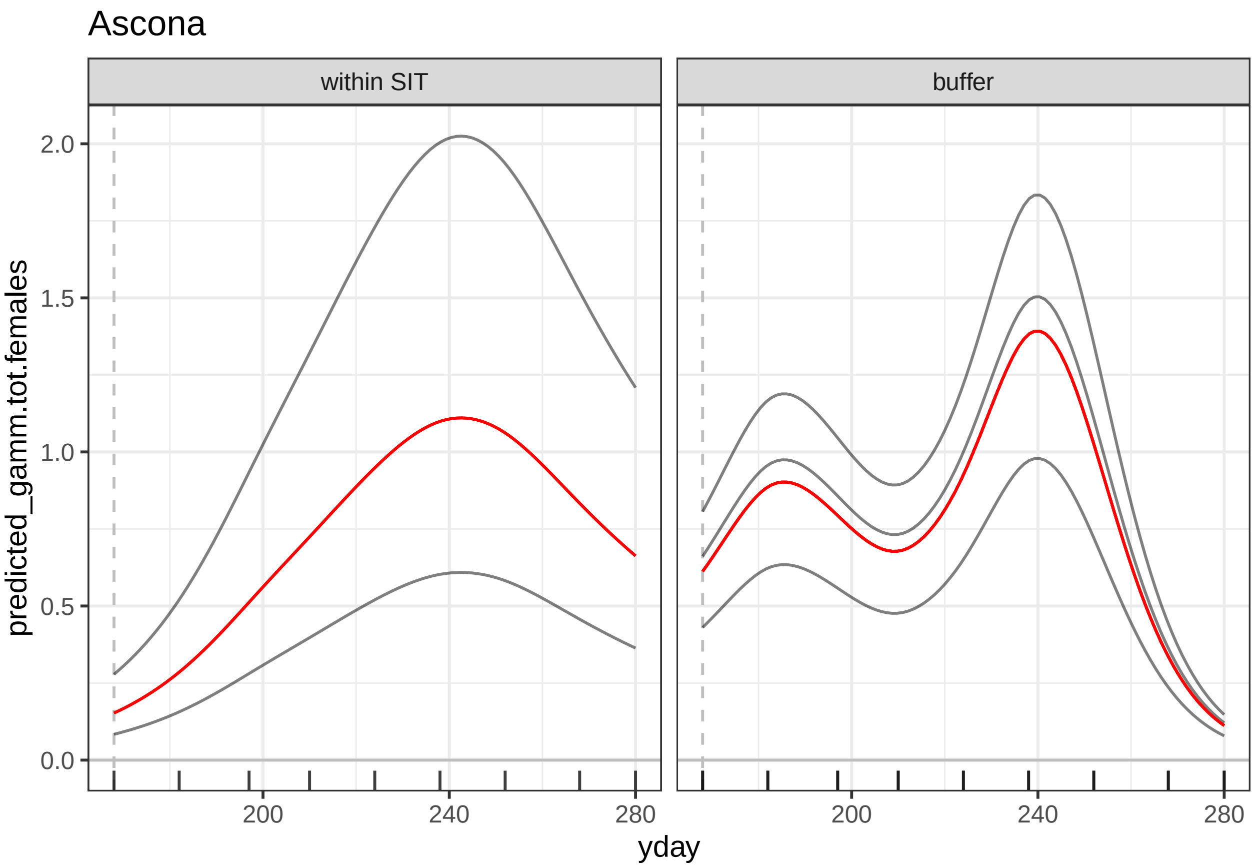

Each black line corresponds to the prediction for a given BG trap, whereas the red line corresponds to the prediction at population level.

The number of different BG traps in each zone is very limited, thus the predictions are not be very reliable.

We plot the predictions at population level on the same plot.

```
gg.prediction.gamm.together <- ggplot(data = d.pred.gamm_aug,
                                      mapping = aes(y = predicted_gamm.tot.females.pop,
                                                    x = yday,
                                                    colour = zona.SIT.discrete.fac)) +
  geom_vline(xintercept = yday(as.Date("2025-06-17")),
             linetype = "dashed", colour = "grey") +
  geom_line() +
  geom_hline(yintercept = c(0), colour = "grey") +
  facet_wrap(~ municipality.fac) +
  labs(colour = "Zone") +
  theme(legend.position = "bottom")
gg.prediction.gamm.together
```

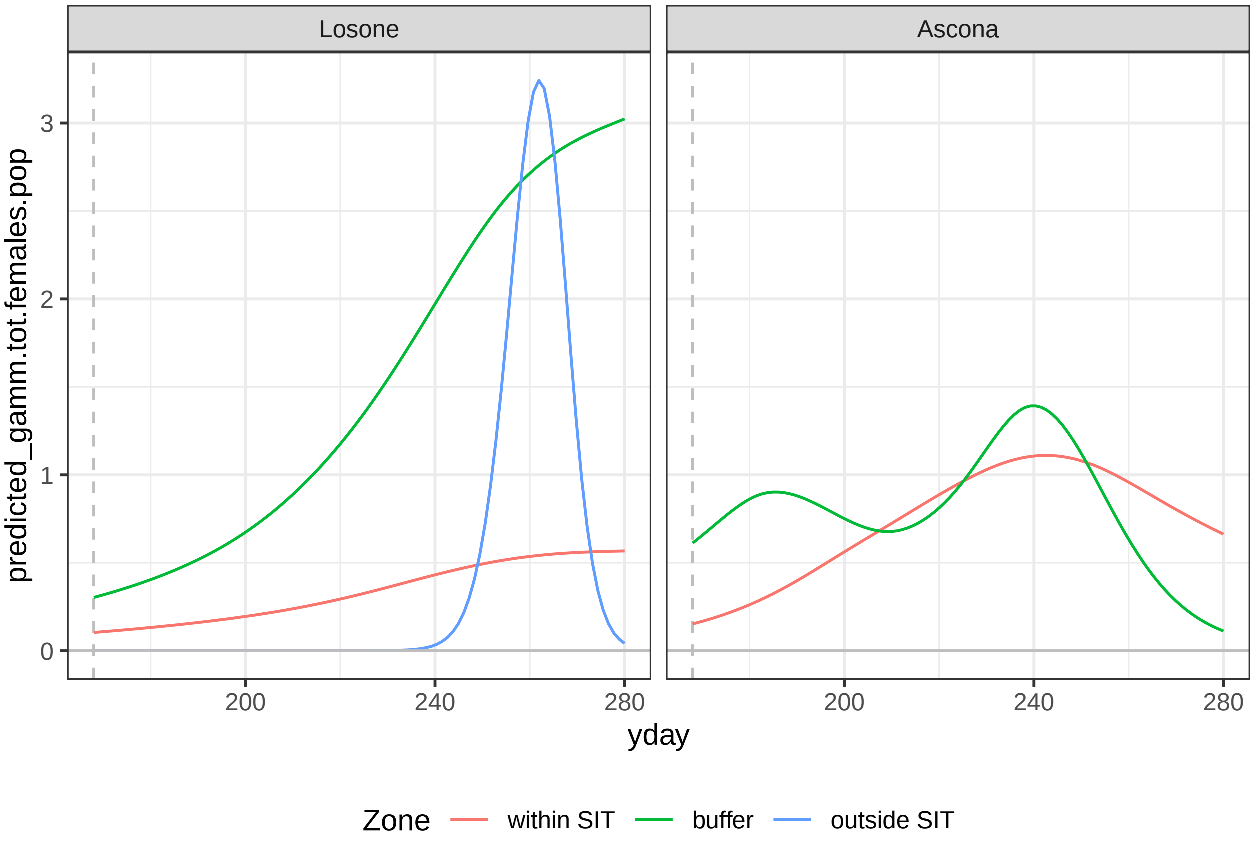

In Losone, the treated zone has consistently predicted lower female counts than the buffer area. However, we see that in the untreated zone, the predicted female counts is zero until day 240 and then increases dramatically, surpassing the treated zone. In Ascona, there is not a predicted curve consistently higher than the other. The untreated zone is not present.

Note that we did not plot the confidence bands here because we already have three curves overlaid. Adding the confidence bands would make the plot too cluttered and difficult to read. Instead, we will focus on plotting the differences between zones in the next section, along with the confidence bands, which will provide a clearer picture of the treatment effects.

5.6.1 Plotting the differences

We now plot the predicted differences between female counts in the treatment zones throughout the season, separately for each municipality.

We are no longer interested in the prediction at BG trap level, but only at population level. Thus, we create a new data set containing only the necessary information for this purpose.

```
## We want to predict at population level, thus we don't care about the random
## effects. This means that we just need one observation per group.
d.pred.gamm_aug_pop <- d.pred.gamm_aug %>%
  group_by(yday, municipality.fac, zona.SIT.discrete.fac) %>%
  slice(1) %>%
  ungroup()
##
## We need to start the comparison where all zones have available data.
d.pred.gamm_aug_pop <- d.pred.gamm_aug_pop %>%
```

```
  filter(yday >= max(yday.min) & yday <= min(yday.max)) %>%
  select(-yday.min, -yday.max)
```

5.6.1.1 Buffer versus within SIT zone – Losone

We start by looking at the difference between the buffer and the treated zone in Losone.

We create the data set on which we are going to calculate the contrasts. We make sure that we first have all the observations belonging to the "within SIT" level, followed by those of the "buffer" level.

```
d.pred.gamm_aug.Losone.25.SIT.buffer <- d.pred.gamm_aug_pop %>%
  filter(municipality.fac == "Losone") %>%
  filter(zona.SIT.discrete.fac %in% c("within SIT", "buffer")) %>%
  droplevels() %>%
  arrange(zona.SIT.discrete.fac, yday)
##
## check
d.pred.gamm_aug.Losone.25.SIT.buffer %>%
  pull(zona.SIT.discrete.fac)
```

```
  [1] within SIT within SIT within SIT within SIT within SIT within SIT
  [7] within SIT within SIT within SIT within SIT within SIT within SIT
 [13] within SIT within SIT within SIT within SIT within SIT within SIT
 [19] within SIT within SIT within SIT within SIT within SIT within SIT
 [25] within SIT within SIT within SIT within SIT within SIT within SIT
 [31] within SIT within SIT within SIT within SIT within SIT within SIT
 [37] within SIT within SIT within SIT within SIT within SIT within SIT
 [43] within SIT within SIT within SIT within SIT within SIT within SIT
 [49] within SIT within SIT within SIT within SIT within SIT within SIT
 [55] within SIT within SIT within SIT within SIT within SIT within SIT
 [61] within SIT within SIT within SIT within SIT within SIT within SIT
 [67] within SIT within SIT within SIT within SIT within SIT within SIT
 [73] within SIT within SIT within SIT within SIT within SIT within SIT
 [79] within SIT within SIT within SIT within SIT within SIT within SIT
 [85] within SIT within SIT within SIT within SIT within SIT within SIT
 [91] within SIT within SIT within SIT within SIT within SIT within SIT
 [97] within SIT within SIT within SIT within SIT buffer     buffer
[103] buffer     buffer     buffer     buffer     buffer     buffer
[109] buffer     buffer     buffer     buffer     buffer     buffer
[115] buffer     buffer     buffer     buffer     buffer     buffer
[121] buffer     buffer     buffer     buffer     buffer     buffer
[127] buffer     buffer     buffer     buffer     buffer     buffer
[133] buffer     buffer     buffer     buffer     buffer     buffer
[139] buffer     buffer     buffer     buffer     buffer     buffer
[145] buffer     buffer     buffer     buffer     buffer     buffer
[151] buffer     buffer     buffer     buffer     buffer     buffer
[157] buffer     buffer     buffer     buffer     buffer     buffer
[163] buffer     buffer     buffer     buffer     buffer     buffer
[169] buffer     buffer     buffer     buffer     buffer     buffer
[175] buffer     buffer     buffer     buffer     buffer     buffer
[181] buffer     buffer     buffer     buffer     buffer     buffer
[187] buffer     buffer     buffer     buffer     buffer     buffer
[193] buffer     buffer     buffer     buffer     buffer     buffer
[199] buffer     buffer
```

```
Levels: within SIT buffer
```

```r
## makes sense
```

Next, we create the model matrix for calculating the contrasts.

```r
## Model matrix
W.predictions.buffer.sit.Losone <- predict(gamm.tot.females.Losone,
                                           newdata = d.pred.gamm_aug.Losone.25.SIT.buffer,
                                           type = "lpmatrix",

                                           ## Prediction at population level
                                           exclude = 's(ID_BG_trap.fac)')
```

We also extract the coefficients of the model and the corresponding variance-covariance matrix.

```r
c.coeff.gamm.tot.females.Losone <- coef(gamm.tot.females.Losone)
vcov.c.coeff.gamm.tot.females.Losone <- vcov(gamm.tot.females.Losone)
```

We can now compute the contrasts.

```r
## 1) We count the number of rows of the matrix representing the "within SIT" level
n <- d.pred.gamm_aug.Losone.25.SIT.buffer %>%
  filter(zona.SIT.discrete.fac == "within SIT") %>%
  nrow() %>%
  print()
```

```
[1] 100
```

```r
##
## check that "buffer" matrix has the same number of rows
d.pred.gamm_aug.Losone.25.SIT.buffer %>%
  filter(zona.SIT.discrete.fac == "buffer") %>%
  nrow()
```

```
[1] 100
```

```r
## makes sense

## 2) We calculate the contrasts, exploiting the model matrix.

##  i) We calculate the contrast matrix L as (X_buffer - X_withinSIT)

## We made sure that we first have all the "within SIT" observations,
## and then all the "buffer" ones, thus we can do as follows:
L.buffer.Losone  <- W.predictions.buffer.sit.Losone[(n + 1):(2*n), , drop = FALSE] -
  W.predictions.buffer.sit.Losone[1:n, , drop = FALSE]

##  ii) multiply L by beta to calculate the difference between the predicted values
## => eta = L * beta
est.buffer.Losone <- (L.buffer.Losone %*% c.coeff.gamm.tot.females.Losone) %>%
  as.vector()
```

Now that we have computed the contrasts, we can calculate the corresponding standard error.

```r
## 3) We calculate the corresponding standard error, which is given by
## the variance of L*beta
## Var(L * beta) = diag( L %*% Var(beta) %*% t(L) )
## and then we take the sqrt for the standard error
```

```
## the following code computes it
se.buffer.Losone  <-
  ( (L.buffer.Losone %*% vcov.c.coeff.gamm.tot.females.Losone) * L.buffer.Losone ) %>%
  rowSums() %>%
  sqrt()
## equivalent to
# se.buffer.Losone.2 <-
# ( (L.buffer.Losone %*% vcov.c.coeff.gamm.tot.females.Losone) %*% t(L.buffer.Losone) ) %>%
#   diag() %>%
#   sqrt()
## but requires less computing power
```

We store everything in a data frame and plot the results.

```
d.diff.buffer.Losone <- data.frame(
  yday = d.pred.gamm_aug.Losone$yday[1:n],
  diff = est.buffer.Losone,
  lo = est.buffer.Losone - qnorm(0.975) * se.buffer.Losone,
  hi = est.buffer.Losone + qnorm(0.975) * se.buffer.Losone) %>%
  ##
  ## back transform the estimated values
  mutate(exp.diff = exp(diff),
         exp.lo = exp(lo),
         exp.hi = exp(hi))
```

```
ggplot(d.diff.buffer.Losone, mapping = aes(x = yday, y = diff)) +
  geom_ribbon(aes(ymin = lo, ymax = hi), fill = "lightgray", alpha = 0.5) +
  geom_line(linewidth = 1) +
  geom_hline(yintercept = 0, linetype = "dashed") +
  geom_vline(xintercept = yday("2025-06-17"), linetype = "dashed", colour = "grey") +
  labs(title = "Losone",
       y = "buffer minus treated zone") +
  coord_cartesian(ylim = c(-3, 3))
```

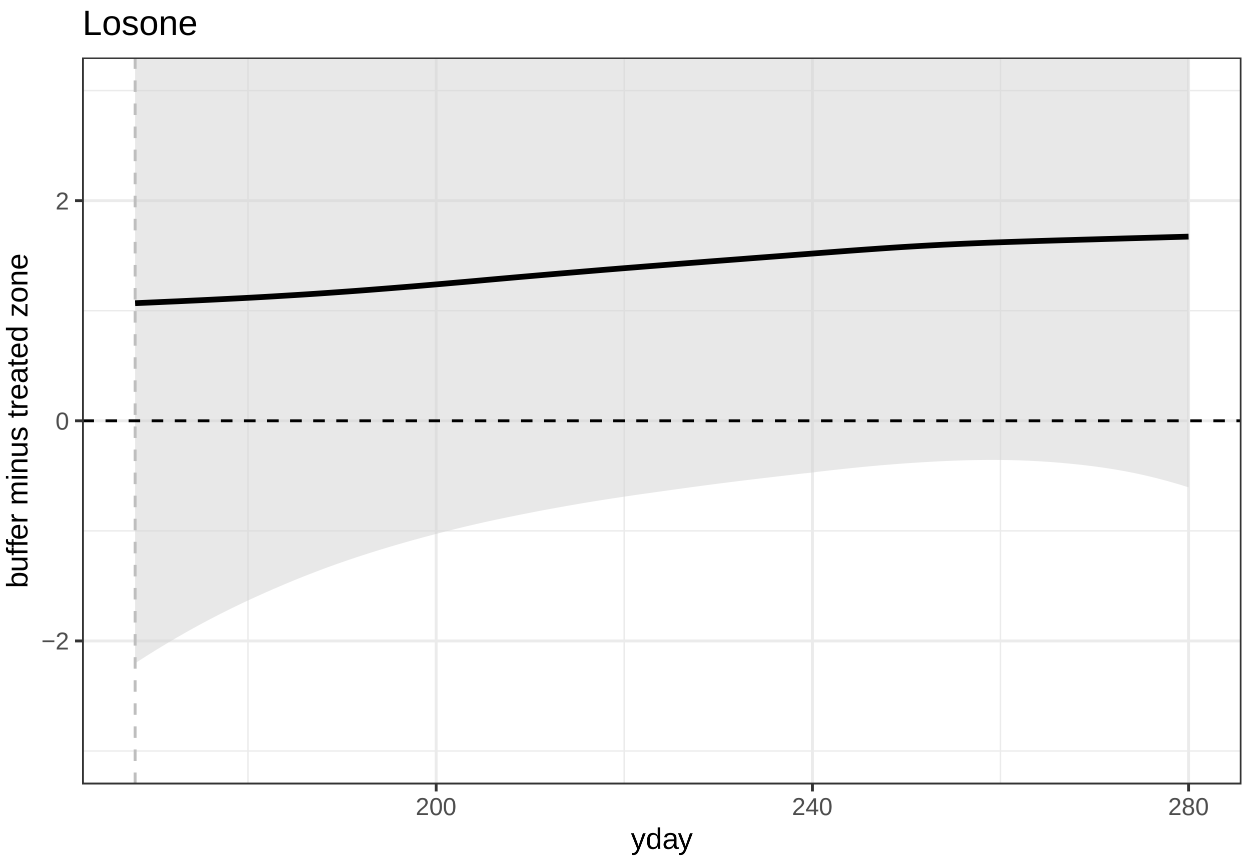

The plot shows the difference between the predicted values on the linear predictor scale for the buffer and treated zones, along with a 95% confidence interval. A horizontal dashed line at 0 indicates no difference between the two zones, while a vertical dashed line marks the start of the treatment on June 17, 2025.

We back-transform the difference by applying the exponential function to return it to the original scale. After this transformation, the values can be interpreted as ratios of predicted female counts: a value of 1 indicates no difference between zones, values greater than 1 indicate higher predicted female counts in the buffer zone relative to the treated zone, and values below 1 indicate the opposite.

```
## In the back transformed scale
gg.buffer.losone.exp <- ggplot(d.diff.buffer.Losone,
                               mapping = aes(x = yday, y = exp.diff)) +
  geom_ribbon(aes(ymin = exp.lo, ymax = exp.hi), fill = "lightgray", alpha = 0.5) +
  geom_line(linewidth = 1) +
  geom_hline(yintercept = 1, linetype = "dashed") +
  geom_vline(xintercept = yday("2025-06-17"), linetype = "dashed", colour = "grey") +
  labs(title = "Losone",
       y = "buffer minus treated zone \nin the back transformed scale")
gg.buffer.losone.exp
```

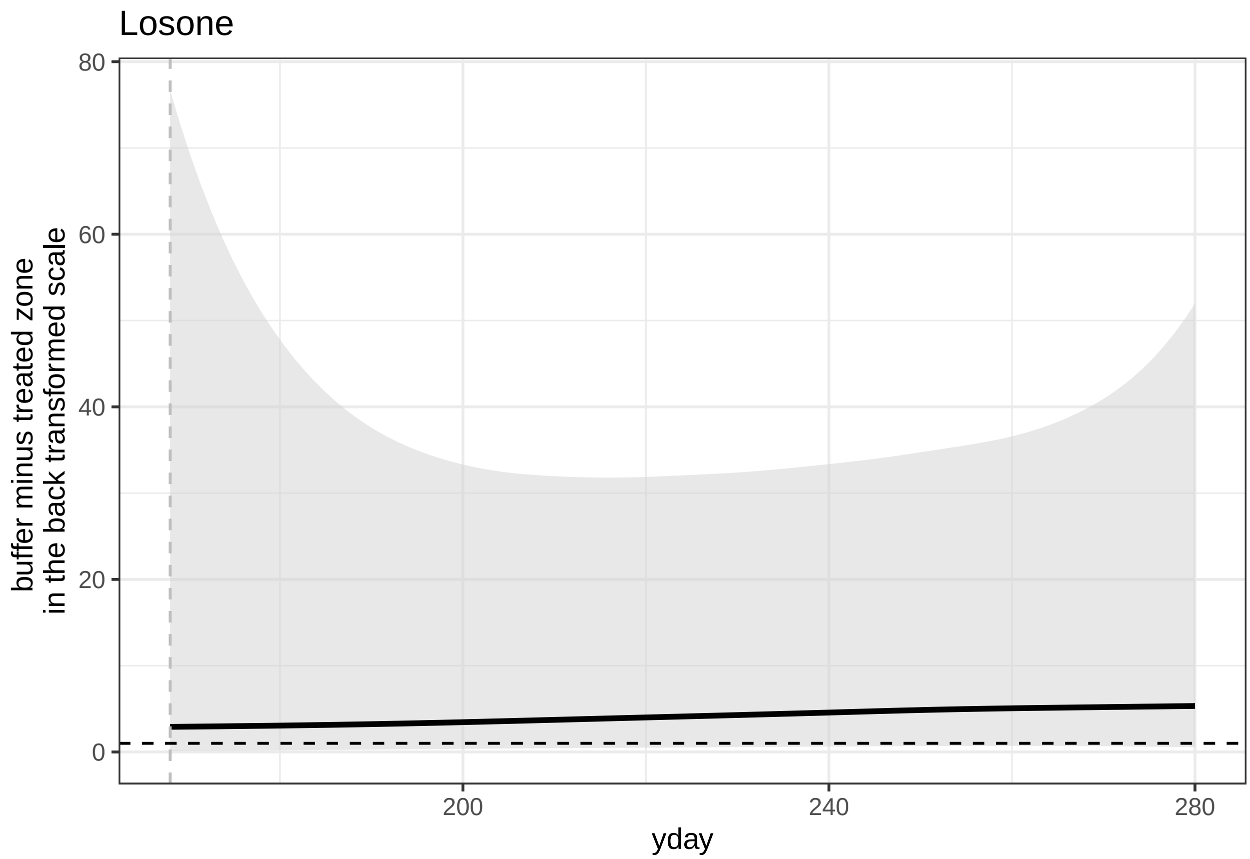

We zoom in to concentrate on the most relevant part of the graph.

```
## we zoom in
gg.buffer.losone.exp +
  coord_cartesian(ylim = c(0, 10))
```

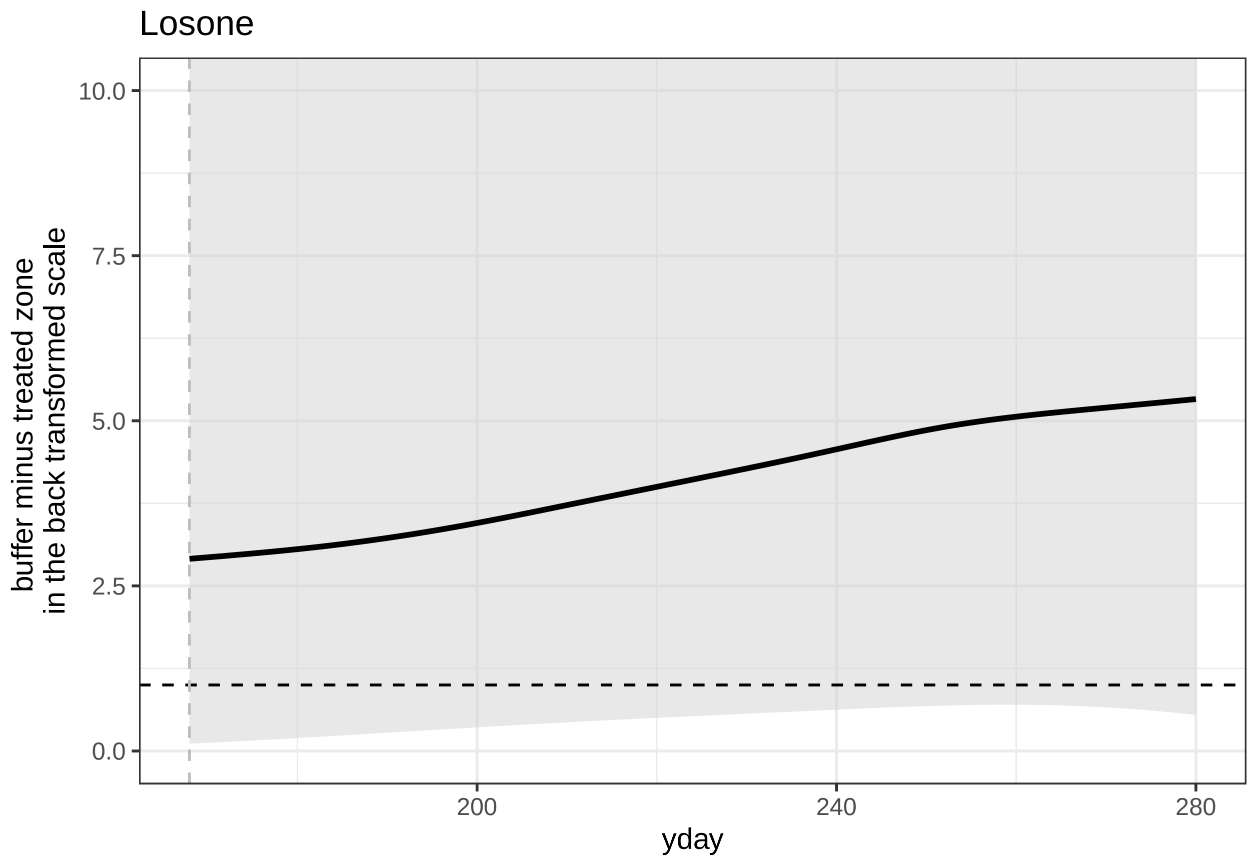

The buffer zone has predicted higher female counts compared to the treated zone. However, this difference is never statistically significant (based on the 95% confidence intervals) throughout the season.

5.6.1.2 Buffer versus within SIT zone – Ascona

We redo the same exact process, but for Ascona. For the full explanation, look at the section above.

```
d.pred.gamm_aug.Ascona.25.SIT.buffer <- d.pred.gamm_aug_pop %>%
  filter(municipality.fac == "Ascona") %>%
  filter(zona.SIT.discrete.fac %in% c("within SIT", "buffer")) %>%
  droplevels() %>%
  arrange(zona.SIT.discrete.fac, yday)
##
## check
d.pred.gamm_aug.Ascona.25.SIT.buffer %>%
  pull(zona.SIT.discrete.fac)
```

```
 [1] within SIT within SIT within SIT within SIT within SIT within SIT
 [7] within SIT within SIT within SIT within SIT within SIT within SIT
[13] within SIT within SIT within SIT within SIT within SIT within SIT
[19] within SIT within SIT within SIT within SIT within SIT within SIT
[25] within SIT within SIT within SIT within SIT within SIT within SIT
[31] within SIT within SIT within SIT within SIT within SIT within SIT
[37] within SIT within SIT within SIT within SIT within SIT within SIT
[43] within SIT within SIT within SIT within SIT within SIT within SIT
[49] within SIT within SIT within SIT within SIT within SIT within SIT
[55] within SIT within SIT within SIT within SIT within SIT within SIT
```

```
 [61] within SIT within SIT within SIT within SIT within SIT within SIT
 [67] within SIT within SIT within SIT within SIT within SIT within SIT
 [73] within SIT within SIT within SIT within SIT within SIT within SIT
 [79] within SIT within SIT within SIT within SIT within SIT within SIT
 [85] within SIT within SIT within SIT within SIT within SIT within SIT
 [91] within SIT within SIT within SIT within SIT within SIT within SIT
 [97] within SIT within SIT within SIT within SIT buffer     buffer
[103] buffer     buffer     buffer     buffer     buffer     buffer
[109] buffer     buffer     buffer     buffer     buffer     buffer
[115] buffer     buffer     buffer     buffer     buffer     buffer
[121] buffer     buffer     buffer     buffer     buffer     buffer
[127] buffer     buffer     buffer     buffer     buffer     buffer
[133] buffer     buffer     buffer     buffer     buffer     buffer
[139] buffer     buffer     buffer     buffer     buffer     buffer
[145] buffer     buffer     buffer     buffer     buffer     buffer
[151] buffer     buffer     buffer     buffer     buffer     buffer
[157] buffer     buffer     buffer     buffer     buffer     buffer
[163] buffer     buffer     buffer     buffer     buffer     buffer
[169] buffer     buffer     buffer     buffer     buffer     buffer
[175] buffer     buffer     buffer     buffer     buffer     buffer
[181] buffer     buffer     buffer     buffer     buffer     buffer
[187] buffer     buffer     buffer     buffer     buffer     buffer
[193] buffer     buffer     buffer     buffer     buffer     buffer
[199] buffer     buffer
Levels: within SIT buffer
```

```r
## makes sense
```

```r
## Model matrix
W.predictions.buffer.sit.Ascona <- predict(gamm.tot.females.Ascona,
                                           newdata = d.pred.gamm_aug.Ascona.25.SIT.buffer,
                                           type = "lpmatrix",

                                           ## Prediction at population level
                                           exclude = 's(ID_BG_trap.fac)')
##
c.coeff.gamm.tot.females.Ascona <- coef(gamm.tot.females.Ascona)
vcov.c.coeff.gamm.tot.females.Ascona <- vcov(gamm.tot.females.Ascona)


## Calculate the contrasts

## First, count the rows of the matrix containing only "within SIT"
n2 <- d.pred.gamm_aug.Ascona.25.SIT.buffer %>%
  filter(zona.SIT.discrete.fac == "within SIT") %>%
  nrow() %>%
  print()
```

```
[1] 100
```

```r
##
## check that "buffer" has the same dimension
d.pred.gamm_aug.Ascona.25.SIT.buffer %>%
  filter(zona.SIT.discrete.fac == "buffer") %>%
  nrow()
```

```
[1] 100
```

```r
## makes sense

## Calculate the contrasts
L.buffer.Ascona  <- W.predictions.buffer.sit.Ascona[(n2 + 1):(2*n2), , drop = FALSE] -
  W.predictions.buffer.sit.Ascona[1:n2, , drop = FALSE]
est.buffer.Ascona <- as.vector(L.buffer.Ascona %*% c.coeff.gamm.tot.females.Ascona)

## Now, we calculate the corresponding standard error
se.buffer.Ascona  <-
  ( (L.buffer.Ascona %*% vcov.c.coeff.gamm.tot.females.Ascona) * L.buffer.Ascona) %>%
  rowSums() %>%
  sqrt()

d.diff.buffer.Ascona <- data.frame(
  yday = d.pred.gamm_aug.Ascona$yday[1:n2],
  diff = est.buffer.Ascona,
  lo = est.buffer.Ascona - qnorm(0.975) * se.buffer.Ascona,
  hi = est.buffer.Ascona + qnorm(0.975) * se.buffer.Ascona
) %>%
  mutate(exp.diff = exp(diff),
         exp.lo = exp(lo),
         exp.hi = exp(hi))
```

We plot the result.

```r
ggplot(d.diff.buffer.Ascona, mapping = aes(x = yday, y = diff)) +
  geom_ribbon(aes(ymin = lo, ymax = hi), fill = "lightgray", alpha = 0.5) +
  geom_line(linewidth = 1) +
  geom_hline(yintercept = 0, linetype = "dashed") +
  geom_vline(xintercept = yday("2025-06-17"), linetype = "dashed", colour = "grey") +
  labs(title = "Ascona",
       y = "buffer minus treated zone")
```

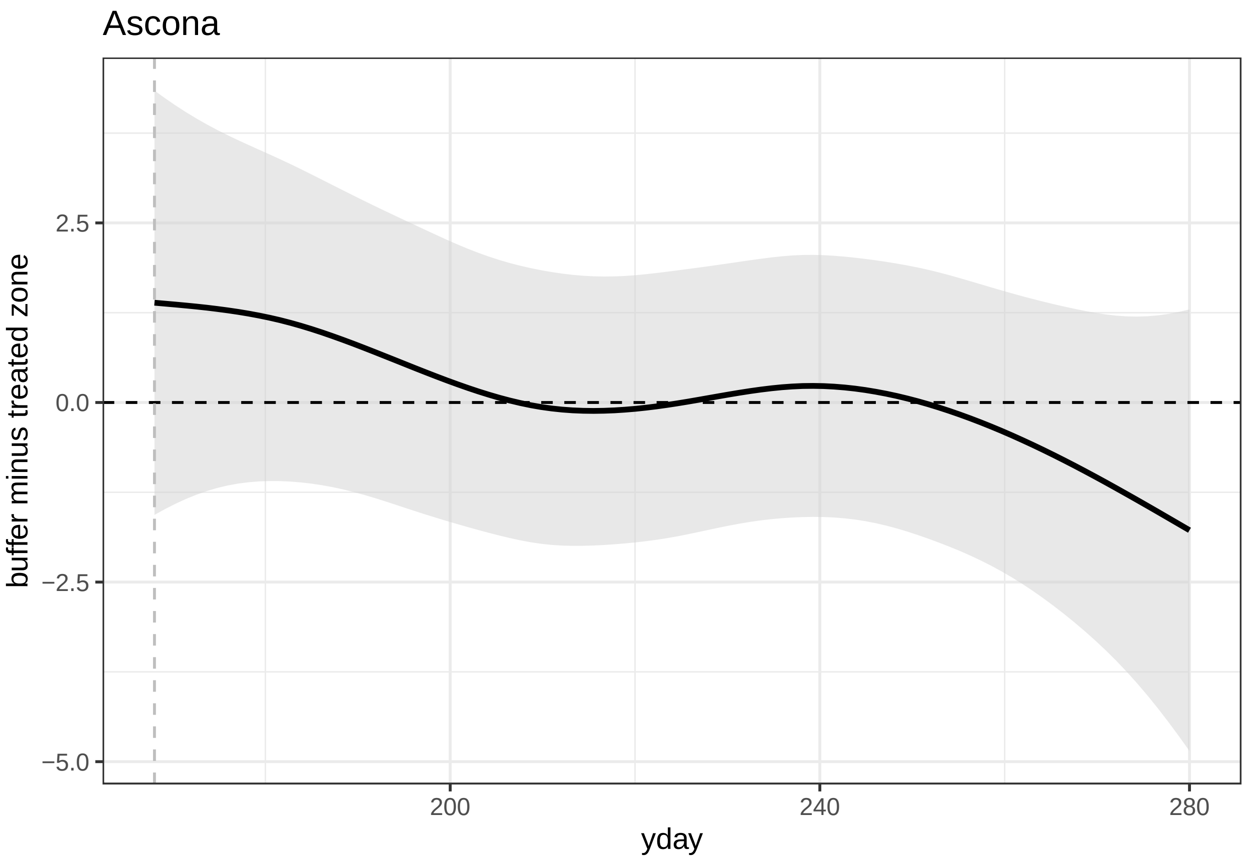

The plot shows the difference between the predicted values on the linear predictor scale for the buffer and treated zones, along with a 95% confidence interval. A horizontal dashed line at 0 indicates no difference between the two zones, while a vertical dashed line marks the start of the treatment on June 17, 2025.

We back-transform the difference by applying the exponential function to return it to the original scale. After this transformation, the values can be interpreted as ratios of predicted female counts: a value of 1 indicates no difference between zones, values greater than 1 indicate higher predicted female counts in the buffer zone relative to the treated zone, and values below 1 indicate the opposite.

```
## in the back transformed scale
gg.buffer.Ascona <- ggplot(d.diff.buffer.Ascona, mapping = aes(x = yday, y = exp.diff)) +
  geom_ribbon(aes(ymin = exp.lo, ymax = exp.hi), fill = "lightgray", alpha = 0.5) +
  geom_line(linewidth = 1) +
  geom_hline(yintercept = 1, linetype = "dashed") +
  geom_vline(xintercept = yday("2025-06-17"), linetype = "dashed", colour = "grey") +
  labs(title = "Ascona",
       y = "buffer minus treated zone \nin the back transformed scale")
gg.buffer.Ascona
```

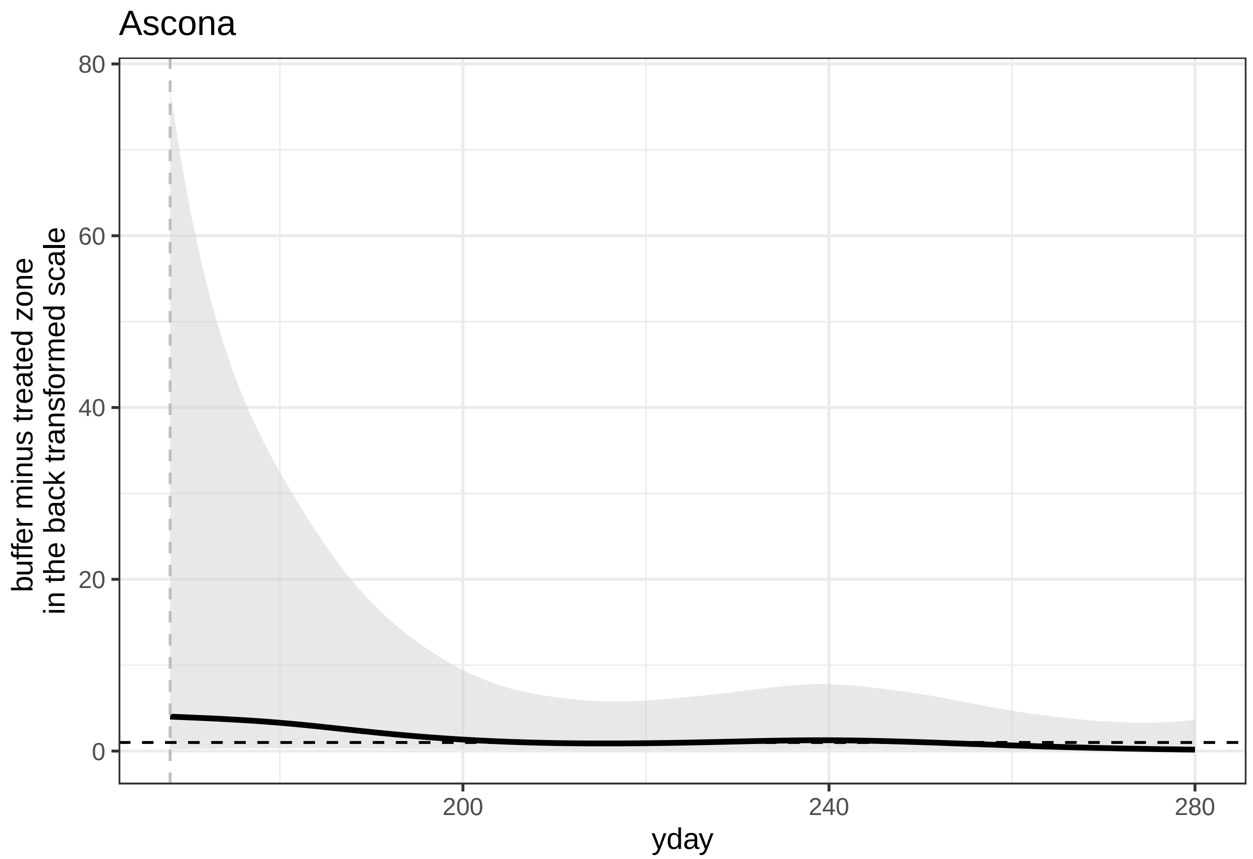

We zoom in to concentrate on the most relevant part of the graph. We use the same zoom as for Losone.

```
## we zoom in, using the same zoom as for Losone
gg.buffer.Ascona +
  coord_cartesian(ylim = c(0, 10))
```

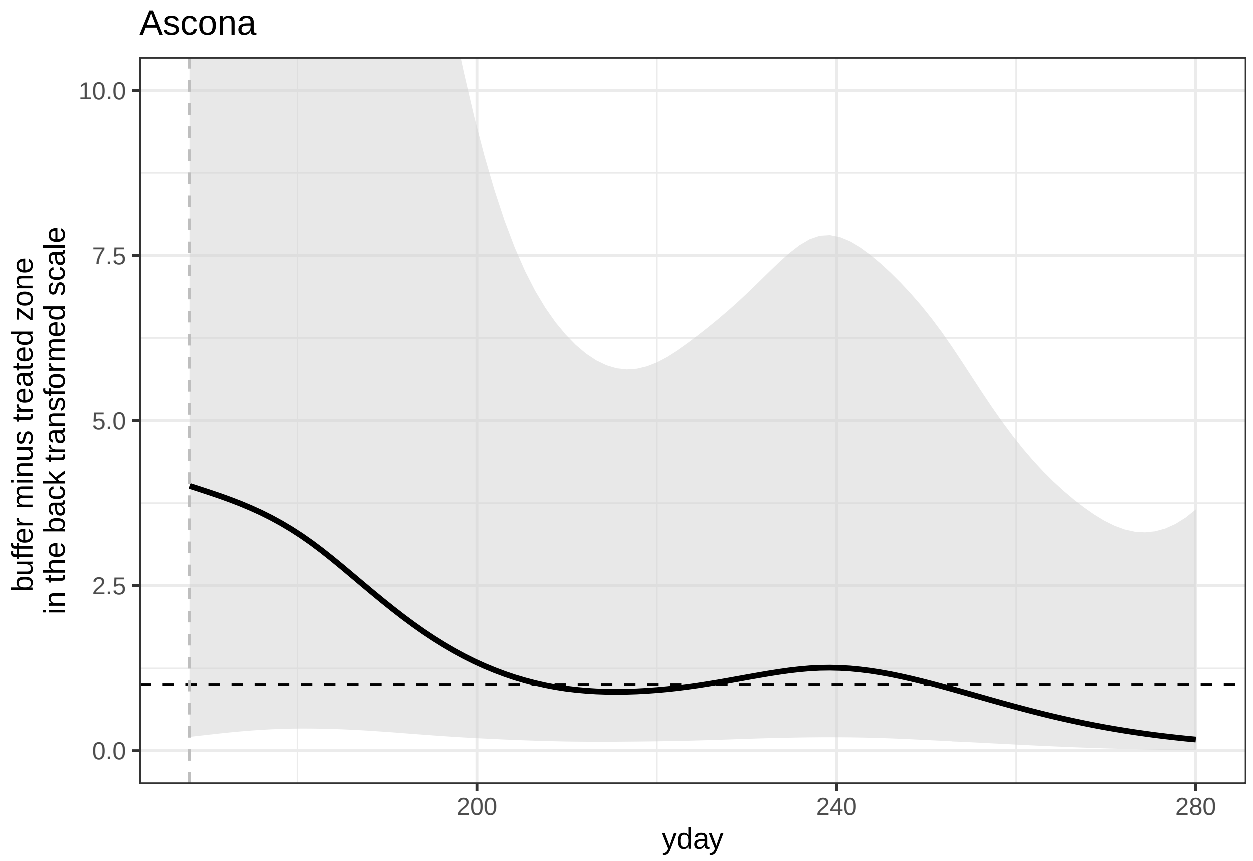

The buffer area initially has higher female counts than the treated zone; however, this difference reverses over time. Moreover, it is never statistically significant.

5.6.1.3 outside SIT versus within SIT zone – Losone We now perform the same steps as in the previous two sections, but replacing the buffer area with the untreated zone. We start with Losone.

```
d.pred.gamm_aug.Losone.25.SIT.outside <- d.pred.gamm_aug_pop %>%
  filter(municipality.fac == "Losone") %>%
  filter(zona.SIT.discrete.fac %in% c("within SIT", "outside SIT")) %>%
  droplevels() %>%
  arrange(zona.SIT.discrete.fac, yday)
##
## check
d.pred.gamm_aug.Losone.25.SIT.outside %>%
  pull(zona.SIT.discrete.fac)
```

```
 [1] within SIT  within SIT  within SIT  within SIT  within SIT  within SIT
 [7] within SIT  within SIT  within SIT  within SIT  within SIT  within SIT
[13] within SIT  within SIT  within SIT  within SIT  within SIT  within SIT
[19] within SIT  within SIT  within SIT  within SIT  within SIT  within SIT
[25] within SIT  within SIT  within SIT  within SIT  within SIT  within SIT
[31] within SIT  within SIT  within SIT  within SIT  within SIT  within SIT
[37] within SIT  within SIT  within SIT  within SIT  within SIT  within SIT
[43] within SIT  within SIT  within SIT  within SIT  within SIT  within SIT
[49] within SIT  within SIT  within SIT  within SIT  within SIT  within SIT
[55] within SIT  within SIT  within SIT  within SIT  within SIT  within SIT
```

```
 [61] within SIT  within SIT  within SIT  within SIT  within SIT  within SIT
 [67] within SIT  within SIT  within SIT  within SIT  within SIT  within SIT
 [73] within SIT  within SIT  within SIT  within SIT  within SIT  within SIT
 [79] within SIT  within SIT  within SIT  within SIT  within SIT  within SIT
 [85] within SIT  within SIT  within SIT  within SIT  within SIT  within SIT
 [91] within SIT  within SIT  within SIT  within SIT  within SIT  within SIT
 [97] within SIT  within SIT  within SIT  within SIT  outside SIT outside SIT
[103] outside SIT outside SIT outside SIT outside SIT outside SIT outside SIT
[109] outside SIT outside SIT outside SIT outside SIT outside SIT outside SIT
[115] outside SIT outside SIT outside SIT outside SIT outside SIT outside SIT
[121] outside SIT outside SIT outside SIT outside SIT outside SIT outside SIT
[127] outside SIT outside SIT outside SIT outside SIT outside SIT outside SIT
[133] outside SIT outside SIT outside SIT outside SIT outside SIT outside SIT
[139] outside SIT outside SIT outside SIT outside SIT outside SIT outside SIT
[145] outside SIT outside SIT outside SIT outside SIT outside SIT outside SIT
[151] outside SIT outside SIT outside SIT outside SIT outside SIT outside SIT
[157] outside SIT outside SIT outside SIT outside SIT outside SIT outside SIT
[163] outside SIT outside SIT outside SIT outside SIT outside SIT outside SIT
[169] outside SIT outside SIT outside SIT outside SIT outside SIT outside SIT
[175] outside SIT outside SIT outside SIT outside SIT outside SIT outside SIT
[181] outside SIT outside SIT outside SIT outside SIT outside SIT outside SIT
[187] outside SIT outside SIT outside SIT outside SIT outside SIT outside SIT
[193] outside SIT outside SIT outside SIT outside SIT outside SIT outside SIT
[199] outside SIT outside SIT
Levels: within SIT outside SIT
```

```r
## makes sense
```

```r
## Model matrix
W.predictions.outside.sit <- predict(gamm.tot.females.Losone,
                                     newdata = d.pred.gamm_aug.Losone.25.SIT.outside,
                                     type = "lpmatrix",

                                     ## Prediction at population level
                                     exclude = 's(ID_BG_trap.fac)')

## Calculate the contrasts

## First, count the rows of the matrix containing only "within SIT"
n3 <- d.pred.gamm_aug.Losone.25.SIT.outside %>%
  filter(zona.SIT.discrete.fac == "within SIT") %>%
  nrow() %>%
  print()
```

```
[1] 100
```

```r
##
## check that "outside SIT" has the same dimension
d.pred.gamm_aug.Losone.25.SIT.outside %>%
  filter(zona.SIT.discrete.fac == "outside SIT") %>%
  nrow()
```

```
[1] 100
```

```r
## makes sense

## Calculate the estimates of the contrasts, starting from the model matrix.
```

```r
## Proceed as follows: first calculate L, then multiply it by beta.
## L.outside.Losone * beta = (X_outside SIT - X_withinSIT) * beta
L.outside.Losone  <- W.predictions.outside.sit[(n3 + 1):(2*n3), , drop = FALSE] -
  W.predictions.outside.sit[1:n3, , drop = FALSE]
est.outside.Losone <- as.vector(L.outside.Losone %*% c.coeff.gamm.tot.females.Losone)

## Now, we calculate the corresponding standard error
se.outside.Losone  <-
  ( (L.outside.Losone %*% vcov.c.coeff.gamm.tot.females.Losone) * L.outside.Losone ) %>%
  rowSums() %>%
  sqrt()

d.diff.outside.Losone <- data.frame(
  yday = d.pred.gamm_aug.Losone$yday[1:n3],
  diff = est.outside.Losone,
  lo = est.outside.Losone - qnorm(0.975) * se.outside.Losone,
  hi = est.outside.Losone + qnorm(0.975) * se.outside.Losone
) %>%
  mutate(exp.diff = exp(diff),
         exp.lo = exp(lo),
         exp.hi = exp(hi))
```

We plot the result.

```r
ggplot(d.diff.outside.Losone, mapping = aes(x = yday, y = diff)) +
  geom_ribbon(aes(ymin = lo, ymax = hi), fill = "lightgray", alpha = 0.5) +
  geom_line(linewidth = 1) +
  geom_hline(yintercept = 0, linetype = "dashed") +
  geom_vline(xintercept = yday("2025-06-17"), linetype = "dashed", colour = "grey") +
  labs(title = "Losone",
       y = "outside SIT minus treated zone")
```

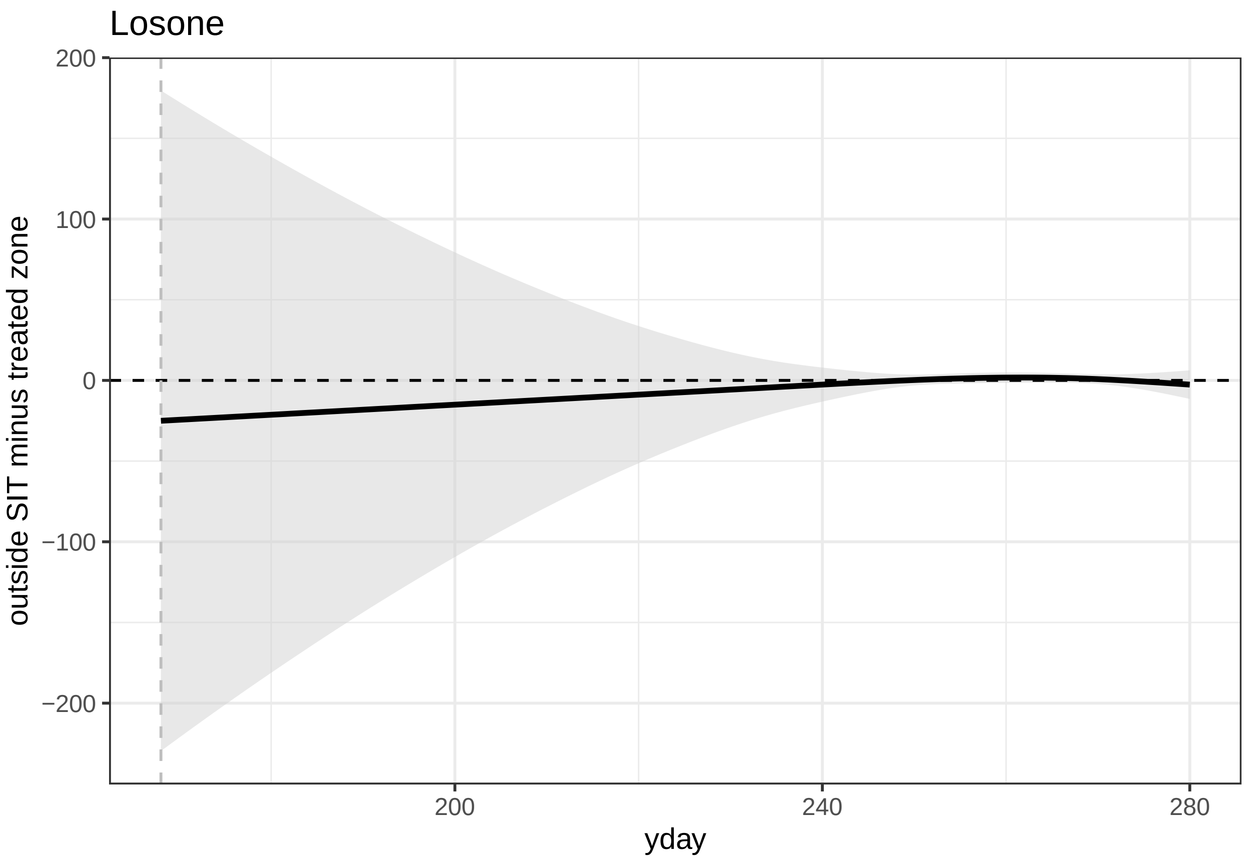

The plot shows the difference between the predicted values on the linear predictor scale for the untreated and treated zones, along with a 95% confidence interval. A horizontal dashed line at 0 indicates no difference between the two zones, while a vertical dashed line marks the start of the treatment on June 17, 2025.

We back-transform the difference by applying the exponential function to return it to the original scale. After this transformation, the values can be interpreted as ratios of predicted female counts: a value of 1 indicates no difference between zones, values greater than 1 indicate higher predicted female counts in the untreated zone relative to the treated zone, and values below 1 indicate the opposite.

```
gg.outside.Losone <- ggplot(d.diff.outside.Losone, mapping = aes(x = yday, y = exp.diff)) +
  geom_ribbon(aes(ymin = exp.lo, ymax = exp.hi), fill = "lightgray", alpha = 0.5) +
  geom_line(linewidth = 1) +
  geom_hline(yintercept = 1, linetype = "dashed") +
  geom_vline(xintercept = yday("2025-06-17"), linetype = "dashed", colour = "grey") +
  labs(title = "Losone",
       y = "outside SIT minus treated zone \nin the back transformed scale")
gg.outside.Losone
```

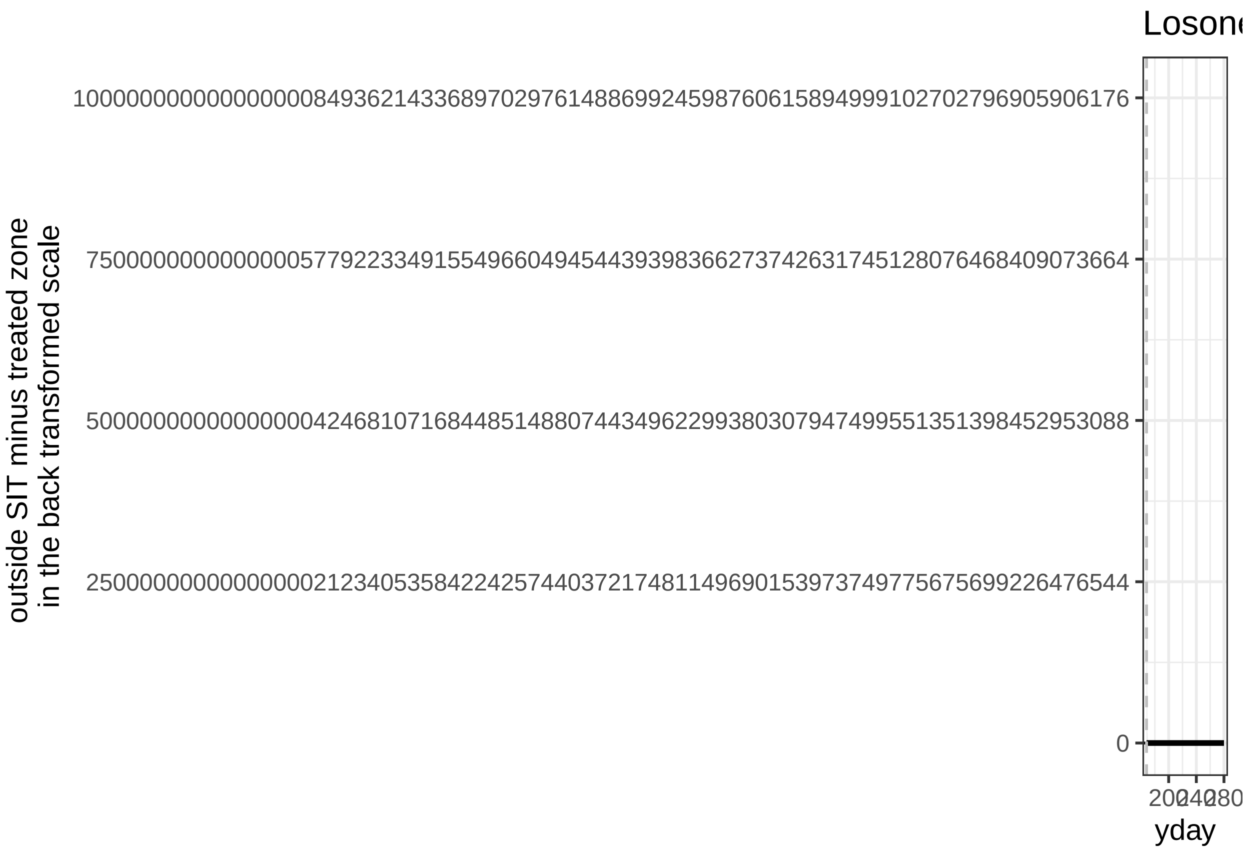

We zoom in to concentrate on the most relevant part of the graph. We use the same zoom as for Losone.

```
## we zoom in
gg.outside.Losone +
  coord_cartesian(ylim = c(0, 10))
```

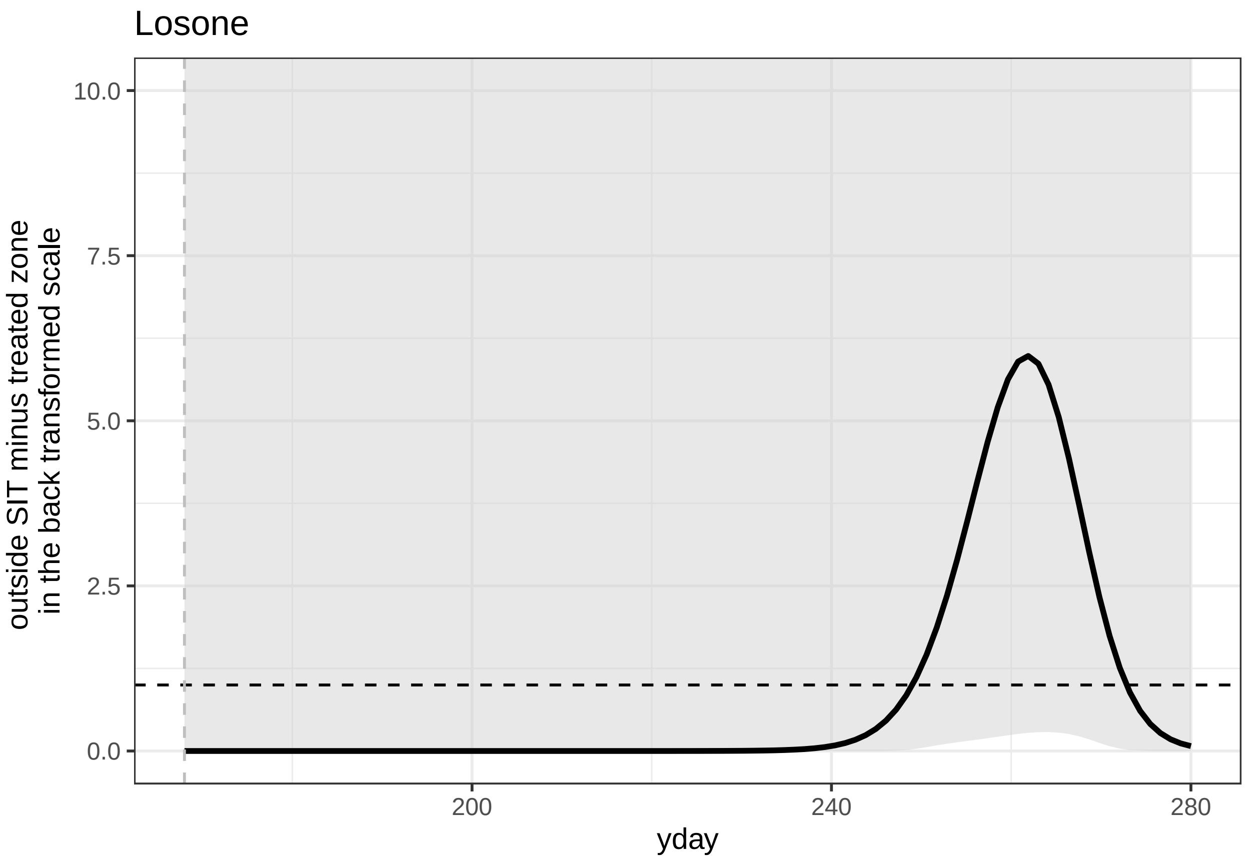

Predicted female counts are generally higher in the treated zone than in the untreated zone throughout the season. This difference remains stable until approximately day 240, when it reverses and a peak is observed in the untreated zone. Following this peak, the difference reverses again. Nevertheless, substantial uncertainty surrounds these estimates, and it is not statistically significant at any point during the season.

5.7 Residual analysis

First of all, we apply the `gam.check()` function to the model, which produces some diagnostic information.

```
par(mfrow = c(2, 2))
gam.check(gamm.tot.females.Losone)
```

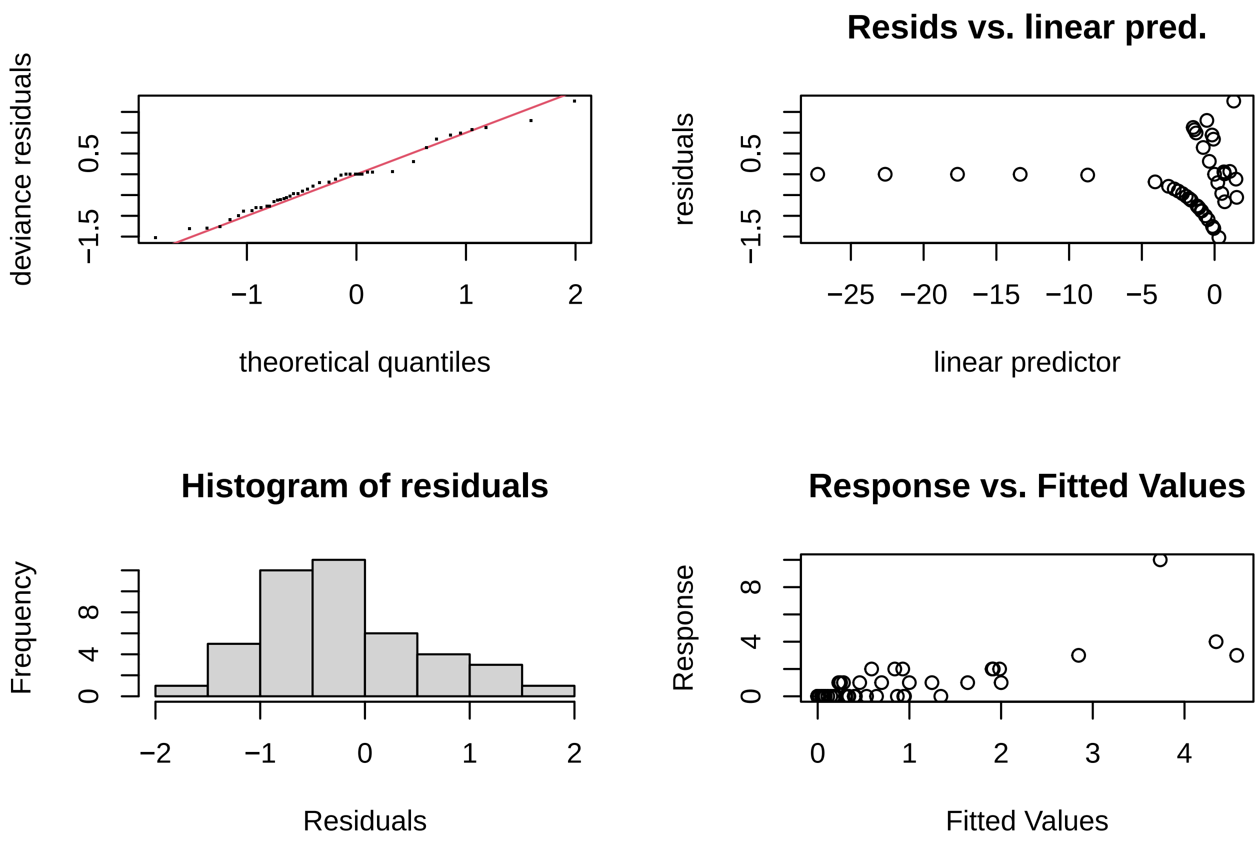


```
Method: REML   Optimizer: outer newton
full convergence after 6 iterations.
Gradient range [-0.0000000111253463941363,0.0000000141725693542094]
(score 43.7494272841916 & scale 1).
Hessian positive definite, eigenvalue range [0.0131683121561565,0.725411052336639].
Model rank =  32 / 32

Basis dimension (k) checking results. Low p-value (k-index<1) may
indicate that k is too low, especially if edf is close to k'.

                                          k'  edf k-index p-value
s(ID_BG_trap.fac)                       5.00 1.47      NA      NA
s(yday):zona.SIT.discrete.facwithin SIT  8.00 1.41    1.04    0.74
s(yday):zona.SIT.discrete.facbuffer      8.00 1.73    1.04    0.69
s(yday):zona.SIT.discrete.facoutside SIT 8.00 2.45    1.04    0.73
```

```r
par(mfrow = c(1, 1))

par(mfrow = c(2, 2))
gam.check(gamm.tot.females.Ascona)
```

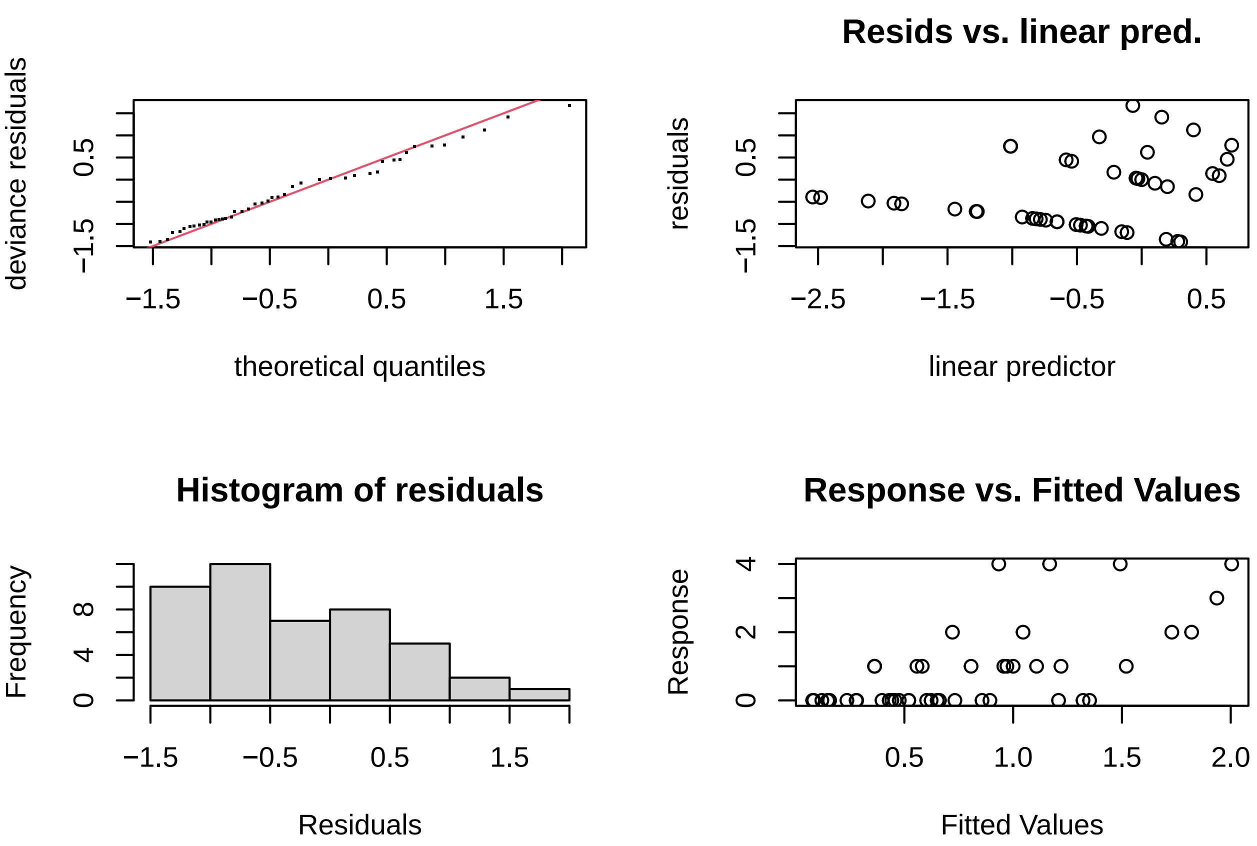


```
Method: REML   Optimizer: outer newton
full convergence after 4 iterations.
Gradient range [-0.0000159668795662871,-0.000000303884447050429]
(score 55.6753131728402 & scale 1).
Hessian positive definite, eigenvalue range [0.09076160314421,1.18294952540333].
Model rank =  23 / 23

Basis dimension (k) checking results. Low p-value (k-index<1) may
indicate that k is too low, especially if edf is close to k'.

                                          k'  edf k-index p-value
s(ID_BG_trap.fac)                       5.00 2.05      NA      NA
s(yday):zona.SIT.discrete.facwithin SIT 8.00 1.96     0.9    0.56
s(yday):zona.SIT.discrete.facbuffer     8.00 3.26     0.9    0.56
```

```
par(mfrow = c(1, 1))
```

Then, we store the pearson residuals in the original data frame, and we plot the residuals against the fitted values to see whether there is still structure in the data.

```
d.adults.Losone$resid_gamm.tot.eggs <- resid(gamm.tot.females.Losone,
                                             type = "pearson")
##
ggplot(data = d.adults.Losone,
       mapping = aes(y = resid_gamm.tot.eggs,
                     x = fitted_gamm.hatching)) +
  geom_hline(yintercept = 0) +
```

```r
  geom_point(alpha = 0.2) +
  geom_smooth()
```

```
`geom_smooth()` using method = 'loess' and formula = 'y ~ x'
```

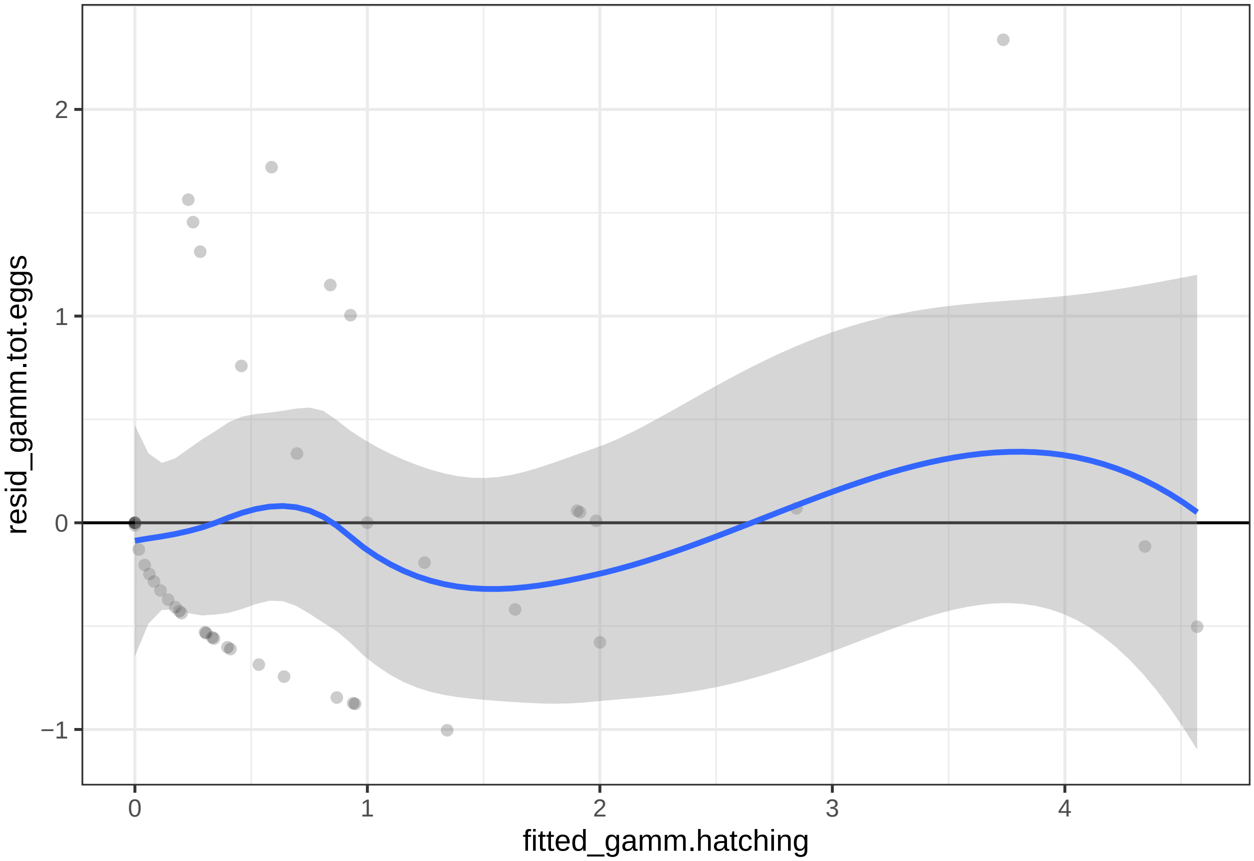

The blue line lies close to the x-axis, indicating that there appears to be little remaining structure in the residuals. Moreover, the 95% confidence band includes zero.

```r
d.adults.Ascona$resid_gamm.tot.eggs <- resid(gamm.tot.females.Ascona,
                                             type = "pearson")
##
ggplot(data = d.adults.Ascona,
       mapping = aes(y = resid_gamm.tot.eggs,
                     x = fitted_gamm.hatching)) +
  geom_hline(yintercept = 0) +
  geom_point(alpha = 0.2) +
  geom_smooth()
```

```
`geom_smooth()` using method = 'loess' and formula = 'y ~ x'
```

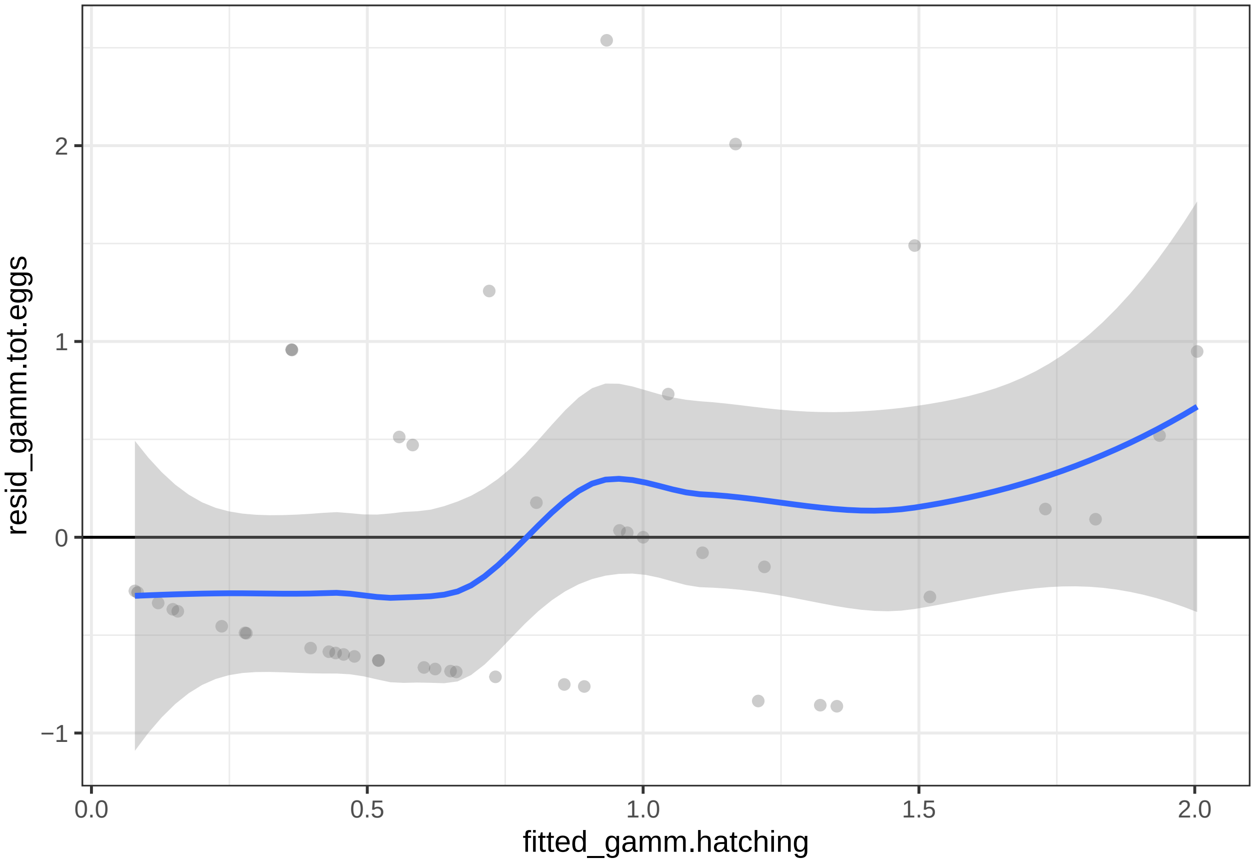

The blue line lies close to the x-axis, indicating that there appears to be little remaining structure in the residuals. Moreover, the 95% confidence band includes zero.

We plot the qq-plot for the random effects.

```
## QQ for random effects
plot(sm(gamm.tot.females.Losone, 1))
```

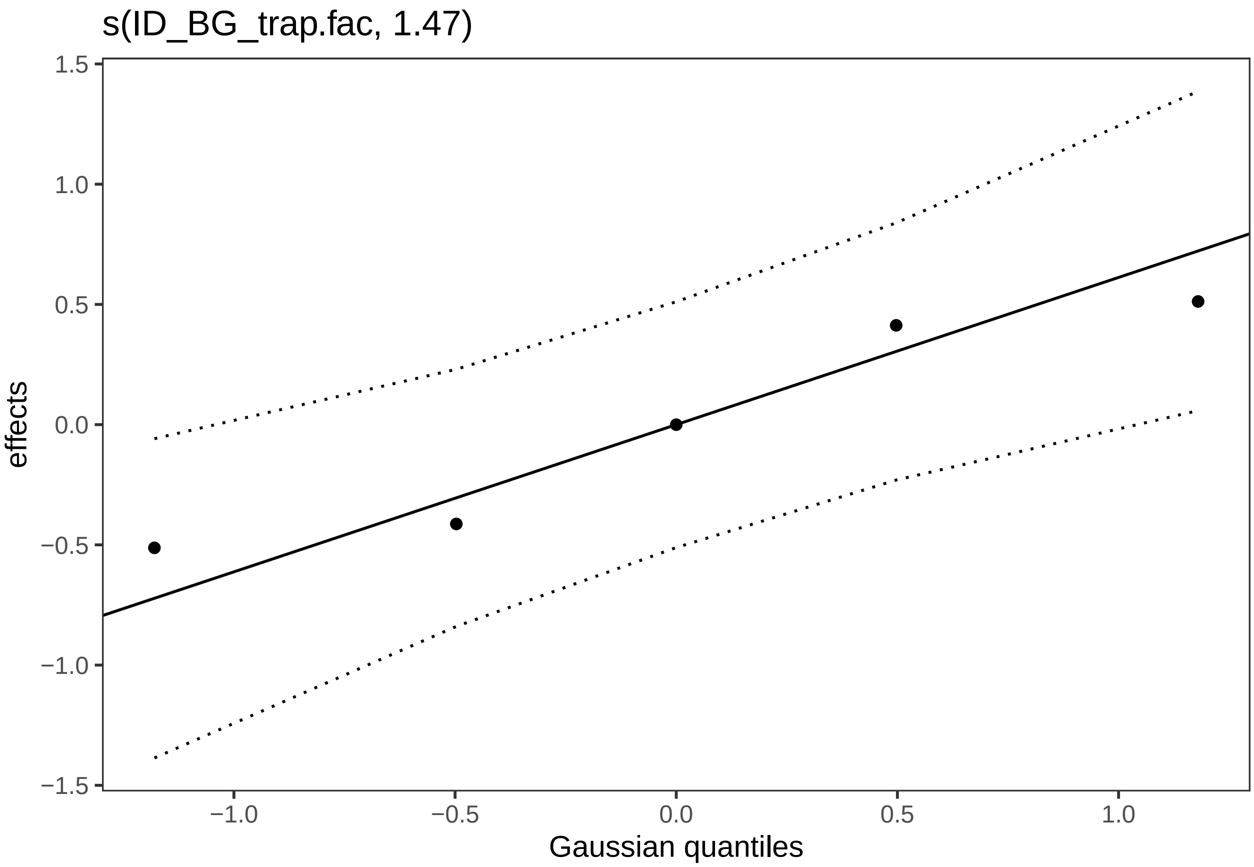


```
##
plot(sm(gamm.tot.females.Ascona, 1))
```

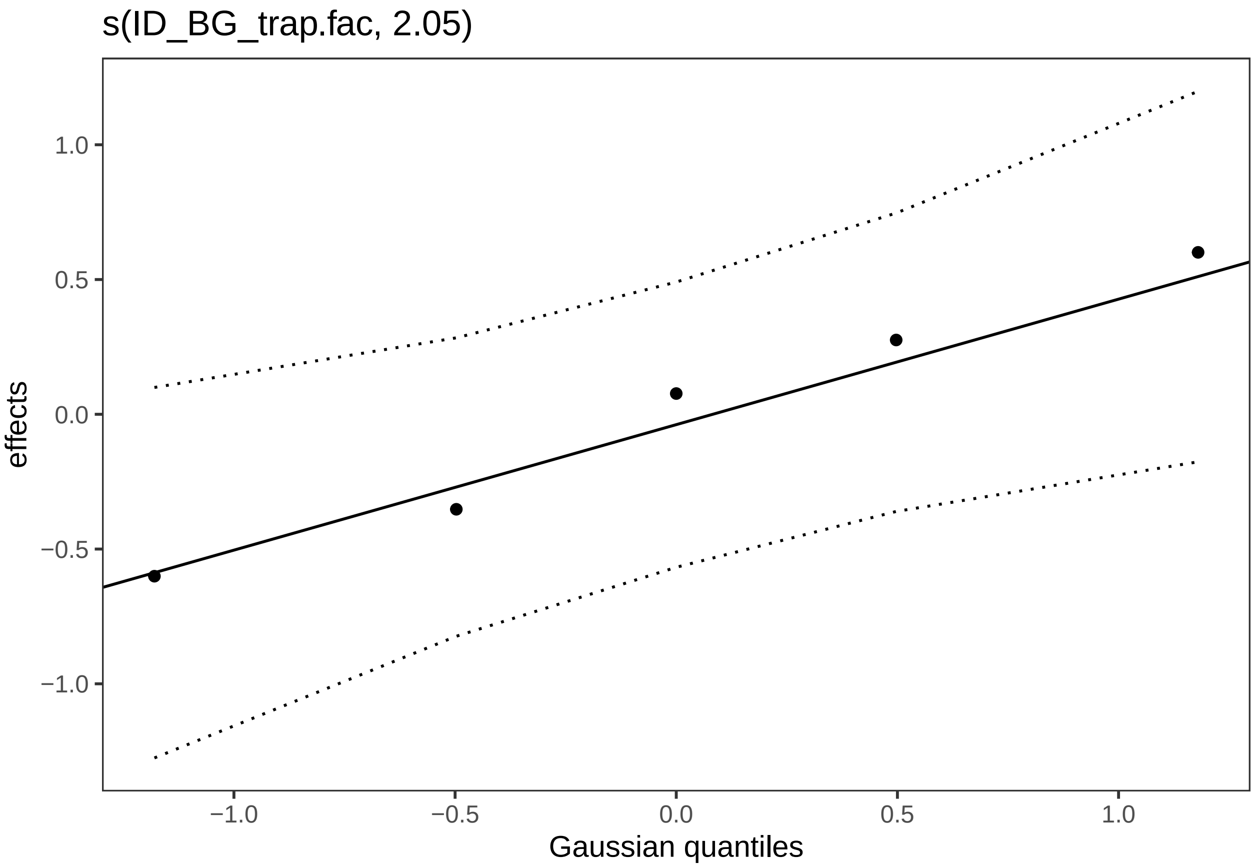

There doesn't seem to be a significant departure from the normality assumption for either models.

We now plot the residuals over time to further check whether there is any structure left in the data.

```
ggplot(data = d.adults.Losone,
       mapping = aes(y = resid_gamm.tot.eggs,
                     x = yday)) +
  geom_hline(yintercept = 0) +
  geom_point(alpha = 0.2) +
  geom_smooth(method = "loess")
```

```
`geom_smooth()` using formula = 'y ~ x'
```

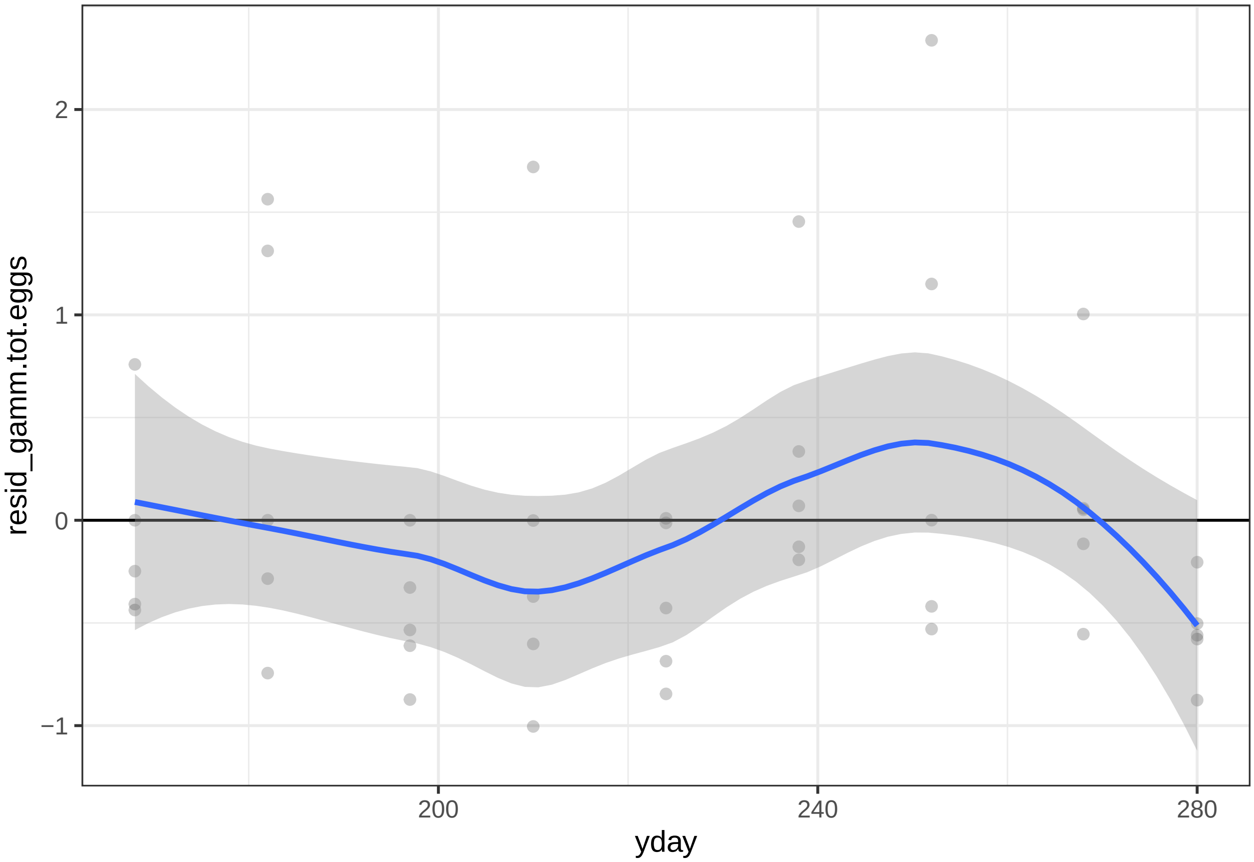


```
##
ggplot(data = d.adults.Ascona,
       mapping = aes(y = resid_gamm.tot.eggs,
                     x = yday)) +
  geom_hline(yintercept = 0) +
  geom_point(alpha = 0.2) +
  geom_smooth(method = "loess")
```

```
`geom_smooth()` using formula = 'y ~ x'
```

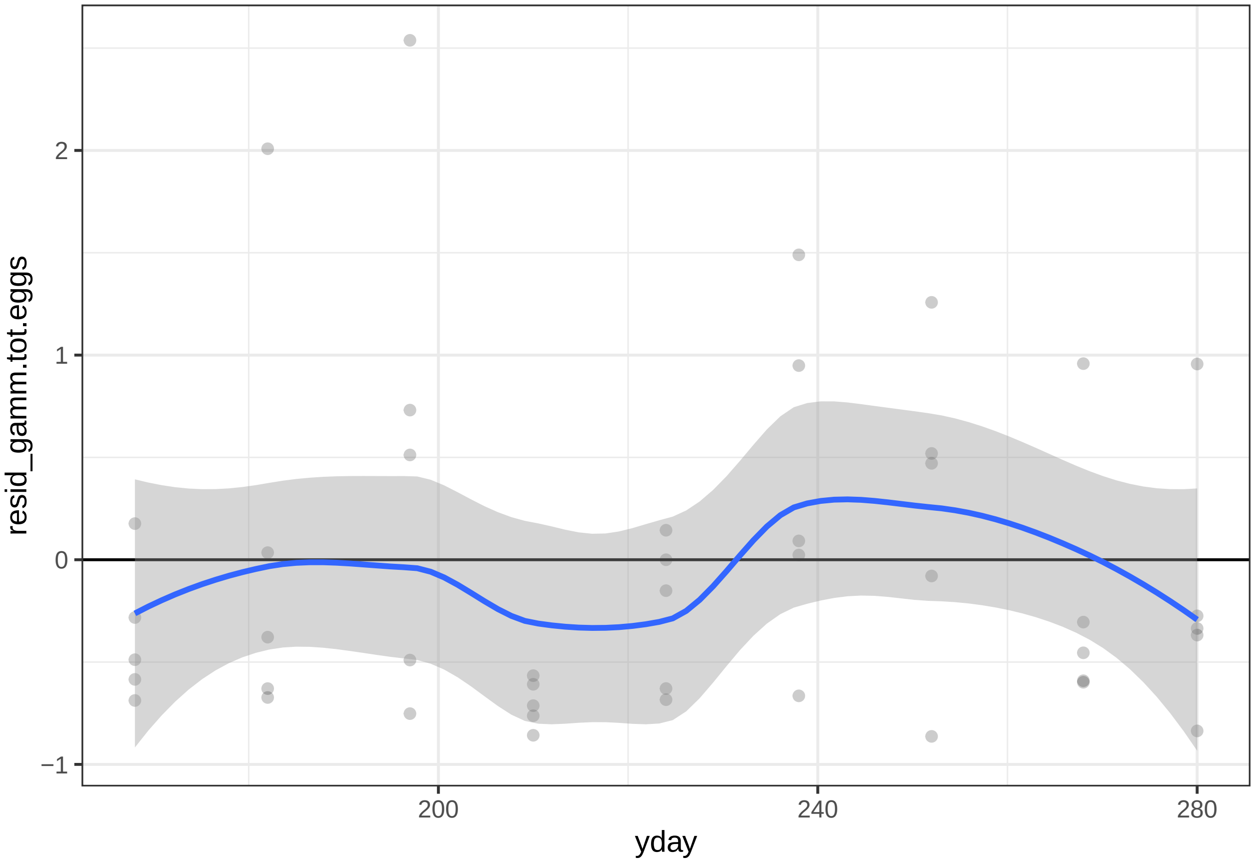

There does not seem to be any structure left in the data.

The following two plots display the residuals in Losone and Ascona, respectively.
The observations belonging to the same ovitrap are connected with a line. These plots are additionally drawn to check temporal correlation and variability.

```
## for Losone
ggplot(data = d.adults.Losone,
       mapping = aes(y = resid_gamm.tot.eggs,
                     x = yday,
                     group = unique.ID)) +
  geom_hline(yintercept = 0) +
  geom_point(alpha = 0.2) +
  geom_line(alpha = 0.2) +
  labs(title = "Losone")
```

Losone

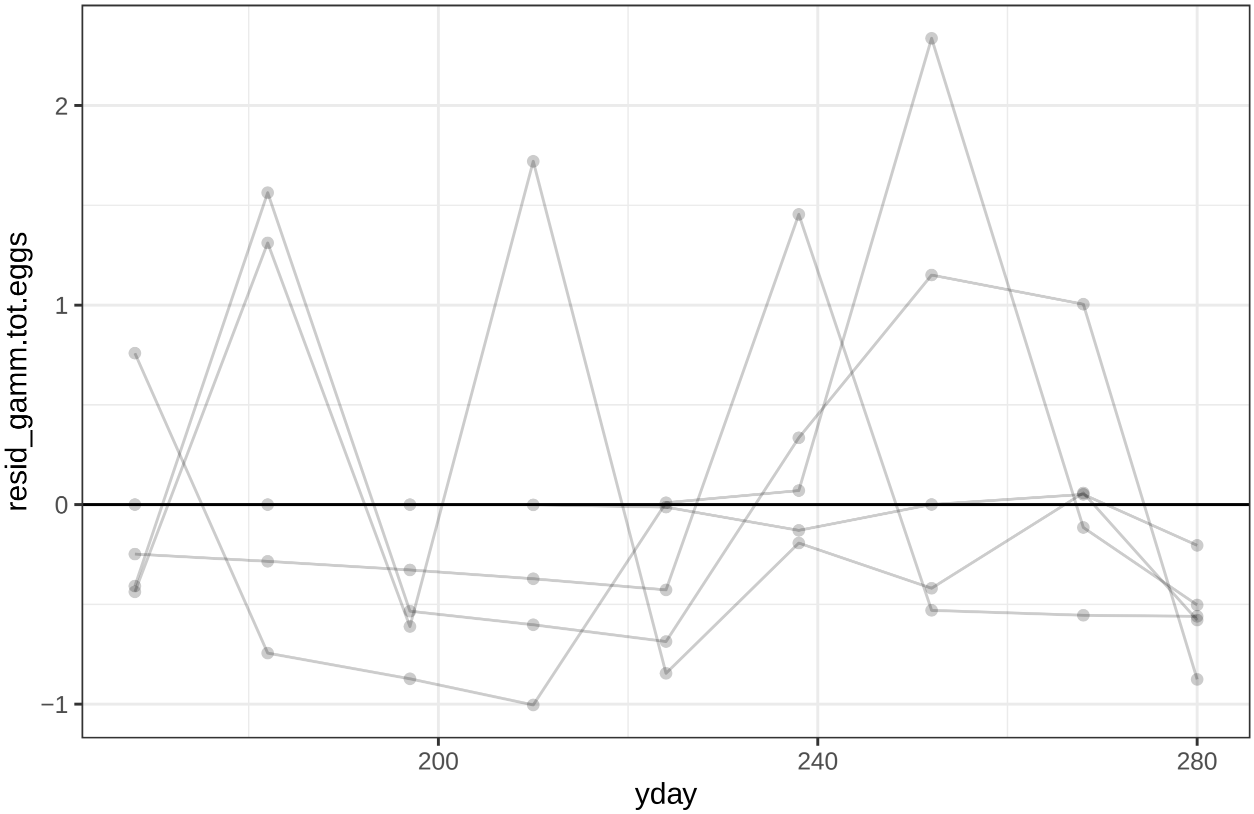


```
##
## For Ascona
ggplot(data = d.adults.Ascona,
       mapping = aes(y = resid_gamm.tot.eggs,
                     x = yday,
                     group = unique.ID)) +
  geom_hline(yintercept = 0) +
  geom_point(alpha = 0.2) +
  geom_line(alpha = 0.2) +
  labs(title = "Ascona")
```

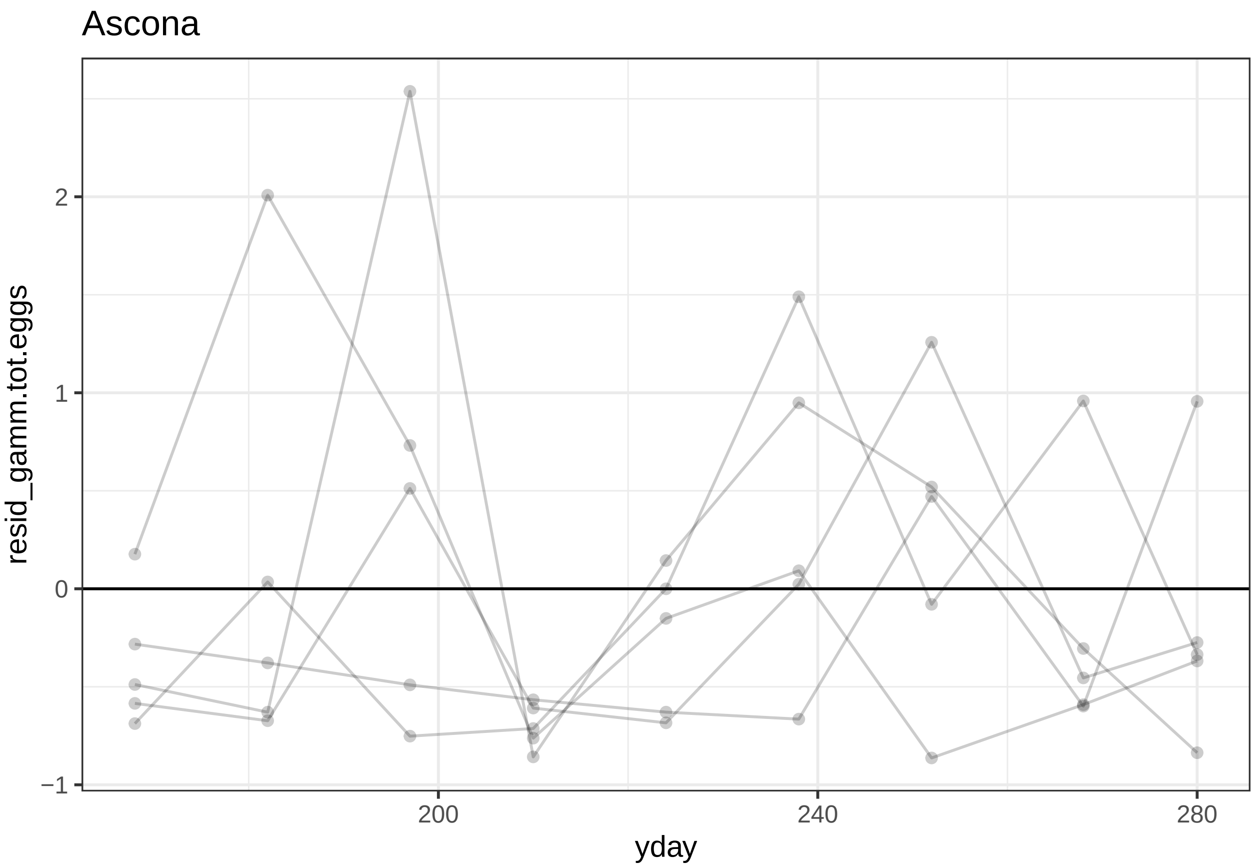

There doesn't seem to be any structure left in the data.

Let's look at the single ovitraps.

```
## for Losone
ggplot(data = d.adults.Losone,
       mapping = aes(y = resid_gamm.tot.eggs,
                     x = yday,
                     group = unique.ID)) +
  geom_hline(yintercept = 0) +
  geom_point() +
  geom_line() +
  facet_wrap(~ unique.ID) +
  theme(
    strip.background = element_blank(),
    strip.text.x = element_blank()) +
  coord_cartesian(ylim = c(-4,4)) +
  labs(title = "Losone")
```

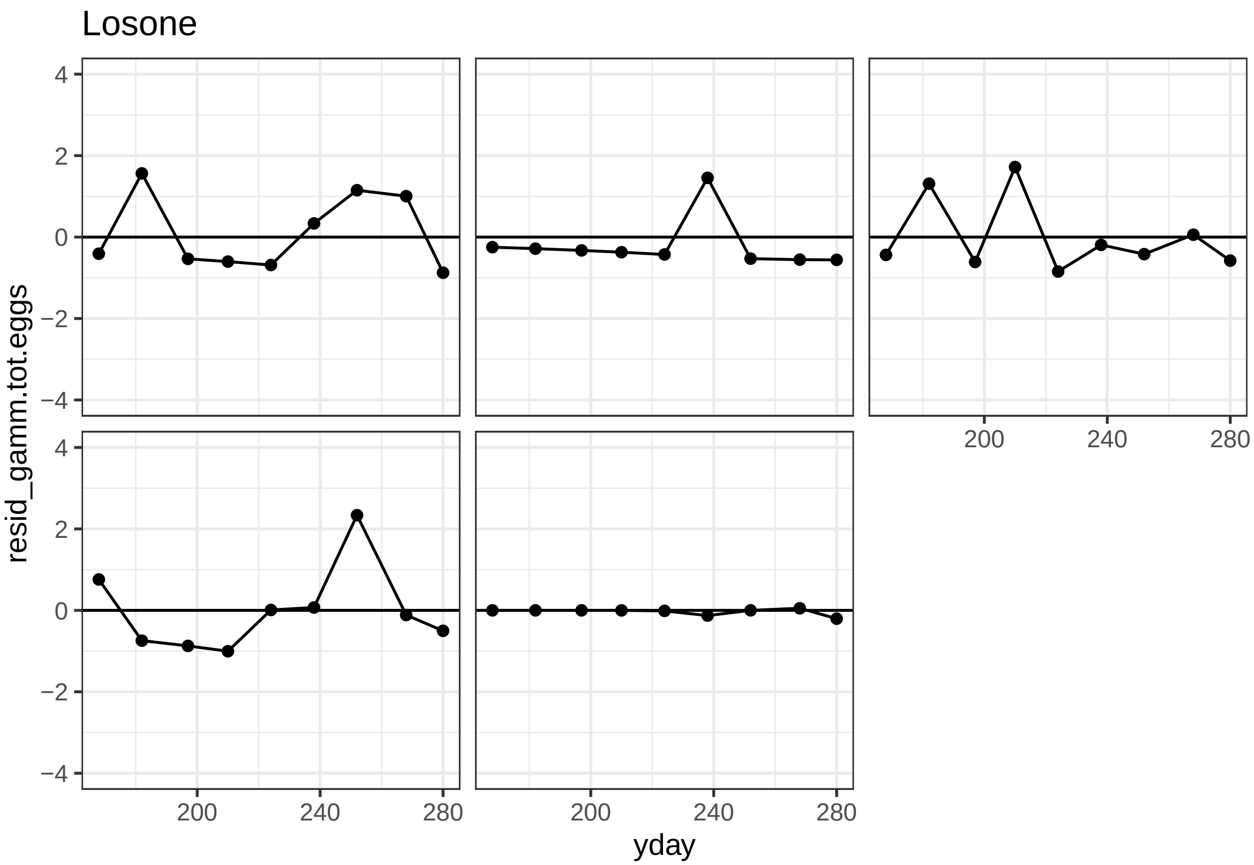


```
##
## for Ascona
ggplot(data = filter(d.adults.Ascona,
                     municipality.fac == "Ascona"),
       mapping = aes(y = resid_gamm.tot.eggs,
                     x = yday,
                     group = unique.ID)) +
  geom_hline(yintercept = 0) +
  geom_point() +
  geom_line() +
  facet_wrap(~ unique.ID) +
  theme(
    strip.background = element_blank(),
    strip.text.x = element_blank()) +
  coord_cartesian(ylim = c(-4, 4)) +
  labs(title = "Ascona")
```

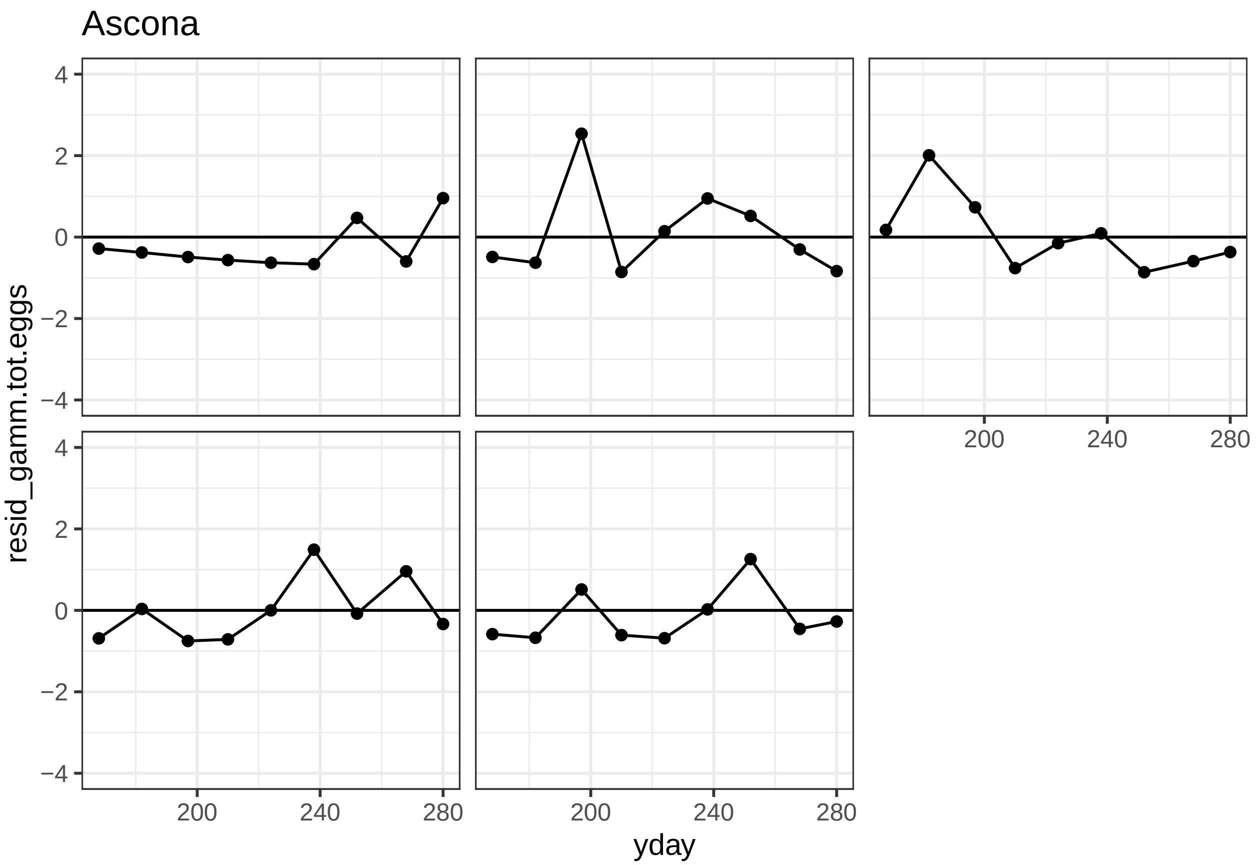

In both cases, there does not seem to be much structure left in the data.

6 Methods

We modelled the response variable “total number of Ae. albopictus female” (*Ae.albopictus.female*) using a Generalised Additive Mixed Model (GAMM) with a negative binomial distribution to account for overdispersion. The model included a smooth term for sampling date, expressed as day of the year (*yday*), and we allowed this smooth to vary by treatment zone (*zona.SIT.discrete.fac*), resulting in zone-specific smooth functions. Treatment zone, a categorical variable with three levels, was included as a fixed effect. To account for repeated measurements of the same trap, we included trap identity (*ID_BG_trap.fac*) as a random intercept.

This modelling procedure was applied separately for the municipalities of Ascona and Losone.

Model complexity was evaluated using Likelihood-Ratio (Chi-square) tests as well as AIC and BIC. All analyses were performed in `R` using the packages `{mgcv}` and `{mgcViz}`, with statistical significance assessed at the 5% level. For additional details, see Section 5 of the Appendix.

@Diego: You can find the citations for the packages in the section "References"

7 Results

All models converge without numerical issues. The proportion of deviance explained is 63.9 % for Losone and 37.5 % for Ascona, indicating a satisfactory fit for both municipalities.

Model comparison shows that the more complex models do not improve model performance. In particular, the models including the smooth–by–treatment interaction do not provide a significantly better fit than the ones without the interaction (Likelihood-Ratio Chi-square test, p= 0.417331926013942 for Losone, p= 0.106825977194743 for Ascona). This indicates that allowing for treatment-specific smooth functions does not capture additional structure in the data.

Furthermore, including a fixed effect for the treatment zone does not improve model fit (p= 0.228236269753985 for Losone, p= 0.907412281081743 for Ascona), indicating that there is insufficient evidence to support the presence of a SIT effect between zones.

However, the lack of significant effects should be interpreted with caution, as the limited amount of available data may reduce the statistical power to detect treatment differences.

8 Conclusions

The analyses conducted do not reveal a clear effect of the SIT treatment on female Ae. albopictus abundance. This result should be interpreted in light of several constraints of the current dataset, including the generally low female counts, the limited number of BG traps, and the absence of both historical data and a pre-treatment baseline. These limitations (stemming from the fact that adult monitoring was costly and therefore only a limited number of BG traps could be deployed) reduce the statistical power and the ability to detect potential treatment effects.

Exploratory comparisons among municipalities indicate that Ascona and Losone (treated) exhibit similar seasonal trajectories, with female counts remaining low until mid-season, whereas Caslano (untreated) shows an earlier increase (see Figure XYZ). However, without data from previous years, it is not possible to determine whether these differences reflect an effect of the SIT intervention or natural spatial–temporal variability. Furthermore, in both Ascona and Losone, buffer and untreated zones display patterns comparable to the treated areas, providing no clear evidence of differences attributable to the treatment.

Overall, while preliminary patterns can be described, the current monitoring framework does not allow robust inference on SIT efficacy. Future evaluations would benefit from earlier and more often monitoring, increased

trap density, and integration of multi-year reference data to strengthen the analytical capability and reduce uncertainty.

@Diego: For the paper, we suggest stating that this analysis does not provide evidence in favour of a SIT effect. However, this lack of evidence is likely attributable to the limitations described above. Moreover, the absence of evidence should not be interpreted as evidence of absence. For this reason, we recommend not presenting the analyses in detail in the main text, but instead explaining that we did not have enough data to find evidence of a SIT effect, and that they can find the full report in the appendix. You may also add that the available data are limited because this was conceived as a pilot study. Monitoring adult populations is costly, and the initial goal was to assess whether significant results could already be detected from the egg data. Had this been the case, we would then have justified a larger investment in adult monitoring. Conversely, it would not have been sensible to allocate substantial resources to female data collection without first establishing whether detectable effects were present in the egg data.

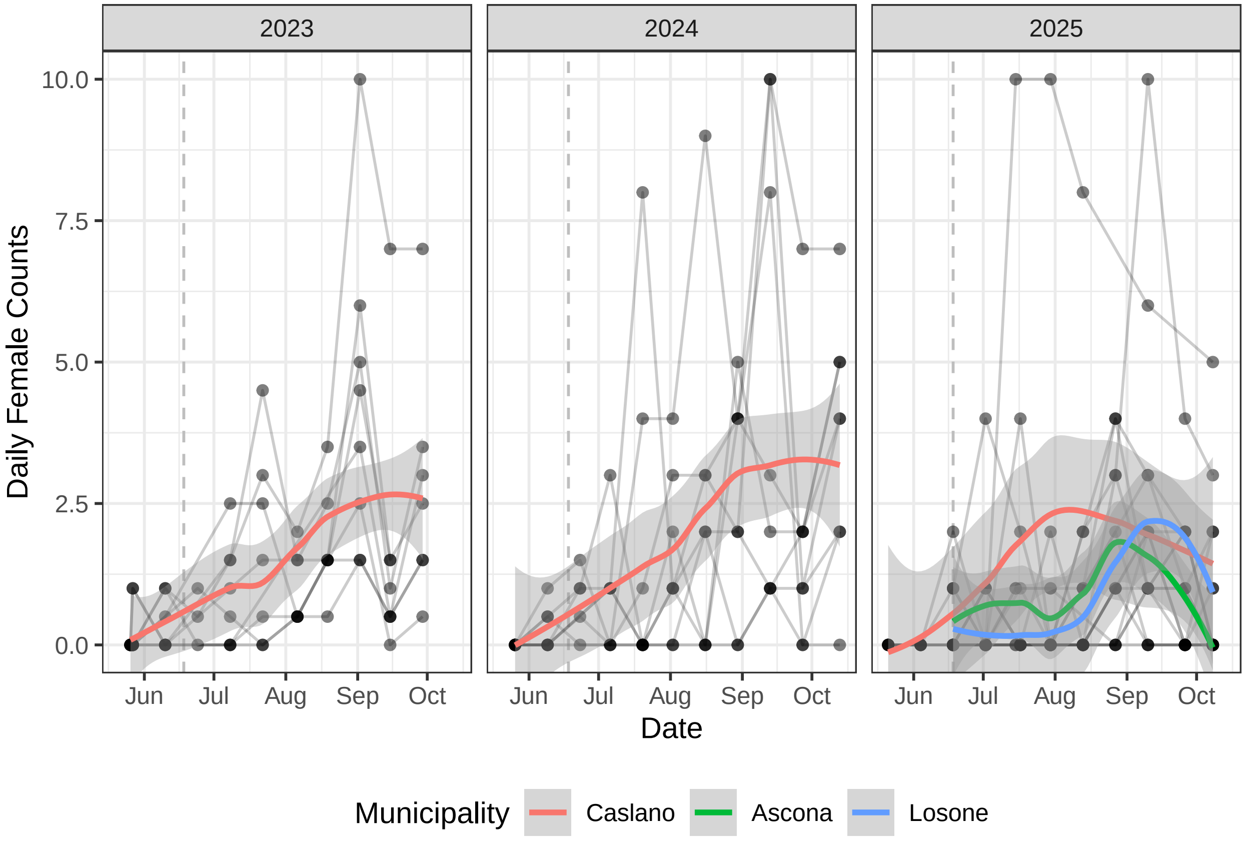

Figure 1: This figure shows daily female counts over time in 2023 (left), 2024 (middle), and 2025 (right). Grey points represent individual observations, and observations from the same BG trap are connected with a line. On top of these lines are three additional coloured lines that represent the smoothed average for each municipality: blue for Losone, red for Caslano, and green for Ascona. Note that data for Losone and Ascona are available only for 2025, and therefore are not shown for the previous years. The dashed vertical line on 17.06.2025 indicates the start of the treatment. In 2025, Losone and Ascona display a similar pattern, with low counts until mid-season, whereas Caslano shows an earlier increase.

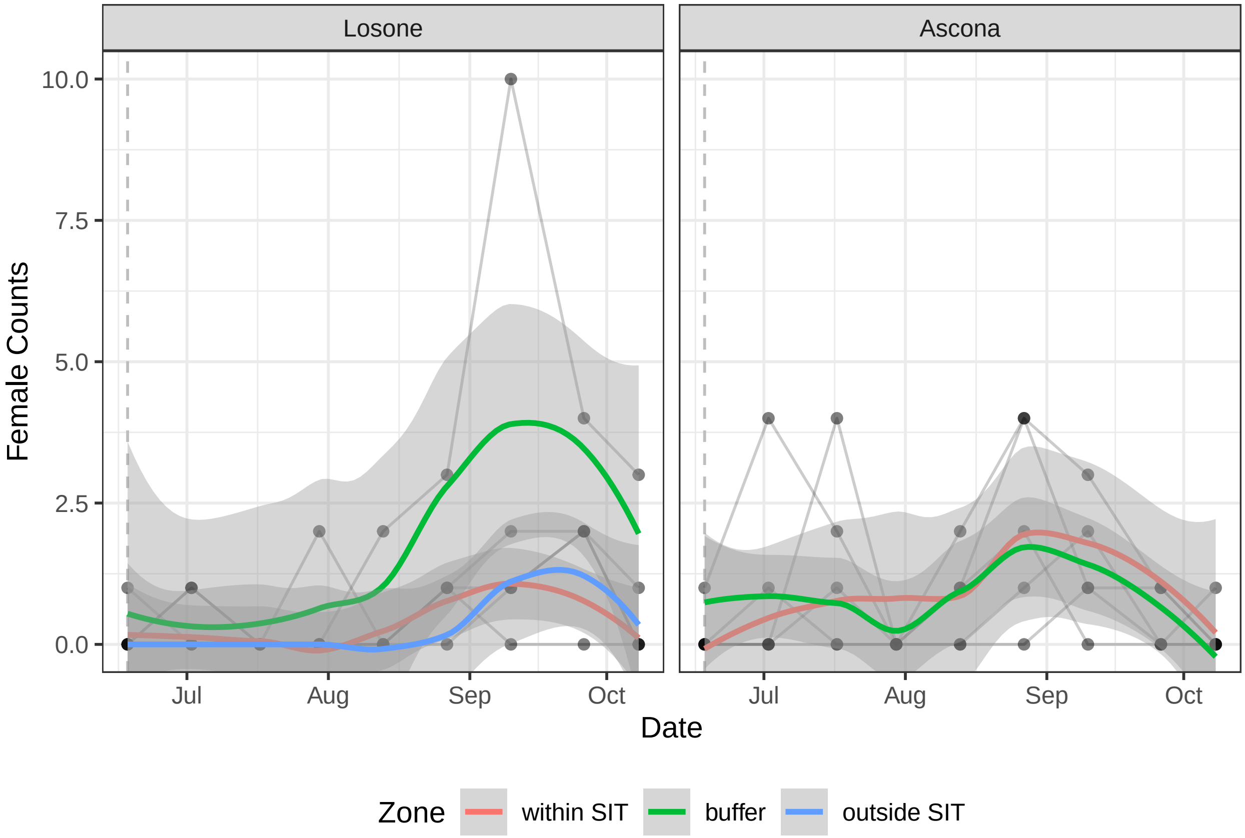

Figure 2: This figure shows female counts over time for Losone (left) and Ascona (right). Grey points represent individual observations, and observations from the same BG trap are connected with a line. On top of these lines are three additional coloured lines that represent the smoothed average for each zone: blue for the untreated zone, red for the treated zone, and green for the buffer zone. The dashed vertical line at 17.06.2025 marks the start of the treatment. In both municipalities, female counts follow a similar temporal pattern across the three zones.

9 References

```r
## Packages
citation("mgcViz")
```

```
To cite the mgcViz package in publications use:

  Fasiolo, M., Nedellec, R., Goude, Y. and Wood, S.N., 2020. Scalable
  visualization methods for modern generalized additive models. Journal
  of computational and Graphical Statistics, 29(1), pp.78-86.

A BibTeX entry for LaTeX users is

  @Article{,
    title = {Scalable visualisation methods for modern Generalized Additive Models.},
    journal = {Journal of the Royal Statistical Society (B)},
    volume = {29},
    number = {1},
    pages = {78-86},
```

```
    year = {2020},
    author = {{Fasiolo} and {Matteo} and {Nedellec} and {Rapha{"e}l} and {Goude} and {Yannig} and {Wood
  }

As mgcViz is often updated, you may want to cite its version number.
Find it with 'help(package=mgcViz)'.
```

```r
citation("mgcv")[[1]] ## return several papers
```

```
Wood, S.N. (2011) Fast stable restricted maximum likelihood and
marginal likelihood estimation of semiparametric generalized linear
models. Journal of the Royal Statistical Society (B) 73(1):3-36

A BibTeX entry for LaTeX users is

  @Article{,
    title = {Fast stable restricted maximum likelihood and marginal
likelihood estimation of semiparametric generalized linear models},
    journal = {Journal of the Royal Statistical Society (B)},
    volume = {73},
    number = {1},
    pages = {3--36},
    year = {2011},
    author = {S. N. Wood},
    doi = {10.1111/j.1467-9868.2010.00749.x},
  }
```

```r
##
## R itself
citation()
```

```
To cite R in publications use:

  R Core Team (2025). _R: A Language and Environment for Statistical
  Computing_. R Foundation for Statistical Computing, Vienna, Austria.
  <https://www.R-project.org/>.

A BibTeX entry for LaTeX users is

  @Manual{,
    title = {R: A Language and Environment for Statistical Computing},
    author = {{R Core Team}},
    organization = {R Foundation for Statistical Computing},
    address = {Vienna, Austria},
    year = {2025},
    url = {https://www.R-project.org/},
  }

We have invested a lot of time and effort in creating R, please cite it
when using it for data analysis. See also 'citation("pkgname")' for
citing R packages.
```
