# Supplementary material for: Assessing the Sterile Insect Technique in Small, Non-Isolated Urban Areas: A Field Trial Against Aedes albopictus in Southern Switzerland
Source: Insects. 2026 Jul 31;17(8):793. doi: 10.3390/insects17080793 (PMC13513938; doi:10.3390/insects17080793)
Supplement: Supplementary file 1 [file insects-17-00793-s001.zip › Table_S1.pdf]

Table S1. Operational data for each sterile male release event conducted during the 2025 SIT trial in Ascona and Losone, including shipment success, transport duration, post-transport mortality, estimated numbers of viable males released, and release rates per hectare. N/A: not applicable.

Release event	Calendar week	Date of shipment[1]	Total number males sent	Date of arrival and release	Time of arrival	Duration of transport [hours]	Time of release	Mortality of male after transport [%]	Total viable males released (Ascona and Losone sites)	Total viable males released at each site	Release rate per hectare (viable males)	Release rate per hectare per week (viable males)
1	25	16.06.2025	56,000	17.06.2025	13:40	24	15:00	7.03	52,063	26,032	2,169	
2	25	19.06.2025	48,000	19.06.2025	10:30	3	10:30	2.37	46,896	23,448	1,954	4,123
3	26	23.06.2025	56,000	24.06.2025	14:30	24	15:30	11.83	49,392	24,696	2,058	
4	26	26.06.2025	56,000	27.06.2025	12:00	24	14:00	12.33	49,112	24,556	2,046	4,104
5	27	30.06.2025	54,000	01.07.2025	12:00	24	13:20	13.37	46,818	23,409	1,951	
6	27	02.07.2025	54,000	03.07.2025	13:30	24	14:00	11.17	48,006	24,003	2,000	3,951
7	28	07.07.2025	54,000	08.07.2025	14:00	24	14:30	19.43	43,524	21,762	1,814	
8	28	10.07.2025	54,000	**FAILED**	N/A	N/A	N/A	N/A	N/A	N/A	N/A	1,814
9	29	14.07.2025	60,000	15.07.2025	14:00	24	14:30	7.00	55,800	27,900	2,325	
10	29	17.07.2025	60,000	18.07.2025	14:00	24	14:30	14.80	51,120	25,560	2,130	4,455
11	30	21.07.2025	56,000	23.07.2025	13:45	**48**	14:00	24.53	42,280	21,140	1,762	
12	30	24.07.2025	72,000	**FAILED**	N/A	N/A	N/A	N/A	N/A	N/A	N/A	1,762
13	31	28.07.2025	64,000	29.07.2025	14:00	24	14:30	6.50	59,840	29,920	2,493	
14	31	30.07.2025	53,000	31.07.2025	13:45	24	14:00	17.60	43,672	21,836	1,820	4,313
15	32	04.08.2025	50,000	05.08.2025	13:45	24	14:00	11.30	44,350	22,175	1,848	
16	32	07.08.2025	60,000	08.08.2025	13:45	24	14:00	14.13	51,540	25,770	2,148	3,996
17	33	11.08.2025	56,000	13.08.2025	13:30	**48**	14:00	40.90	33,096	16,548	1,379	
18	33	13.08.2025	60,000	14.08.2025	13:45	24	14:00	12.90	52,260	26,130	2,178	3,557
19	34	18.08.2025	80,000	19.08.2025	14:00	24	14:15	28.67	57,040	28,520	2,377	
20	34	21.08.2025	60,000	22.08.2025	14:00	24	14:20	7.40	55,560	27,780	2,315	4,692
21	35	25.08.2025	56,000	26.08.2025	14:00	24	14:15	23.00	43,288	21,644	1,804	

Release event	Calendar week	Date of shipment[1]	Total number males sent	Date of arrival and release	Time of arrival	Duration of transport [hours]	Time of release	Mortality of male after transport [%]	Total viable males released (Ascona and Losone sites)	Total viable males released at each site	Release rate per hectare (viable males)	Release rate per hectare per week (viable males)
22	35	28.08.2025	60,000	29.08.2025	14:00	24	14:15	27.00	43,980	21,990	1,833	3,637
23	36	01.09.2025	52,000	02.09.2025	14:00	24	14:15	20.00	41,465	20,732	1,728	
24	36	04.09.2025	52,000	05.09.2025	14:00	24	15:00	30.00	36,296	18,148	1,512	3,240
25	37	08.09.2025	48,000	10.09.2025	14:00	**48**	15:00	77.00	10,944	5,472	456	
26	37	11.09.2025	60,000	**FAILED**	N/A	N/A	N/A	N/A	N/A	N/A	N/A	456
27	38	15.09.2025	80,000	16.09.2025	16:00	24	16:00	42.00	46,560	23,280	1,940	1,940
28	39	24.09.2025	56,000	25.09.2025	14:00	24	14:00	61.00	21,840	10,920	910	910
29	40	29.09.2025	80,000	01.10.2025	14:00	**48**	14:30	88.00	9,600	4,800	400	400

[1] Males were always transported by courier, except on 19.06.2025 when they were transported directly by car from the production facility (transportation time of only 3 hours).
