# Supplementary material for: Assessing the Sterile Insect Technique in Small, Non-Isolated Urban Areas: A Field Trial Against Aedes albopictus in Southern Switzerland
Source: Insects. 2026 Jul 31;17(8):793. doi: 10.3390/insects17080793 (PMC13513938; doi:10.3390/insects17080793)
Supplement: Supplementary file 1 [file insects-17-00793-s001.zip › Table_S2.pdf]

Table S2. Detailed sample size information by site, zone, trap network, and sampling period.

Site	Year	Trap network[1]	Zone	No. traps	No. observations
Ascona	2024	OTR-MON	Pre-intervention	25	225
Losone	2024	OTR-MON	Pre-intervention	20	180
Ascona	2025	OTR-SIT	SIT-treated	12	120
Ascona	2025	OTR-SIT	non-SIT buffer	15	150
Ascona	2025	OTR-SIT	non-SIT outside	0	0
Ascona	2025	OTR-MON	SIT-treated	1	10
Ascona	2025	OTR-MON	non-SIT buffer	5	50
Ascona	2025	OTR-MON	non-SIT outside	19	190
Losone	2025	OTR-SIT	SIT-treated	12	120
Losone	2025	OTR-SIT	non-SIT buffer	16	160
Losone	2025	OTR-SIT	non-SIT outside	0	0
Losone	2025	OTR-MON	SIT-treated	1	10
Losone	2025	OTR-MON	non-SIT buffer	3	30
Losone	2025	OTR-MON	non-SIT outside	16	160

[1] OTR-SIT: ovitraps deployed specifically for the SIT trial in 2025. OTR-MON: ovitraps belonging to the routine municipal surveillance network, providing continuous data from both the pre-intervention year (2024) and the intervention year (2025)
